# Azoacetylenes for the Synthesis of Arylazotriazole Photoswitches

**DOI:** 10.1021/jacs.1c06014

**Published:** 2021-09-03

**Authors:** Patrick Pfaff, Felix Anderl, Moritz Fink, Moritz Balkenhohl, Erick M. Carreira

**Affiliations:** Laboratorium für Organische Chemie, ETH Zürich, D-CHAB, Vladimir-Prelog-Weg 3, 8093 Zürich, Switzerland

## Abstract

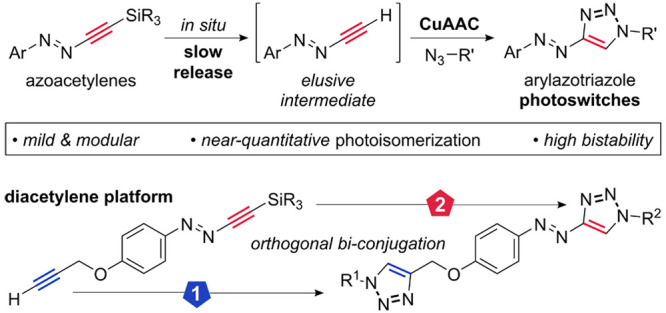

We report a modular approach toward novel arylazotriazole photoswitches and their photophysical characterization. Addition of lithiated TIPS-acetylene to aryldiazonium tetrafluoroborate salts gives a wide range of azoacetylenes, constituting an underexplored class of stable intermediates. *In situ* desilylation transiently leads to terminal arylazoacetylenes that undergo copper-catalyzed cycloadditions (CuAAC) with a diverse collection of organoazides. These include complex molecules derived from natural products or drugs, such as colchicine, taxol, tamiflu, and arachidonic acid. The arylazotriazoles display near-quantitative photoisomerization and long thermal *Z*-half-lives. Using the method, we introduce for the first time the design and synthesis of a diacetylene platform. It permits implementation of consecutive and diversity-oriented approaches linking two different conjugants to independently addressable acetylenes within a common photoswitchable azotriazole. This is showcased in the synthesis of several photoswitchable conjugates, with potential applications as photoPROTACs and biotin conjugates.

The observation of photochromism in the prototypical azobenzene^1^ has inspired the study of photoswitches in diverse research contexts, ranging from materials science^2^ to medicine.^3^ With the emergence of photopharmacology, photoswitchable agents hold the promise to directly impact human health via reversible and spatiotemporal control of drug activity, potentially limiting off-tissue toxicity.^4,5^ Although photoswitches are widely applied in various modern settings, methods for their synthesis largely rely on traditional approaches (Scheme 1A). The development and implementation of practical, convenient synthetic methods can provide access to new photoswitches with desirable photophysical properties, enabling novel applications.

**Scheme 1:**
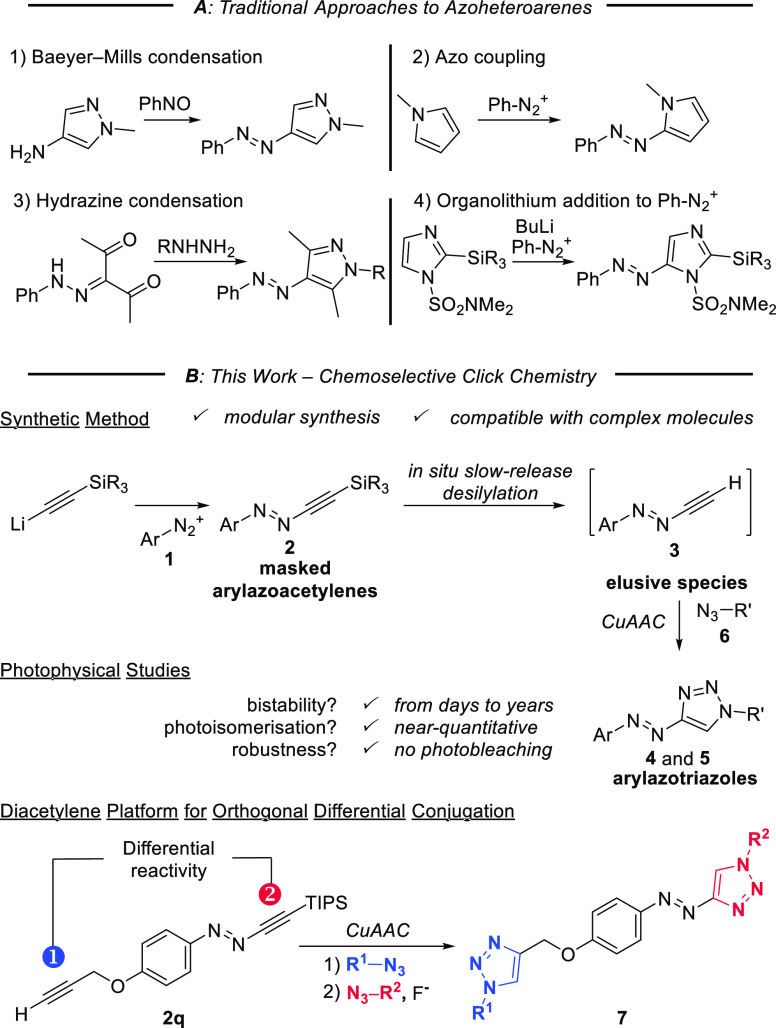
Conventional Approaches and Present Work

Given the success of arylazopyrazoles with near-quantitative photoisomerization and high bistability pioneered by Fuchter,^6,7^ we envisioned that 1,4-substituted arylazotriazoles **4** could possess beneficial photophysical properties (Scheme 1B).^8^ For biological applications, switchable scaffolds are desirable that allow convergent coupling of complex chemical structures.^4,5^ Herein, we report a novel strategy to efficiently access arylazotriazoles **4** in a modular approach that is compatible with introduction of highly functionalized molecules (Scheme 1B).^9^ The azotriazoles described display high bistability (days to years at room temperature), near-quantitative photoisomerization (*E*→*Z*, >98%; *Z*→*E*, >90%), and photostability against bleaching. We further report a diacetylene platform **2q** that enables consecutive coupling of two different complex azides to furnish photoswitchable azotriazole conjugates either in a one-pot protocol or in diversity-oriented divergent two-step procedures.

In recent years, heteroarylazobenzenes have gained considerable attention as photoswitches.^10^ A range of these incorporating pyrazoles,^6^ imidazoles,^11,12^ and thiophenes^13,14^ have been synthesized and photophysically characterized (Scheme 1A). These procedures, reported to work well in simple systems, rely on either condensation reactions,^15−17^ electrophilic aromatic substitution,^18^ or organometal addition to aryldiazonium salts.^12^

Synthetic approaches to arylazotriazoles and their implementation in complex settings relevant to biology require development of mild synthetic methods characterized by chemoselectivity and modularity.^19,20^ A well-established class of reactions that meets these criteria is click chemistry.^21^ Specifically, Cu(I)-catalyzed azide–alkyne cycloadditions (CuAACs) have been widely adopted,^22,23^ and numerous approaches are available for preparation of azides^24−28^ and alkynes.^29−31^

In this context, we sought to develop a general strategy for the synthesis of arylazotriazole switches **4** via CuAAC (Scheme 1B), which would proceed from a common, versatile building block. The parent terminal acetylene **3** is the prototype of a class of compounds that is underexplored^32,33^ and elusive.^34^ As a reactive intermediate, it would have to be generated *in situ* from a masked precursor, as shown for **2**. Inspired by a report by Feringa,^35^ we hypothesized that arylazoacetylenes might be prepared by addition of lithiated alkyne derivatives to aryldiazonium tetrafluoroborates.

Our efforts commenced with attempts to efficiently access masked arylazoacetylenes **2**. Addition of lithiated TMS-acetylene to phenyldiazonium tetrafluoroborate at −78 °C led to the clean formation of phenylazo-TMS-acetylene **2** (Scheme 1B, R = Me, Ar = Ph), which was isolated after aqueous workup. During purification of the material, however, continuous decomposition of the compound was observed (see the Supporting Informatin (SI)). We hypothesized that an increase in steric bulk of the silyl group might lead to improved stability, enabling handling and subsequent use of the azoacetylene.^33^

Addition of lithiated TIPS-acetylene to PhN_2_BF_4_ at −78 °C led to formation of TIPS-protected phenylazoacetylene (**2a**, >99%, Scheme 2A). To our delight, **2a** proved to be thermally stable and was stored for about a year at room temperature without decomposition, as determined by ^1^H NMR. To examine the generality of the protocol, TIPS-protected arylazoacetylenes were prepared, bearing both electron-donating (**2b**–**2d**, **2g**, **2h**, **2m**) and electron-withdrawing substituents (**2e**, **2f**, **2i**–**2l**, **2n**) in 66–99% yield. 2,6-Disubstituted arylazoacetylenes (**2d**–**2g**) were prepared (74–89%) because of the beneficial photophysical properties of the corresponding arylazobenzenes.^36−38^

**Scheme 2:**
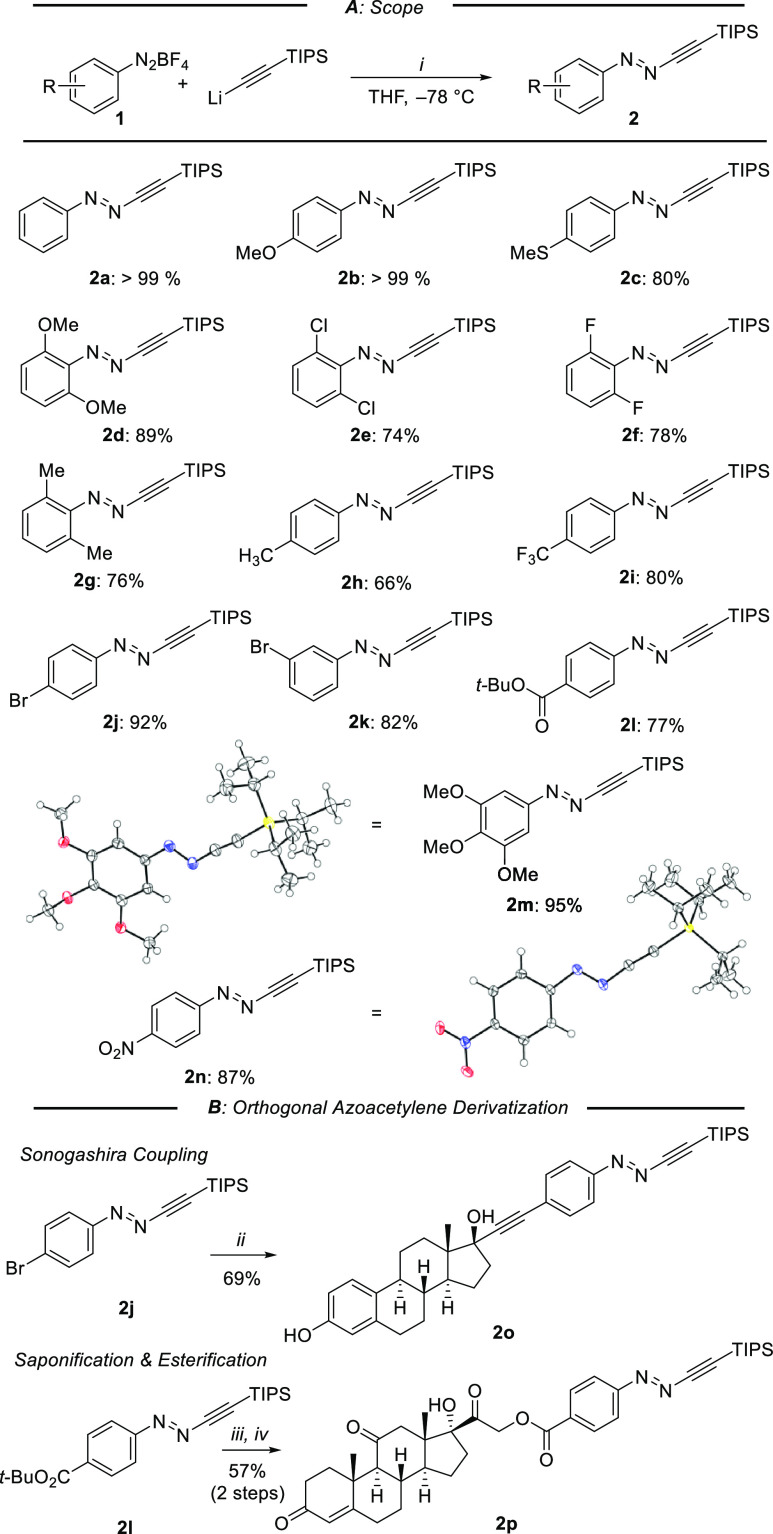
Generation of Arylazoacetylenes and Derivatization Reagents and conditions: (i) Li-TIPS-acetylene (1.0 equiv, 0.6 M, THF–hexane), −78 °C, THF; (ii) ethinylestradiol (1.0 equiv), *i*Pr_2_NH (5.0 equiv), CuI (15 mol%), Pd(*t*-Bu_3_P)_2_ (15 mol%), 45 °C, dioxane–PhMe (5:1); (iii) Me_3_SiOTf (1.05 equiv), 2,6-lutidine (1.50 equiv), 40 °C, CH_2_Cl_2_; (iv) cortisone (1.0 equiv), DMAP (1.0 equiv), EDC (1.1 equiv).

Photochromism was inspected for **2a**, **2b**, and **2i** (see Figures S1–S3 for UV–vis spectra). Thermal half-lives of the (*Z*)-isomers of **2a**, **2b**, and **2i** were determined to be on the order of minutes (Figures S17–S19), with electron-poor **2i** showing the longest half-life (*t*_1/2_ ca. 30 min). Electron-rich **2b** was stable under irradiation, while **2a** and **2i** underwent photobleaching (Figures S81–S83).

To study if the novel arylazoacetylenes are sufficiently robust for derivatization, we examined functionalization reactions on masked azoacetylenes **2j** and **2l** (Scheme 2B). Sonogashira coupling of *p*-bromoazoacetylene **2j** with ethinylestradiol was conducted with (*t*-Bu_3_P)_2_Pd as catalyst,^39^ giving **2o** (69% yield). Alternatively, deprotection of *tert*-butyl ester **2l**(40) allowed subsequent esterification with cortisone, giving **2p** (57% yield, two steps).

With a broad set of TIPS-masked arylazoacetylenes in hand, we turned our attention to development of mild conditions for *in situ* desilylative CuAAC, compatible with functionalized, complex conjugants. Importantly, in biological applications it would be desirable to minimize subsequent onerous manipulations, such as deprotections or oxidation state adjustments, following the click conjugation step.

The thermal lability of terminal azoacetylenes **3** and their potential for dimerization^32,41^ suggested conditions in which their concentration is kept low over the course of the reaction. We reasoned that slow release of **3** from the TIPS precursor would be possible by controlled delivery of fluoride. Transiently produced terminal azoacetylene **3** would then undergo rapid CuAAC (Scheme 1B). Initial attempts toward controlling the supply of fluoride were based on the use of a solid–liquid interface. This involved KF/MeOH and relied on slow dissolution of KF over the course of the reaction.

Examination of the scope for these conditions, however, revealed a lack of generality. Further screening led to the identification of a set of liquid–liquid biphasic conditions (THF–H_2_O (3:1)) with aqueous KF or aqueous CsF/Bu_4_NBr at either rt or 40 °C (for optimization, see Tables T1–T3 in the SI). Collectively, this set of reaction conditions enabled access to a wide range of azotriazoles, including electron-donating and -withdrawing substituents (Scheme 3).

**Scheme 3:**
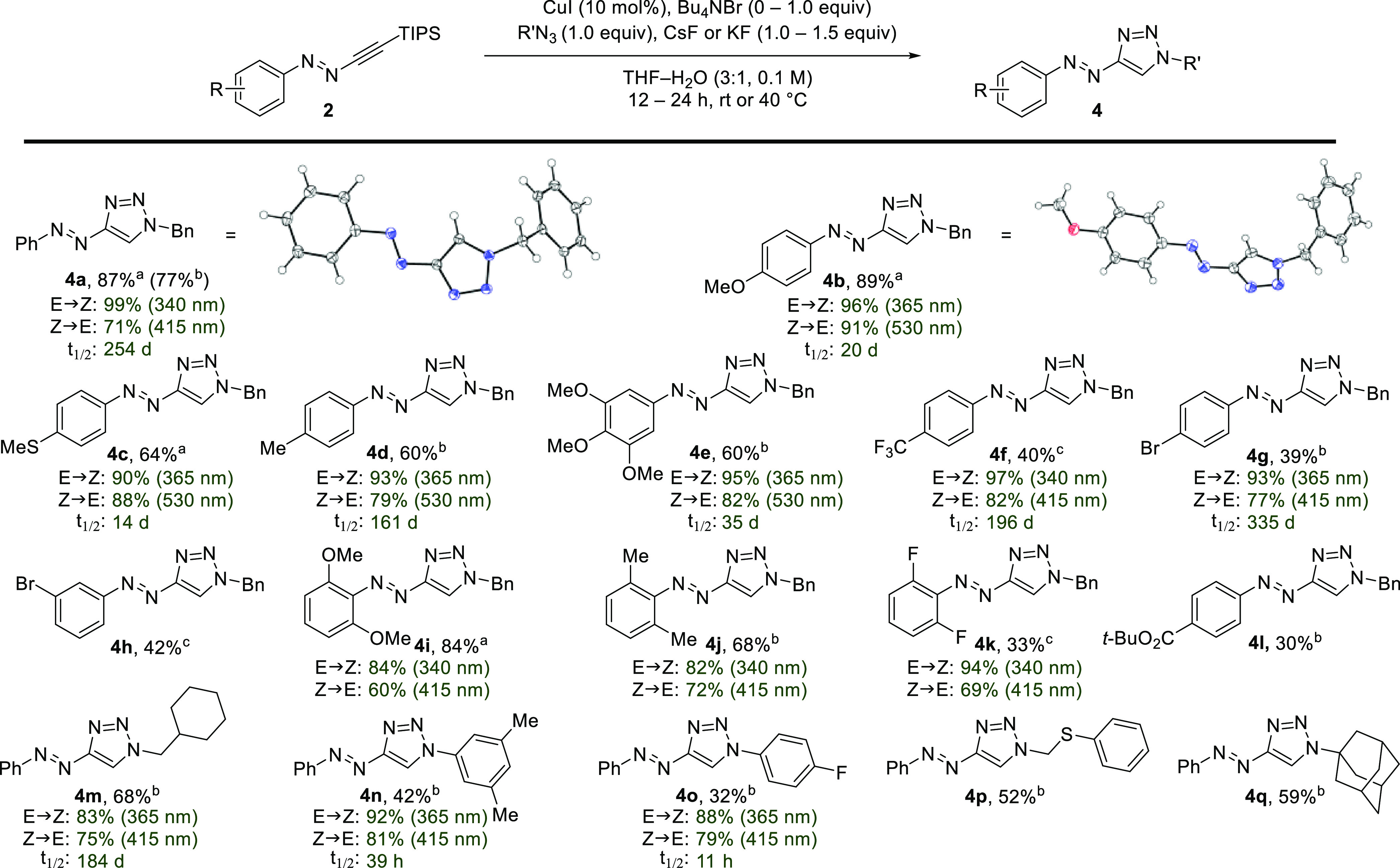
Desilylative CuAAC of Azoacetylenes to give Azotriazoles, and their Photophysical Details Reagents and conditions: (a) CsF (1.0 equiv), Bu_4_NBr (1.0 equiv), 40 °C; (b) CsF (1.0–1.5 equiv), Bu_4_NBr (0.2 equiv), rt; (c) KF (1.0 equiv), rt. Photostationary states were reached after irradiation of samples (100 μM DMSO) for 30 min (340 nm) or 20 min (all other wavelengths).

The safety of nitrogen-rich arylazoacetylenes and arylazotriazoles was assessed by thermal analyses. TGA and DSC measurements revealed slow thermal decomposition over temperature ranges of at least 100 °C, with maximum heat flows below 3 W/g. Further analysis by a conservatively modified set of Yoshida correlations did not hint at shock sensitivity or explosive behavior^42^ (see SI for safety statement and experimental details).

We next systematically studied the photophysical properties of *N-*benzyl-substituted azotriazoles **4a**–**4g** (Scheme 3; for details see SI). As determined by HPLC assay, all displayed high photostationary state (PSS) *Z*-content (>90%) upon irradiation at the π–π* absorption bands. No detectable photobleaching was observed for **4a** and **4b** after several irradiation cycles (Figures S84 and S85). A representative selection of UV–vis spectra for **4a**, **4b**, and **4f** in DMSO is shown in Figure 1. When compared to parent **4a**, compounds bearing electron-donating substituents, as shown for *p*-OMe (**4b**), displayed red-shifted absorption spectra.

**Figure 1:**
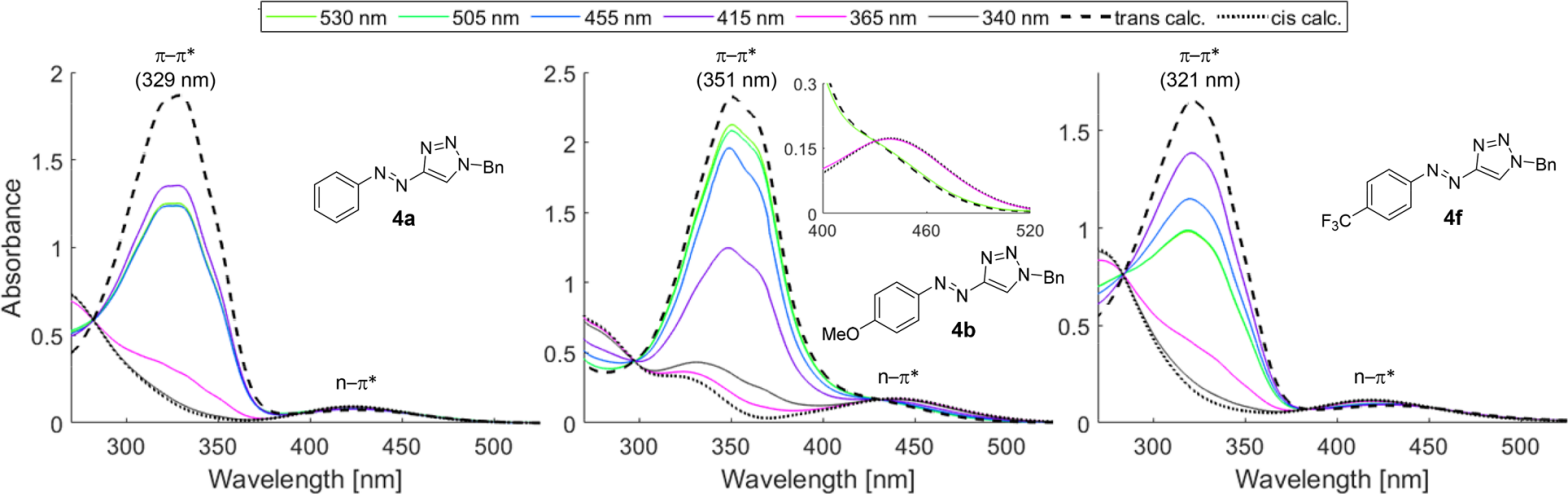
Selected UV–vis spectra of compounds **4a**, **4b**, and **4f** measured in DMSO (100 μM) irradiated for 30 min (340 nm) or 20 min (all other wavelengths).

In connection to this, we observed separation of the n−π* bands of the isomeric pair *E*/*Z*-**4b**. This allowed selective irradiation of the n−π* absorption of *Z*-**4b**, leading to high restoration of the *E*-isomer by irradiation at 530 nm (91%). This is in line with observations made by Li with phenyl ether derivatives of arylazopyrazoles.^43^ Switches incorporating electron-withdrawing substituents, as illustrated for **4f**, elicited less efficient return to the thermodynamic ground state at 530 nm and required irradiation at 415 nm for high *E*-PSS content (82%). 2,6-Disubstituted arylazotriazoles, such as **4i**–**4k**, possessed a slightly reduced *E*/*Z* ratio in the PSS when compared to other analogs.

Subsequently, the thermal half-lives of metastable *Z*-isomers were determined. Electron-rich compounds (**4b**, **4c**, **4e**) possessed *t*_1/2_ in the range of weeks at 25 °C, while parent **4a**, alkylated **4d**, and electron-deficient switches (**4f**, **4g**) displayed higher stability (from 161 to 335 d at 25 °C), making all ideal for applications when high bistability is desired. Bistability was influenced by *N*-bound residues of azotriazoles (**4**, R′, Scheme 3). *N*-Aryl groups (**4n**, **4o**) led to shorter *Z*-half-lives (11–39 h) when compared to *N*-benzyl-substituted **4a** (254 d). Other *N*-alkyl-substituted azotriazoles such as **4m** (184 d) remained in a similar range. Together, these results suggest coupling of arylazoacetylenes incorporating *p*-electron-donating substituents to alkyl azides for optimal photoswitching properties.

In the context of applying this approach to the synthesis of photopharmacological probes, we examined access to photoswitches embedded within functionally rich molecules (Chart 1). We thus generated azotriazole derivatives of carbohydrate glucose (**5a**), antiviral tamiflu (**5b**), lipid arachidonic acid (**5c**), vitamin biotin (**5d**), steroid ethinylestradiol (**5e**), alkaloid colchicine (**5f**), and diterpenoid taxol (**5g**),^44^ which were produced in 46–88% yield. This set of complex molecules comprises functional groups such as alcohols, esters, (thio)ethers, phenols, skipped dienes, ketones, amides, and ureas, demonstrating broad functional group tolerance.

**Chart 1:**
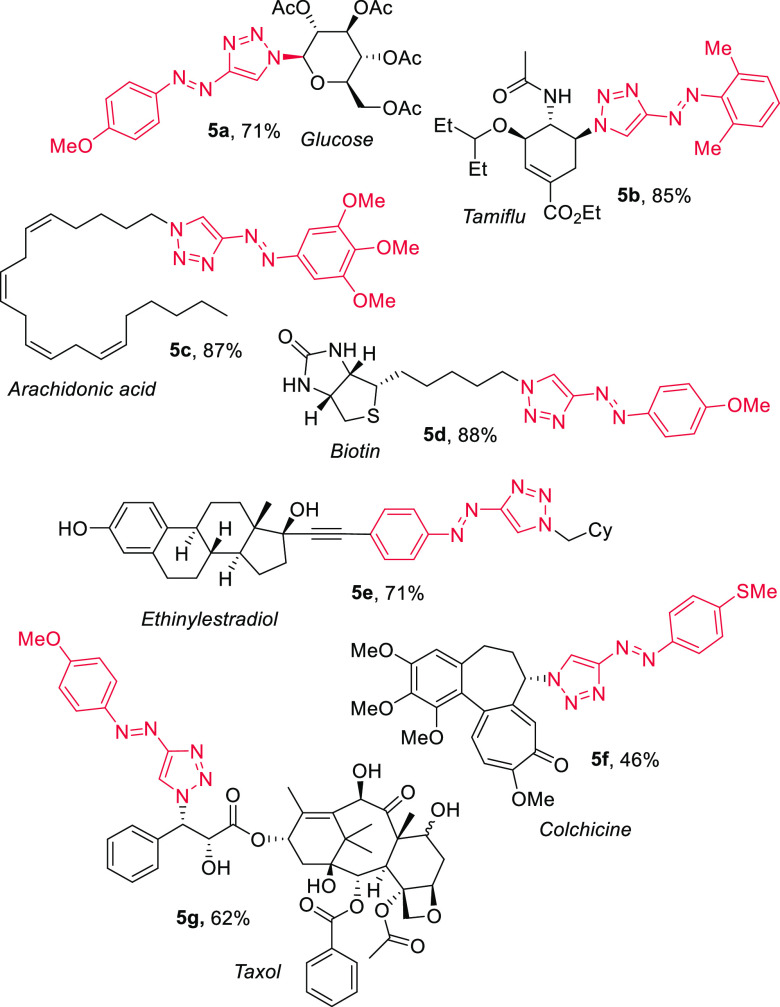
Azotriazoles and Complex Conjugatesa

a Arylazoacetylene (1.0 equiv), azide derivative (1.0 equiv), CsF (1.0 equiv), Bu_4_NBr (1.0 equiv), 40 °C, THF–H_2_O (3:1, 0.1 M).

Conventional conjugation approaches frequently employ amides, esters, or ethers for conjugant attachment to azobenzenes.^4,44,45^ In contrast, the method described herein links the objects of study directly to arylazotriazoles, which can result in shorter topological distances with increased rigidity due to fewer attendant degrees of freedom between conjoined fragments. This holds potential for design of photoswitchable probes with amplified differential biological activity between *cis*- and *trans-*photoisomers.

We showed that functionally rich molecules can be singly introduced onto arylazotriazoles via either azide (**5a**–**5d**, **5f**, **5g**) or arylazoacetylene (**5e**). By extension, this gives entry to bifunctional probes linked by photoswitchable units. We were especially interested in the design of a bis-conjugation platform that would allow streamlined assembly of conjugants using two consecutive click reactions. A common challenge for generation of photoswitchable conjugates is the requirement of two independent sites of linkage and attendant orthogonal, mutually compatible modes of reactivity on either side of photoswitchable actuators.^43,46^ To address this issue, we turned our attention to the development of a diacetylene platform that would allow the execution of two distinctly addressable click reactions.

We wondered whether incorporation of a terminal acetylene onto the TIPS-masked azoacetylene (Scheme 4, **A**) would lead to a bis-conjugation platform in which the former is intrinsically “on” while the latter, by virtue of the masking group, is “off”, allowing each to be sequentially engaged using the same CuAAC reaction mode (Scheme 4). The first coupling partner (R^1^N_3_) would react chemoselectively at the terminal acetylene (**A**→**B**). Following formation of the first cycloadduct, addition of fluoride and a second partner (R^2^N_3_) would then furnish a fully assembled photoswitchable conjugate **D** (**B**→**C**→**D**, Scheme 4). If successful, this approach would not be burdened by additional chemical manipulations. In reducing this plan to practice and due to the beneficial photophysical properties measured for phenyl ether derivatives, a terminal acetylene unit was incorporated as a *p*-propargyl ether, as shown for **2q**, synthesized from 4-propynyloxyphenyl-diazonium tetrafluoroborate (see SI).

**Scheme 4:**
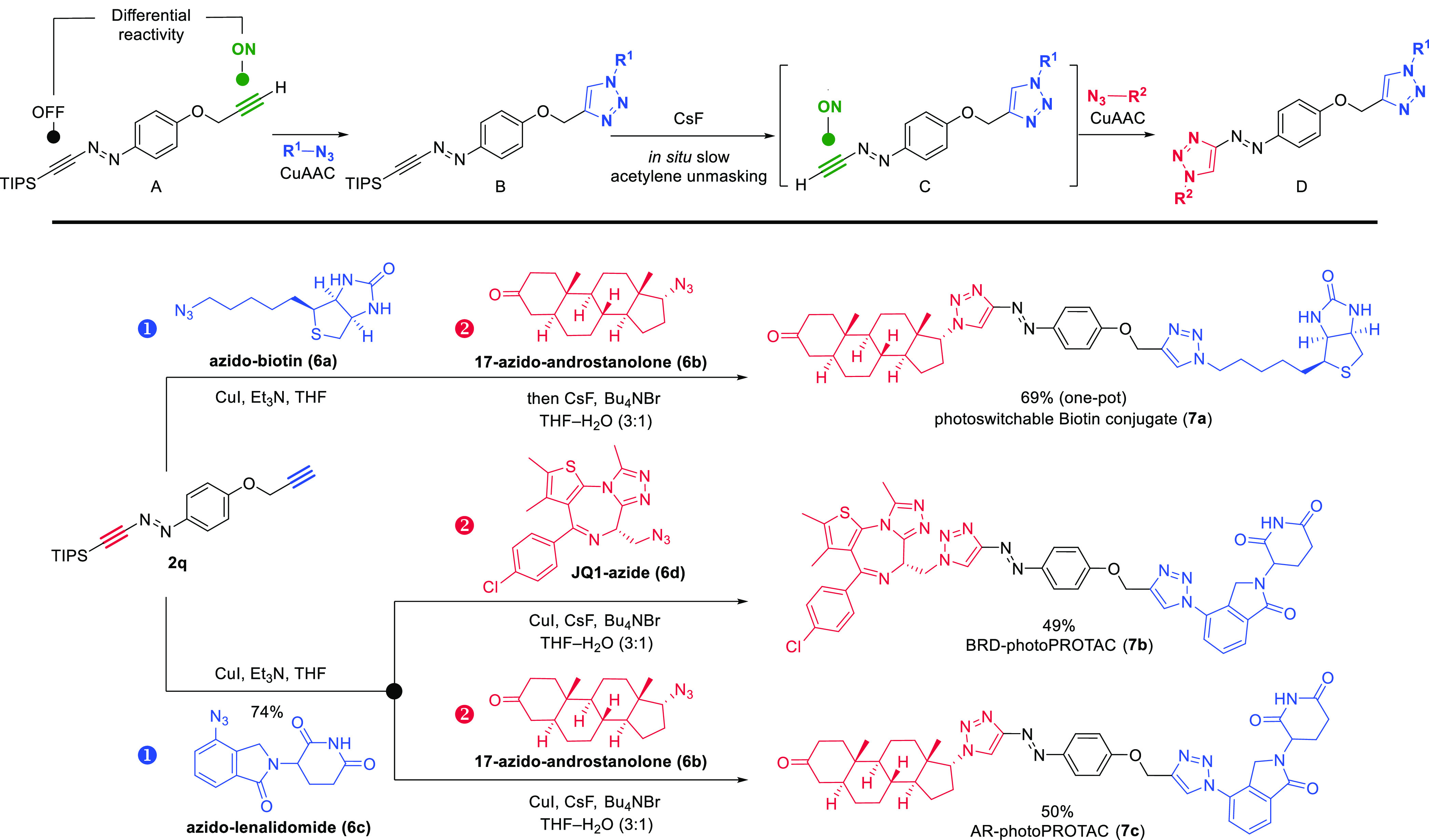
Diacetylene Platform for Consecutive CuAAC Conjugation

We applied this strategy to the generation of a photoswitchable biotin–androstanolone conjugate. Sequential reaction of **2q** with azido-biotin **6a** and—following addition of aqueous CsF—with azido-androstanolone derivative **6b** produced conjugate **7a** in 69% yield in a single-pot operation. Biotin conjugates have ample applications for immobilization of protein targets on streptavidin-coated surfaces. Therefore, photoswitchable biotin conjugates have the potential to reversibly control protein immobilization and translocalization by irradiation.^47^

The inherent versatility of diacetylene **2q** enables diversity-oriented synthesis approaches to conveniently access divergent sets of photoswitchable conjugates. For example, this is desirable in the context of photoswitchable PROTACs (photoPROTACs),^46,48^ in which the order of introduction of the E3 ligase ligand or protein-of-interest (POI) recruiter as part of an optimization process can be chosen at will. The first click reaction then provides a common intermediate which serves as a point of departure for subsequent introduction of a variety of conjugants (different POI or E3 ligase ligands). To illustrate this concept, reaction of **2q** with azido-lenalidomide (**6c**) generated a lenalidomide-linked azoacetylene intermediate (not shown), which was subsequently reacted with either JQ1-azide (**6d**) or azido-androstanolone (**6b**) under the desilylative CuAAC conditions. This gives divergent access to two photoPROTAC candidates, **7b** and **7c**, with the potential to target bromodomain proteins (BRDs) and androgen receptor (AR), respectively.^49^ Gratifyingly, the photophysical properties of model compound **4b** translated well to conjugate **7b**, as evidenced by near-quantitative photoisomerization (*E*-**7b** → *Z-***7b**, 96%; *Z*-**7b** → *E*-**7b**, 90%) and high bistability (see SI).

In summary, we have developed a novel, modular approach toward photoswitchable azotriazoles. Their thorough characterization revealed beneficial photophysical properties such as near-quantitative photoisomerization and long thermal (*Z*)-half-lives. The underexplored class of azoacetylenes can be easily generated by addition of lithiated TIPS-acetylene to diazonium tetrafluoroborate salts. We describe *in situ* desilylative CuAAC reactions between azoacetylenes and a wide range of organoazides, including examples derived from complex natural products. We introduce a diacetylene platform **2q** which allows the execution of two consecutive CuAACs linking two azides via a photoswitchable azotriazole either in a one-pot fashion or in a diversity-oriented two-step procedure. The modular azotriazole photoswitches reported with *N*-alkyl substituents offer high and predictable bistability irrespective of the substitution pattern, making them ideal motifs for the generation of bistable photoswitchable conjugates. Given the broad applicability of CuAAC conjugation strategies, this new approach will find widespread use in the growing field of photoswitches.

## Supporting Information Available

Details on the syntheses and analyses of presented compounds, NMR spectra, crystallographic data, thermal analyses, and photophysical measurements, including Figures S1–S97 and Tables T1–T6 (PDF)

## Accession Codes

CCDC 2088782, 2088785, 2088787, and 2088788 contain the supplementary crystallographic data for this paper. These data can be obtained free of charge via www.ccdc.cam.ac.uk/data_request/cif, or by emailing data_request@ccdc.cam.ac.uk, or by contacting The Cambridge Crystallographic Data Centre, 12 Union Road, Cambridge CB2 1EZ, UK; fax: +44 1223 336033.

## Notes

During the final stages of this work we became aware of work by Prof. T. Li (Shanghai Jiao Tong University).^50^ After discussion we requested publication as companion papers.
