# Supplementary material for: Azoacetylenes for the Synthesis of Arylazotriazole Photoswitches
Source: J Am Chem Soc. 2021 Sep 3;143(36):14495–501. doi: 10.1021/jacs.1c06014 (PMC8447256; doi:10.1021/jacs.1c06014)
Supplement: Supplementary file 1 — ja1c06014_si_001.pdf [file ja1c06014_si_001.pdf]

Azoacetylenes for the Synthesis of Arylazotriazole Photoswitches

Patrick Pfaff, Felix Anderl, Moritz Fink, Moritz Balkenhohl and Erick M. Carreira*

Laboratorium für Organische Chemie

Eidgenössische Technische Hochschule Zürich

8093 Zürich, Switzerland

erickm.carreira@org.chem.ethz.ch

Supporting Information

General Information 2

Optimization of Reaction Conditions 5

General Procedures 7

Preparation of Aryldiazonium Tetrafluoroborate-Salt Starting Materials (1a–1n) 11

Preparation of Arylazoacetylenes 2a–p 25

Preparation of Arylazotriazoles 4a–q 43

Preparation of Azide Derivative Starting Materials SI-5a-1–SI-5g-1 60

Preparation of Azotriazole Derivatives 5a–g 67

Preparation of Azide Derivatives 6a–d 76

Preparation of Diacetylene Platform Reagent 2q 82

Preparation of Azotriazole Conjugates 7a–c 85

Photophysical Characterization and Procedures 91

Overview of Photophysical Properties of Selected Azoacetylenes (Table T4) 96

Overview of Photophysical Properties of Selected Azotriaoles (Table T5) 97

UV-Vis Spectra at Photostationary States after Irradiation at Stated Wavelengths 98

Bistability Measurements – Thermal Relaxation and Eyring Plots 107

Photostability Experiments 140

Photoisomerisation of PROTAC Conjugate 7b 143

Thermal Analysis of Azoacetylenes and Azotriazoles and General Procedures 145

Single Crystal X-Ray Data 154

NMR spectra 163

Determination of Isosbestic Points in HPLC Eluent for Photostationary State Measurement Extracted UV-VIS from HPLC-PDA Detector 243

Photostationary State Determination from HPLC Chromatograms at Isosbestic Wavelength 251

General Information

Unless otherwise noted, all reactions were carried out under nitrogen atmosphere in glassware dried with a heat gun (650 °C) under high vacuum (<1 mbar). Syringes which were used to transfer anhydrous solvents or reagents were purged three times with nitrogen prior to use. All reagents were purchased from commercial suppliers (ABCR, ACROS, Sigma Aldrich, Fluka, TCI, Strem, Alfa, Combi-Blocks or Fluorochem) and used without further purification. Anhydrous solvents over molecular sieves were purchase from Acros and used as received.

Safety Statement

No unexpected or unusually high safety hazards were encountered during this work.

Diazonium tetrafluoroborate salts as prepared in this report, although reported to be stable, are potentially energetic.[1] While no hazardous incidences occurred in our laboratory during this work, care should be taken at any point by using the appropriate personal protective equipment, safety glasses, lab coat and, possibly, blast shields. The diazonium salts should not be prepared above multigram scale.

The safety of a representative set of arylazoacetylenes and arylazotriazoles has been assessed by means of thermal analysis (TGA, DSC and Yoshida Correlation, see SI section "Thermal Analysis"). While this analysis did not indicate explosion hazards for arylazoacetylenes and arylazotriazoles as prepared in this manuscript, caution is advised when handling nitrogen-rich compounds. It is noteworthy that all arylazoacetylenes and arylazotriazoles prepared in this manuscript abided by a C/N ratio ≥ 3 (except for compound **4o** for which C/N = 2.8). Analogues with smaller C/N ratios have not been studied and might require further safety analysis and protective measurements.

When working with LEDs, direct eye contact with the light source should be avoided to prevent eye damage.

Chromatography

Analytical thin layer chromatography (TLC) was performed on Merck silica gel 60 F254 TLC glass plates and visualized with 254 nm light and potassium permanganate or ceric ammonium

[1] Schotten, C.; Leprevost, S. K.; Yong, L. M.; Hughes, C. E.; Harris, K. D. M.; Browne, D. L. Comparison of the Thermal Stabilities of Diazonium Salts and Their Corresponding Triazenes. *Org. Process Res. Dev.* **2020**, 24, 2336–2341.

molybdate staining solutions followed by heating. Organic solutions were concentrated by rotary evaporation at 40 °C. Purification of reaction products was carried out by flash chromatography using Brunschwig silica 32-63, 60Å under 0.3–0.5 bar overpressure. Neutral silica gel was obtained from NACALAI TESQUE, INC.: Silica Gel 60, spherical, neutral.

$KMnO_4$ solution: $KMnO_4$ (3.0 g), 5 drops of conc. H_2SO_4 in water (300 mL).

Ceric ammonium molybdate solution: $Ce(SO_4)_2$ (5 g), $(NH_4)_6Mo_7O_{24}$*$4H_2O$ (25 g), H_2SO_4 conc. (50 mL) in H_2O (450 mL)

Analytical Data

NMR: ^{1}H NMR spectra were recorded on a Bruker AVIII 600 MHz spectrometer with He or prodigy N_2 cryo-probes, Bruker AVIII HD 500 MHz and 400 spectrometers as well as Bruker Neo 500 MHz and 400 MHz spectrometers, and are reported in ppm with the solvent resonance as the reference unless noted otherwise ($CDCl_3$ at 7.26 ppm, C_6D_6 at 7.16 ppm, CD_2Cl_2 at 5.32 ppm, DMSO-d_6 at 2.50 ppm, CD_3CN at 1.94 ppm). Peaks are reported as (s = singlet, d = doublet, t = triplet, q = quartet, m = multiplet or unresolved, br = broad signal, coupling constant(s) in Hz, integration). ^{13}C NMR spectra were recorded with ^{1}H-decoupling on Bruker AVIII 150 MHz spectrometers with He or prodigy N_2 cryo-probes, Bruker AVIII HD 125 MHz and 100 MHz spectrometers as well as Bruker Neo 125 MHz and 100 MHz spectrometers, and are reported in ppm with the solvent resonance as the reference unless noted otherwise ($CDCl_3$ at 77.16 ppm, C_6D_6 at 128.06 ppm, CD_2Cl_2 at 54.00 ppm, DMSO-d_6 at 39.52 ppm, CD_3CN at 1.32 ppm).

Mass spectrometry: High resolution mass spectrometric data were obtained at the mass spectrometry service operated by the Laboratory of Organic Chemistry at the ETHZ on VG-TRIBRID for electron impact ionization (ESI), Varian IonSpec Spectrometer for electrospray ionization (ESI), or IonSpec Ultima Fourier Transform Mass Spectrometer for matrix-assisted laser desorption/ionization (MALDI) and are reported as (*m*/*z*).

Infrared spectra (IR) were recorded neat on a Perkin-Elmer Spectrum Two FT-IR spectrometer. The main peaks are reported as absorption maxima (cm^{-1}).

Optical Rotations were measured on a Jasco P-2000 Polarimeter using a 10 cm cuvette.

UV VIS spectroscopy. UV-Vis spectra were recorded on a Jasco V-630 spectrophotometer in the appropriate solvent at respective concentration. Stock solutions of the respective azo compounds were prepared by dissolving 0.6-6 mg compound, weighed on a fine-balance scale, in anhydrous DMSO at the appropriate concentration. Time-course measurements were performed on Perkin Elmer Lambda 20, 35 or 40 devices each equipped with PTP-6 Peltier elements for temperature control.

LED equipment. Irradiation was performed with a fiber-coupled LED set-up, purchased from ThorLabs Inc., including the following LEDs: 340 nm LED (M340F3), 365 nm LED (M365FP1), 415 nm LED (M415F3), 455 nm LED (M455F3), 505 nm LED (M505F3), 530 nm LED (M530F2). The light beam was directed with a glass fiber (400 UMT custom MUC) and the LEDs were powered by a LEDD1B-Driver and used at 1.2 A amperage and full power.

Optimization of Reaction Conditions

Table T1: Desilylative CuAAC of phenylazoacetylene **2a** to give phenylazotriazole **4a**.

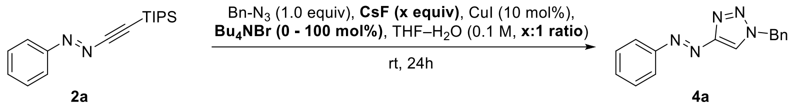

	CsF (equiv)	Bu_4NBr (mol%)	THF–H_2O (ratio, 0.1 M)	Yield **4a**[a]	remaining **2a**[a]
1	1.5 equiv.	-	4:1	19%	69%
2	1.5 equiv.	20 mol%	9:1	69%	-
3	1.5 equiv.	20 mol%	4:1	75%	-
4	1.5 equiv.	20 mol%	3:1	82%	1%
5	1.5 equiv.	20 mol%	2:1	78%	10%
6	1.5 equiv.	20 mol%	1:1	44%	44%
7	1.5 equiv.	5 mol%	3:1	76%	10%
8	1.5 equiv.	10 mol%	3:1	76%	13%%
9	1.5 equiv.	20 mol%	3:1	80%	1%
10	1.5 equiv.	50 mol%	3:1	82%	-
11	1.5 equiv.	100 mol%	3:1	77%	-
**12**	**1.0 equiv**	**20 mol%**	**3:1**	**93%**	**2%**
13	1.1 equiv.	20 mol%	3:1	87%	3%
14	1.25 equiv.	20 mol%	3:1	92%	2%
15	1.5 equiv.	20 mol%	3:1	89%	-
16	2.0 equiv	20 mol%	3:1	89%	-

[a] determined by NMR using dimethyl terephthalate as standard.

Table T2: Desilylative CuAAC of arylazoacetylenes with electron-donating substituents (with **2b** as representative example).

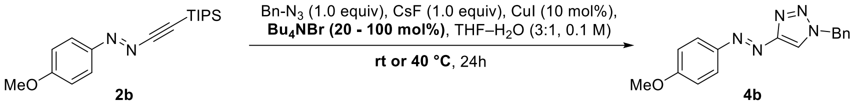

entry	Bu_4NBr (mol%)	Temperature (°C)	yield **4b**[a]	remaining **2b**[a]
1	20 mol%	rt	24%	49%
2	100 mol%	rt	78%	16%
3	20 mol%	40 °C	70%	22%
**4**	**100 mol%**	**40 °C**	**92%**	**-**

[a] determined by NMR using dimethyl terephthalate as standard.

Table T3: Desilylative CuAAC of arylazoacetylenes with electron-withdrawing substituents (with **2i** as representative example).

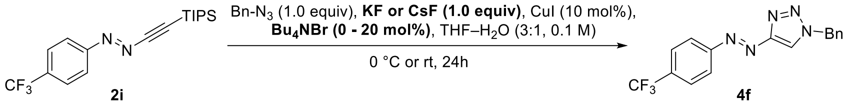

entry	fluoride source	Bu_4NBr (mol%)	Temperature (°C)	yield **4f**[a]	remaining **2i**[a]
1	CsF (1.0 equiv)	-	rt	30%	7%
2	CsF (1.0 equiv)	5 mol%	rt	32%	-
3	CsF (1.0 equiv)	20 mol%	rt	22%	-
**4**	**KF (1.0 equiv)**	**-**	**rt**	**35%**	**4%**
5	KF (1.0 equiv)	5 mol%	rt	31%	
6	KF (1.0 equiv)	20 mol%	rt	15%	
7	KF (1.0 equiv)	-	0 °C	-	-

[a] determined by NMR using dimethyl terephthalate as standard.

General Procedures

General Procedure 1 (GP1): Preparation of aryldiazonium tetrafluoroborates **1c–j, 1l–n**

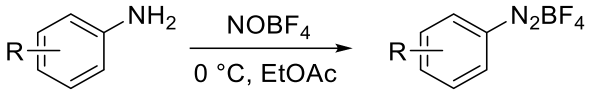

$NOBF_4$ (1.20 equiv) was dissolved in anhydrous EtOAc (1.67 mL/mmol aniline; if the aniline is liquid: 3.35 mL/mmol aniline; in some cases reaction was performed in anhydrous CH_3CN as indicated for each compound). The respective aniline (1.00 equiv) was either added neatly (if liquid) or as solution in EtOAc (1.67 mL/mmol) at 0 °C to the $NOBF_4$ solution (total concentration: 0.3 M). The reaction mixture was stirred at 0 °C for 1 hour. If conversion was incomplete (as monitored by TLC), the reaction mixture was warmed to room temperature and stirred for another hour. Then, ice-cold Et_2O was added at 0 °C and the resulting suspension was filtered by vacuum filtration. The obtained solid was washed with ice-cold Et_2O and air-dried under vacuum for 3 minutes. The prepared diazonium tetrafluoroborate salt was transferred to a pre-dried flask under N_2 and employed within the same day in the subsequent preparation of arylazoacetylenes (**GP3**) to avoid long-term storage of aryl diazonium salts.

General Procedure 2 (GP2): Preparation of a 0.6 M Lithium-TIPS-acetylene in THF–hexane

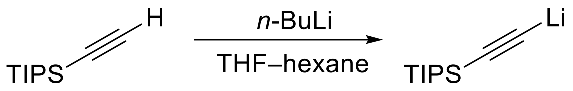

Triisopropylsilylacetylene (12.8 mL, 57.0 mmol, 1.00 equiv) was added to a graduated, flame-dried Schlenk tube and was dissolved in anhydrous THF (22.8 mL) under N_2. *n*-BuLi (1.6 M in hexanes, 35.6 mL, 57.0 mmol, 1.00 equiv) was added to the stirred solution at –78 °C and the mixture was allowed to warm to room temperature. The solution was diluted with THF (25.0 mL) to a total volume of 95.0 mL (0.6 M). The obtained stock solution could be stored for several months at 0 °C without loss of concentration.

General Procedure 3 (GP3): Preparation of arylazoacetylenes **2a–2n**

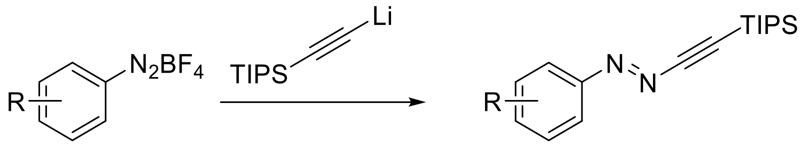

The diazonium tetrafluoroborate salt (1.00 equiv) was suspended in anhydrous THF (3.34 mL/mmol) in a pre-dried flask under N_2. A lithium-TIPS-acetylene stock solution (600 mM in THF–hexane, 1.00 equiv) was quickly added at –78 °C (total concentration: 0.2 M) and after 5 minutes, the cooling bath was removed. The mixture was stirred for 12 hours at room temperature and was quenched by addition of sat. aq. $NaHCO_3$ and diluted with EtOAc. The aq. phase was extracted with EtOAc (2x), the combined organic layers were washed with brine and dried over $MgSO_4$. After concentrating under reduced pressure, the crude product was purified by flash column chromatography on silica gel using the appropriate eluent.

General Procedure 4 (GP4): Preparation of azotriazoles **4d–e**, **4g**, **4j**, **4l**-**q**

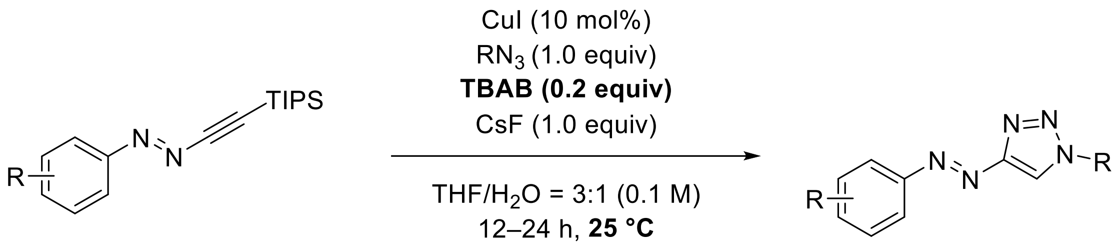

Azoacetylene (1.0 equiv), CuI (10 mol%) and TBAB (20 mol%) were added to a pre-dried flask and set under nitrogen atmosphere. The mixture was dissolved in THF/H_2O (3:1, 0.1 M, also taking the water in account which is added via the aq. CsF solution) and the respective azide (1.0 equiv) was added dropwise. An aq. solution of CsF (1.5 M, 1.0 equiv) was added, and the reaction was stirred at 25 °C for the given time. After filtration through a plug of celite, further eluted with ethyl acetate (50 mL/0.3 mmol azoacetylene), the mixture was concentrated in vacuo. The crude product was purified by flash column chromatography on silica gel using the appropriate eluent.

General Procedure 5 (GP5): Preparation of azotriazoles **4a–c**, **4i**, **5** and **7**.

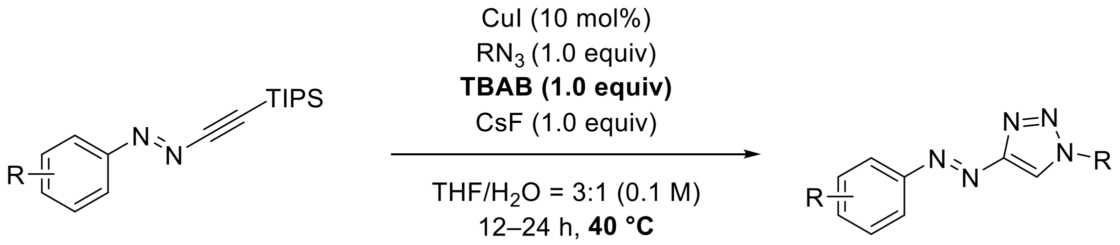

Azoacetylene (1.0 equiv), CuI (10 mol%) and TBAB (1.0 equiv) were added to a pre-dried flask and set under nitrogen atmosphere. The mixture was dissolved in THF/H_2O (3:1, 0.1 M, also taking the water in account which is added via the aq. CsF solution) and the respective azide (1.0 equiv) was added dropwise. An aq. solution of CsF (1.5 M, 1.0 equiv) was added, and the reaction was stirred at 40 °C for the given time. After filtration through a plug of celite, further eluted with ethyl acetate (50 mL/0.3 mmol azoacetylene), the mixture was concentrated in vacuo. The crude product was purified by flash column chromatography on silica gel using the appropriate eluent.

General Procedure 6 (GP6): Preparation of azotriazoles **4f**, **4h**, **4k**.

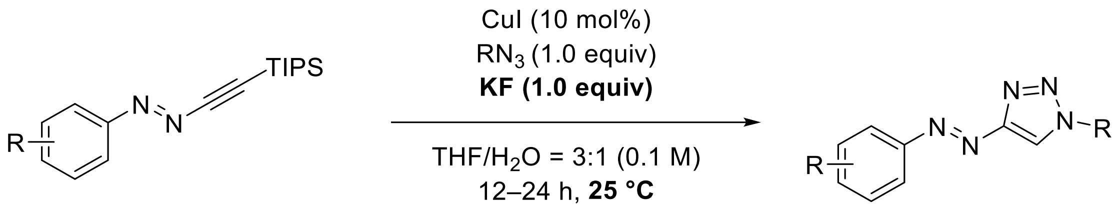

Azoacetylene (1.0 equiv) and CuI (10 mol%) were added to a pre-dried flask and set under nitrogen atmosphere. The mixture was dissolved in THF/H_2O (3:1, 0.1 M, also taking the water in account which is added via the aq. KF solution) and the respective azide (1.0 equiv) was added dropwise. An aq. solution of KF (1.5 M, 1.0 equiv) was added, and the reaction was stirred at 25 °C for the given time. After filtration through a plug of celite, further eluted with ethyl acetate (50 mL/0.3 mmol azoacetylene), the mixture was concentrated in vacuo. The crude product was purified by flash column chromatography on silica gel using the appropriate eluent.

Preparation of Aryldiazonium Tetrafluoroborate-Salt Starting Materials (1a–1n)

Phenyldiazonium tetrafluoroborate (1a)[2]

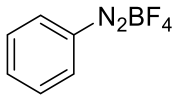

1a

1a was prepared according to literature.[3] Aniline (667 µl, 7.30 mmol, 1.00 equiv) was dissolved in water (3.9 mL) and aq. HBF_4 (48 wt%, 3.4 mL, total 1 M). $NaNO_2$ (504 mg, 7.30 mmol, 1.00 equiv) was dissolved in water (2.2 mL) and added to the reaction mixture at 0 °C. The reaction mixture was stirred for 1 h, followed by addition of ice-cold Et_2O (6.5 mL). The suspension was filtered and washed with Et_2O (2 x 6.5 mL) to obtain **1a** (1.30 g, 6.79 mmol, 93%) as white solid.

^{1}H-NMR (400 MHz, CD_3CN): δ / ppm = 8.51 – 8.47 (m, 2H), 8.28 – 8.23 (m, 1H), 7.96 – 7.90 (m, 2H).

^{13}C-NMR (101 MHz, CD_3CN): δ / ppm = 142.9, 133.3, 132.7, 115.6.

^{19}F-NMR (377 MHz, CD_3CN): δ / ppm = -150.9.

[2] Physical data in accordance with: Schotten, C.; Leprevost, S. K.; Yong, L. M.; Hughes, C. E.; Harris, K. D. M.; Browne, D. L. Comparison of the Thermal Stabilities of Diazonium Salts and Their Corresponding Triazenes. *Org. Process Res. Dev.* **2020**, 24, 2336–2341.

[3] Hanson, P.; Jones, J. R.; Taylor, A. B.; Walton, P. H.; Timms, A. W. Sandmeyer Reactions. Part 7.1An Investigation into the Reduction Steps of Sandmeyer Hydroxylation and Chlorination Reactions. *J. Chem. Soc. Perkin Trans. 2* **2002**, 6, 1135–1150.

4-Methoxydphenyldiazonium tetrafluoroborate (1b)[4]

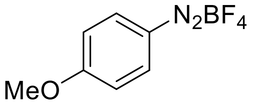

1b

1b was prepared according to literature.[5] 4-methoxyaniline (0.400 g, 3.25 mmol, 1.00 equiv) was dissolved in EtOH (3.25 mL, 1 M) and aq. HBF_4 (48.0 wt%, 0.850 mL, 6.50 mmol, 2.00 equiv). *Tert*-butyl nitrite (90.0 wt%, 0.860 mL, 6.50 mmol, 2.00 equiv) was added dropwise at 0 °C. The reaction mixture was stirred for 1 h, followed by addition of ice-cold Et_2O (3 mL). The suspension was filtered and washed with Et_2O (2 x 3 mL) to obtain **1b** (585 mg, 2.64 mmol, 81%) as white solid.

^{1}H-NMR (400 MHz, CD_3CN): δ / ppm = 8.42 – 8.36 (m, 2H), 7.37 – 7.31 (m, 2H), 4.06 (d, *J* = 1.1 Hz, 3H).

^{13}C-NMR (101 MHz, CD_3CN): δ / ppm = 171.4, 136.7, 118.9, 102.5 (HMBC), 58.6.

^{19}F-NMR (377 MHz, CD_3CN): δ / ppm = -151.7.

[4] Physical data in accordance with: Schotten, C.; Leprevost, S. K.; Yong, L. M.; Hughes, C. E.; Harris, K. D. M.; Browne, D. L. Comparison of the Thermal Stabilities of Diazonium Salts and Their Corresponding Triazenes. *Org. Process Res. Dev.* **2020**, 24, 2336–2341.

[5] Kovács, S.; Bayarmagnai, B.; Aillerie, A.; Gooßen, L. J. Practical Reagents and Methods for Nucleophilic and Electrophilic Phosphorothiolations. Adv. Synth. Catal. 2018, 360 (10), 1913–1918.

4-(Methylthio)phenyldiazonium tetrafluoroborate (1c)[6]

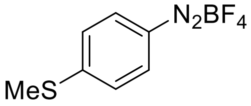

1c

1c was prepared according to **GP1** using 4-(methylthio)aniline (432 mg, 386 μl, 3.10 mmol, 1.00 equiv) and $NOBF_4$ (435 mg, 3.72 mmol, 1.20 equiv). The reaction was conducted in anhydrous EtOAc (10.3 mL, 0.3 M). After addition of ice-cold Et_2O, the dark suspension was filtered and washed with Et_2O (2 x 5 mL) and EtOH (2 x 5 mL). **1c** (0.530 g, 2.22 mmol, 72%) was obtained as dark-green solid.

^{1}H-NMR (400 MHz, CD_3CN): δ / ppm = 8.25 (d, *J* = 9.1 Hz, 2H), 7.65 (d, *J* = 9.1 Hz, 2H), 2.65 (s, 3H).

^{13}C-NMR (101 MHz, CD_3CN): δ / ppm = 162.6, 132.8, 127.8, 106.5, 15.3.

^{19}F-NMR (377 MHz, CD_3CN): δ / ppm = -151.2.

[6] Physical data in accordance with: Bremerich, M.; Conrads, C. M.; Langletz, T.; Bolm, C. Additions to N-Sulfinylamines as an Approach for the Metal-Free Synthesis of Sulfonimidamides: O-Benzotriazolyl Sulfonimidates as Activated Intermediates. *Angew. Chemie - Int. Ed.* **2019**, 58, 19014–19020.

2,6-Dimethoxyphenyldiazonium tetrafluoroborate (1d)[7]

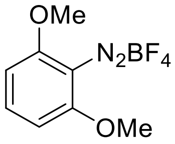

1d

1d was prepared according to **GP1** using 2,6-dimethoxyaniline (256 mg, 1.67 mmol, 1.00 equiv) and $NOBF_4$ (254 mg, 2.17 mmol, 1.30 equiv). The reaction was conducted in anhydrous acetonitrile (5.6 mL, 0.3 M) at 0 °C. The reaction was stirred for 1 hour at 0 °C after which the reaction mixture was added slowly to ice-cold Et_2O (50 mL) while stirring. After filtration, **1d** (236 mg, 0.937 mmol, 56%) was obtained as a brown solid after filtration.

^{1}H-NMR (400 MHz, CD_3CN): δ / ppm = 8.10 (t, *J* = 8.8 Hz, 1H), 6.96 (d, *J* = 8.8 Hz, 2H), 4.14 (s, 6H).

^{13}C-NMR (101 MHz, CD_3CN): δ / ppm = 164.2, 147.4, 106.8, 91.4 (HMBC[8]), 59.9.

^{19}F-NMR (377 MHz, CD_3CN): δ / ppm = -151.9.

[7] Physical data in accordance with: Hansen, M. J.; Lerch, M. M.; Szymanski, W.; Feringa, B. L. Direct and Versatile Synthesis of Red-Shifted Azobenzenes. *Angew. Chemie - Int. Ed.* **2016**, 55, 13514–13518.

[8] Due to quadrupolar coupling to ^{14}N, carbons bound to diazonium can have very low intensity in ^{13}C NMR spectra even at high sample concentration. In some cases, detection of these peaks was only possible via HMBC spectra as indicated above.

2,6-Dichlorophenyldiazonium tetrafluoroborate (1e)[9]

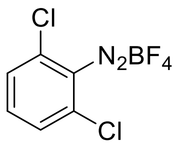

1e

1e was prepared according to **GP1** using 2,6-dichloroaniline (808 mg, 4.99 mmol, 1.00 equiv) $NOBF_4$ (911 mg, 7.80 mmol, 1.56 equiv). The reaction was conducted in anhydrous EtOAc (11.5 mL, 0.3 M) at 0 °C for 1 h. After addition of ice-cold Et_2O, the suspension was filtered and washed with Et_2O (2 x 5 mL) and EtOH (2 x 5 mL). **1e** (1.23 g, 4.72 mmol, 95%) was obtained as an off-white solid,

^{1}H-NMR (500 MHz, CD_3CN): δ / ppm = 8.22 (dd, *J* = 8.7, 8.1 Hz, 1H), 7.98 – 7.96 (m, 2H).

^{13}C-NMR (126 MHz, CD_3CN): δ / ppm = 144.9, 140.2, 132.4, 116.8.

^{19}F-NMR (471 MHz, CD_3CN): δ / ppm = -151.8.

HRMS (ESI) calcd. for $C_6H_3Cl_2N_2$ [M^+ (without BF_4^-)]: 172.9668, found 172.9665.

[9] Physical data in accordance with: Hansen, M. J.; Lerch, M. M.; Szymanski, W.; Feringa, B. L. Direct and Versatile Synthesis of Red-Shifted Azobenzenes. *Angew. Chemie - Int. Ed.* **2016**, 55, 13514–13518.

2,6-Difluorophenyldiazonium tetrafluoroborate (1f)[10]

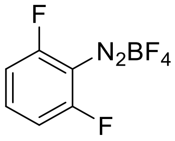

1f

1f was prepared according to **GP1** using 2,6-difluoroaniline (445 mg, 371 µl, 3.45 mmol, 1.00 equiv) $NOBF_4$ (604 mg, 5.17 mmol, 1.50 equiv). The reaction was conducted in anhydrous EtOAc (11.5 mL, 0.3 M) at 0 °C for 1 h. After addition of ice-cold Et_2O, the suspension was filtered and washed with Et_2O (2 x 5 mL) and EtOH (2 x 5 mL). **1f** (0.710 g, 3.12 mmol, 90%) was obtained as an off-white solid,

^{1}H-NMR (500 MHz, C D_3CN): δ / ppm = 8.47 – 8.40 (m, 1H), 7.67 – 7.61 (m, 2H).

Note: In the ^{1}H-NMR, the 2H multiplet signal at 7.67 to 7.61 ppm integrated to 3.0 instead of 2.0 while matching literature-reported data on NMR shifts (Feringa and co-workers[10]). While this might be affected by a strong ^{1}H-^{19}F coupling, the exact nature of this effect is unknown. The diazonium salt competently underwent subsequent reaction to give **2f**.

^{13}C-NMR (126 MHz, CD_3CN): δ / ppm = 162.1 (d, J = 279.6 Hz), 149.4 (t, J = 11.4 Hz), 116.3 (dd, J = 13.8, 5.0 Hz), 96.4 (HMBC[11]).

^{19}F-NMR (471 MHz, CD_3CN): δ / ppm = -99.2, -152.0.

HRMS (ESI) calcd. for $C_6H_3F_2N_2$ [M^+ (without BF_4^-)]: 141.0259, found 141.0261.

[10] Physical data in accordance with: Hansen, M. J.; Lerch, M. M.; Szymanski, W.; Feringa, B. L. Direct and Versatile Synthesis of Red-Shifted Azobenzenes. *Angew. Chemie - Int. Ed.* **2016**, 55, 13514–13518.
[11] Due to quadrupolar coupling to ^{14}N, carbons bound to diazonium can have very low intensity in ^{13}C NMR spectra even at high sample concentration. In some cases, detection of these peaks was only possible via HMBC spectra as indicated above.

2,6-Dimethylphenyldiazonium tetrafluoroborate (1g)[12]

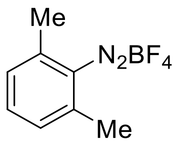

1g

1g was prepared according to **GP1** using 2,6-dimethylaniline (0.390 g, 397 µl, 3.22 mmol, 1.00 equiv) and $NOBF_4$ (451 mg, 3.86 mmol, 1.20 equiv). The reaction was conducted in anhydrous EtOAc (10.7 mL, 0.3 M). After addition of ice-cold Et_2O, the dark suspension was filtered and washed with Et_2O (2 x 5 mL) and EtOH (2 x 5 mL). **1g** (0.290 g, 1.30 mmol, 41%) was obtained as brown solid.

Note: Upon dissolution of a sample in both CD_3CN and $(CD_3)_2CO$ continuous decomposition of the obtained diazonium salt was observed which was accompanied by N_2 evolution. Analysis of the NMR spectra indicated formation of Balz-Schiemann product 2-fluoro-1,3-dimethylbenzene as a mixture with **1g**. Due to this stability issue, only an impure ^{1}H-NMR spectrum could be obtained as a mixture with 2-fluoro-1,3-dimethylbenzene. The undissolved salt, however, was found to be sufficiently stable over the course of a day for subsequent use in the reaction with Lithium-TIPS-acetylene to give product **2g**.

^{1}H-NMR (400 MHz, $(CD_3)_2CO$)**:** δ / ppm = 8.13 (td, *J* = 7.8, 1.3 Hz, 1H), 7.78 – 7.72 (m, 2H), 2.90 (d, *J* = 0.9 Hz, 6H).

[12] A previous report on this diazonium salt appeared in: Çeken, B.; Kízíl, M. Synthesis and DNA-Cleaving Activity of a Series of Substituted Arenediazonium Ions. *Russ. J. Bioorganic Chem.* **2008**, 34, 488–498. This report includes ^{1}H-NMR signals unusually shifted upfield. We assume a similar decomposition like in our case must have taken place unnoticed during measurement.

4-Methylphenyldiazonium tetrafluoroborate (1h)[13]

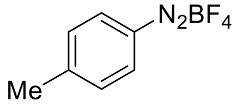

1h

1h was prepared according to **GP1** using *p*-toluidine (368 mg, 3.43 mmol, 1.00 equiv) and $NOBF_4$ (481 mg, 4.12 mmol, 1.20 equiv). The reaction was conducted in anhydrous EtOAc (11.4 mL, 0.3 M) at 0 °C for 1 h. After addition of ice-cold Et_2O, the dark suspension was filtered and washed with Et_2O (2 x 5 mL) and EtOH (2 x 5 mL). **1h** (602 mg, 2.91 mmol, 85%) was obtained as brown solid, containing 10% of an unknown impurity as judged by ^{1}H NMR.

^{1}H-NMR (400 MHz, CD_3CN): δ / ppm = 8.39 – 8.33 (m, 2H), 7.76 – 7.71 (m, 2H), 2.61 (s, 3H).

^{13}C-NMR (101 MHz, CD_3CN): δ / ppm = 157.2, 133.4, 133.3, 111.4, 23.2.

^{19}F-NMR (377 MHz, CD_3CN): δ / ppm = -151.3.

[13] Physical data in accordance with: Xing, B.; Ni, C.; Hu, J. Hypervalent Iodine(III)-Catalyzed Balz–Schiemann Fluorination under Mild Conditions. *Angew. Chemie - Int. Ed.* **2018**, 57, 9896–9900.

4-(Trifluoromethyl)phenyldiazonium tetrafluoroborate (1i)[14]

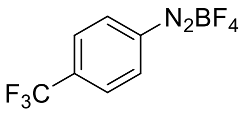

1i

1i was prepared according to **GP1** using 4-(trifluoromethyl)aniline (506 mg, 394 μl, 3.14 mmol, 1.00 equiv) and $NOBF_4$ (0.440 g, 3.77 mmol, 1.20 equiv). The reaction was conducted in anhydrous EtOAc at 0 °C for 1 h. Ice-cold Et_2O was added, and the resulting sticky precipitate was suspended in a mixture of Et_2O and EtOAc. After filtration, **1i** (0.590 g, 2.27 mmol, 72%) was obtained as a white solid.

^{1}H-NMR (400 MHz, CD_3CN): δ / ppm = 8.74 (d, *J* = 8.8 Hz, 2H), 8.22 (d, *J* = 8.8 Hz, 2H).

^{13}C-NMR (101 MHz, CD_3CN): δ / ppm = 141.4 (q, *J* = 34.2 Hz), 134.6, 129.8 (q, *J* = 3.8 Hz), 123.3 (q, *J* = 273.6 Hz), 120.7 (q, *J* = 1.5 Hz).

^{19}F-NMR (377 MHz, CD_3CN): δ / ppm = -64.8, -150.2.

[14] Physical data in accordance with: Ma, X.; Herzon, S. B. Cobalt Bis(Acetylacetonate)–Tert-Butyl Hydroperoxide–Triethyl-Silane: A General Reagent Combination for the Markovnikov-Selective Hydrofunctionalization of Alkenes by Hydrogen Atom Transfer. *Beilstein J. Org. Chem.* **2018**, 14, 2259–2265.

4-Bromophenyldiazonium tetrafluoroborate (1j)[15]

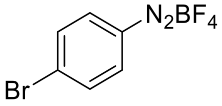

1j

1j was prepared according to **GP1** using 4-bromoaniline (562 mg, 3.27 mmol, 1.00 equiv) and $NOBF_4$ (458 mg, 3.92 mmol, 1.20 equiv). The reaction was conducted in anhydrous EtOAc (10.9 mL, 0.3 M). After addition of ice-cold Et_2O, the dark suspension was filtered and washed with Et_2O (2 x 5 mL) and EtOH (2 x 5 mL). **1j** (865 mg, 3.19 mmol, 98%) was obtained as brown solid.

^{1}H-NMR (400 MHz, CD_3CN): δ / ppm = 8.38 – 8.32 (m, 2H), 8.13 – 8.08 (m, 2H).

^{13}C-NMR (101 MHz, CD_3CN): δ / ppm = 139.8, 136.4, 134.4, 114.3 (HMBC[16]).

^{19}F-NMR (377 MHz, CD_3CN): δ / ppm = -151.5.

---

[15] Physical data in accordance with: Schotten, C.; Leprevost, S. K.; Yong, L. M.; Hughes, C. E.; Harris, K. D. M.; Browne, D. L. Comparison of the Thermal Stabilities of Diazonium Salts and Their Corresponding Triazenes. *Org. Process Res. Dev.* **2020**, 24, 2336–2341.

[16] Due to quadrupolar coupling to ^{14}N, carbons bound to diazonium can have very low intensity in ^{13}C NMR spectra even at high sample concentration. In some cases, detection of these peaks was only possible via HMBC spectra as indicated above.

3-Bromophenyldiazonium tetrafluoroborate (1k)[17]

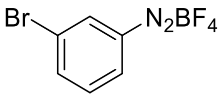

1k

3-Bromoaniline (0.500 g, 2.91 mmol, 1.00 equiv) was suspended in aq. HBF_4 (48.0 wt%, 1.13 mL, 7.28 mmol, 2.50 equiv). H_2O (5.0 mL) was added, and the solution was cooled to 0 °C. $NaNO_2$ (225 mg, 3.26 mmol, 1.12 equiv) was dissolved in H_2O (450 µl) and added to the reaction mixture. The mixture was stirred for 45 minutes at 0 °C. After filtration and washing with water (3 mL), EtOH (3 mL) and Et_2O (3 x 3 mL), **1k** (0.690 g, 2.55 mmol , 88%) was obtained as an off-white solid.

^{1}H-NMR (400 MHz, CD_3CN): δ / ppm = 8.63 (t, *J* = 2.0 Hz, 1H), 8.50 (ddd, *J* = 8.3, 2.0, 1.0 Hz, 1H), 8.39 (ddd, *J* = 8.3, 2.0, 1.0 Hz, 1H), 7.84 (t, *J* = 8.3 Hz, 1H).

^{13}C-NMR (101 MHz, CD_3CN): δ / ppm = 146.2, 134.9, 134.1, 132.6, 124.5, 117.1.

^{19}F-NMR (377 MHz, CD_3CN): δ / ppm = -151.2.

[17] Physical data in accordance with: Patouret, R.; Kamenecka, T. M. Synthesis of 2-Aryl-2H-Tetrazoles via a Regioselective [3+2] Cycloaddition Reaction. *Tetrahedron Lett.* **2016**, 57, 1597–1599.

4-(*tert*-butoxycarbonyl)phenyldiazonium tetrafluoroborate (1l)[18]

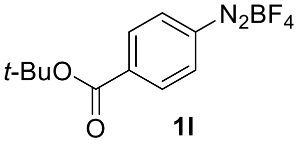

1l

1l was prepared according to **GP1** using *tert*-butyl 4-aminobenzoate (0.550 g, 2.85 mmol, 1.00 equiv) and $NOBF_4$ (415 mg, 3.55 mmol, 1.25 equiv). The reaction was conducted in anhydrous EtOAc. After stirring for 1 hour at 0 °C, the reaction mixture was allowed to warm to room temperature. After another hour, ice-cold ether was added and after filtration, **1l** (785 mg, 2.69 mmol, 94%) was obtained as a white solid.

^{1}H-NMR (400 MHz, CD_3CN): δ / ppm = 8.60 – 8.56 (m, 2H), 8.40 – 8.36 (m, 2H), 1.60 (s, 9H).

^{13}C-NMR (101 MHz, CD_3CN): δ / ppm = 163.1, 143.9, 133.7, 132.8, 119.0, 84.9, 28.1.

^{19}F-NMR (377 MHz, CD_3CN): δ / ppm = -151.1.

[18] Physical data in accordance with: Hansen, M. J.; Lerch, M. M.; Szymanski, W.; Feringa, B. L. Direct and Versatile Synthesis of Red-Shifted Azobenzenes. *Angew. Chemie - Int. Ed.* **2016**, 55, 13514–13518.

3,4,5-trimethoxyphenyldiazonium tetrafluoroborate (1m)[19]

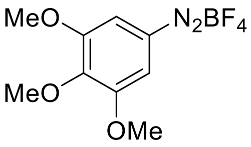

1m

1m was prepared according to **GP1** using 3,4,5-trimethoxyaniline (545 mg, 2.97 mmol, 1.00 equiv) and $NOBF_4$ (434 mg, 3.72 mmol, 1.25 equiv.). The reaction was conducted in anhydrous CH_3CN–CH_2Cl_2 (2.5:1, total volume 10 mL, 0.3 M). $NOBF_4$ was dissolved in CH_3CN (3.0 mL) and the aniline was added as a solution in CH_2Cl_2–CH_3CN (2:1, 3 mL) dropwise at –30 °C, followed by rinsing with CH_3CN (2 x 1.5 mL). The reaction mixture was warmed to –15 °C over the course of 30 minutes and was then allowed to stir at 0 °C for one hour. Following addition of 10 mL ice-cold Et_2O at 0 °C, the resulting sticky precipitate was suspended in EtOH (5 mL) After filtration and further washing with EtOH (3 x 5 mL), **1m** (0.230 g, 0.816 mmol, 27%) was obtained as a brown solid.

^{1}H-NMR (400 MHz, CD_3CN): δ / ppm = 7.77 (s, 2H), 4.13 (s, 3H), 3.92 (s, 6H).

^{13}C-NMR (101 MHz, CD_3CN): δ / ppm = 154.3, 152.5, 111.4, 104.8 (HMBC[20]), 62.9, 58.1.

^{19}F-NMR (377 MHz, CD_3CN): δ / ppm = -151.6.

---

[19] Physical data in accordance with: Rastogi, S. K.; Zhao, Z.; Gildner, M. B.; Shoulders, B. A.; Velasquez, T. L.; Blumenthal, M. O.; Wang, L.; Li, X.; Hudnall, T. W.; Betancourt, T.; Du, L.; Brittain, W. J. Synthesis, Optical Properties and in Vitro Cell Viability of Novel Spiropyrans and Their Photostationary States. *Tetrahedron* **2021**, 80, 131854.

[20] Due to quadrupolar coupling to ^{14}N, carbons bound to diazonium can have very low intensity in ^{13}C NMR spectra even at high sample concentration. In some cases, detection of these peaks was only possible via HMBC spectra as indicated above.

4-Nitrophenyldiazonium tetrafluoroborate (1n)[21]

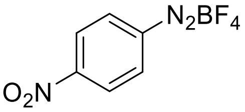

1n

1n was prepared according to **GP1** using 4-nitroaniline (529 mg, 3.83 mmol, 1.00 equiv) and $NOBF_4$ (537 mg, 4.60 mmol, 1.20 equiv). The reaction was conducted in anhydrous EtOAc (12.8 mL, 0.3 M). The reaction mixture was stirred at 0 °C for 1 hour. After addition of ice-cold Et_2O, the dark suspension was filtered and rinsed with Et_2O (2 x 5 mL). **1n** (855 mg, 3.61 mmol, 94%) was obtained as white solid.

^{1}H-NMR (400 MHz, CD_3CN): δ / ppm = 8.78 – 8.74 (m, 2H), 8.65 – 8.60 (m, 2H).

^{13}C-NMR (101 MHz, CD_3CN): δ / ppm = 155.3, 135.5, 127.6, 121.6.

^{19}F-NMR (377 MHz, CD_3CN): δ / ppm = -151.2.

[21] Physical data in accordance with: Schotten, C.; Leprevost, S. K.; Yong, L. M.; Hughes, C. E.; Harris, K. D. M.; Browne, D. L. Comparison of the Thermal Stabilities of Diazonium Salts and Their Corresponding Triazenes. *Org. Process Res. Dev.* **2020**, 24, 2336–2341.

Preparation of Arylazoacetylenes 2a–p

(E)-1-phenyl-2-((trimethylsilyl)ethynyl)diazene (2a-TMS, observation of decomposition)

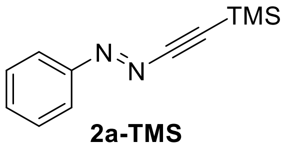

2a-TMS

TMS-acetylene (183 μl, 1.30 mmol, 1.00 equiv) was dissolved in anhydrous THF (2.2 mL) under N_2. *n*-BuLi (1.6 M in hexanes, 815 μl, 1.30 mmol,1.00 equiv) was added at –78 °C and the mixture was stirred for 1 hour during which the temperature raised to –60 °C. Phenyldiazonium tetrafluoroborate (250 mg, 1.30 mmol, 1.00 equiv) was suspended in anhydrous THF (2.2 mL) and cooled to –78 °C. The cold (–78 °C) solution of Li-TMS-acetylene was then added rapidly by cannula to the diazonium salt suspension and rinsed with another 2.2 mL of anhydrous THF. The mixture was allowed to warm to 0 °C over the course of 1 hour and was quenched by addition of sat. aq. $NaHCO_3$. The mixture was diluted with water and EtOAc, the phases were separated, and the aq. phase was extracted with EtOAc. The combined org. layers were washed with brine and dried over Na_2SO_4.

After concentration under reduced pressure, crude NMR indicates clean product formation. After purification by column chromatography (20% CH_2Cl_2 in hexanes), however, continuous decomposition of **2a-TMS** was observed which intensified after storing overnight (see NMR spectra for comparison).

^{1}H-NMR (400 MHz, $CDCl_3$): δ / ppm = 7.85 – 7.80 (m, 2H), 7.57 – 7.47 (m, 3H), 0.33 (s, 9H).

IR (Diamond-ATR, neat): $\tilde{\nu}$ / cm^{-1} = 3065, 2959, 2899, 2096, 1595, 1559, 1497, 1458, 1250, 1209, 1151, 1064, 879, 841, 760, 686.

HRMS (EI) calcd. for $C_{11}H_{14}N_2Si$ $[M]^+$: 202.0921, found 202.0920.

(E)-1-phenyl-2-((triisopropylsilyl)ethynyl)diazene (2a)

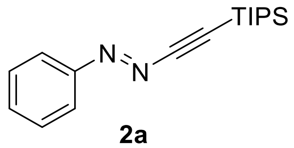

2a

2a was prepared according to **GP3** using diazonium tetrafluoroborate **1a** (1.27 g, 6.62 mmol, 1.00 equiv) and Li-TIPS-acetylene (600 mM in THF–hexane, 11.0 mL, 1.00 equiv) in anhydrous THF (22.1 mL, 0.2 M in total). The crude product was purified by flash column chromatography (SiO_2, hexanes to 4% EtOAc in hexanes) to obtain **2a** (1.89 g, 6.60 mmol, >99%) as dark red oil.

Note: a sample of a mixed fraction of a former, unoptimized batch (containing residual TIPS-acetylene after column chromatography) was stored for about a year at room temperature in the dark (see NMR spectra for comparison). During this period, no decomposition of **2a** was observed.

^{1}H-NMR (400 MHz, $CDCl_3$): δ / ppm = 7.87 – 7.82 (m, 2H), 7.56 – 7.46 (m, 3H), 1.29 – 1.14 (m, 21H).

^{13}C-NMR (101 MHz, $CDCl_3$): δ / ppm = 153.8, 133.1, 129.4, 122.7, 115.8, 110.0, 18.8, 11.5.

IR (Diamond-ATR, neat): $\tilde{\nu}$ / cm^{-1} = 2943, 2891, 2865, 2092, 1585, 1459, 1384, 1205, 1150, 1062, 996, 881, 766, 696, 676, 660, 598, 543.

HRMS (ESI) calcd. for $C_{17}H_{27}N_2Si$ [M+H$^+$]: 287.1938, found 287.1941.

(E)-1-(4-methoxyphenyl)-2-((triisopropylsilyl)ethynyl)diazene (2b)

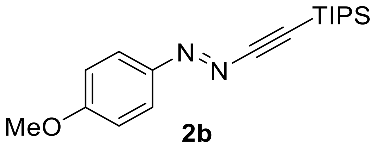

2b

2b was prepared according to **GP3** using diazonium tetrafluoroborate **1b** (559 mg, 2.52 mmol, 1.00 equiv) and Li-TIPS-acetylene (600 mM in THF–hexane, 4.20 mL, 1.00 equiv) in anhydrous THF (8.4 mL, 0.2 M in total). The crude product was purified by flash column chromatography (SiO_2, hexanes to 5% EtOAc in hexanes) to obtain **2b** (795 mg, 2.51 mmol, >99%) as dark red oil.

^{1}H-NMR (400 MHz, $CDCl_3$): δ / ppm = 7.85 – 7.81 (m, 2H), 7.00 – 6.95 (m, 2H), 3.89 (s, 3H), 1.27 – 1.13 (m, 21H).

^{13}C-NMR (101 MHz, $CDCl_3$): δ / ppm =163.8, 148.4, 124.8, 114.5, 111.4, 110.0, 55.7, 18.7, 11.4.

IR (Diamond-ATR, neat): $\tilde{\nu}$ / cm^{-1} = 2943, 2891, 2866, 2092, 1598, 1581, 1498, 1463, 1379, 1256, 1181, 1144, 1071, 1031, 996, 882, 839, 668.

HRMS (ESI) calcd. for $C_{18}H_{29}N_2OSi$ $[M+H]^+$: 317.2044, found 317.2037.

(E)-1-(4-(methylthio)phenyl)-2-((triisopropylsilyl)ethynyl)diazene (2c)

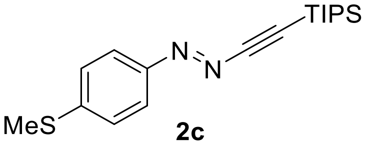

2c

2c was prepared according to **GP3** using diazonium tetrafluoroborate **1c** (0.530 g, 2.23 mmol, 1.00 equiv) and Li-TIPS-acetylene (600 mM in THF–hexane, 3.71 mL, 1.00 equiv) in anhydrous THF (7.39 mL, 0.2 M in total). The crude product was purified by flash column chromatography (SiO_2, 5% CH_2Cl_2 in hexanes to 10% CH_2Cl_2 in hexanes to 20% CH_2Cl_2 in hexanes) to obtain **2c** (0.590 g, 1.77 mmol, 80%) as dark red oil.

^{1}H-NMR (400 MHz, $CDCl_3$): δ / ppm = 7.79 – 7.74 (m, 2H), 7.31 – 7.27 (m, 2H), 2.55 (s, 3H), 1.27 – 1.13 (m, 21H).

^{13}C-NMR (101 MHz, $CDCl_3$): δ / ppm = 151.2, 146.3, 125.7, 123.1, 114.4, 110.3, 18.8, 15.1, 11.5.

IR (Diamond-ATR, neat): $\tilde{\nu}$ / cm^{-1} = 2943, 2891, 2865, 2091, 1582, 1563, 1463, 1375, 1218, 1154, 1092, 996, 880, 828, 716, 677, 660, 621, 541, 459.

HRMS (ESI) calcd. for $C_{18}H_{29}N_2SSi$ $[M+H]^+$: 333.1815, found 333.1812.

(E)-1-(2,6-dimethoxyphenyl)-2-((triisopropylsilyl)ethynyl)diazene (2d)

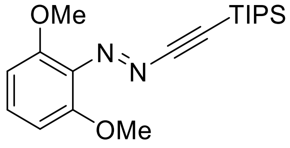

2d

2d was prepared according to **GP3** using diazonium tetrafluoroborate **1d** (236 mg, 937 μmol, 1.00 equiv) and Li-TIPS-acetylene (600 mM in THF–hexane, 1.56 mL, 1.00 equiv) in anhydrous THF (3.12 mL, 0.2 M in total). The crude product was purified by flash column chromatography (SiO_2, 5% EtOAc in hexanes to 15% EtOAc in hexanes to 30% EtOAc in hexanes) to obtain **2d** (0.290 g, 837 μmol, 89%) as red solid.

^{1}H-NMR (400 MHz, $CDCl_3$): δ / ppm = 7.33 (t, *J* = 8.5 Hz, 1H), 6.66 (d, *J* = 8.5 Hz, 2H), 3.89 (s, 6H), 1.25 – 1.11 (m, 21H).

^{13}C-NMR (101 MHz, $CDCl_3$): δ / ppm = 154.0, 133.9, 133.1, 115.5, 110.0, 104.8, 56.6, 18.8, 11.6.

IR (Diamond-ATR, neat): $\tilde{\nu}$ / cm^{-1} = 2944, 2892, 2866, 2121, 2079, 1583, 1482, 1461, 1381, 1258, 1202, 1120, 1254, 994, 917, 883, 779, 728, 669.

HRMS (ESI) calcd. for $C_{19}H_{31}N_2O_2Si$ $[M+H]_+$: 347.2149, found 347.2145.

(E/Z)-1-(2,6-dichlorophenyl)-2-((triisopropylsilyl)ethynyl)diazene (2e)

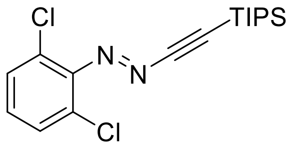

2e

2e was prepared according to **GP3** using diazonium tetrafluoroborate **2e** (0.660 g, 2.53 mmol, 1.00 equiv) and Li-TIPS-acetylene (600 mM in THF–hexane, 4.22 mL, 1.00 equiv) in anhydrous THF (8.44 mL, 0.2 M in total). The crude product was purified by flash column chromatography (SiO_2, 10% CH_2Cl_2 in hexanes to 20% CH_2Cl_2 in hexanes) to obtain **2e** (0.670 g, 1.89 mmol, 75%, obtained as two fractions enriched in either *E* or *Z*-isomer; combined ratio 0.61 : 1 *E*/*Z*; spectrum shown contains 1.30 : 1 *E*/*Z*) as dark red oil.

Minor *E*-isomer:

^{1}H-NMR (500 MHz, $CDCl_3$): δ / ppm = 7.41 (d, *J* = 8.2 Hz, 2H), 7.22 (dd, *J* = 8.2, 7.7 Hz, 1H), 1.29 – 1.15 (m, 21H).

^{13}C-NMR (126 MHz, $CDCl_3$): δ / ppm = 148.3, 129.9, 129.4, 127.4, 123.6, 108.6, 18.7, 11.3.

Major *Z*-isomer:

^{1}H-NMR (500 MHz, $CDCl_3$): δ / ppm = 7.32 (dd, *J* = 8.1, 0.4 Hz, 2H), 7.15 (dd, *J* = 8.5, 7.7 Hz, 1H), 1.04 – 0.89 (m, 21H).

^{13}C-NMR (126 MHz, $CDCl_3$): δ / ppm = 151.6, 128.6, 128.6, 124.0, 120.6, 105.8, 18.3, 11.0.

IR (Diamond-ATR, neat): 2944, 2891, 2866, 1562, 1462, 1435, 1201, 1058, 996, 881, 790, 775, 736, 677, 660, 606.

HRMS (ESI) calcd. for $C_{17}H_{25}Cl_2N_2Si$ $[M+H]^+$: 355.1159, found 355.1161.

(E)-1-(2,6-difluorophenyl)-2-((triisopropylsilyl)ethynyl)diazene (2f)

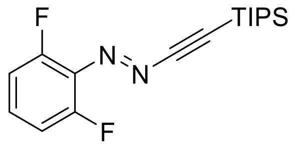

2f

2f was prepared according to **GP3** using diazonium tetrafluoroborate **1f** (655 mg, 2.87 mmol, 1.00 equiv) and Li-TIPS-acetylene (600 mM in THF–hexane, 4.78 mL, 1.00 equiv) in anhydrous THF (9.56 mL, 0.2 M in total). The crude product was purified by flash column chromatography (SiO_2, 20% CH_2Cl_2 in hexanes) to obtain **2f** (725 mg, 2.25 mmol, 78%, obtained as two fractions of which one contained pure *E*-isomer; a second fraction contained 1.45 : 1 *E*/*Z*; combined ratio 6.11 : 1 E/Z) as dark red oil.

Major *E*-isomer

^{1}H-NMR (400 MHz, $CDCl_3$): δ / ppm =7.44 – 7.35 (m, 1H), 7.09 – 7.02 (m, 2H), 1.27 – 1.13 (m, 21H).

^{13}C-NMR (101 MHz, $CDCl_3$): δ / ppm = 155.9 (dd, *J* = 264.2, 3.9 Hz), 132.8 (t, *J* = 10.6 Hz), 132.2 (t, *J* = 9.3 Hz), 123.0, 113.0 (dd, *J* = 20.6, 3.7 Hz), 110.1, 18.8, 11.5.

^{19}F-NMR (376 MHz, $CDCl_3$): δ / ppm = -119.2.

IR (Diamond-ATR, neat): $\tilde{\nu}$ / cm^{-1} = 2945, 2892, 2861, 1612, 1587, 1570, 1477, 1243, 1206, 1081, 1010, 882, 788, 747, 725, 678, 662, 638, 462.

HRMS (ESI) calcd. for $C_{17}H_{24}F_2N_2NaSi$ [M+Na$^+$]: 345.1569, found 345.1570.

(E)-1-(2,6-dimethylphenyl)-2-((triisopropylsilyl)ethynyl)diazene (2g)

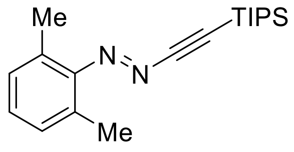

2g

2g was prepared according to **GP3** using diazonium tetrafluoroborate **1g** (275 mg, 1.25 mmol, 1.00 equiv) and Li-TIPS-acetylene (600 mM in THF–hexane, 2.08 mL, 1.00 equiv) in anhydrous THF (4.17 mL, 0.2 M in total). The crude product was purified by flash column chromatography (SiO_2, hexanes to 5% CH_2Cl_2 in hexanes to 10% CH_2Cl_2 in hexanes) to obtain **2g** (0.300 g, 954 μmol, 76%) as dark red oil.

^{1}H-NMR (400 MHz, $CDCl_3$): δ / ppm = 7.20 (dd, *J* = 8.4, 6.6 Hz, 1H), 7.13 – 7.09 (m, 2H), 2.40 (s, 6H), 1.29 – 1.14 (m, 21H).

^{13}C-NMR (101 MHz, $CDCl_3$): δ / ppm = 151.6, 132.8, 130.6, 129.7, 112.6, 109.6, 19.9, 18.8, 11.5.

IR (Diamond-ATR, neat): $\tilde{\nu}$ / cm^{-1} = 2944, 2891, 2866, 2091, 1590, 1463, 1381, 1246, 1194, 1073, 996, 928, 883, 771, 749, 677, 661, 632.

HRMS (ESI) calcd. for $C_{19}H_{31}N_2Si$ $[M+H]^+$: 315.2251, found 315.2258.

((E)-1-(p-tolyl)-2-((triisopropylsilyl)ethynyl)diazene (2h)

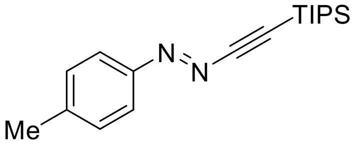

2h

2h was prepared according to **GP3** using diazonium tetrafluoroborate **1h** (0.580 g, 2.82 mmol, 1.00 equiv) and Li-TIPS-acetylene (600 mM in THF–hexane, 4.69 mL, 1.00 equiv) in anhydrous THF (9.41 mL, 0.2 M in total). The crude product was purified by flash column chromatography (SiO_2, hexanes to 5% CH_2Cl_2 in hexanes) to obtain **2h** (0.560 g, 1.86 mmol, 66%) as dark red oil.

^{1}H-NMR (400 MHz, $CDCl_3$): δ / ppm = 7.76 – 7.72 (m, 2H), 7.31 – 7.27 (m, 2H), 2.43 (s, 3H), 1.27 – 1.13 (m, 21H).

^{13}C-NMR (101 MHz, $CDCl_3$): δ / ppm = 152.1, 144.2, 130.1, 122.8, 114.1, 110.0, 21.8, 18.8, 11.5.

IR (Diamond-ATR, neat): $\tilde{\nu}$ / cm^{-1} = 2944, 2891, 2866, 2093, 1600, 1463, 1385, 1209, 1153, 1068, 996, 881, 825, 783, 667, 544.

HRMS (ESI) calcd. for $C_{18}H_{29}N_2Si$ $[M+H]^+$: 301.2095, found 301.2089.

(E)-1-(4-(trifluoromethyl)phenyl)-2-((triisopropylsilyl)ethynyl)diazene (2i)

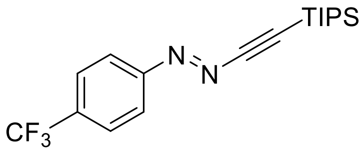

2i

2i was prepared according to **GP3** using diazonium tetrafluoroborate **1i** (0.590 g, 2.27 mmol, 1.00 equiv) and Li-TIPS-acetylene (600 mM in THF–hexane, 3.78 mL, 1.00 equiv) in anhydrous THF (7.52 mL, 0.2 M in total). The crude product was purified by flash column chromatography (SiO_2, hexanes to 2.5% EtOAc in hexanes) to obtain **2i** (0.640 g, 1.81 mmol, 80%) as dark red oil.

^{1}H-NMR (400 MHz, $CDCl_3$): δ / ppm = 7.95 – 7.91 (m, 2H), 7.79 – 7.74 (m, 2H), 1.29 – 1.14 (m, 21H).

^{13}C-NMR (101 MHz, $CDCl_3$): δ / ppm = 155.3, 134.0 (q, *J* = 32.7 Hz), 126.6 (q, *J* = 3.8 Hz), 123.8 (q, *J* = 272.7 Hz), 122.8, 121.0, 110.0, 18.8, 11.5.

^{19}F-NMR (377 MHz, $CDCl_3$): -62.8.

IR (Diamond-ATR, neat): $\tilde{\nu}$ / cm^{-1} = 2945, 2893, 2867, 2095, 1464, 1321, 1170, 1133, 1105, 1064, 1013, 881, 850, 771, 677 610.

HRMS (ESI) calcd. for $C_{18}H_{26}F_3N_2Si$ $[M+H]^+$: 355.1812, found 355.1811.

(E)-1-(4-bromophenyl)-2-((triisopropylsilyl)ethynyl)diazene (2j)

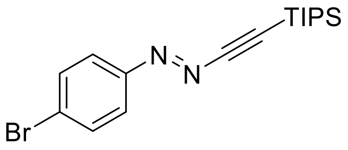

2j

2j was prepared according to **GP3** using diazonium tetrafluoroborate **1j** (865 mg, 3.19 mmol, 1.00 equiv) and Li-TIPS-acetylene (600 mM in THF–hexane, 5.32 mL, 1.00 equiv) in anhydrous THF (10.7 mL, 0.2 M in total). The crude product was purified by flash column chromatography (SiO_2, 5% CH_2Cl_2 in hexanes to 10% CH_2Cl_2 in hexanes to 20% CH_2Cl_2 in hexanes) to obtain **2j** (1.07 g, 2.93 mmol, 92%) as dark red oil.

^{1}H-NMR (400 MHz, $CDCl_3$): δ / ppm = 7.73 – 7.69 (m, 2H), 7.65 – 7.61 (m, 2H), 1.28 – 1.13 (m, 21H).

^{13}C-NMR (101 MHz, $CDCl_3$): δ / ppm = 152.5, 132.7, 128.0, 124.0, 117.8, 110.0, 18.8, 11.5.

IR (Diamond-ATR, neat): $\tilde{\nu}$ / cm^{-1} = 2944, 2891, 2866, 2092, 1581, 1571, 1472, 1384, 1205, 1152, 1067, 1007, 997, 880, 833, 716, 678, 660, 612, 540, 450.

HRMS (ESI) calcd. for $C_{17}H_{26}BrN_2Si$ $[M+H]^+$: 365.1043, found 365.1044.

(E)-1-(3-bromophenyl)-2-((triisopropylsilyl)ethynyl)diazene (2k)

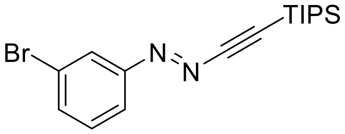

2k

2k was prepared according to **GP3** using diazonium tetrafluoroborate **1k** (632 mg, 2.33 mmol, 1.00 equiv) and Li-TIPS-acetylene (600 mM in THF–hexane, 3.89 mL, 1.00 equiv) in anhydrous THF (7.81 mL, 0.2 M in total). The crude product was purified flash column chromatography (SiO_2, hexanes to 5% CH_2Cl_2 in hexanes) to obtain **2k** (702 mg, 1.92 mmol, 82%) as dark red oil.

^{1}H-NMR (400 MHz, CDCl$_3$): δ / ppm = 7.95 (t, *J* = 1.9 Hz, 1H), 7.82 (ddd, *J* = 8.0, 2.0, 1.0 Hz, 1H), 7.65 (ddd, *J* = 8.0, 2.0, 1.0 Hz, 1H), 7.39 (t, *J* = 8.0 Hz, 1H), 1.29 – 1.13 (m, 21H).

^{13}C-NMR (101 MHz, CDCl$_3$): δ / ppm = 154.5, 135.5, 130.7, 123.9, 123.4, 123.2, 119.2, 109.8, 18.8, 11.5.

IR (Diamond-ATR, neat): $\tilde{\nu}$ / cm^{-1} = 2944, 2866, 2891, 2092, 1572, 1461, 1185, 1154, 1070, 996, 883, 786, 734, 722, 678, 663, 600.

HRMS (ESI) calcd. for $C_{17}H_{26}BrN_2Si$ $[M+H]^+$: 365.1043, found 365.1051.

tert-butyl (E)-4-(((triisopropylsilyl)ethynyl)diazenyl)benzoate (2l)

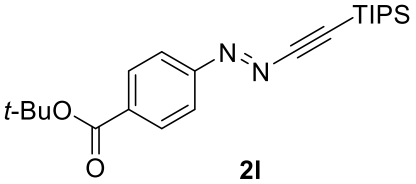

2l

2l was prepared according to **GP3** using diazonium tetrafluoroborate **1l** (785 mg, 2.69 mmol, 1.00 equiv) and Li-TIPS-acetylene (600 mM in THF–hexane, 4.48 mL, 1.00 equiv) in anhydrous THF (8.9 mL, 0.2 M in total). The crude product was purified by flash column chromatography (SiO_2, hexanes to 2.5% EtOAc in hexanes to 5% EtOAc in hexanes to 10% EtOAc in hexanes) to obtain **2l** (0.800 g, 2.07 mmol, 77%) as dark red oil.

^{1}H-NMR (400 MHz, $CDCl_3$): δ / ppm = 8.12 – 8.08 (m, 2H), 7.88 – 7.83 (m, 2H), 1.61 (s, 9H), 1.29 – 1.12 (m, 21H).

^{13}C-NMR (101 MHz, $CDCl_3$): δ / ppm = 165.0, 155.7, 135.5, 130.6, 122.3, 119.9, 110.2, 81.9, 28.3, 18.8, 11.5.

IR (Diamond-ATR, neat): $\tilde{\nu}$ / cm^{-1} = 2944, 2892, 2866, 1717, 1462, 1368, 1289, 1257, 1171, 1115, 1070, 1012, 866, 775, 710, 660.

HRMS (ESI) calcd. for $C_{22}H_{35}N_2O_2Si$ [M+H]$^+$: 387.2462, found 387.2465.

((E)-1-((triisopropylsilyl)ethynyl)-2-(3,4,5-trimethoxyphenyl)diazene (2m)

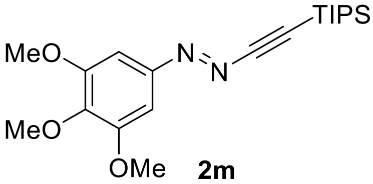

2m

2m was prepared according to **GP3** using diazonium tetrafluoroborate **1m** (0.220 g, 780 μmol, 1.00 equiv) and Li-TIPS-acetylene (600 mM in THF–hexane, 1.30 mL, 1.00 equiv) in anhydrous THF (2.6 mL, 0.2 M in total). The crude product was purified by flash column chromatography (SiO_2, hexanes to 5% EtOAc in hexanes to 10% EtOAc in hexanes) to obtain **2m** (0.280 g, 744 μmol, 95%) as dark red solid.

^{1}H-NMR (400 MHz, $CDCl_3$): δ / ppm = 7.16 (s, 2H), 3.95 (s, 3H), 3.91 (s, 6H), 1.27 – 1.13 (m, 21H).

^{13}C-NMR (101 MHz, $CDCl_3$): δ / ppm = 153.6, 149.7, 142.7, 114.5, 110.0, 100.4, 61.3, 56.4, 18.8, 11.5.

IR (Diamond-ATR, neat): $\tilde{\nu}$ / cm^{-1} = 3519 (broad), 2943, 2891, 2866, 2093, 1594, 1497, 1464, 1423, 1310, 1221, 1130, 1006, 883, 765, 677.

HRMS (ESI) calcd. for $C_{20}H_{32}N_2NaO_3Si$ $[M+Na]^+$: 399.2074, found 399.2074.

(E)-1-(4-nitrophenyl)-2-((triisopropylsilyl)ethynyl)diazene (2n)

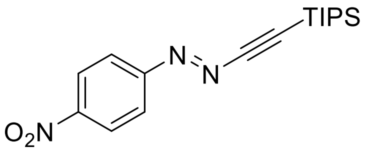

2n

2n was prepared according to **GP3** using diazonium tetrafluoroborate **1n** (855 mg, 3.61 mmol, 1.00 equiv) and Li-TIPS-acetylene (600 mM in THF–hexane, 6.02 mL, 1.00 equiv) in anhydrous THF (12.0 mL, 0.2 M in total). The crude product was purified by flash column chromatography (SiO_2, hexanes to 5% CH_2Cl_2 in hexanes to 10% CH_2Cl_2 in hexanes) to obtain **2n** (1.04 g, 3.14 mmol, 87%) as red/violet solid.

^{1}H-NMR (400 MHz, $CDCl_3$): δ / ppm = 8.39 – 8.33 (m, 2H), 8.00 – 7.94 (m, 2H), 1.30 – 1.14 (m, 21H).

^{13}C-NMR (101 MHz, $CDCl_3$): δ / ppm = 156.4, 149.9, 124.9, 124.9 (HMBC), 123.3, 110.4, 18.8, 11.4.

IR (Diamond-ATR, neat): $\tilde{\nu}$ / cm^{-1} = 3109, 2942, 2891, 2865, 1936, 1608, 1530, 1463, 1343, 1320, 1202, 1064, 995, 883, 859, 842, 753, 717, 678, 656, 616, 600.

HRMS (ESI) calcd. for $C_{17}H_{25}N_3NaO_2Si$ $[M+Na]^+$: 354.1608, found 354.1611.

(8R,9S,13S,14S,17S)-13-methyl-17-((4-((E)-((triisopropylsilyl)ethynyl)diazenyl)phenyl)-ethynyl)-7,8,9,11,12,13,14,15,16,17-decahydro-6H-cyclopenta[a]phenanthrene-3,17-diol (2o)

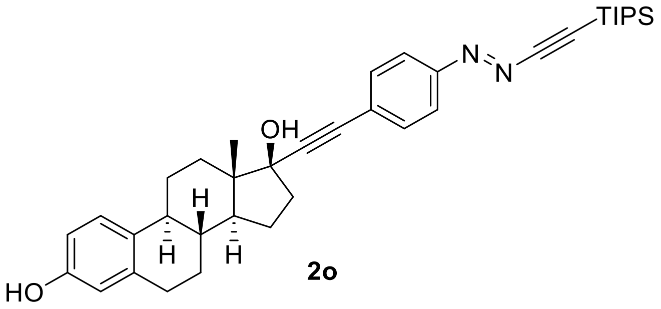

2o

2j (90 mg, 0.25 mmol, 1.0 equiv), ethinylestradiol (73 mg, 0.25 mmol, 1.0 equiv) and CuI (3.3 mg, 17 μmol, 7.0 mol%) were dissolved in anhydrous dioxane (0.50 mL) under N_2. Freshly distilled diisopropylamine (0.18 mL, 1.2 mmol, 5.0 equiv) was added. In a glovebox, Pd((*t*-Bu)$_3$P)$_2$ (8.8 mg, 17 μmol, 7.0 mol%) was weighed into a round bottom flask. The Pd-catalyst was dissolved in dioxane (2 x 0.25 mL, poor solubility) and PhMe (0.25 mL) and added to the reaction mixture (total volume 1.25 mL dioxane–PhMe (5:1), 0.20 M). The reaction mixture was stirred at 45 °C for 13 h upon which more Pd((*t*-Bu)$_3$P)$_2$ (8.8 mg, 17 μmol, 7.0 mol%, as a solution in 2 x 0.12 mL PhMe) and CuI (3.3 mg, 17 μmol, 7.0 mol%) were added. After another 2 h, the reaction mixture was concentrated in vacuo and the crude mixture was purified by flash column chromatography (SiO_2, 20% EtOAc in hexanes to 40% EtOAc in hexanes) to afford **2o** (98 mg, 0.17 mmol, 69%) as a red oil.

^{1}H-NMR (400 MHz, $CDCl_3$): δ / ppm = 7.83 – 7.76 (m, 2H), 7.58 – 7.53 (m, 2tztH), 7.17 (dd, *J* = 8.5, 1.1 Hz, 1H), 6.64 (dd, *J* = 8.5, 2.8 Hz, 1H), 6.57 (d, *J* = 2.8 Hz, 1H), 4.74 (s, 1H), 2.91 – 2.75 (m, 2H), 2.50 – 2.34 (m, 2H), 2.24 (td, *J* = 11.2, 4.2 Hz, 1H), 2.13 (ddd, *J* = 13.8, 11.8, 3.7 Hz, 1H), 2.07 (s, 1H), 2.01 – 1.72 (m, 5H), 1.58 – 1.32 (m, 4H), 1.31 – 1.12 (m, 21H), 0.95 (s, 3H).

^{13}C-NMR (101 MHz, $CDCl_3$): δ / ppm = 153.5, 152.9, 138.4, 132.7, 132.6, 127.8, 126.7, 122.7, 118.1, 115.4, 112.9, 110.2, 96.9, 85.8, 80.7, 50.1, 47.9, 43.8, 39.6, 39.2, 33.3, 29.8, 27.3, 26.6, 23.1, 18.8, 13.0, 11.5.

IR (Diamond-ATR, neat): $\tilde{\nu}$ / cm^{-1} = 3369 (broad), 2942, 2865, 2246, 2217, 2091, 1611, 1594, 1498, 1461, 1381, 1286, 1247, 1210, 1151, 1060, 1011, 912, 881, 846, 816, 733, 678, 667, 612.

HRMS (ESI) calcd. for $C_{37}H_{49}N_2O_2Si$ [M+H]$^+$: 581.3558, found 581.3558.

2-((8S,9S,10R,13S,14S,17R)-17-hydroxy-10,13-dimethyl-3,11-dioxo-2,3,6,7,8,9,10,11,12,13,14,15,16,17-tetradecahydro-1H-cyclopenta[a]phenanthren-17-yl)-2-oxoethyl 4-((E)-((triisopropylsilyl)ethynyl)diazenyl)benzoate (2p)

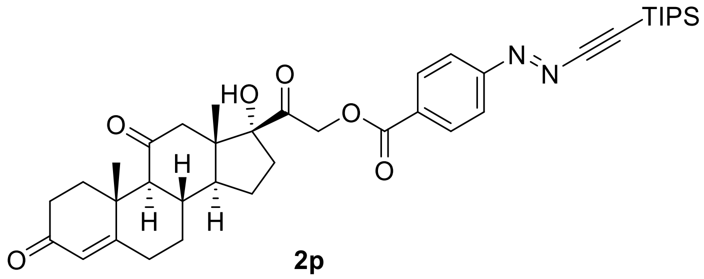

2p

2l (79 mg, 0.20 mmol, 1.0 equiv) was dissolved in anhydrous CH_2Cl_2 (2.0 mL, 0.1 M). 2,6-Lutidine (35 μl, 0.31 mmol, 1.5 equiv, stored over 4A molecular sieves) was added to the reaction mixture. Me_3SiOTf (39 μl, 0.21 mmol, 1.1 equiv; bulb-to-bulb distilled) was added dropwise at 0 °C. The reaction mixture was stirred at rt for 2 hours, followed by 40 °C for 12 hours. Then, the reaction mixture was quenched by addition of MeOH and concentrated under reduced pressure. Deprotected **2l-acid** was obtained as a mixture with lutidinium triflate (105 mg, 63 wt-% purity as judged by NMR, >99%). The crude material was used as is in the subsequent step.

^{1}H-NMR of 2l-acid (400 MHz, $CDCl_3$): δ / ppm = 8.23 (d, *J* = 8.3 Hz, 2H), 7.90 (d, *J* = 8.3 Hz, 2H), 2.85 (s, 3H), 1.29 – 1.14 (m, 21H).

Note: Spectral data extracted from ^{1}H-NMR spectrum of a mixture as stated above.

2l-acid (41 mg, 63 wt-%, 78 μmol, 1.0 equiv), cortisone (28 mg, 78 μmol, 1.0 equiv) and DMAP (9.6 mg, 78 μmol, 1.0 equiv) were dissolved in anhydrous CH_2Cl_2 (0.78 mL, 0.1 M). EDC·HCl (17 mg, 86 μmol, 1.1 equiv) was added to the reaction mixture which was stirred for 12 h at room temperature. The reaction mixture was concentrated in vacuo and the crude material was purified by flash column chromatography (SiO_2, 30% EtOAc in hexanes to 60% EtOAc in hexanes) to give **2p** (0.030 g, 45 μmol, 57% over two steps) as a red oil.

^{1}H-NMR (400 MHz, $CDCl_3$): δ / ppm = 8.21 – 8.17 (m, 2H), 7.91 – 7.87 (m, 2H), 5.74 (d, *J* = 1.7 Hz, 1H), 5.36 (d, *J* = 17.5 Hz, 1H), 4.97 (d, *J* = 17.5 Hz, 1H), 2.94 (d, *J* = 13.1 Hz, 2H), 2.88 – 2.75 (m, 2H), 2.54 – 2.27 (m, 6H), 1.96 (tdd, *J* = 11.0, 8.7, 7.1, 4.0 Hz, 4H), 1.77 – 1.60 (m, 2H), 1.55 – 1.45 (m, 1H), 1.42 (s, 3H), 1.35 – 1.27 (m, 1H), 1.27 – 1.08 (m, 21H), 0.72 (s, 3H).

^{13}C-NMR (101 MHz, $CDCl_3$): δ / ppm = 209.1, 204.3, 200.1, 168.9, 165.4, 156.2, 132.6, 131.1, 124.7, 122.5, 121.3, 110.2, 89.2, 68.5, 62.7, 51.5, 50.0, 50.0, 38.4, 36.6, 35.3, 34.9, 33.9, 32.5, 32.4, 23.4, 18.8, 17.3, 15.7, 11.4.

IR (Diamond-ATR, neat): $\tilde{\nu}$ / cm^{-1} = 3444 (broad),2944, 2866, 2249, 1719, 1708, 1656, 1461, 1361, 1275, 1233, 1221, 1200, 1108, 915, 882, 864, 731, 712, 678.

HRMS (ESI) calcd. for $C_{39}H_{53}N_2O_6Si$ $[M+H]^+$: 673.3667, found 673.3668.

Preparation of Arylazotriazoles 4a–q

(*E*)-1-Benzyl-4-(phenyldiazenyl)-1H-1,2,3-triazole (4a)

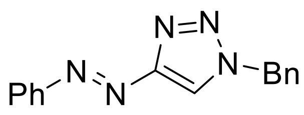

4a

Azotriazole **4a** was prepared via **GP5**, using azoacetylene **2a** (86 mg, 0.30 mmol, 1.0 equiv), CuI (5.7 mg, 0.030 mmol, 10 mol%), TBAB (97 mg, 0.30 mmol, 1.0 equiv), benzyl azide (0.040 g, 0.30 mmol, 1.0 equiv), and CsF (aq. 1.5 M, 0.20 mL, 0.30 mmol, 1.0 equiv) and the reaction was stirred for 12 h at 40 °C. After work-up, the crude product was purified via column chromatography (hexane:ethyl acetate = 8:2, R_f = 0.3) to give **4a** (69 mg, 0.26 mmol, 87%) as a yellow solid.

Alternatively,

azotriazole **4a** was prepared via **GP4**, using azoacetylene **2a** (86 mg, 0.30 mmol, 1.0 equiv), CuI (5.7 mg, 0.030 mmol, 10 mol%), TBAB (19 mg, 0.060 mmol, 20 mol%), benzyl azide (40 mg, 0.30 mmol, 1.0 equiv), and CsF (aq. 1.5 M, 0.20 mL, 0.30 mmol, 1.0 equiv) and the reaction was stirred for 24 h. After work-up, the crude product was purified via column chromatography (hexane:ethyl acetate = 8:2, R_f = 0.3) to give **4a** (61 mg, 0.23 mmol, 77%) as a yellow solid.

^{1}H-NMR (400 MHz, $CDCl_3$): δ / ppm = 7.94 – 7.88 (m, 2H), 7.86 (s, 1H), 7.52 – 7.46 (m, 3H), 7.44 – 7.38 (m, 3H), 7.37 – 7.32 (m, 2H), 5.60 (s, 2H).

^{13}C-NMR (101 MHz, $CDCl_3$): δ / ppm = 160.9, 152.6, 134.0, 131.8, 129.4, 129.3, 129.2, 128.5, 123.1, 115.0, 55.0.

IR (Diamond-ATR, neat): $\tilde{\nu}$ / cm^{-1} = 3140, 3065, 1586, 1470, 1456, 1237, 1153, 1038, 815, 769,716, 706, 687.

HRMS (ESI) calcd. for $C_{15}H_{13}N_5Na$ [M+Na]$^+$: 286.1063, found 286.1065.

(*E*)-1-Benzyl-4-((4-methoxyphenyl)diazenyl)-1H-1,2,3-triazole (4b)

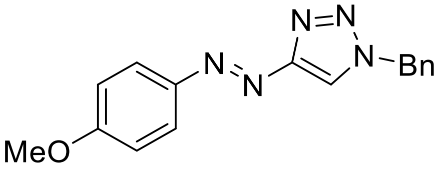

4b

Azotriazole **4b** was prepared via **GP5**, using azoacetylene **2b** (86 mg, 0.30 mmol, 1.0 equiv), CuI (5.7 mg, 0.030 mmol, 10 mol%), TBAB (97 mg, 0.30 mmol, 1.0 equiv), benzyl azide (40 mg, 0.30 mmol, 1.0 equiv), and CsF (aq. 1.5 M, 0.20 mL, 0.30 mmol, 1.0 equiv) and the reaction was stirred for 12 h at 40 °C. After work-up, the crude product was purified via column chromatography (hexane:ethyl acetate = 8:2, R_f = 0.3) to give **4b** (78 mg, 0.27 mmol, 89%) as a yellow solid.

^{1}H-NMR (400 MHz, $CDCl_3$): δ / ppm = 7.93 – 7.87 (m, 2H), 7.80 (s, 1H), 7.43 – 7.31 (m, 5H), 7.01 – 6.95 (m, 2H), 5.58 (s, 2H), 3.87 (s, 3H).

^{13}C-NMR (101 MHz, $CDCl_3$): δ / ppm = 162.7, 161.1, 147.0, 134.2, 129.4, 129.2, 128.4, 125.1, 114.5, 114.4, 55.7, 55.0.

IR (Diamond-ATR, neat): $\tilde{\nu}$ / cm^{-1} = 3139, 2945, 2839, 1600, 1583, 1501, 1456, 1252, 1145, 1029, 840, 817, 713, 703.

HRMS (ESI) calcd. for $C_{16}H_{16}N_5O$ [M+H]$^+$: 294.1349, found 294.1350.

(*E*)-1-Benzyl-4-((4-(methylthio)phenyl)diazenyl)-1H-1,2,3-triazole (4c)

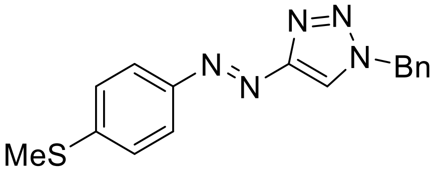

4c

Azotriazole **4c** was prepared via **GP5**, using azoacetylene **2c** (0.10 g, 0.30 mmol, 1.0 equiv), CuI (5.7 mg, 0.030 mmol, 1.0 equiv), TBAB (97 mg, 0.30 mmol, 1.0 equiv), benzyl azide (40 mg, 0.30 mmol, 1.0 equiv), and CsF (aq. 1.5 M, 0.20 mL, 0.30 mmol, 1.0 equiv) and the reaction was stirred for 12 h at 40 °C. After work-up, the crude product was purified via column chromatography (hexane:ethyl acetate = 8:2 to 1:1, R_f = 0.25) to give **4c** (59 mg, 0.19 mmol, 64%, E/Z = 12:1) as an orange solid.

^{1}H-NMR (400 MHz, $CDCl_3$): δ / ppm = 7.88 – 7.80 (m, 3H), 7.43 – 7.27 (m, 7H), 5.59 (s, 2H), 2.53 (s, 3H).

Note: Peaks of the main diastereomer are given.

^{13}C-NMR (101 MHz, $CDCl_3$): δ / ppm = 161.0, 149.9, 144.2, 134.1, 129.4, 129.2, 128.4, 125.9, 123.6, 114.8, 55.0, 15.2.

Note: Peaks of the main diastereomer are given.

IR (Diamond-ATR, neat): $\tilde{\nu}$ / cm^{-1} = 3108, 3034, 2919, 1567, 1587, 1496, 1303, 1234, 1043, 824, 711.

HRMS (ESI) calcd. for $C_{16}H_{15}N_5NaS$ $[M+H]^+$**:** 332.0940, found 332.0936.

(*E*)-1-Benzyl-4-(p-tolyldiazenyl)-1H-1,2,3-triazole (4d)

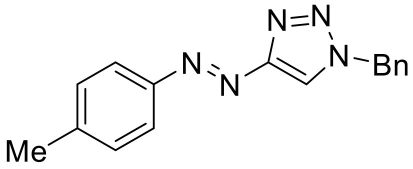

4d

Azotriazole **4d** was prepared via **GP4**, using azoacetylene **2h** (0.090 g, 0.30 mmol, 1.0 equiv), CuI (5.7 mg, 0.030 mmol, 1.0 equiv), TBAB (19 mg, 0.060 mmol, 20 mol%), benzyl azide (40 mg, 0.30 mmol, 1.0 equiv), and CsF (aq. 1.5 M, 0.20 mL, 0.30 mmol, 1.0 equiv) and the reaction was stirred for 24 h. After work-up, the crude product was purified via column chromatography (hexane:ethyl acetate = 8:2, R_f = 0.25) to give **4d** (50 mg, 0.18 mmol, 60%) as a yellow solid.

^{1}H-NMR (400 MHz, $CDCl_3$): δ / ppm = 7.85 – 7.77 (m, 3H), 7.44 – 7.31 (m, 5H), 7.30 – 7.26 (m, 2H), 5.58 (s, 2H), 2.41 (s, 3H).

^{13}C-NMR (101 MHz, $CDCl_3$): δ / ppm = 161.0, 150.8, 142.6, 134.1, 129.9, 129.4, 129.2, 128.4, 123.1, 114.7, 55.0, 21.7.

IR (Diamond-ATR, neat): $\tilde{\nu}$ / cm^{-1} = 3113, 3033, 1602, 1498, 1456, 1434, 1234, 1204, 1153. 1162, 1042, 824, 741, 707, 700.

HRMS (ESI) calcd. for $C_{16}H_{16}N_5$ $[M+H]^+$: 278.1400, found 278.1396.

(*E*)-1-Benzyl-4-((3,4,5-trimethoxyphenyl)diazenyl)-1H-1,2,3-triazole (4e)

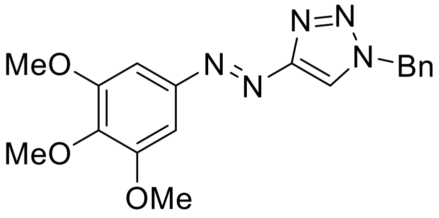

4e

Azotriazole **4e** was prepared via **GP4**, using azoacetylene **2m** (0.11 g, 0.30 mmol, 1.0 equiv), CuI (5.7 mg, 0.030 mmol, 10 mol%), TBAB (19 mg, 0.060 mmol, 20 mol%), benzyl azide (40 mg, 0.30 mmol, 1.0 equiv), and CsF (aq. 1.5 M, 0.20 mL, 0.30 mmol, 1.0 equiv) and the reaction was stirred for 24 h. After work-up, the crude product was purified via column chromatography (hexane:ethyl acetate = 6:4, R_f = 0.3) to give **4e** (64 mg, 0.18 mmol, 60%) as a yellow solid.

^{1}H-NMR (400 MHz, $CDCl_3$): δ / ppm = 7.83 (s, 1H), 7.44 – 7.29 (m, 5H), 7.23 (s, 2H), 5.58 (s, 2H), 3.91 (s, 3H), 3.90 (s, 6H).

^{13}C-NMR (101 MHz, $CDCl_3$): δ / ppm = 160.7, 153.6, 148.4, 141.3, 134.0, 129.4, 129.2, 128.4, 114.8, 100.7, 61.1, 56.3, 55.0.

IR (Diamond-ATR, neat): $\tilde{\nu}$ / cm^{-1} = 3102, 3067, 2991, 2962, 2246, 1593, 1493, 1465, 1123, 920, 720.

HRMS (ESI) calcd. for $C_{18}H_{20}N_5O_3$ $[M+H]^+$: 354.1561, found 354.1558

(*E*)-1-Benzyl-4-((4-(trifluoromethyl)phenyl)diazenyl)-1*H*-1,2,3-triazole (4f)

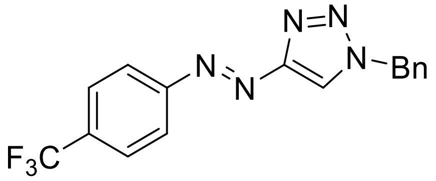

4f

Azotriazole **4f** was prepared via **GP6**, using azoacetylene **2i** (0.11 g, 0.30 mmol, 1.0 equiv), CuI (5.7 mg, 0.030 mmol, 10 mol%), benzyl azide (0.040 g, 0.30 mmol, 1.0 equiv), and KF (aq. 1.5 M, 0.20 mL, 0.30 mmol, 1.0 equiv) and the reaction was stirred for 24 h. After work-up, the crude product was purified via column chromatography (hexane:ethyl acetate = 8:2, R_f = 0.3) to give **4f** (40 mg, 0.12 mmol, 40%) as a yellow-brown solid.

^{1}H-NMR (400 MHz, $CDCl_3$): δ / ppm = 8.03 – 7.96 (m, 2H), 7.91 (s, 1H), 7.76 (d, *J* = 8.3 Hz, 2H), 7.45 – 7.33 (m, 5H), 5.62 (s, 2H).

^{13}C-NMR (101 MHz, $CDCl_3$): δ / ppm = 160.6, 154.4, 133.9, 133.0 (q, *J* = 32.6 Hz), 129.5, 129.4, 128.5, 126.5 (q, *J* = 3.8 Hz), 123.9 (q, *J* = 272 Hz), 123.3, 115.8, 55.1.

^{19}F-NMR (376 MHz, $CDCl_3$): δ / ppm = -62.7.

IR (Diamond-ATR, neat): $\tilde{\nu}$ / cm^{-1} = 3098, 3037, 2957, 1715, 1611, 1526, 1361, 1325.

HRMS (ESI) calcd. for $C_{16}H_{13}F_3N_5$ $[M+H]^+$: 332.1118, found 332.1117

(*E*)-1-Benzyl-4-((4-bromophenyl)diazenyl)-1*H*-1,2,3-triazole (4g)

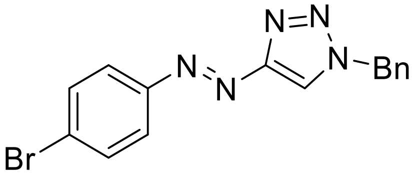

4g

Azotriazole **4g** was prepared via **GP4**, using azoacetylene **2j** (0.11 g, 0.30 mmol, 1.0 equiv), CuI (5.7 mg, 0.030 mmol, 10 mol%), TBAB (19 mg, 0.060 mmol, 20 mol%), benzyl azide (40 mg, 0.30 mmol, 1.0 equiv), and CsF (aq. 1.5 M, 0.20 mL, 0.30 mmol) and the reaction was stirred for 24 h. After work-up, the crude product was purified via column chromatography (hexane:ethyl acetate = 8:2, R_f = 0.25) to give **4g** (40 mg, 0.12 mmol, 39%) as a dark yellow solid.

^{1}H-NMR (400 MHz, CDCl$_3$): δ / ppm = 7.86 (s, 1H), 7.82 – 7.74 (m, 2H), 7.67 – 7.59 (m, 2H), 7.45 – 7.32 (m, 5H), 5.60 (s, 2H).

^{13}C-NMR (101 MHz, CDCl$_3$): δ / ppm = 160.7, 151.3, 133.9, 132.6, 129.5, 129.3, 128.5, 126.4, 124.6, 115.3, 55.1.

IR (Diamond-ATR, neat): $\tilde{\nu}$ / cm^{-1} = 3097, 3061, 3034, 2864, 1584, 1574, 1481, 1363, 1328, 1121, 1020, 830, 710.

HRMS (ESI) calcd. for $C_{15}H_{12}BrN_5Na$ [M+Na]$^+$. 364.0168, found 364.0166.

(*E*)-1-Benzyl-4-((3-bromophenyl)diazenyl)-1*H*-1,2,3-triazole (4h)

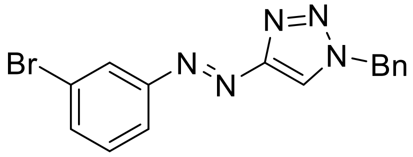

4h

Azotriazole **4h** was prepared via **GP6**, using azoacetylene **2k** (0.11 g, 0.30 mmol, 1.0 equiv), CuI (5.7 mg, 0.030 mmol, 10 mol%), benzyl azide (40 mg, 0.30 mmol, 1.0 equiv), and KF (aq. 1.5 M, 0.20 mL, 0.30 mmol, 1.0 equiv) and the reaction was stirred for 24 h. After work-up, the crude product was purified via column chromatography (hexane:ethyl acetate = 8:2, R_f = 0.3) to give **4h** (43 mg, 0.13 mmol, 42%, *E*:*Z* > 20:1) as a yellow solid.

^{1}H-NMR (400 MHz, $CDCl_3$): δ / ppm = 8.05 (t, *J* = 1.9 Hz, 1H), 7.89 – 7.81 (m, 2H), 7.59 (ddd, *J* = 7.9, 2.0, 1.0 Hz, 1H), 7.46 – 7.30 (m, 6H), 5.60 (s, 2H).

^{13}C-NMR (101 MHz, $CDCl_3$): δ / ppm = 160.5, 153.5, 134.4, 133.9, 130.6, 129.5, 129.3, 128.5, 125.0, 123.3, 123.0, 115.5, 55.1.

IR (Diamond-ATR, neat): $\tilde{\nu}$ / cm^{-1} = 3098, 3065, 3033, 2957, 1721, 1571, 1636, 1455, 1328, 1273, 995, 844, 711, 680.

HRMS (ESI) calcd. for $C_{15}H_{13}BrN_5$ $[M+H]^{+}$.: 342.0349, found 342.0347

(*E*)-1-Benzyl-4-((2,6-dimethoxyphenyl)diazenyl)-1*H*-1,2,3-triazole (4i)

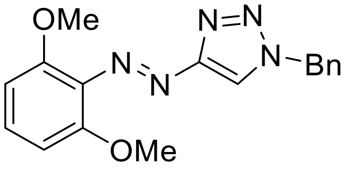

4i

Azotriazole **4i** was prepared via **GP5**, using azoacetylene **2d** (51 mg, 0.15 mmol, 1.0 equiv), CuI (3.0 mg, 15 µmol, 10 mol%), TBAB (49 mg, 0.15 mmol, 1.0 equiv), benzyl azide (0.020 g, 0.15 mmol, 1.0 equiv), and CsF (aq. 1.5 M, 0.10 mL, 0.15 mmol, 1.0 equiv) and the reaction was stirred for 24 h at 40 °C. After work-up, the crude product was purified via column chromatography (hexane:ethyl acetate = 6:4, R_f = 0.25) to give **4i** (40 mg, 0.12 mmol, 84%, *E*:*Z* = 7:1) as a yellow solid.

^{1}H-NMR (400 MHz, CDCl$_3$): δ / ppm = 7.84 (s, 1H), 7.43 – 7.31 (m, 6H), 7.30 – 7.22 (m, 1H), 6.65 (d, *J* = 8.5 Hz, 2H), 5.56 (s, 2H), 3.83 (s, 6H).

Note: Peaks of the main diastereomer are given.

^{13}C-NMR (101 MHz, CDCl$_3$): δ / ppm = 161.6, 153.2, 134.1, 133.0, 130.8, 129.3, 129.1, 128.6, 114.3, 104.9, 56.5, 55.0.

Note: Peaks of the main diastereomer are given.

IR (Diamond-ATR, neat): $\tilde{\nu}$ / cm^{-1} = 3135, 3065, 3007, 2939, 1586, 1475, 1255, 1108, 1037, 720, 700.

HRMS (ESI) calcd. for $C_{17}H_{18}N_5O_2$ [M+H]$^+$**:** 324.1455, found 324.1454.

(*E*)-1-Benzyl-4-((2,6-dimethylphenyl)diazenyl)-1*H*-1,2,3-triazole (4j)

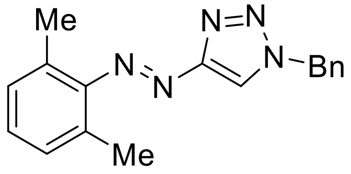

4j

Azotriazole **4j** was prepared via **GP4**, using azoacetylene **2g** (92 mg, 0.29 mmol, 1.0 equiv), CuI (5.7 mg, 0.030 mmol, 10 mol%), TBAB (19 mg, 0.060 mmol, 20 mol%), benzyl azide (0.040 g, 0.30 mmol, 1.0 equiv), and CsF (aq. 1.5 M, 0.20 mL, 0.30 mmol, 1.0 equiv) and the reaction was stirred for 24 h. After work-up, the crude product was purified via column chromatography (hexane:ethyl acetate = 8:2, R_f = 0.3) to give **4j** (58 mg, 0.20 mmol, 68% *E*:*Z* > 20:1) as an orange solid.

^{1}H-NMR (400 MHz, CDCl$_3$): δ / ppm = 7.79 (s, 1H), 7.47 – 7.32 (m, 5H), 7.19 – 7.07 (m, 3H), 5.60 (s, 2H), 2.38 (s, 6H).

^{13}C-NMR (101 MHz, CDCl$_3$): δ / ppm = 161.4, 150.7, 134.2, 131.9, 129.41, 129.39, 129.2, 129.2, 128.4, 113.8, 55.0, 19.4.

IR (Diamond-ATR, neat): $\tilde{\nu}$ / cm^{-1} = 3095, 3066, 3031, 2965, 1443, 1457, 1287, 1050, 773, 965.

HRMS (ESI) calcd. for $C_{17}H_{18}N_5$ $[M+H]^+$: 292.1557, found 292.1558.

(*E*)-1-Benzyl-4-((2,6-difluorophenyl)diazenyl)-1*H*-1,2,3-triazole (4k)

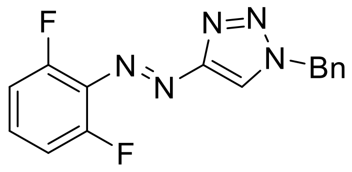

4k

Azotriazole **4k** was prepared via **GP6**, using azoacetylene **2f** (97 mg, 0.30 mmol, 1.0 equiv), CuI (5.7 mg, 0.030 mmol, 10 mol%), benzyl azide (40 mg, 0.30 mmol, 1.0 equiv), and KF (aq. 1.5 M, 0.20 mL, 0.30 mmol, 1.0 equiv) and the reaction was stirred for 24 h. After work-up, the crude product was purified via column chromatography (hexane:ethyl acetate = 8:2, R_f = 0.25) to give **4k** (0.030 g, 0.10 mmol, 33%, *E*:*Z* > 20:1) as a yellow solid.

^{1}H-NMR (400 MHz, $CDCl_3$): δ / ppm = 7.90 (s, 1H), 7.43 – 7.29 (m, 6H), 7.07 – 6.97 (m, 2H), 5.60 (s, 2H).

^{13}C-NMR (101 MHz, $CDCl_3$): δ / ppm = 161.4, 156.0 (dd, *J* = 261.3, 4.1 Hz), 133.9, 131.4 (t, *J* = 10.5 Hz), 131.1 (t, *J* = 9.7 Hz), 129.4, 129.3, 128.5, 115.2, 113.0 – 112.3 (m), 55.0.

^{19}F-NMR (377 MHz, $CDCl_3$): δ / ppm -120.1.

IR (Diamond-ATR, neat): $\tilde{\nu}$ / cm^{-1} = 3137, 3066, 3035, 2950, 1615, 1570, 1478, 1241, 1039, 1013, 787, 720.

HRMS (ESI) calcd. for $C_{15}H_{12}F_2N_5$ $[M+H]^+$**:** 300.1055, found 300.1053.

tert-Butyl (_E_)-4-((1-benzyl-1_H_-1,2,3-triazol-4-yl)diazenyl)benzoate (4l)

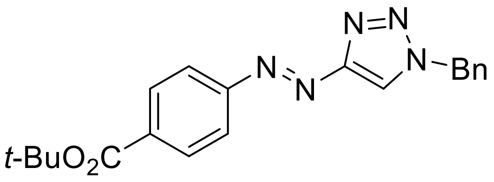

4l

Azotriazole **4l** was prepared via **GP4**, using azoacetylene **2l** (0.14 g, 0.35 mmol, 1.0 equiv), CuI (6.7 mg, 35 µmol, 10 mol%), TBAB (22 mg, 0.070 mmol, 20 mol%), benzyl azide (47 mg, 0.35 mmol, 1.0 equiv), and CsF (aq. 1.5 M, 0.23 mL, 0.35 mmol, 1.0 equiv) and the reaction was stirred for 24 h. After work-up, the crude product was purified via column chromatography (hexane:ethyl acetate = 8:2, R_f = 0.25) to give **4l** (39 mg, 0.11 mmol, 30%, *E*:*Z* > 20:1) as a brown solid.

^{1}H-NMR (400 MHz, $CDCl_3$): δ / ppm = 8.15 – 8.07 (m, 2H), 7.96 – 7.86 (m, 3H), 7.46 – 7.31 (m, 5H), 5.61 (s, 2H), 1.61 (s, 9H).

^{13}C-NMR (101 MHz, $CDCl_3$): δ / ppm = 165.3, 160.8, 155.7, 134.5, 133.9, 130.6, 129.5, 129.3, 128.5, 122.8, 115.5, 81.7, 55.1, 28.3.

IR (Diamond-ATR, neat): $\tilde{\nu}$ / cm^{-1} = 3066, 2977, 2933, 1708, 1604, 1289, 1115, 1011, 848, 710.

HRMS (ESI) calcd. for $C_{20}H_{22}N_5O_2$ $[M+H]^+$: 364.1768, found 364.1767.

(*E*)-1-(Cyclohexylmethyl)-4-(phenyldiazenyl)-1*H*-1,2,3-triazole (4m)

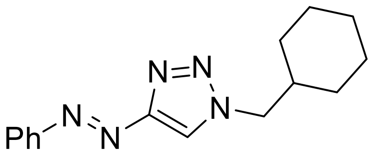

4m

Azotriazole **4m** was prepared via **GP4**, using azoacetylene **2a** (86 mg, 0.30 mmol, 1.0 equiv), CuI (5.7 mg, 0.030 mmol, 10 mol%), TBAB (19 mg, 0.060 mmol, 20 mol%), cyclohexylmethyl azide (42 mg, 0.30 mmol, 1.0 equiv), and CsF (aq. 1.5 M, 0.20 mL, 0.30 mmol, 1.0 equiv) and the reaction was stirred for 24 h. After work-up, the crude product was purified via column chromatography (hexane:ethyl acetate = 8:2, R_f = 0.3) to give **4m** (55 mg, 0.20 mmol, 68%, *E*:*Z* > 20:1) as an orange solid.

^{1}H-NMR (400 MHz, CDCl$_3$): δ / ppm = 7.96 – 7.89 (m, 3H), 7.51 – 7.45 (m, 3H), 4.23 (d, *J* = 7.1 Hz, 2H), 1.93 (ttt, *J* = 10.7, 7.0, 3.4 Hz, 1H), 1.77 – 1.60 (m, 5H), 1.28 – 0.96 (m, 5H).

^{13}C-NMR (101 MHz, CDCl$_3$): δ / ppm = 160.5, 152.6, 131.7, 129.2, 123.1, 115.5, 57.2, 38.8, 30.5, 26.1, 25.5.

IR (Diamond-ATR, neat): $\tilde{\nu}$ / cm^{-1} = 3123, 3107, 2921, 2851, 1466, 1448, 1345, 1097, 1000, 769, 687.

HRMS (ESI) calcd. for $C_{15}H_{20}N_5$ [M+H]$^+$: 270.1713, found 270.1711.

(*E*)-1-(3,5-Dimethylphenyl)-4-(phenyldiazenyl)-1*H*-1,2,3-triazole (4n)

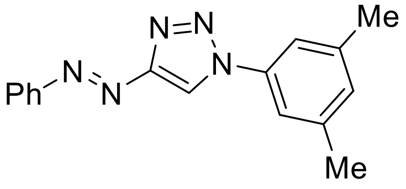

4n

Azotriazole **4n** was prepared via **GP4**, using azoacetylene **2a** (86 mg, 0.30 mmol, 1.0 equiv), CuI (5.7 mg, 0.030 mmol, 10 mol%), TBAB (19 mg, 0.060 mmol, 20 mol%), 1-azido-3,5-dimethylbenzene[22] (44 mg, 0.30 mmol, 1.0 equiv), and CsF (aq. 1.5 M, 0.20 mL, 0.30 mmol, 1.0 equiv) and the reaction was stirred for 24 h. After work-up, the crude product was purified via column chromatography (hexane:ethyl acetate = 8.5:1.5, R_f = 0.25) to give **4n** (35 mg, 0.13 mmol, 42%, *E*:*Z* = 5.5:1) as a red oil.

^{1}H-NMR (400 MHz, $CDCl_3$): δ / ppm = 8.36 (s, 1H), 8.02 – 7.92 (m, 2H), 7.55 – 7.49 (m, 3H), 7.43 (dt, *J* = 1.5, 0.7 Hz, 2H), 7.10 (tt, *J* = 1.6, 0.8 Hz, 1H), 2.41 (d, *J* = 0.8 Hz, 6H).

Note: Peaks of the main diastereomer are given.

^{13}C-NMR (101 MHz, $CDCl_3$): δ / ppm = 160.9, 152.7, 136.7, 131.9, 131.1, 129.3, 123.2, 120.3, 118.4, 112.8, 21.4.

Note: Peaks of the main diastereomer are given.

IR (Diamond-ATR, neat): $\tilde{\nu}$ / cm^{-1} = 3135, 3063, 2919, 1617, 1597, 1489, 1351, 1071, 1036, 847, 829, 763, 685.

HRMS (ESI) calcd. for $C_{16}H_{16}N_5$ $[M+H]^+$: 278.1400, found 278.1400

[22] Illam, P. M.; Donthireddy, S. N. R.; Chakrabartty, S.; Rit, A. *Organometallics* **2019**, *38*, 2610-2623.

(*E*)-1-(4-Fluorophenyl)-4-(phenyldiazenyl)-1*H*-1,2,3-triazole (4o)

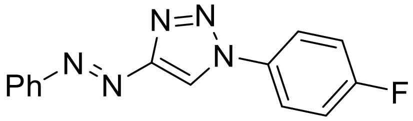

4o

Azotriazole **4o** was prepared via **GP4**, using azoacetylene **2a** (86 mg, 0.30 mmol, 1.0 equiv), CuI (5.7 mg, 0.030 mmol, 10 mol%), TBAB (19 mg, 0.060 mmol, 20 mol%), 1-azido-4-fluorobenzene[23] (41 mg, 0.30 mmol, 1.0 equiv), and CsF (aq. 1.5 M, 0.20 mL, 0.30 mmol, 1.0 equiv) and the reaction was stirred for 24 h. After work-up, the crude product was purified via column chromatography (hexane:ethyl acetate = 8.5:1.5, R_f = 0.25) to give **4o** (26 mg, 97 µmol, 32%, *E*:*Z* = 2.3:1) as a yellow solid.

^{1}H-NMR (400 MHz, $CDCl_3$): δ / ppm = 8.35 (s, 1H), 8.03 – 7.92 (m, 2H), 7.88 – 7.75 (m, 2H), 7.56 – 7.51 (m, 3H), 7.31 – 7.23 (m, 2H).

Note: Peaks of the main diastereomer are given.

^{13}C-NMR (101 MHz, $CDCl_3$): δ / ppm = 162.9 (d, J = 250.4 Hz), 161.1, 147.3 (d, J = 2.0 Hz), 133.1, 132.1, 129.4, 123.3, 122.8 (d, J = 8.7 Hz), 117.1 (d, J = 23.4 Hz), 112.9.

Note: Peaks of the main diastereomer are given.

^{19}F-NMR (377 MHz, $CDCl_3$): δ / ppm = -111.0.

IR (Diamond-ATR, neat): $\tilde{\nu}$ / cm^{-1} = 3129, 3070, 2945, 1506, 1513, 1227, 835, 762, 685, 528.

HRMS (ESI) calcd. for $C_{14}H_{10}FN_5Na$ [M+Na]$^+$: 290.0812, found 290.0815.

---

[23] Zhou, S.; Liao, H.; Liu, M.; Feng, G.; Fu, B.; Li, R.; Cheng, M.; Zhao, Y.; Gong, P. *Bioorg. Med. Chem.* **2014**, *22*, 6438-6452.

(*E*)-4-(Phenyldiazenyl)-1-((phenylthio)methyl)-1*H*-1,2,3-triazole (4p)

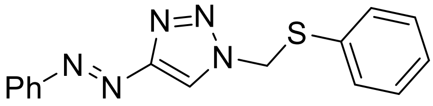

4p

Azotriazole **4p** was prepared via **GP4**, using azoacetylene **2a** (86 mg, 0.30 mmol, 1.0 equiv), CuI (5.7 mg, 0.030 mmol, 10 mol%), TBAB (19 mg, 0.060 mmol, 20 mol%), azidomethyl phenyl sulfide (0.050 g, 0.30 mmol, 1.0 equiv), and CsF (aq. 1.5 M, 0.20 mL, 0.30 mmol, 1.0 equiv) and the reaction was stirred for 24 h. After work-up, the crude product was purified via column chromatography (hexane:ethyl acetate = 8:2, R_f = 0.3) to give **4p** (46 mg, 0.16 mmol, 52%, *E*:*Z* > 20:1) as a yellow solid.

^{1}H-NMR (400 MHz, $CDCl_3$): δ / ppm = 8.02 (s, 1H), 7.97 – 7.89 (m, 2H), 7.55 – 7.45 (m, 3H), 7.40 – 7.27 (m, 5H), 5.68 (s, 2H).

^{13}C-NMR (101 MHz, $CDCl_3$): δ / ppm = 160.7, 152.6, 132.4, 131.9, 131.5, 129.8, 129.3, 129.1, 123.2, 115.0, 54.8.

IR (Diamond-ATR, neat): $\tilde{\nu}$ / cm^{-1} = 3110, 3062, 3010, 2953, 1584, 1483, 1467, 1228, 1000, 740, 686.

HRMS (ESI) calcd. for $C_{15}H_{14}N_5S$ $[M+H]^+$: 296.0964, found 296.0961

1-(adamantan-1-yl)-4-((*E*)-phenyldiazenyl)-1*H*-1,2,3-triazole (4q)

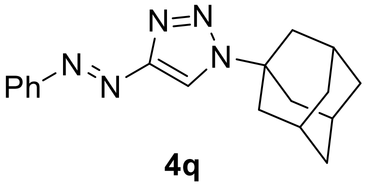

4q

Azotriazole **4q** was prepared via **GP4**, using azoacetylene **2a** (86 mg, 0.30 mmol, 1.0 equiv), CuI (5.7 mg, 0.030 mmol, 10 mol%), TBAB (19 mg, 0.060 mmol, 20 mol%), 1-azidoadamantane (53 mg, 0.30 mmol, 1.0 equiv), and CsF (aq. 1.5 M, 0.20 mL, 0.30 mmol, 1.0 equiv) and the reaction was stirred for 24 h. After work-up, the crude product was purified via column chromatography (hexane:ethyl acetate = 8.5:1.5, R_f = 0.3) to give **4q** (55 mg, 0.18 mmol, 59%, *E*:*Z* > 20:1) as a yellow solid.

^{1}H-NMR (400 MHz, $CDCl_3$): δ / ppm = 8.00 (s, 1H), 7.94 – 7.90 (m, 2H), 7.52 – 7.42 (m, 3H), 2.28 (s, 9H), 1.79 (d, *J* = 3.4 Hz, 6H).

^{13}C-NMR (101 MHz, $CDCl_3$): δ / ppm = 160.1, 152.7, 131.5, 129.2, 123.0, 111.3, 60.5, 42.9, 35.9, 29.5.

IR (Diamond-ATR, neat): $\tilde{\nu}$ / cm^{-1} = 3065, 3133, 2909, 2854, 1506, 1455, 1309, 1203, 1169, 696, 689.

HRMS (ESI) calcd. for $C_{18}H_{22}N_5$ $(M+H)^+$: 308.1870, found 308.1872.

Preparation of Azide Derivative Starting Materials SI-5a-1–SI-5g-1

Tetraacetyl-β-glucopyranosyl azide SI-5a-1 – (2R,3R,4S,5R,6R)-2-(acetoxymethyl)-6-azidotetrahydro-2H-pyran-3,4,5-triyl triacetate

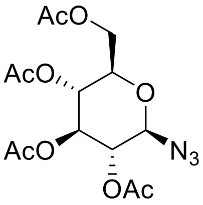

SI-5a-1

SI-5a-1 was prepared from β-glucopyranose pentaacetate (**SI-5a-0)** according to a literature procedure employing Me_3SiN_3 and $SnCl_4$ in CH_2Cl_2.[24]

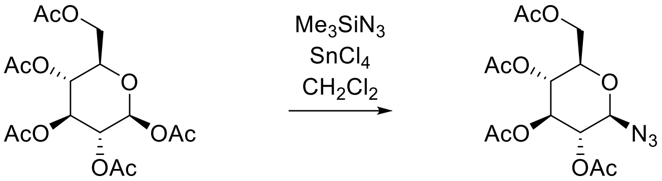

SI-5a-0 **SI-5a-1**

The physical properties of azide **SI-5a-1** were in good accordance with the published data.

[24] Thomas, G. B.; Rader, L. H.; Park, J.; Abezgauz, L.; Danino, D.; DeShong, P.; English, D. S. Carbohydrate Modified Catanionic Vesicles: Probing Multivalent Binding at the Bilayer Interface. J. Am. Chem. Soc. 2009, 131 (15), 5471–5477.

Tamiflu-derived azide SI-5b-1 – ethyl (3R,4R,5S)-4-acetamido-5-azido-3-(pentan-3-yloxy)cyclohex-1-ene-1-carboxylate

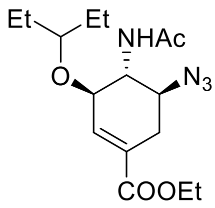

SI-5b-1

SI-5b-1 was prepared from Oseltamivir phosphate (**SI-5b-0**) according to a literature procedure employing FSO_2N_3.[25] The physical properties of the product were in good accordance with the published data.

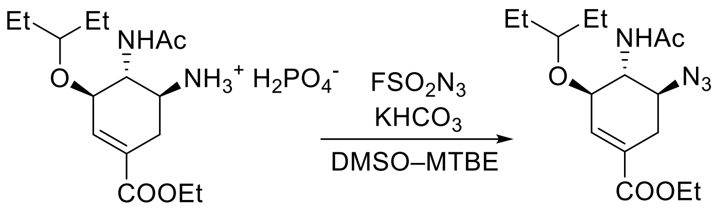

[25] Meng, G.; Guo, T.; Ma, T.; Zhang, J.; Shen, Y.; Sharpless, K. B.; Dong, J. Modular Click Chemistry Libraries for Functional Screens Using a Diazotizing Reagent. Nature 2019, 574 (7776), 86–89.

Arachidonic acid derived azide SI-5c-1 – (5Z,8Z,11Z,14Z)-1-azidoicosa-5,8,11,14-tetraene

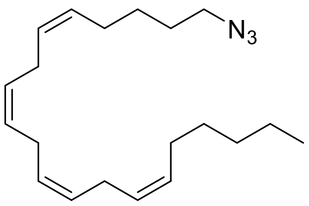

SI-5c-1

SI-5c-1 was prepared from arachidonic acid (**SI-5c-0**) via corresponding alcohol and mesylate according to literature procedures[26,27] employing $LiAlH_4$, MsCl and NaN_3. The physical properties of **SI-5c-1** were in good accordance with the published data.

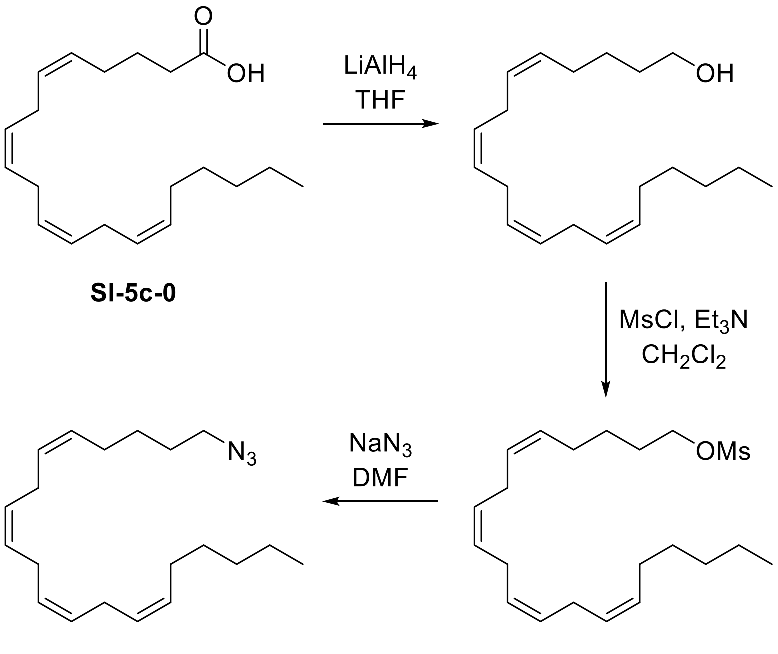

[26] Maharvi, G. M.; Edwards, A. O.; Fauq, A. H. Chemical Synthesis of Deuterium-Labeled and Unlabeled Very Long Chain Polyunsaturated Fatty Acids. Tetrahedron Lett. 2010, 51 (49), 6426–6428.
[27] Parkkari, T.; Savinainen, J. R.; Raitio, K. H.; Saario, S. M.; Matilainen, L.; Sirviö, T.; Laitinen, J. T.; Nevalainen, T.; Niemi, R.; Järvinen, T. Synthesis, Cannabinoid Receptor Activity, and Enzymatic Stability of Reversed Amide Derivatives of Arachidonoyl Ethanolamide. Bioorganic Med. Chem. 2006, 14 (15), 5252–5258.

Biotin derived azide 6a/SI-5d-1

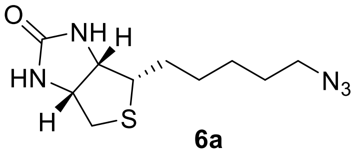

6a

See below for entry **6a**

Colchicine derived azide SI-5f-1 – (S)-7-azido-1,2,3,10-tetramethoxy-6,7-dihydrobenzo[a]heptalen-9(5H)-one

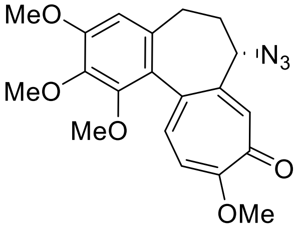

SI-5f-1

SI-5f-1 was prepared from *N*-deacetyl colchicine (**SI-5f-0**) which was prepared from Colchicine according to a literature procedure.[28]

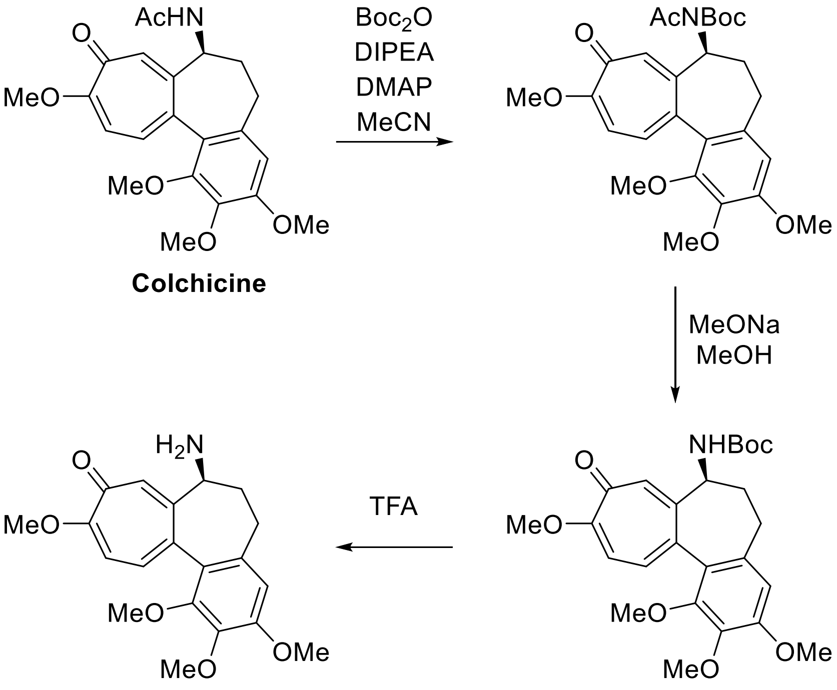

[28] Bagnato, J. D.; Eilers, A. L.; Horton, R. A.; Grissom, C. B. Synthesis and Characterization of a Cobalamin-Colchicine Conjugate as a Novel Tumor-Targeted Cytotoxin. J. Org. Chem. 2004, 69 (26), 8987–8996.

SI-5f-0 was converted to **SI-5f-1** applying a method developed by Sharpless, Dong and co-workers.[29]

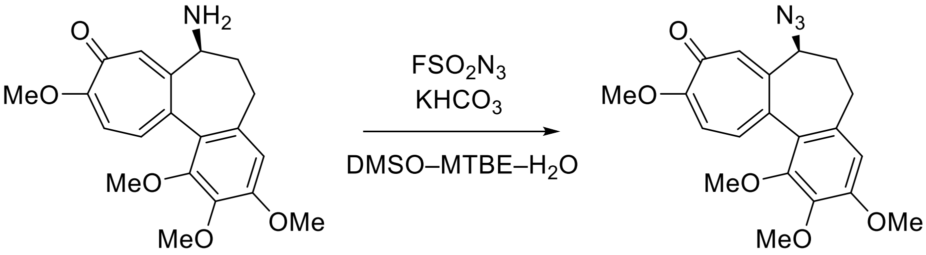

Sequentially, solutions of FSO_2N_3 (prepared according to footnote 29, ~ 0.3 M in DMSO–MTBE as judged by ^{19}F-NMR using TsF as internal standard, 0.33 mL, ~ 0.10 mmol, 1.0 equiv) and of $KHCO_3$ (3.0 M in H_2O, 0.13 mL, 0.39 mmol, 3.9 equiv) were added to solid N-deacteyl colchicine (36 mg, 0.10 mmol, 1.0 equiv). The resulting pale-yellow mixture was further stirred at ambient temperature. After 30 min, the reaction mixture was diluted with EtOAc (20 mL) and the resulting mixture was washed with saturated aqueous NaCl solution (4x10 mL). The organic layer was dried over Na_2SO_4 and concentrated under reduced pressure. Purification of the residue by flash chromatography (CH_2Cl_2:MeOH = 19:1) yielded **SI 5f-1** as pale-yellow oil (27 mg, 70%). Its physical properties were in good accordance with the published data.[30]

---

[29] Meng, G.; Guo, T.; Ma, T.; Zhang, J.; Shen, Y.; Sharpless, K. B.; Dong, J. Modular Click Chemistry Libraries for Functional Screens Using a Diazotizing Reagent. Nature 2019, 574 (7776), 86–89.
[30] Bagnato, J. D.; Eilers, A. L.; Horton, R. A.; Grissom, C. B. Synthesis and Characterization of a Cobalamin-Colchicine Conjugate as a Novel Tumor-Targeted Cytotoxin. J. Org. Chem. 2004, 69 (26), 8987–8996

Docetaxel-derived azide SI-5g-1 – (2aR,4S,4aS,6R,9S,11S,12S,12aR,12bS)-12b-acetoxy-9-(((2R,3S)-3-azido-2-hydroxy-3-phenylpropanoyl)oxy)-4,6,11-trihydroxy-4a,8,13,13-tetramethyl-5-oxo-2a,3,4,4a,5,6,9,10,11,12,12a,12b-dodecahydro-1H-7,11-methanocyclodeca[3,4]benzo[1,2-b]oxet-12-yl benzoate

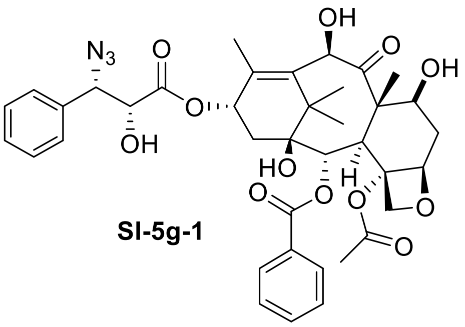

SI-5g-1

SI-5g-1 was prepared from Docetaxel according to a literature procedure. The physical properties of the product were in good accordance with the published data.[31]

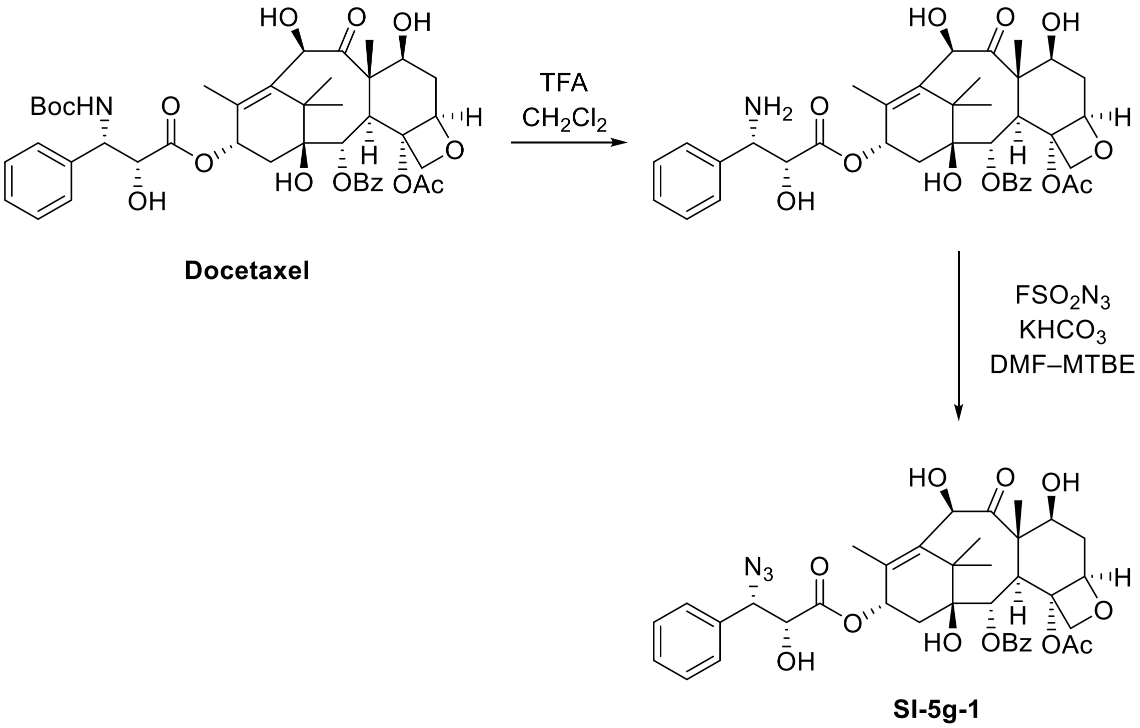

[31] Meng, G.; Guo, T.; Ma, T.; Zhang, J.; Shen, Y.; Sharpless, K. B.; Dong, J. Modular Click Chemistry Libraries for Functional Screens Using a Diazotizing Reagent. Nature 2019, 574 (7776), 86–89.

Preparation of Azotriazole Derivatives 5a–g

(2*S*,3*S*,4*R*,5*S*,6*S*)-2-(acetoxymethyl)-6-(4-((*E*)-(4-methoxyphenyl)diazenyl)-1*H*-1,2,3-triazol-1-yl)tetrahydro-2*H*-pyran-3,4,5-triyl triacetate (5a)

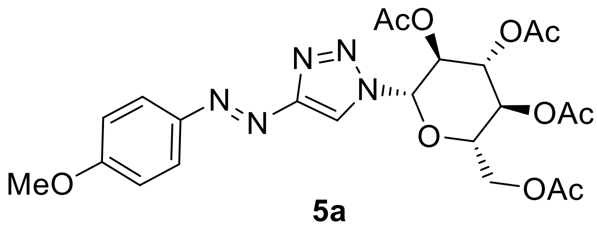

5a

Azotriazole **5a** was prepared via **GP5**, using azoacetylene **2b** (95 mg, 0.30 mmol, 1.0 equiv), CuI (5.7 mg, 0.030 mmol, 10 mol%), TBAB (97 mg, 0.30 mmol, 1.0 equiv), azide **SI-5a-1** (0.11 g, 0.30 mmol, 1.0 equiv), and CsF (aq. 1.5 M, 0.20 mL, 0.30 mmol, 1.0 equiv) and the reaction was stirred for 24 h at 40 °C. After work-up, the crude product was purified via column chromatography (hexane:ethyl acetate = 1:1, R_f = 0.3) to give **5a** (0.11 g, 0.21 mmol, 71%, *E*:*Z* = 3:2) as a yellow solid.

Major diastereomer: (assignment via HSQC/HBMC)

^{1}H-NMR (400 MHz, $CDCl_3$): δ / ppm = 8.16 (s, 1H), 7.97 – 7.91 (m, 2H), 7.02 – 6.98 (m, 2H), 5.95 – 5.89 (m, 1H) 5.51 – 5.43 (m, 2H), 5.28 – 5.22 (m, 1H), 4.30 (d, *J* = 4.9 Hz, 1H), 4.16 (dd, *J* = 12.7, 2.1 Hz, 1H), 4.04 (ddd, *J* = 10.2, 5.1, 2.1 Hz, 1H), 3.88 (s, 3H), 2.07 (d, *J* = 6.3 Hz, 6H), 2.03 (s, 3H), 1.90 (s, 3H).

^{13}C-NMR (101 MHz, $CDCl_3$): δ / ppm = 170.6, 170.0, 169.5, 169.1, 163.0, 160.9, 147.0, 125.4, 114.5, 113.4, 86.5, 75.4, 72.7, 70.5, 67.8, 61.6, 55.7, 20.8, 20.6, 20.6, 20.3.

Minor diastereomer: (assignment via HSQC/HBMC)

^{1}H-NMR (400 MHz, $CDCl_3$): δ / ppm = 8.77 (d, *J* = 2.1 Hz, 1H), 8.10 – 8.05 (m, 2H), 7.06 – 7.02 (m, 2H), 5.41 (t, *J* = 9.5 Hz, 1H), 5.17 (d, *J* = 9.7 Hz, 1H), 5.16 (dd, *J* = 9.2, 2.2 Hz, 1H), 4.96 (t, *J* = 9.4 Hz, 1H), 4.34 (d, *J* = 5.0 Hz, 1H), 4.24 (dd, *J* = 12.4, 2.3 Hz, 1H), 3.92 (ddd, *J* = 10.3, 4.8, 2.4 Hz, 1H), 3.88 (s, 3H), 2.11 (s, 3H), 2.09 (s, 3H), 2.05 (s, 3H), 2.01 (s, 3H).

^{13}C-NMR (101 MHz, $CDCl_3$): δ / ppm = 170.8, 170.4, 169.6, 169.5, 162.2, 161.1, 149.6, 130.1, 121.9, 113.4, 90.8, 74.0, 73.7, 72.2, 68.5, 62.1, 55.8, 20.9, 20.9, 20.74, 20.73.

IR (Diamond-ATR, neat): $\tilde{\nu}$ / cm^{-1} = 2942, 2842, 1747, 1659, 1598, 1515, 1215, 1031, 1067, 730.

HRMS (ESI) calcd. for $C_{23}H_{28}N_5O_{10}$ $[M+H]^+$**:** 534.1831, found 534.1815.

[a]$_D^{25}$ = –32.8 (c = 1.0, $CHCl_3$).

Ethyl (3*R*,4*R*,5*S*)-4-acetamido-5-(4-((*E*)-(2,6-dimethylphenyl)diazenyl)-1*H*-1,2,3-triazol-1-yl)-3-(pentan-3-yloxy)cyclohex-1-ene-1-carboxylate (5b)

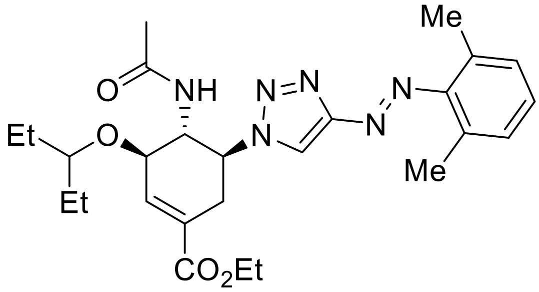

5b

Azotriazole **5b** was prepared via **GP5**, using azoacetylene **2g** (31 mg, 99 µmol, 1.0 equiv), CuI (2.0 mg, 11 µmol, 10 mol%), TBAB (33 mg, 0.10 mmol, 1.0 equiv), tamiflu azide **SI-5b-1** (33 mg, 98 µmol, 1.0 equiv), and CsF (aq. 1.5 M, 0.20 mL, 0.30 mmol, 1.0 equiv) and the reaction was stirred for 12 h at 40 °C. After work-up, the crude product was purified via column chromatography (hexane:ethyl acetate = 5.5:4.5, R_f = 0.25) to give **5b** (41 mg, 83 µmol, 85%, *E*:*Z* > 20:1) as an orange oil.

^{1}H-NMR (400 MHz, $CDCl_3$): δ / ppm = 7.95 (s, 1H), 7.19 – 7.06 (m, 3H), 6.91 – 6.86 (m, 1H), 6.76 (d, *J* = 7.5 Hz, 1H), 5.46 (ddd, *J* = 11.7, 9.4, 7.2 Hz, 1H), 4.77 (dq, *J* = 8.9, 2.2 Hz, 1H), 4.23 (q, *J* = 7.1 Hz, 2H), 4.04 (ddd, *J* = 11.8, 9.0, 7.6 Hz, 1H), 3.38 (p, *J* = 5.7 Hz, 1H), 3.12 – 3.05 (m, 2H), 2.36 (s, 6H), 1.84 (s, 3H), 1.58 – 1.42 (m, 4H), 1.30 (t, *J* = 7.1 Hz, 3H), 0.90 (dt, *J* = 14.7, 7.4 Hz, 6H).

^{13}C-NMR (101 MHz, $CDCl_3$): δ / ppm = 171.7, 165.7, 160.4, 150.5, 138.8, 131.9, 129.4, 129.3, 128.0, 114.6, 82.6, 73.7, 61.3, 57.8, 57.5, 31.6, 26.4, 25.8, 23.4, 19.4, 14.3, 9.7, 9.4.

IR (Diamond-ATR, neat): $\tilde{\nu}$ / cm^{-1} = 3334, 3105, 2936, 2966, 1718, 1661, 1530, 1464, 1440, 1249, 1235, 1079, 945, 774, 583.

HRMS (ESI) calcd. for $C_{26}H_{36}N_6NaO_4$ $[M+Na]^+$: 519.2690, found 519.2687.

[a]$_D^{25}$ = –85.0 (c = 1.0, $CHCl_3$).

Arachidonic acid derived azotriazole 5c – 1-((5Z,8Z,11Z,14Z)-icosa-5,8,11,14-tetraen-1-yl)-4-((E)-(3,4,5-trimethoxyphenyl)diazenyl)-1H-1,2,3-triazole

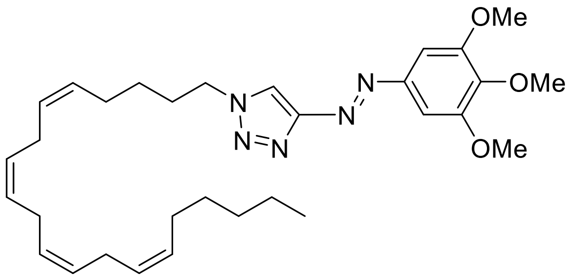

5c

Azotriazole **5c** was prepared via **GP5**, using azoacetylene **2m** (11 mg, 0.030 mmol, 1.0 equiv), CuI (0.6 mg, 3 µmol, 0.1 equiv), TBAB (0.10 g, 0.30 mmol, 1.0 equiv), azide **SI 5c-1** (9.0 mg, 0.030 mmol, 1.0 equiv), and CsF (aq. 1.5 M, 0.020 mL, 0.030 mmol, 1.0 equiv) and the reaction was stirred for 18 h at 40 °C. After work-up, the crude product was purified via column chromatography (22% EtOAc in hexanes to 30% EtOAc in hexanes, Rf = 0.3) to give **5c** (14 mg, 26 µmol, 87%) as a yellow oil.

^{1}H NMR (400 MHz, $CDCl_3$) δ / ppm = 7.92 (s, 1H), 7.28 (s, 2H), 5.52 – 5.26 (m, 8H), 4.43 (t, *J* = 7.2 Hz, 2H), 3.94 (s, 6H), 3.93 (s, 3H), 2.86 – 2.76 (m, 6H), 2.14 (q, *J* = 7.3 Hz, 2H), 2.08 – 1.93 (m, 4H), 1.45 (tt, *J* = 10.3, 6.4 Hz, 2H), 1.37 – 1.20 (m, 6H), 0.87 (t, *J* = 6.8 Hz, 3H).

^{13}C NMR (101 MHz, $CDCl_3$) δ / ppm = 160.5, 153.6, 148.5, 141.3, 130.7, 129.0, 129.0, 128.8, 128.4, 128.1, 127.9, 127.6, 114.7, 100.8, 61.2, 56.4, 51.1, 31.6, 29.8, 29.4, 27.3, 26.6, 26.5, 25.8 (three signals overlap), 22.7, 14.2.

IR (film): 3135, 3010, 2929, 2857, 1597, 1495, 1466, 1431, 1413, 1377, 1351, 1309, 1220, 1180, 1127, 1036, 1005, 923, 845, 714, 687, 630, 532 (cm^{-1}).

HRMS (ESI): calculated for $C_{31}H_{45}N_5NaO_3$ [M+Na$^+$]: 558.3415, found 558.3414

(3a*S*,4*S*,6a*R*)-4-(5-(4-((*E*)-(4-methoxyphenyl)diazenyl)-1*H*-1,2,3-triazol-1-yl)pentyl)tetrahydro-1*H*-thieno[3,4-*d*]imidazol-2(3*H*)-one (5d)

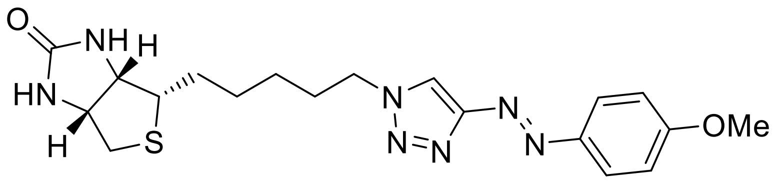

5d

Azotriazole **5d** was prepared via **GP5**, using azoacetylene **2b** (63 mg, 0.20 mmol, 1.0 equiv), CuI (4.0 mg, 21µmol, 10 mol%), TBAB (65 mg, 0.20 mmol, 1.0 equiv), biotin azide **6a** (51 mg, 0.20 mmol, 1.0 equiv), and CsF (aq. 1.5 M, 0.13 mL, 0.20 mmol, 1.0 equiv) and the reaction was stirred for 12 h at 40 °C. After work-up, the product was obtained by filtration of the reaction mixture and washing with DCM to give solid **5d** (23 mg, 55 µmol). Further pure material was obtained by column chromatography of the filtrate (DCM:MeOH = 9.5:0.5, R_f = 0.35) to give **5d** (50 mg, 0.12 mmol) as a light-brown solid. Combining product by filtration and column chromatography gave **5d** (73 mg, 0.18 mmol*, E*:*Z* > 20:1) in 88% yield.

^{1}H-NMR (400 MHz, DMSO-d$_6$): δ / ppm = 8.58 (s, 1H), 7.86 (d, *J* = 8.6 Hz, 2H), 7.14 (d, *J* = 8.6 Hz, 2H), 6.39 (d, *J* = 30.8 Hz, 2H), 4.42 (t, *J* = 7.0 Hz, 2H), 4.29 (dd, *J* = 7.7, 5.0 Hz, 1H), 4.12 (t, *J* = 5.6 Hz, 1H), 3.87 (s, 3H), 3.08 (dd, *J* = 8.0, 4.8 Hz, 1H), 2.81 (dd, *J* = 12.4, 5.1 Hz, 1H), 2.57 (d, *J* = 12.4 Hz, 1H), 1.89 (d, *J* = 7.4 Hz, 2H), 1.60 (d, *J* = 8.2 Hz, 1H), 1.50 – 1.21 (m, 5H).

^{13}C-NMR (101 MHz, DMSO-d$_6$): δ / ppm = 187.8, 162.7, 162.2, 159.9, 146.3, 124.4, 115.5, 114.7, 61.0, 59.2, 55.7, 55.4, 50.0, 29.3, 28.1, 27.9, 25.8.

IR (Diamond-ATR, neat): $\tilde{\nu}$ / cm^{-1} = 3524, 3215, 3130, 2837, 1698, 1681, 1602, 1469, 1445, 1243, 1148, 1035, 843, 600.

HRMS (ESI) calcd. for $C_{19}H_{26}N_7O_2S$ [M+H]$^+$: 416.1863, 416.1860.

[a]$_D^{25}$ = 13.8 (c = 0.327, DMSO).

(8*R*,9*S*,13*S*,14*S*,17*S*)-17-((4-((*E*)-(1-(cyclohexylmethyl)-1*H*-1,2,3-triazol-4-yl)diazenyl)phenyl)ethynyl)-13-methyl-7,8,9,11,12,13,14,15,16,17-decahydro-6*H*-cyclopenta[*a*]phenanthrene-3,17-diol (5e)

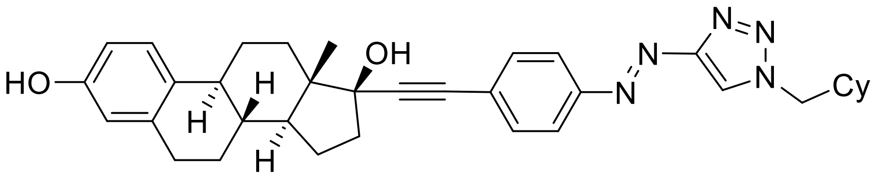

5e

Azotriazole **5e** was prepared via **GP5**, using azoacetylene **2o** (29 mg, 0.050 mmol, 1.0 equiv), CuI (1 mg, 5 µmol, 0.1 equiv), TBAB (16 mg, 0.050 mmol, 1.0 equiv), cyclohexylmethyl azide (7.0 mg, 0.050 mmol, 1.0 equiv), and CsF (aq. 1.5 M, 33 µL, 0.050 mmol, 1.0 equiv) and the reaction was stirred for 12 h at 40 °C. After work-up, the crude product was purified via column chromatography (hexane:ethyl acetate = 6:4, R_f = 0.2) to give **5e** (20 mg, 36 µmol, 71%, *E*:*Z* = 13:1) as a yellow oil.

^{1}H-NMR (400 MHz, $CDCl_3$): δ / ppm = 7.93 (s, 1H), 7.91 – 7.87 (m, 2H), 7.60 – 7.54 (m, 2H), 7.16 (dd, *J* = 8.6, 1.0 Hz, 1H), 6.66 (dd, *J* = 8.4, 2.8 Hz, 1H), 6.59 (d, *J* = 2.7 Hz, 1H), 4.26 (d, *J* = 7.2 Hz, 2H), 2.f90 – 2.73 (m, 2H), 2.50 – 2.32 (m, 2H), 2.24 (td, *J* = 10.9, 4.0 Hz, 1H), 2.12 (ddd, *J* = 13.8, 11.7, 3.6 Hz, 1H), 2.03 – 1.60 (m, 11H), 1.58 – 1.32 (m, 4H), 1.31 – 1.12 (m, 4H), 1.10 – 0.98 (m, 2H), 0.95 (s, 3H), 0.91 – 0.81 (m, 1H).

Note: $CDCl_3$ contained TMS as internal reference (Peak at 0.00 ppm).

^{13}C-NMR (101 MHz, $CDCl_3$): δ / ppm = 160.4, 153.6, 151.8, 138.3, 132.6, 132.5, 126.7, 126.4, 123.1, 115.7, 115.4, 112.9, 95.9, 85.8, 80.6, 57.4, 50.0, 47.9, 43.8, 39.6, 39.2, 38.8, 33.3, 30.6, 29.8, 27.3, 26.6, 26.1, 25.6, 23.1, 13.0.

IR (Diamond-ATR, neat): $\tilde{\nu}$ / cm^{-1} = 3325, 2926, 2954, 1729, 1610, 1597, 1497, 1468, 1404, 1286, 964, 907, 728.

HRMS (ESI) calcd. for $C_{35}H_{42}N_5O_2$ $[M+H]^+$: 564.3333, found 564.3339.

[a]$_D^{25}$ = –40.1 (c = 1.0, $CHCl_3$).

Colchicine-derived azotriazole 5f – (S,E)-3-hydroxy-1,2,10-trimethoxy-7-(4-((4-(methylthio)phenyl)diazenyl)-1H-1,2,3-triazol-1-yl)-6,7-dihydrobenzo[a]heptalen-9(5H)-one

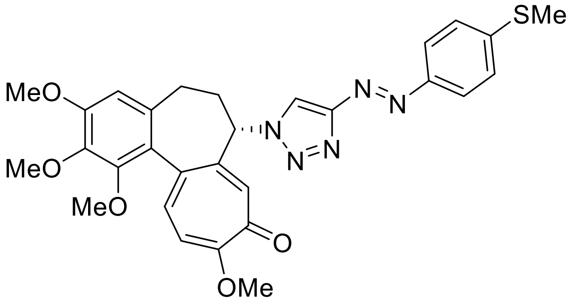

5f

Azotriazole **5f** was prepared via **GP5**, using azoacetylene **2c** (14 mg, 0.050 mmol, 1.0 equiv), CuI (2.0 mg, 0.010 µmol, 10 mol%), TBAB (16 mg, 0.050 mmol, 1.0 equiv), azide **SI 5f-1** (19 mg, 0.050 mmol, 1.0 equiv), and CsF (aq. 1.5 M, 0.036 mL, 0.054 mmol, 1.1 equiv) and the reaction was stirred for 24 h at 40 °C. After work-up, the crude product was purified via column chromatography (EtOAc:MeOH = 50:1, R_f = 0.15) to give **5f** (13 mg, 23 µmol, 46%) as a dark yellow oil.

The product consisted of a mixture of *E*/*Z* isomers in a ratio of ~10:1, for clarity only NMR data of the major isomer is reported.

^{1}H NMR (500 MHz, $CDCl_3$) δ / ppm = 8.04 (s, 1H), 7.87 (d, *J* = 8.2 Hz, 2H), 7.35-7.30 (m, 3H), 6.81 (d, *J* = 10.9 Hz, 1H), 6.60 (s, 1H), 6.58 (s, 1H), 5.50 (dd, *J* = 12.1, 4.9 Hz, 1H), 3.97 (s, 3H), 3.94 (s, 3H), 3.92 (s, 3H), 3.77 (s, 3H), 2.82-2.72 (m, 2H), 2.67–2.57 (m, 2H), 2.54 (s, 3H).

^{13}C NMR (126 MHz, $CDCl_3$) δ / ppm = 178.9, 171.3, 164.5, 154.2, 151.2, 147.1, 144.4, 141.9, 135.7, 134.4, 133.5, 131.8, 126.7, 125.9, 125.0, 123.7, 114.9, 111.9, 107.6, 63.4, 61.5, 61.3, 56.6, 56.3, 35.6, 29.8, 15.2.

IR (film): 2931, 2852, 1722, 1586, 1487, 1399, 135, 1322, 1254, 1139, 1088, 1020, 832, 752 (cm^{-1}).

HRMS (ESI) calculated for $C_{29}H_{29}N_5NaO_5S$ [M+Na$^+$]: 582.1782, found: 582.1779.

[α]$_D$ $^{25°}$= +22.4 (c = 0.98, $CHCl_3$).

Docetaxel derived azotriazole 5g – (2aR,4aS,6R,9S,11S,12S,12aR,12bS)-12b-acetoxy-4,6,11-trihydroxy-9-(((2R,3S)-2-hydroxy-3-(4-((E)-(4-methoxyphenyl)diazenyl)-1H-1,2,3-triazol-1-yl)-3-phenylpropanoyl)oxy)-4a,8,13,13-tetramethyl-5-oxo-2a,3,4,4a,5,6,9,10,11,12,12a,12b-dodecahydro-1H-7,11-methanocyclodeca[3,4]benzo[1,2-b]oxet-12-yl benzoate

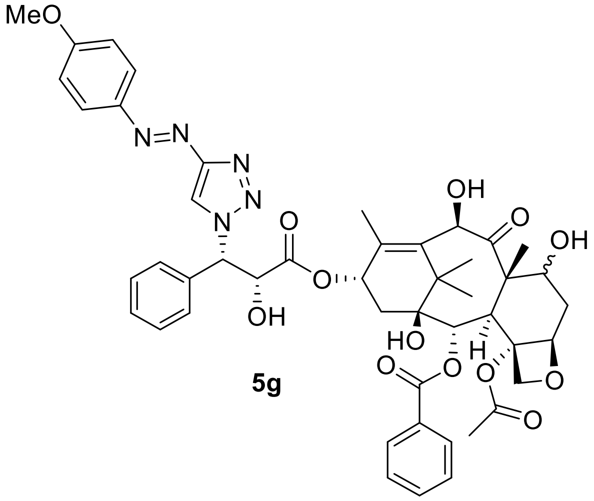

5g

Azotriazoles **5g** (two separable epimers[32]) were prepared via **GP5**, using azoacetylene **2b** (16 mg, 0.051 mmol, 1.9 equiv), CuI (0.80 mg, 4.0 µmol, 15 mol%), TBAB (13 mg, 40 µmol, 1.5 equiv), azide **SI-5g-1** (20 mg, 0.027 mmol, 1.0 equiv), and CsF (aq. 1.5 M, 0.030 mL, 0.045 mmol, 1.7 equiv) and the reaction was stirred for 15 h at 40 °C. After work-up, the crude product was purified via column chromatography (acetone in hexanes = 40% → 42.5%; hexane:acetone 3:2 R_f = 0.18 and 0.10 respectively) to give firstly **(*7R*)-5g** (8.0 mg, 9.0 µmol , 33%) as a dark yellow oil, followed by **(*7S*)-5g** (7.0 mg, 8.0 µmol, 29%) as a dark yellow oil.

[32] Randall, J. D.; Eyckens, D. J.; Stojcevski, F.; Francis, P. S.; Doeven, E. H.; Barlow, A. J.; Barrow, A. S.; Arnold, C. L.; Moses, J. E.; Henderson, L. C. Modification of Carbon Fibre Surfaces by Sulfur-Fluoride Exchange Click Chemistry. ChemPhysChem 2018, 19 (23), 3176–3181.

(7*S*)-5g

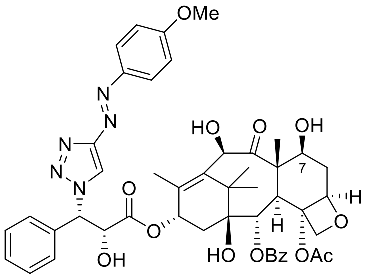

^{1}H NMR (600 MHz, MeOD) δ / ppm = 8.58 (s, 1H), 8.09 – 8.06 (m, 2H), 7.94 – 7.91 (m, 2H), 7.70 – 7.66 (m, 1H), 7.60 – 7.55 (m, 4H), 7.46 (td, *J* = 7.9, 1.4 Hz, 2H), 7.35 (td, *J* = 7.4, 1.4 Hz, 1H), 7.10 – 7.07 (m, 2H), 6.17 (d, *J* = 7.8 Hz, 1H), 6.09 (td, *J* = 9.1, 1.7 Hz, 1H), 5.62 (d, *J* = 7.2 Hz, 1H), 5.30 (d, *J* = 7.8 Hz, 1H), 5.23 (s, 1H), 4.99 (dd, *J* = 9.7, 2.2 Hz, 1H), 4.22 – 4.17 (m, 3H), 3.90 (d, *J* = 1.5 Hz, 3H), 3.85 (d, *J* = 7.3 Hz, 1H), 2.45 – 2.41 (m, 1H), 2.40 (s, 3H), 2.12 (dd, *J* = 15.3, 9.3 Hz, 1H), 1.85 – 1.78 (m, 5H), 1.69 (s, 3H), 1.13 (s, 3H), 1.10 (s, 3H).

^{13}C NMR (151 MHz, MeOD) δ / ppm = 211.0, 172.8, 171.8, 167.6, 164.6, 161.4, 148.2, 138.9, 138.1, 136.1, 134.6, 131.5, 131.1, 130.6, 130.2, 129.7, 129.2, 126.0, 117.9, 115.5, 86.0, 82.3, 79.2, 77.6, 76.4, 75.6, 74.7, 72.6, 72.6, 69.3, 58.9, 56.2, 47.9, 44.5, 37.5, 36.8, 27.0, 23.2, 21.6, 14.4, 10.4.

IR (film) 3417, 2920, 1717, 1600, 1584, 1501, 1452, 1418, 1371, 1315, 1250, 1179, 1146, 1108, 1070, 1026, 982, 948, 916, 889, 841, 810, 753, 710, 667, 530, 418 (cm^{-1}).

HRMS (ESI) calculated for $C_{47}H_{51}N_5NaO_{13}$ [M+Na$^+$]: 916.3376, found: 916.3378.

[α]$_D$ $^{25°}$= –10.5 (c = 0.2, $CHCl_3$).

(*7R*)-5g

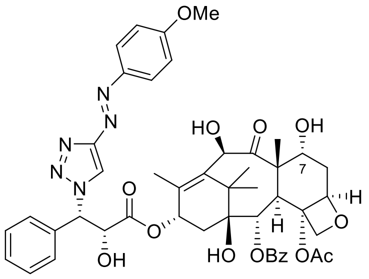

^{1}H NMR (500 MHz, MeOD) δ / ppm = 8.59 (s, 1H), 8.09 (d, *J* = 7.5 Hz, 2H), 7.92 (d, *J* = 9.7 Hz, 2H), 7.80 – 7.66 (m, 1H), 7.64 – 7.53 (m, 4H), 7.47 (t, *J* = 7.6 Hz, 2H), 7.34 (t, *J* = 7.3 Hz, 1H), 7.08 (dt, *J* = 9.5, 2.4 Hz, 2H), 6.17 (d, *J* = 7.8 Hz, 1H), 6.06 (t, *J* = 9.1 Hz, 1H), 5.68 (d, *J* = 7.5 Hz, 1H), 5.44 (s, 1H), 5.29 (d, *J* = 7.8 Hz, 1H), 4.97 (t, *J* = 6.6 Hz, 1H), 4.42 (d, *J* = 8.6 Hz, 1H), 4.28 (d, *J* = 8.6 Hz, 1H), 3.94 – 3.82 (m, 4H), 3.59 (bs, 1H), 2.49 (s, 3H), 2.30 – 2.23 (m, 2H), 2.16 – 2.11 (m, 1H), 1.83 (dd, *J* = 15.5, 8.6 Hz, 1H), 1.71 (s, 3H), 1.66 (s, 3H), 1.11 (s, 3H), 1.08 (s, 3H).

^{13}C NMR (126 MHz, MeOD) δ /ppm = 215.4, 173.9, 172.7, 167.7, 164.6, 148.2, 138.5, 137.9, 136.1, 134.7, 131.5, 131.1, 130.6, 130.2, 129.8, 129.2, 126.0, 115.5, 83.9, 83.4, 79.4, 79.0, 78.8, 77.5, 77.0, 74.7, 72.5, 69.4, 58.5, 56.2, 56.1, 43.9, 41.4, 37.2, 36.2, 29.5, 26.4, 23.2, 21.3, 17.3, 14.7.

IR (film) 3443, 2935, 1716, 1601, 1502, 1452, 1374, 1255, 1180, 1147, 1108, 1069, 1027, 988, 841, 711, 485 (cm^{-1}).

HRMS (ESI) calculated for $C_{47}H_{51}N_5NaO_{13}$ [$M+Na^+$]: 916.3376, found: 916.3362.

[α]$_D$ $^{25°}$= +356.8 (c = 0.49, $CHCl_3$).

Preparation of Azide Derivatives 6a–d

Biotin-derived azide (6a) – (3aS,4S,6aR)-4-(5-azidopentyl)tetrahydro-1H-thieno[3,4-d]imidazol-2(3H)-one

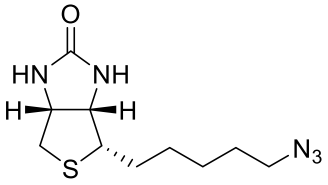

6a

6a was derived from Biotin via a modified literature procedure.[33,34]

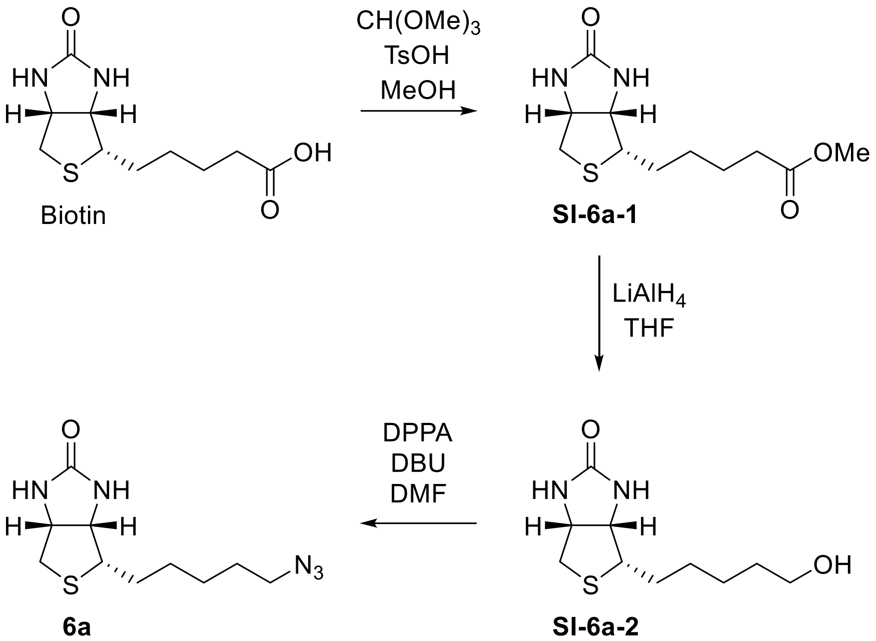

SI-6a-1

Solid TsOH·H_2O (38 mg, 0.20 mmol, 10 mol%) was added to a stirred suspension/solution of D-Biotin (0.49 g, 2.0 mmol, 1.0 equiv) and trimethyl orthoformate (2.2 mL, 0.020 mol, 10 equiv) in MeOH (10 mL) and the resulting mixture was heated to reflux. After 2 h, the reaction mixture was cooled to ambient temperature and concentrated under reduced pressure. Purification of the residue by flash chromatography (10% MeOH in CH_2Cl_2 to 15% MeOH in CH_2Cl_2) yielded

[33] Siebertz, K. D.; Hackenberger, C. P. R. Chemoselective Triazole-Phosphonamidate Conjugates Suitable for Photorelease. Chem. Commun. 2018, 54 (7), 763–766.
[34] Fang, L.; Trigiante, G.; Kousseff, C. J.; Crespo-Otero, R.; Philpott, M. P.; Watkinson, M. Biotin-Tagged Fluorescent Sensor to Visualize "mobile" Zn2+ in Cancer Cells. Chem. Commun. 2018, 54 (69), 9619–9622.

biotin methyl ester **SI-6a-1** (0.46 g, 89%). Its physical properties were in good accordance with the published data (Chem. Commun. 2018, 54 (7), 763–766).

SI-6a-2

A solution of $LiAlH_4$ (1.0 M in THF, 3.0 mL, 3.0 mmol, 1.8 equiv) was added slowly to a stirred solution/suspension of biotin methyl ester (0.46 g, 1.7 mmol, 1.0 equiv) in anhydrous THF (60 mL) at ambient temperature. After the addition, the reaction mixture was stirred for another 15 min, before sequentially H_2O (0.12 mL), aqueous NaOH solution (3 M, 0.12 mL) and H_2O (0.36 mL) were added carefully. The resulting thick suspension was filtered through a plug of celite. The residue was rinsed with THF (4x30 mL) and a mixture of CH_2Cl_2:MeOH (9:1, 5x50 mL). The combined filtrate was concentrated under reduced pressure to yield crude biotinol **SI-6a-2** This intermediate was used directly in the next step.

6a

DBU (0.80 mL, 5.3 mmol, 3.1 equiv) was added to a stirred solution of crude **SI-6a-2** (assumed 1.7 mmol) and DPPA (1.1 mL, 5.1 mmol, 3.0 equiv) in anhydrous DMF (15 mL) at 0 °C. After 15 min at 0 °C, the reaction mixture was heated to 65 °C. After 19 h, the reaction mixture was cooled to ambient temperature and poured into H_2O (200 mL). The resulting mixture was extracted with EtOAc (3x100 mL). The combined organic layers were dried over Na_2SO_4 and concentrated under reduced pressure. Purification of the residue by flash chromatography (4.75% MeOH in CH_2Cl_2 to 6.25% MeOH in CH_2Cl_2 to 9.1% MeOH in CH_2Cl_2) yielded azide **6a** as colorless solid (0.27 g, 63%, over two steps). Its physical properties were in good accordance with the published data (Chem. Commun. 2018, 54, 9619-9622).

Androstanolone-derived azide (6b) – (5S,8R,9S,10S,13S,14S,17R)-17-azido-10,13-dimethylhexadecahydro-3H-cyclopenta[a]phenanthren-3-one

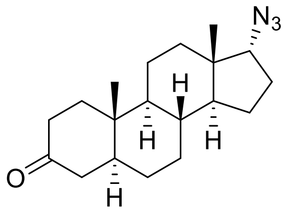

6b

6b was prepared from androstanolone according to a modified literature procedure,[35] substituting DMPU for HMPA in the second step. The physical properties of azide **6b** were in good accordance with the published data.

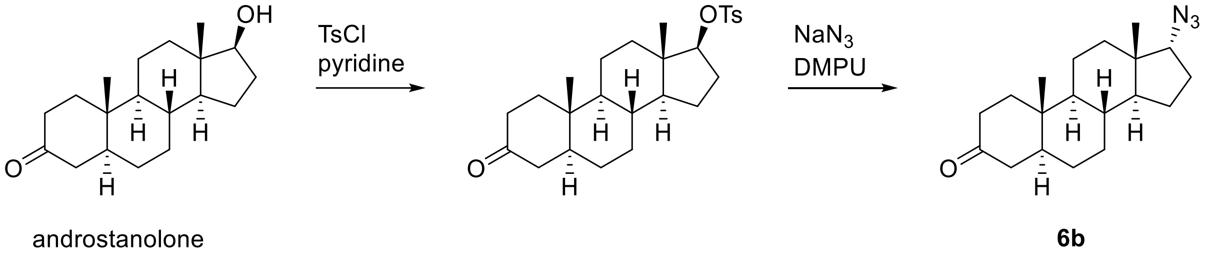

[35] Vidrna, L.; Černý, I.; Pouzar, V.; Borovská, J.; Vyklický, V.; Vyklický, L.; Chodounská, H. Azido Analogs of Neuroactive Steroids. Steroids 2011, 76 (10–11), 1043–1050.

Lenalidomide azide 6c – 3-(4-azido-1-oxoisoindolin-2-yl)piperidine-2,6-dione

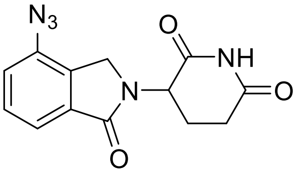

6c

6c was prepared from lenalidomide according to a literature procedure employing FSO_2N_3,[36] with the modification that the reaction was diluted by addition of additional DMSO (to a total concentration of 0.1 M) and only 1.0 equiv of FSO_2N_3 was employed. After stirring for 48h at rt, sequential addition of aq. sodium ascorbate (0.15 M) and acetone led to selective precipitation of **6c** which was isolated by filtration. The physical properties of the product were in good accordance with the published data.

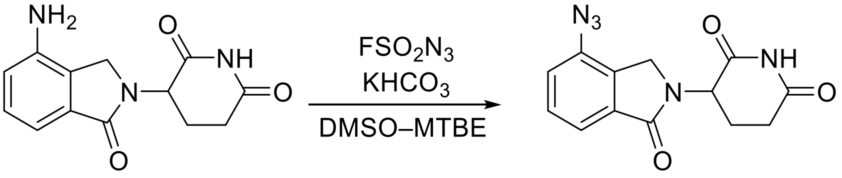

[36] Meng, G.; Guo, T.; Ma, T.; Zhang, J.; Shen, Y.; Sharpless, K. B.; Dong, J. Modular Click Chemistry Libraries for Functional Screens Using a Diazotizing Reagent. Nature 2019, 574 (7776), 86–89.

JQ1-azide 6d – (S)-6-(azidomethyl)-4-(4-chlorophenyl)-2,3,9-trimethyl-6H-thieno[3,2-f][1,2,4]triazolo[4,3-a][1,4]diazepine

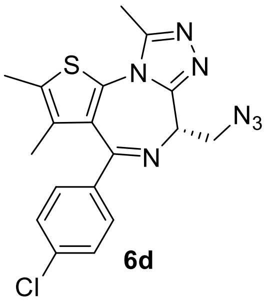

6d

6d was generated from JQ1-carboxylic acid which was converted to JQ-1 amine (**SI-6d-1**) according to a literature procedure.[37] The physical properties of **SI-6d-1** were in good accordance with the published data.

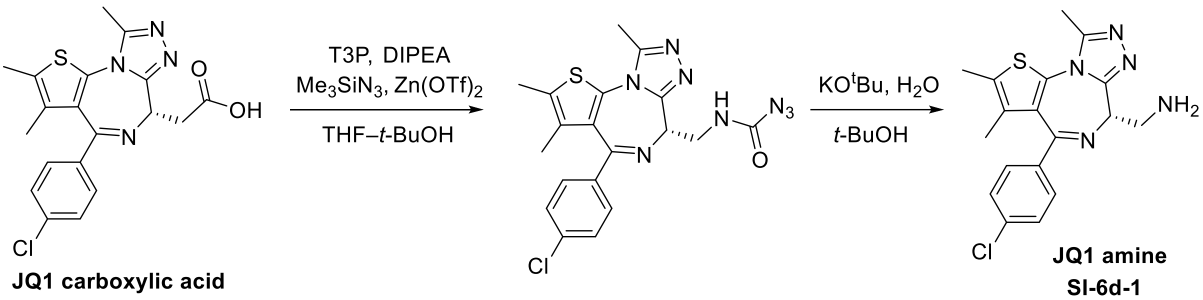

JQ1 carboxylic acid

JQ1 amine
SI-6d-1

[37] Pfaff, P.; Samarasinghe, K. T. G.; Crews, C. M.; Carreira, E. M. Reversible Spatiotemporal Control of Induced Protein Degradation by Bistable PhotoPROTACs. ACS Cent. Sci. 2019, 5 (10), 1682–1690.

SI-6d-1 was transformed to **6d** via a method of Sharpless, Dong and co-workers.[38]

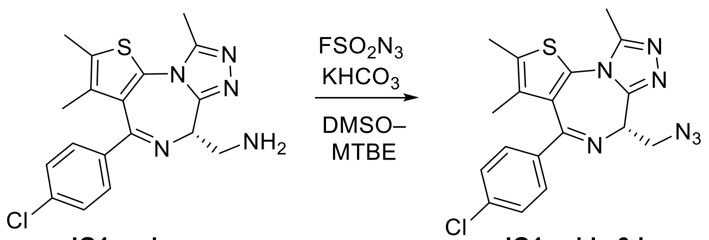

JQ1 amine
SI-6d-1

JQ1 azide 6d

Sequentially, solutions of FSO_2N_3 (prepared according to footnote 38, ~ 0.3 M in DMSO/MTBE as judged by ^{19}F-NMR using TsF as internal standard, 0.20 mL, ~ 0.060 mmol, 1.2 equiv) and of $KHCO_3$ (3.0 M in H_2O, 0.067 mL, 0.20 mmol, 4.0 equiv) were added to solid **JQ1 amine** (**SI-6d-1**) (18 mg, 0.050 mmol, 1.0 equiv). The resulting yellow mixture was stirred at ambient temperature. After 30 min, a second batch of FSO_2N_3 solution (~ 0.3 M in DMSO/MTBE, 0.10 mL, ~ 0.030 mmol, 0.60 equiv) and of $KHCO_3$ (3.0 M in H_2O, 0.067 mL, 0.20 mmol, 4.0 equiv) were added. After 1 h, the reaction mixture was diluted with EtOAc (15 mL) and the resulting mixture was washed with saturated aqueous NaCl solution (3x10 mL). The organic layer was dried over Na_2SO_4 and concentrated under reduced pressure. Purification of the residue by flash chromatography (CH_2Cl_2:MeOH = 30:1 → 20:1) yielded **6d** as pale-yellow oil (12 mg, 60%).

^{1}H NMR (500 MHz, $CDCl_3$) δ / ppm = 7.48 (d, J = 8.2 Hz, 2H), 7.36 (d, J = 8.7 Hz, 2H), 4.51 (dd, J = 12.5, 8.9 Hz, 1H), 4.28 (dd, J = 8.8, 4.4 Hz, 1H), 4.14 (dd, J = 12.6, 4.4 Hz, 1H), 2.70 (s, 3H), 2.42 (d, J = 0.8 Hz, 3H), 1.72 (d, J = 0.8 Hz, 3H).

^{13}C NMR (126 MHz, $CDCl_3$) δ / ppm = 164.9, 154.0, 150.0, 137.2, 136.5, 132.2, 131.3, 131.1, 130.5, 130.1, 129.0, 57.7, 52.2, 14.7, 13.3, 11.9.

IR (film): 2925, 2855, 2223, 2098, 1718, 1591, 1565, 1528, 1487, 1442, 1419, 1399, 1379, 1362, 1309, 1275, 1221, 1175, 1089, 1014, 989, 962, 910, 839, 802, 730, 697, 666, 645, 607, 596, 562, 536, 523, 500, 485 (cm^{-1}).

HRMS (ESI) calculated for $C_{18}H_{17}ClN_7S$ [M+H$^+$]: 398.0949, found 398.0939.

[α]$_D$ $^{25°}$= + 51.1 (c = 0.033, $CHCl_3$).

---

[38] Meng, G.; Guo, T.; Ma, T.; Zhang, J.; Shen, Y.; Sharpless, K. B.; Dong, J. Modular Click Chemistry Libraries for Functional Screens Using a Diazotizing Reagent. Nature 2019, 574 (7776), 86–89.

Preparation of Diacetylene Platform Reagent 2q – (E)-1-(4-(prop-2-yn-1-yloxy)phenyl)-2-((triisopropylsilyl)ethynyl)diazene

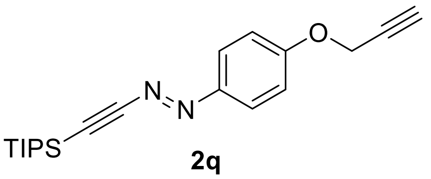

2q

2q was generated starting from 4-nitrophenol which was transformed to aniline **SI-2q-1** following a literature-known procedure.[39] The physical properties of the product were in good accordance with the reported data.

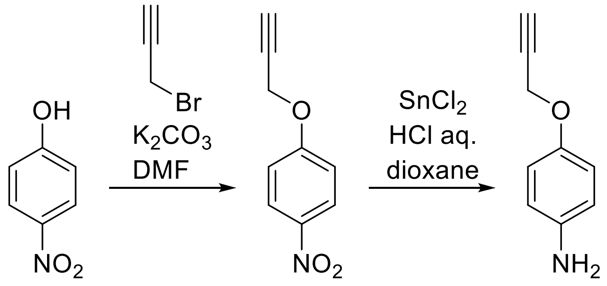

SI-2q-1

[39] Wang, X.; Zhang, Y.; Tan, H.; Wang, Y.; Han, P.; Wang, D. Z. Enantioselective Organocatalytic Mannich Reactions with Autocatalysts and Their Mimics. J. Org. Chem. 2010, 75 (7), 2403–2406.

SI-2q-1 was transformed to the corresponding aryldiazonium tetrafluoroborate salt **SI-2q-2** (4-Propynyloxyphenyldiazonium tetrafluoroborate) following **GP1.** Its physical properties were in good accordance with reported data.[40]

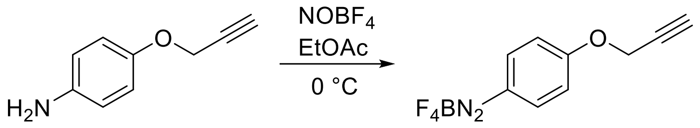

SI-2q-1 **SI-2q-2**

A solution of 4-propynyloxyaniline (0.52 g, 3.5 mmol, 1.0 equiv) in anhydrous EtOAc (2.0 mL) was added dropwise to a stirred solution of $NOBF_4$ (0.50 g, 4.3 mmol, 1.2 equiv) in anhydrous EtOAc (12 mL, 0.25 M) at 0 °C. After the addition, the reddish-brown reaction mixture was warmed to ambient temperature under exclusion of light. After 2 h, the reaction mixture was diluted with ice-cold Et_2O (15 mL) and the resulting suspension was cooled to 0 °C. The brown precipitate was filtered off, washed with ice-cold Et_2O (2x20 mL) and dried under reduced pressure to yield the crude diazonium salt (0.63 g, 2.6 mmol, 73%).

^{1}H NMR (400 MHz, CD_3CN) δ / ppm = 8.43 (d, *J* = 9.4 Hz, 2H), 7.40 (d, *J* = 9.5 Hz, 2H), 5.02 (d, *J* = 2.4 Hz, 2H), 3.02 (t, *J* = 2.4 Hz, 1H).

^{13}C NMR (101 MHz, CD_3CN) δ / ppm = 168.8, 136.6, 119.4, 104.0, 79.1, 77.0, 58.8.

^{19}F-NMR (377 MHz, CD_3CN) δ / ppm = -151.5.

[40] Randall, J. D.; Eyckens, D. J.; Stojcevski, F.; Francis, P. S.; Doeven, E. H.; Barlow, A. J.; Barrow, A. S.; Arnold, C. L.; Moses, J. E.; Henderson, L. C. Modification of Carbon Fibre Surfaces by Sulfur-Fluoride Exchange Click Chemistry. ChemPhysChem 2018, 19 (23), 3176–3181.

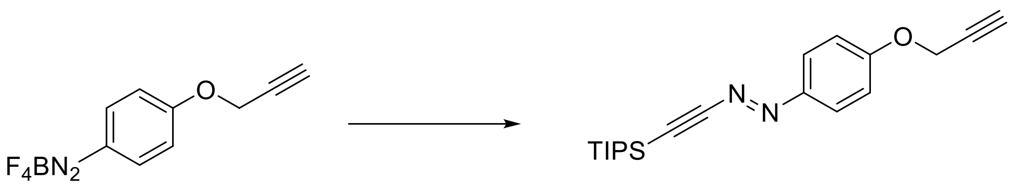

SI-2q-2 **2q**

2q was prepared according to **GP3** using diazonium tetrafluoroborate **SI-2q-2** (620 mg, 2.50 mmol, 1.00 equiv) and Li-TIPS-acetylene (600 mM in THF–hexane, 4.20 mL, 1.00 equiv) in anhydrous THF (8.0 mL, 0.2 M in total) at – 78 °C. The reaction was warmed to room temperature over the course of 1 h and after stirring for another hour at room temperature was poured into saturated aqueous $NaHCO_3$ solution (30 mL). The resulting mixture was extracted with Et_2O (3x50 mL), the combined organic layers were dried over Na_2SO_4 and concentrated under reduced pressure. Purification of the residue by flash chromatography (3% EtOAc in hexanes to 5% EtOAc in hexanes) yielded the product as dark red oil (392 mg, 1.15 mmol, 46%).

^{1}H NMR (400 MHz, CDCl3) δ /ppm = 7.84 (d, *J* = 9.1 Hz, 2H), 7.06 (d, *J* = 9.1 Hz, 2H), 4.78 (d, *J* = 2.4 Hz, 2H), 2.58 (s, 1H), 1.26 – 1.06 (m, 21H).

^{13}C NMR (101 MHz, CDCl3) δ / ppm = 161.7, 148.9, 124.8, 115.4, 112.5, 110.0, 77.8, 76.4, 56.2, 18.8, 11.5.

IR (film) 3298, 3075, 2943, 2890, 2865, 2726, 2622, 2275, 2124, 2092, 2007, 1902, 1594, 1495, 1461, 1377, 1314, 1299, 1264, 1245, 1221, 1174, 1146, 1111, 1070, 1020, 996, 926, 881, 837, 805, 780, 754, 723, 665 (cm^{-1}).

HRMS (ESI) calculated for $C_{20}H_{29}N_2OSi$ [M+H$^+$]: 341.2044, found 341.2041.

Preparation of Azotriazole Conjugates 7a–c

Biotin-Androstanolone Azotriazole Conjugate 7a – (3aS,4S,6aR)-4-(4-(4-((4-((E)-(1-((5S,8R,9S,10S,13S,14S,17R)-10,13-dimethyl-3-oxohexadecahydro-1H-cyclopenta[a]phenanthren-17-yl)-1H-1,2,3-triazol-4-yl)diazenyl)phenoxy)methyl)-1H-1,2,3-triazol-1-yl)butyl)tetrahydro-1H-thieno[3,4-d]imidazol-2(3H)-one

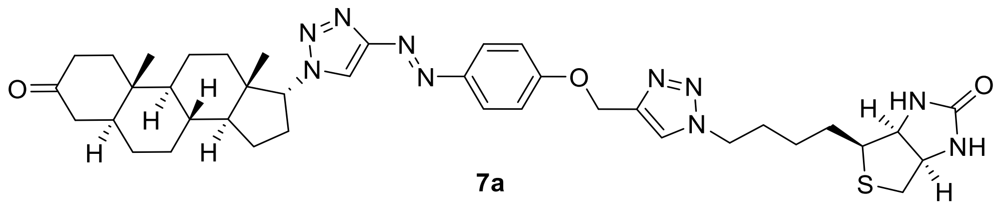

7a

Triethylamine (15 µL, 0.10 mmol, 2.0 equiv) was added to a stirred solution of diacetylene **2q** (17 mg, 0.050 mmol, 1.0 equiv), biotin-derived azide **6a** (13 mg, 0.050 mmol, 1.0 equiv) and CuI (1.0 mg, 5.0 µmol, 10 mol%) in THF (0.35 mL). The resulting deep red mixture was stirred at ambient temperature under exclusion of light. After 2 h, both starting materials were completely consumed as judged by TLC (hexane:EtOAc = 4:1). At this point, azide **6b** (17 mg, 54 µmmol, 1.1 equiv), TBAB (16 mg, 0.050 mmol, 1.0 equiv), water (0.1 mL) and a solution of CsF (1.5 M in water, 36 µL, 54 µmol, 1.1 equiv) were added. The resulting mixture was stirred at ambient temperature for 2 h, before it was heated to 40 °C. After 26 h at 40 °C, the reaction mixture was cooled to ambient temperature and was diluted with a mixture of $CHCl_3$ and MeOH (9:1, 5 mL). The resulting mixture was filtered through a plug of Na_2SO_4 (~1 cm) and celite (~1 cm) and the residue was rinsed with the same mixture (3x3 mL). The brown filtrate was concentrated under reduced pressure. Purification of the residue by flash chromatography (10% MeOH in $CHCl_3$) yielded the product as intense yellow oil that solidified upon storage in the freezer (26 mg, 34 µmmol, 69%).

^{1}H NMR (400 MHz, $CDCl_3$) δ / ppm = 7.91 (d, *J* = 8.6 Hz, 2H), 7.83 (s, 1H), 7.69 (s, 1H), 7.08 (d, *J* = 8.7 Hz, 2H), 6.20 (s, 1H), 5.43 (s, 1H), 5.27 (s, 2H), 4.67 (dd, *J* = 8.5, 1.9 Hz, 1H), 4.46 (dd, *J* = 7.9, 4.8 Hz, 1H), 4.36 (t, *J* = 7.1 Hz, 2H), 4.26 (dd, *J* = 7.9, 4.6 Hz, 1H), 3.10 (dd, *J* = 8.2, 4.9 Hz, 1H), 2.86 (dd, *J* = 12.8, 4.8 Hz, 1H), 2.69 (d, *J* = 12.8 Hz, 1H), 2.62 – 2.49 (m, 1H), 2.41 – 2.18 (m, 4H), 2.15 – 2.03 (m, 2H), 2.00 – 1.87 (m, 4H), 1.79 (dd, *J* = 13.1, 3.3 Hz, 1H), 1.69 – 1.55 (m, 4H), 1.55 – 1.29 (m, 12H), 0.98 (ap. s, 6H), 0.72 – 0.54 (m, 1H), 0.30 (td, *J* = 13.3, 12.7, 4.3 Hz, 1H).

^{13}C NMR (101 MHz, $CDCl_3$) δ / ppm = 211.9, 163.9, 161.2, 160.1, 147.3, 143.5, 125.1, 123.2, 115.3, 114.6, 71.0, 62.4, 62.1, 60.2, 55.7, 50.5, 49.9, 46.6, 46.5, 44.7, 40.7, 38.4, 38.1, 35.8, 35.7, 32.7, 31.9, 30.0, 28.9, 28.7, 28.5, 28.5, 26.5, 25.3, 20.8, 18.8, 13.9, 11.5.

IR (film): 3235, 3136, 2934, 2856, 2397, 1698, 1598, 1583, 1498, 1447, 1386, 1333, 1313, 1298, 1247, 1221, 1181, 1146, 1108, 1040, 1000, 947, 912, 840, 812, 760, 733, 700, 637, 533, 504, 448, 410 (cm^{-1}).

HRMS (ESI) calculated for $C_{40}H_{55}N_{10}O_3S$ [$M+H^+$]: 755.4174, found: 755.4170.

[α]$_D$ $^{25°}$= +27.2 (c = 0.35, $CHCl_3$).

Preparation of 7a and 7b

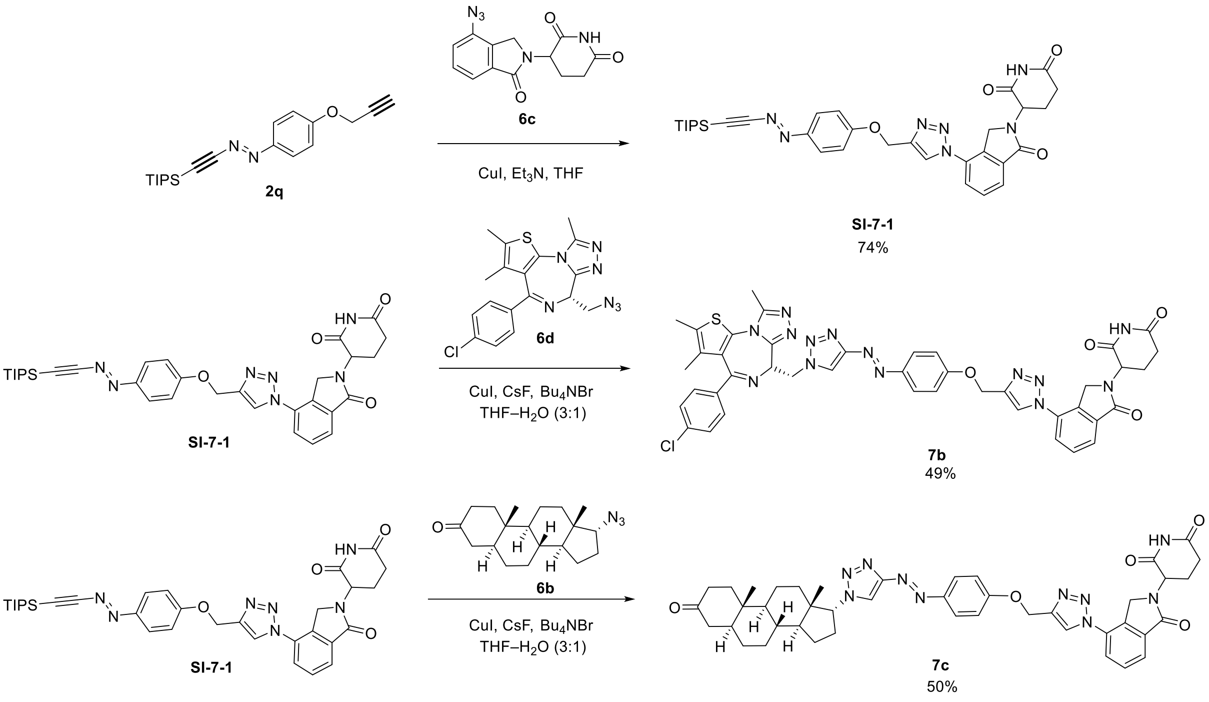

Lenalidomide azoacetylene intermediate (SI-7-1) – (*E*)-3-(1-oxo-4-(4-((4-(((triisopropylsilyl)ethynyl)diazenyl)phenoxy)methyl)-1*H*-1,2,3-triazol-1-yl)isoindolin-2-yl)piperidine-2,6-dione

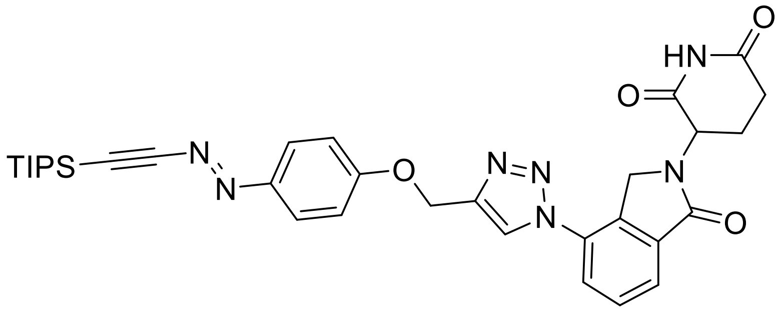

SI-7-1

Azoacetylene **2q** (34 mg, 0.10 mmol), CuI (1.9 mg, 0.010 mmol), azido-lenalidomide **6c** (25 mg, 0.10 mmol) were dissolved in THF (1.0 mL) and NEt_3 (0.030 mL, 0.20 mmol) was added dropwise. After stirring at 40 °C for 12 h, the solvent was removed in vacuo and the crude product was purified via column chromatography (DCM:Methanol = 10:0.4, R_f = 0.25) to give **SI-7-1** (46 mg, 74 µmol, 74%, *E*:*Z* = 10:1) as a red oil.

^{1}H-NMR (400 MHz, $CDCl_3$): δ / ppm = 8.53 (s, 1H), 8.21 (s, 1H), 7.95 (dd, *J* = 7.2, 1.3 Hz, 1H), 7.86 – 7.80 (m, 2H), 7.71 – 7.61 (m, 2H), 7.13 – 7.06 (m, 2H), 5.38 (s, 2H), 5.31 – 5.22 (m, 1H), 4.80 (s, 2H), 2.97 – 2.77 (m, 2H), 2.44 (qd, *J* = 13.0, 5.3 Hz, 1H), 2.22 (dtd, *J* = 12.8, 5.1, 2.5 Hz, 1H), 1.24 – 1.11 (m, 21H).

Note: $CDCl_3$ contained TMS as internal reference (Peak at 0.00 ppm).

^{13}C-NMR (101 MHz, $CDCl_3$): δ / ppm = 171.3, 169.6, 167.9, 162.1, 148.8, 144.3, 134.8, 133.6, 132.1, 129.9, 124.9, 124.6, 122.5, 121.5, 115.3, 112.7, 110.0, 62.1, 52.1, 48.3, 31.6, 23.4, 18.8, 11.5.

IR (Diamond-ATR, neat): $\tilde{\nu}$ / cm^{-1} = 3250, 2960, 2900, 1710, 1550, 1250, 1150, 750, 700.

HRMS (ESI) calcd. for $C_{33}H_{39}N_7NaO_4Si$ $[M+Na]^+$: 648.2725, found 648.2719.

Lenalidomide-JQ1 Azotriazole BRD photoPROTAC candidate 7b – 3-(4-(4-((4-((E)-(1-(((S)-4-(4-chlorophenyl)-2,3,9-trimethyl-6H-thieno[3,2-f][1,2,4]triazolo[4,3-a][1,4]diazepin-6-yl)methyl)-1H-1,2,3-triazol-4-yl)diazenyl)phenoxy)methyl)-1H-1,2,3-triazol-1-yl)-1-oxoisoindolin-2-yl)piperidine-2,6-dione

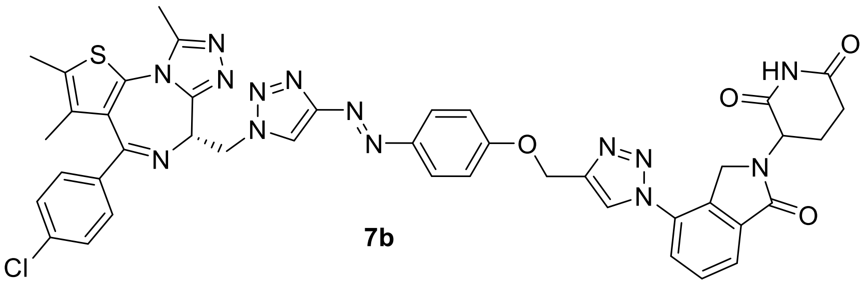

7b

Azotriazole PROTAC candidate **7b** was prepared via **GP5**, using azoacetylene **SI-7-1** (13 mg, 0.020 mmol), CuI (0.40 mg, 2.0 µmol), TBAB (6.8 mg, 21 µmol), **azido-JQ1 6d** (8.5 mg, 21 µmol), and CsF (aq. 1.5 M, 13 µL, 0.020 mmol) and the reaction was stirred for 12 h. After work-up, the crude product was purified via column chromatography (DCM:MeOH = 10:0.3 and Chloroform:MeOH = 10:0.3, R_f = 0.2) to give **7b** (8.5 mg, 9.8 µmol, 49%, *E*:*Z* = 13:1) as a yellow oil.

^{1}H NMR (500 MHz, $CDCl_3$) δ / ppm = 8.48 (s, 1H), 8.20 (s, 1H), 8.08 (d, *J* = 10.2 Hz, 1H), 7.99 (t, *J* = 7.6 Hz, 3H), 7.78 – 7.62 (m, 2H), 7.34 (t, *J* = 7.3 Hz, 4H), 7.15 (d, *J* = 8.5 Hz, 2H), 5.66 (dd, *J* = 13.8, 6.8 Hz, 1H), 5.48 – 5.44 (m, 1H), 5.42 (s, 2H), 5.24 (dd, *J* = 13.2, 4.9 Hz, 1H), 4.84 (s, 2H), 4.74–4.67 (m, 1H), 2.96 (d, *J* = 17.5 Hz, 1H), 2.90 – 2.77 (m, 1H), 2.68 (s, 3H), 2.54 – 2.43 (m, 1H), 2.40 (s, 3H), 2.26 (d, *J* = 11.3 Hz, 1H), 1.65 (s, 3H).

^{13}C NMR (126 MHz, $CDCl_3$) δ / ppm = 170.9, 169.2, 168.0, 165.2, 161.0, 160.3, 153.8, 150.4, 147.6, 144.7, 137.4, 136.3, 134.9, 133.7, 132.3, 132.3, 131.5, 131.1, 130.3, 130.0, 129.0, 125.3, 124.6, 122.7, 121.4, 118.4, 115.3, 62.2, 57.1, 52.2, 52.0, 48.3, 31.7, 29.8, 23.5, 14.6, 13.3, 11.9.

IR (film) 2960, 2926, 2873, 1698, 1598, 1565, 1529, 1499, 1464, 1419, 1377, 1334, 1316, 1298, 1241, 1202, 1147, 1107, 1090, 1031, 1014, 918, 841, 808, 753, 731, 638, 607, 569, 536, 485, 469, 444, 404 (cm^{-1}).

HRMS (ESI) calculated for $C_{42}H_{36}ClN_{14}O_4S$ [$M+H^+$]: 867.2448, found: 867.2436.

[α]$_D$ $^{25°}$= –27.3 (c = 0.28, $CHCl_3$).

Lenalidomide-Androstanolone Azotriazole photoPROTAC candidate 7c – 3-(4-(4-((4-((E)-(1-((5S,8R,9S,10S,13S,14S,17R)-10,13-dimethyl-3-oxohexadecahydro-1H-cyclopenta[a]phenanthren-17-yl)-1H-1,2,3-triazol-4-yl)diazenyl)phenoxy)methyl)-1H-1,2,3-triazol-1-yl)-1-oxoisoindolin-2-yl)piperidine-2,6-dione

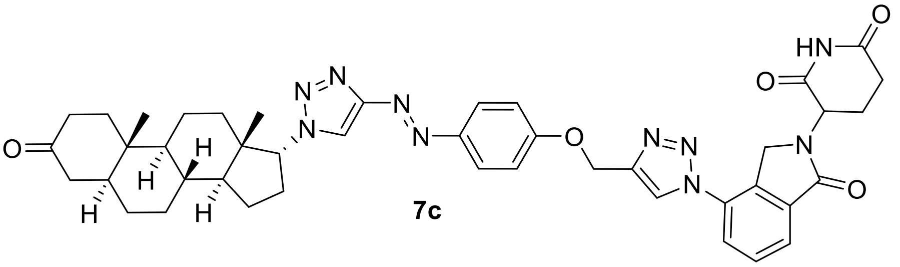

7c

Azotriazole PROTAC candidate **7c** was prepared via **GP5**, using azoacetylene **SI-7-1** (13 mg, 0.020 mmol), CuI (0.40 mg, 2.0 µmol), TBAB (6.8 mg, 21 µmol), **azido-stanolone 6b** (6.3 mg, 0.020 mmol), and CsF (aq. 1.5 M, 13 µL, 0.020 mmol) and the reaction was stirred for 12 h. After work-up, the crude product was purified via column chromatography (DCM:MeOH = 10:0.3, R_f = 0.2 and Chloroform:MeOH = 10:0.5, R_f = 0.2) to give **7c** (7.8 mg, 9.9 µmol, 50%, *E*:*Z* = 4:1) as a yellow oil.

^{1}H-NMR (400 MHz, CDCl$_3$): δ / ppm = 8.19 (d, *J* = 13.2 Hz, 2H), 8.01 – 7.93 (m, 3H), 7.83 (s, 1H), 7.73 – 7.67 (m, 2H), 7.16 – 7.11 (m, 2H), 5.40 (s, 2H), 5.32 – 5.21 (m, 1H), 4.84 (d, *J* = 5.7 Hz, 2H), 4.72 – 4.65 (m, 1H), 2.96 – 2.79 (m, 2H), 2.62 – 2.40 (m, 2H), 2.39 – 2.16 (m, 5H), 2.13 – 1.99 (m, 2H), 1.94 – 1.77 (m, 2H), 1.48 (tdd, *J* = 12.4, 7.2, 3.7 Hz, 4H), 1.42 – 1.16 (m, 6H), 1.08 (td, *J* = 12.0, 4.8 Hz, 1H), 0.99 (d, *J* = 1.9 Hz, 6H), 0.69 – 0.59 (m, 1H), 0.37 – 0.26 (m, 1H).

Note: Peaks of the main diastereomer are given.

^{13}C-NMR (126 MHz, CDCl$_3$): δ / ppm = [211.9, 211.8], [171.0, 170.9], [169.28, 169.27], 168.0, [160.94, 160.93], [160.10, 160.09], 147.6, 144.6, 134.9, 133.7, 132.2, 130.0, 125.1, 124.7, 122.6, 121.4, 115.3, [114.60, 114.57], 71.1, 62.2, 53.1, 52.2, [50.98, 50.96], 48.3, 46.61, 46.55, 44.7, 38.5, 38.2, 35.9, 35.8, 32.7, 32.0, 31.7, 28.9, 28.8, 25.3, 23.5, 20.9, 18.8, 10.6.

Note: Peaks of the main diastereomer (regarding E/Z) are given. Because Lenalidomide was employed as a racemate, a 1:1 mixture of diastereomers is obtained. In some cases this leads to the observation of ^{13}C peak pairs in a 1:1 ratio. These peaks are reported in pairs in the following format [Peak 1, Peak 2].

IR (Diamond-ATR, neat): $\tilde{\nu}$ / cm^{-1} = 3105, 2923, 285, 1687, 1598, 1372, 1298, 1239, 1017, 727, 647.

HRMS (ESI) calcd. for $C_{43}H_{48}N_{10}NaO_5$ [M+Na]$^+$**:** 807.3701, found 807.3697..

[a]$_D^{25}$ = –6.2 (c = 0.333, CHCl$_3$).

Photophysical Characterization and Procedures

Photophysical Procedure 1 (PP1): Determination of Photostationary State Compositions.

A stock solution of the respective azo compound was diluted in a 2 mL brown glass GC vial with anhydrous DMSO to give a 100 µM solution. The GC vial was placed in a custom-made dark chamber and the solution irradiated at 340 nm, 365 nm, 415 nm, 455 nm, 505 nm, and 530 nm, respectively. This was done by going from small to high wavelengths to avoid interference due to low absorption at high wavelengths. After 20 min of irradiation (30 min for irradiation at 340 nm), the photostationary state was reached and the GC-vial was properly sealed with a septum cap and placed on a HPLC machine (Waters e2965 separation module equipped with a 2998 PDA detector) for separation of the respective E- and Z-isomer. 10 µL of the solution were injected onto a ReprosilChiral-NR column and the isomers eluted with MeCN/H_2O (4:6) containing 0.1% formic acid.

The UV/Vis spectra, recorded by the PDA detector during the HPLC run, were extracted for each isomer and compound at the respective time of elution. Addition of the extracted isomeric spectra afforded the combined spectrum of the isomeric mixture in the eluent mixture (this is necessary considering potential solvatochromic effects affecting the position of the isosbestic wavelength). Plotting of the spectra using an in-house written MATLAB script allowed determination of the isosbestic wavelength. To quantify the E/Z ratio at each individual PSS, the chromatograms at the isosbestic wavelength were extracted and integrated. The generated data is compiled in **Tables T4 and 5** (see end of SI for HPLC chromatograms and extracted PDA spectra).

After the HPLC injection, the sample was taken from the machine, the solution transferred to a quartz cuvette and the UV-Vis absorbance spectrum recorded on a Jasco V-630 spectrometer to obtain UV-Vis spectra in DMSO (100 µM).

The sample was transferred back into the GC vial, irradiated at the next wavelength in ascending order, and processed as described above.

Photophysical Procedure 2 (PP2): Calculation of pure *E* and *Z*-spectra from known UV-Vis compositions at photostationary states

UV-Vis-absorption spectra of the pure *E*- and *Z*-isomer were calculated from the most *E*-enriched PSS and *Z*-enriched PSS by solving the following system of linear equations, resulting from Beer's law.

$$A_{PSS1}(\lambda) = x_{E1} \cdot A_E(\lambda) + x_{Z1} \cdot A_Z(\lambda)$$

$$A_{PSS2}(\lambda) = x_{E2} \cdot A_E(\lambda) + x_{Z2} \cdot A_Z(\lambda)$$

To this end, the linsolve function in MATLAB R2020b was applied, taking as input the absorption spectra of the *E*-enriched PSS (A_{PSS1}) and *Z*-enriched PSS (A_{PSS2}), as well as the respective molar fraction of the E-isomer (x_E) and Z-isomer (x_Z) at the given PSS (as determined by **PP1**). This allowed extraction of absorption maxima of pure *E* and *Z*-isomers which are reported in **Tables T4 and T5**.

Photophysical Procedure 3 (PP3): Determination of Thermal *Z*-Half-Lives.

A stock solution of the respective azo compound was diluted in a 20 mL brown glass screw cap vial with the respective amount of dry DMSO to give a 50 µM solution. 1-4 mL of the stirred solution were irradiated at 340 nm (for **4a**) or 365 nm (for all other compounds investigated).

After 20 min, 1 mL of the irradiated solution was transferred to a quartz cuvette and the cuvette was placed on a Perkin Elmer Lambda UV-Vis machine, equipped with a Peltier element for temperature control. The solution was allowed to warm to the temperature controlled by the Peltier element for 5-10 min after which monitoring of the absorbance at 350 nm was commenced (the observation wavelength was chosen as a compromise for the sake of measuring multiple samples at the same time). At the end of the measurement, the internal temperature of the cuvette was checked with a thermometer (Omega HH81A).

In some cases, the acquisition was stopped after the curves plateaued. The thermal relaxation of the respective *Z*-enriched state was plotted using an in-house written MATLAB script and the curves were fitted with an exponential function to determine the rate constant k, assuming a first-order rate law.

$$[Z]_t = Ae^{-kt}$$

$[Z]_t$ – concentration of the Z-isomer, k – rate constant of the thermal decay, t- time, A – scaling factor

In case the thermal relaxation was recorded at 25.0 °C, the thermal half-life was calculated as

$$t_{1/2} = \frac{ln2}{k}.$$

$t_{1/2}$ – thermal half-live

For compounds with thermal half-lives in the range of weeks and more at 25 °C, the thermal half-life at 25 °C was calculated by extrapolation using Eyring's equation.

$$\ln\frac{k}{T} = \frac{-\Delta H^{\ddagger}}{R} \cdot \frac{1}{T} + \ln\frac{\kappa k_B}{h} + \frac{\Delta S^{\ddagger}}{R}$$

$\Delta H^{\ddagger}$ - enthalpy of activation, $\Delta S^{\ddagger}$ - entropy of activation, R – ideal gas constant, κ – transmission coefficient, k_B – Boltzmann's constant, h – Planck's constant, T – temperature

in the form

$$\ln\frac{k}{T} = C_1 \cdot \frac{1}{T} + C_2$$

From plotting the respective rate constants k against the temperature of the measurement and subsequent linear regression using MATLAB R2020b, the values of C1 and C2 were obtained and used to calculate the thermal half-life at 25.0 °C.

$$k = T \cdot exp\left(C_1 \cdot \frac{1}{T} + C_2\right)$$

$$t_{1/2} = \frac{ln2}{k}$$

Photophysical Procedure 4 (PP4): Demonstration of Photostability.

A stock solution of **4a** or **4b** was diluted in a quartz cuvette to give 0.75 mL of a 100 µM solution. The cuvette was placed in a dark chamber and the solution irradiated at 365 nm for 20 min. The UV-Vis absorbance at 329 nm (for **4a**) or 351 nm (for **4b**) was determined using a Jasco V-630 spectrometer. Afterwards, the solution was irradiated at 415 nm (for **4a**) or 455 nm (for **4b**) for 8 min and the absorbance recorded as described. The irradiation wavelength was alternated multiple times to proof the absence of photobleaching by showing that the system returns to the same PSS after each run. For **4b** the PSS was reached after irradiation at 365 nm for 11 min, which was then applied after the first cycle.

For compounds **2a–b** and **2i** the procedure was adapted as specified in the caption of **Figures S81**, **S82** and **S83**.

Overview of Photophysical Properties of Selected Azoacetylenes (Table T4)

compound	Thermal Half Lives at 25 °C	Maxima of absorption[a]				Isosbestic wavelength (nm)		PSS (340 nm)[a]		PSS (365 nm)[a]		PSS (415 nm)[a]		PSS (455 nm)[a]		PSS (505 nm)[a]		PSS (530 nm)[a]	
#	$t_{1/2}$	(*Z*) ππ* $λ_{MAX}$(nm)	(*Z*) nπ* $λ_{MAX}$(nm)	(*E*) ππ* $λ_{MAX}$(nm)	(*E*) nπ* $λ_{MAX}$(nm)	MeCN/H_2O (4:6)	DMSO	Z	E	Z	E	Z	E	Z	E	Z	E	Z	E
**2a**	17 min	308	446	337	465	280	284	14	86	**62**	**38**	**7**	**93**	10	90	16	84	20	80
**2b**	8 min	n.d.[e]	n.d.[e]	380	overlapped[b]	n.d.	n.d.	n.d.[c]	n.d.[c]	n.d.[c]	n.d.[c]	n.d.[c]	n.d.[c]	n.d.[c]	n.d.[c]	n.d.[c]	n.d.[c]	n.d.[c]	n.d.[c]
**2i**	28 min	n.d.[e]	n.d.[e]	325	454	n.d.	n.d.	n.d.[d]	n.d.[d]	n.d.[d]	n.d.[d]	n.d.[d]	n.d.[d]	n.d.[d]	n.d.[d]	n.d.[d]	n.d.[d]	n.d.[d]	n.d.[d]

[a] determined in DMSO

[b] absorption maximum could not be determined due to overlapping of adjacent bands

[c] HPLC assay unsuitable to precisely determine PSS due to short thermal half-life of *Z*-isomer (8 min).

[d] not determined due to significant photobleaching during irradiation.

[e] pure (*Z*)-spectra could not be determined as PSS-compositions are unknown.

Overview of Photophysical Properties of Selected Azotriaoles (Table T5)

compound	Thermal Half Lives at 25 °C	Maximum of absorption[a]				Isosbestic wavelength (nm)		PSS (340 nm)[a]		PSS (365 nm)[a]		PSS (415 nm)[a]		PSS (455 nm)[a]		PSS (505 nm)[a]		PSS (530 nm)[a]	
#	$t_{1/2}$	(*Z*) ππ* λ_{MAX}(nm)	(*Z*) nπ* λ_{MAX}(nm)	(*E*) ππ* λ_{MAX}(nm)	(*E*) nπ* λ_{MAX}(nm)	MeCN/H_2O (4:6)	DMSO	Z	E	Z	E	Z	E	Z	E	Z	E	Z	E
**4a**	254 d	<270[b]	422	329	425	281	282	**99**	**1**	87	13	**29**	**71**	34	66	35	65	34	66
**4b**	20 d	overlapped[c]	439	351	overlapped[c]	298	298	89	11	**96**	**4**	44	56	23	77	10	90	**9**	**91**
**4c**	14 d	336	442	372	overlapped[c]	308	291	69	31	**90**	**10**	69	31	33	67	13	87	**12**	**88**
**4d**	161 d	<270[b]	428	337	423	287	286	79	21	**93**	**7**	29	71	27	73	22	78	**21**	**79**
**4e**	35 d	328	433	358	overlapped[c]	286	283	66	34	**95**	**5**	63	37	35	65	21	79	**18**	**82**
**4f**	196 d	<270[b]	419	321	428	286	284	**97**	**3**	77	23	**18**	**82**	28	72	37	63	36	64
**4g**	335 d	<270[b]	425	337	425	292	288	57	43	**93**	**7**	**23**	**77**	25	75	25	75	24	76
**4i**	n.d.[d]	overlapped[c]	432	328	448	289	281	**84**	**16**	82	18	**40**	**60**	50	50	67	33	68	32
**4j**	n.d.[d]	<270[b]	421	320	440	286	281	**82**	**18**	80	20	**28**	**72**	45	55	64	36	68	32
**4k**	n.d.[d]	275	421	318	431	284	286	**94**	**6**	75	25	**31**	**69**	44	56	60	40	60	40
**4m**	184 d	<270[b]	421	328	425	284	283	69	31	**83**	**17**	**25**	**75**	31	69	31	69	30	70
**4n**	39 h	273	423	328	overlapped[c]	303	293	74	26	**92**	**8**	19	81	18	82	16	84	**15**	**85**
**4o**	11 h	274	overlapped[c]	321	overlapped[c]	296	290	84	16	**88**	**12**	21	79	20	80	18	82	**17**	**83**

[a] determined in DMSO

[b] absorption maximum was not detected due to the cut-off defined by the spectral window of DMSO

[c] absorption maximum could not be determined due to overlapping of adjacent bands

[d] The precise quantitation of the thermal (Z)-half-lives of 2,6-disubstituted derivatives 4i, 4j and 4k was not possible within our experimental setup. The high bistability of these compounds required elevated temperatures to observe relaxation at measurable rates. We presume, however, that at such temperatures slow thermal decomposition occurs in parallel, precluding precise quantitation.

UV-Vis Spectra at Photostationary States after Irradiation at Stated Wavelengths

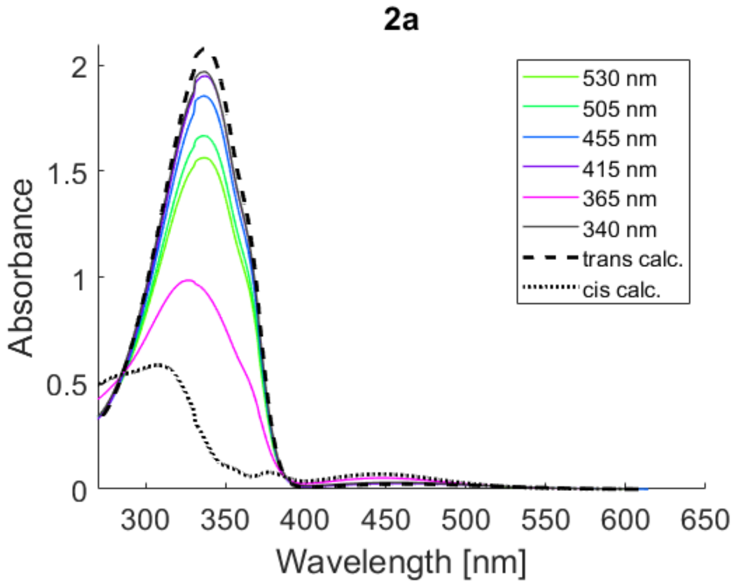

Figure S1: UV-Vis spectra of **2a** in DMSO (100 µM), recorded after irradiation at the stated wavelengths to reach the photostationary state; calculated spectra drawn in dashed lines are estimates due to photobleaching during irradiation.

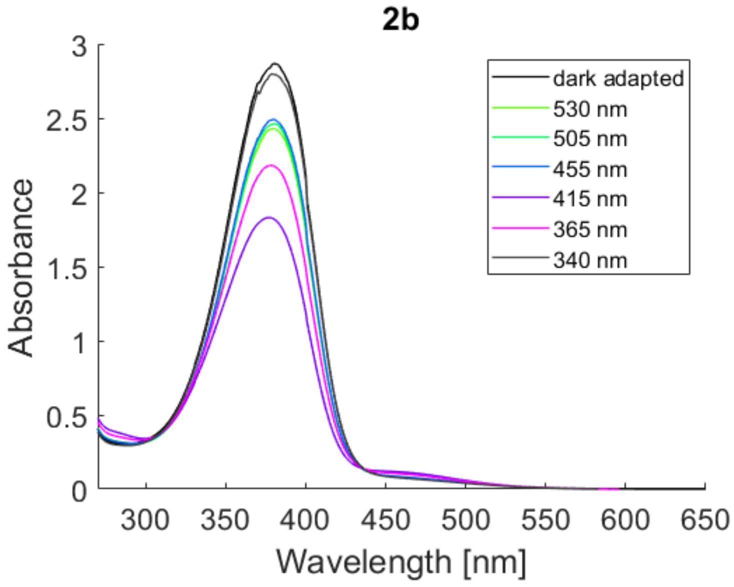

Figure S2: UV-Vis spectra of **2b** in DMSO (100 µM), recorded after irradiation at the stated wavelengths to reach the photostationary state; dark adapted refers to the original solution prior to irradiation.

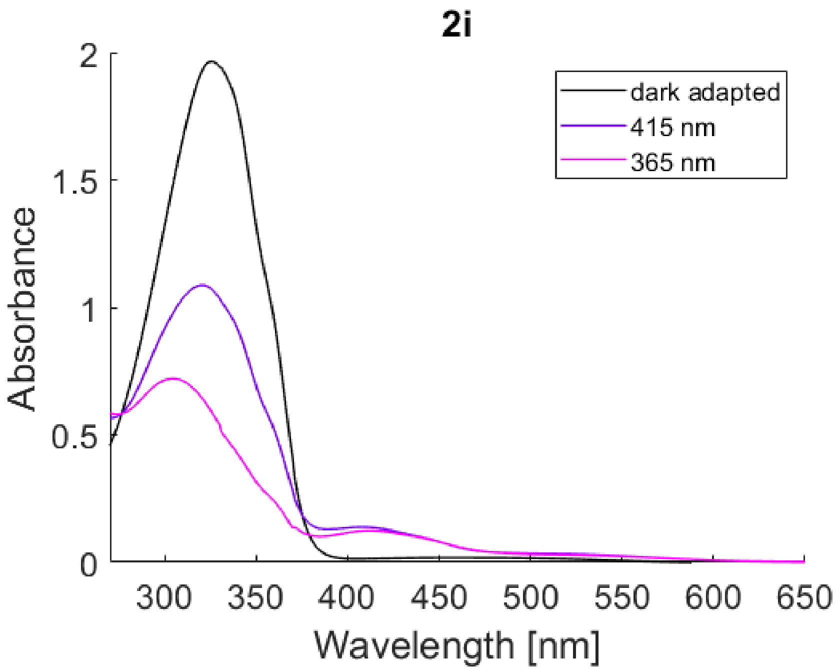

Figure S3: UV-Vis spectra of **2i** in DMSO (100 µM), recorded after irradiation at the stated wavelengths to reach the photostationary state; dark adapted refers to the original solution prior to irradiation. Only two wavelengths shown due to photobleaching during measurement (see below).

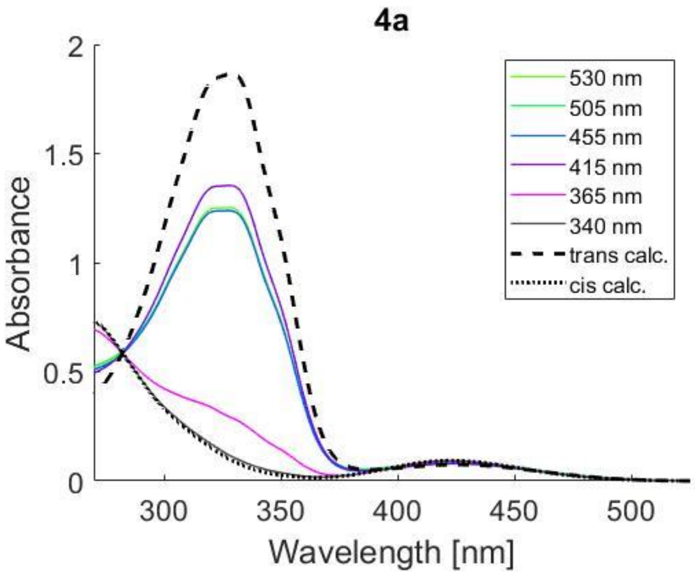

Figure S4: UV-Vis spectra of **4a** in DMSO (100 µM), recorded after irradiation at the stated wavelengths to reach the photostationary state; calculated spectra are drawn in dashed lines.

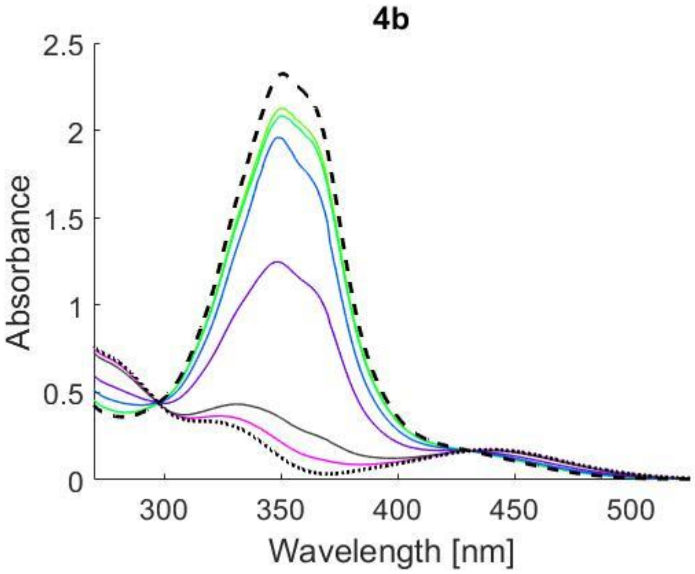

Figure S5: UV-Vis spectra of **4b** in DMSO (100 µM), recorded after irradiation at the stated wavelengths to reach the photostationary state; calculated spectra are drawn in dashed lines.

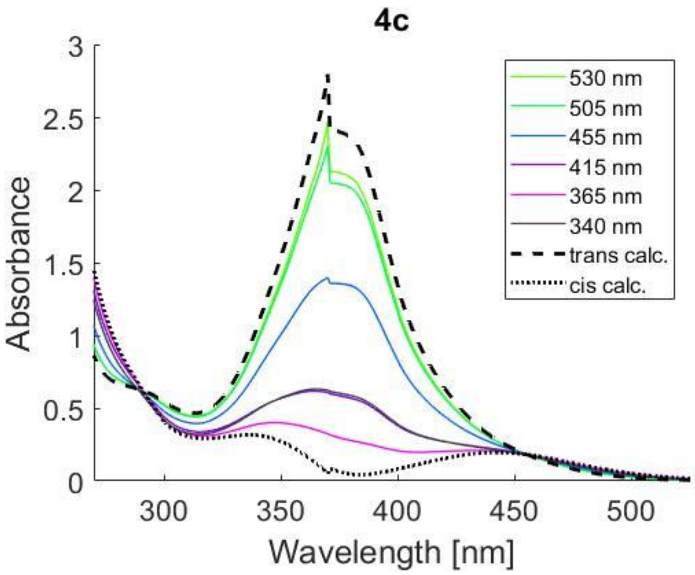

Figure S6: UV-Vis spectra of **4c** in DMSO (100 µM), recorded after irradiation at the stated wavelengths to reach the photostationary state; calculated spectra are drawn in dashed lines. The observed artifact at 370 nm occurred due to a technical issue of the photospectrometer.

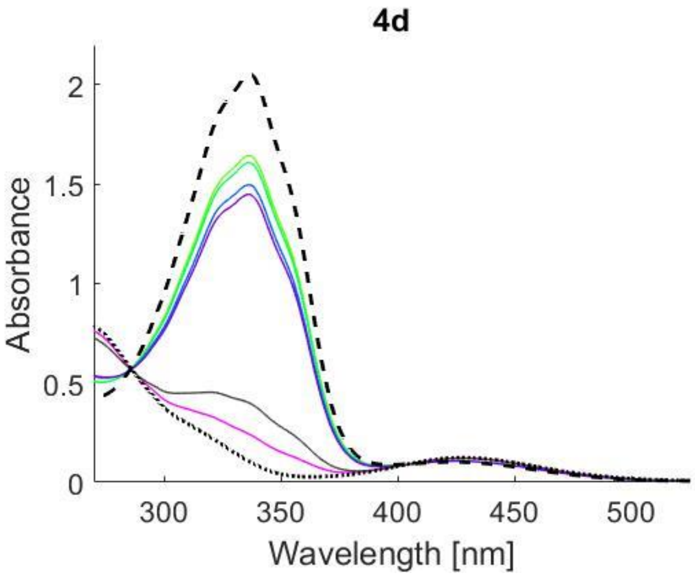

Figure S7: UV-Vis spectra of **4d** in DMSO (100 µM), recorded after irradiation at the stated wavelengths to reach the photostationary state; calculated spectra are drawn in dashed lines.

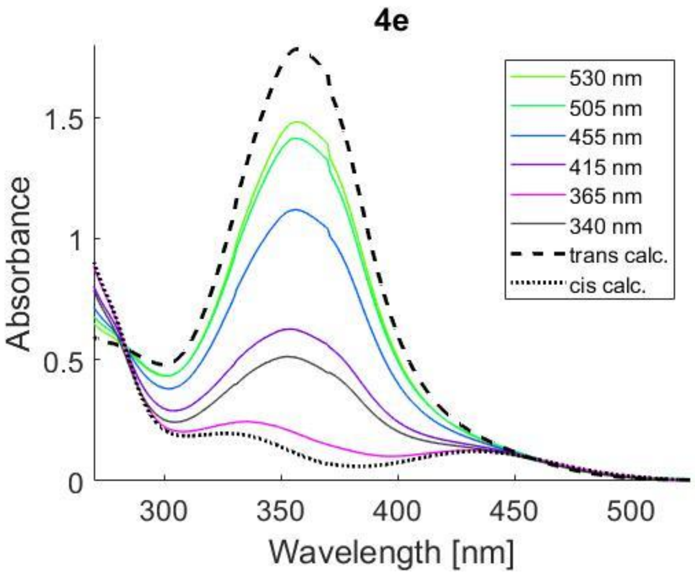

Figure S8: UV-Vis spectra of **4e** in DMSO (100 µM), recorded after irradiation at the stated wavelengths to reach the photostationary state; calculated spectra are drawn in dashed lines.

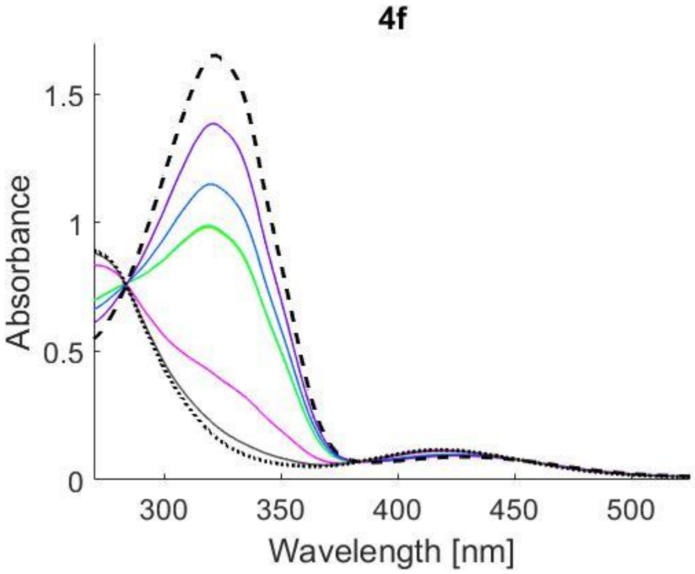

Figure S9: UV-Vis spectra of **4f** in DMSO (100 µM), recorded after irradiation at the stated wavelengths to reach the photostationary state; calculated spectra are drawn in dashed lines.

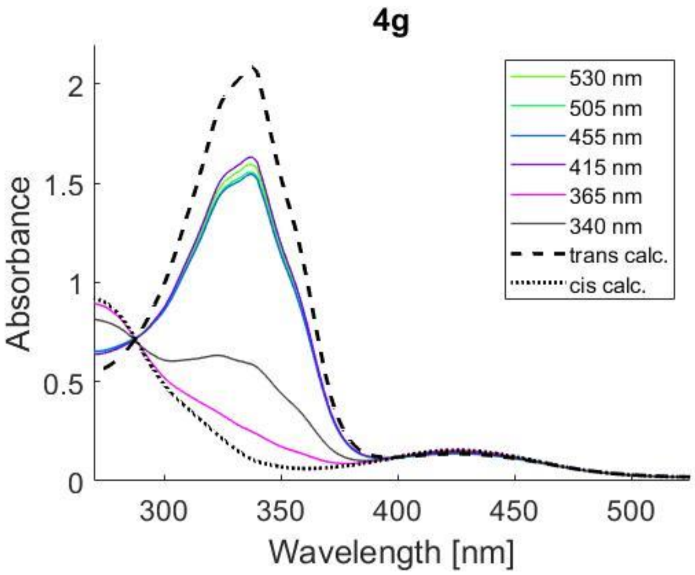

Figure S10: UV-Vis spectra of **4g** in DMSO (100 µM), recorded after irradiation at the stated wavelengths to reach the photostationary state; calculated spectra are drawn in dashed lines.

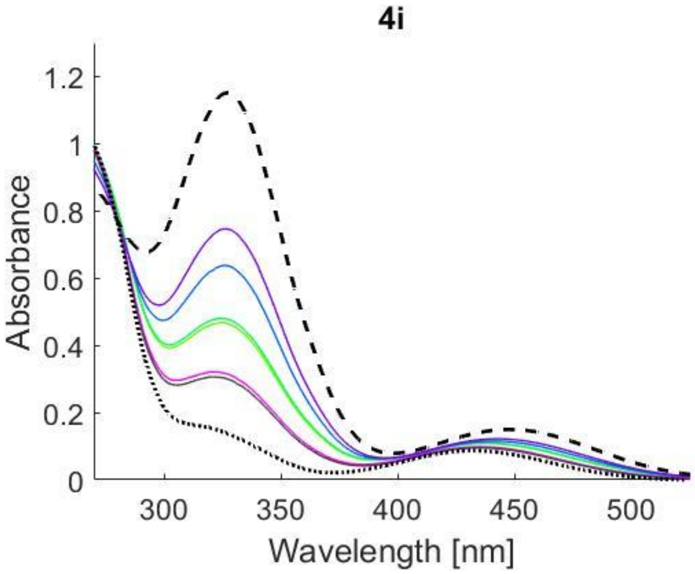

Figure S11: UV-Vis spectra of **4i** in DMSO (100 µM), recorded after irradiation at the stated wavelengths to reach the photostationary state; calculated spectra are drawn in dashed lines.

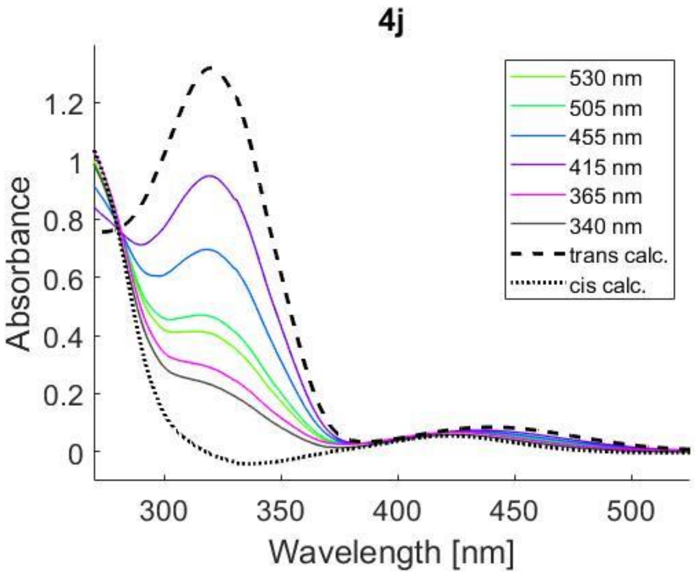

Figure S12: UV-Vis spectra of **4j** in DMSO (100 µM), recorded after irradiation at the stated wavelengths to reach the photostationary state; calculated spectra are drawn in dashed lines.

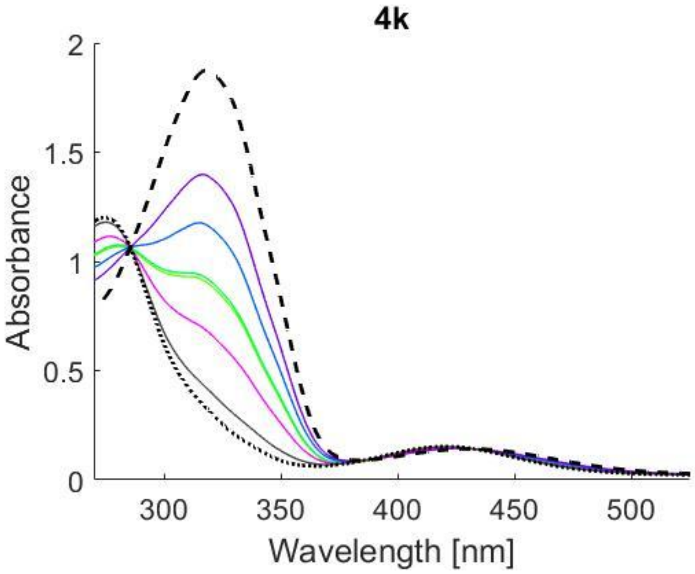

Figure S13: UV-Vis spectra of **4k** in DMSO (100 µM), recorded after irradiation at the stated wavelengths to reach the photostationary state; calculated spectra are drawn in dashed lines.

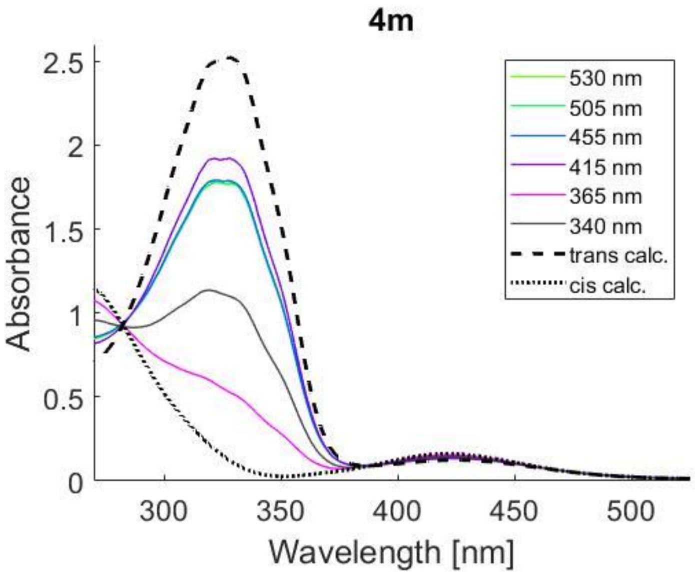

Figure S14: UV-Vis spectra of **4m** in DMSO (150 µM), recorded after irradiation at the stated wavelengths to reach the photostationary state; calculated spectra are drawn in dashed lines.

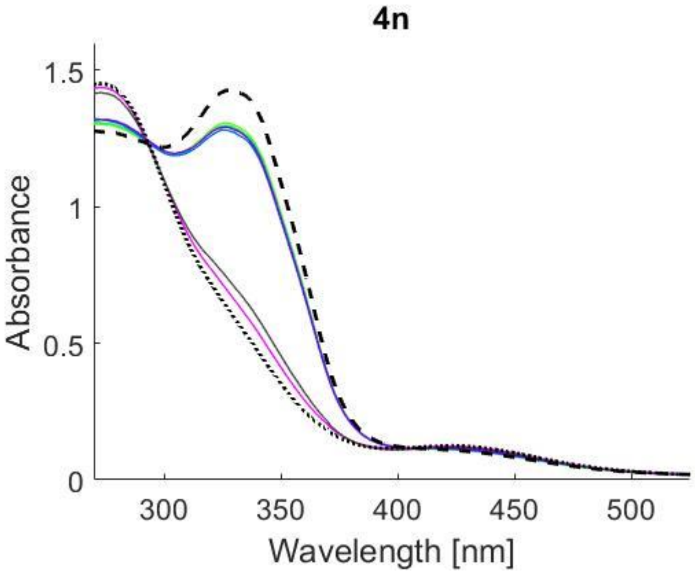

Figure S15: UV-Vis spectra of **4n** in DMSO (100 µM), recorded after irradiation at the stated wavelengths to reach the photostationary state; calculated spectra are drawn in dashed lines.

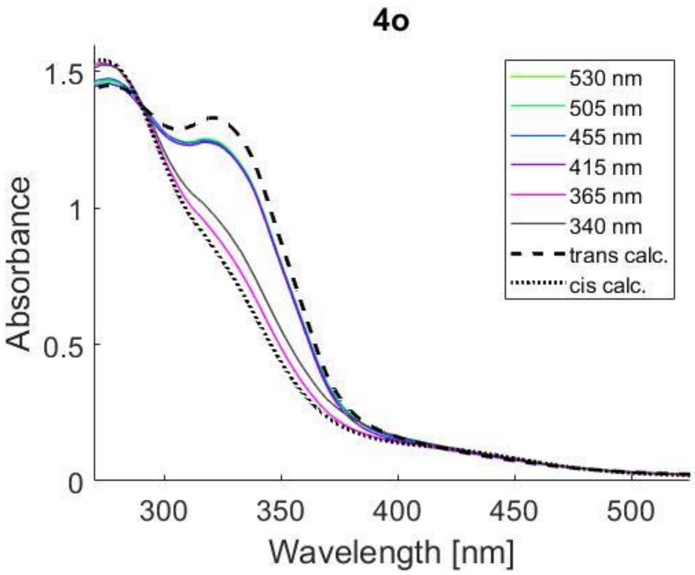

Figure S16: UV-Vis spectra of **4o** in DMSO (100 µM), recorded after irradiation at the stated wavelengths to reach the photostationary state; calculated spectra are drawn in dashed lines.

Bistability Measurements – Thermal Relaxation and Eyring Plots

Thermal Relaxation Plot for 2a

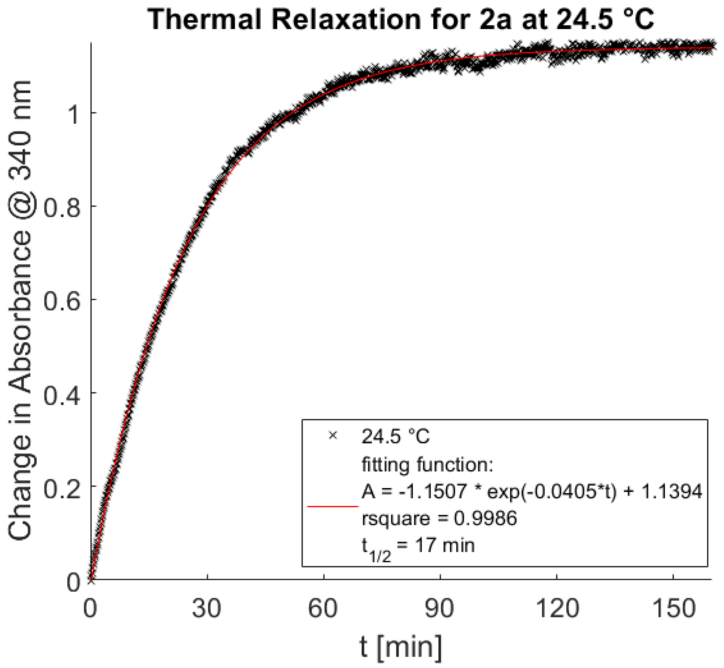

Figure S17: Increase of absorbance at 340 nm and 24.5 °C for a 100 µM solution of **2a** in DMSO after reaching the photostationary state through irradiation at 365 nm measured every 20 s. $t_{1/2}$ is the thermal half-life, calculated from the fitting function assuming a first-order rate law.

Thermal Relaxation Plot for 2b

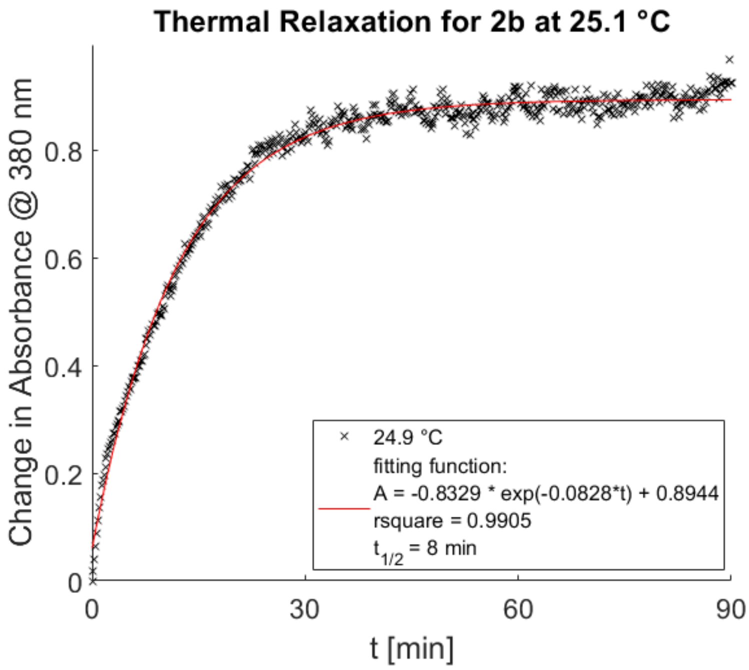

Figure S18: Increase of absorbance at 380 nm and 25.1 °C for a 100 µM solution of **2b** in DMSO after reaching the photostationary state through irradiation at 415 nm measured every 20 s. $t_{1/2}$ is the thermal half-life, calculated from the fitting function assuming a first-order rate law.

Thermal Relaxation Plot for 2i

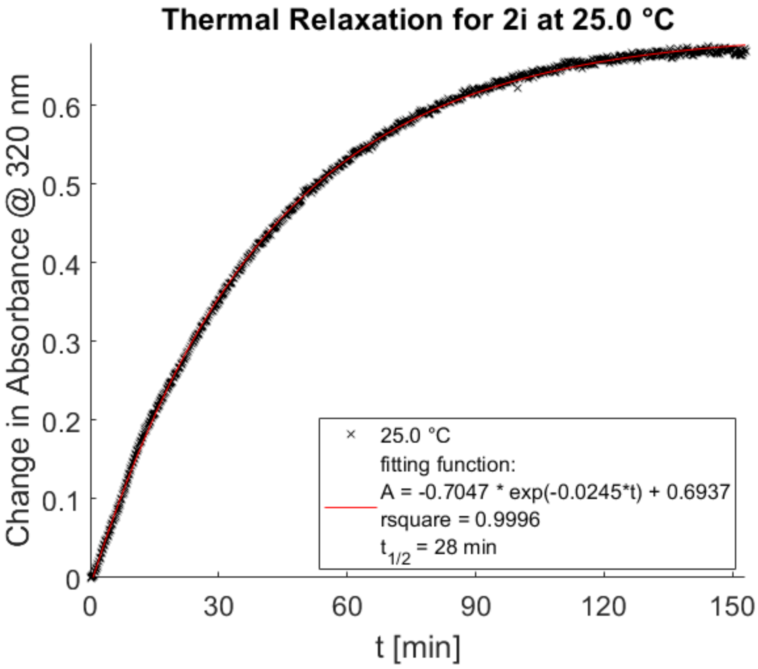

Figure S19: Increase of absorbance at 320 nm and 25.0 °C for a 100 µM solution of **2i** in DMSO after reaching the photostationary state through irradiation at 365 nm measured every 20 s. $t_{1/2}$ is the thermal half-life, calculated from the fitting function assuming a first-order rate law.

Thermal Relaxation Plots for 4a

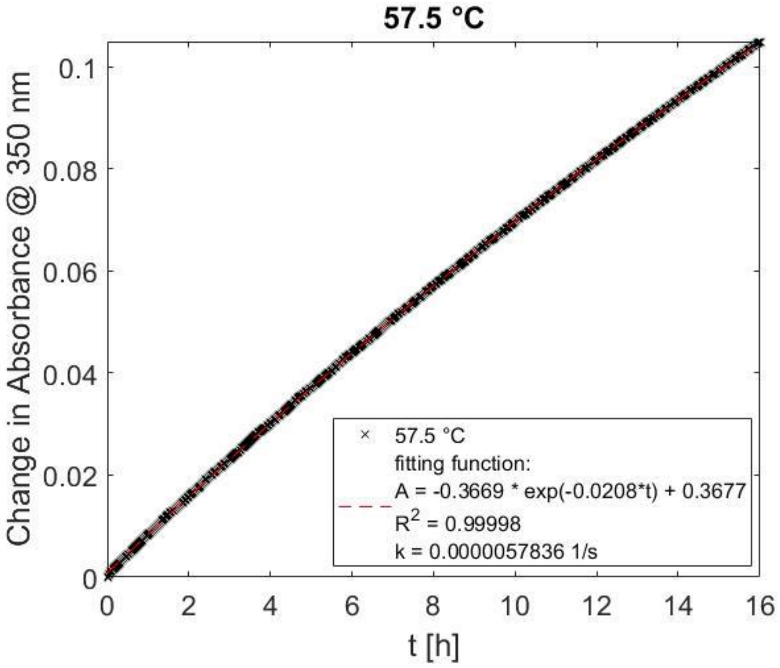

Figure S20: Increase of absorbance at 350 nm and 57.5 °C for a 50 µM solution of **4a** in DMSO after reaching the photostationary state through irradiation at 340 nm measured every 60 s. k is the rate constant of isomerization calculated from the fitting function assuming a first-order rate law.

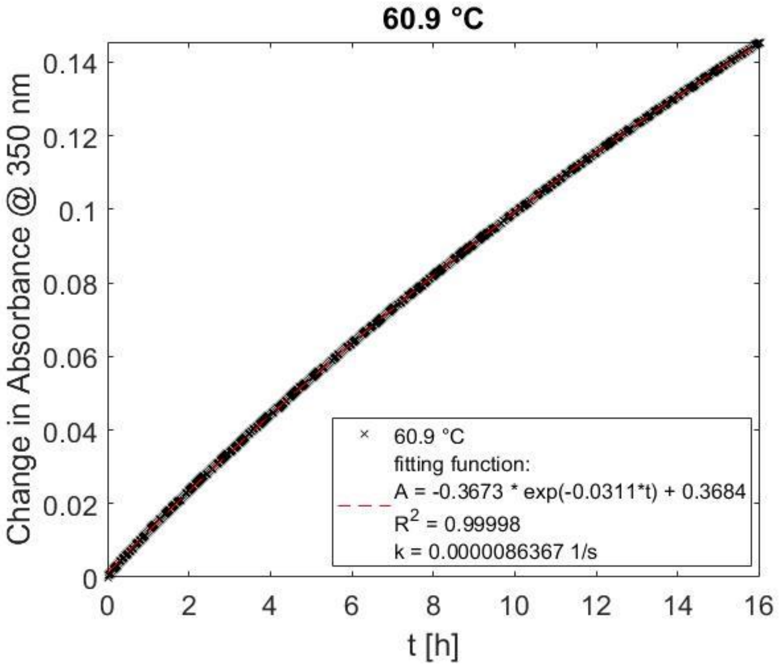

Figure S21: Increase of absorbance at 350 nm and 60.9 °C for a 50 µM solution of **4a** in DMSO after reaching the photostationary state through irradiation at 340 nm measured every 60 s. k is the rate constant of isomerization calculated from the fitting function assuming a first-order rate law.

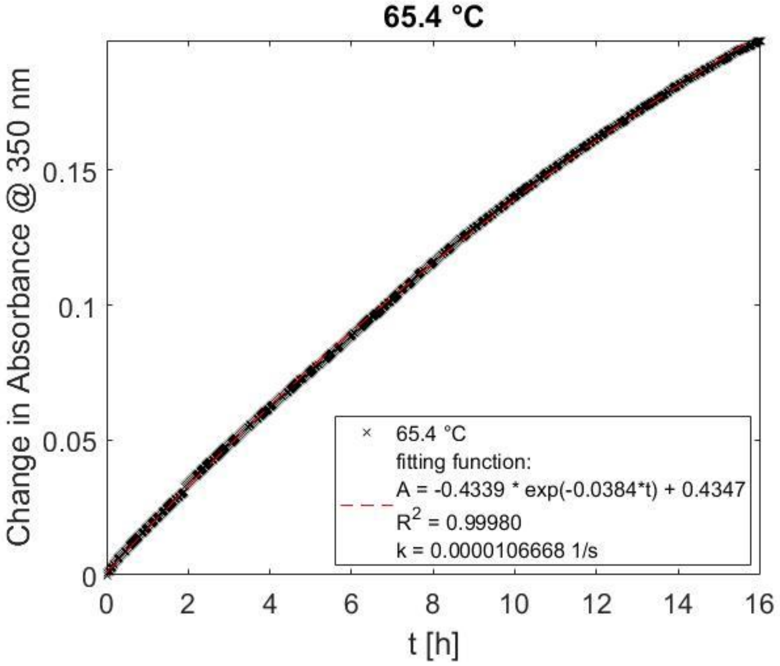

Figure S22: Increase of absorbance at 350 nm and 65.4 °C for a 50 µM solution of **4a** in DMSO after reaching the photostationary state through irradiation at 340 nm measured every 60 s. k is the rate constant of isomerization calculated from the fitting function assuming a first-order rate law.

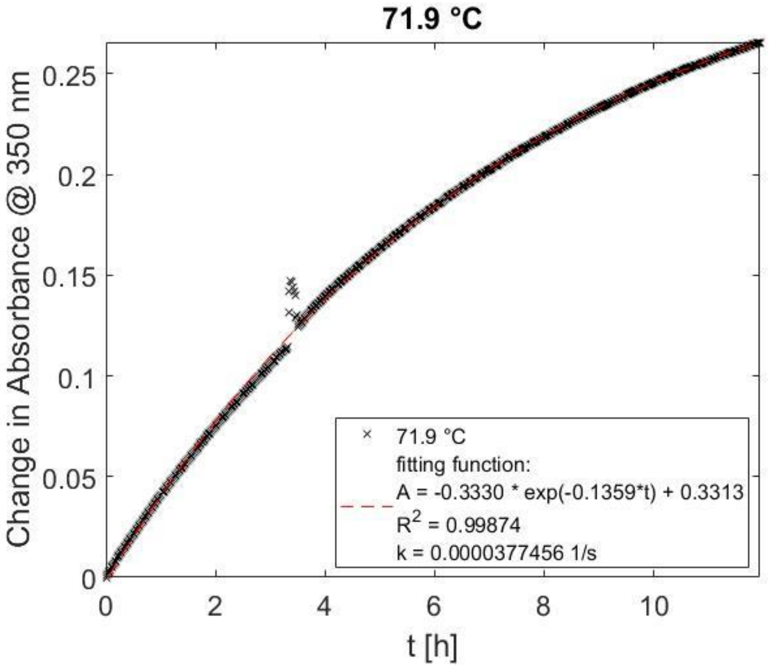

Figure S23: Increase of absorbance at 350 nm and 71.9 °C for a 50 µM solution of **4a** in DMSO after reaching the photostationary state through irradiation at 340 nm measured every 60 s. k is the rate constant of isomerization calculated from the fitting function assuming a first-order rate law.

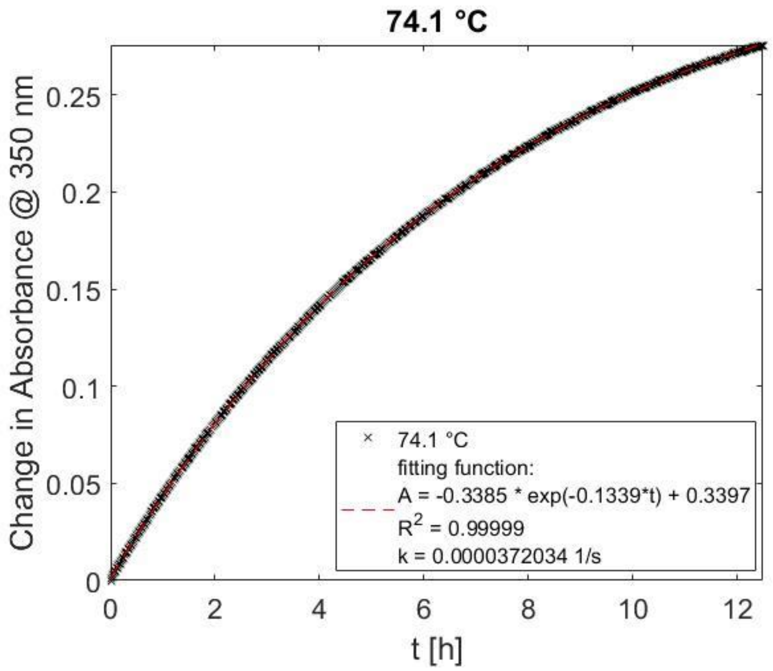

Figure S24: Increase of absorbance at 350 nm and 74.1 °C for a 50 µM solution of **4a** in DMSO after reaching the photostationary state through irradiation at 340 nm measured every 60 s. k is the rate constant of isomerization calculated from the fitting function assuming a first-order rate law.

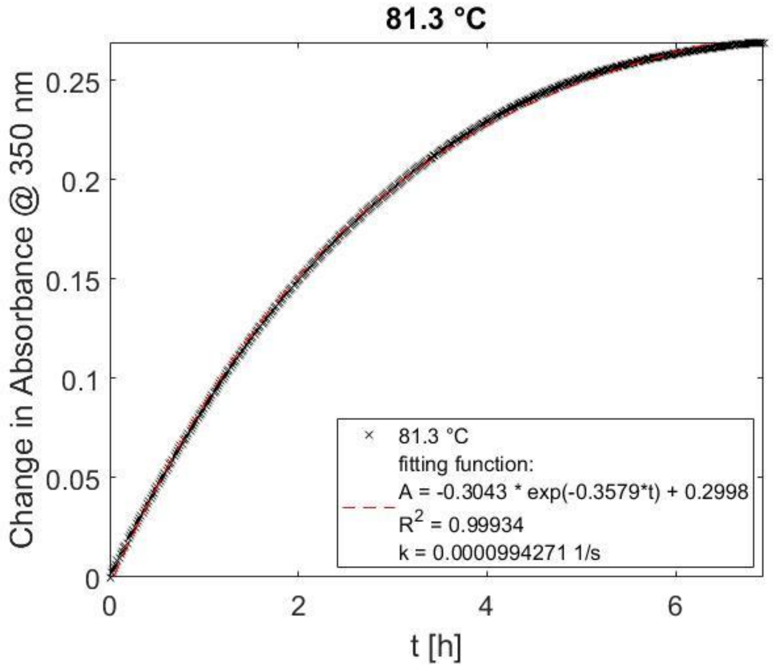

Figure S25: Increase of absorbance at 350 nm and 81.3 °C for a 50 µM solution of **4a** in DMSO after reaching the photostationary state through irradiation at 340 nm measured every 60 s. k is the rate constant of isomerization calculated from the fitting function assuming a first-order rate law.

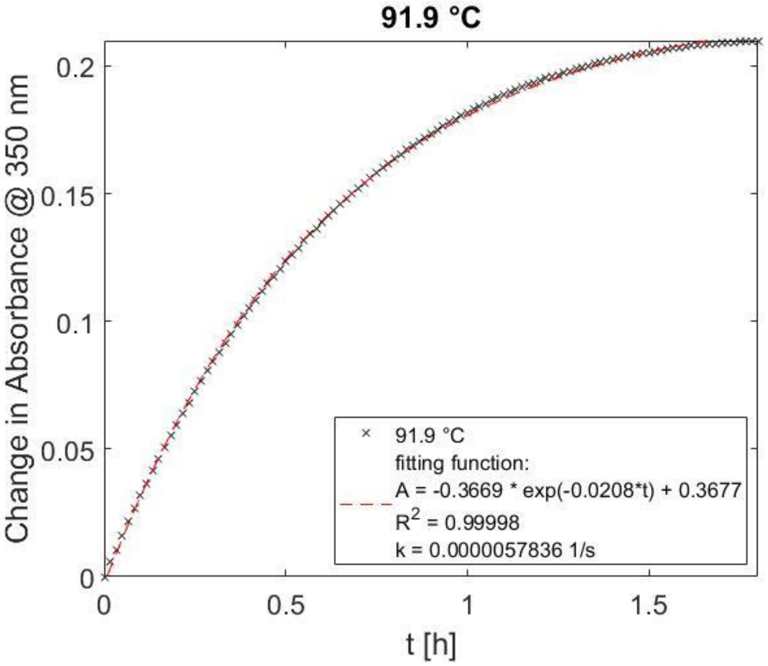

Figure S26: Increase of absorbance at 350 nm and 91.9 °C for a 50 µM solution of **4a** in DMSO after reaching the photostationary state through irradiation at 340 nm measured every 60 s. k is the rate constant of isomerization calculated from the fitting function assuming a first-order rate law.

Eyring Plot for 4a

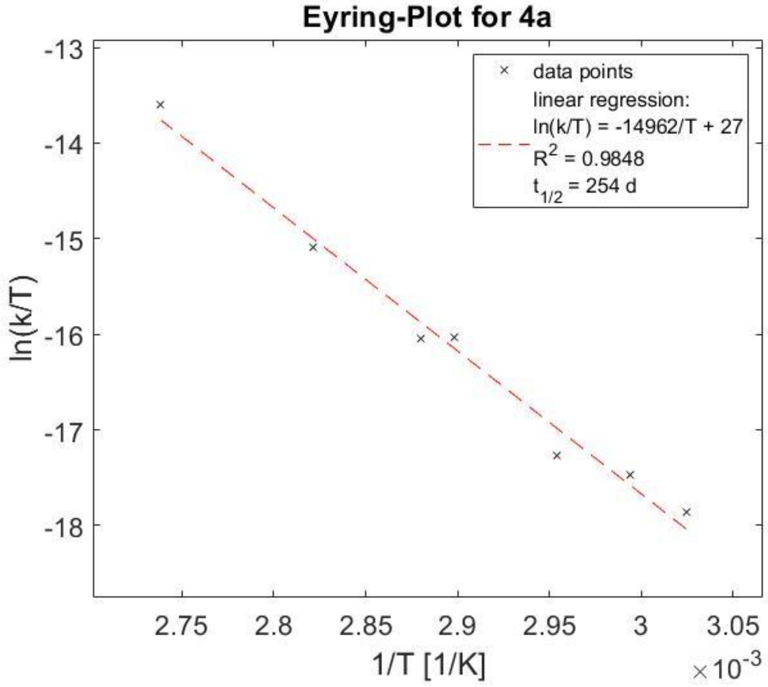

Figure S27: Eyring plot of ln(k/T) against 1/T using the k values obtained for **4a** at different temperatures.

Thermal Relaxation Plots for 4b

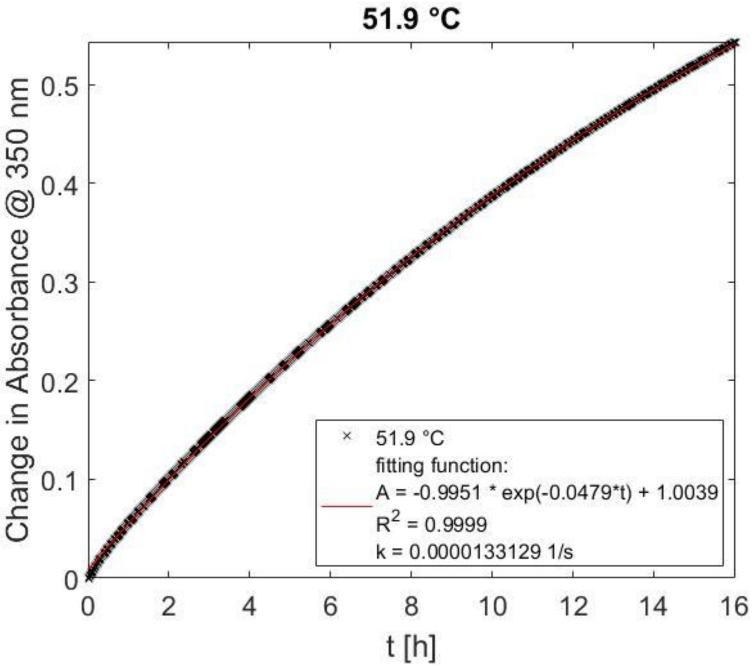

Figure S28: Increase of absorbance at 350 nm and 51.9 °C for a 50 µM solution of **4b** in DMSO after reaching the photostationary state through irradiation at 365 nm measured every 60 s. k is the rate constant of isomerization calculated from the fitting function assuming a first-order rate law.

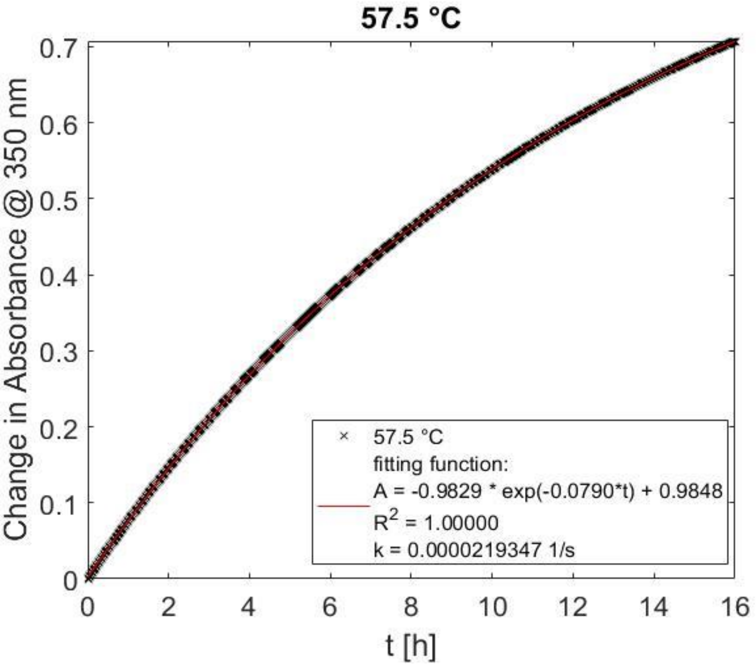

Figure S29: Increase of absorbance at 350 nm and 57.5 °C for a 50 µM solution of **4b** in DMSO after reaching the photostationary state through irradiation at 365 nm measured every 60 s. k is the rate constant of isomerization calculated from the fitting function assuming a first-order rate law.

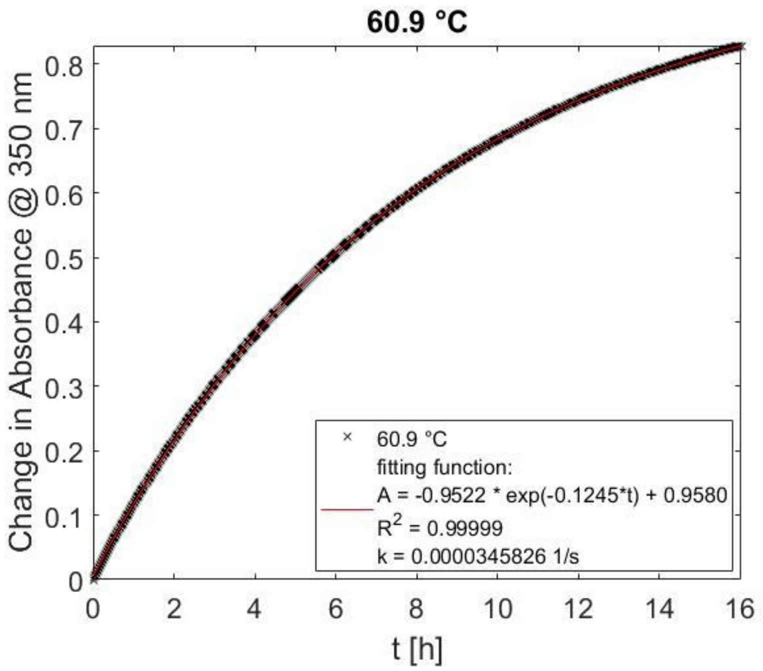

Figure S30: Increase of absorbance at 350 nm and 60.9 °C for a 50 µM solution of **4b** in DMSO after reaching the photostationary state through irradiation at 365 nm measured every 60 s. k is the rate constant of isomerization calculated from the fitting function assuming a first-order rate law.

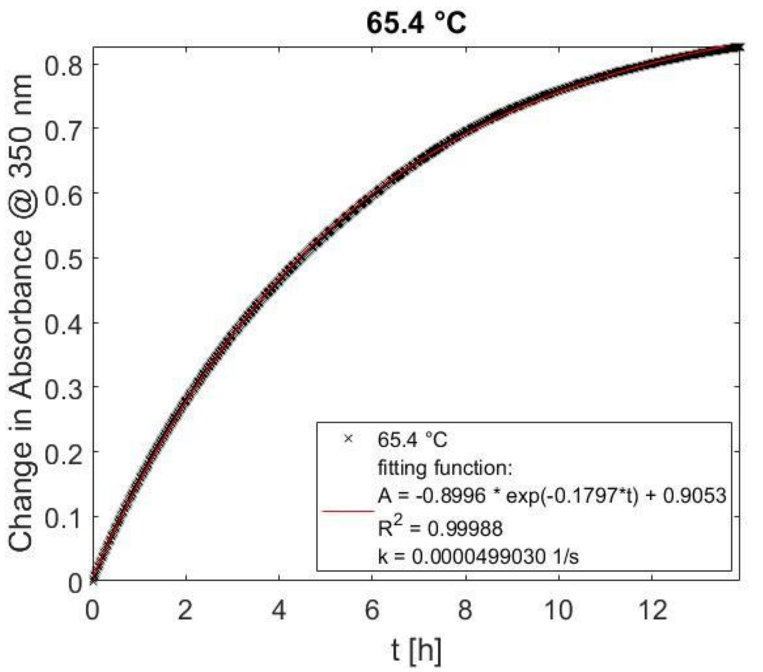

Figure S31: Increase of absorbance at 350 nm and 65.4 °C for a 50 µM solution of **4b** in DMSO after reaching the photostationary state through irradiation at 365 nm measured every 60 s. k is the rate constant of isomerization calculated from the fitting function assuming a first-order rate law.

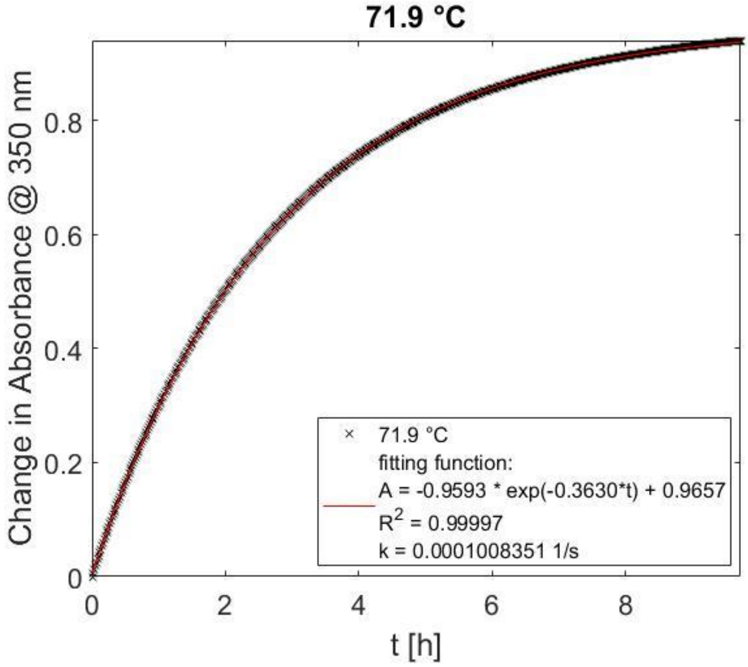

Figure S32: Increase of absorbance at 350 nm and 71.9 °C for a 50 µM solution of **4b** in DMSO after reaching the photostationary state through irradiation at 365 nm measured every 60 s. k is the rate constant of isomerization calculated from the fitting function assuming a first-order rate law.

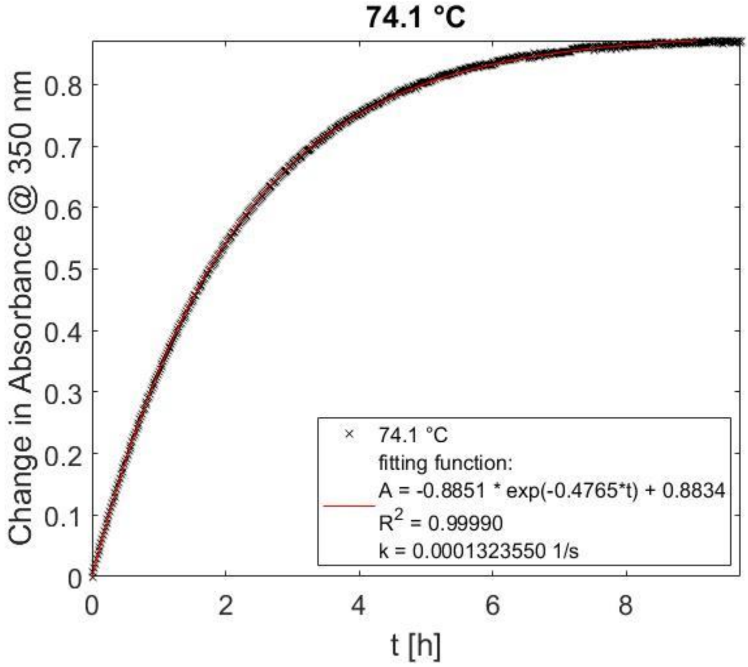

Figure S33: Increase of absorbance at 350 nm and 74.1 °C for a 50 µM solution of **4b** in DMSO after reaching the photostationary state through irradiation at 365 nm measured every 60 s. k is the rate constant of isomerization calculated from the fitting function assuming a first-order rate law.

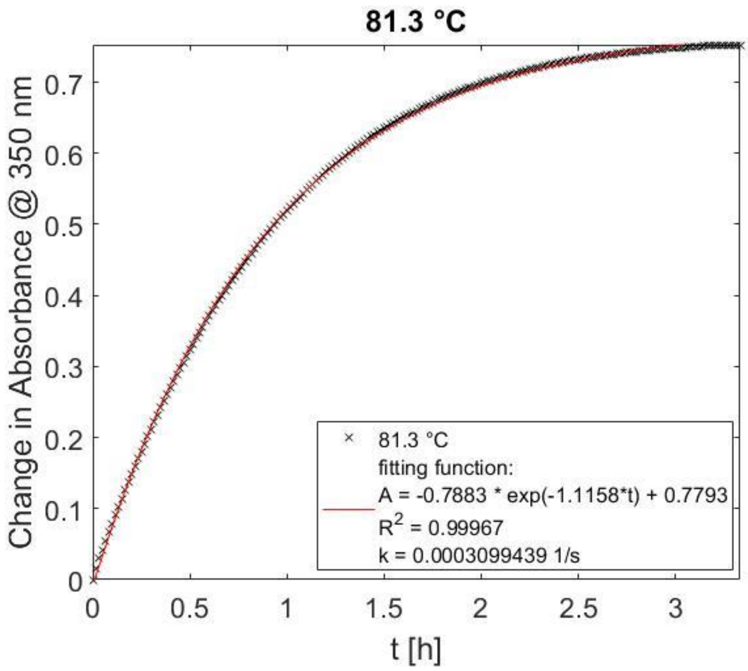

Figure S34: Increase of absorbance at 350 nm and 81.3 °C for a 50 µM solution of **4b** in DMSO after reaching the photostationary state through irradiation at 365 nm measured every 60 s. k is the rate constant of isomerization calculated from the fitting function assuming a first-order rate law.

Eyring Plot for 4b

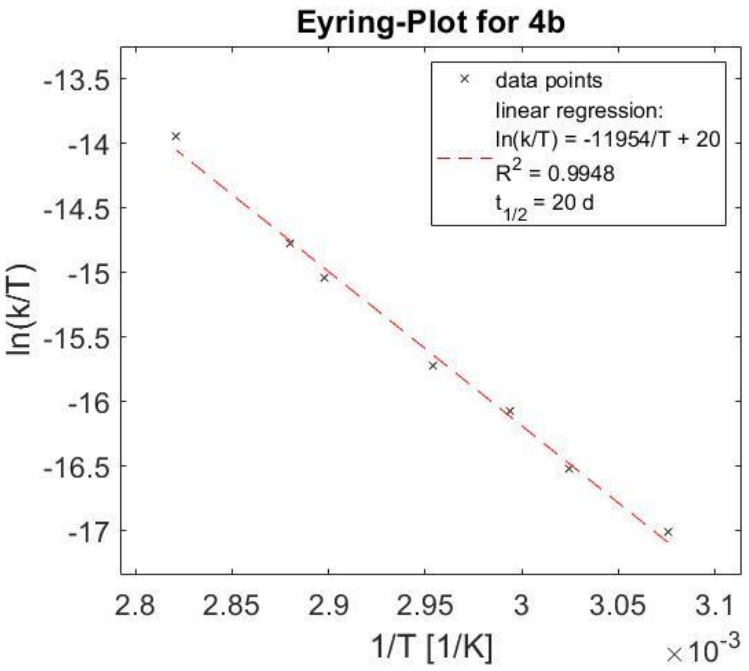

Figure S35: Eyring plot of ln(k/T) against 1/T using the k values obtained for **4b** at different temperatures.

Thermal Relaxation Plots for 4c

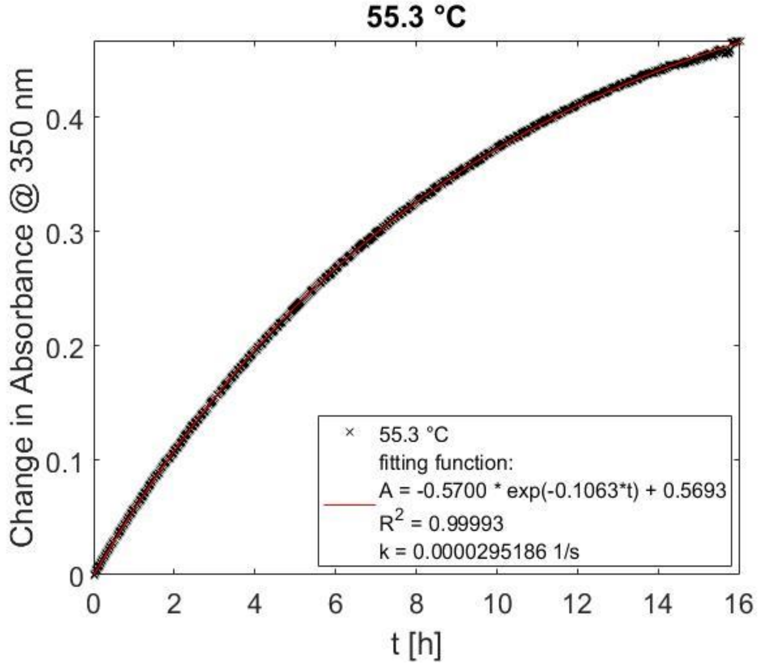

Figure S36: Increase of absorbance at 350 nm and 55.3°C for a 50 µM solution of **4c** in DMSO after reaching the photostationary state through irradiation at 365 nm measured every 60 s. k is the rate constant of isomerization calculated from the fitting function assuming a first-order rate law.

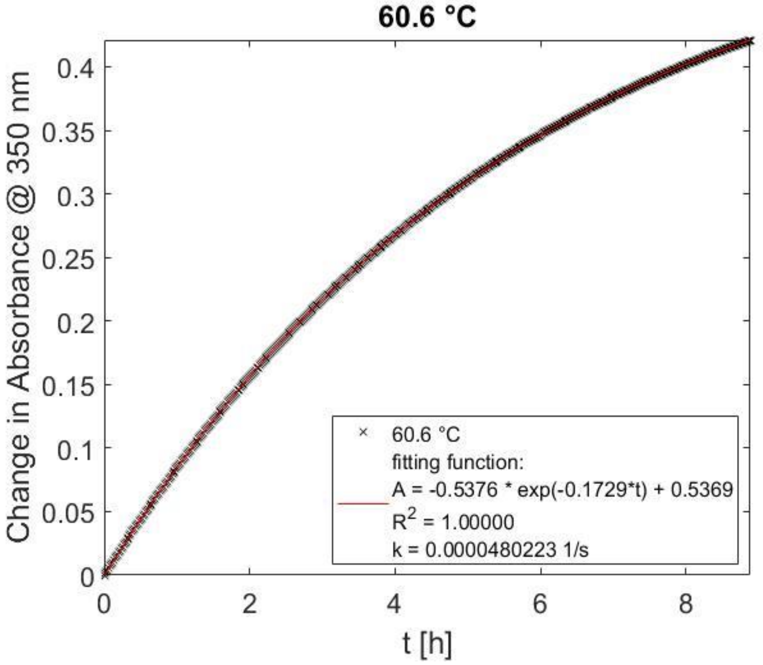

Figure S37: Increase of absorbance at 350 nm and 60.6 °C for a 50 µM solution of **4c** in DMSO after reaching the photostationary state through irradiation at 365 nm measured every 60 s. k is the rate constant of isomerization calculated from the fitting function assuming a first-order rate law.

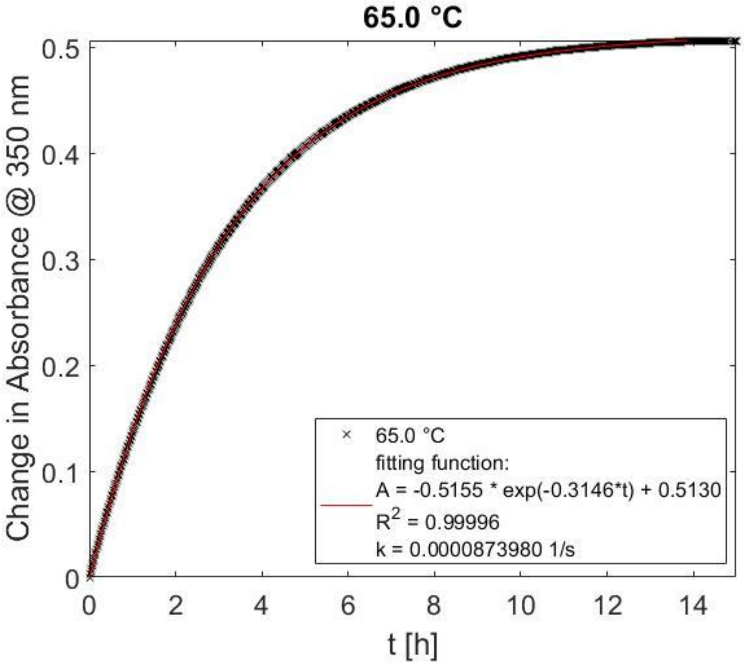

Figure S38: Increase of absorbance at 350 nm and 65.0 °C for a 50 µM solution of **4c** in DMSO after reaching the photostationary state through irradiation at 365 nm measured every 60 s. k is the rate constant of isomerization calculated from the fitting function assuming a first-order rate law.

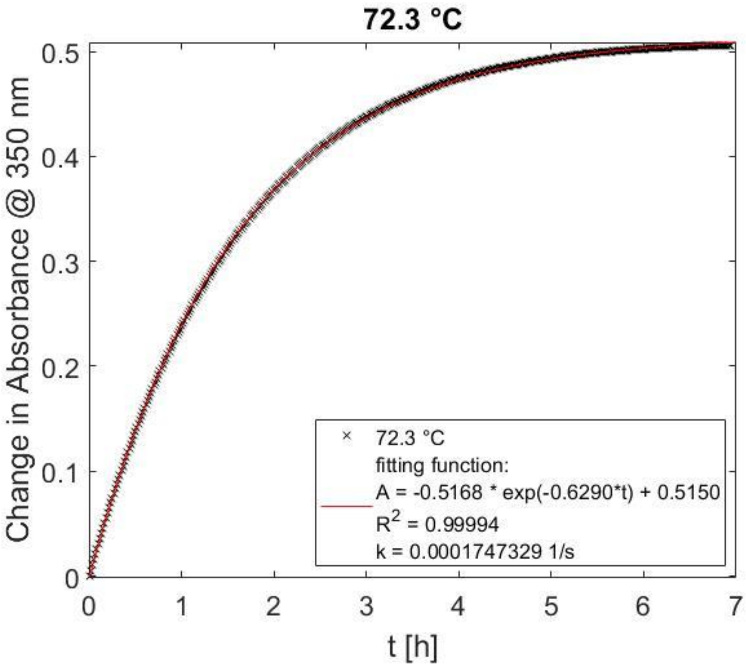

Figure S39: Increase of absorbance at 350 nm and 72.3 °C for a 50 µM solution of **4c** in DMSO after reaching the photostationary state through irradiation at 365 nm measured every 60 s. k is the rate constant of isomerization calculated from the fitting function assuming a first-order rate law.

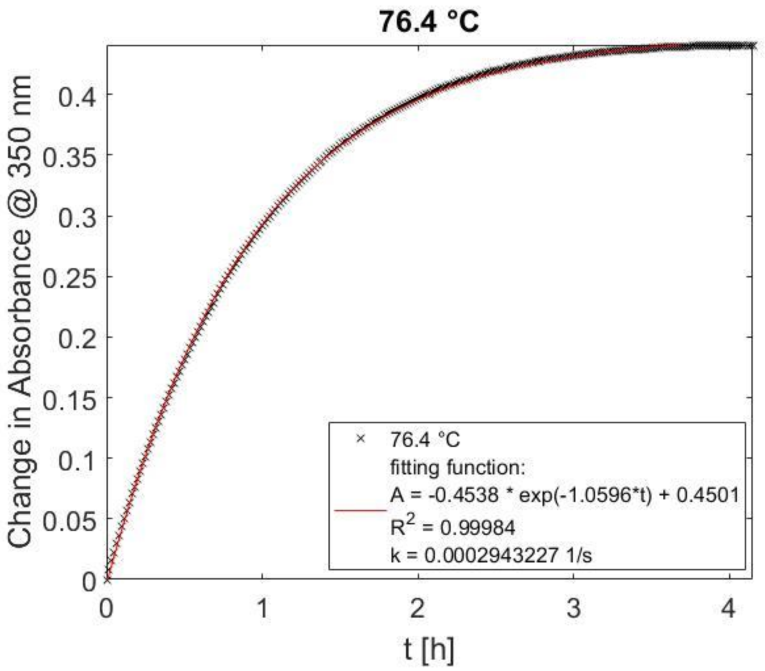

Figure S40: Increase of absorbance at 350 nm and 76.4°C for a 50 µM solution of **4c** in DMSO after reaching the photostationary state through irradiation at 365 nm measured every 60 s. k is the rate constant of isomerization calculated from the fitting function assuming a first-order rate law.

Eyring Plot for 4c

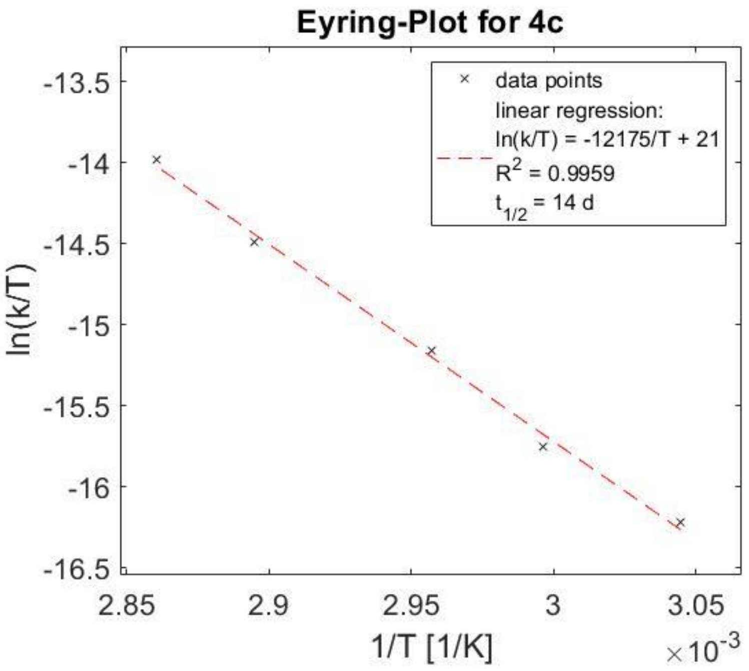

Figure S41: Eyring plot of ln(k/T) against 1/T using the k values obtained for **4c** at different temperatures.

Thermal Relaxation Plots for 4d

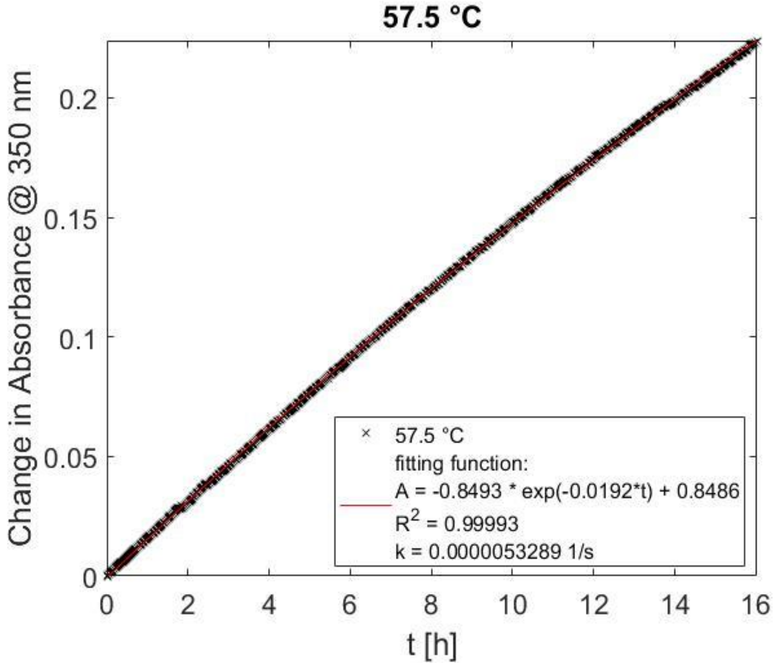

Figure S42: Increase of absorbance at 350 nm and 57.5 °C for a 50 µM solution of **4d** in DMSO after reaching the photostationary state through irradiation at 365 nm measured every 60 s. k is the rate constant of isomerization calculated from the fitting function assuming a first-order rate law.

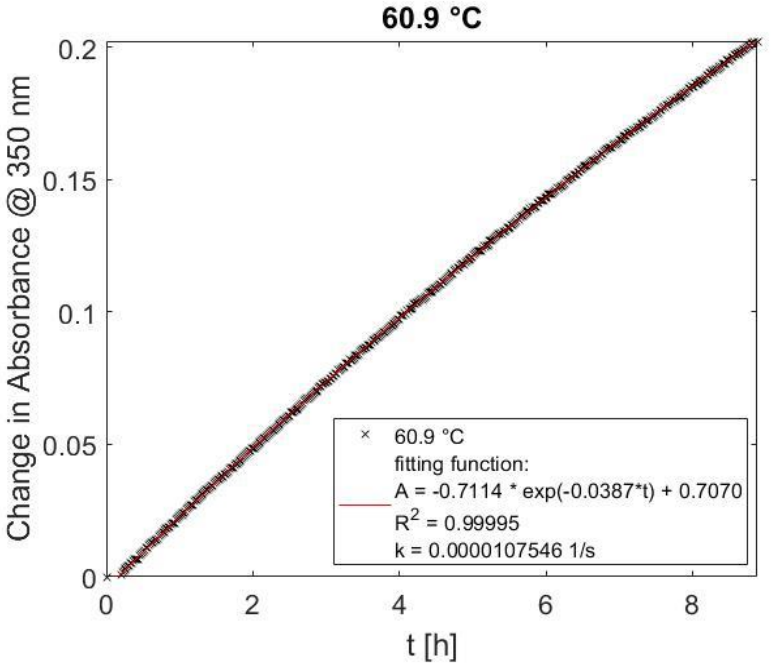

Figure S43: Increase of absorbance at 350 nm and 60.9 °C for a 50 µM solution of **4d** in DMSO after reaching the photostationary state through irradiation at 365 nm measured every 60 s. k is the rate constant of isomerization calculated from the fitting function assuming a first-order rate law.

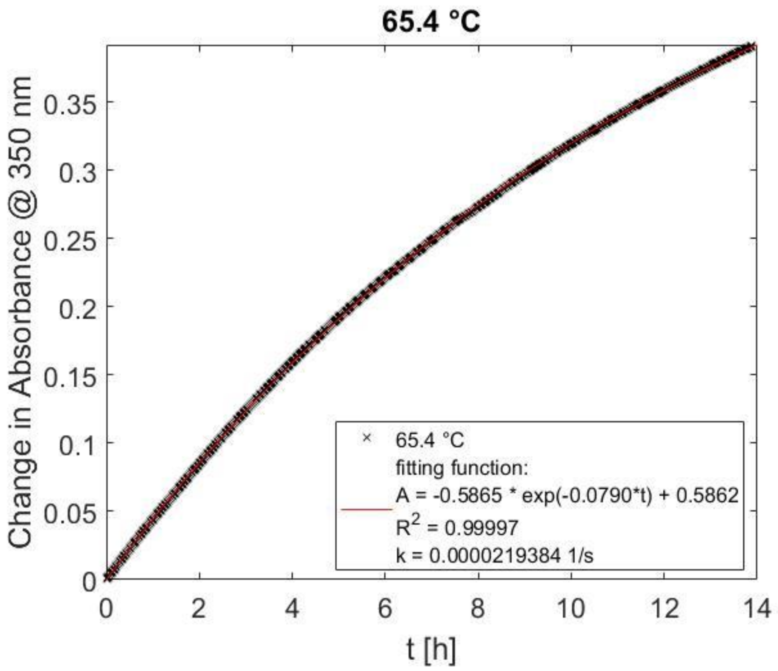

Figure S44: Increase of absorbance at 350 nm and 65.4 °C for a 50 µM solution of **4d** in DMSO after reaching the photostationary state through irradiation at 365 nm measured every 60 s. k is the rate constant of isomerization calculated from the fitting function assuming a first-order rate law.

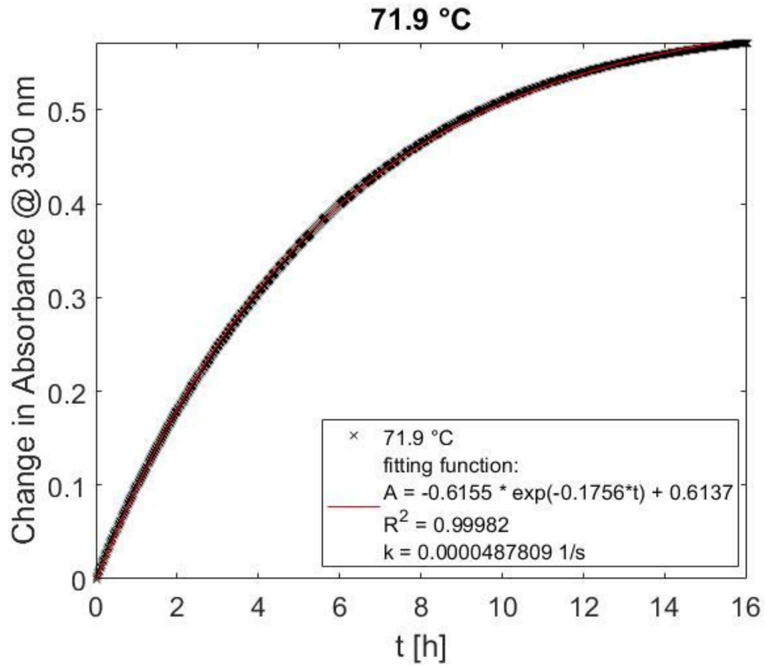

Figure S45: Increase of absorbance at 350 nm and 71.9 °C for a 50 µM solution of **4d** in DMSO after reaching the photostationary state through irradiation at 365 nm measured every 60 s. k is the rate constant of isomerization calculated from the fitting function assuming a first-order rate law.

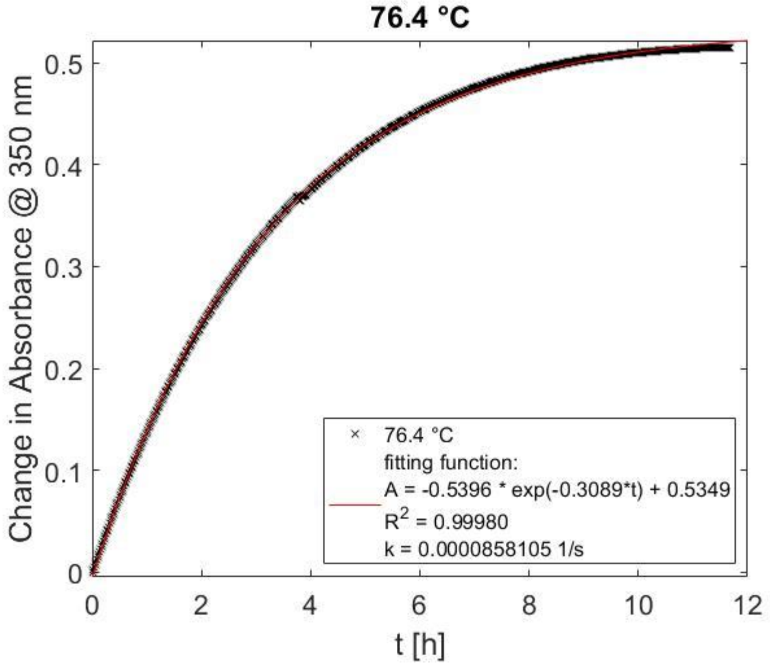

Figure S46: Increase of absorbance at 350 nm and 76.4 °C for a 50 µM solution of **4d** in DMSO after reaching the photostationary state through irradiation at 365 nm measured every 60 s. k is the rate constant of isomerization calculated from the fitting function assuming a first-order rate law.

Eyring Plot for 4d

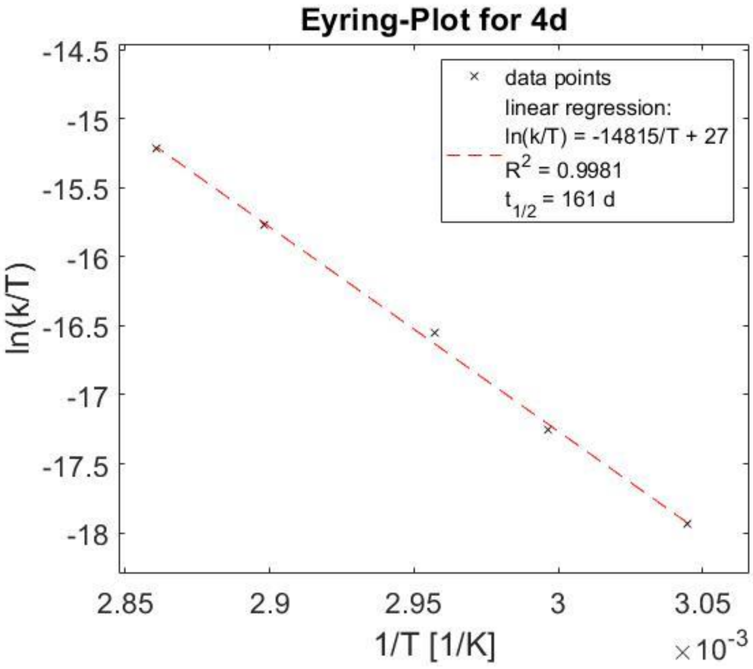

Figure S47: Eyring plot of ln(k/T) against 1/T using the k values obtained for **4d** at different temperatures.

Thermal Relaxation Plots for 4e

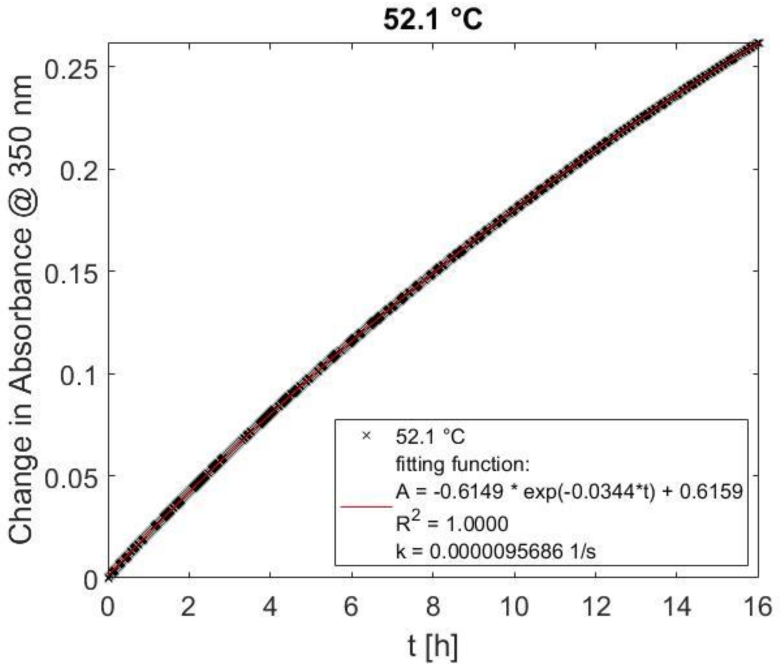

Figure S48: Increase of absorbance at 350 nm and 52.1 °C for a 50 µM solution of **4e** in DMSO after reaching the photostationary state through irradiation at 365 nm measured every 60 s. k is the rate constant of isomerization calculated from the fitting function assuming a first-order rate law.

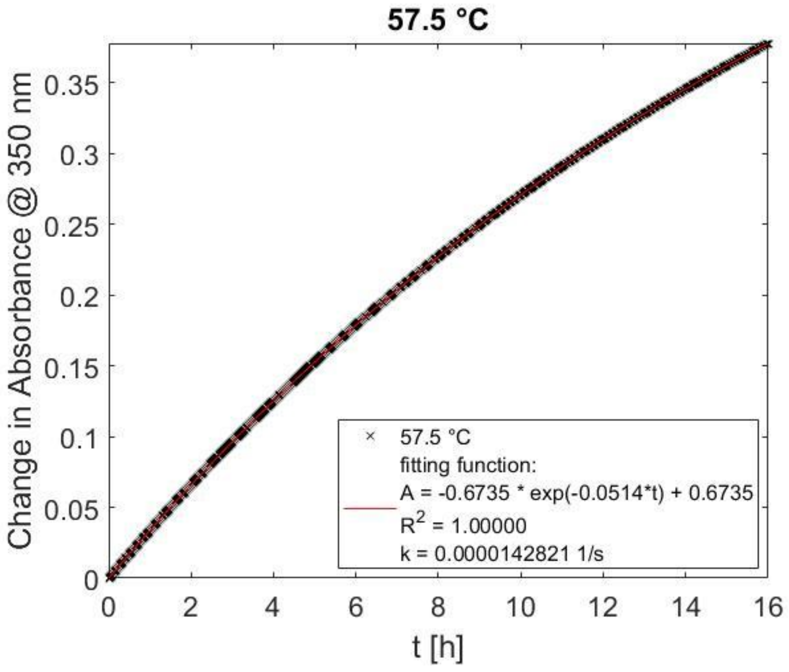

Figure S49: Increase of absorbance at 350 nm and 57.5 °C for a 50 µM solution of **4e** in DMSO after reaching the photostationary state through irradiation at 365 nm measured every 60 s. k is the rate constant of isomerization calculated from the fitting function assuming a first-order rate law.

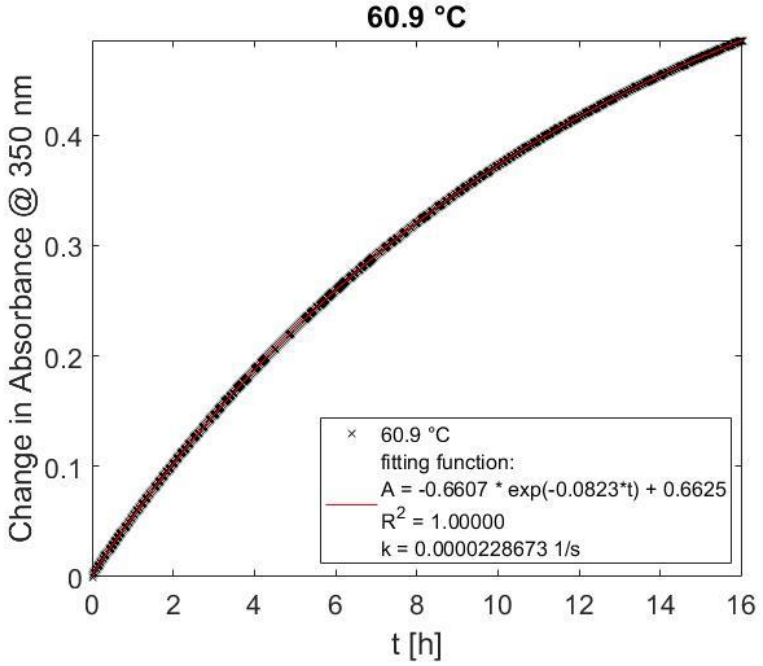

Figure S50: Increase of absorbance at 350 nm and 60.9 °C for a 50 µM solution of **4e** in DMSO after reaching the photostationary state through irradiation at 365 nm measured every 60 s. k is the rate constant of isomerization calculated from the fitting function assuming a first-order rate law.

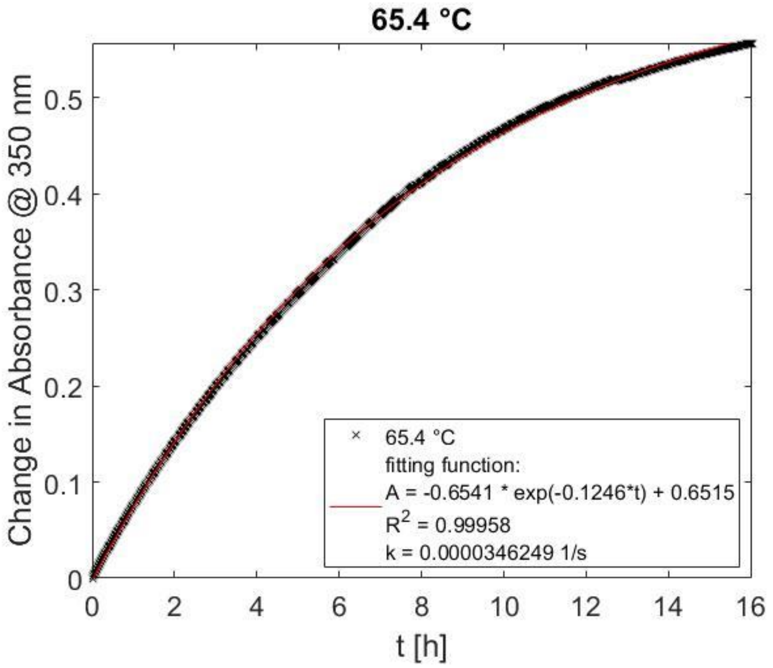

Figure S51: Increase of absorbance at 350 nm and 65.4 °C for a 50 µM solution of **4e** in DMSO after reaching the photostationary state through irradiation at 365 nm measured every 60 s. k is the rate constant of isomerization calculated from the fitting function assuming a first-order rate law.

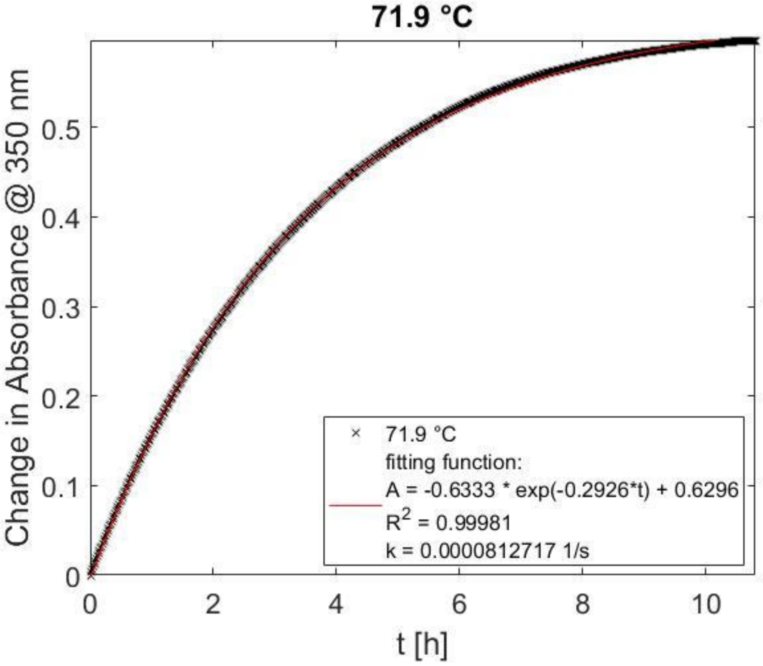

Figure S52: Increase of absorbance at 350 nm and 71.9 °C for a 50 µM solution of **4e** in DMSO after reaching the photostationary state through irradiation at 365 nm measured every 60 s. k is the rate constant of isomerization calculated from the fitting function assuming a first-order rate law.

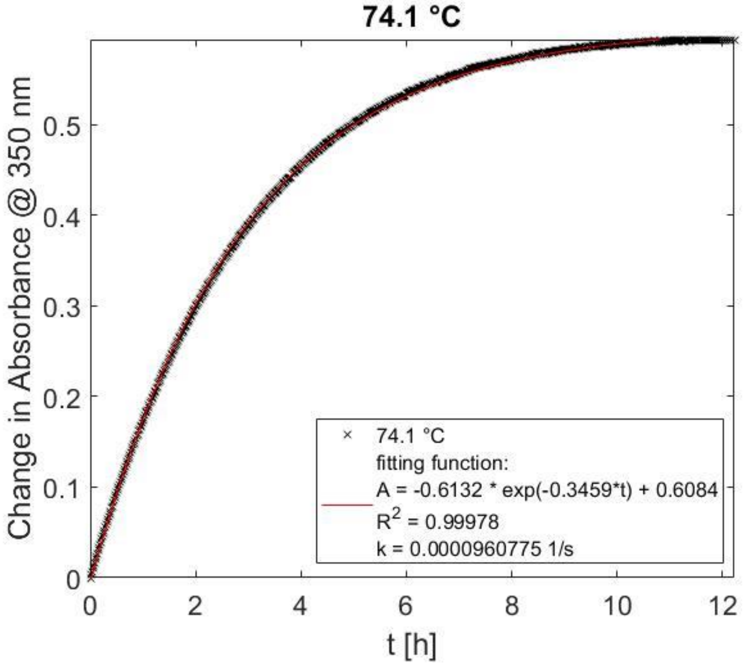

Figure S53: Increase of absorbance at 350 nm and 74.1 °C for a 50 µM solution of **4e** in DMSO after reaching the photostationary state through irradiation at 365 nm measured every 60 s. k is the rate constant of isomerization calculated from the fitting function assuming a first-order rate law.

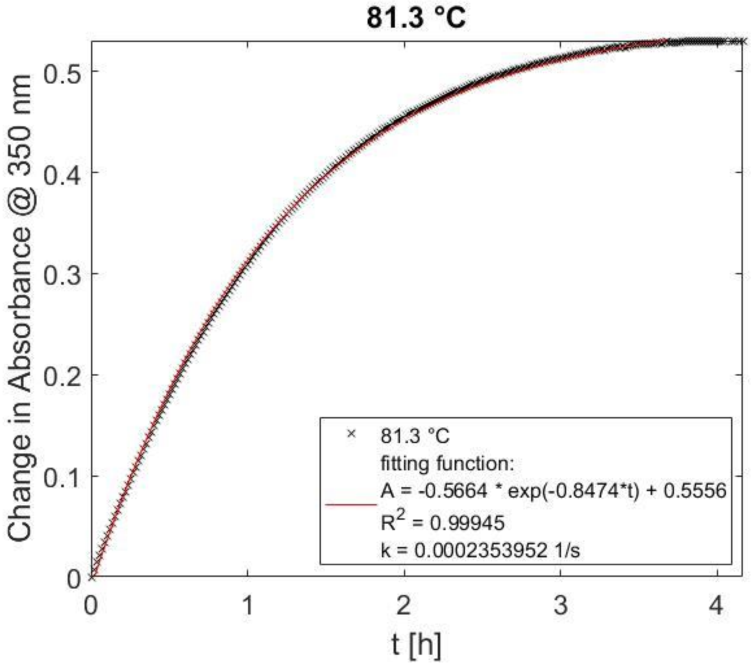

Figure S54: Increase of absorbance at 350 nm and 81.3 °C for a 50 µM solution of **4e** in DMSO after reaching the photostationary state through irradiation at 365 nm measured every 60 s. k is the rate constant of isomerization calculated from the fitting function assuming a first-order rate law.

Eyring Plot for 4e

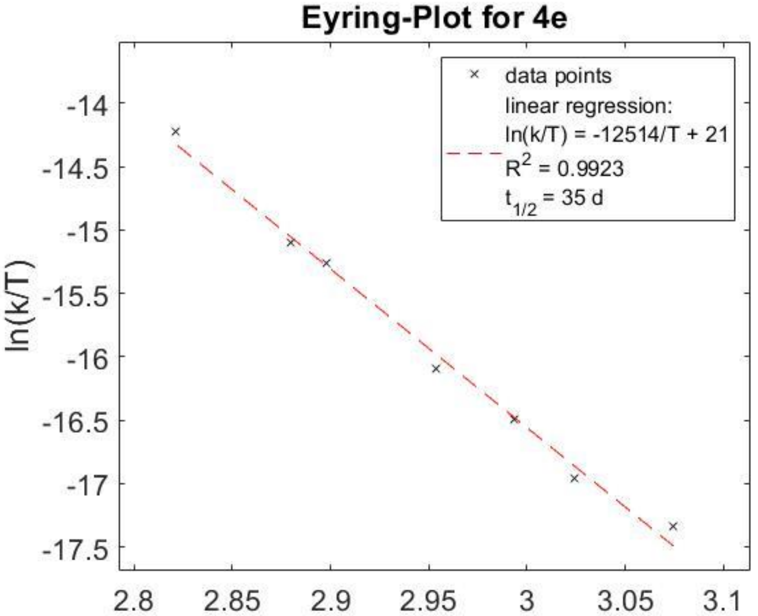

Figure S55: Eyring plot of ln(k/T) against 1/T using the k values obtained for **4e** at different temperatures.

Thermal Relaxation Plots for 4f

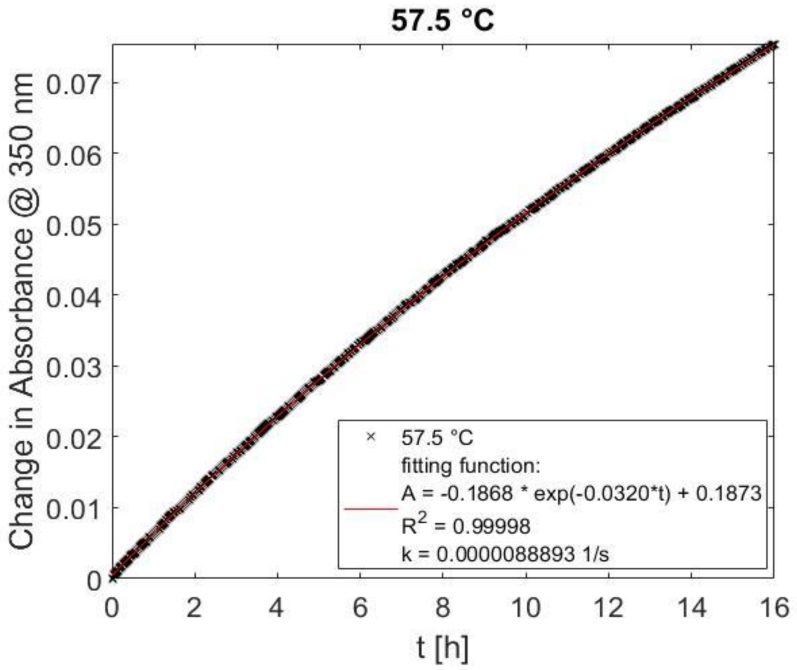

Figure S56: Increase of absorbance at 350 nm and 57.5 °C for a 50 µM solution of **4f** in DMSO after reaching the photostationary state through irradiation at 365 nm measured every 60 s. k is the rate constant of isomerization calculated from the fitting function assuming a first-order rate law.

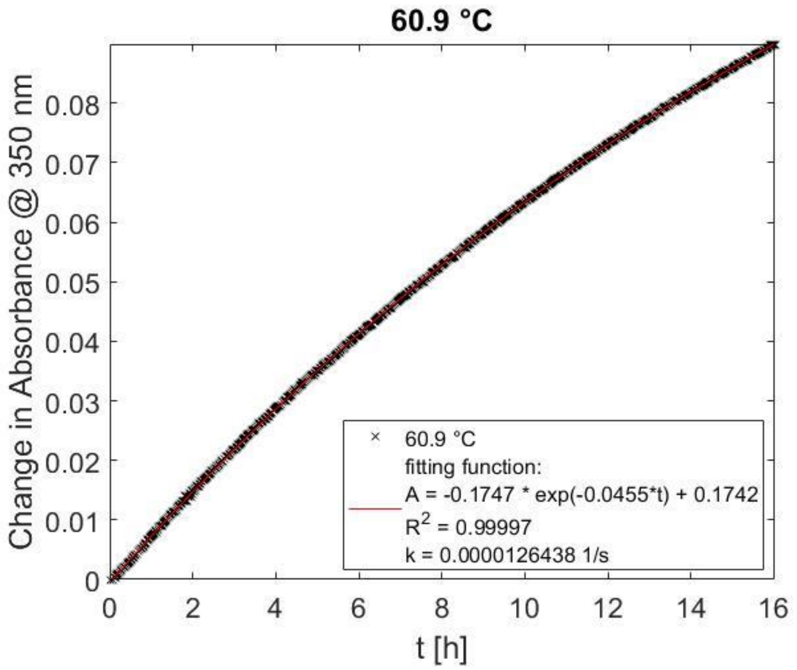

Figure S57: Increase of absorbance at 350 nm and 60.9 °C for a 50 µM solution of **4f** in DMSO after reaching the photostationary state through irradiation at 365 nm measured every 60 s. k is the rate constant of isomerization calculated from the fitting function assuming a first-order rate law.

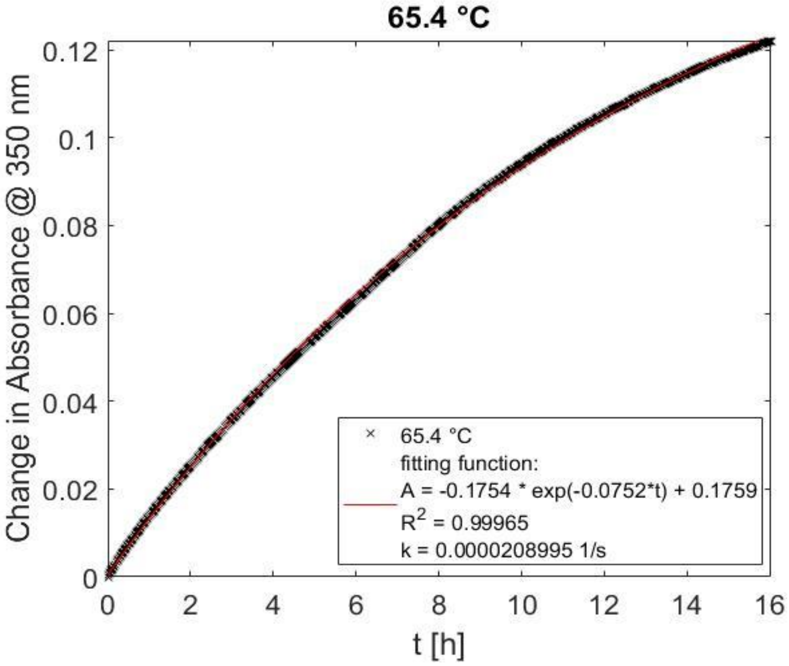

Figure S58: Increase of absorbance at 350 nm and 65.4 °C for a 50 µM solution of **4f** in DMSO after reaching the photostationary state through irradiation at 365 nm measured every 60 s. k is the rate constant of isomerization calculated from the fitting function assuming a first-order rate law.

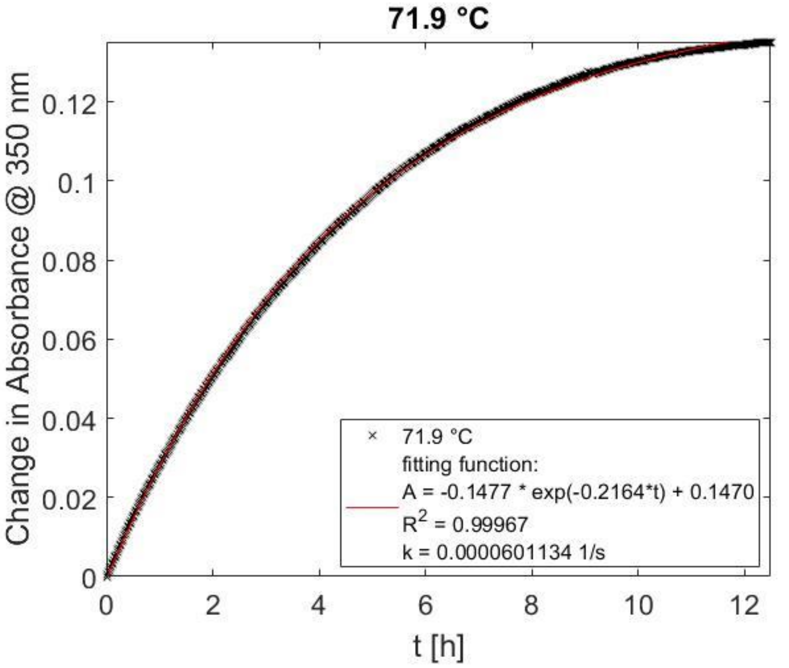

Figure S59: Increase of absorbance at 350 nm and 71.9 °C for a 50 µM solution of **4f** in DMSO after reaching the photostationary state through irradiation at 365 nm measured every 60 s. k is the rate constant of isomerization calculated from the fitting function assuming a first-order rate law.

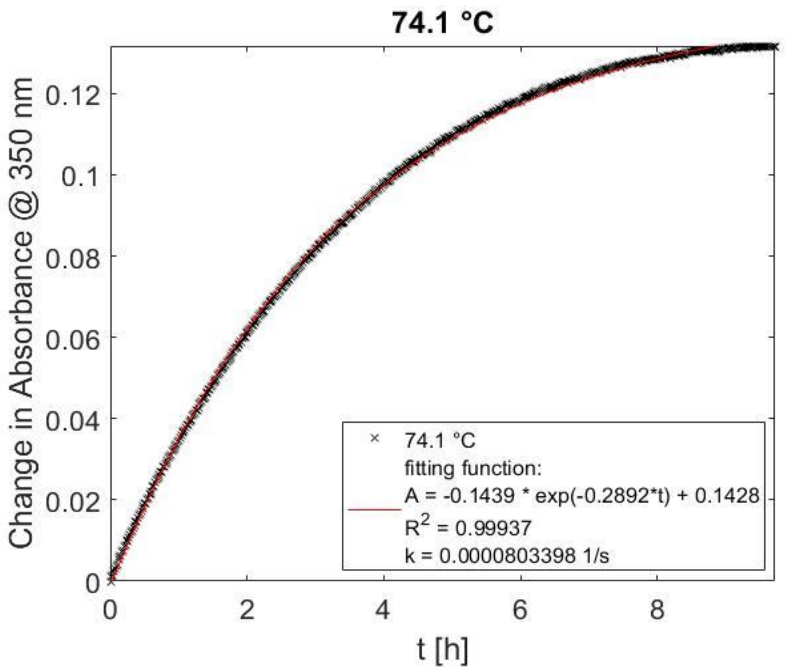

Figure S60: Increase of absorbance at 350 nm and 74.1 °C for a 50 µM solution of **4f** in DMSO after reaching the photostationary state through irradiation at 365 nm measured every 60 s. k is the rate constant of isomerization calculated from the fitting function assuming a first-order rate law.

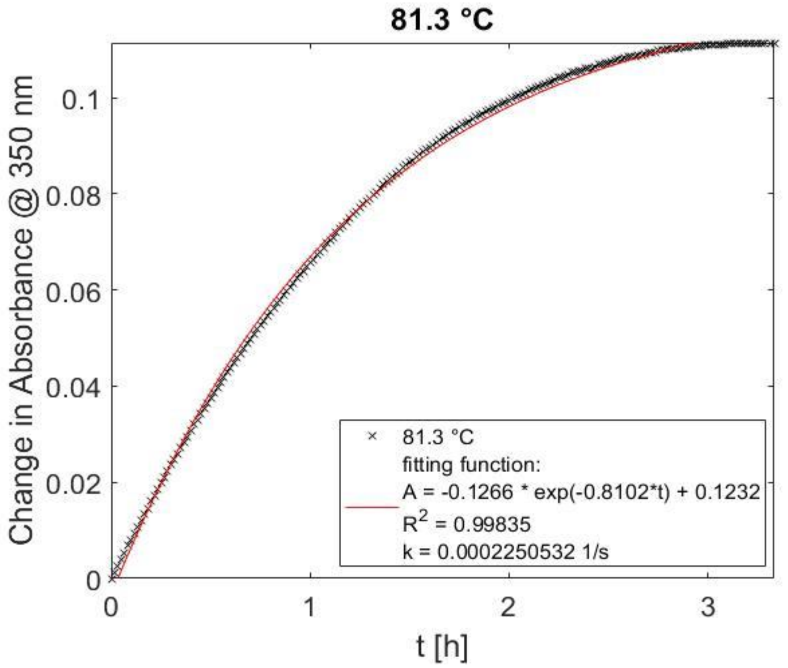

Figure S61: Increase of absorbance at 350 nm and 81.3 °C for a 50 µM solution of **4f** in DMSO after reaching the photostationary state through irradiation at 365 nm measured every 60 s. k is the rate constant of isomerization calculated from the fitting function assuming a first-order rate law.

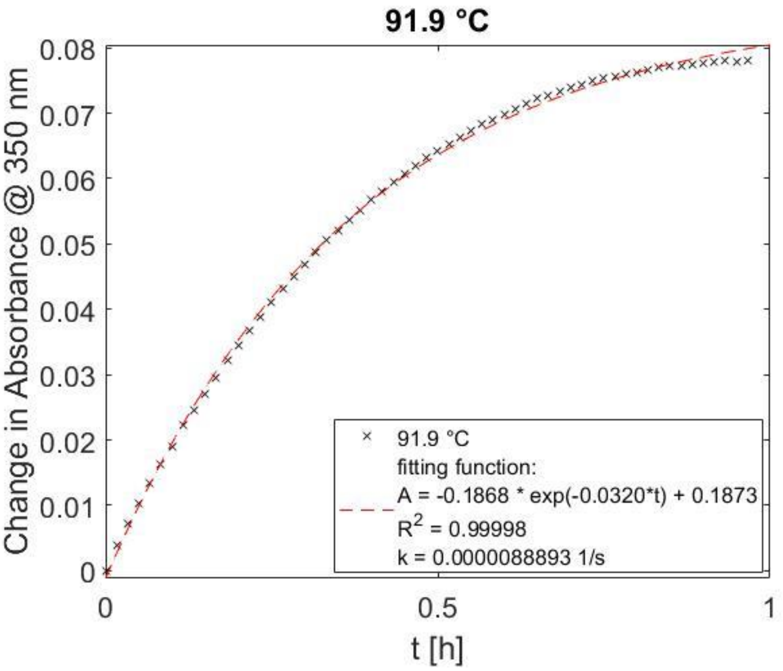

Figure S62: Increase of absorbance at 350 nm and 91.9 °C for a 50 µM solution of **4f** in DMSO after reaching the photostationary state through irradiation at 365 nm measured every 60 s. k is the rate constant of isomerization calculated from the fitting function assuming a first-order rate law.

Eyring Plot for 4f

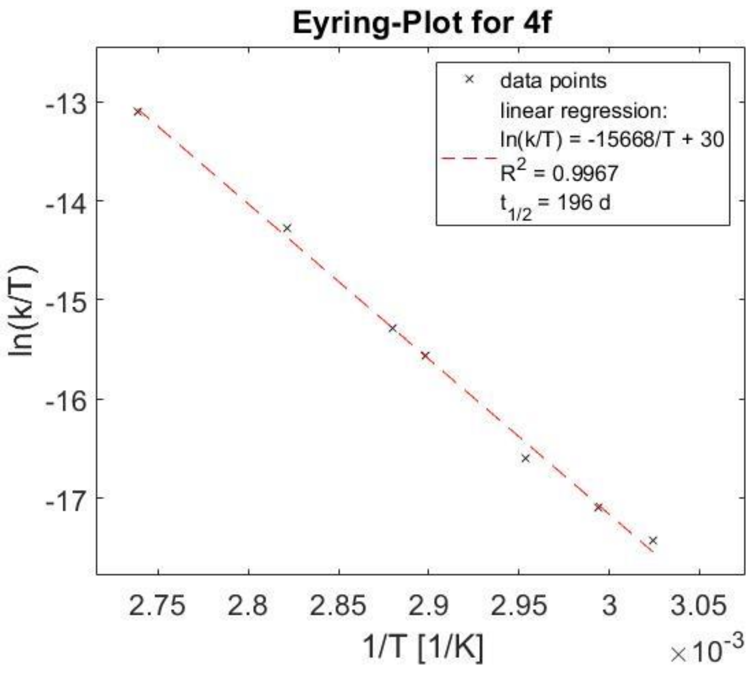

Figure S63: Eyring plot of ln(k/T) against 1/T using the k values obtained for **4f** at different temperatures.

Thermal Relaxation Plots for 4g

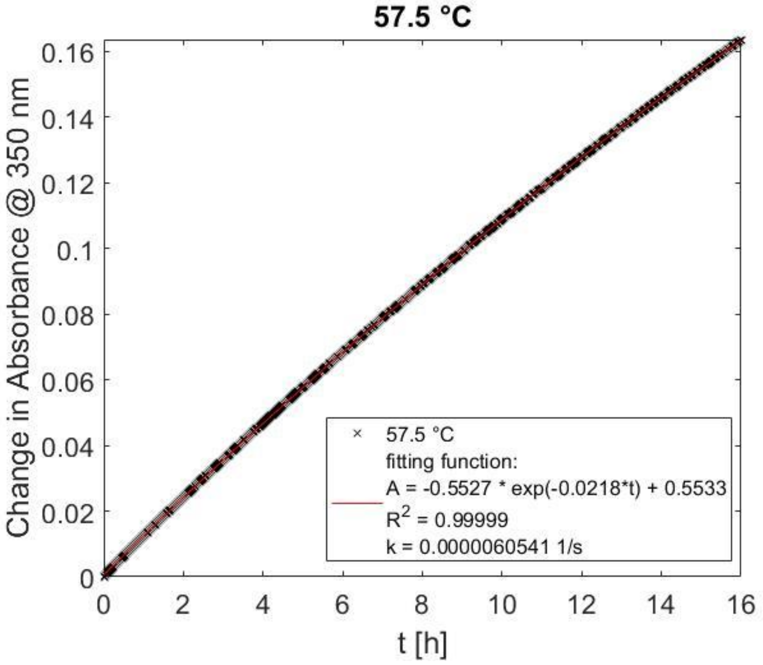

Figure S64: Increase of absorbance at 350 nm and 57.5 °C for a 50 µM solution of **4g** in DMSO after reaching the photostationary state through irradiation at 365 nm measured every 60 s. k is the rate constant of isomerization calculated from the fitting function assuming a first-order rate law.

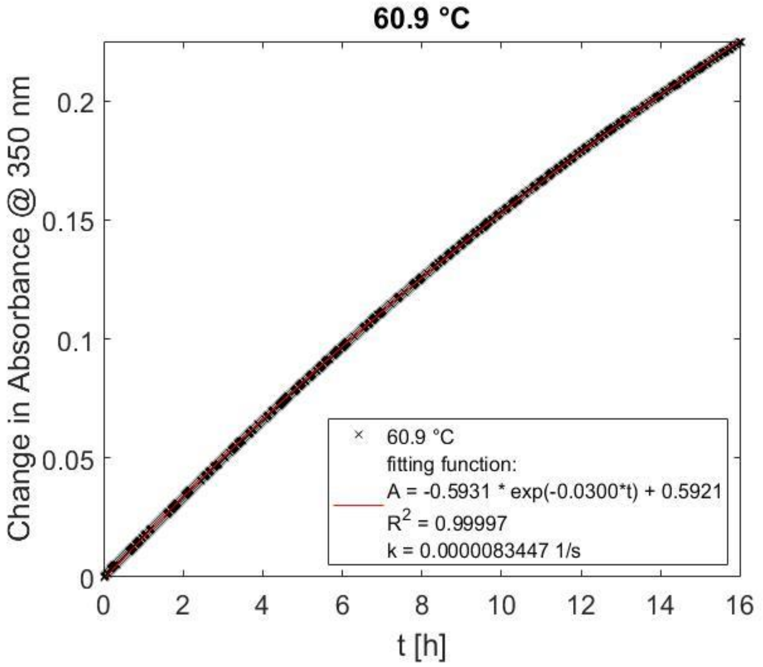

Figure S65: Increase of absorbance at 350 nm and 60.9 °C for a 50 µM solution of **4g** in DMSO after reaching the photostationary state through irradiation at 365 nm measured every 60 s. k is the rate constant of isomerization calculated from the fitting function assuming a first-order rate law.

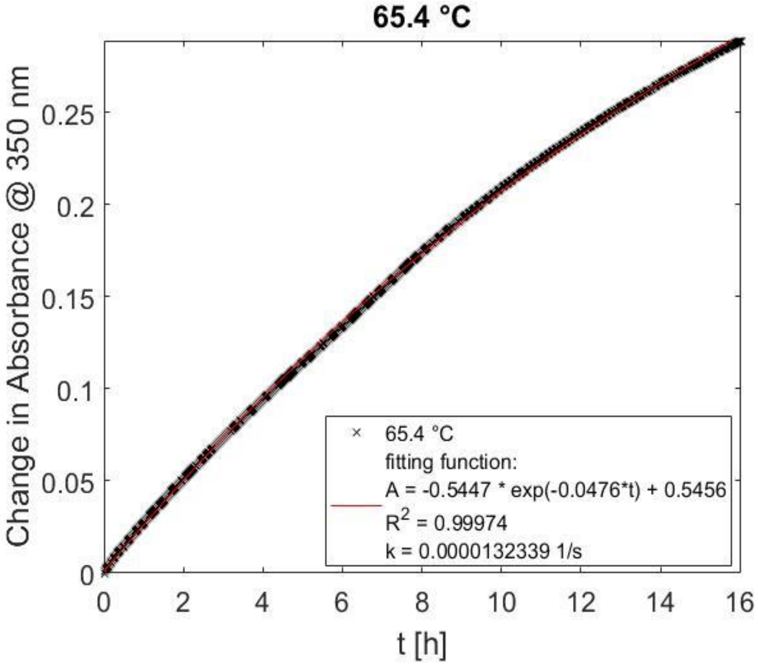

Figure S66: Increase of absorbance at 350 nm and 65.4 °C for a 50 µM solution of **4g** in DMSO after reaching the photostationary state through irradiation at 365 nm measured every 60 s. k is the rate constant of isomerization calculated from the fitting function assuming a first-order rate law.

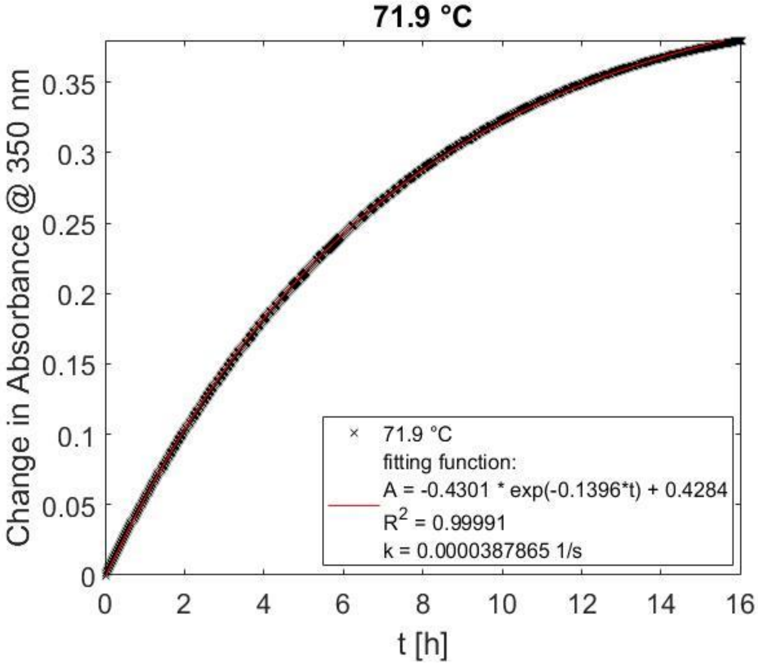

Figure S67: Increase of absorbance at 350 nm and 71.9 °C for a 50 µM solution of **4g** in DMSO after reaching the photostationary state through irradiation at 365 nm measured every 60 s. k is the rate constant of isomerization calculated from the fitting function assuming a first-order rate law.

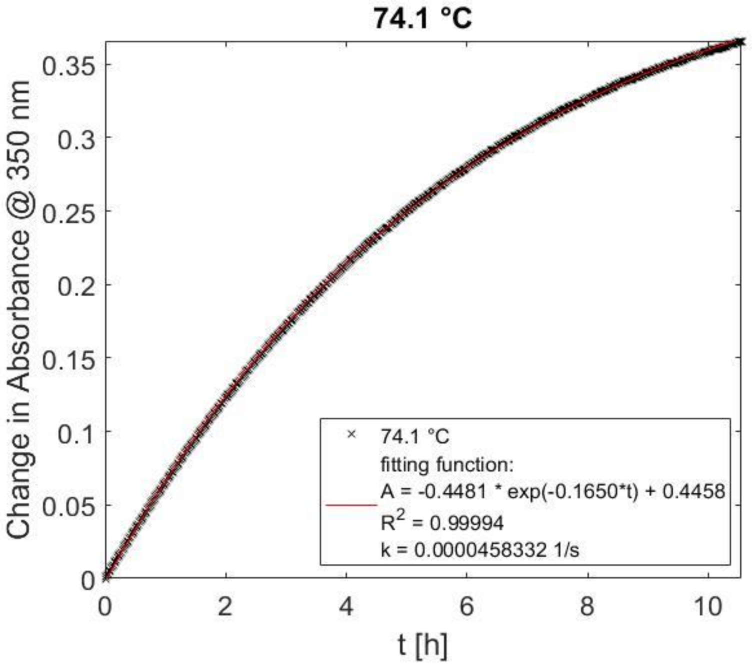

Figure S68: Increase of absorbance at 350 nm and 74.1 °C for a 50 µM solution of **4g** in DMSO after reaching the photostationary state through irradiation at 365 nm measured every 60 s. k is the rate constant of isomerization calculated from the fitting function assuming a first-order rate law.

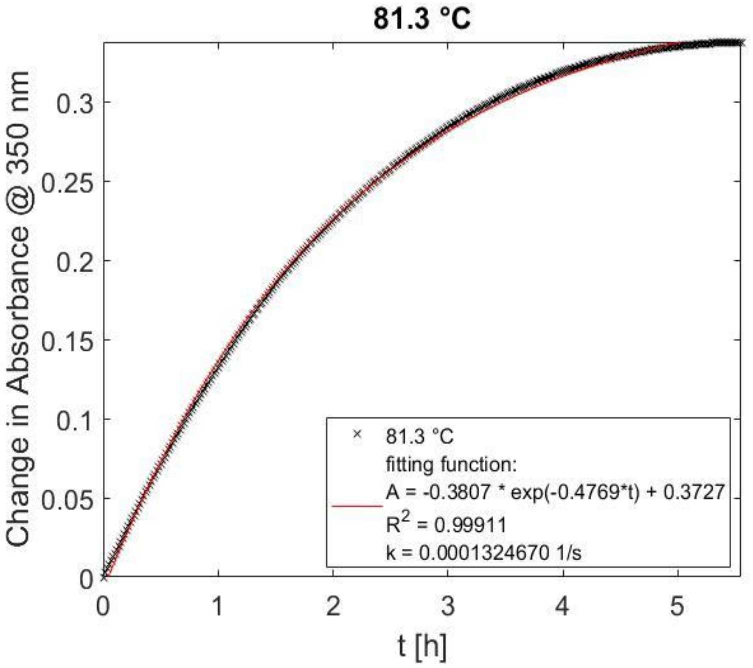

Figure S69: Increase of absorbance at 350 nm and 81.3 °C for a 50 µM solution of **4g** in DMSO after reaching the photostationary state through irradiation at 365 nm measured every 60 s. k is the rate constant of isomerization calculated from the fitting function assuming a first-order rate law.

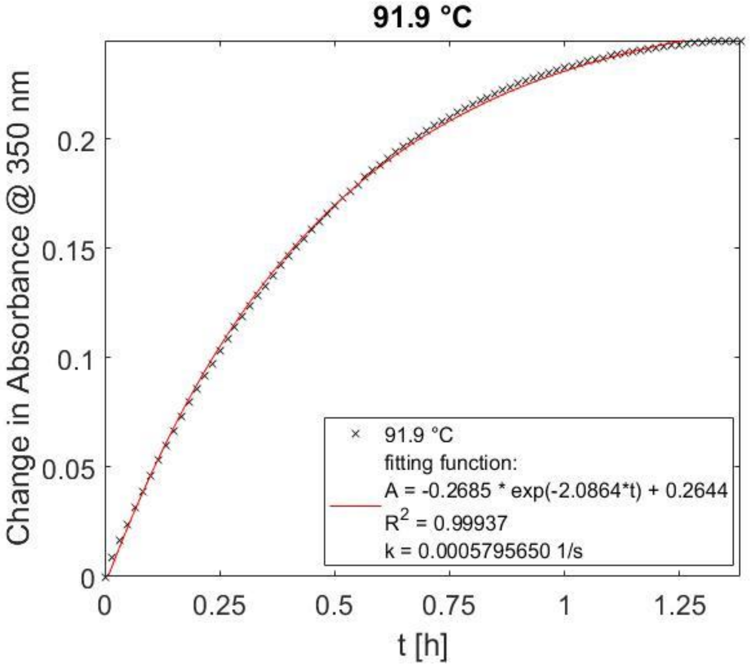

Figure S70: Increase of absorbance at 350 nm and 91.9 °C for a 50 µM solution of **4g** in DMSO after reaching the photostationary state through irradiation at 365 nm measured every 60 s. k is the rate constant of isomerization calculated from the fitting function assuming a first-order rate law.

Eyring Plot for 4g

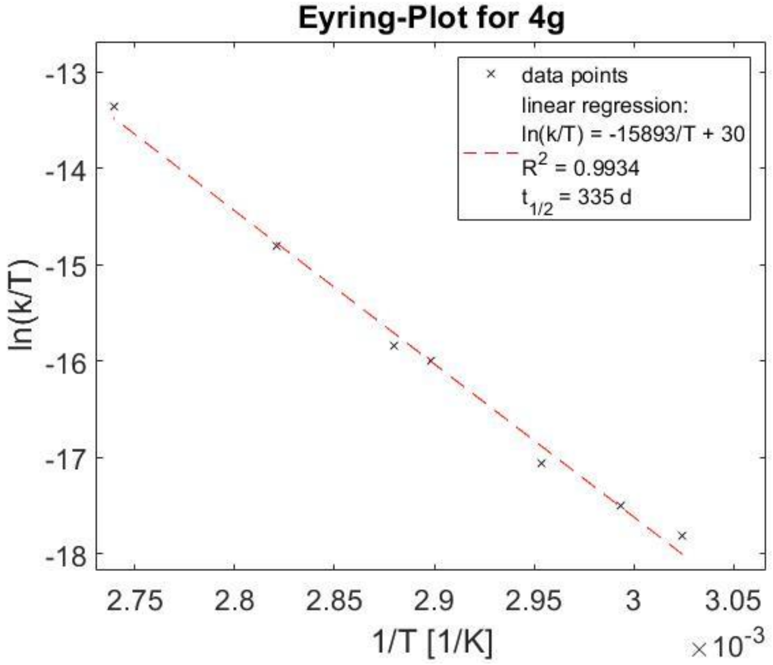

Figure S71: Eyring plot of ln(k/T) against 1/T using the k values obtained for **4g** at different temperatures.

Thermal Relaxation Plots for 4m

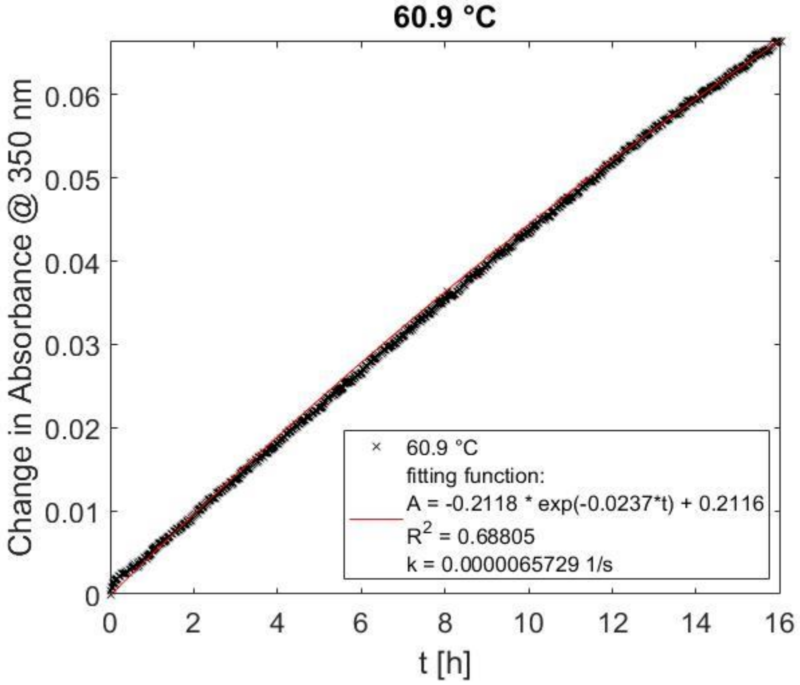

Figure S72: Increase of absorbance at 350 nm and 60.9 °C for a 50 µM solution of **4m** in DMSO after reaching the photostationary state through irradiation at 365 nm measured every 60 s. k is the rate constant of isomerization calculated from the fitting function assuming a first-order rate law.

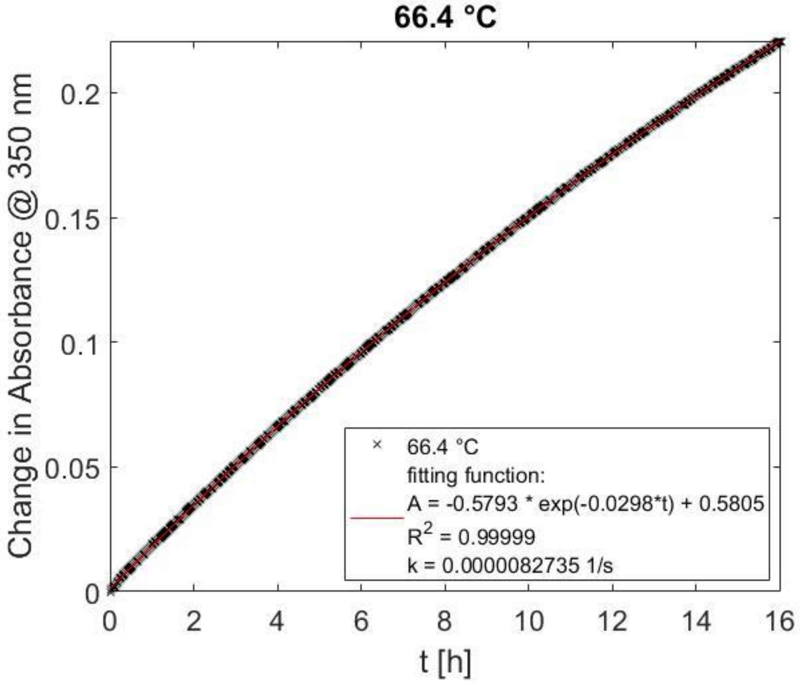

Figure S73: Increase of absorbance at 350 nm and 66.4 °C for a 50 µM solution of **4m** in DMSO after reaching the photostationary state through irradiation at 365 nm measured every 60 s. k is the rate constant of isomerization calculated from the fitting function assuming a first-order rate law.

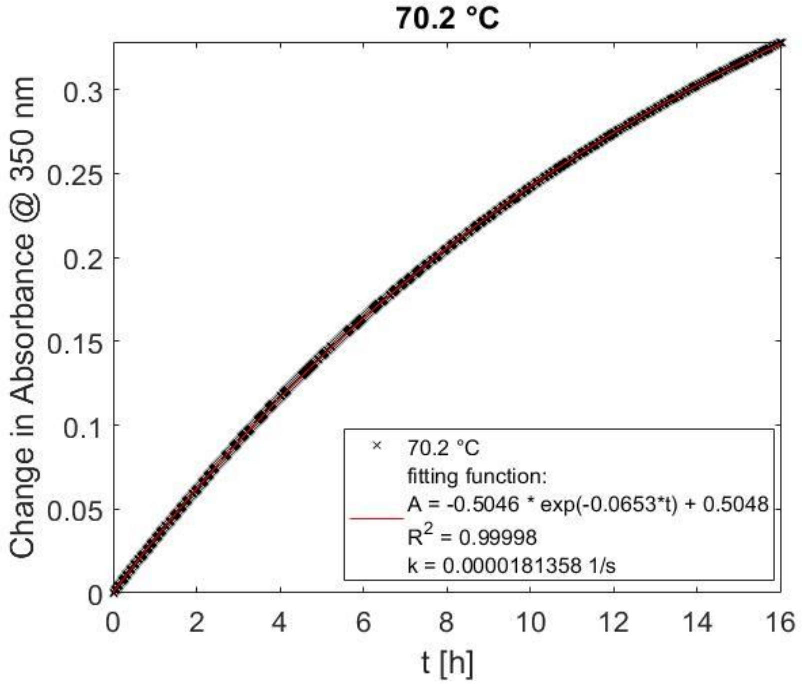

Figure S74: Increase of absorbance at 350 nm and 70.2 °C for a 50 µM solution of **4m** in DMSO after reaching the photostationary state through irradiation at 365 nm measured every 60 s. k is the rate constant of isomerization calculated from the fitting function assuming a first-order rate law.

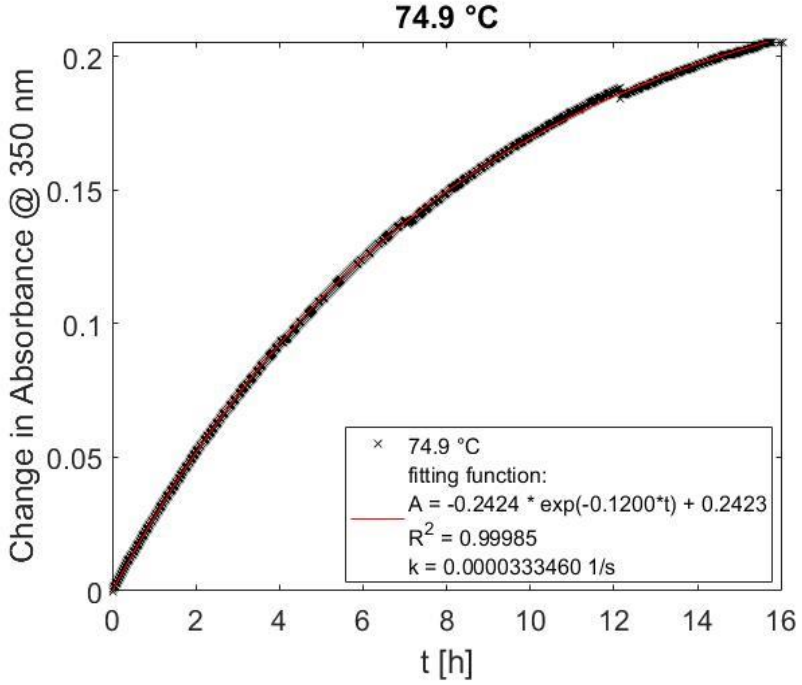

Figure S75: Increase of absorbance at 350 nm and 74.9°C for a 50 µM solution of **4m** in DMSO after reaching the photostationary state through irradiation at 365 nm measured every 60 s. k is the rate constant of isomerization calculated from the fitting function assuming a first-order rate law.

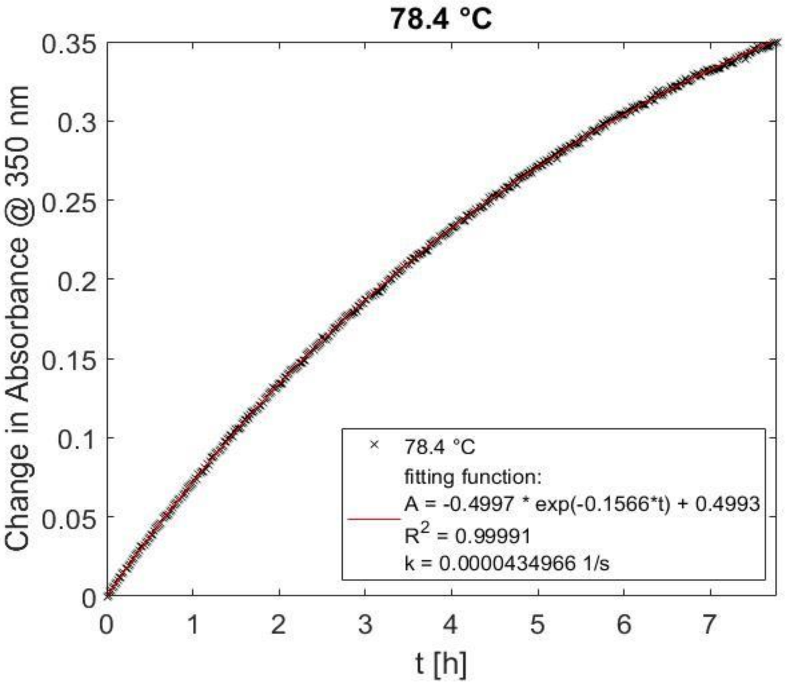

Figure S76: Increase of absorbance at 350 nm and 78.4°C for a 50 µM solution of **4m** in DMSO after reaching the photostationary state through irradiation at 365 nm measured every 60 s. k is the rate constant of isomerization calculated from the fitting function assuming a first-order rate law.

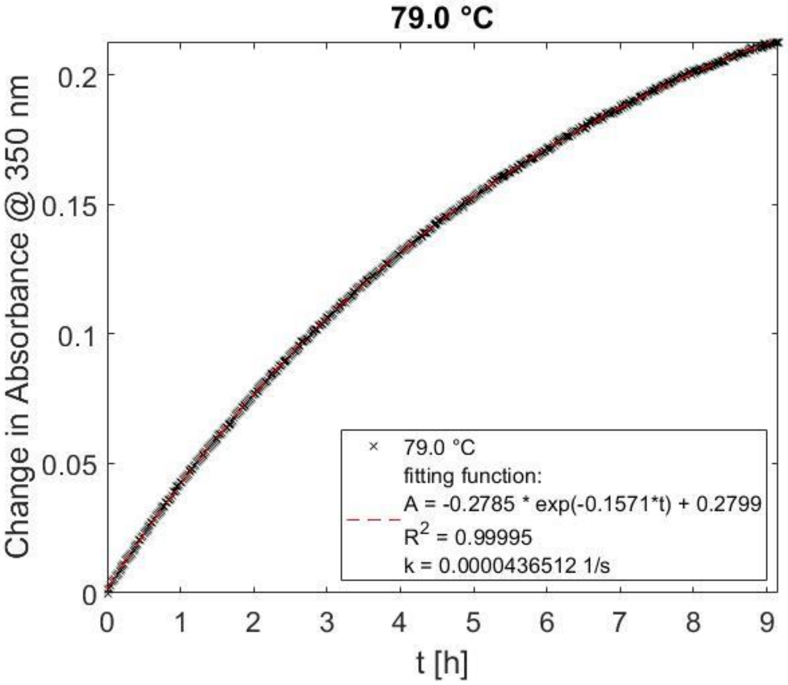

Figure S77: Increase of absorbance at 350 nm and 79.0 °C for a 50 µM solution of **4m** in DMSO after reaching the photostationary state through irradiation at 365 nm measured every 60 s. k is the rate constant of isomerization calculated from the fitting function assuming a first-order rate law.

Eyring Plot for 4m

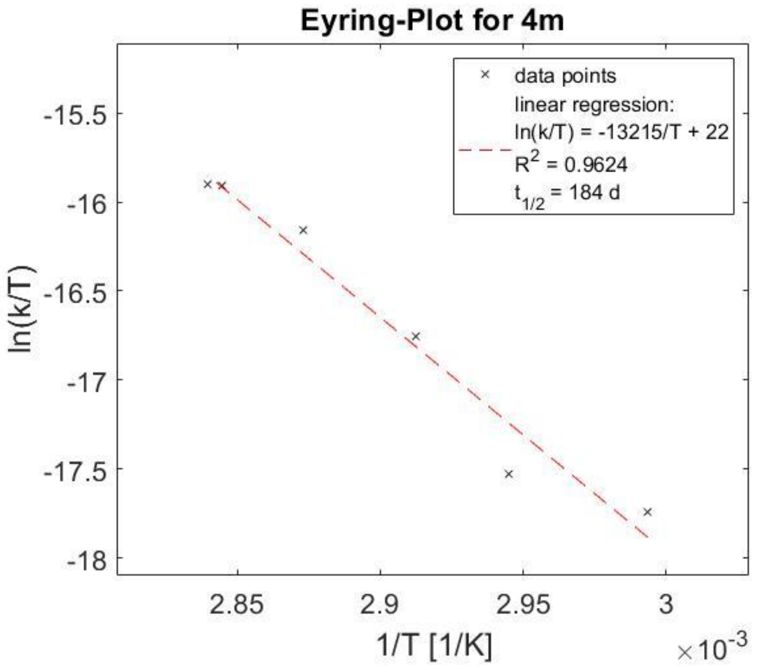

Figure S78: Eyring plot of ln(k/T) against 1/T using the k values obtained for **4m** at different temperatures.

Thermal Relaxation Plot for 4n at 25 °C

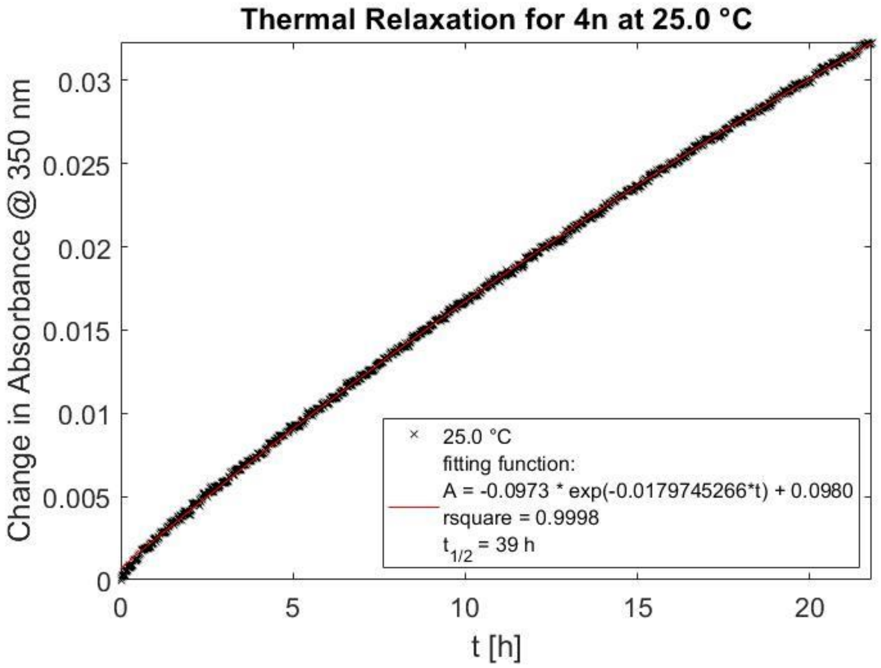

Figure S79: Increase of absorbance at 350 nm and 25.0 °C for a 50 µM solution of **4n** in DMSO after reaching the photostationary state through irradiation at 365 nm measured every 60 s. $t_{1/2}$ is the thermal half-life, calculated from the fitting function assuming a first-order rate law.

Thermal Relaxation Plot for 4o at 25 °C

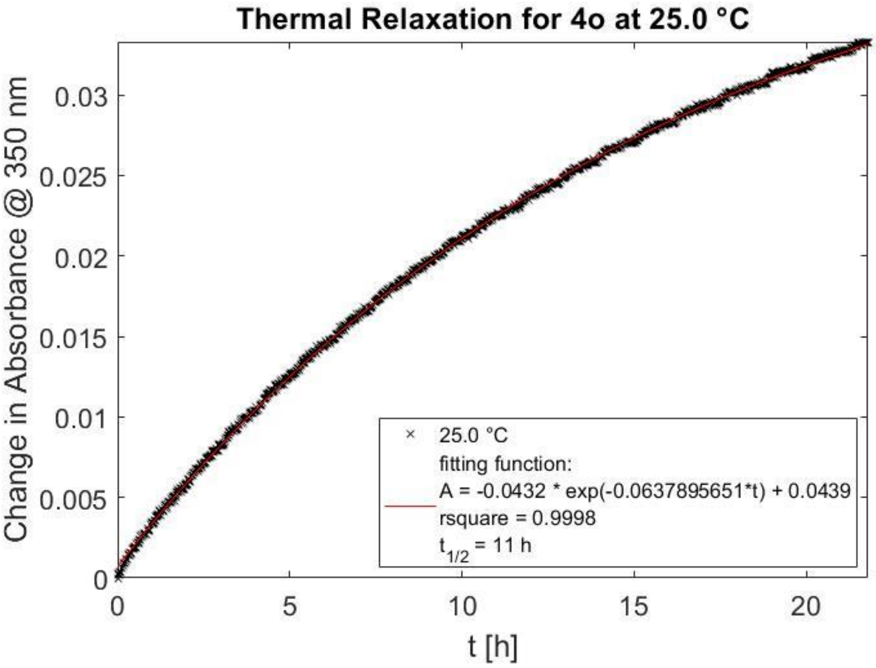

Figure S80: Increase of absorbance at 350 nm and 25.0 °C for a 50 µM solution of **4o** in DMSO after reaching the photostationary state through irradiation at 365 nm measured every 60 s. $t_{1/2}$ is the thermal half-life, calculated from the fitting function assuming a first-order rate law.

Photostability Experiments

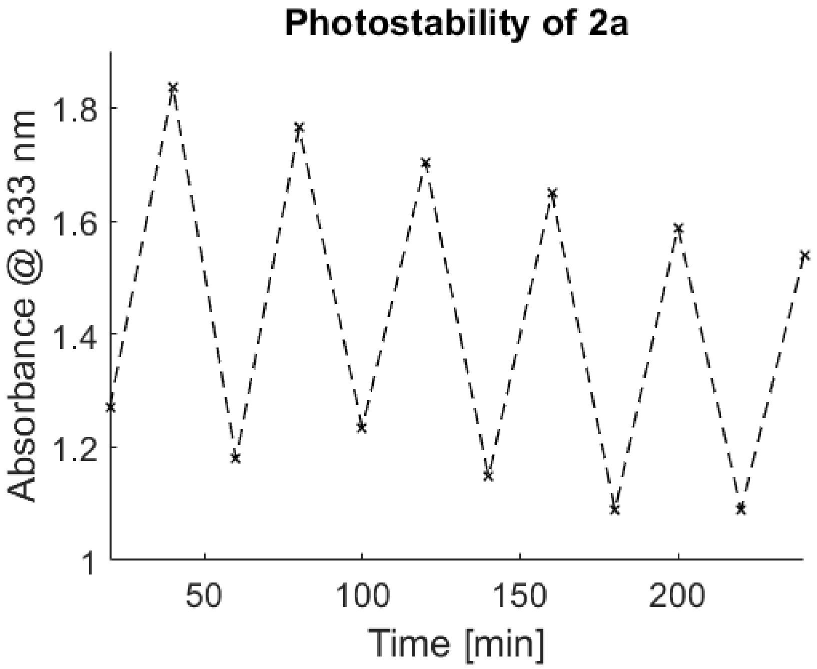

Figure S81: Absorbance at 333 nm for a 100 µM solution of **2a** when alternating irradiation at 365 nm (20 min) and 415 nm (20 min).

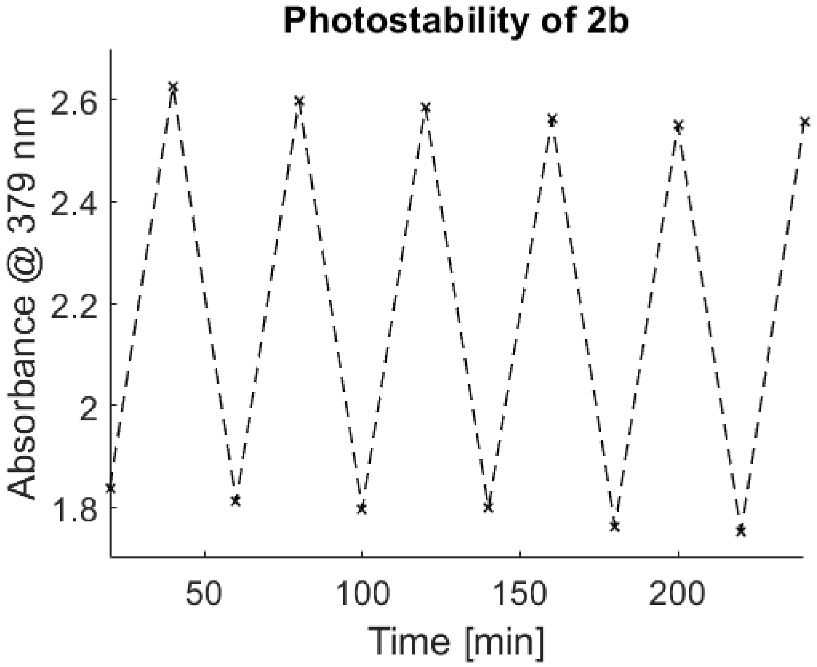

Figure S82: Absorbance at 379 nm for a 100 µM solution of **2b** when alternating irradiation at 415 nm (20 min) and 455 nm (20 min).

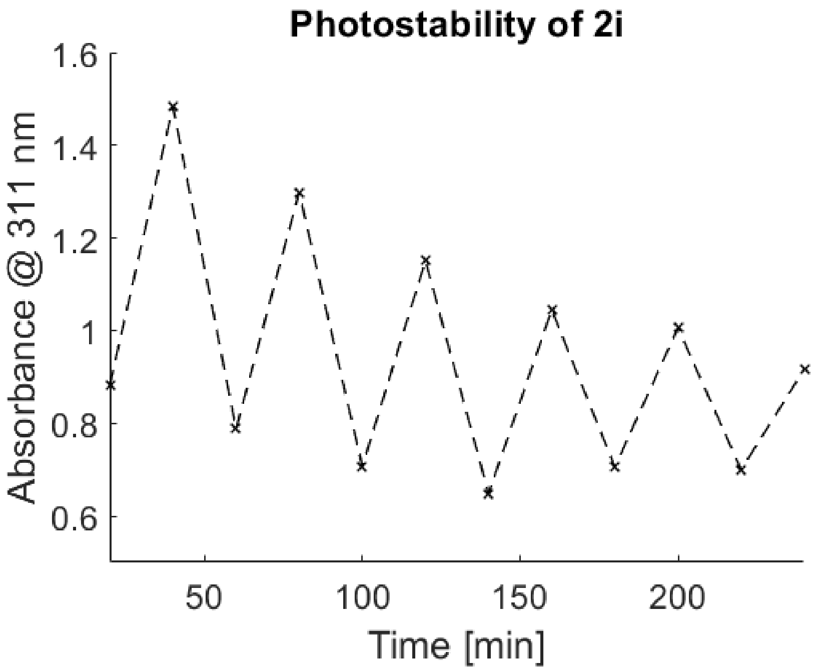

Figure S83: Absorbance at 311 nm for a 100 µM solution of **2i** when alternating irradiation at 365 nm (20 min) and 415 nm (20 min).

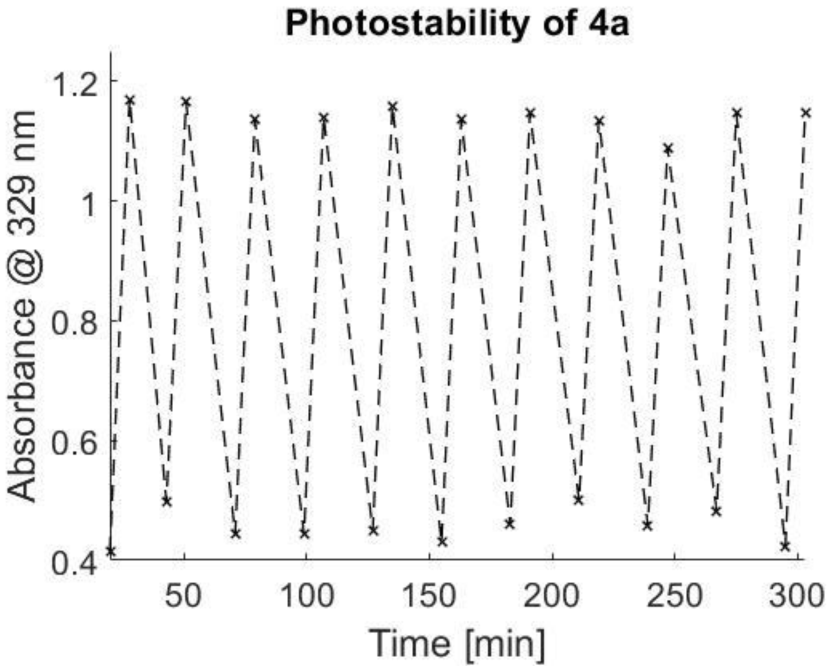

Figure S84: Absorbance at 329 nm for a 100 µM solution of **4a** (DMSO) when alternating irradiation at 365 nm (20 min) and 415 nm (8 min).

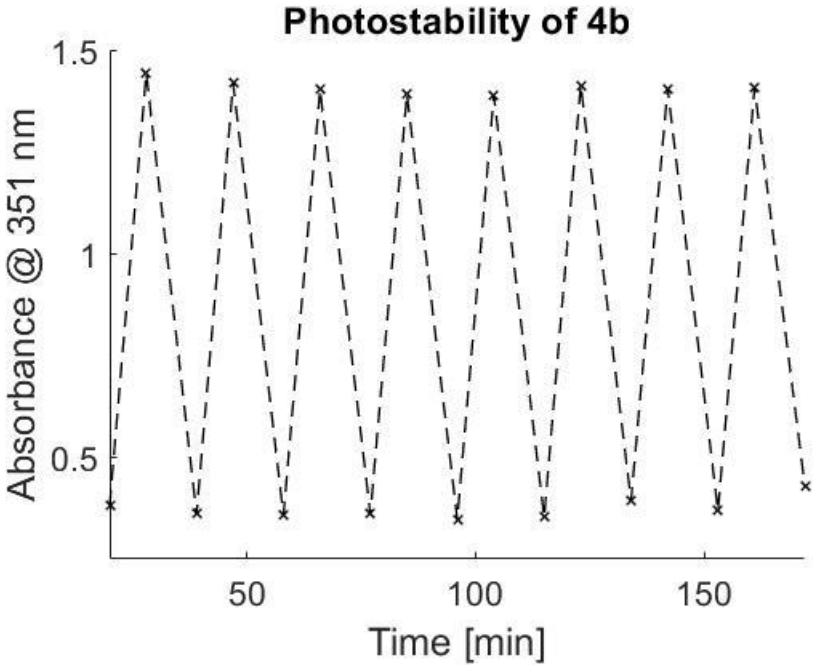

Figure S85: Absorbance at 351 nm for a 100 µM solution of **4b** (DMSO) when alternating irradiation at 365 nm (11 min) and 455 nm (8 min).

Photoisomerisation of PROTAC Conjugate 7b

A stock solution of **7b** was diluted in a 2 mL brown glass GC vial with dry DMSO to give 1 mL of a 100 µM solution. The PSS compositions at 365 nm and 530 nm were determined from the HPLC trace according to **PP1** (the eluent was MeCN/H2O (55:45), containing 0.01% formic acid).

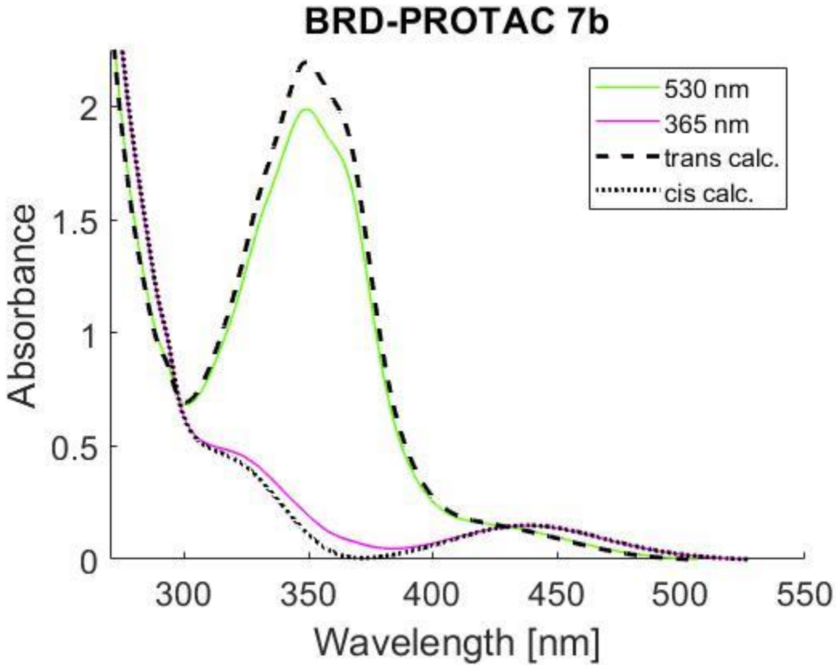

Figure S86: UV-Vis spectra of **7b** in DMSO (100 µM), recorded after irradiation at the stated wavelengths to reach the photostationary state; calculated spectra as determined according to **PP2** are drawn in dashed lines.

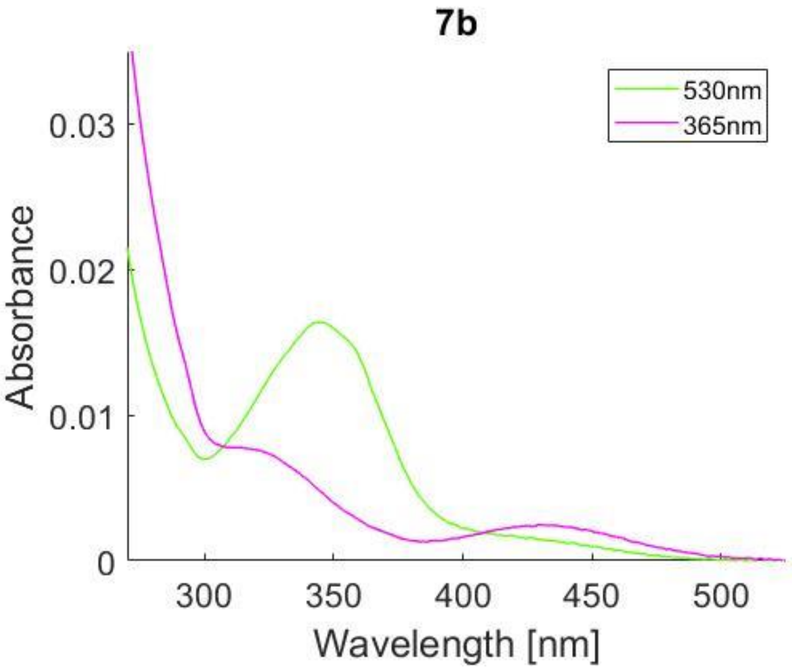

Figure S87: Extracted HPLC-UV-Vis spectra of **7b** in MeCN/H2O (55:45), extracted according to **PP1**, eluted and recorded after irradiation at the stated wavelengths to reach the photostationary state in DMSO (100 μM); isosbestic wavelength in this solvent ratio estimated to be 307 nm.

Additionally, the thermal stability of *Z*-**7b** was investigated by conducting time-course HPLC experiments after irradiation at 365 nm for 20 min while storing the sample in a brown-glass vial at room temperature.

Table T6: PSS compositions for **7b** and evolution over 36h.

	Z	E
**PSS at 365 nm**	**96**	**4**
**after 1h**	96	4
**after 2h**	95	5
**after 3h**	95	5
**after 6h**	94	6
**after 12h**	93	7
**after 24h**	91	9
**after 36h**	88	12
**PSS at 530 nm**	**10**	**90**

Thermal Analysis of Azoacetylenes and Azotriazoles and General Procedures

Thermal Gravimetric Analysis (TGA)

Thermal Analytic Procedure 1: TGA measurements were performed on a TGA 5500 instrument (TA instruments) using 100 µl platinum pans under N_2 atmosphere. Compounds were assessed as neat substances at the given amount. Measurements commenced at 35 °C with a heating rate of 2 °C/min up to 350 °C, followed by a heating rate of 10 °C/min from 350 °C to 600 °C. Data analysis was performed using TRIOS 5.1.1 software available from TA instruments.

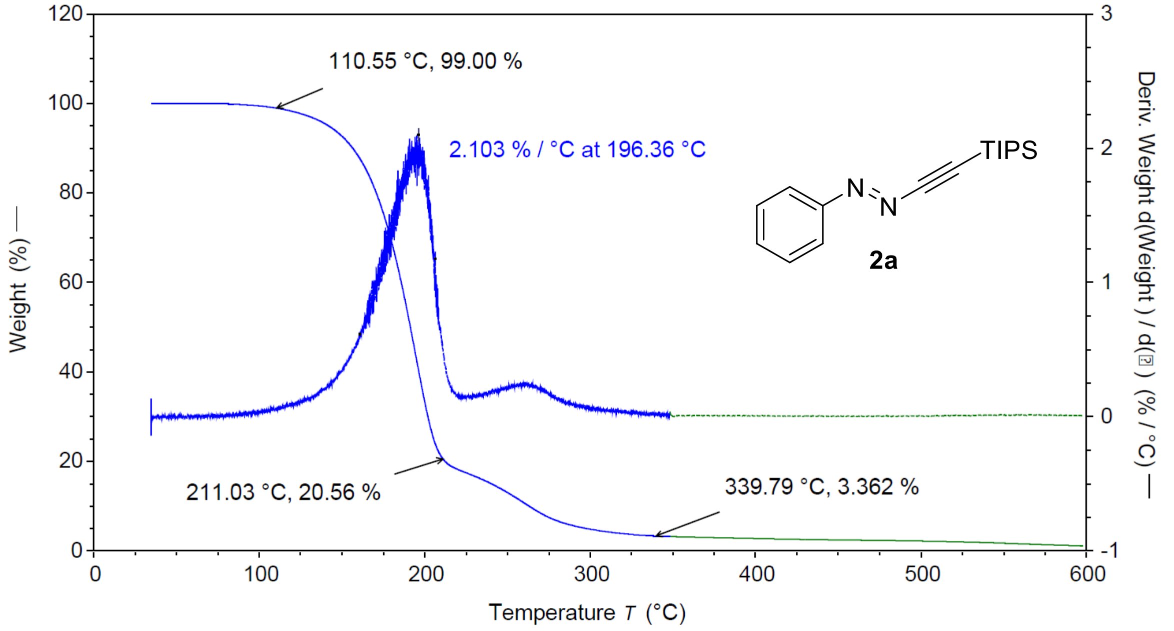

Figure S88: TGA curve of **2a** (oil at rt, 8.218 mg).

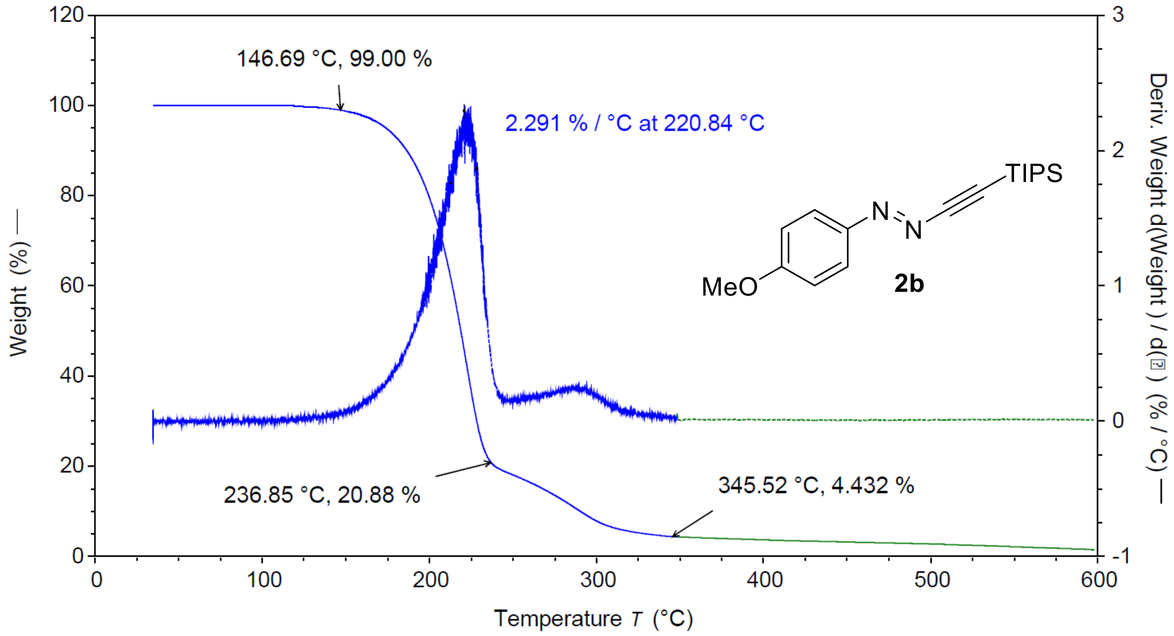

Figure S89: TGA curve of **2b** (oil at rt, 6.799 mg).

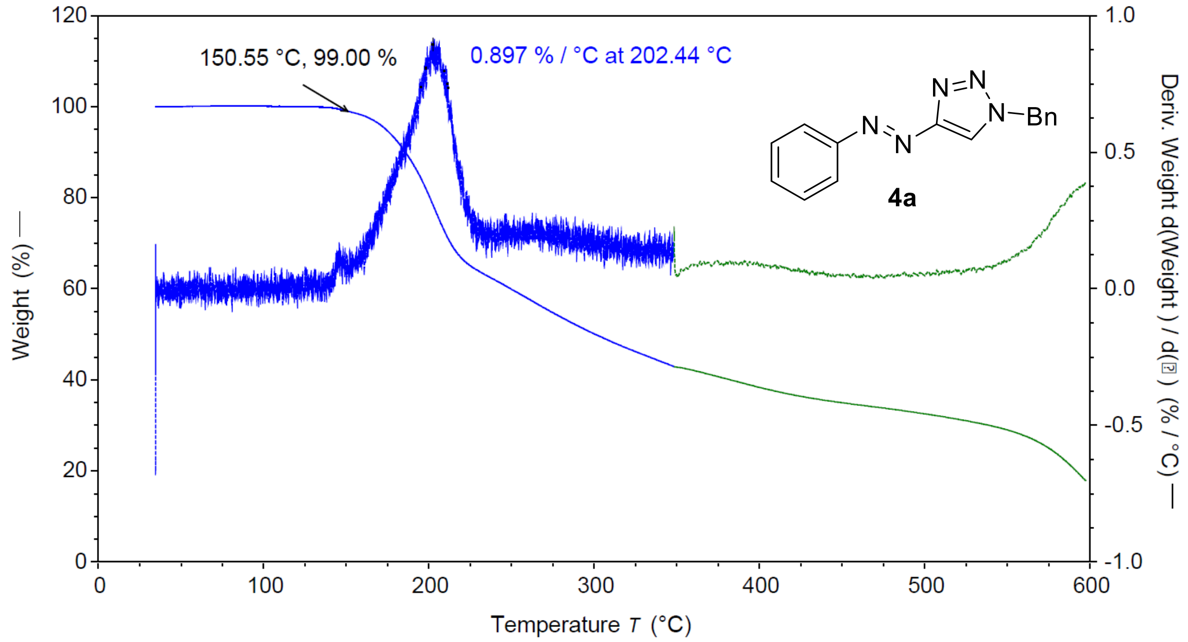

Figure S90: TGA curve of **4a** (solid at rt, 2.353 mg).

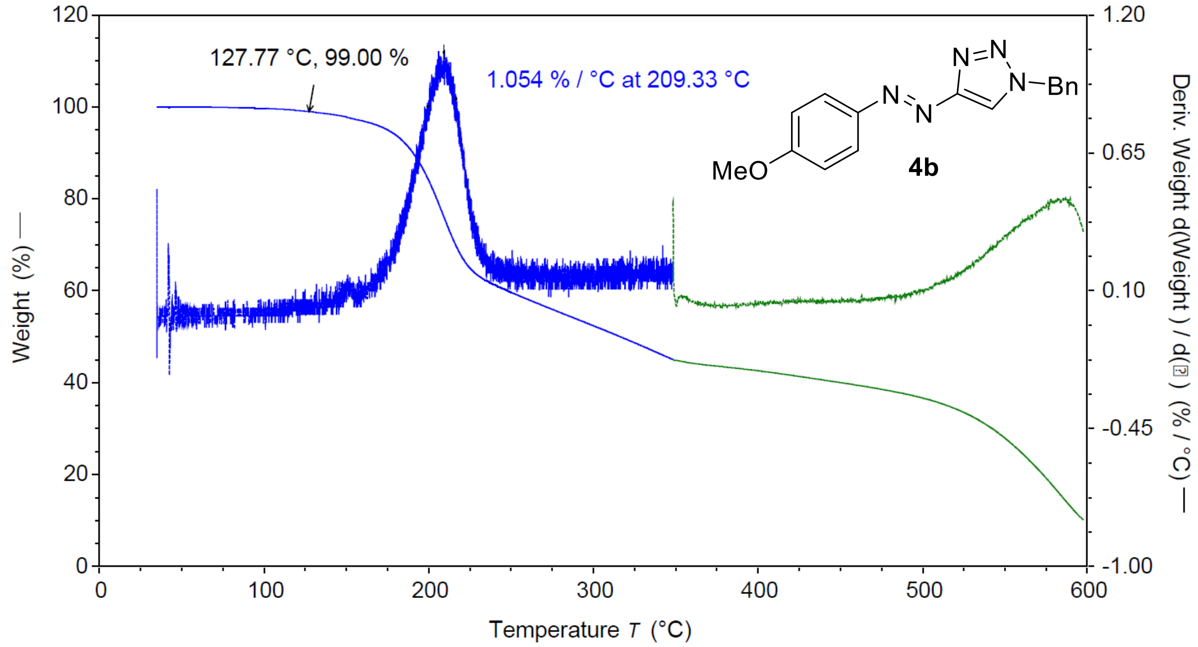

Figure S91: TGA curve of **4b** (solid at rt, 0.940 mg).

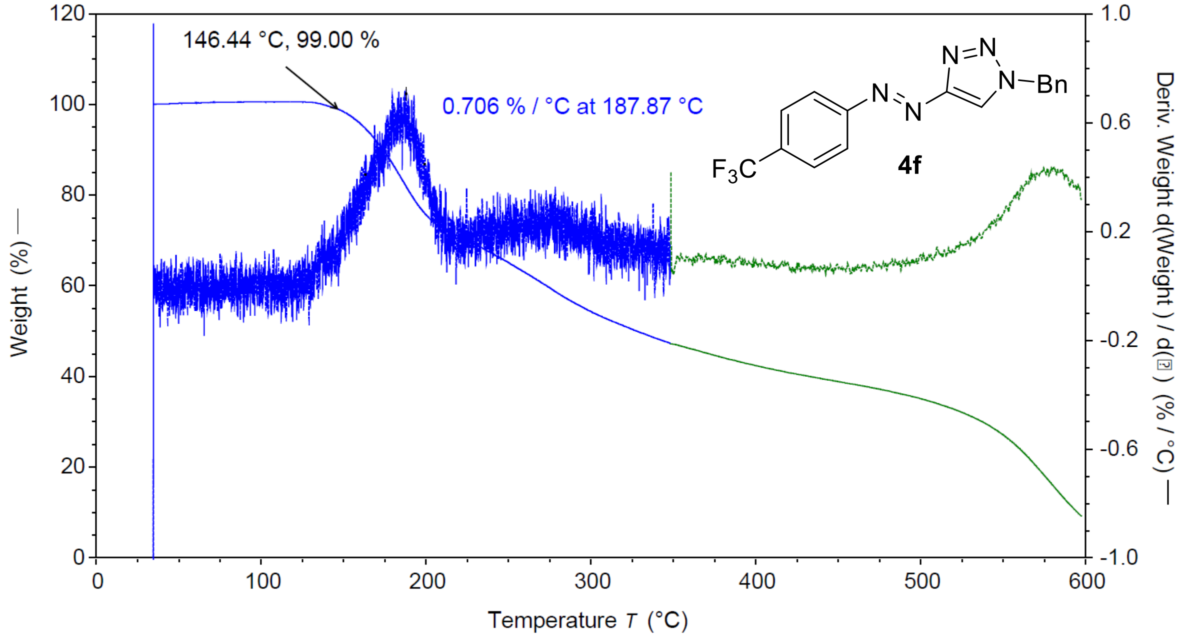

Figure S92: TGA curve of **4f** (solid at rt, 1.101 mg).

Discussion of TGA curves:

Continuous decomposition of **2a** commences at about 111 °C and evolves over a temperature range of more than 200 °C (with a maximum weight differential of 2.1% / °C) before arriving at a stable weight above approximately 340 °C.

Continuous decomposition of **2b** commences at about 147 °C and evolves over a temperature range of about 200 °C (with a maximum weight differential of 2.3% / °C) before arriving at a stable weight above approximately 340 °C.

Continuous decomposition of **4a** commences at about 151 °C and evolves over a temperature range of several hundred °C (with a maximum weight differential of 0.9 % / °C).

Continuous decomposition of **4b** commences at about 128 °C and evolves over a temperature range of several hundred °C (with a maximum weight differential of 1.1 % / °C).

Continuous decomposition of **4f** commences at about 146 °C and evolves over a temperature range of several hundred °C (with a maximum weight differential of 0.7 % / °C).

In contrast to explosive and highly energetic compounds, **2a–b** and **4a–b**, **4f** did not show a rapid loss of weight within a short range of temperature. For reference, energetic 1,1′-azobis-1,2,3-triazole was reported to display a weight loss of 91.75% over a short temperature range of 25 °C.[41]

[41] Li, Y. C.; Qi, C.; Li, S. H.; Zhang, H. J.; Sun, C. H.; Yu, Y. Z.; Pang, S. P. 1,1′-Azobis-1,2,3-Triazole: A High-Nitrogen Compound with Stable N8 Structure and Photochromism. *J. Am. Chem. Soc.* **2010**, 132, 12172–12173.

Dynamic Scanning Calorimetry (DSC)

Thermal Analytic Procedure 2: DSC measurements were performed on a DSC 2500 instrument (TA instruments). Samples were weighed into Tzero Hermetic Pans on a fine balance scale and sealed with Tzero Hermetic Lids. Thermograms were recorded under N_2 atmosphere at a heating rate of 10 °C/min. Thermograms are presented with exotherms going up.

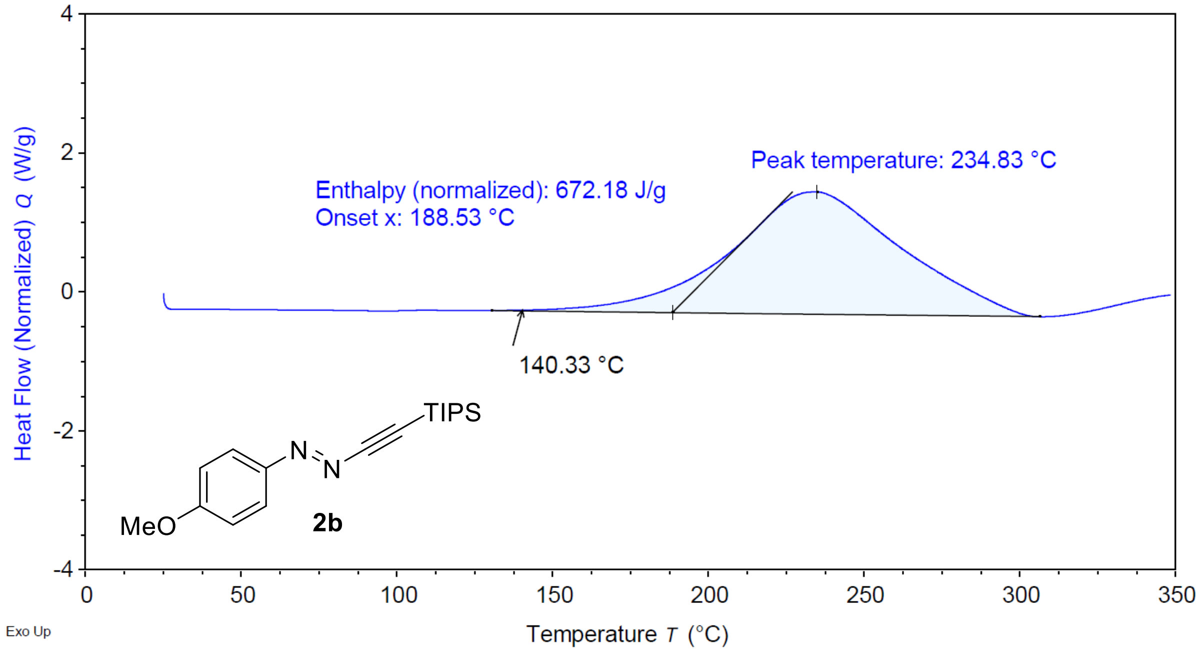

Figure S93: DSC curve of **2b** (oil at rt, 4.772 mg).

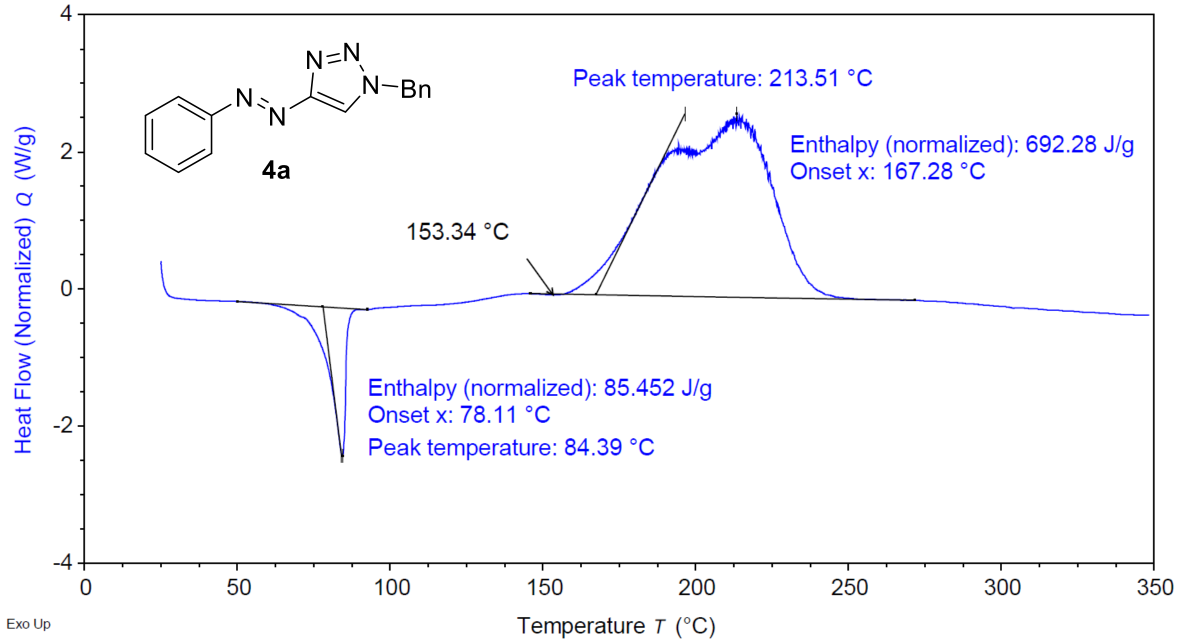

Figure S94: DSC curve of **4a** (solid at rt, 2.346 mg).

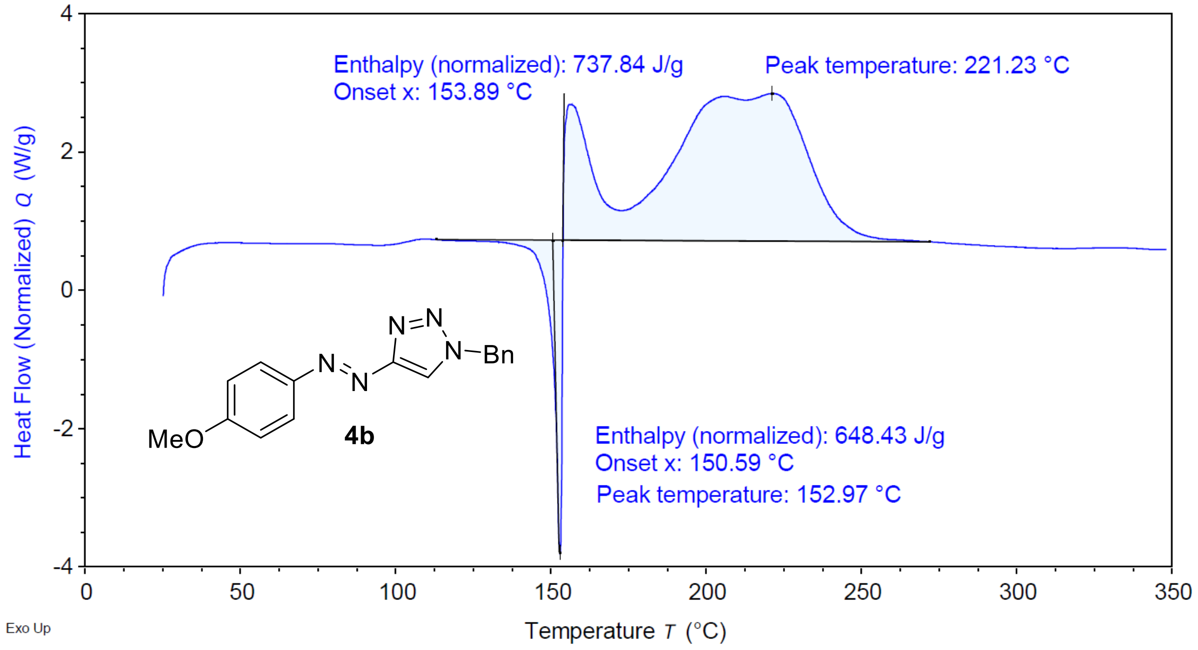

Figure S95: DSC curve of **4b** (solid at rt, 0.567 mg).

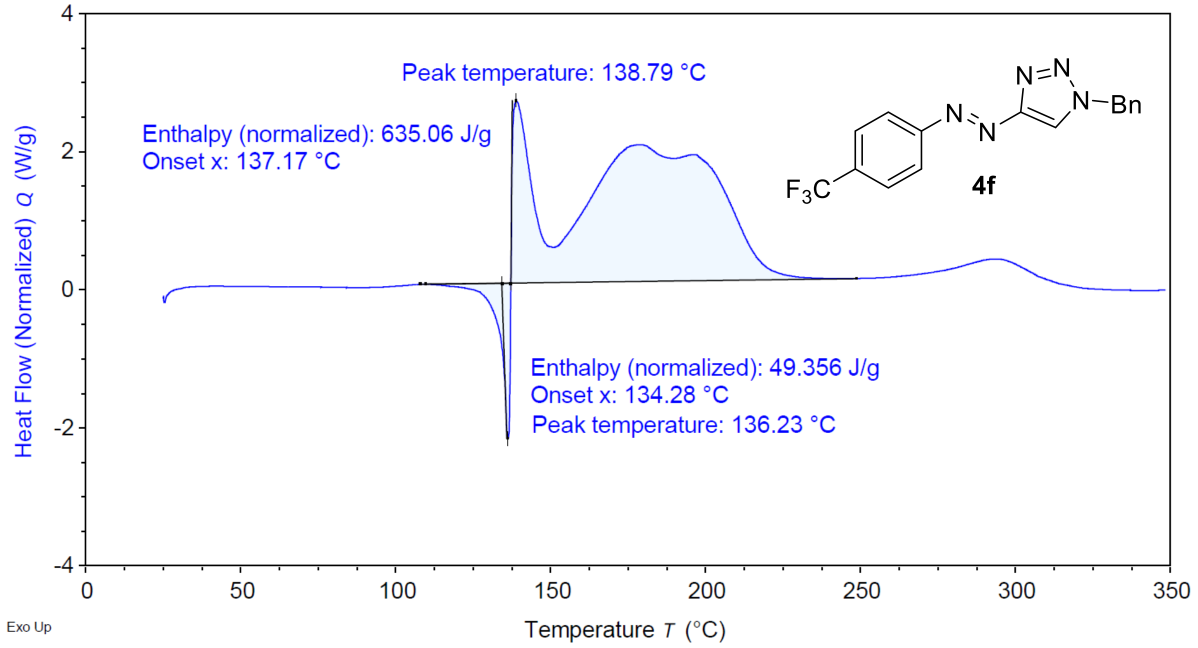

Figure S96: DSC curve of **4f** (solid at rt, 1.372 mg).

Discussion of DSC curves

Decomposition of **2b** above 140 °C is accompanied by an exothermic process which evolves over a temperature range of about 150 °C at a maximum heat flow of 1.44 W/g (peak temperature: 234.8 °C, normalized exothermic enthalpy: 672.2 J/g, onset temperature: 188.5 °C).

Compound **4a** melts at an onset temperature of 78.1 °C (normalized endothermic enthalpy: 85.5 J/g, peak temperature: 84.4 °C). Decomposition above 153 °C is accompanied by an exothermic process which evolves over a temperature range of about 100 °C at a maximum heat flow of 2.56 W/g (peak temperature: 213.5 °C, normalized exothermic enthalpy: 692.3 J/g, onset temperature: 167.3 °C).

Compound **4b** continuously decomposes upon melting at an onset temperature of 150.6 °C (normalized endothermic enthalpy: 648.4 J/g, peak temperature: 153.0 °C). Decomposition above 154 °C is accompanied by an exothermic process which evolves over a temperature range of about 100 °C at a maximum heat flow of 2.85 W/g (peak temperature: 221.2 °C, normalized exothermic enthalpy: 737.8 J/g, onset temperature: 153.9 °C).

Compound **4f** continuously decomposes upon melting at an onset temperature of 134.3 °C (normalized endothermic enthalpy: 49.4 J/g, peak temperature: 136.2 °C). Decomposition above 137 °C is accompanied by an exothermic process which evolves over a temperature range of about 100 °C at a maximum heat flow of 2.75 W/g (peak temperature: 138.8 °C, normalized exothermic enthalpy: 635.1 J/g, onset temperature: 137.2 °C).

In general, compounds **2b**, **4a–b** and **4f** are stable at temperatures well above 100 °C. Exothermic decomposition processes take place over a temperature range of at least 100 °C with maximum heat flows below 3 W/g. This in contrast to DSC profiles of explosive materials which are characterized by a sudden and sharp heat release (heat flows > 30 W/g) within a short temperature range (ranges of 20 to 30 °C).[42]

[42] For reference of characteristic DSC profiles of explosive materials see: (1) Schotten, C.; Leprevost, S. K.; Yong, L. M.; Hughes, C. E.; Harris, K. D. M.; Browne, D. L. Comparison of the Thermal Stabilities of Diazonium Salts and Their Corresponding Triazenes. *Org. Process Res. Dev.* **2020**, 24, 2336–2341. (2) Sperry, J. B.; Stone, S.; Azuma, M.; Barrett, C. Importance of Thermal Stability Data to Avoid Dangerous Reagents: Temozolomide Case Study. *Org. Process Res. Dev.* **2021**, 25, 1690–1700. (3) Li, Y. C.; Qi, C.; Li, S. H.; Zhang, H. J.; Sun, C. H.; Yu, Y. Z.; Pang, S. P. 1,1′-Azobis-1,2,3-Triazole: A High-Nitrogen Compound with Stable N8 Structure and Photochromism. *J. Am. Chem. Soc.* **2010**, 132, 12172–12173.

Yoshida Correlation Analysis

Originally reported by Yoshida and colleagues,[43] the following set of equations have been adopted in the field of Process Safety[44] for the prediction of material properties relating to shock sensitivity ("SS") or explosion propagation ("EP") based on extracted data from DSC measurements:

$$SS = \log(Q_{DSC}) - 0.72\log(T_{DSC} - 25) - 0.98 \quad (1)$$

$$EP = \log(Q_{DSC}) - 0.38\log(T_{DSC} - 25) - 1.67 \quad (2)$$

(with Q_{DSC} = energy of exotherm in cal/g and T_{DSC} = onset temperature of exotherm in °C)

For SS or $EP > 0$, the correlations predict shock sensitivity or explosion propagation of the respective compound. For SS or EP = 0 and with Q_{DSC} in J/g follows:

$$Q_{DSC}\,(SS) = (T_{DSC} - 25)^{0.72} * 10^{0.98} * 4.186 \quad (3)$$

$$Q_{DSC}\,(EP) = (T_{DSC} - 25)^{0.38} * 10^{1.67} * 4.186 \quad (4)$$

Plotting Q_{DSC} against T_{DSC} generates a correlation curve (see below) which predicts the potential for shock sensitivity or explosion propagation – the prediction is positive if the materials is located above and negative if located below the respective curve. Pfizer and other pharmaceutical companies have established modified, more conservative correlations with a 25% lower energy threshold. Also, instead of extrapolated onset temperatures, these modified correlations take left-limit onset temperatures into consideration. From this follows

(Pfizer-modified) $$Q_{DSC}\,(SS) = \left(T_{left\,DSC} - 25\right)^{0.72} * 10^{0.98} * 4.186 * 0.75 \quad (5)$$

(Pfizer-modified) $$Q_{DSC}\,(EP) = (T_{left\,DSC} - 25)^{0.38} * 10^{1.67} * 4.186 * 0.75 \quad (6)$$

---

[43] (1) Yoshida, T.; Yoshizawa, F.; Itoh, M.; Matsunaga, T.; Watanabe, M.; Tamura, M. Prediction of fire and explosion hazards of reactive chemicals. I. Estimation of explosive properties of self-reactive chemicals from SC-DSC data. *Kogyo Kayaku* **1987**, 5, 311−316. (2) Yoshida, T.; Wada, Y.; Foster, N. Safety of Reactive Chemicals and Pyrotechnics; Elsevier Science, 1995 (Volume 5). Chapter 3 "Experimental evaluation of fire and explosion hazards of reactive substances".

[44] For reference of recent applications of Yoshida correlations and conservative modifications for industrial analysis see: (1) Sperry, J. B.; Minteer, C. J.; Tao, J.; Johnson, R.; Duzguner, R.; Hawksworth, M.; Oke, S.; Richardson, P. F.; Barnhart, R.; Bill, D. R.; Giusto, R. A.; Weaver, J. D. Thermal Stability Assessment of Peptide Coupling Reagents Commonly Used in Pharmaceutical Manufacturing. *Org. Process Res. Dev.* **2018**, 22, 1262–1275. (2) Green, S. P.; Wheelhouse, K. M.; Payne, A. D.; Hallett, J. P.; Miller, P. W.; Bull, J. A. Thermal Stability and Explosive Hazard Assessment of Diazo Compounds and Diazo Transfer Reagents. *Org. Process Res. Dev.* **2020**, 24, 67–84. (3) Sperry, J. B.; Stone, S.; Azuma, M.; Barrett, C. Importance of Thermal Stability Data to Avoid Dangerous Reagents: Temozolomide Case Study. *Org. Process Res. Dev.* **2021**, 25, 1690–1700.

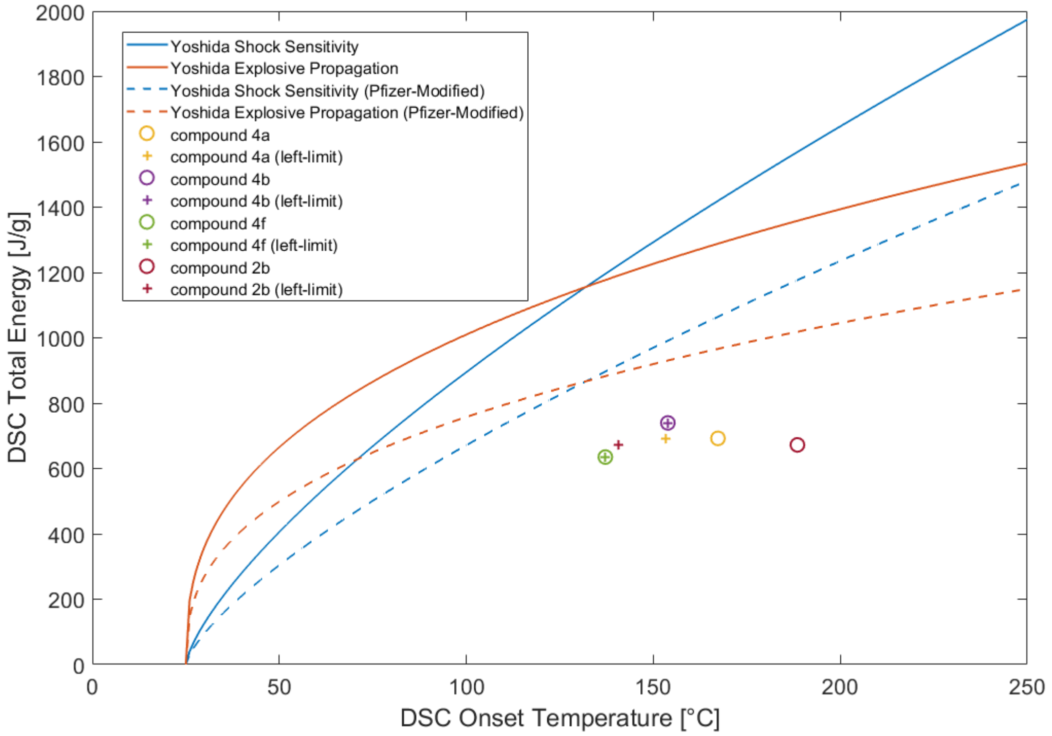

Figure **S97**: Yoshida correlation plot for DSC data of compounds **2b**, **4a–b**, **4f**.

As can be seen in figure **S97** all compounds examined were located well below both original and conservatively modified Yoshida correlation curves. Based on this data and the representative set of compounds, this indicates no expectation of shock sensitivity or explosive behavior for arylazoacetylenes and arylazotriazoles as prepared in this manuscript.

Single Crystal X-Ray Data

Single crystals of compound **2m, 2n, 4a** and **4b** were obtained by storing saturated solutions in MeOH at 4 °C for several hours (overnight), followed by resting at room temperature. Samples were protected from daylight by performing this in amberized vials. CCDC 2088782 (**2m**), CCDC 2088788 (**2n**), CCDC 2088787 (**4a**), CCDC 2088785 (**4b**) contain the supplementary crystallographic data for this paper. These data are provided free of charge by The Cambridge Crystallographic Data Centre via www.ccdc.cam.ac.uk/data_request/cif.

Crystal Structure Data of 2m (CCDC 2088782).

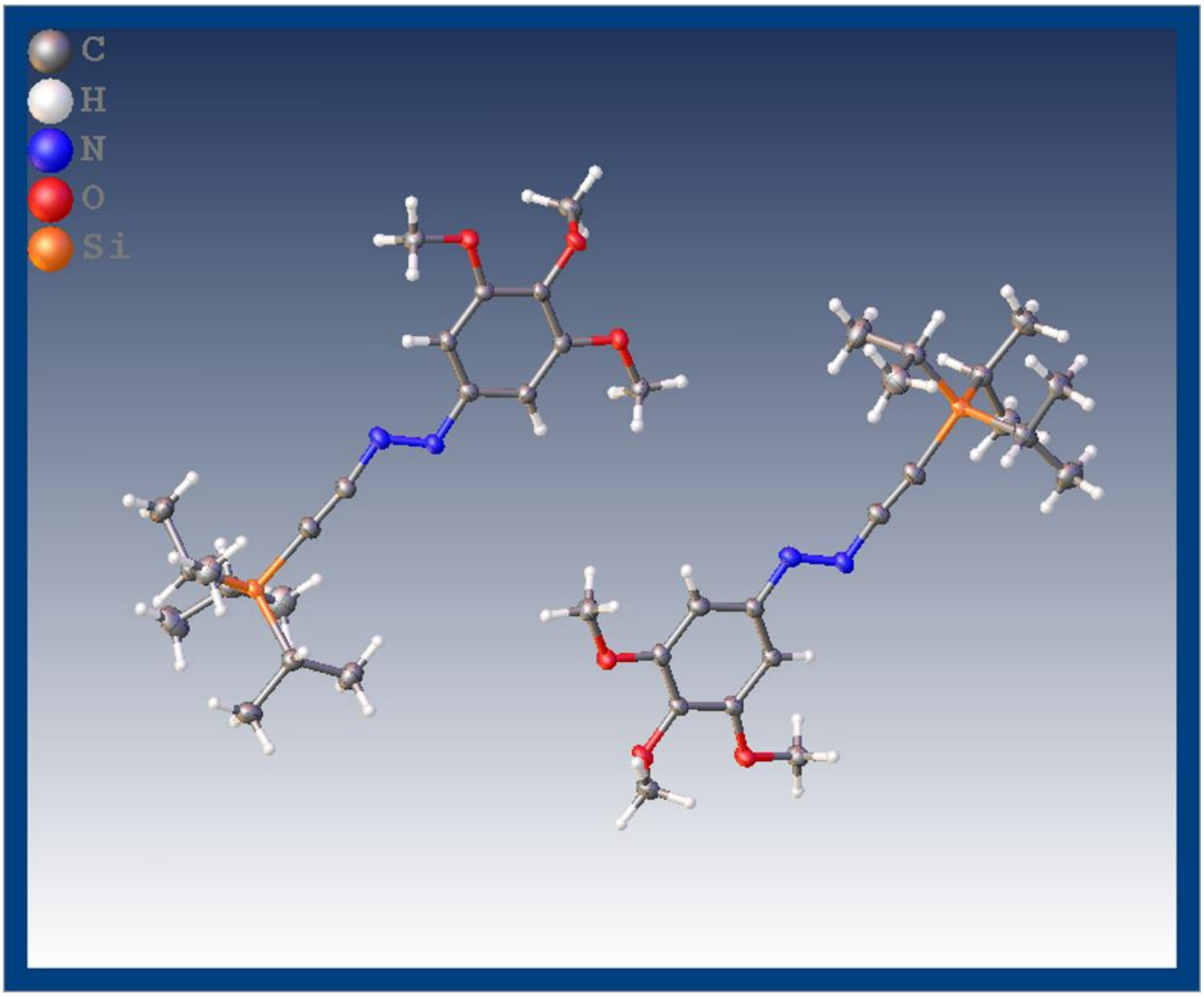

Table T7: Crystal data and structure refinement for 2m (CCDC 2088782).

Identification code	ca060421_1_1
Empirical formula	C20H32N2O3Si
Formula weight	376.56
Temperature/K	100.0(1)
Crystal system	triclinic
Space group	P-1
a/Å	9.1886(4)
b/Å	13.0054(2)
c/Å	18.1434(4)

α/°	90.172(2)
β/°	92.620(2)
γ/°	93.916(2)
Volume/Å3	2160.80(11)
Z	4
P_{calc} g/cm3	1.158
μ/mm-1	1.119
F(000)	816
Crystal size/mm3	0.265 × 0.181 × 0.021
Radiation	Cu Kα (λ = 1.54184)
2Θ range for data collection/°	4.876 to 160.726
Index ranges	-11 ≤ h ≤ 11, -15 ≤ k ≤ 15, -23 ≤ l ≤ 23
Reflections collected	9195
Independent reflections	9195 [Rint = 0.0670, Rsigma = 0.0312]
Data/restraints/parameters	9195/0/488
Goodness-of-fit on F2	1.154
Final R indexes [I>=2σ (I)]	R1 = 0.0714, wR2 = 0.2012
Final R indexes [all data]	R1 = 0.0793, wR2 = 0.2048
Largest diff. peak/hole / e Å-3	0.47/-0.42

Crystal Structure Data of 2n (CCDC 2088788).

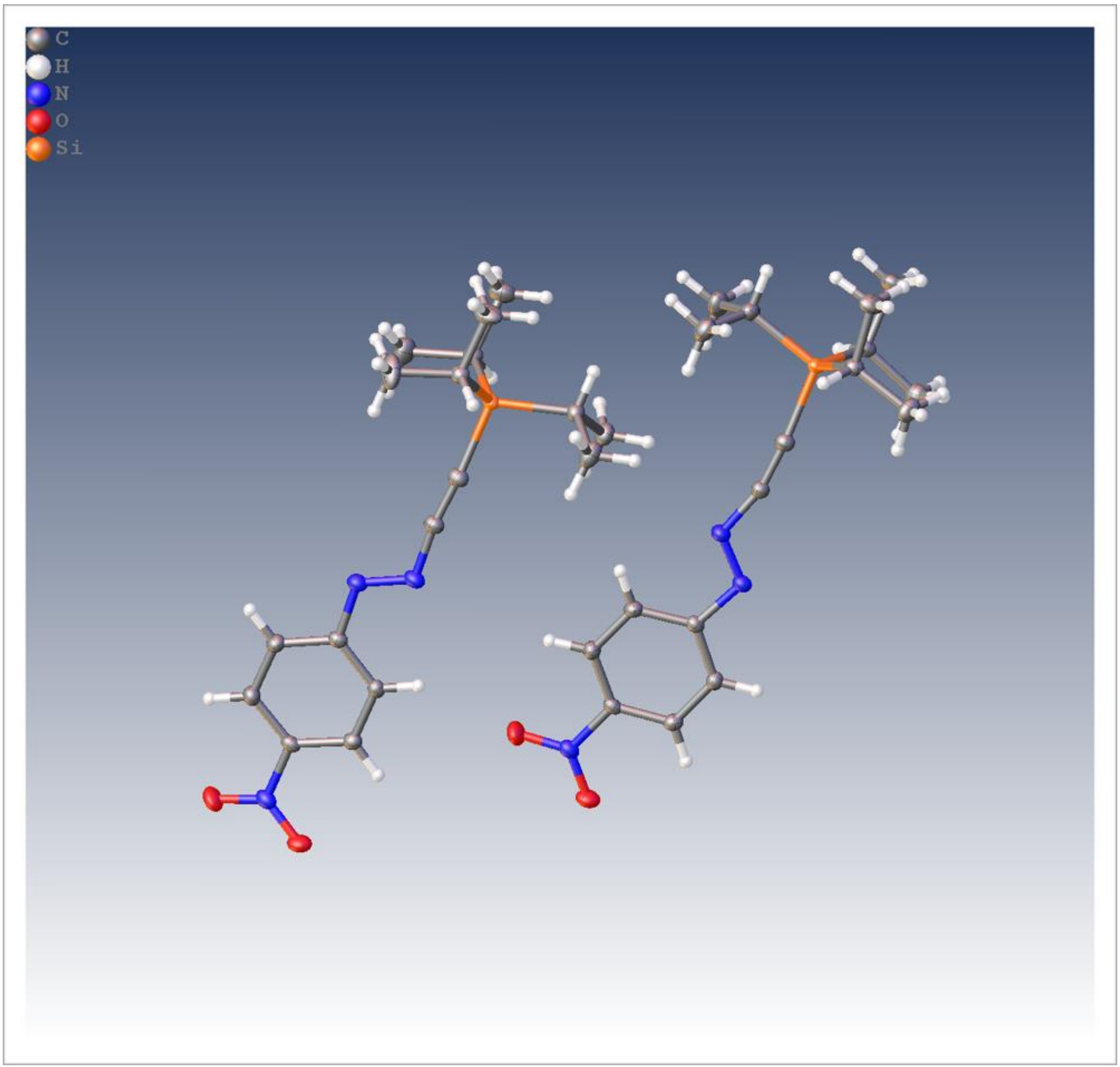

Table T8: Crystal data and structure refinement for 2n (CCDC 2088788).

Identification code	ca280421_1_1
Empirical formula	$C_{17}H_{25}N_3O_2Si$
Formula weight	331.49
Temperature/K	100.0(1)
Crystal system	triclinic
Space group	P-1
a/Å	9.52790(10)

b/Å	10.8085(2)
c/Å	17.9688(3)
α/°	82.4110(10)
β/°	81.0110(10)
γ/°	88.0080(10)
Volume/Å3	1811.54(5)
Z	4
P_{calc} g/cm3	1.215
μ/mm-1	1.246
F(000)	712
Crystal size/mm3	0.273 × 0.185 × 0.068
Radiation	Cu Kα (λ = 1.54184)
2Θ range for data collection/°	5.02 to 159.58
Index ranges	-12 ≤ h ≤ 12, -13 ≤ k ≤ 12, -22 ≤ l ≤ 22
Reflections collected	50141
Independent reflections	7662 [R_{int} = 0.0402, R_{sigma} = 0.0234]
Data/restraints/parameters	7662/0/427
Goodness-of-fit on F2	1.072
Final R indexes [I>=2σ (I)]	R_1 = 0.0333, wR_2 = 0.0885
Final R indexes [all data]	R_1 = 0.0369, wR_2 = 0.0907
Largest diff. peak/hole / e Å-3	0.39/-0.28

Crystal Structure Data of 4a (CCDC 2088787).

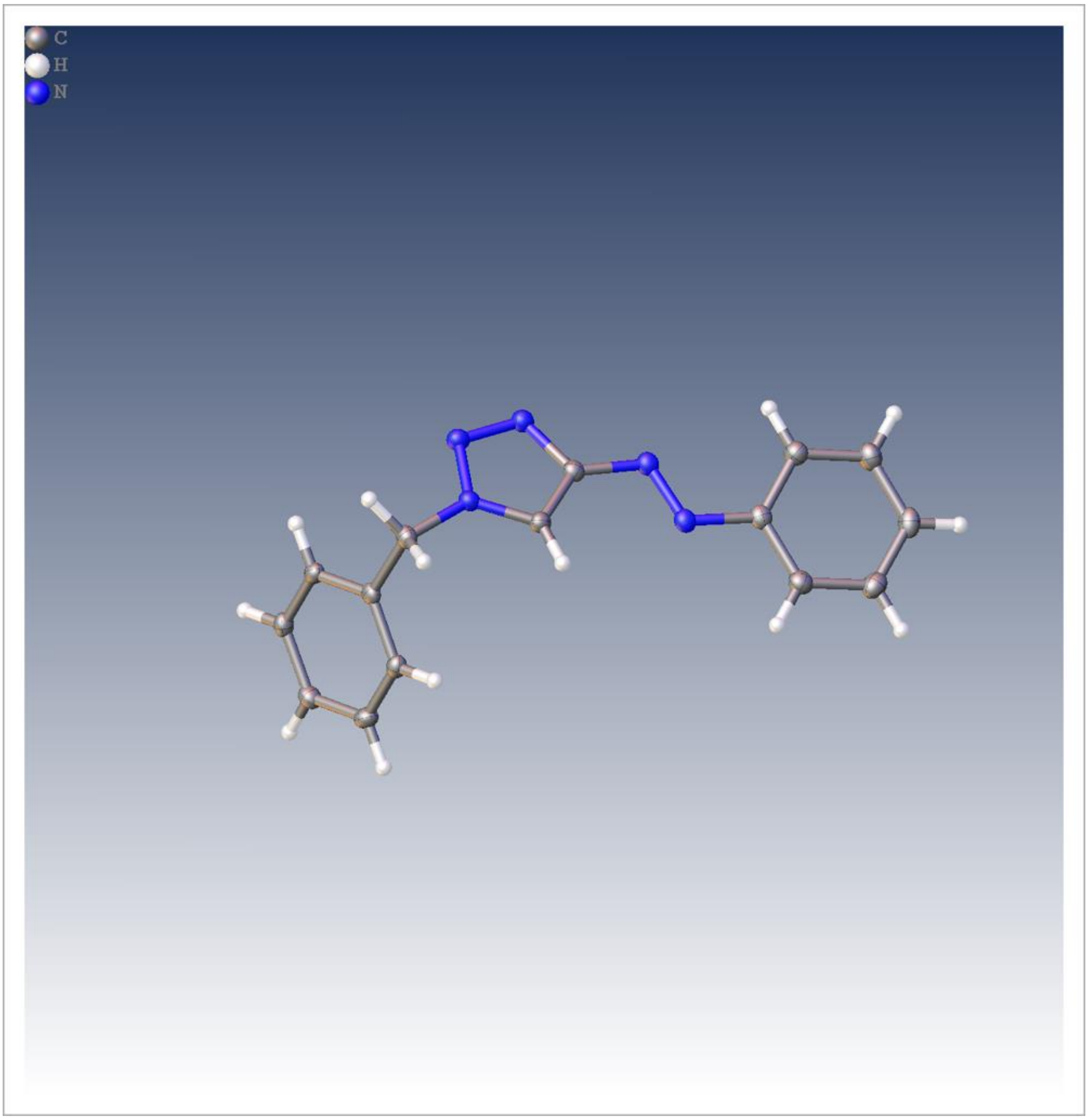

Table T9: Crystal data and structure refinement for 4a (CCDC 2088787).

Identification code	ca051220_1_1
Empirical formula	$C_{15}H_{13}N_5$
Formula weight	263.3
Temperature/K	100.0(1)
Crystal system	monoclinic
Space group	$P2_1/c$

a/Å	12.83951(12)
b/Å	5.52878(4)
c/Å	18.30998(16)
α/°	90
β/°	96.4658(8)
γ/°	90
Volume/Å3	1291.499(19)
Z	4
P_{calc} g/cm3	1.354
μ/mm-1	0.688
F(000)	552
Crystal size/mm3	0.155 × 0.085 × 0.08
Radiation	Cu Kα (λ = 1.54184)
2Θ range for data collection/°	6.928 to 159.806
Index ranges	-15 ≤ h ≤ 16, -7 ≤ k ≤ 6, -23 ≤ l ≤ 23
Reflections collected	30835
Independent reflections	2760 [R_{int} = 0.0366, R_{sigma} = 0.0158]
Data/restraints/parameters	2760/0/181
Goodness-of-fit on F2	1.052
Final R indexes [I>=2σ (I)]	R_1 = 0.0337, wR_2 = 0.0861
Final R indexes [all data]	R_1 = 0.0353, wR_2 = 0.0872
Largest diff. peak/hole / e Å-3	0.26/-0.26

Crystal Structure Data of 4b (CCDC 2088785).

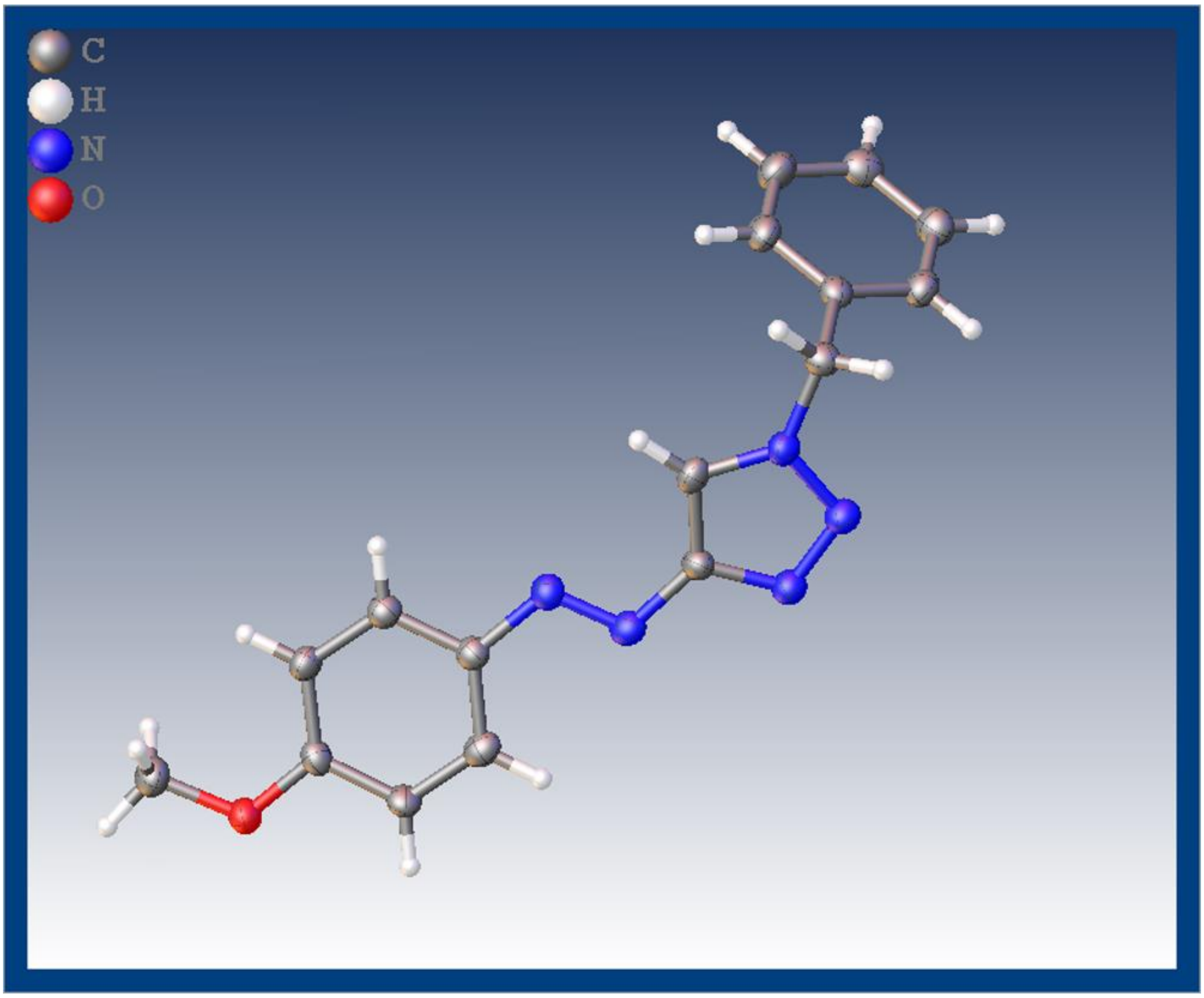

Table T10: Crystal data and structure refinement for 4a (CCDC 2088785).

Identification code	ca270521_1_1
Empirical formula	$C_{16}H_{15}N_5O$
Formula weight	293.33
Temperature/K	100.0(1)
Crystal system	monoclinic
Space group	$P2_1$
a/Å	7.9736(5)
b/Å	5.5716(3)
c/Å	16.4500(9)

α/°	90
β/°	95.015(5)
γ/°	90
Volume/Å3	728.01(7)
Z	2
P_{calc} g/cm3	1.338
μ/mm-1	0.719
F(000)	308
Crystal size/mm3	0.209 × 0.121 × 0.018
Radiation	Cu Kα (λ = 1.54184)
2Θ range for data collection/°	5.392 to 164.268
Index ranges	-8 ≤ h ≤ 9, -7 ≤ k ≤ 7, -20 ≤ l ≤ 21
Reflections collected	13165
Independent reflections	2995 [R_{int} = 0.0706, R_{sigma} = 0.0479]
Data/restraints/parameters	2995/1/200
Goodness-of-fit on F2	1.133
Final R indexes [I>=2σ (I)]	R_1 = 0.0563, wR_2 = 0.1431
Final R indexes [all data]	R_1 = 0.0713, wR_2 = 0.1687
Largest diff. peak/hole / e Å-3	0.33/-0.40
Flack parameter	-0.3(3)

NMR spectra

^{1}H NMR, ^{13}C NMR and ^{19}F NMR spectrum of compound **1a**

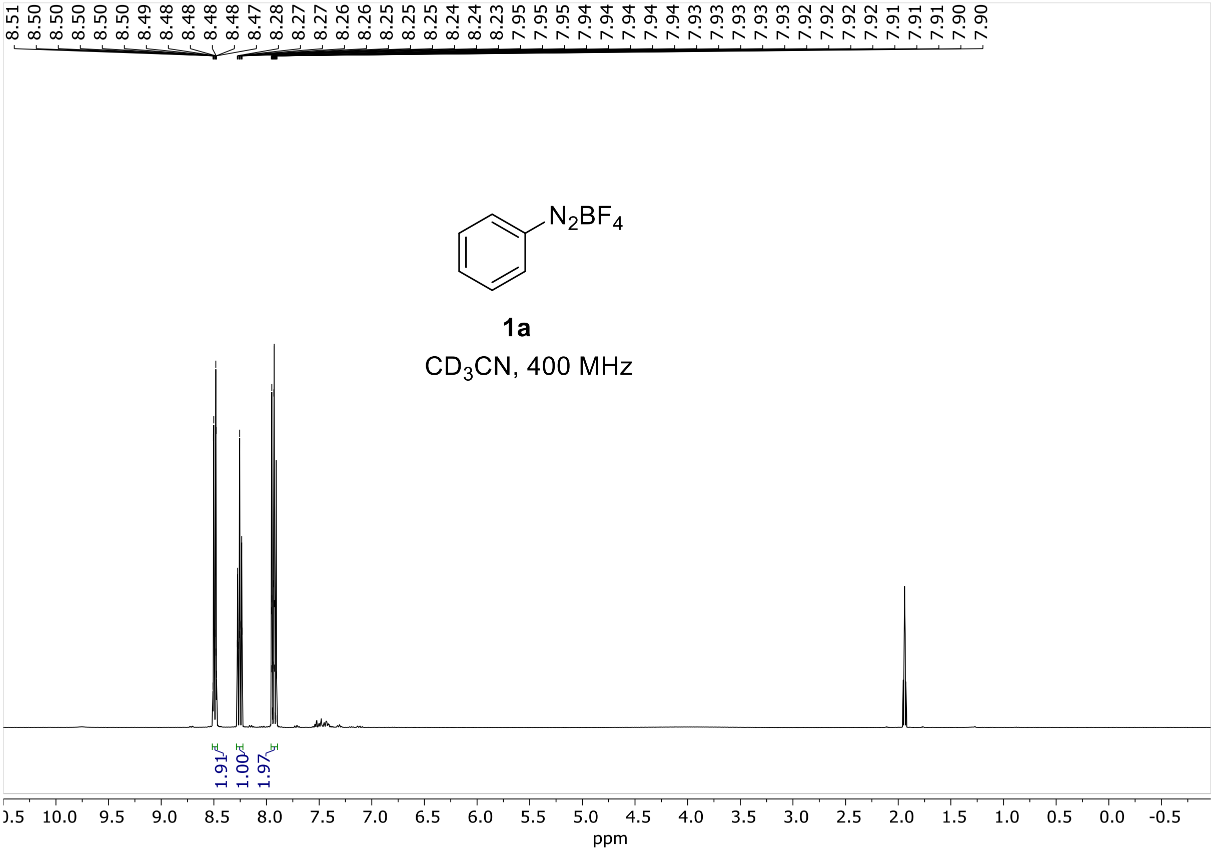

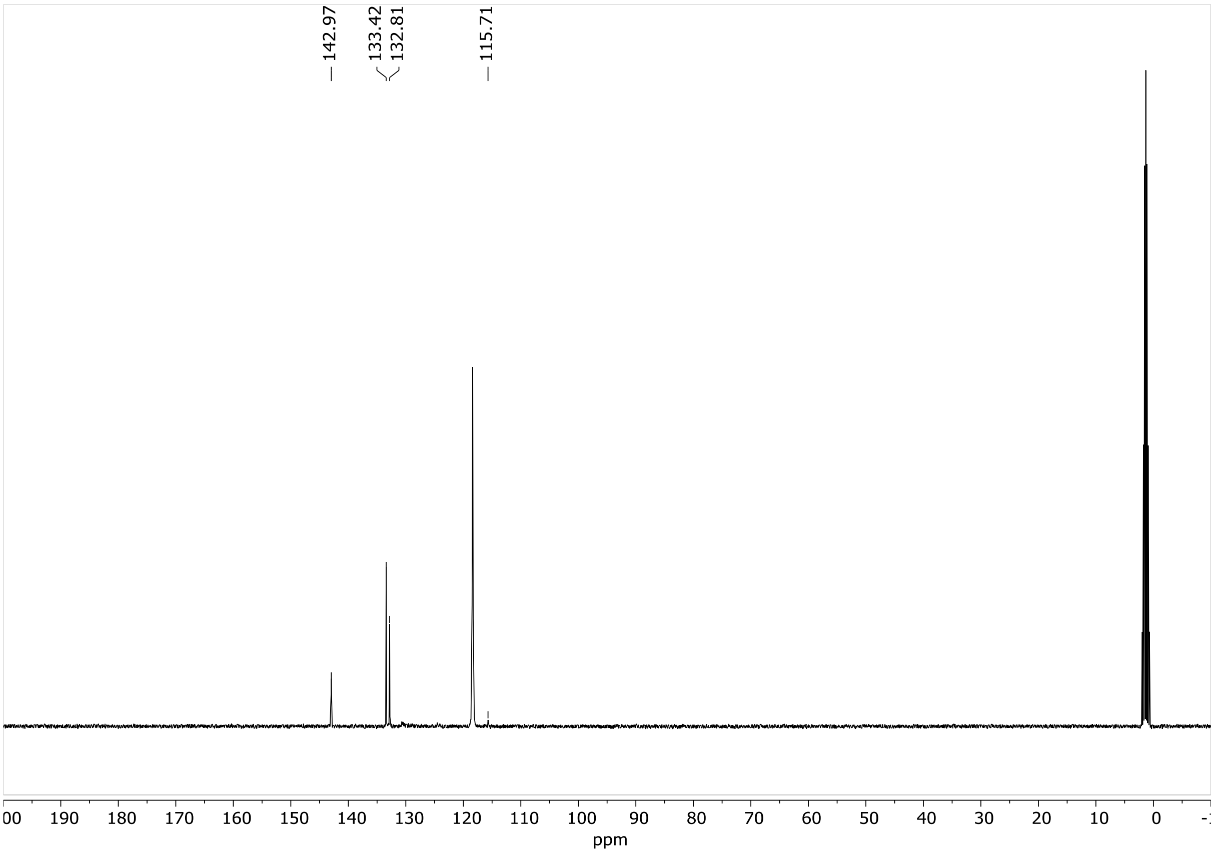

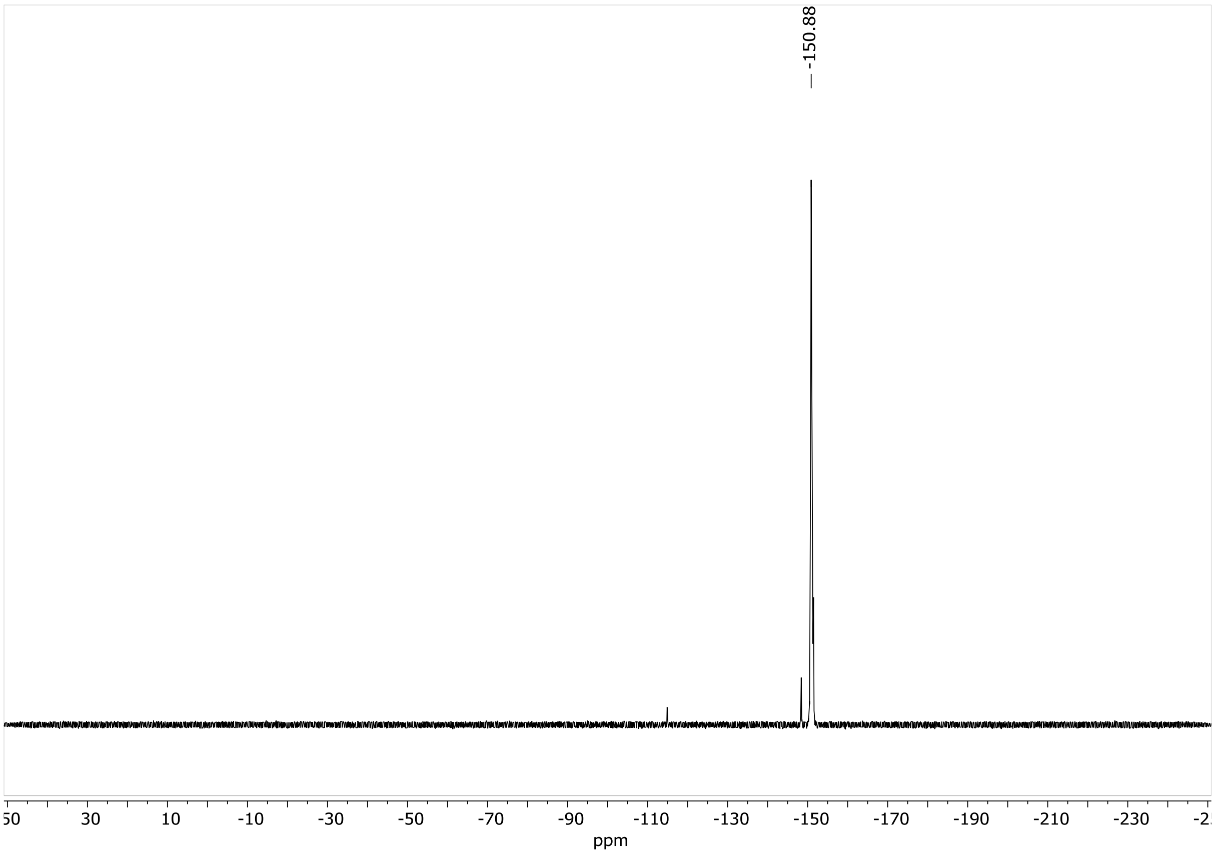
-150.88
50
30
10
-10
-30
-50
-70
-90
-110
-130
-150
-170
-190
-210
-230
ppm

^{1}H NMR, ^{13}C NMR and ^{19}F NMR spectrum of compound **1b**

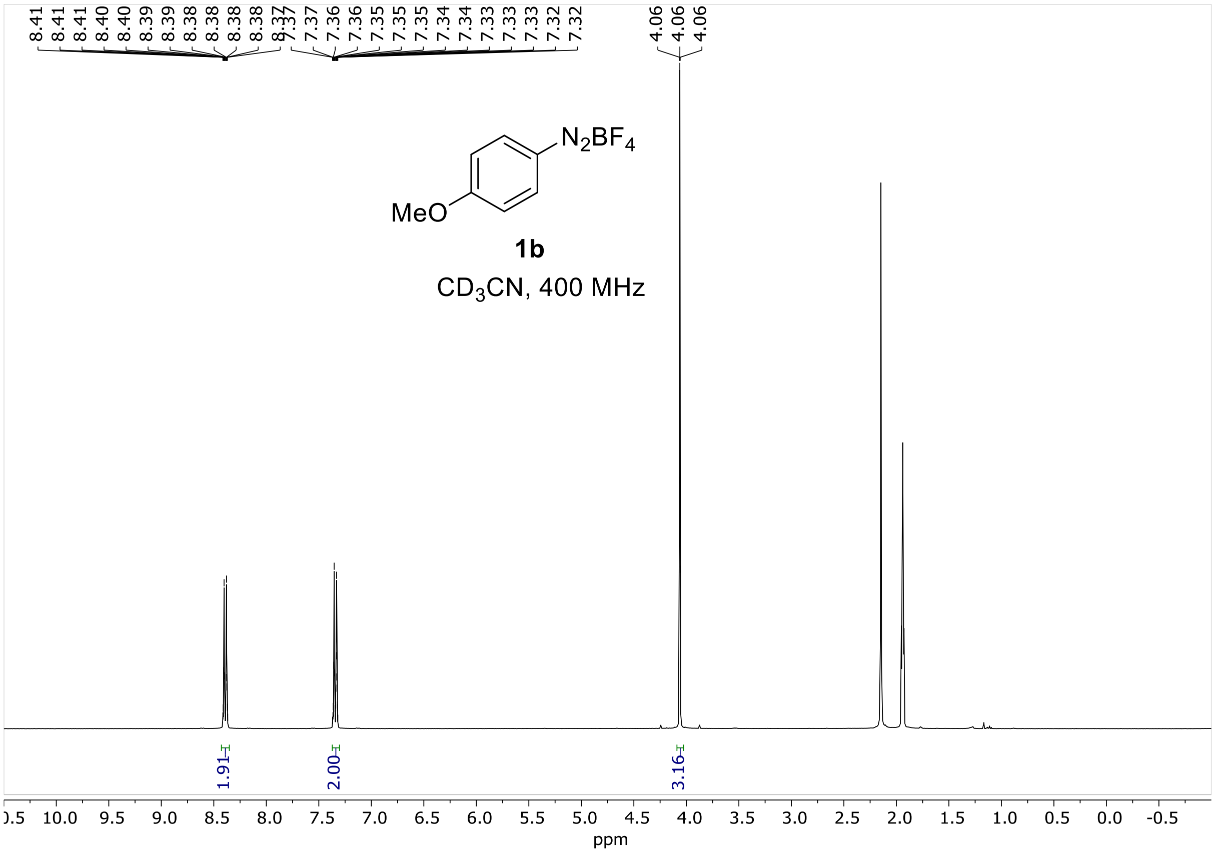

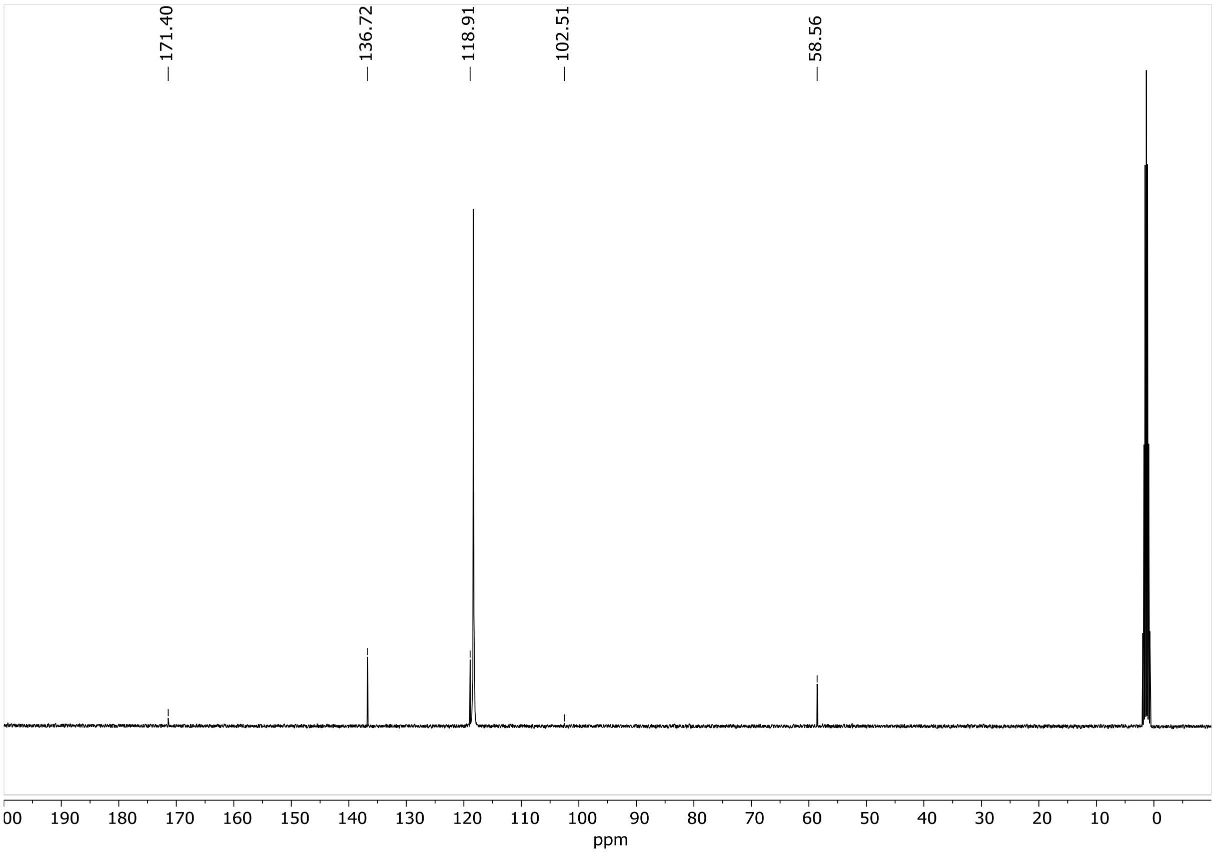

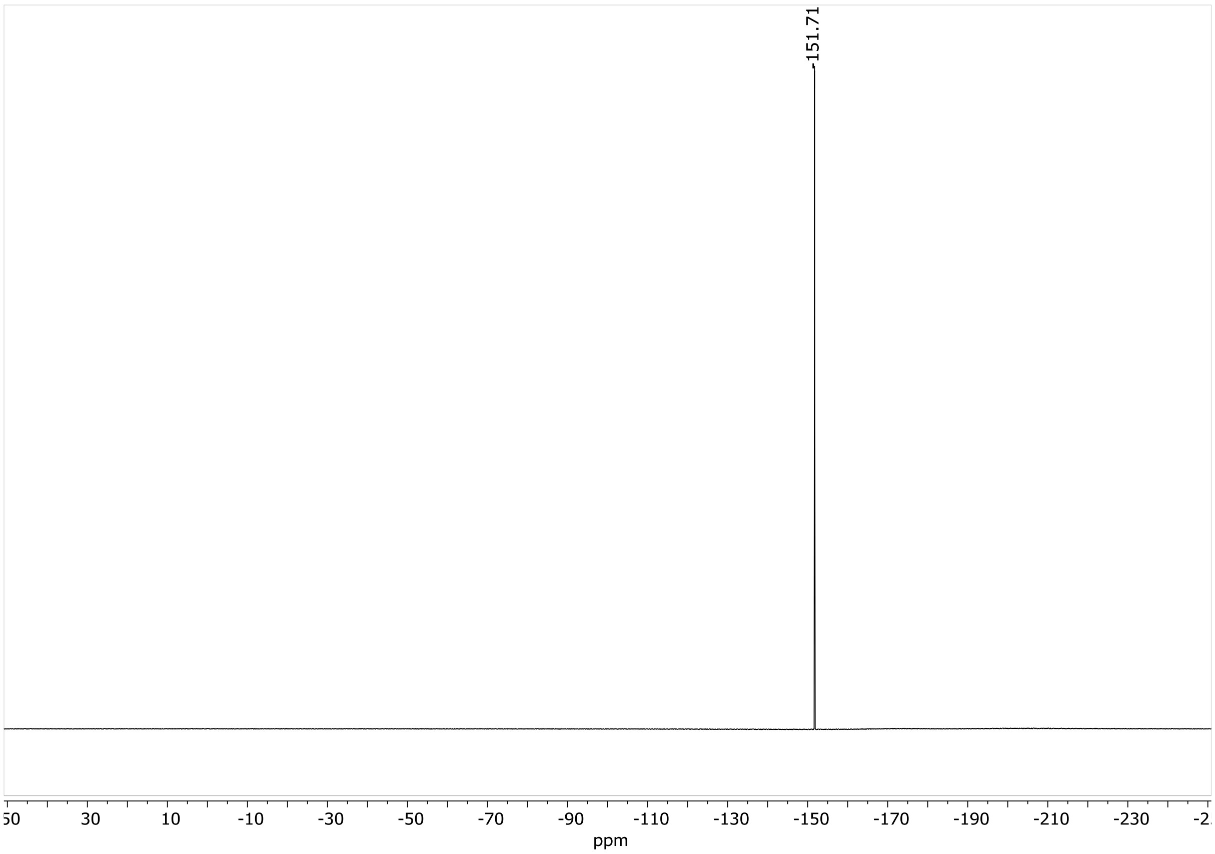
-151.71
50
30
10
-10
-30
-50
-70
-90
-110
-130
-150
-170
-190
-210
-230
ppm

^{1}H NMR,^{13}C NMR and ^{19}F NMR spectrum of compound **1c**

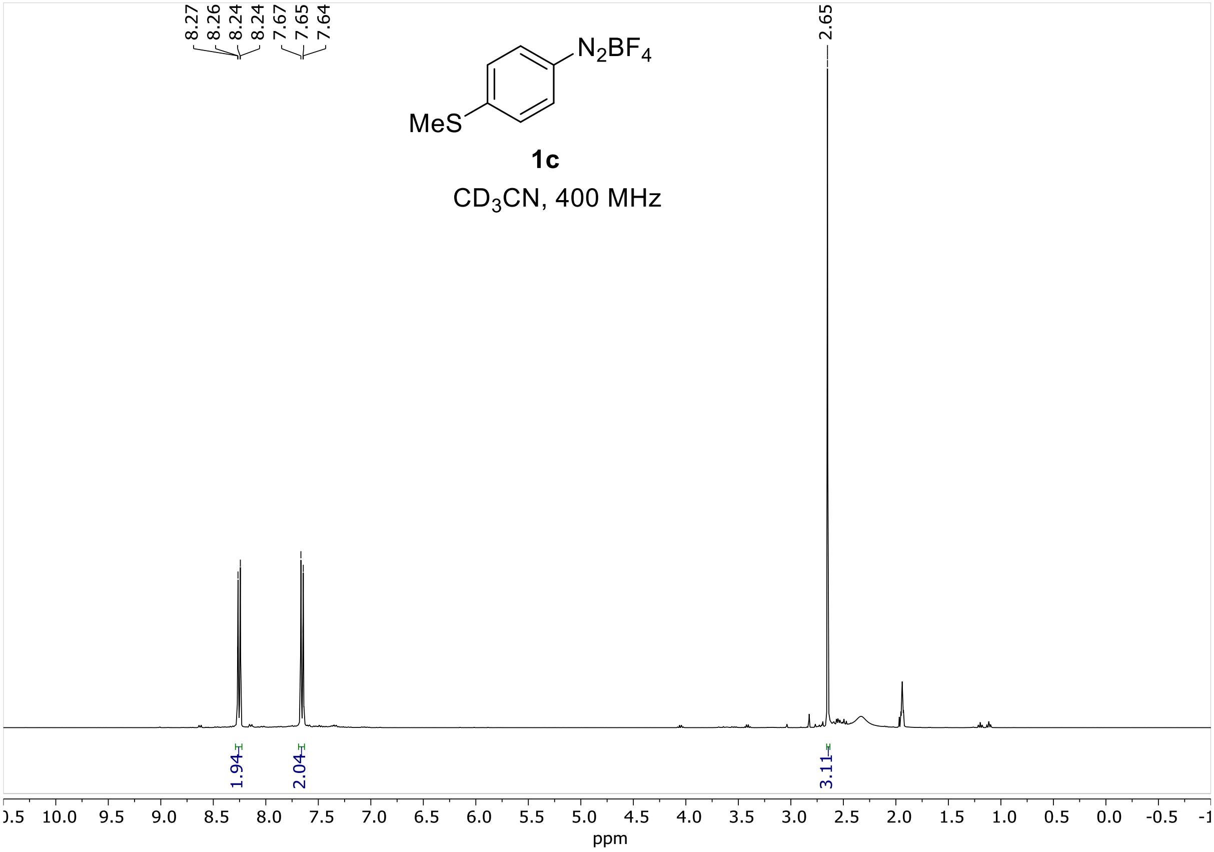

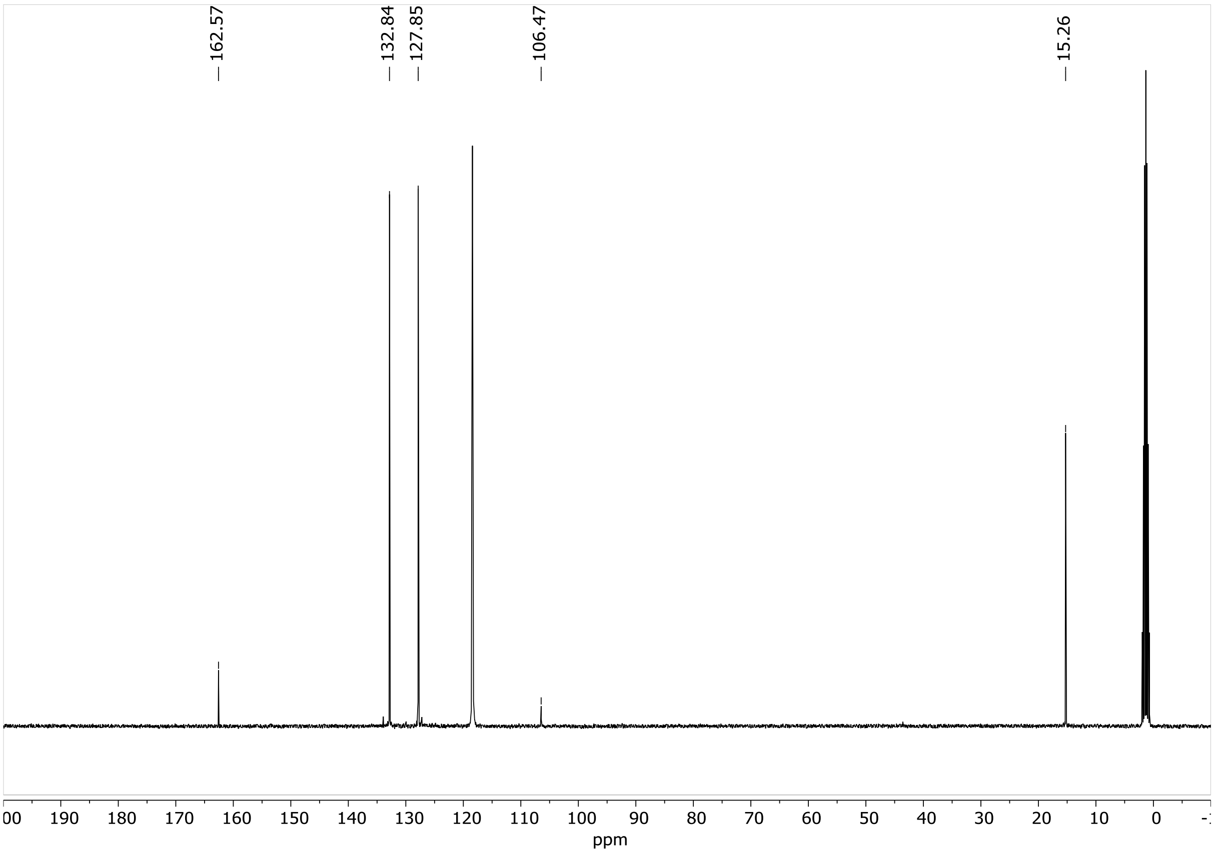

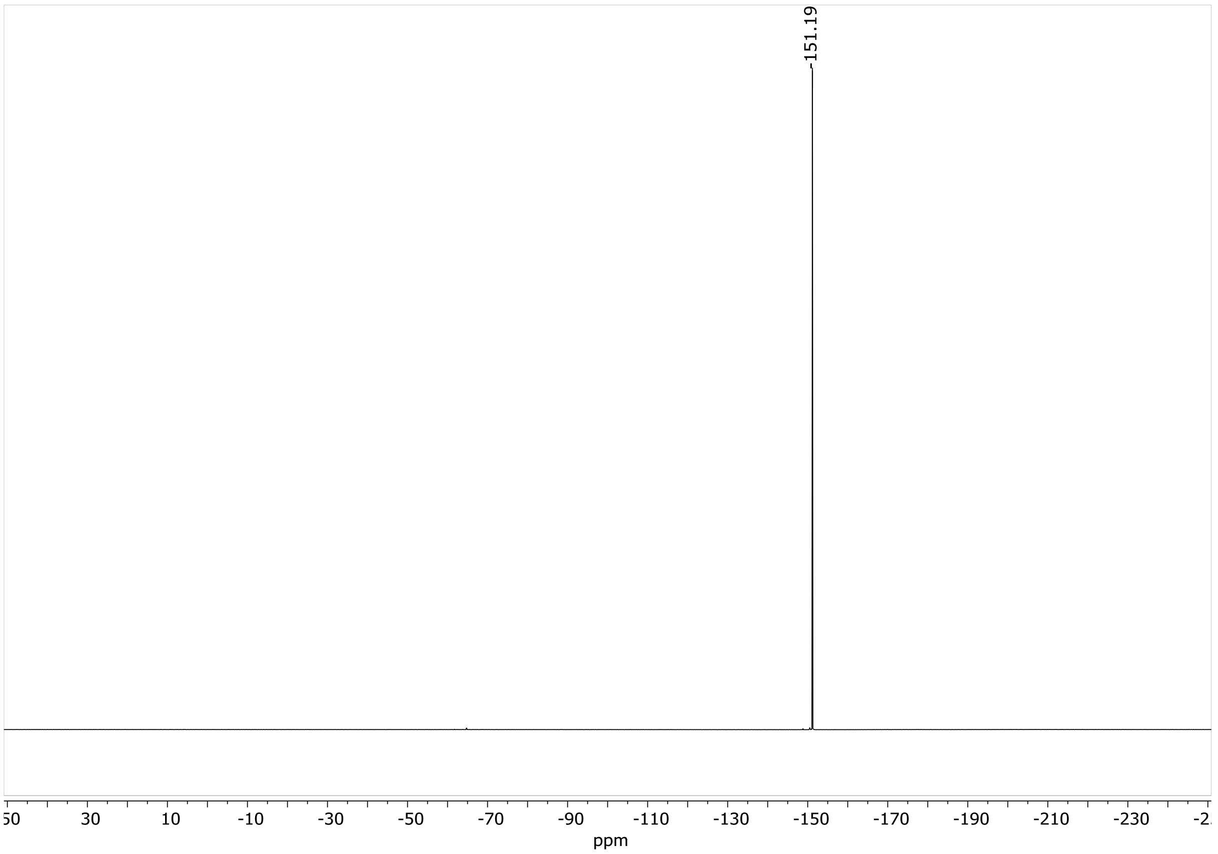
-151.19
30
10
-10
-30
-50
-70
-90
-110
-130
-150
-170
-190
-210
-230
ppm

^1H NMR, ^{13}C NMR and ^{19}F NMR spectrum of compound **1d**

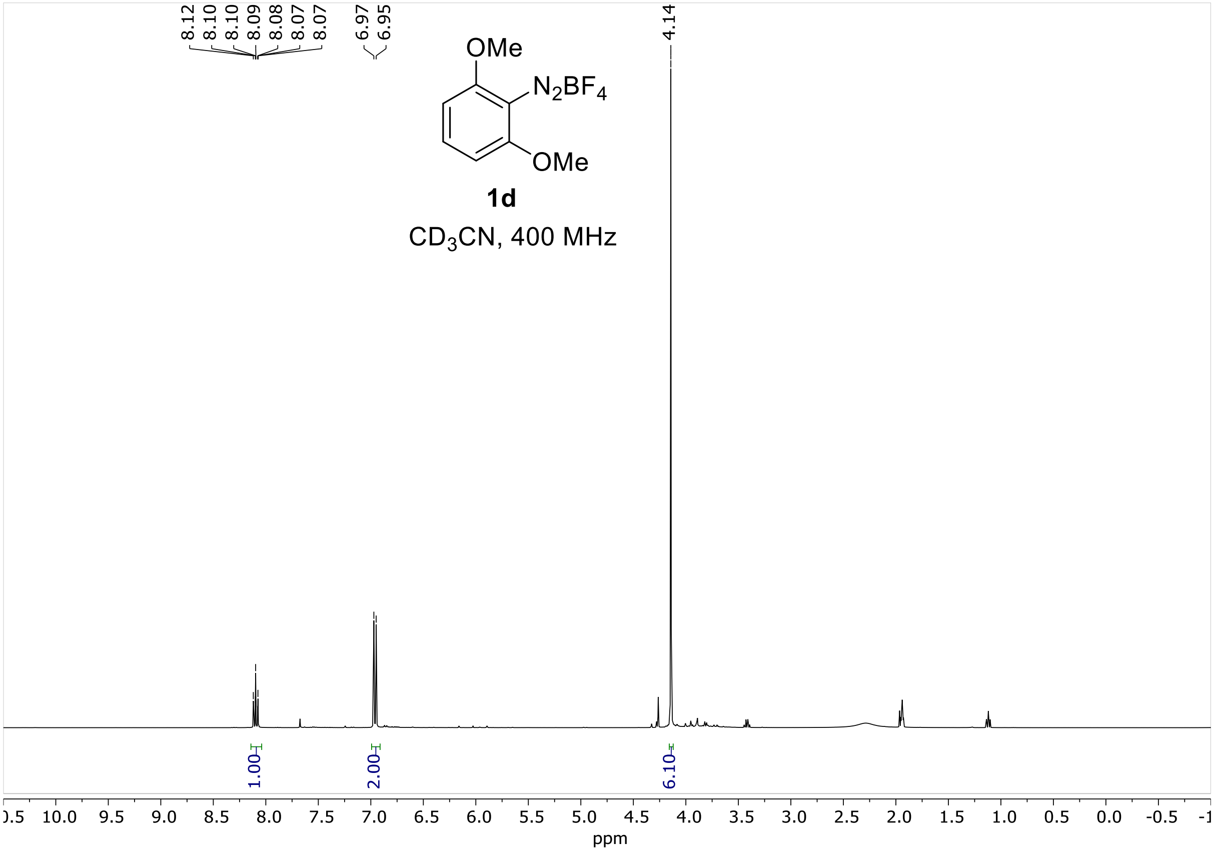

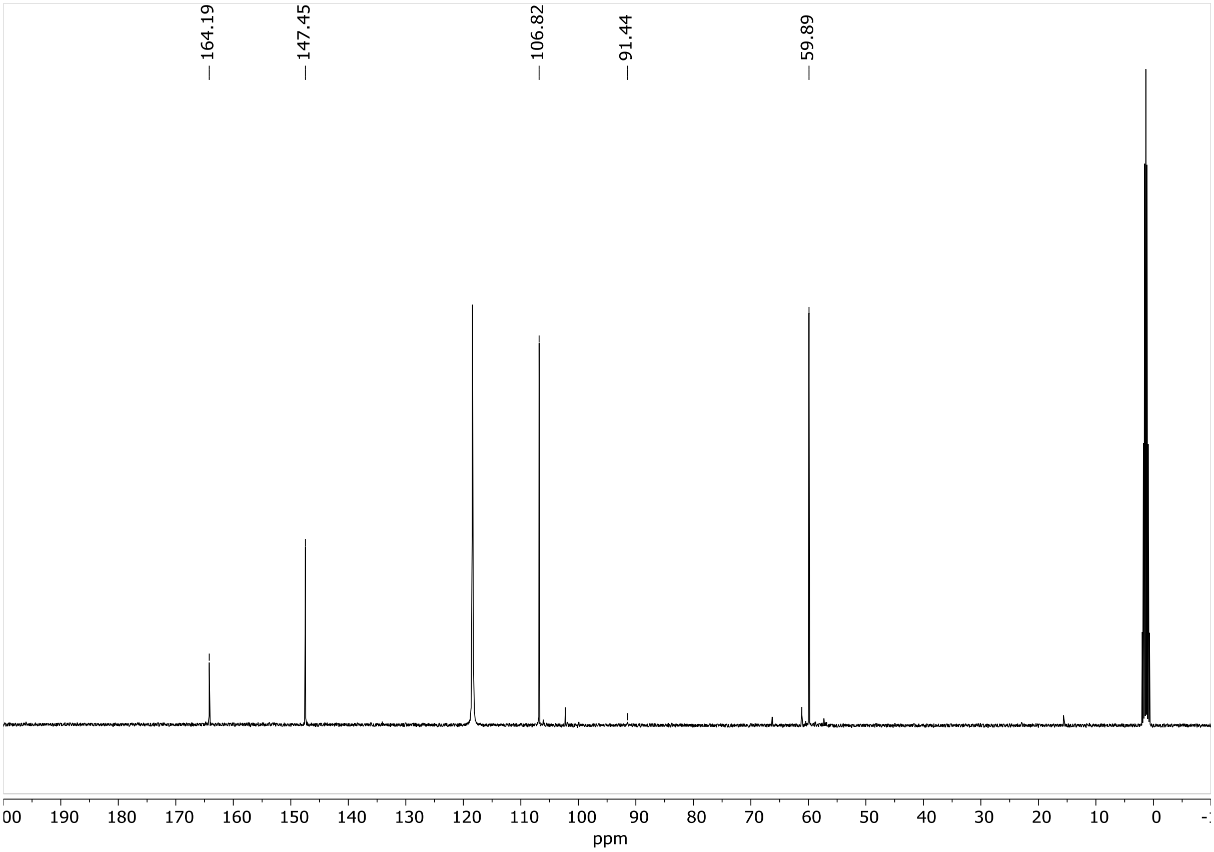

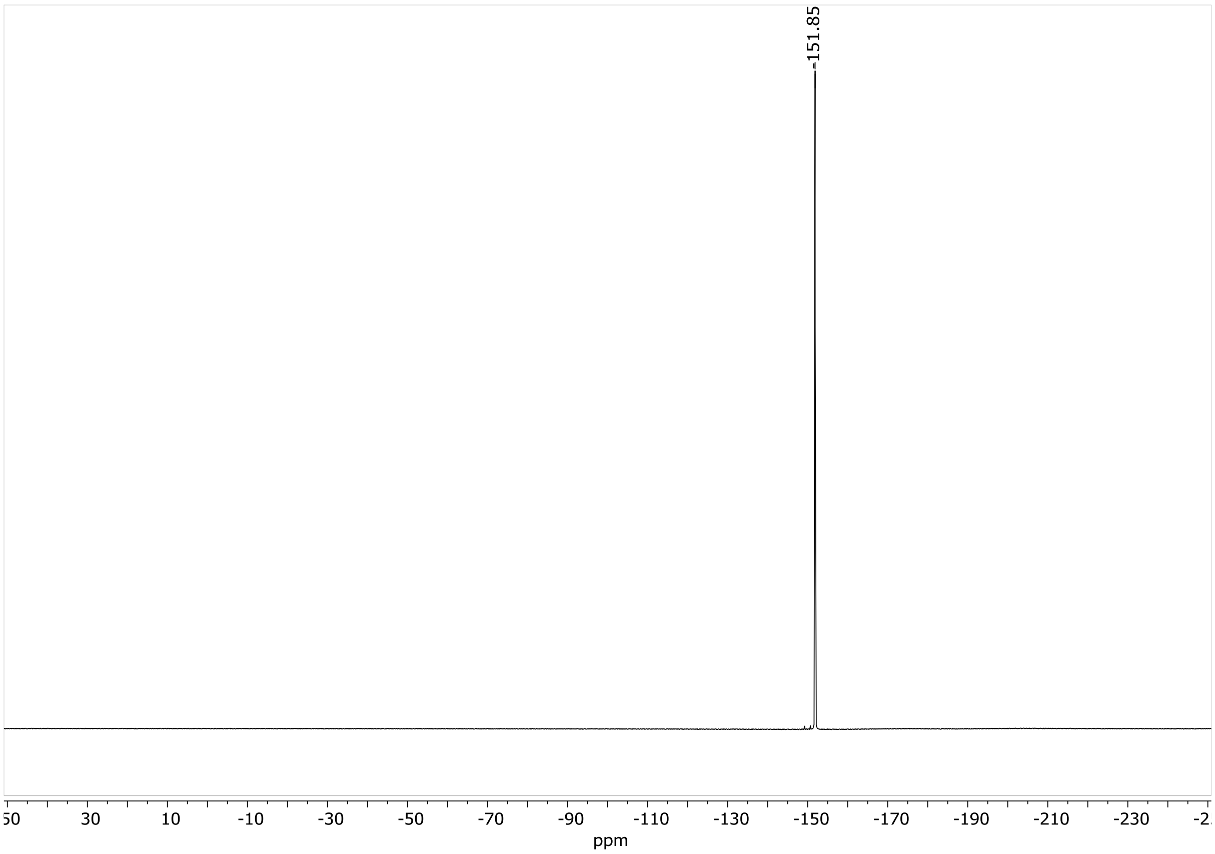
-151.85
ppm

^{1}H NMR,^{13}C NMR and ^{19}F NMR spectrum of compound **1e**

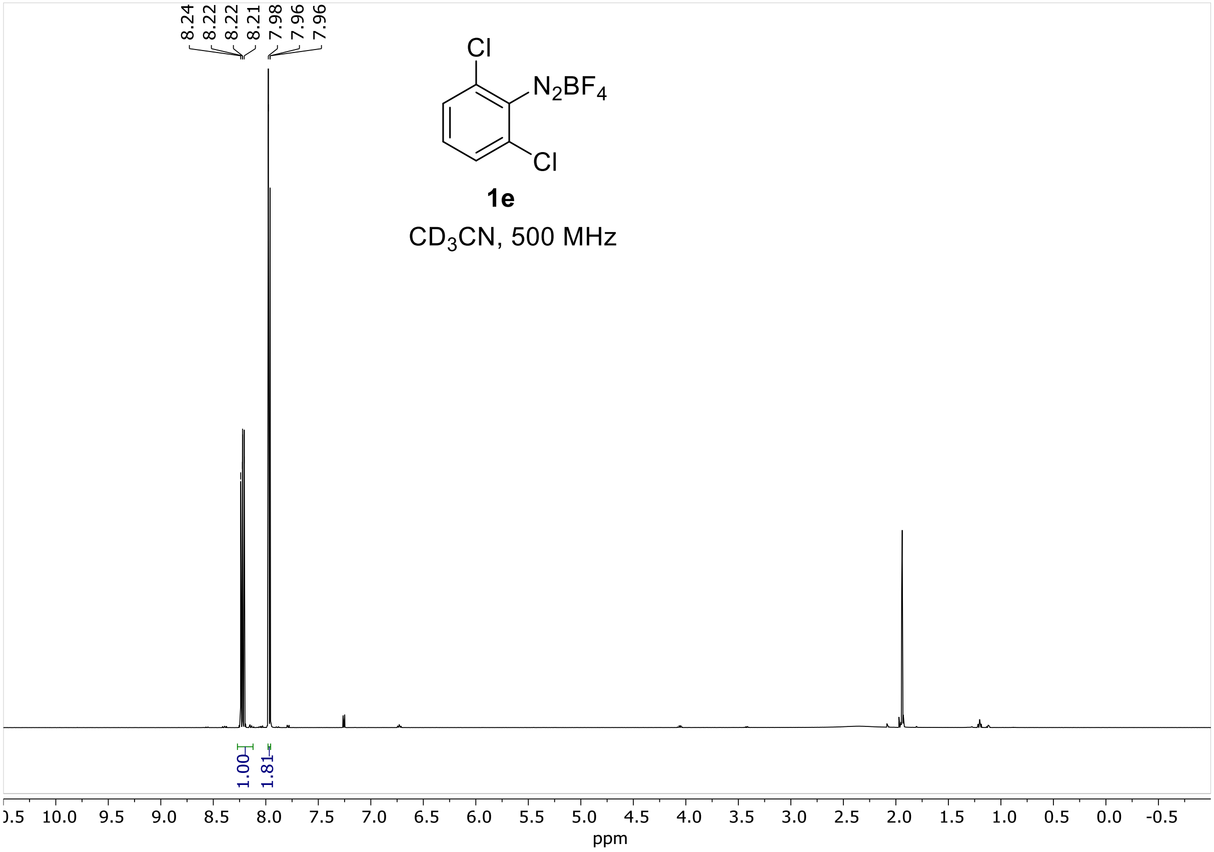

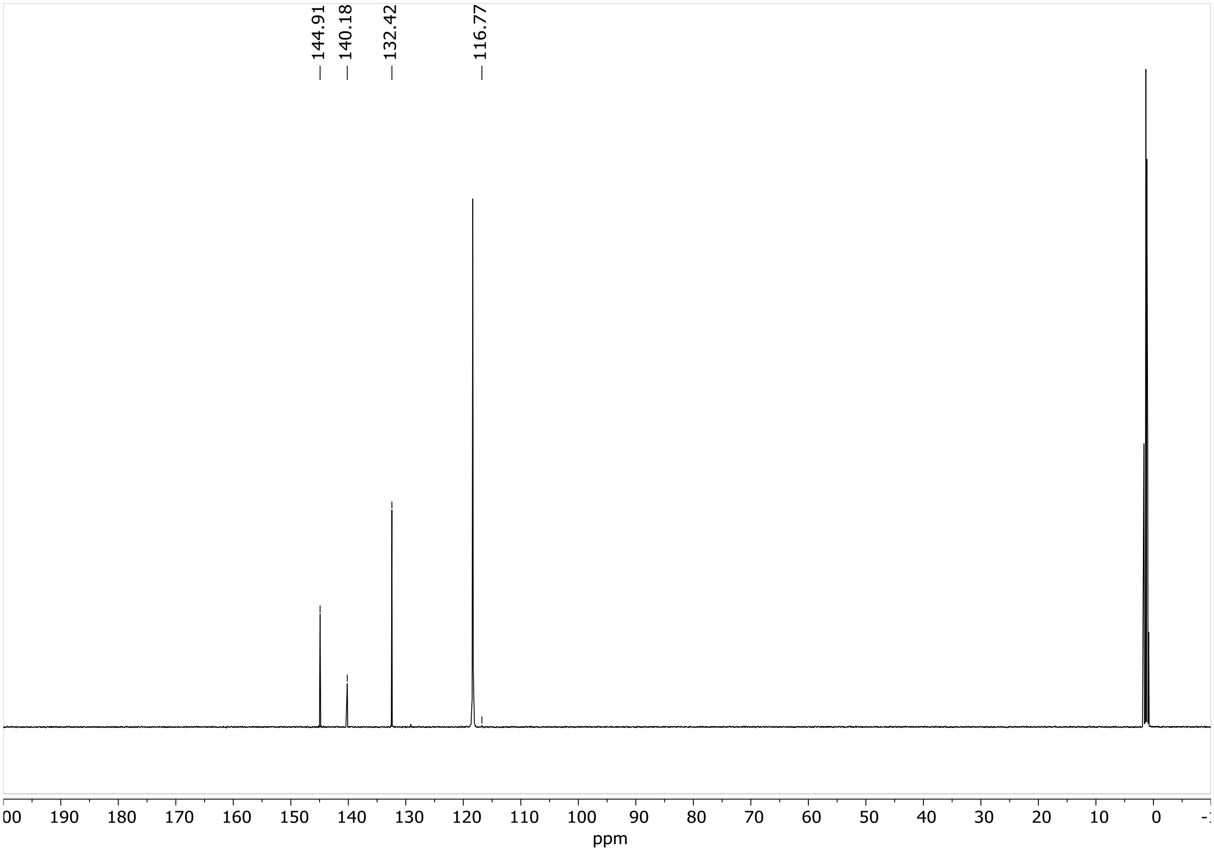

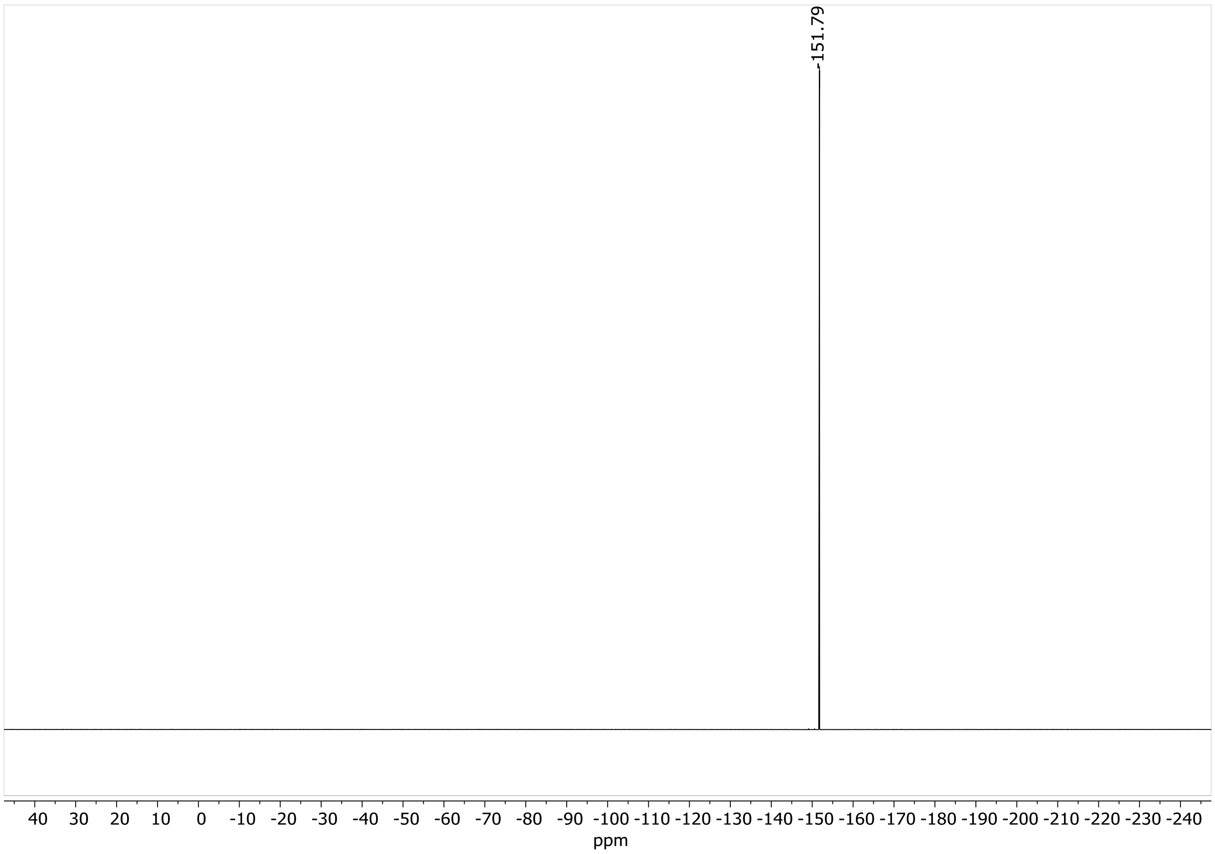
-151.79
40 30 20 10 0 -10 -20 -30 -40 -50 -60 -70 -80 -90 -100 -110 -120 -130 -140 -150 -160 -170 -180 -190 -200 -210 -220 -230 -240
ppm

^{1}H NMR, ^{13}C NMR and ^{19}F NMR spectrum of compound **1f**

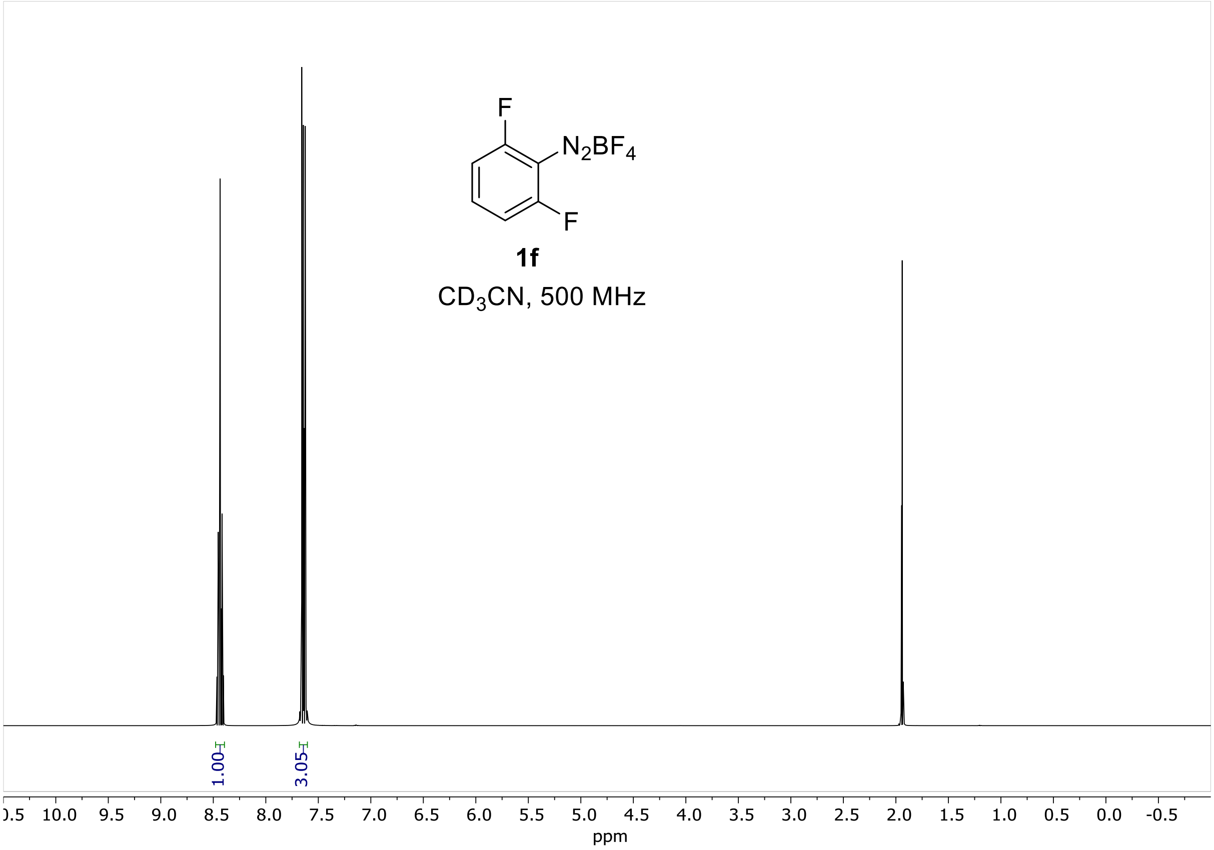

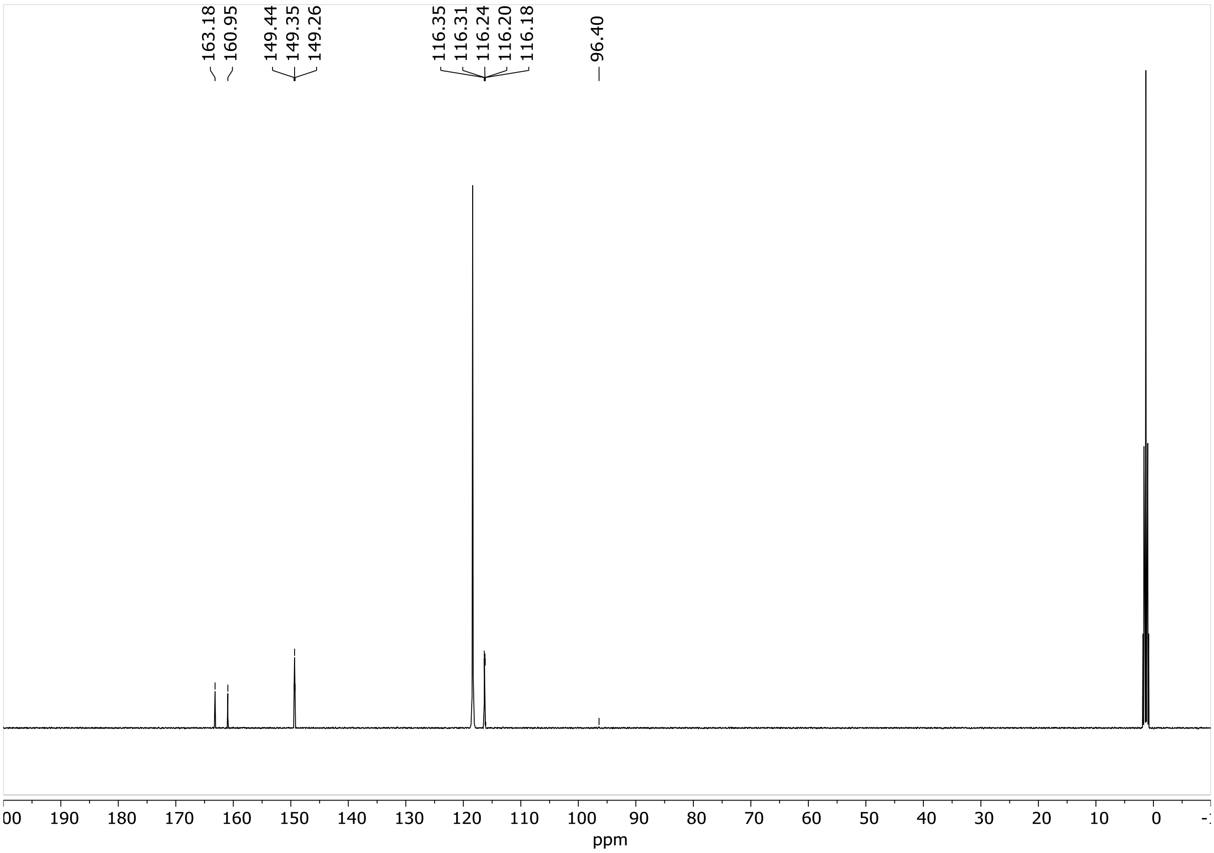

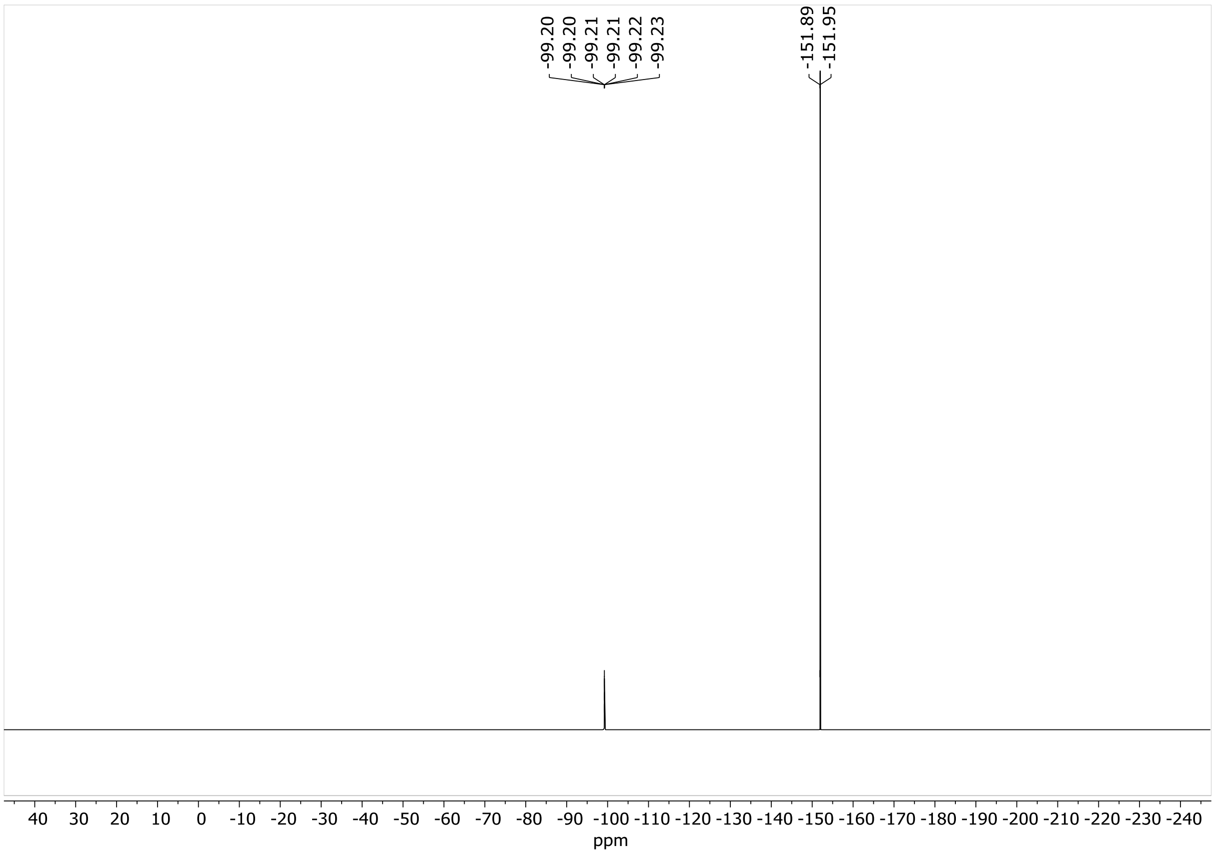
-99.20
-99.20
-99.21
-99.21
-99.22
-99.23
-151.89
-151.95
40 30 20 10 0 -10 -20 -30 -40 -50 -60 -70 -80 -90 -100 -110 -120 -130 -140 -150 -160 -170 -180 -190 -200 -210 -220 -230 -240
ppm

^1H NMR, ^{13}C NMR and ^{19}F NMR spectrum of compound **1h**

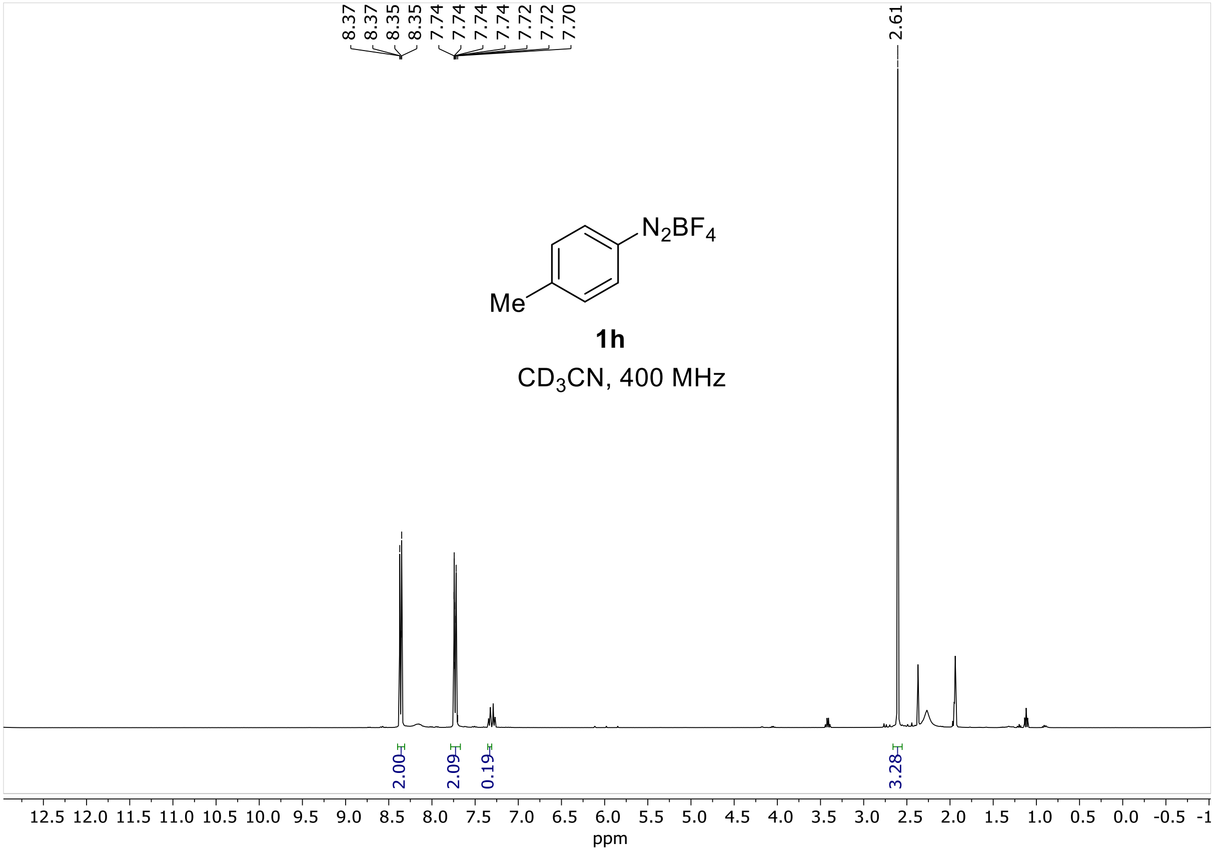

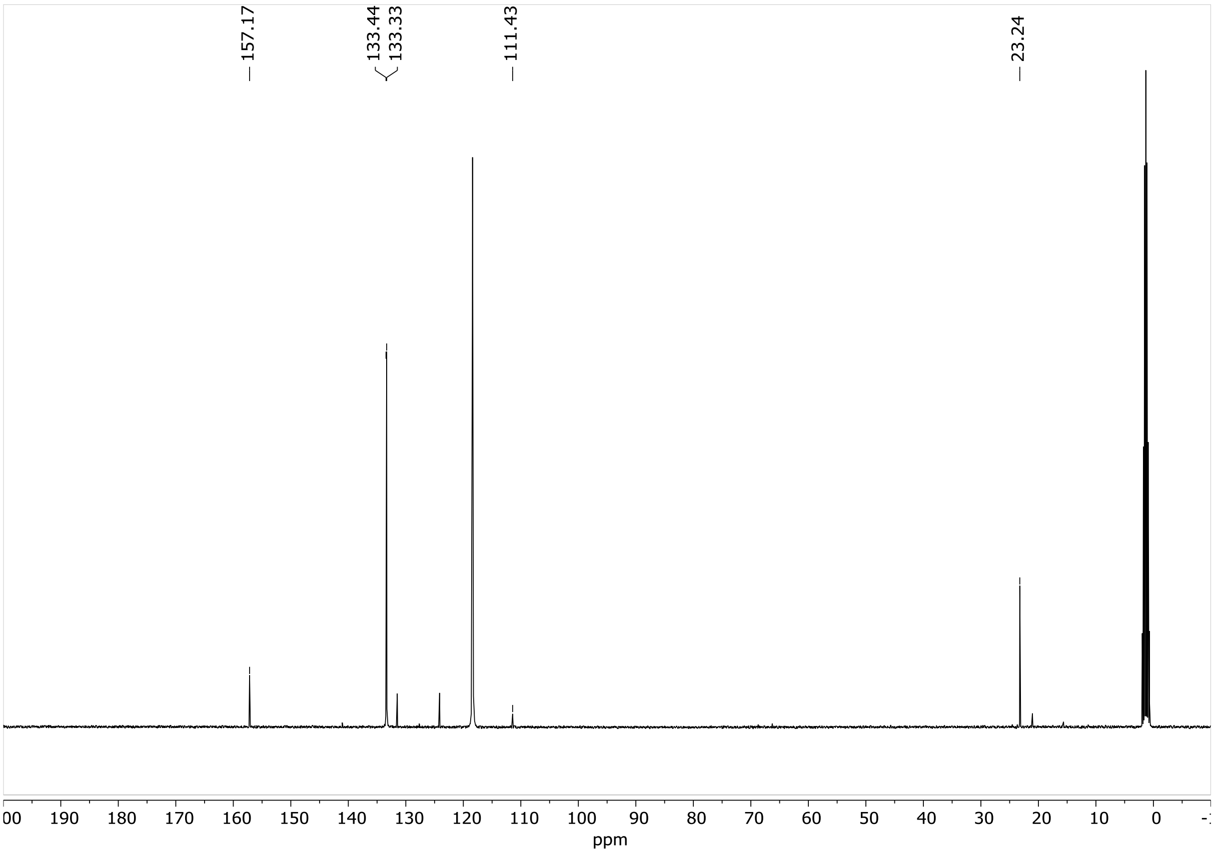

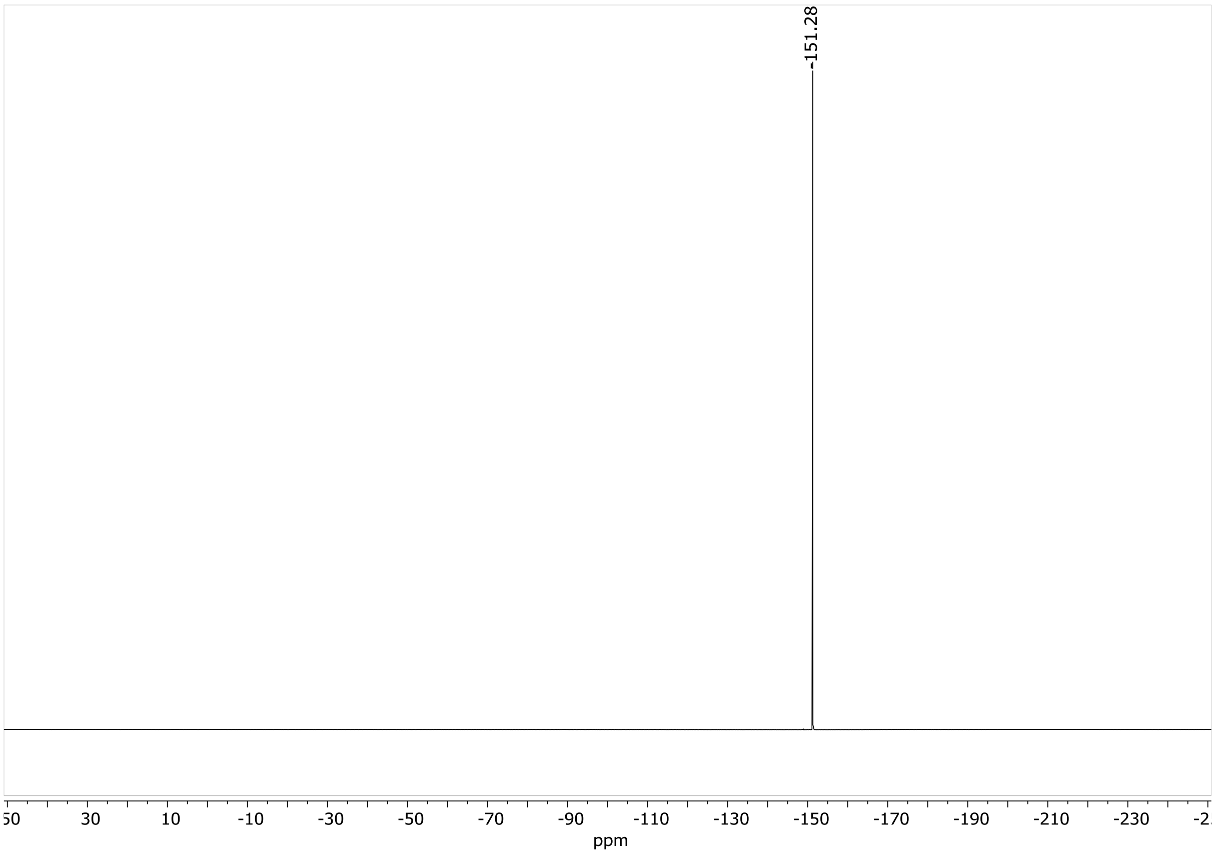

^{1}H NMR,^{13}C NMR and ^{19}F NMR spectrum of compound **1i**

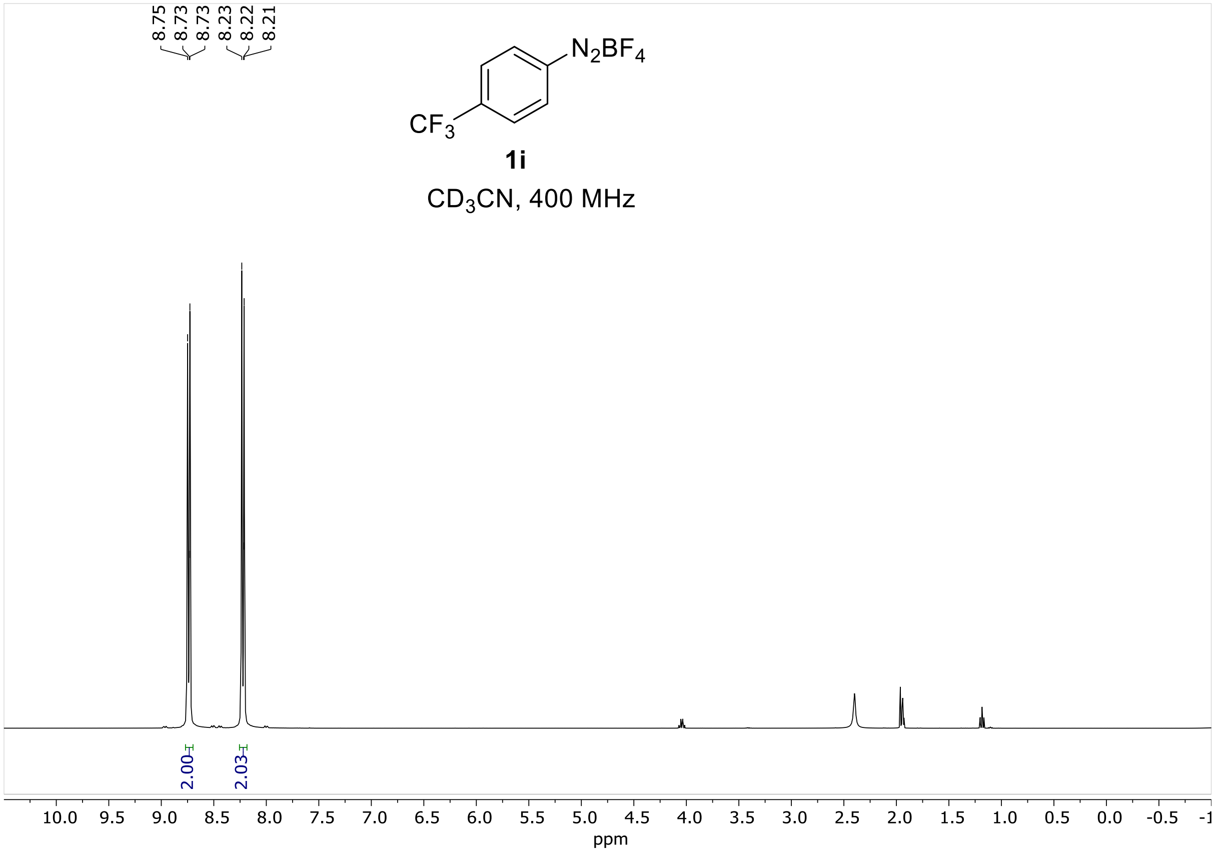

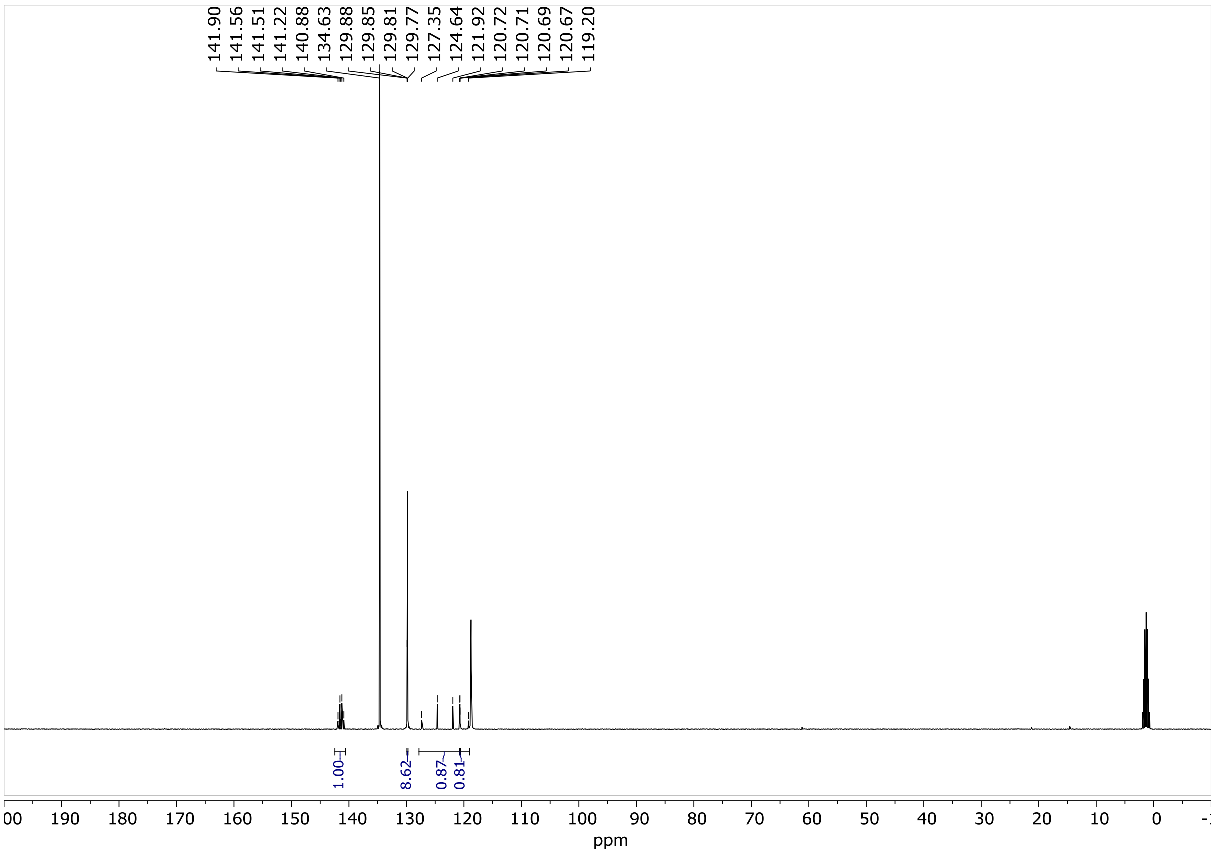

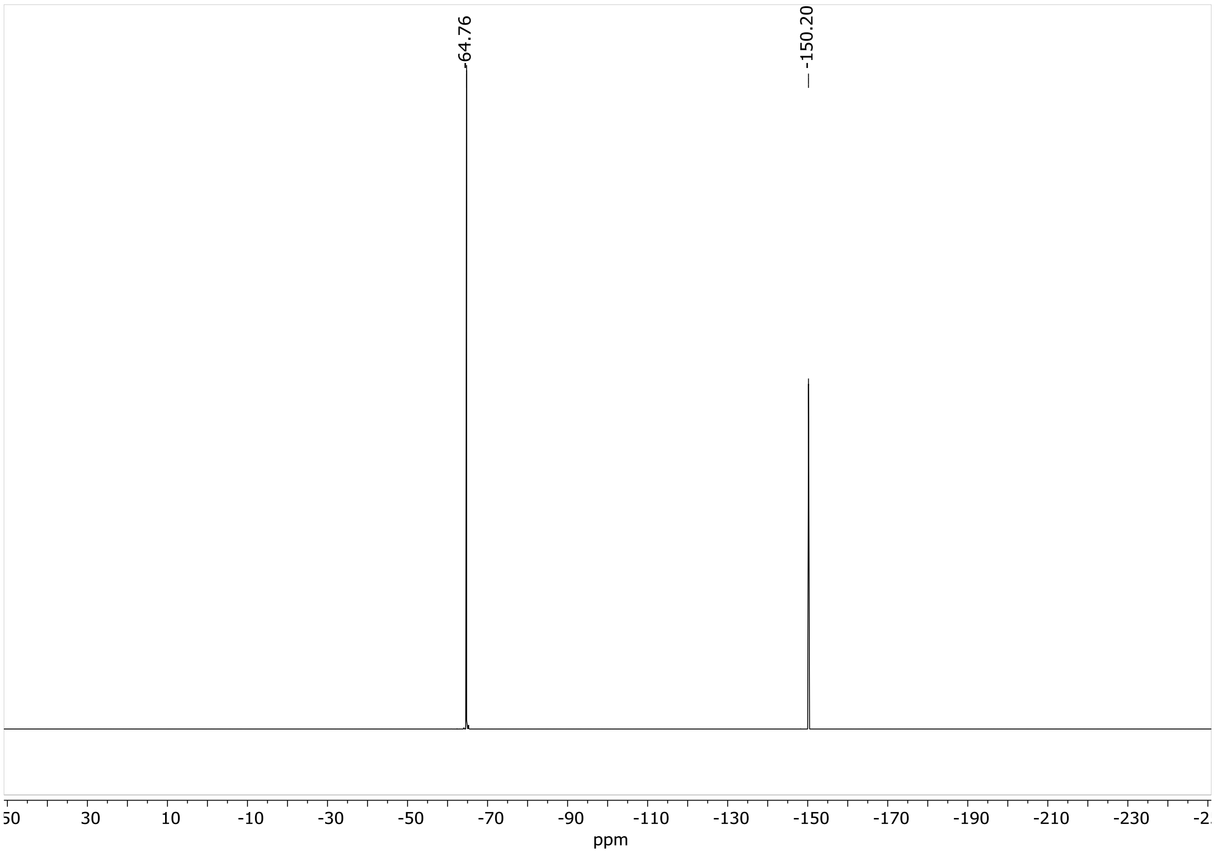

^{1}H NMR,^{13}C NMR and ^{19}F NMR spectrum of compound **1j**

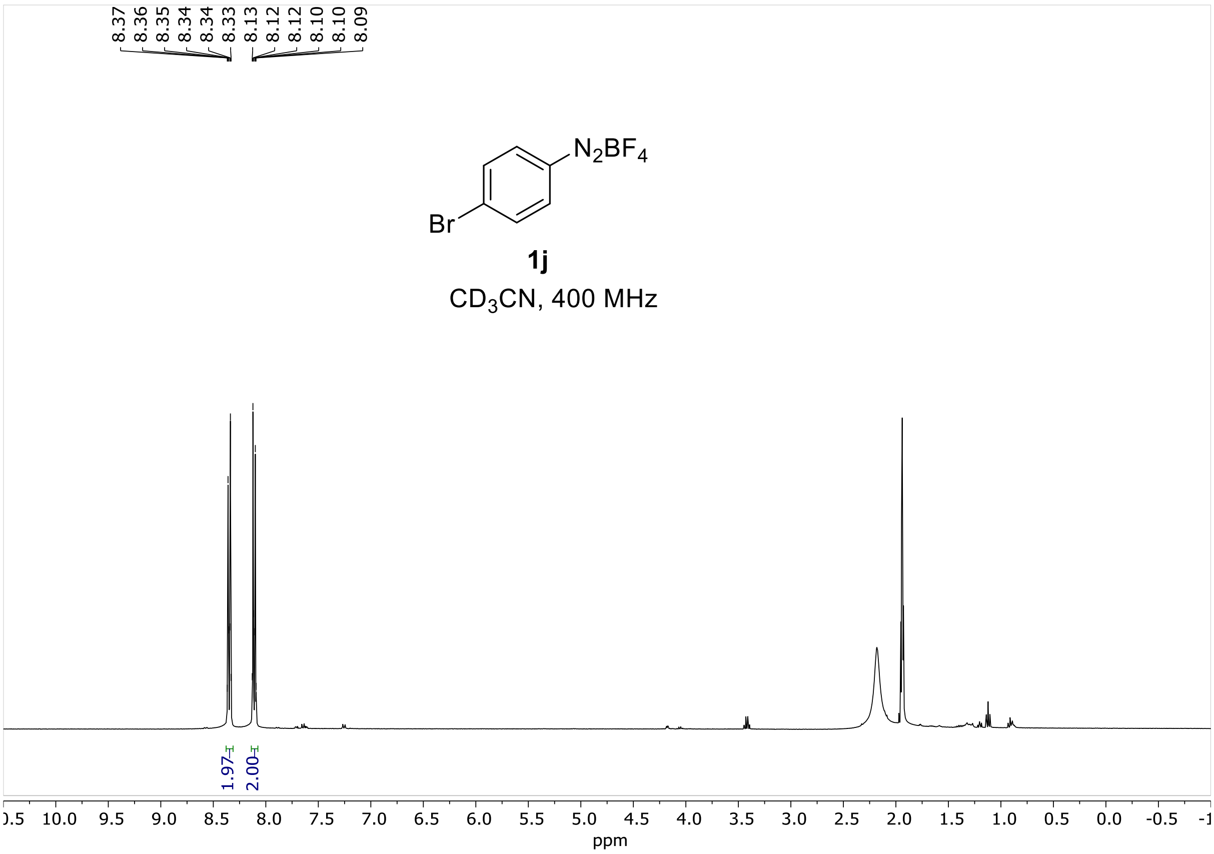
8.37
8.36
8.35
8.34
8.34
8.33
8.13
8.12
8.12
8.10
8.10
8.09
N_2BF_4
Br
1j
CD_3CN, 400 MHz
1.97
2.00
ppm

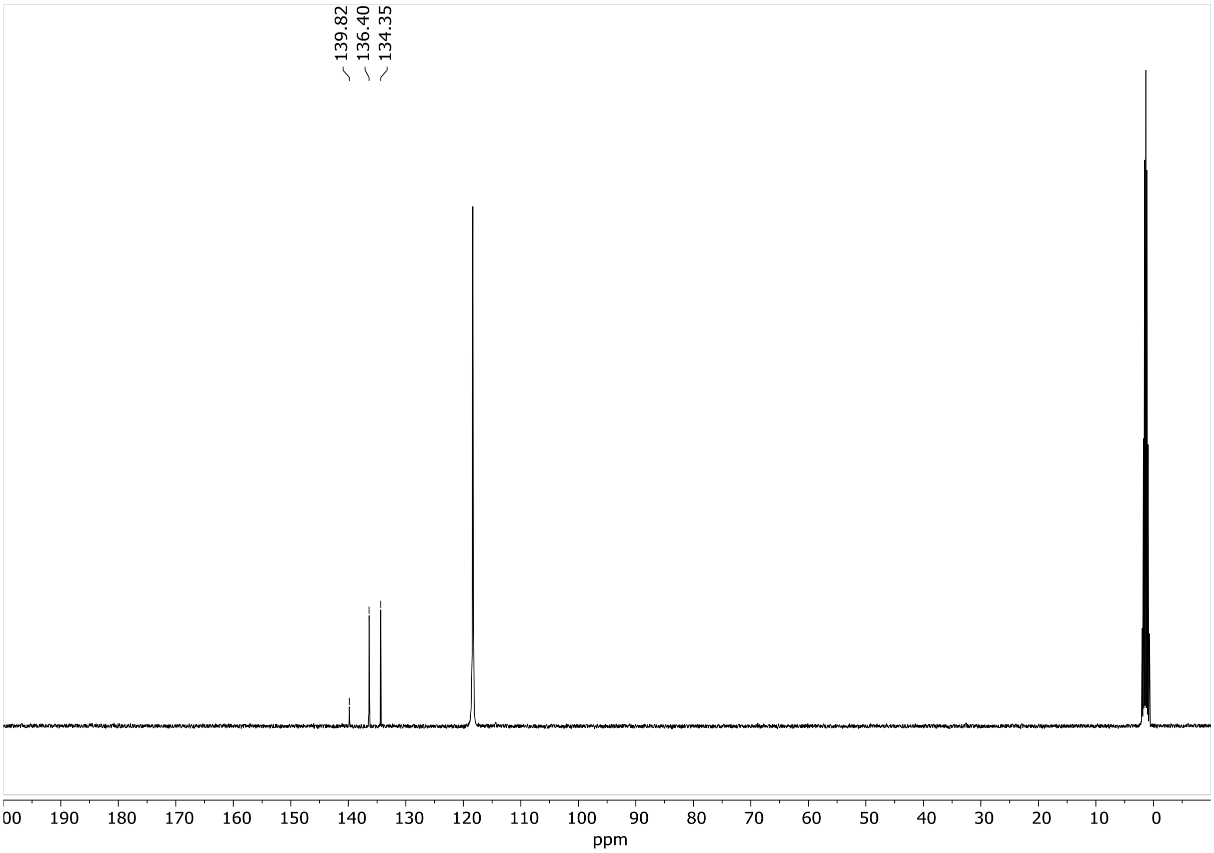
139.82
136.40
134.35
ppm

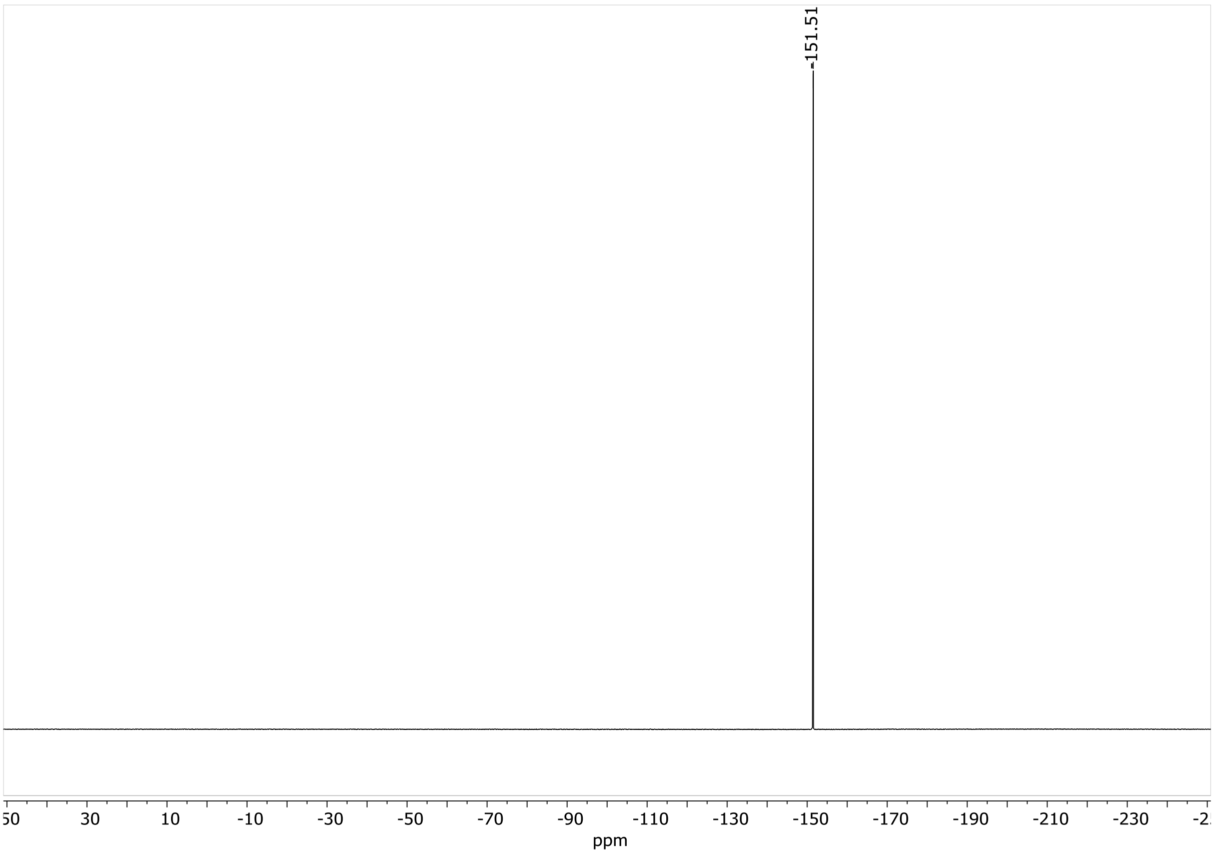

^{1}H NMR,^{13}C NMR and ^{19}F NMR spectrum of compound **1k**

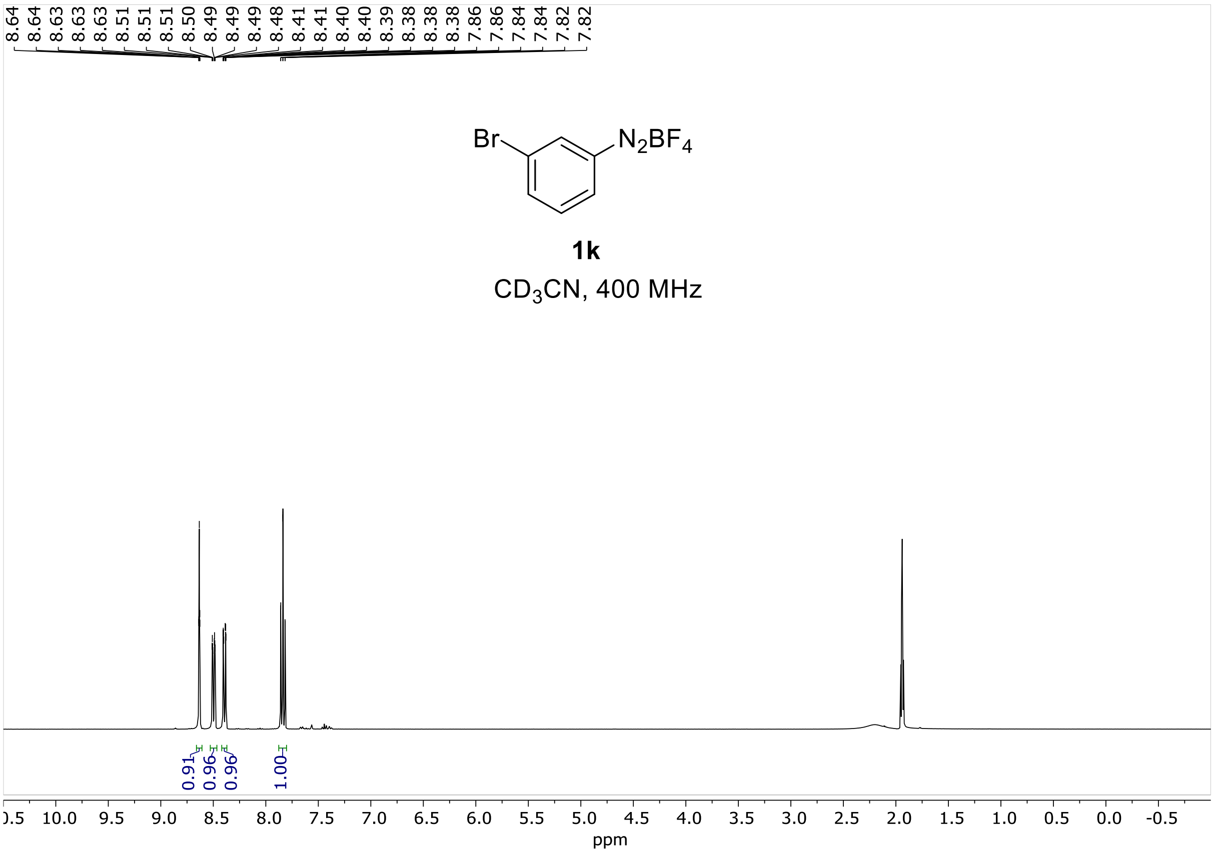

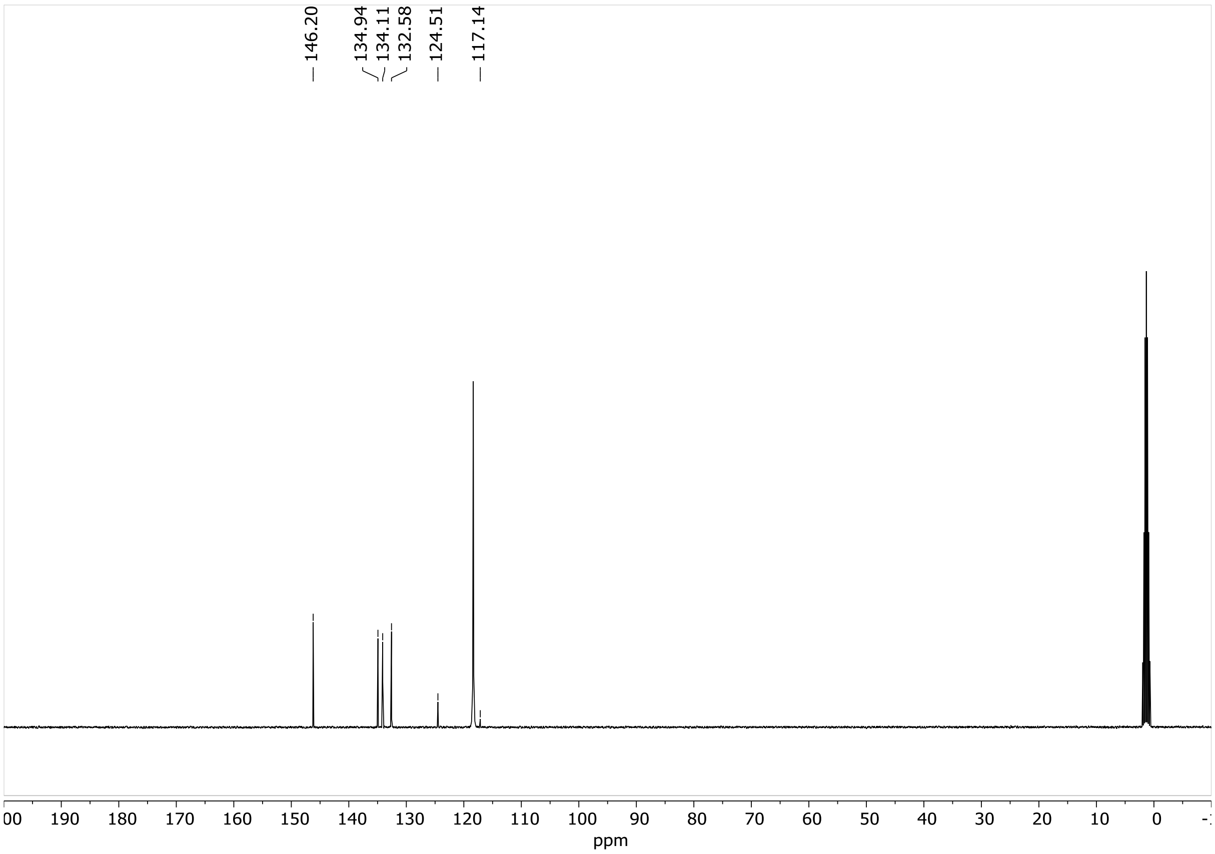

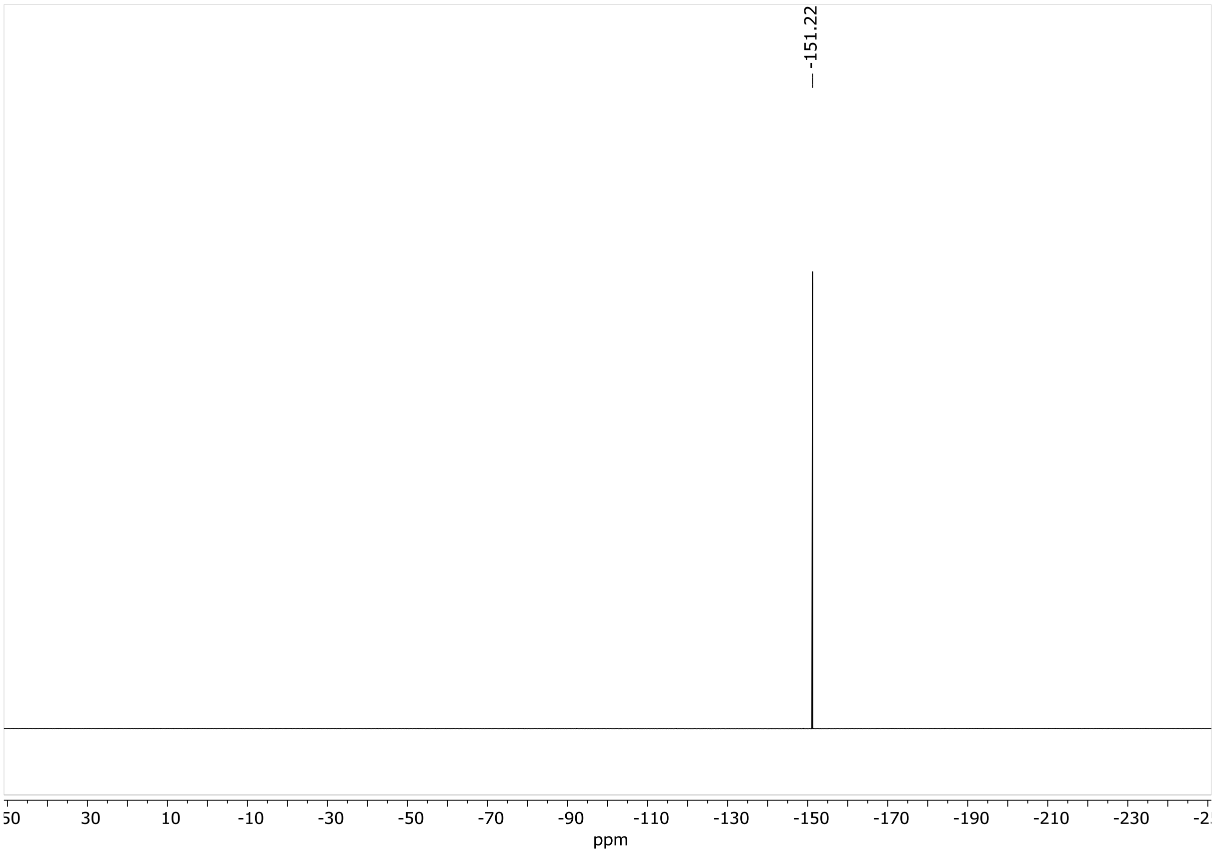

^{1}H NMR,^{13}C NMR and ^{19}F NMR spectrum of compound **1l**

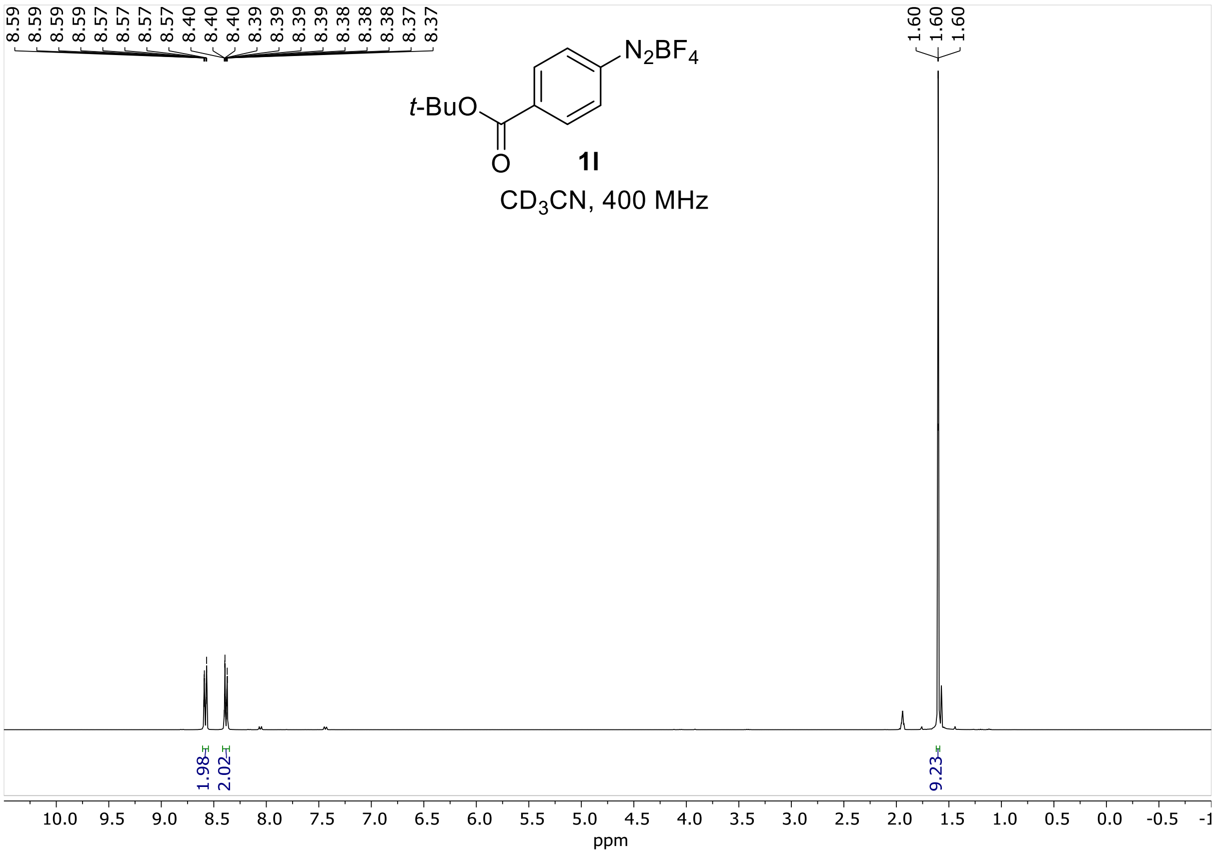
8.59
8.59
8.59
8.59
8.57
8.57
8.57
8.57
8.40
8.40
8.40
8.39
8.39
8.39
8.39
8.38
8.38
8.38
8.37
8.37
1.60
1.60
1.60
N2BF4
t-BuO
O
1l
CD3CN, 400 MHz
1.98
2.02
9.23
10.0 9.5 9.0 8.5 8.0 7.5 7.0 6.5 6.0 5.5 5.0 4.5 4.0 3.5 3.0 2.5 2.0 1.5 1.0 0.5 0.0 -0.5
ppm

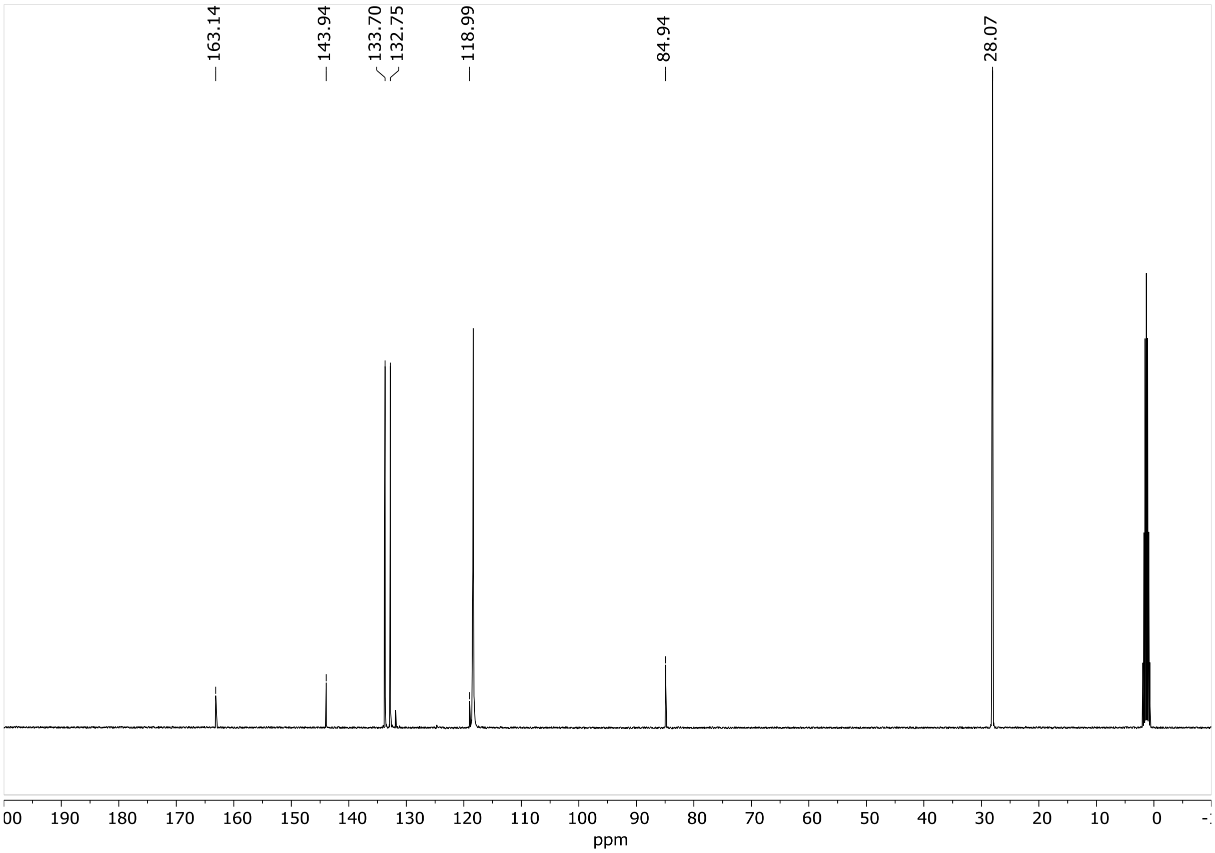
163.14
143.94
133.70
132.75
118.99
84.94
28.07
190 180 170 160 150 140 130 120 110 100 90 80 70 60 50 40 30 20 10 0
ppm

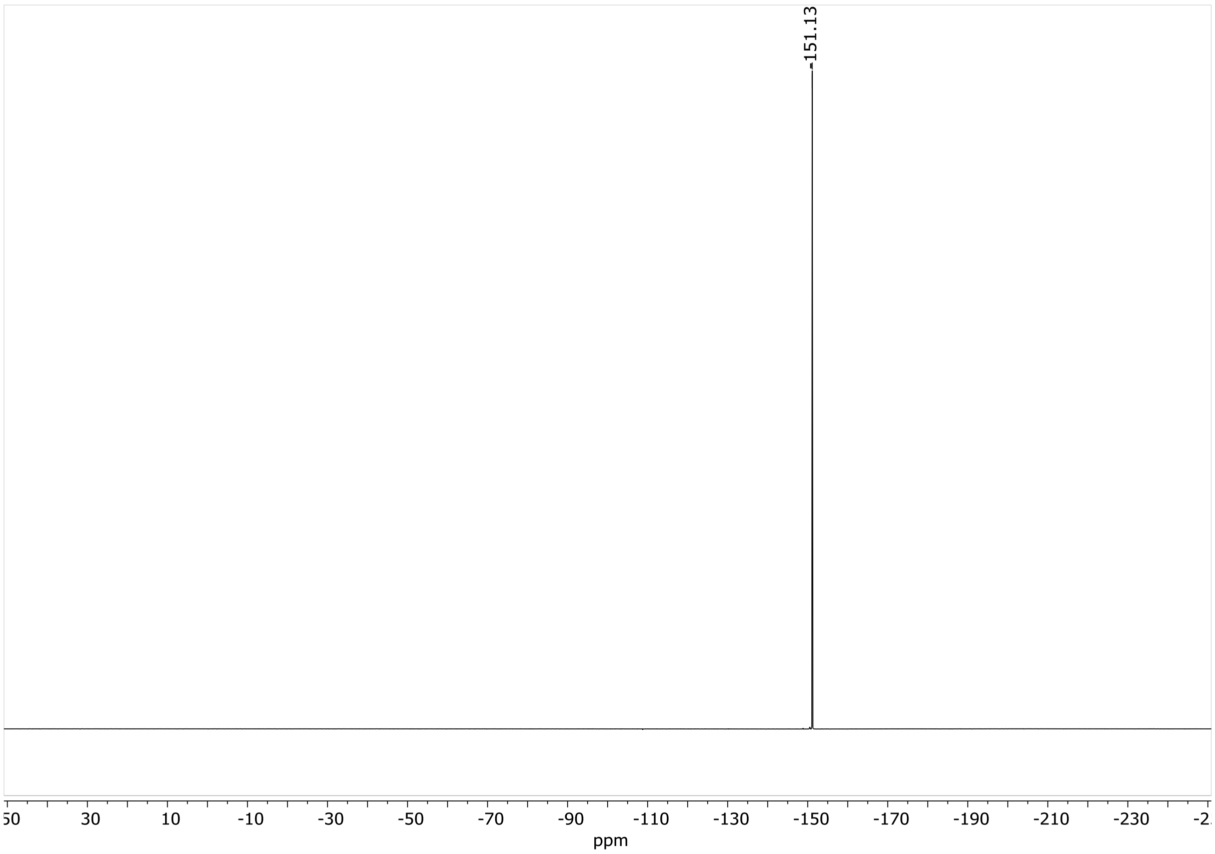

^{1}H NMR,^{13}C NMR and ^{19}F NMR spectrum of compound **1m**

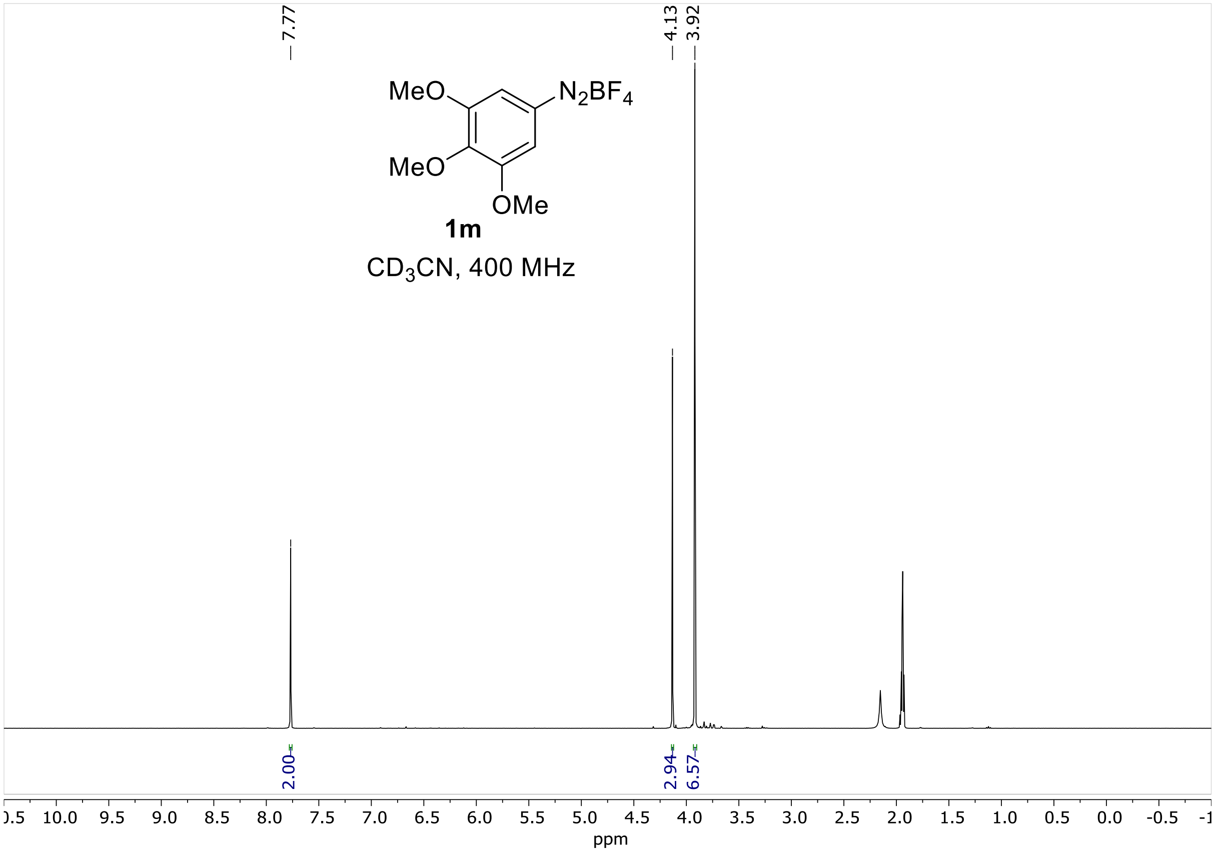

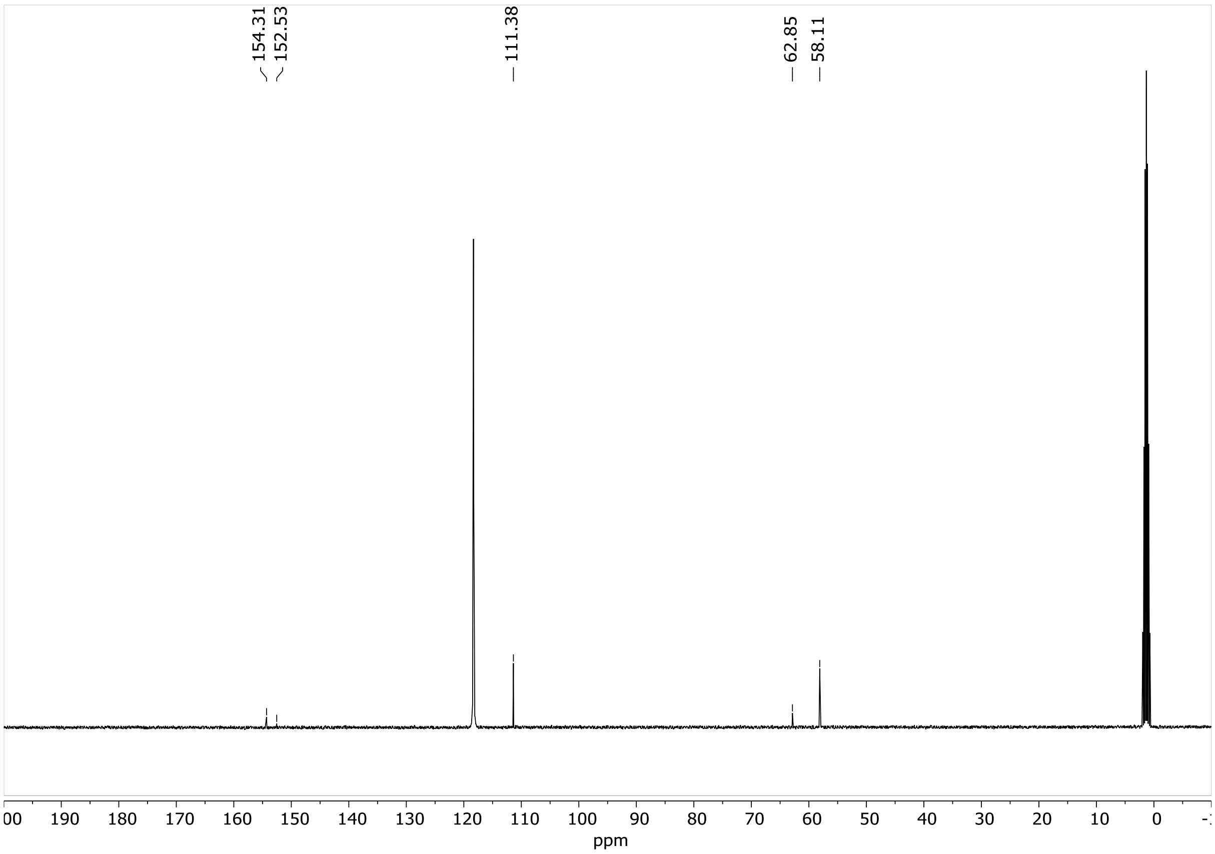
154.31
152.53
111.38
62.85
58.11
ppm

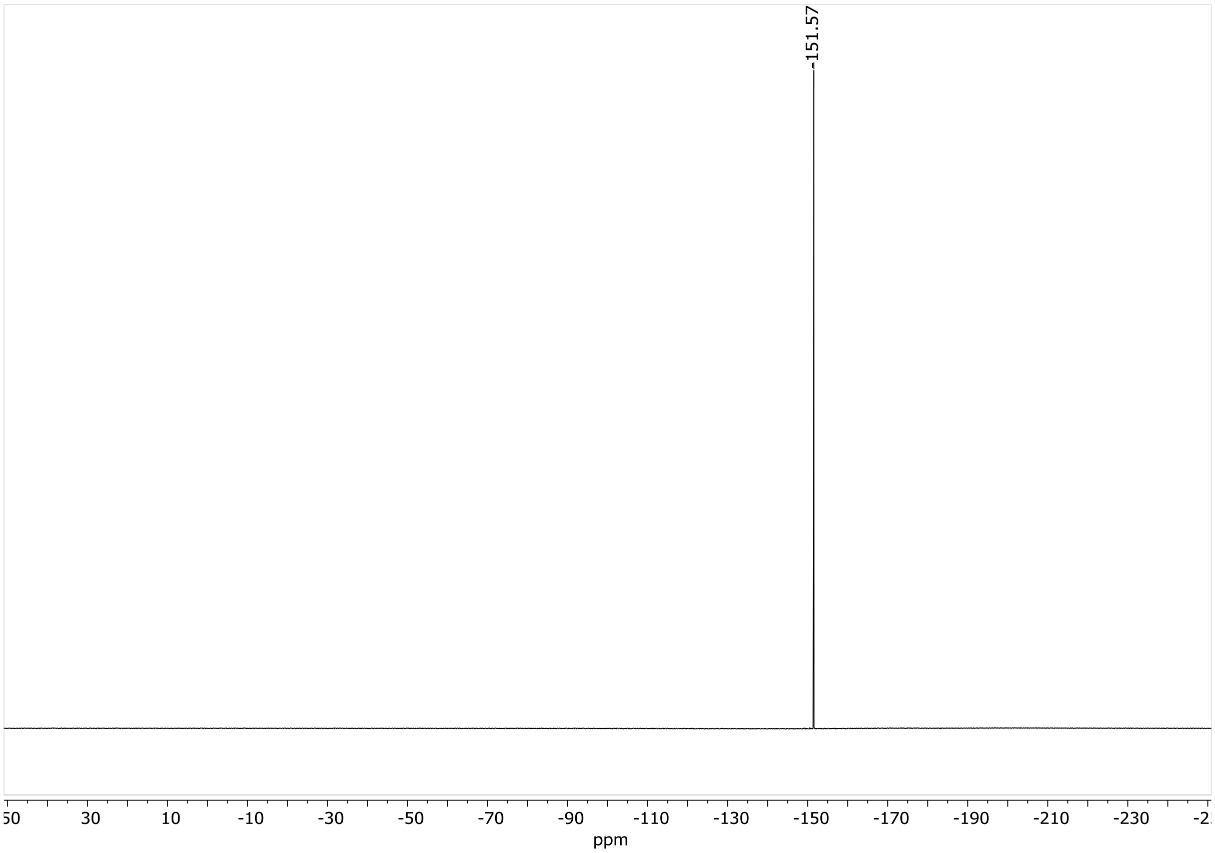
-151.57
ppm

^{1}H NMR,^{13}C NMR and ^{19}F NMR spectrum of compound **1n**

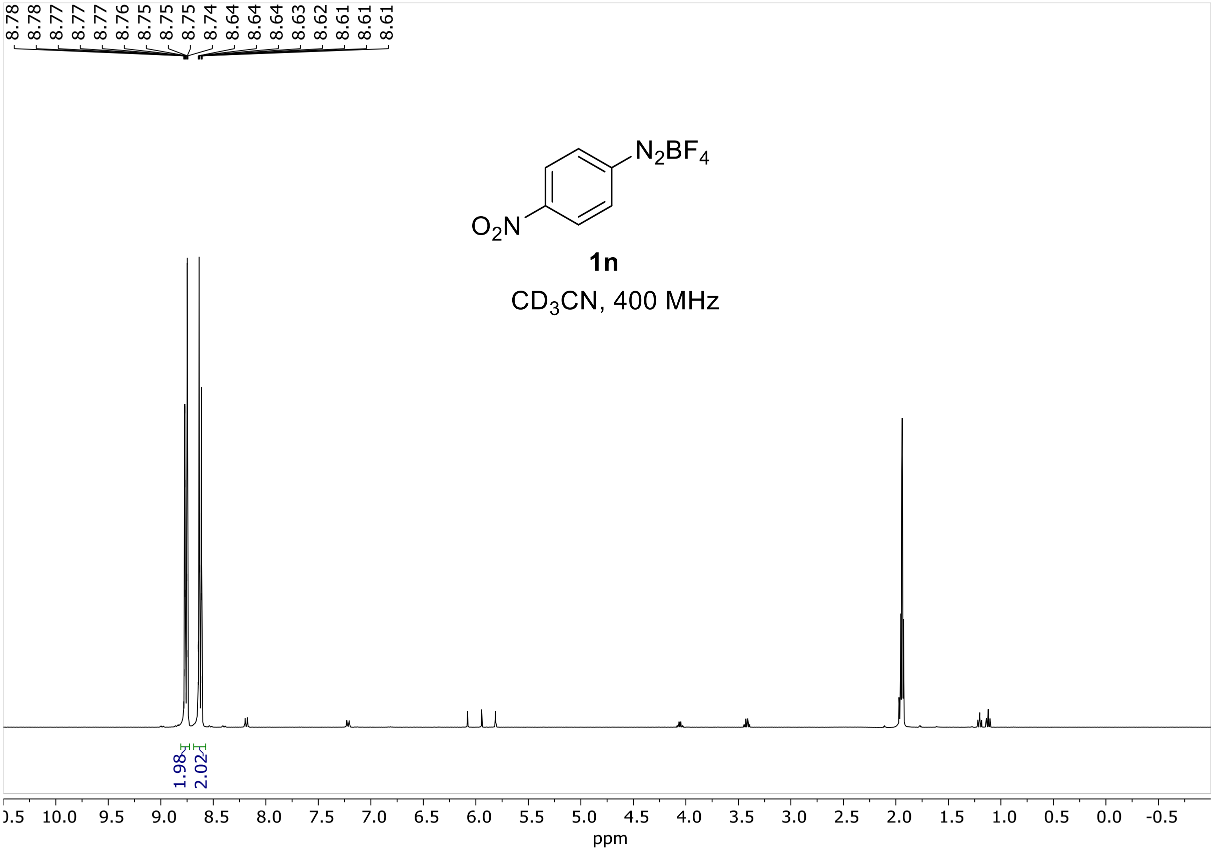

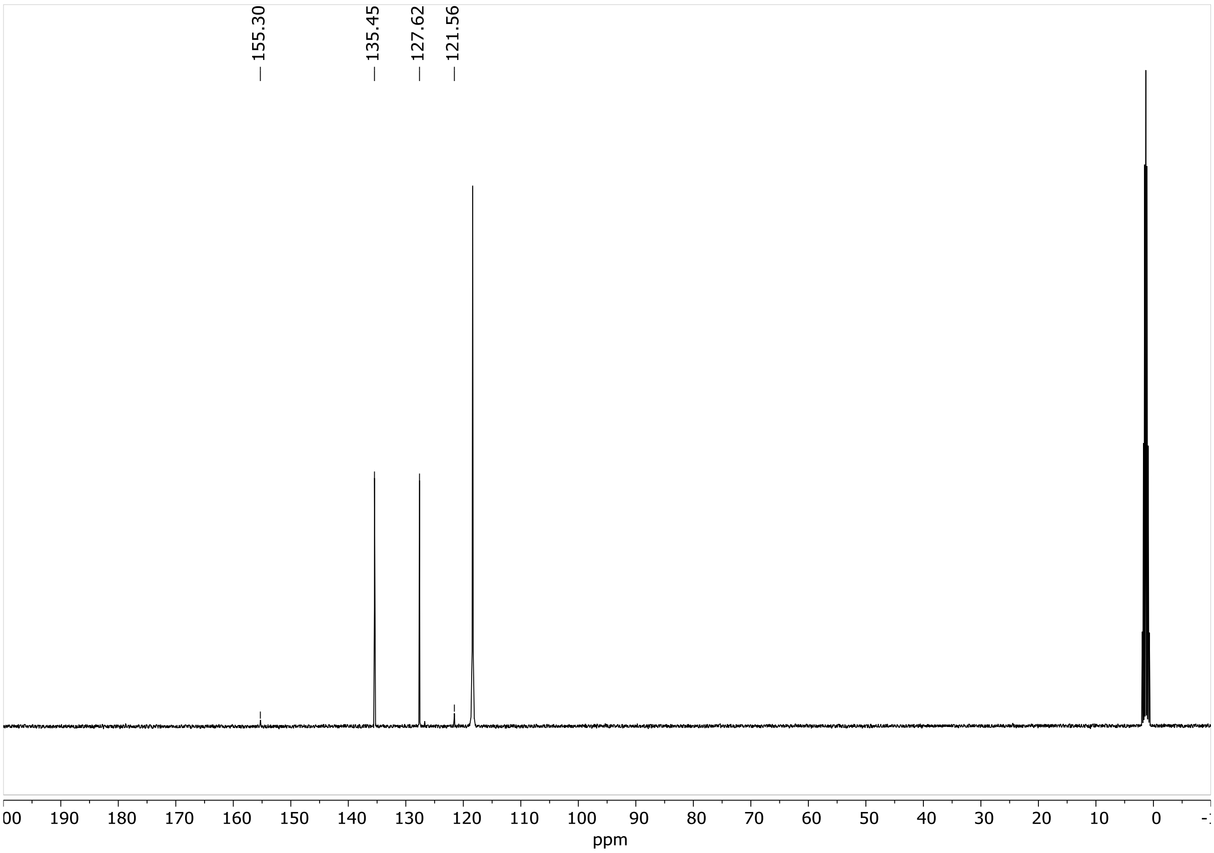

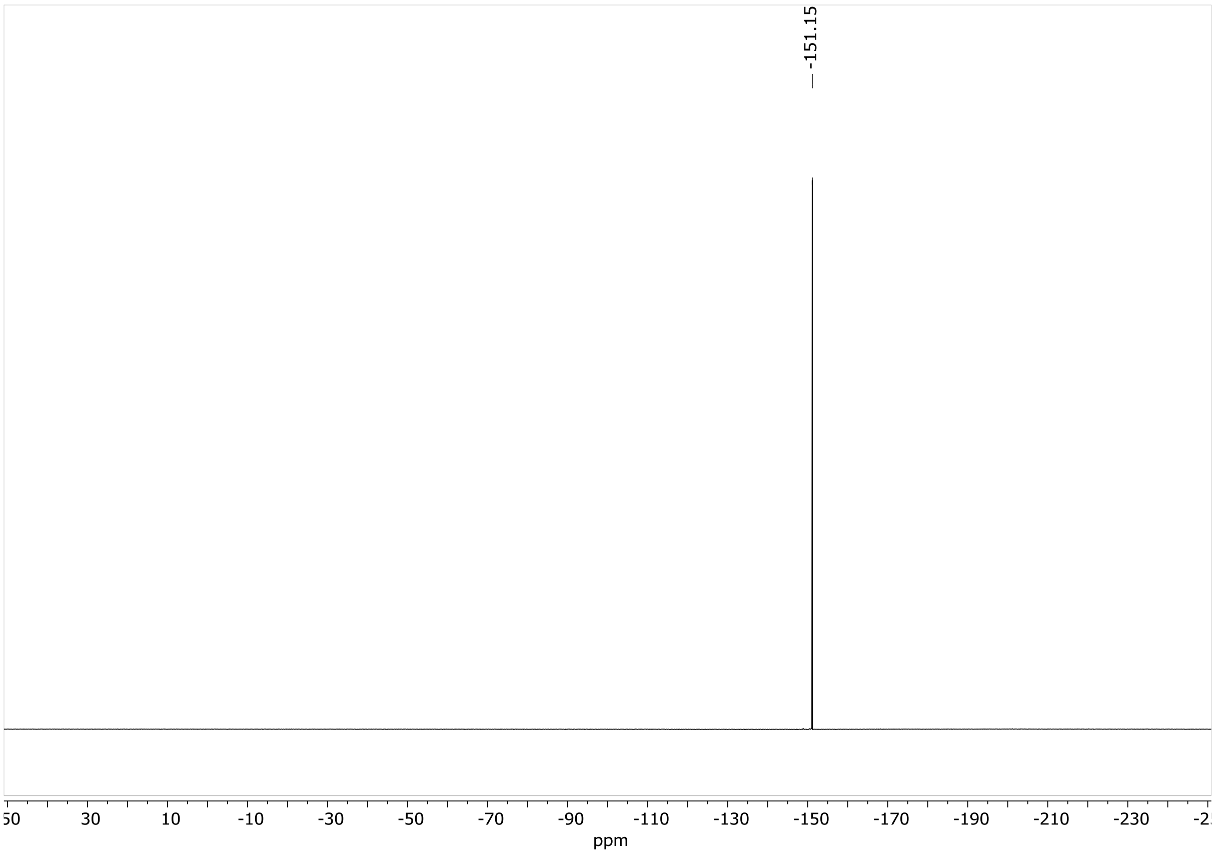
-151.15
50
30
10
-10
-30
-50
-70
-90
-110
-130
-150
-170
-190
-210
-230
ppm

^{1}H NMR spectra of compound **2a-TMS**, observation of continuous degradation

crude NMR after workup:

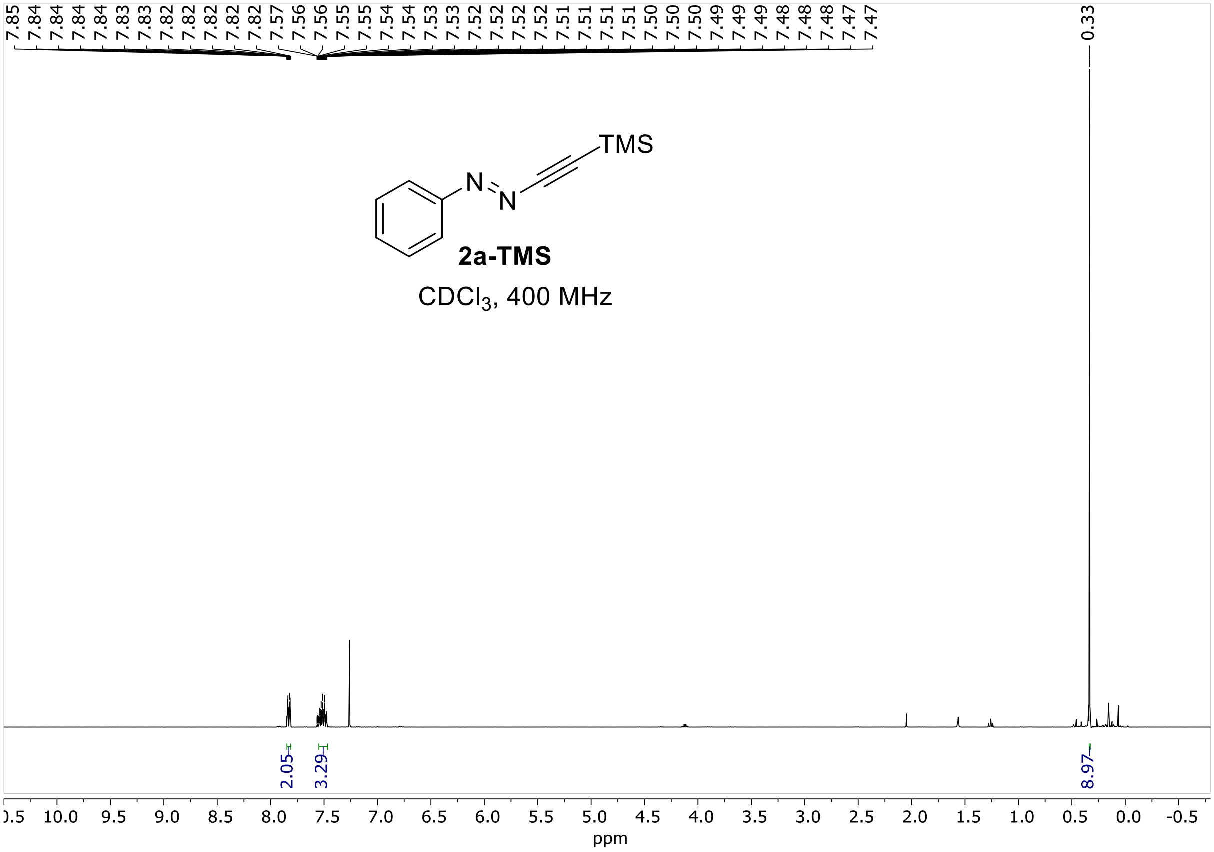

directly after column chromatography (SiO_2, 20% CH_2Cl_2 in hexanes)

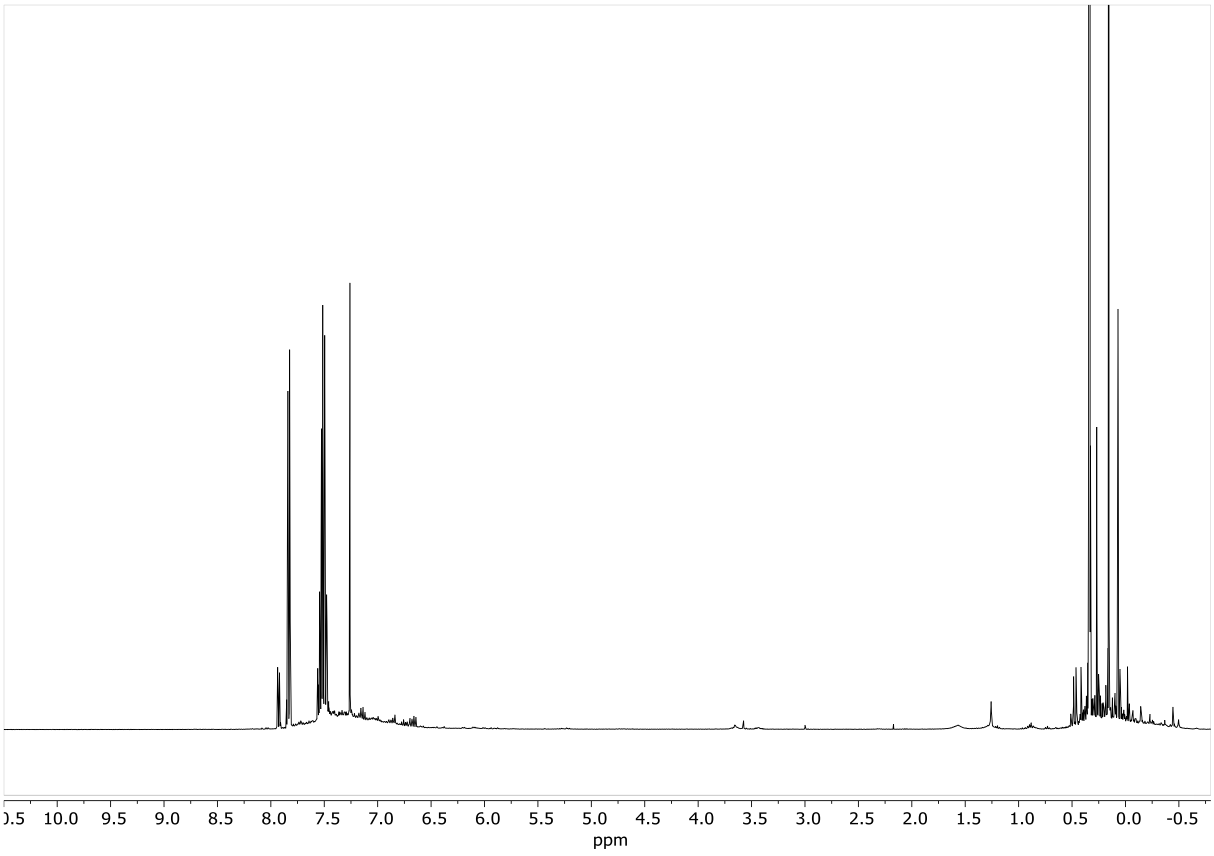

columned material after resting at room temperature overnight:

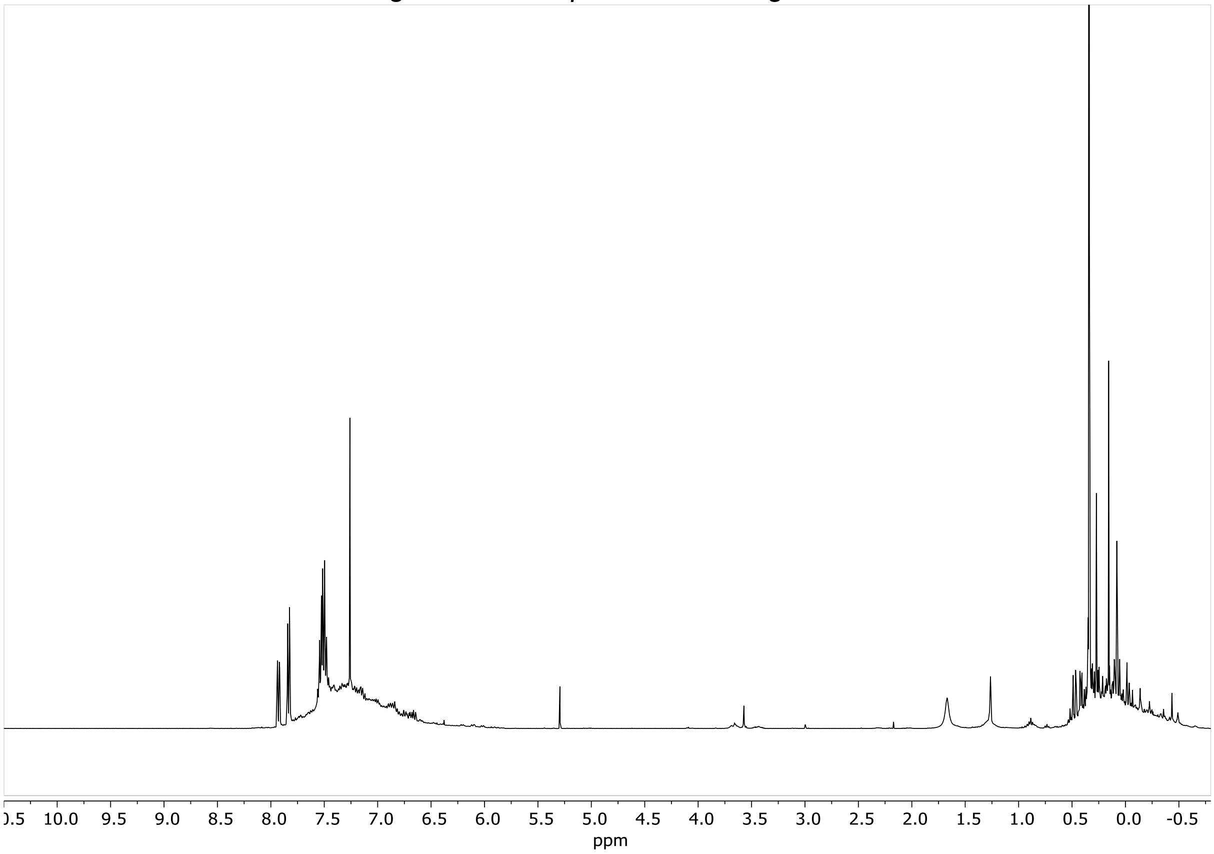

^{1}H NMR and ^{13}C NMR spectra of compound **2a**

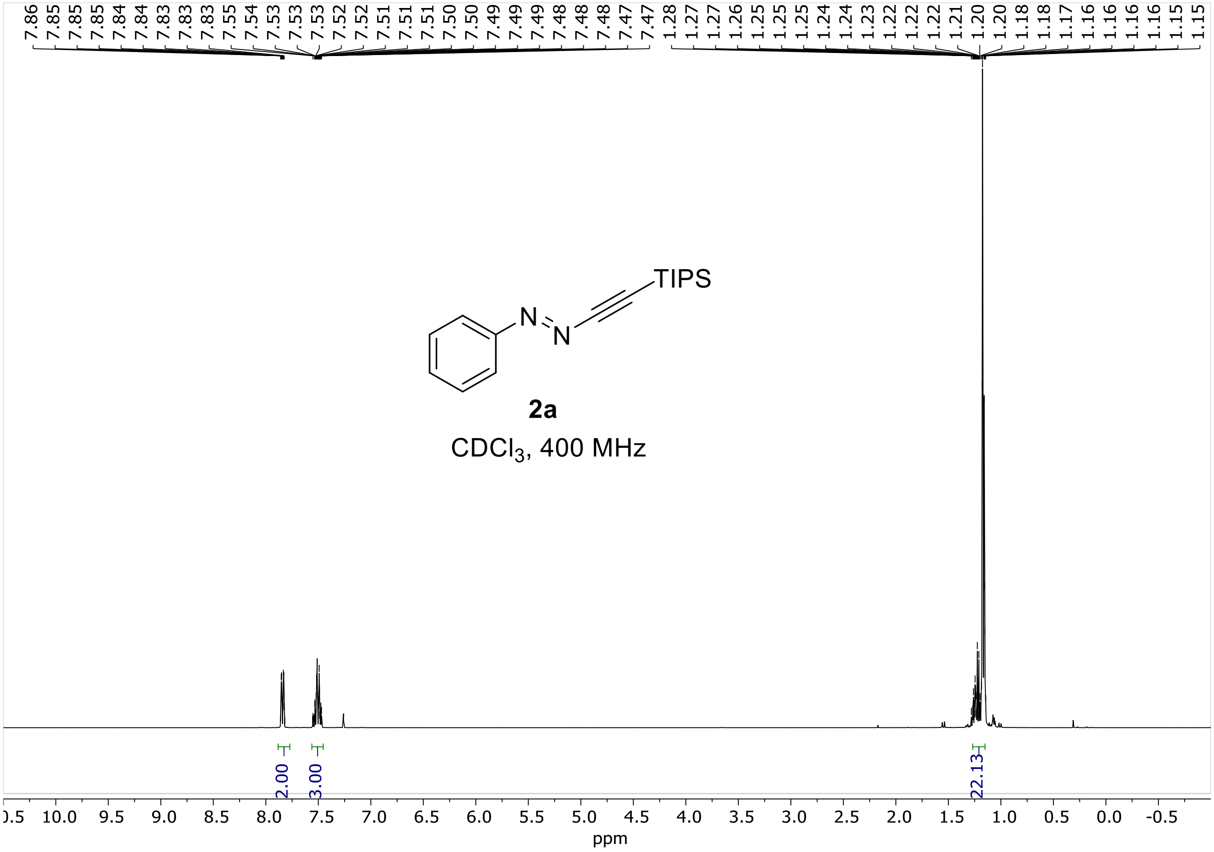

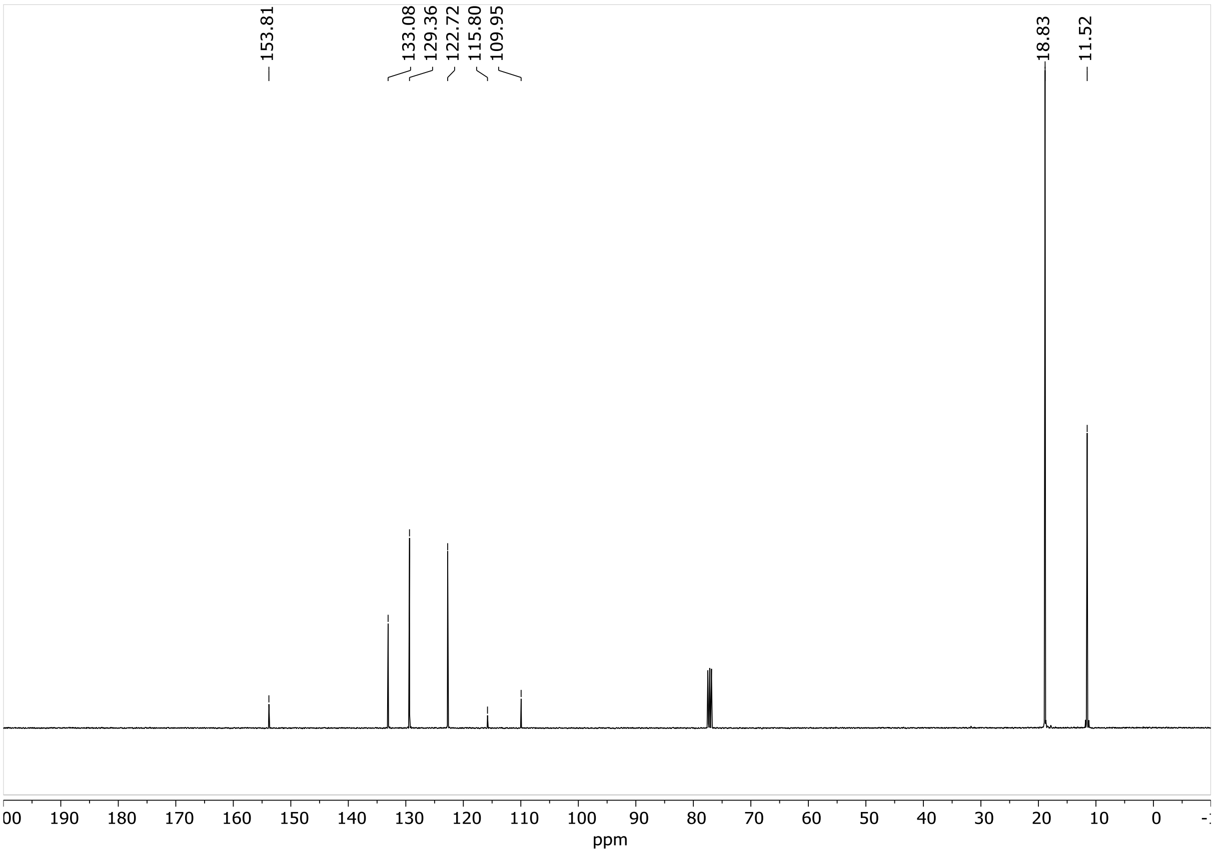

^{1}H-NMR of a sample of **2a** which was stored for 12 months at room temperature, sample contains residual amount of CH_2Cl_2 and about 10% TIPS-acetylene (mixed column fraction of a formerly unoptimized batch).

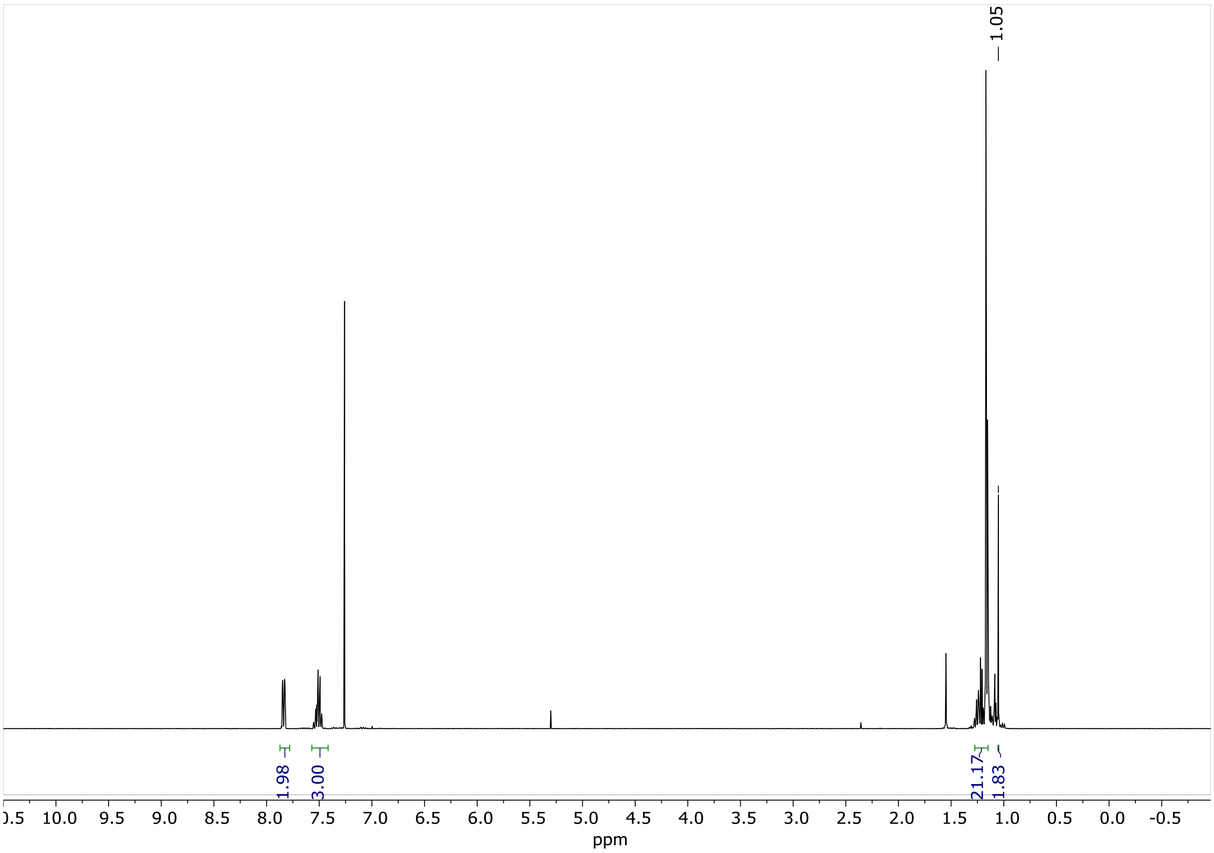

^{1}H NMR and ^{13}C NMR spectra of compound **2b**

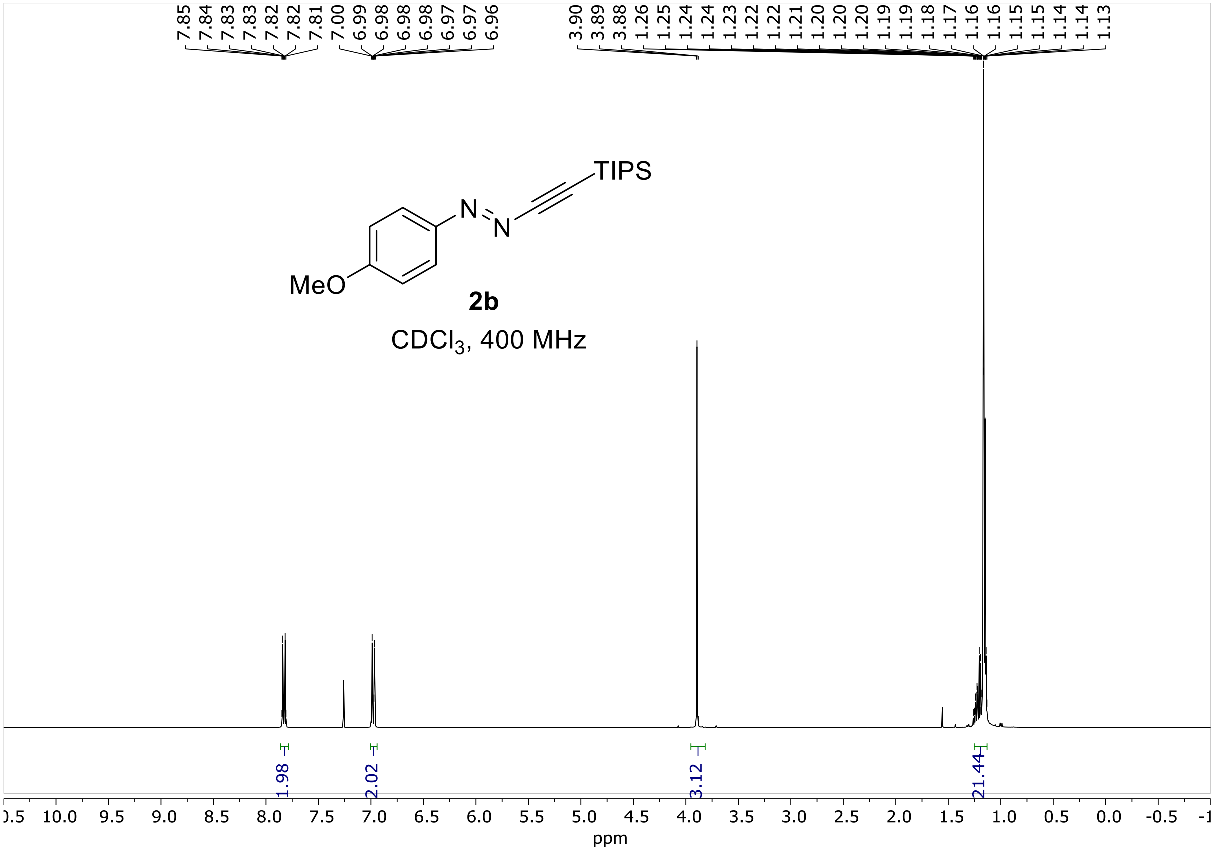

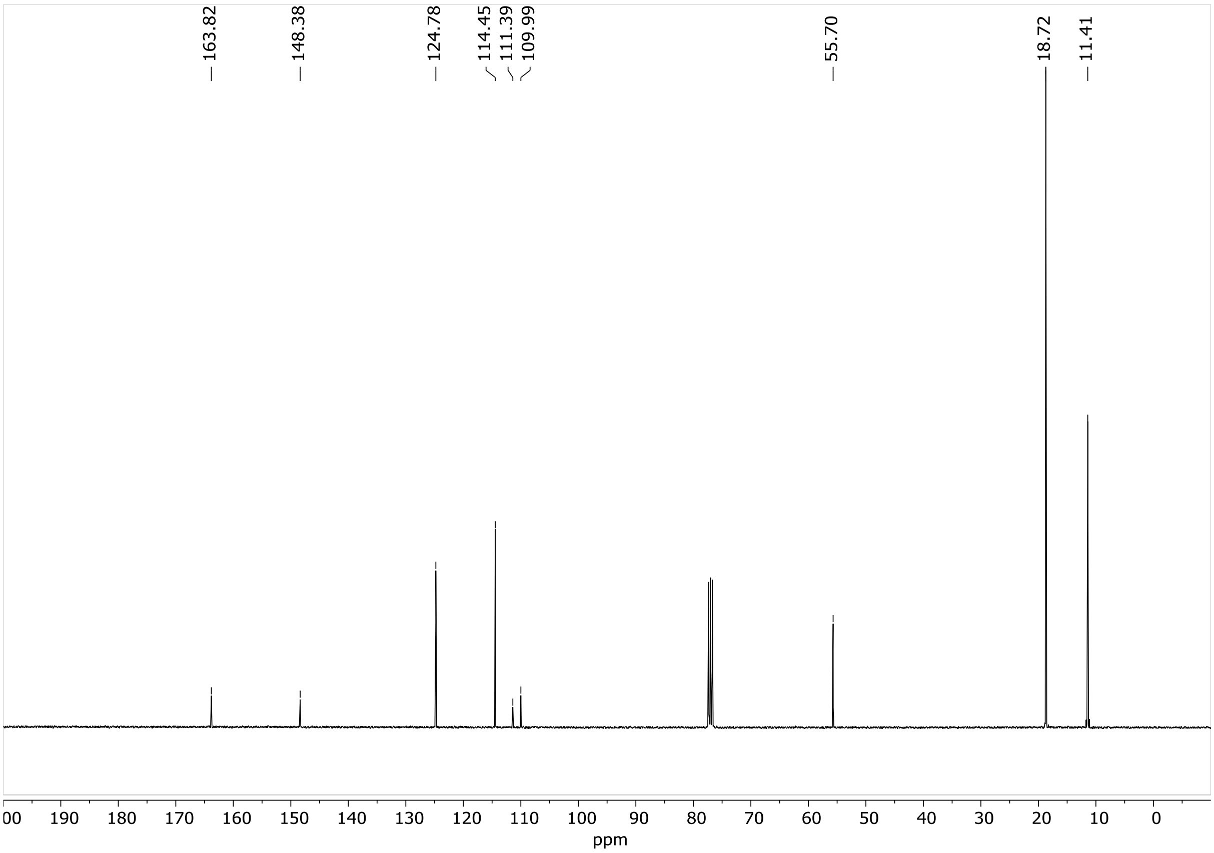

^{1}H NMR and ^{13}C NMR spectra of compound **2c**

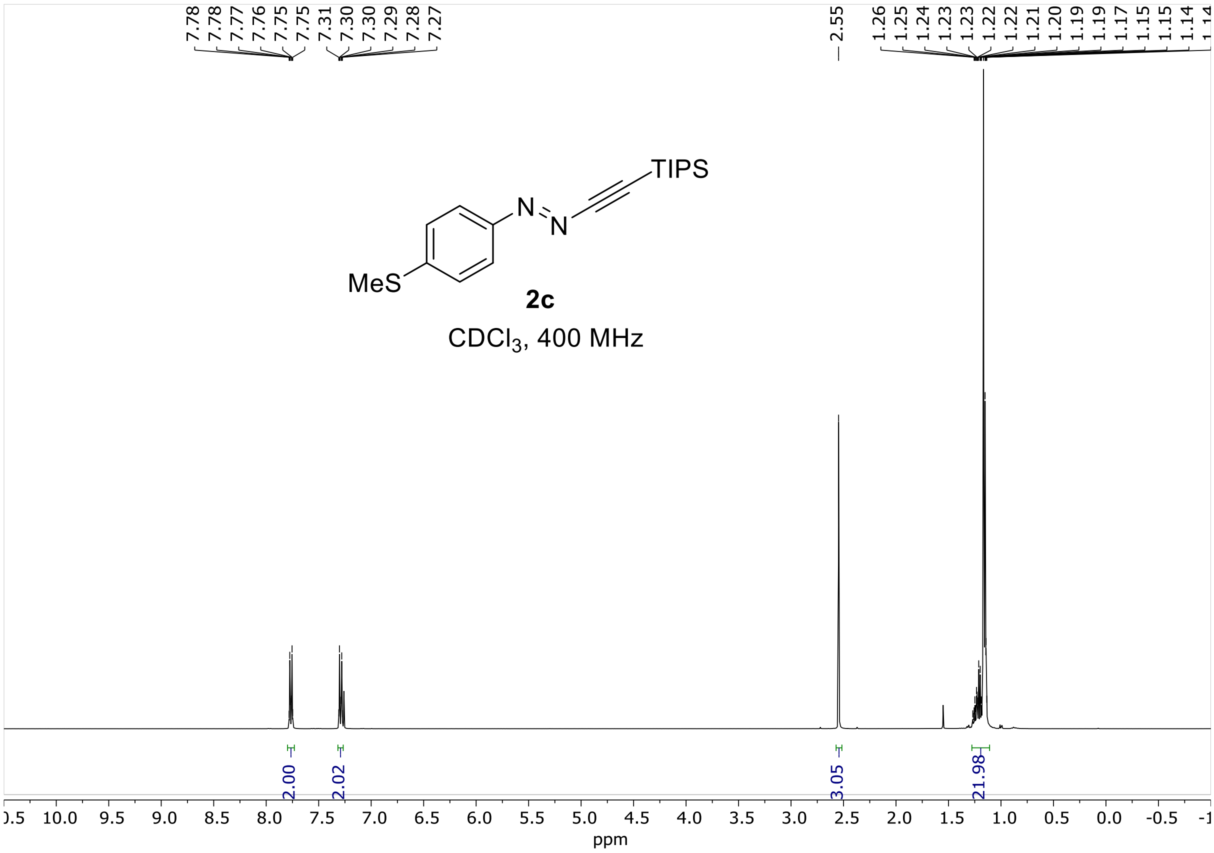

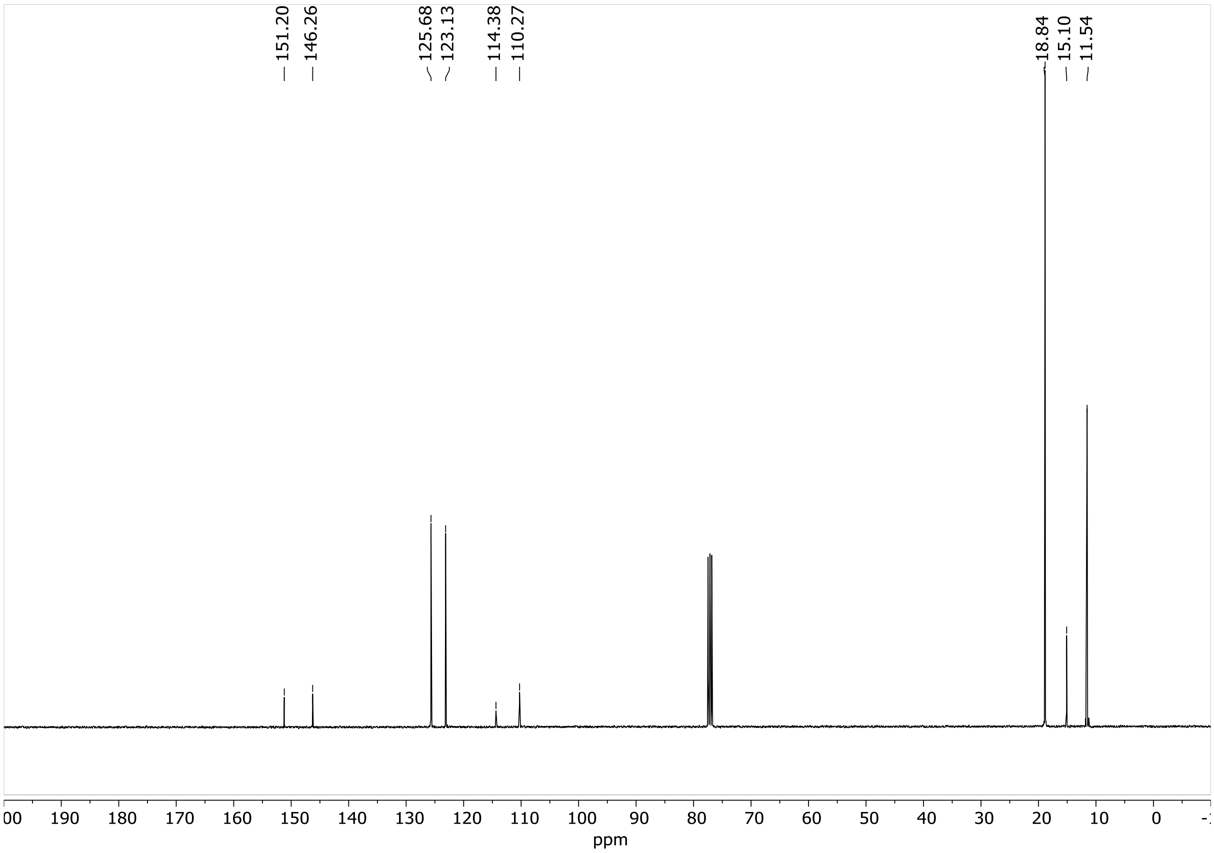

^{1}H NMR and ^{13}C NMR spectra of compound **2d**

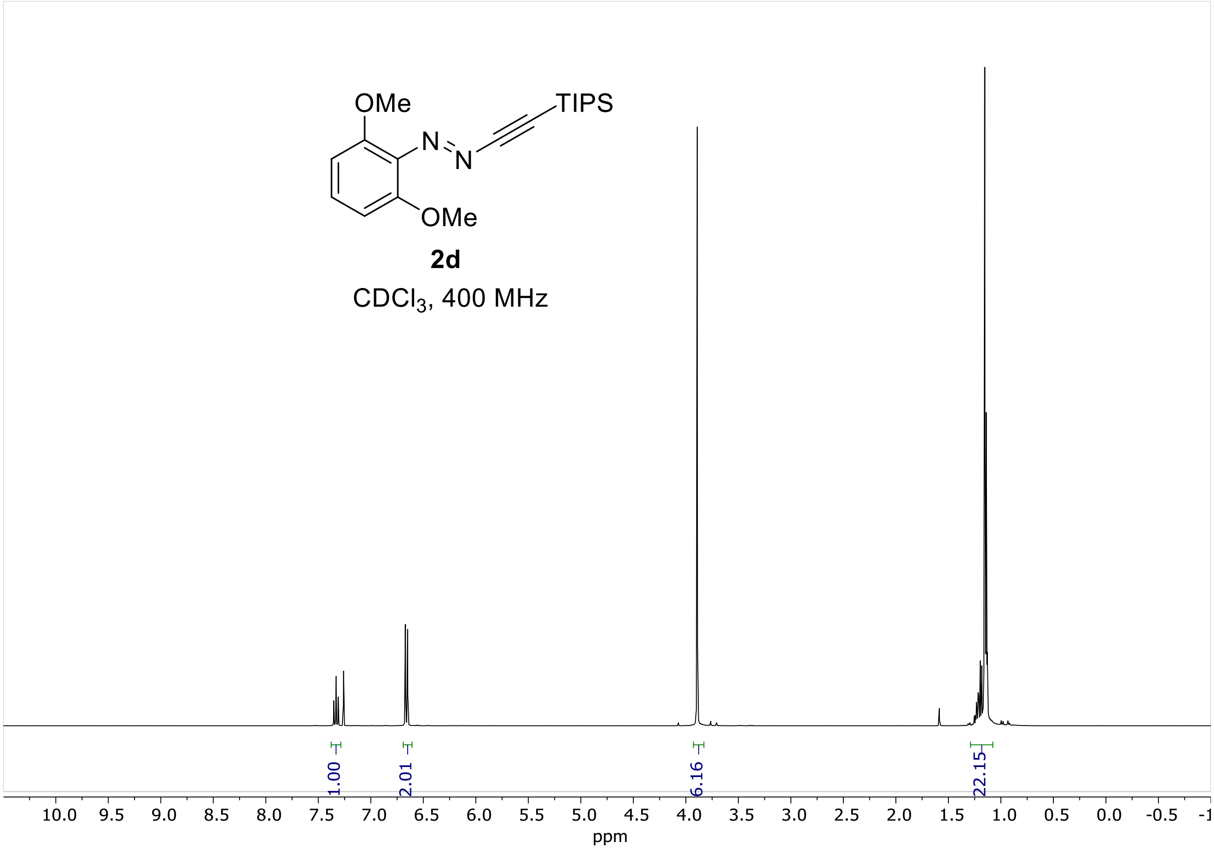

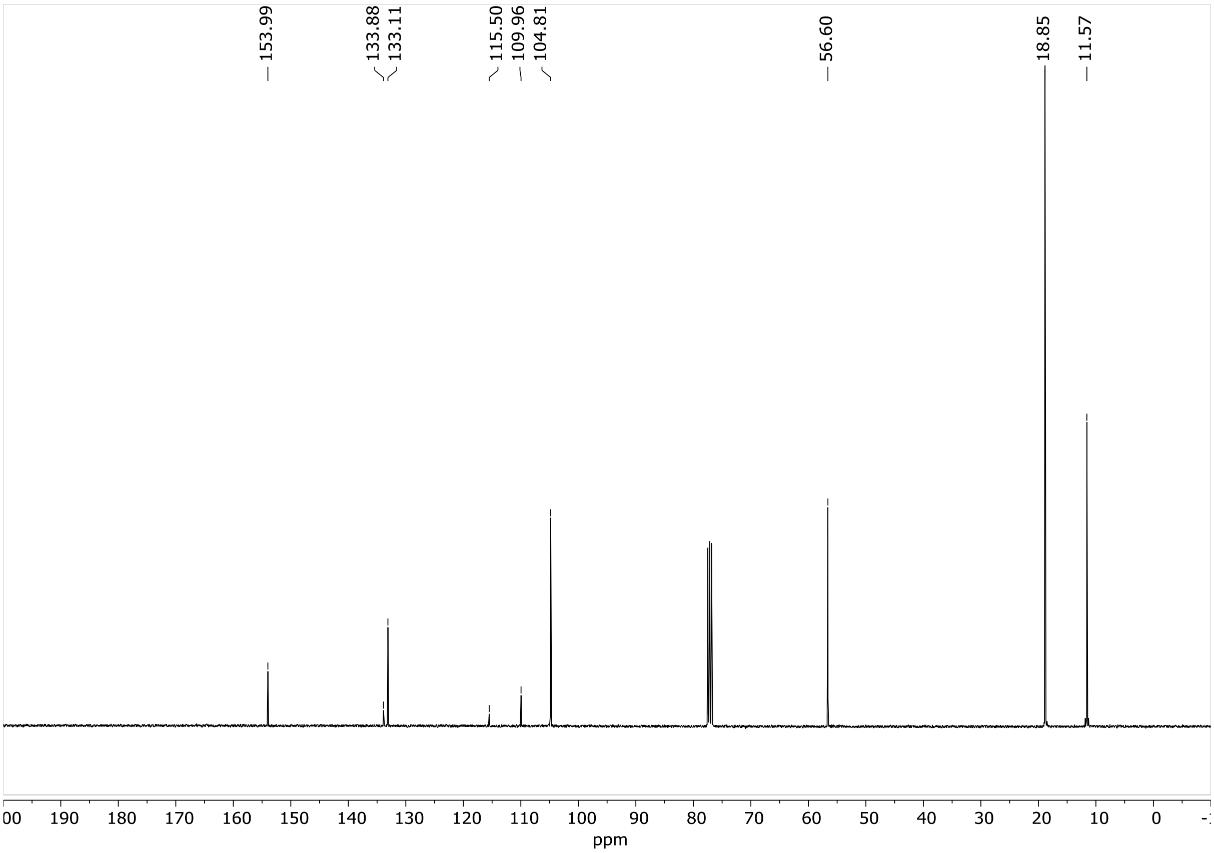

^{1}H NMR and ^{13}C NMR spectra of compound **2e**

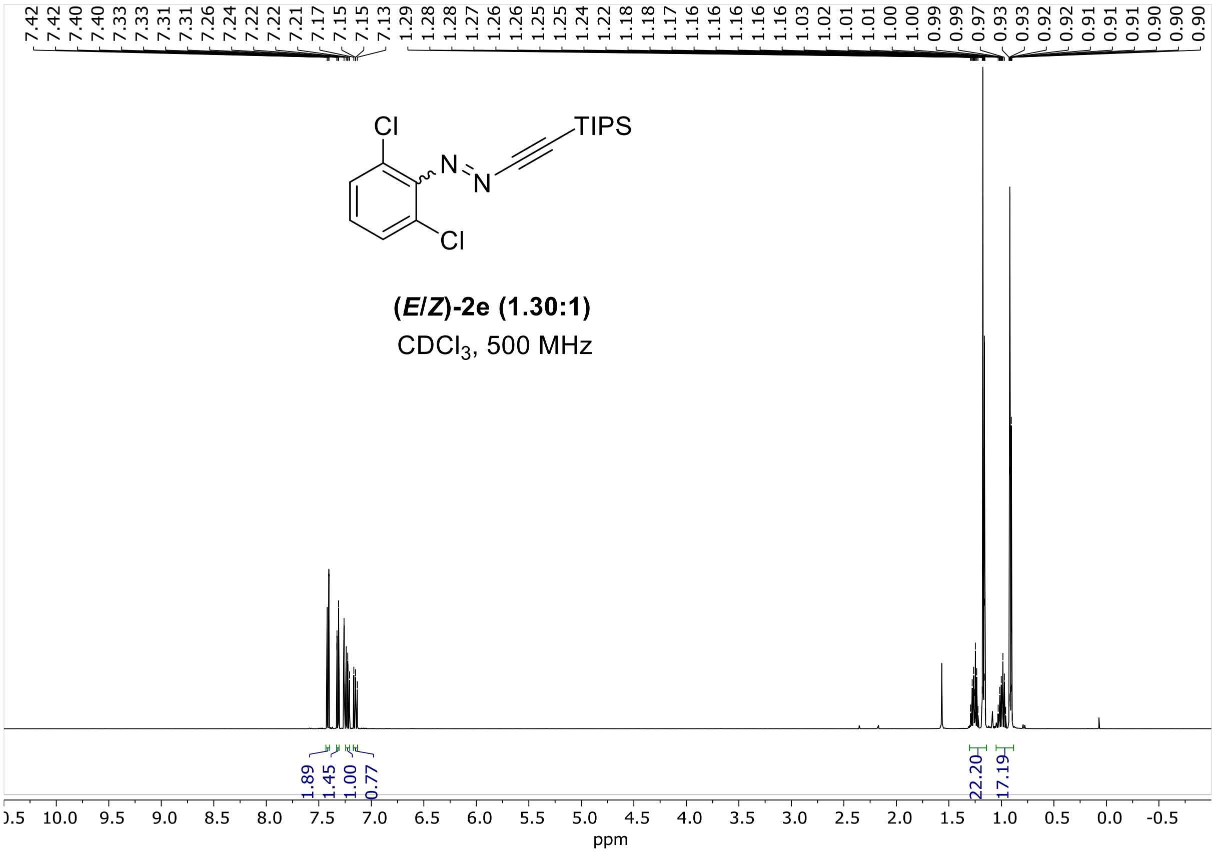

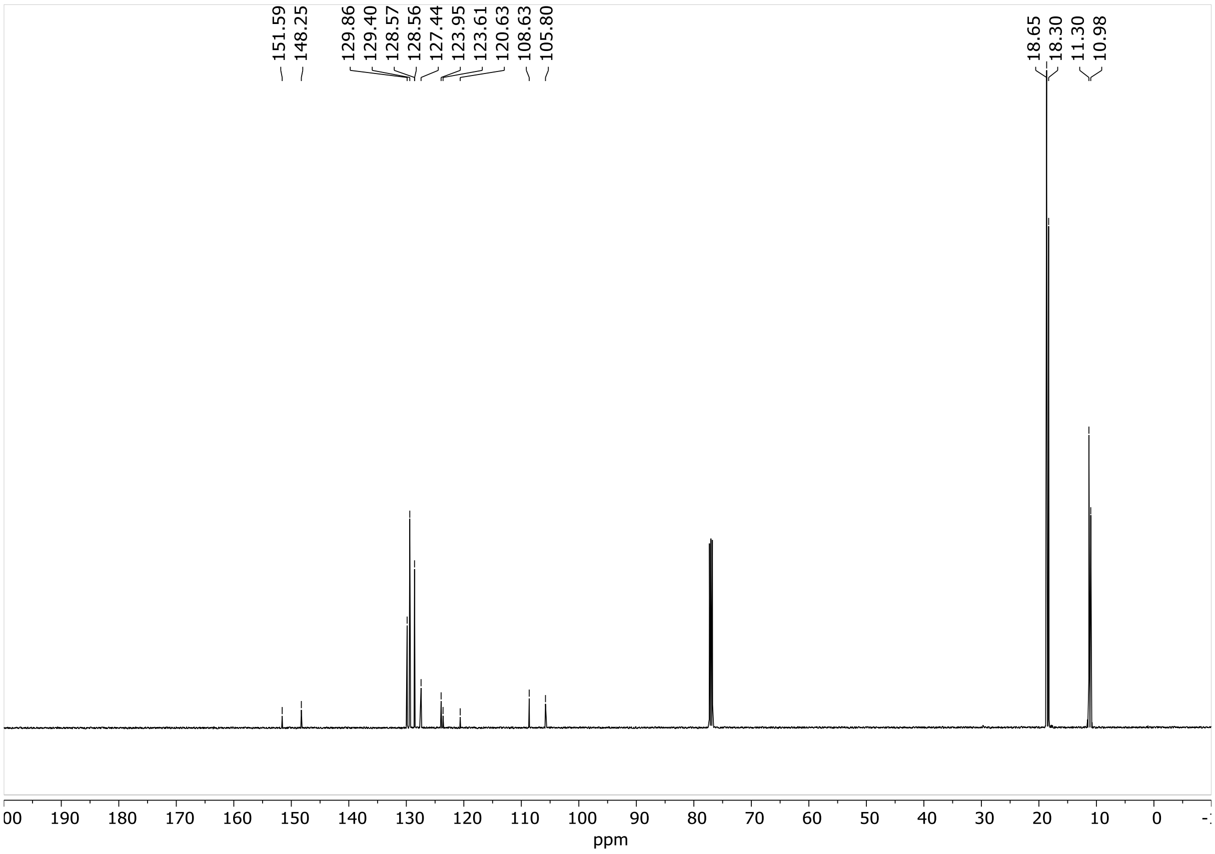

^{1}H NMR, ^{13}C NMR and ^{19}F NMR spectra of compound **2f**

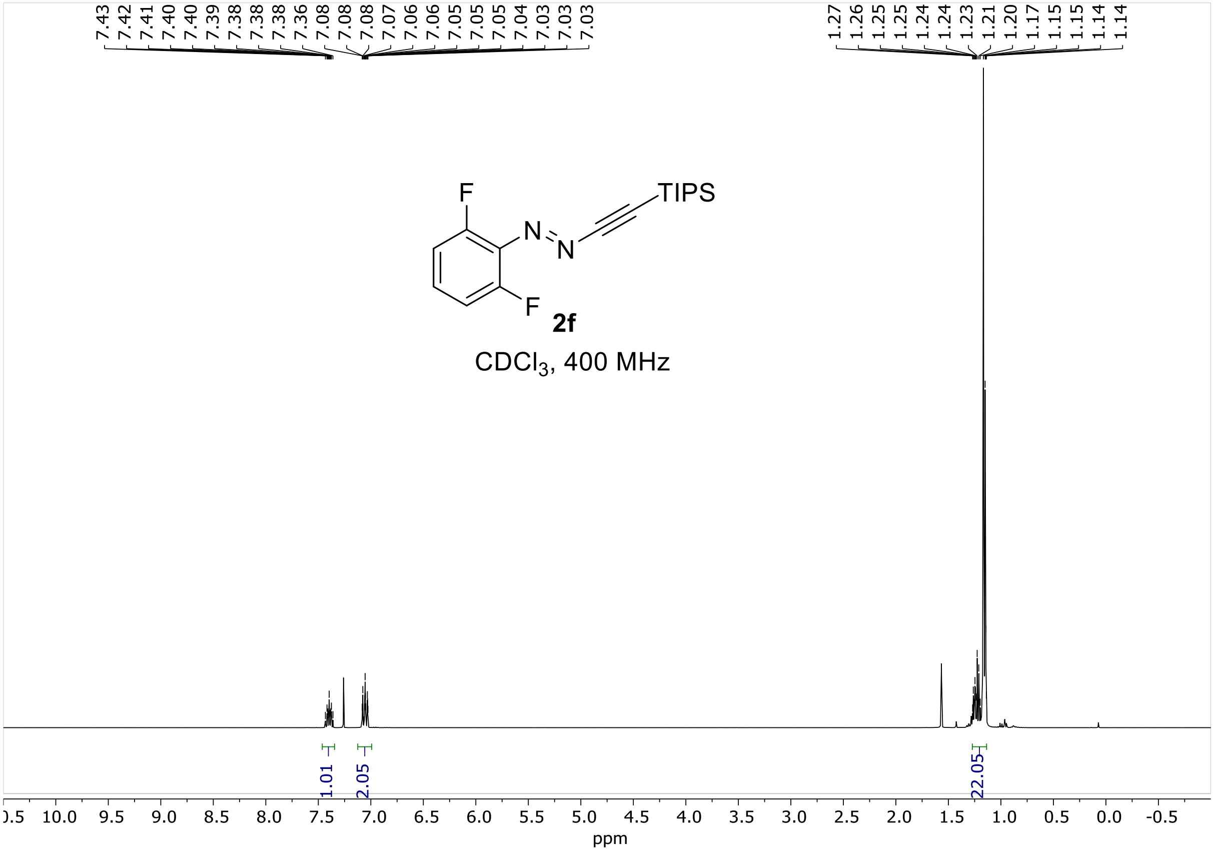

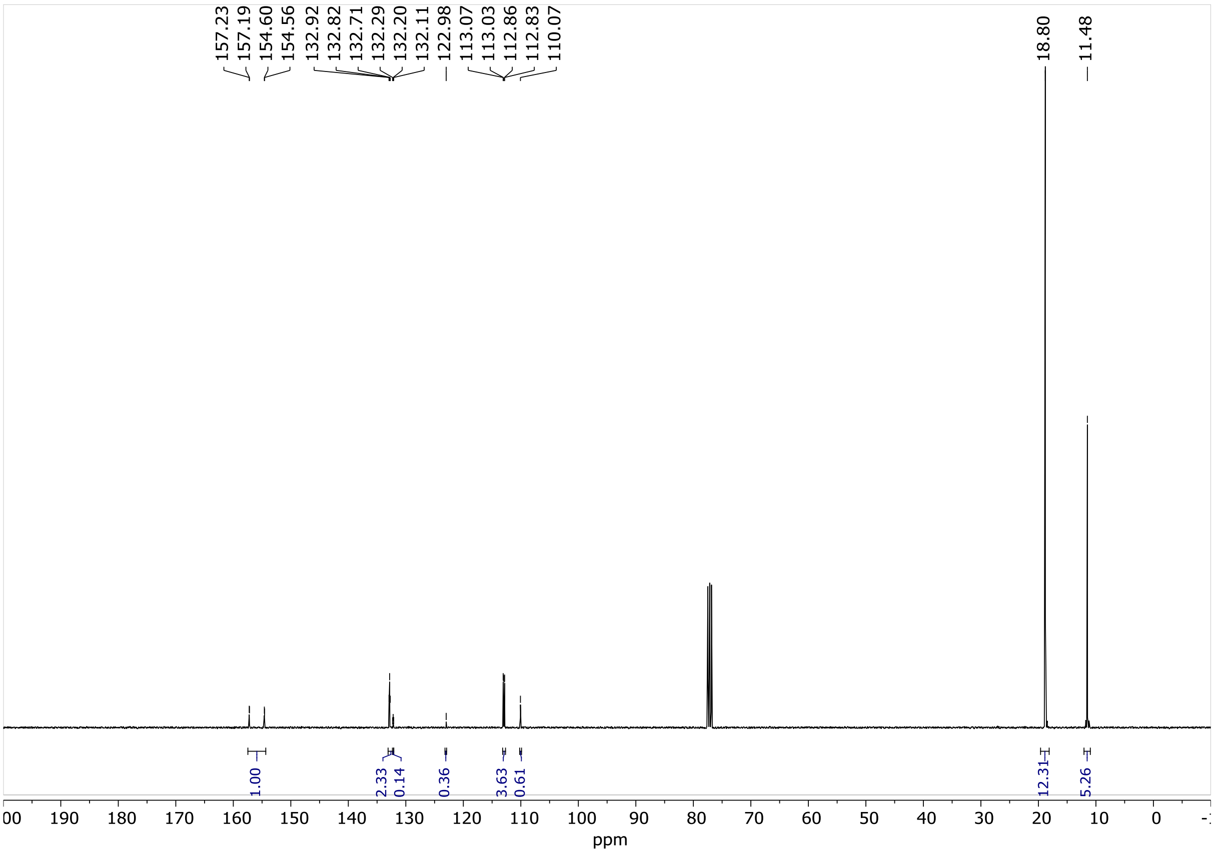

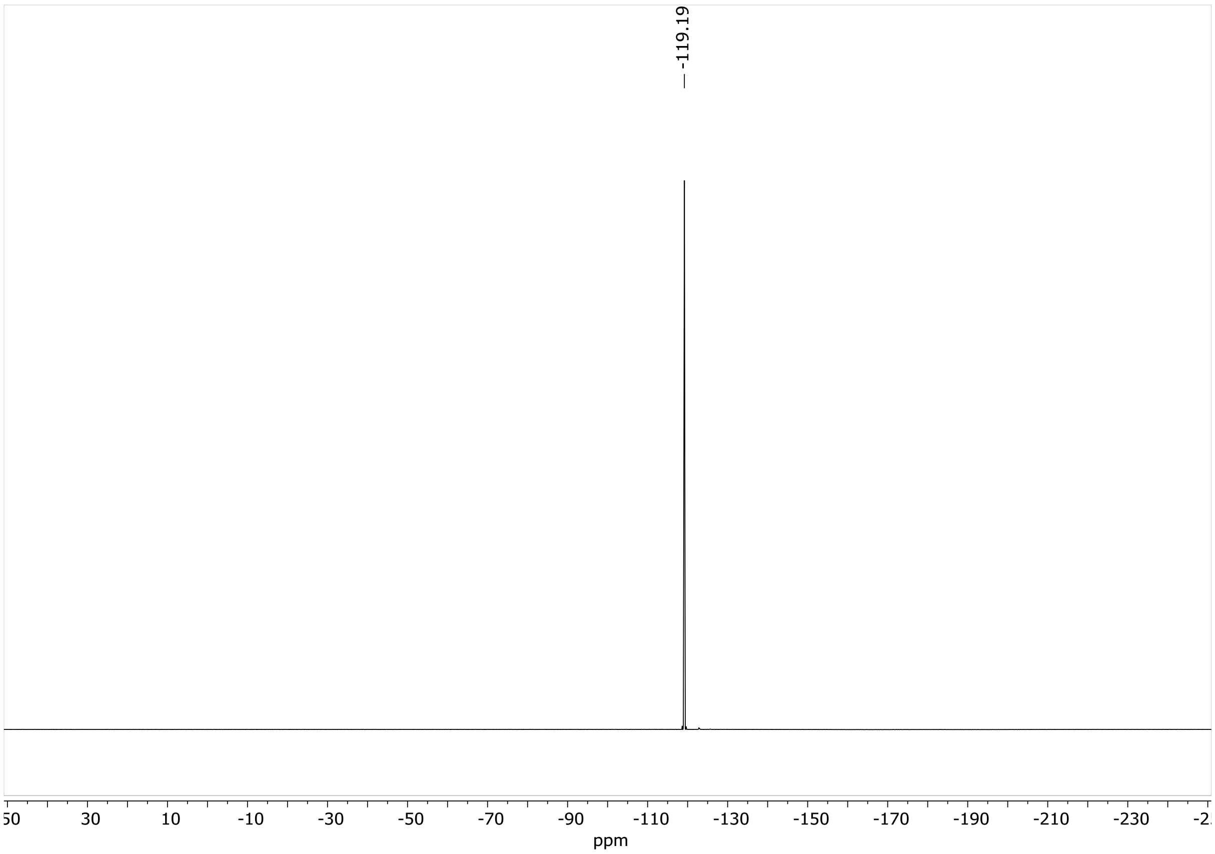

-119.19
ppm

^{1}H NMR and ^{13}C NMR spectra of compound **2g**

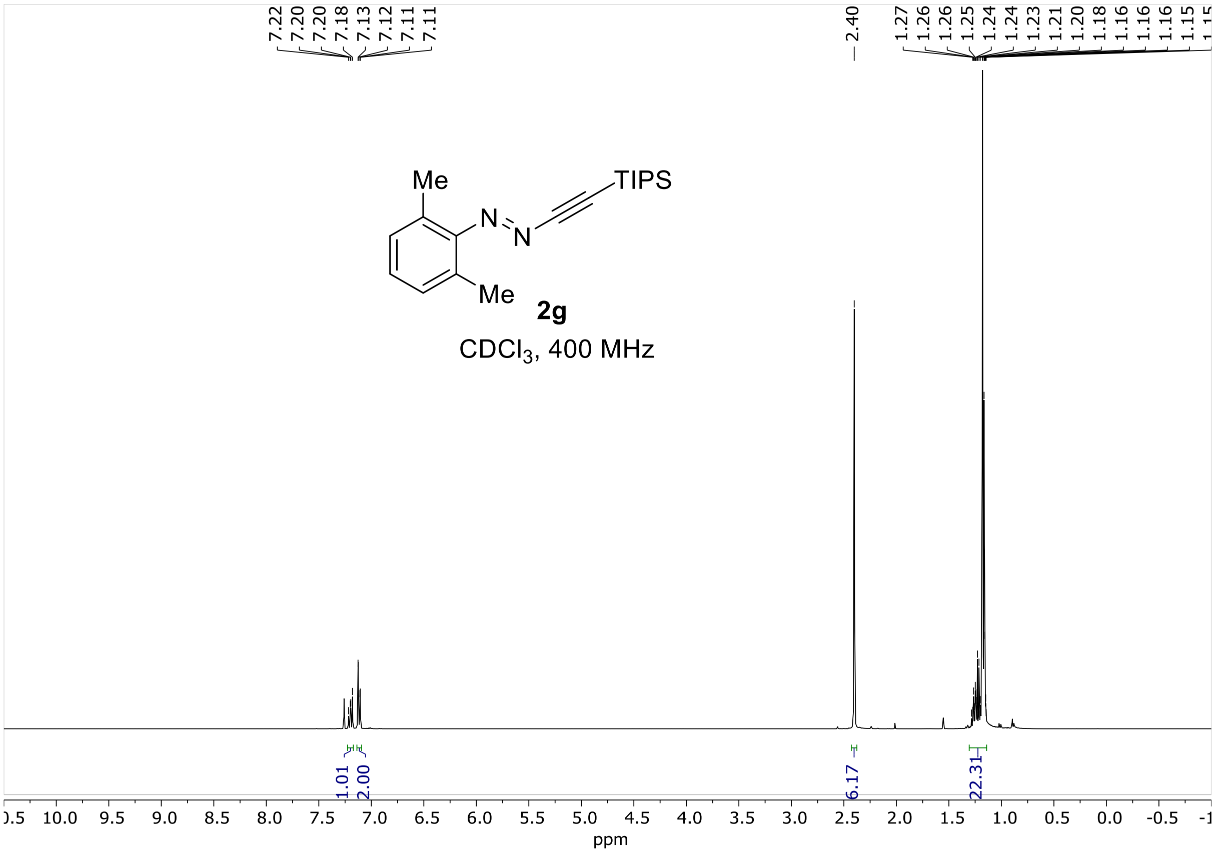

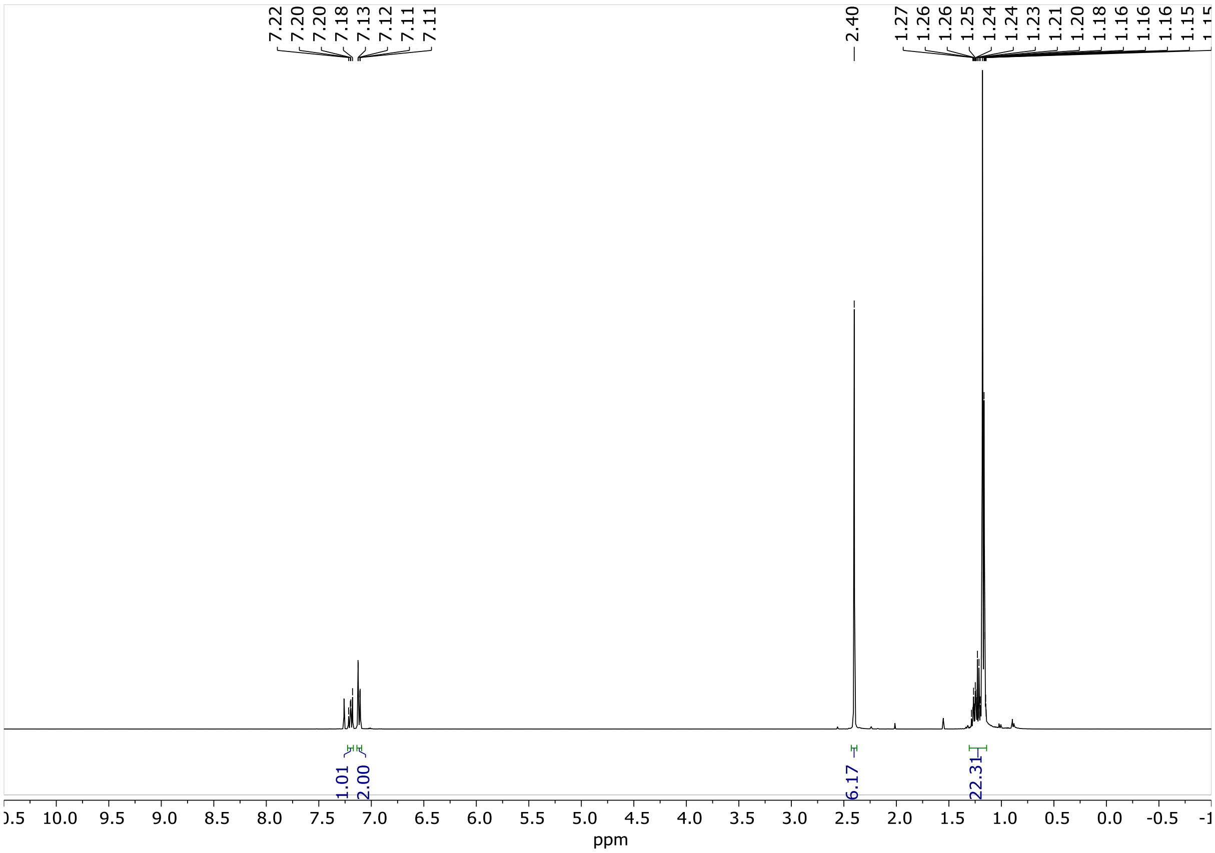

^{1}H NMR and ^{13}C NMR spectra of compound **2h**

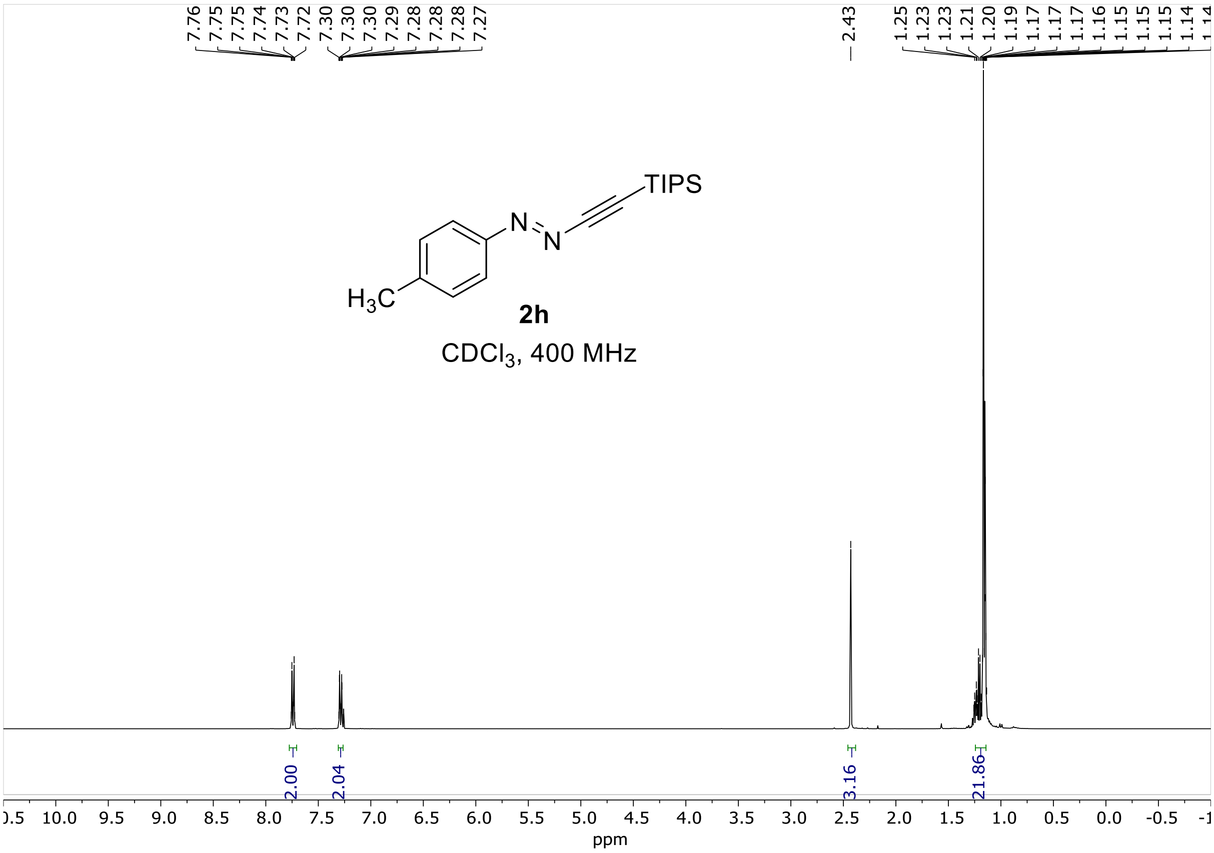

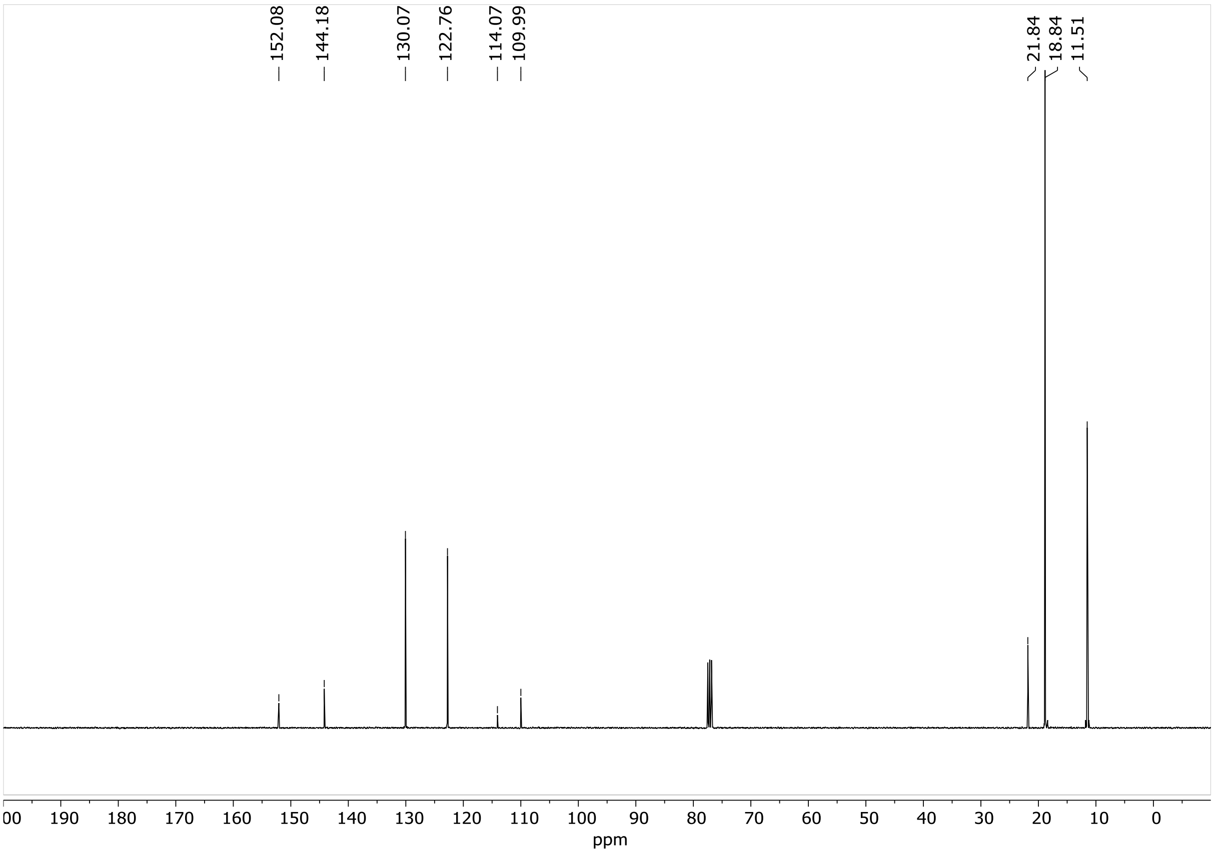

^{1}H NMR, ^{13}C NMR and ^{19}F NMR spectra of compound **2i**

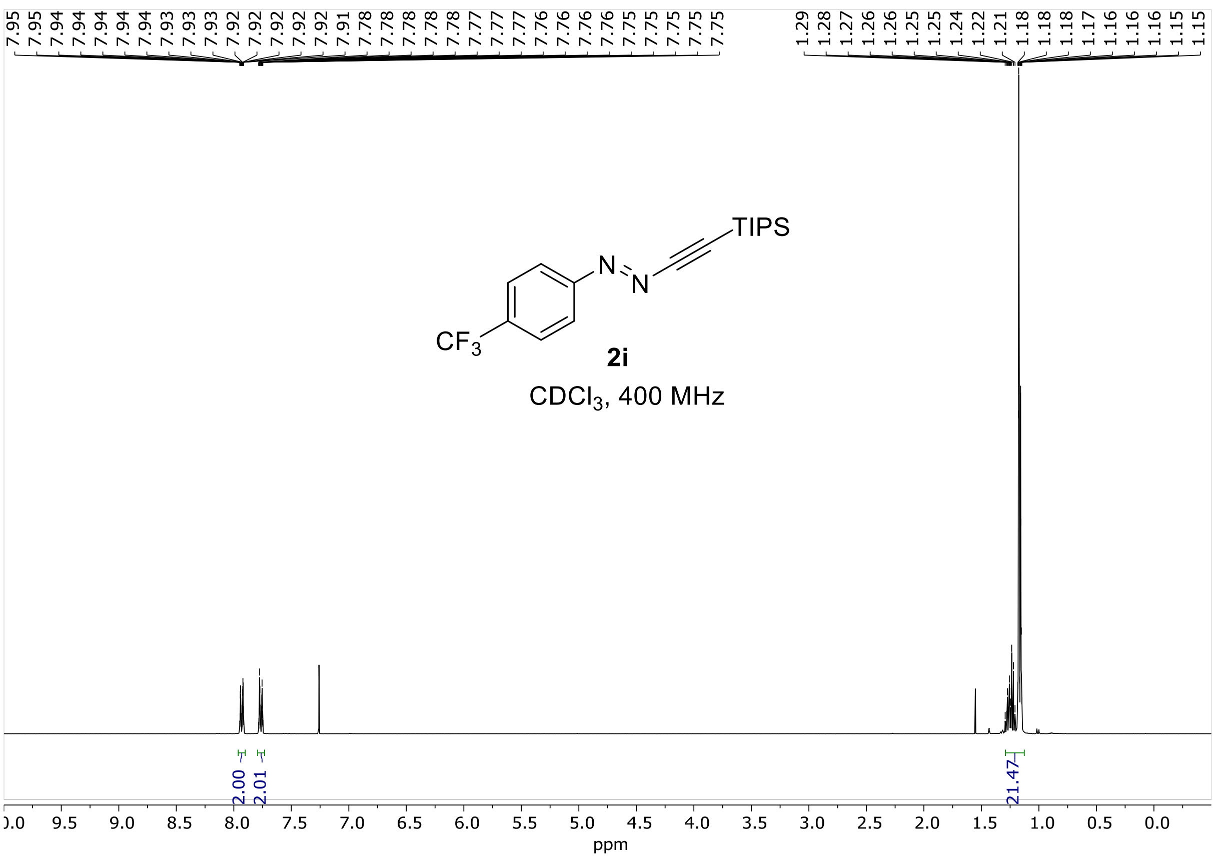

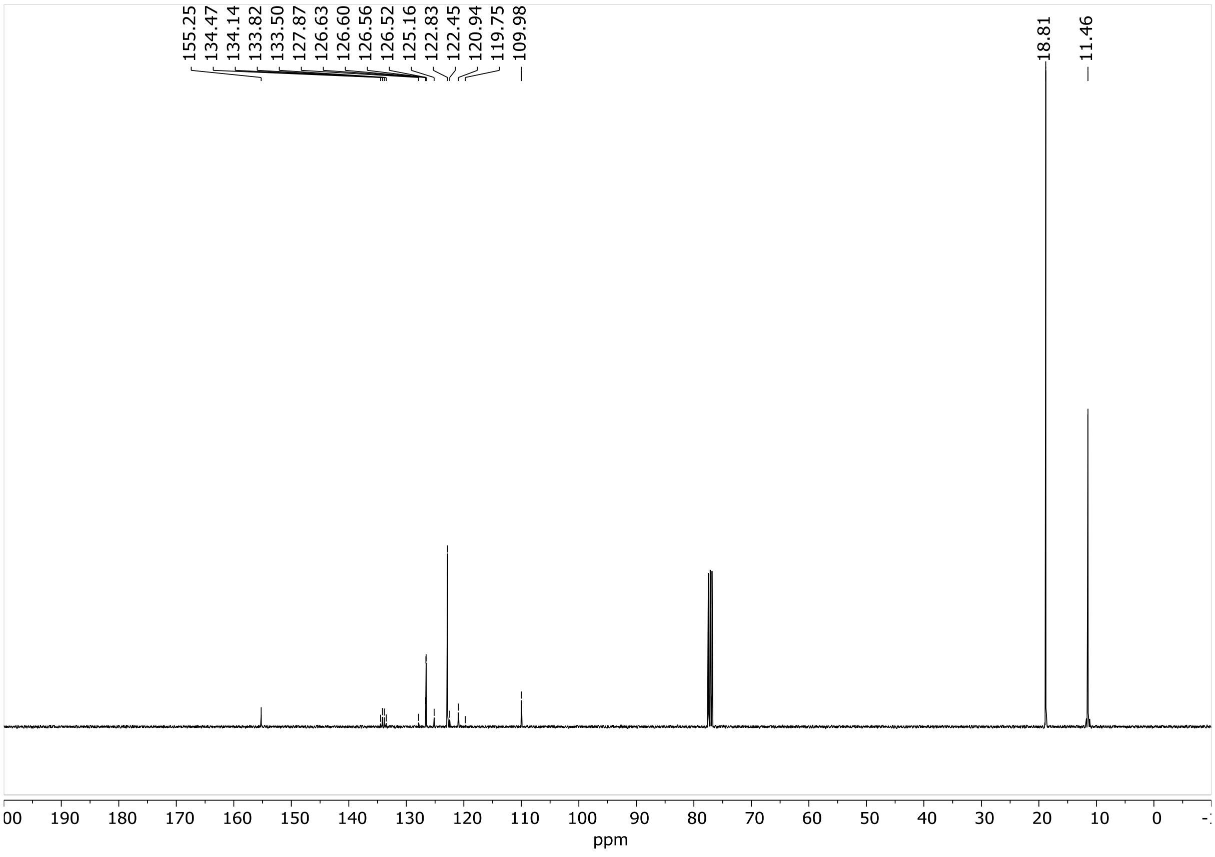

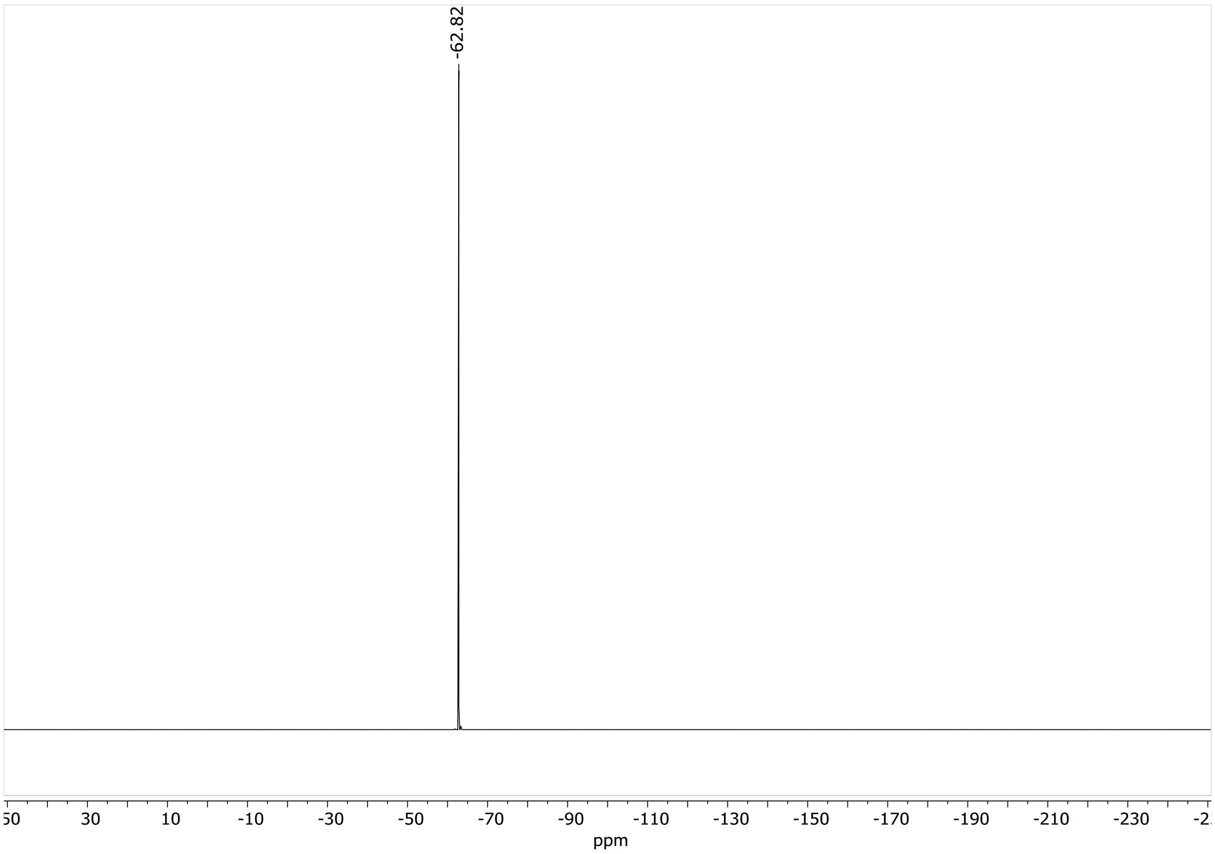
-62.82
50
30
10
-10
-30
-50
-70
-90
-110
-130
-150
-170
-190
-210
-230
ppm

^{1}H NMR and ^{13}C NMR spectra of compound **2j**

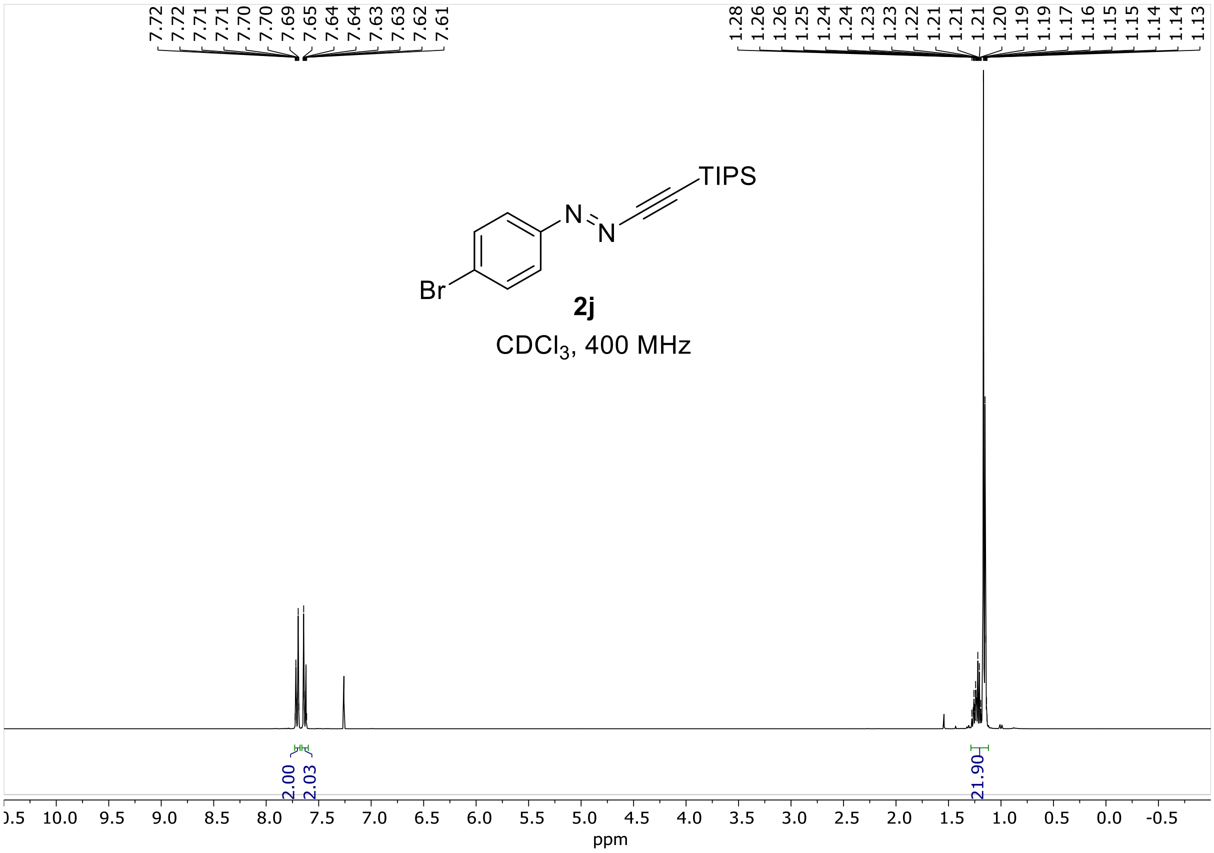

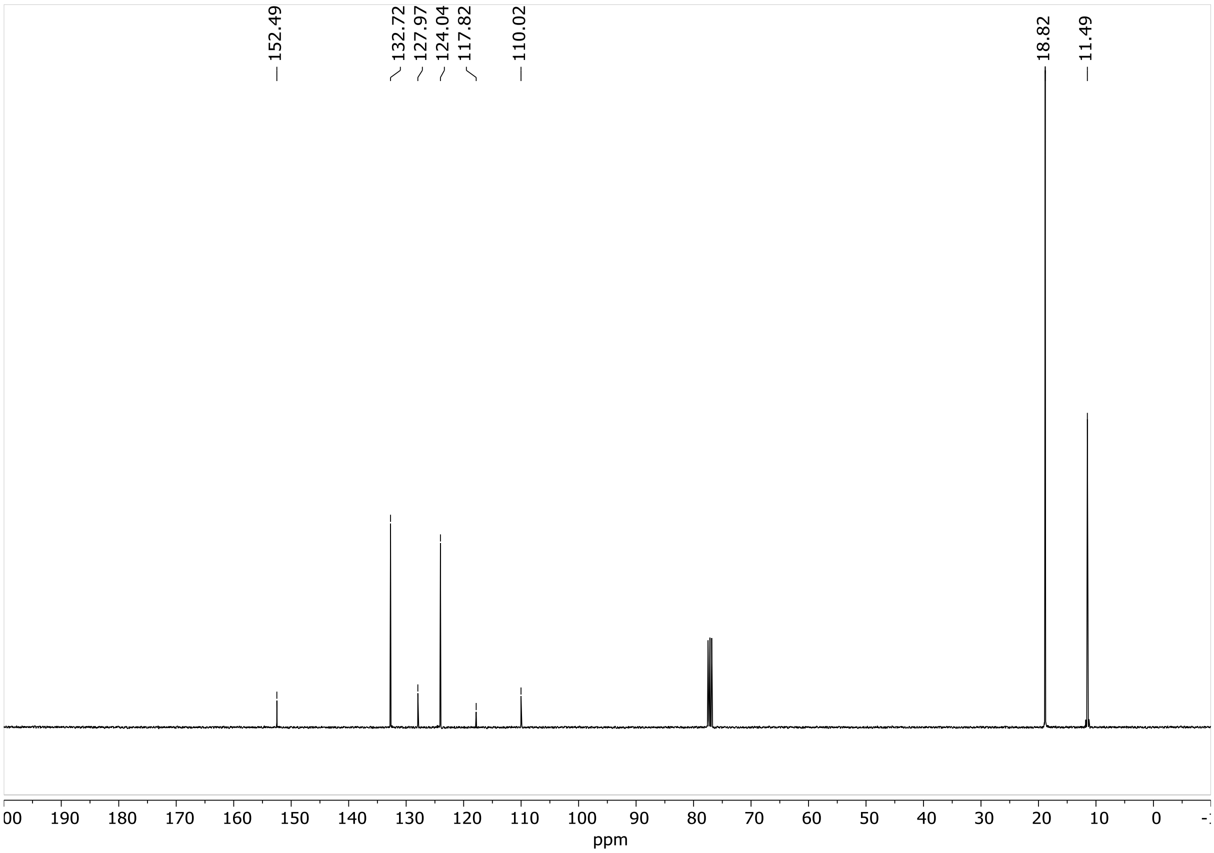

^{1}H NMR and ^{13}C NMR spectra of compound **2k**

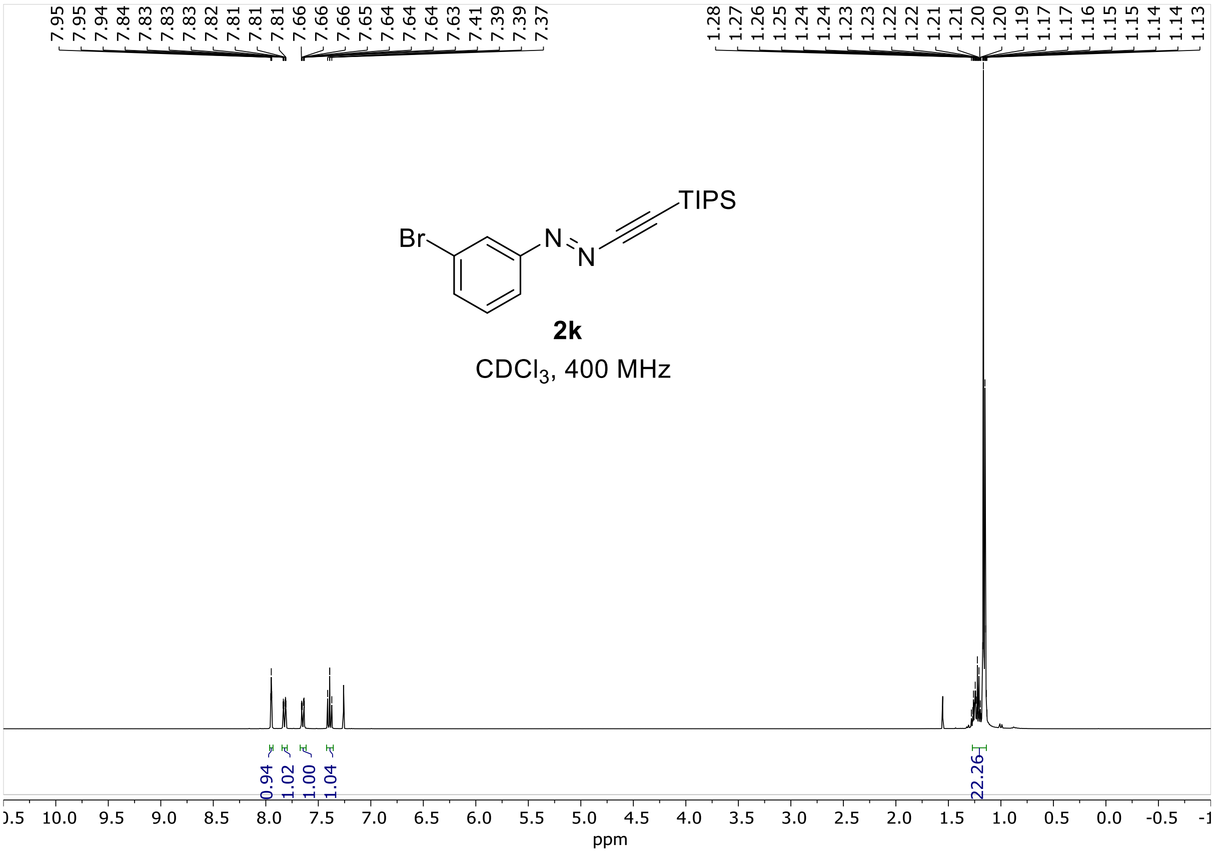

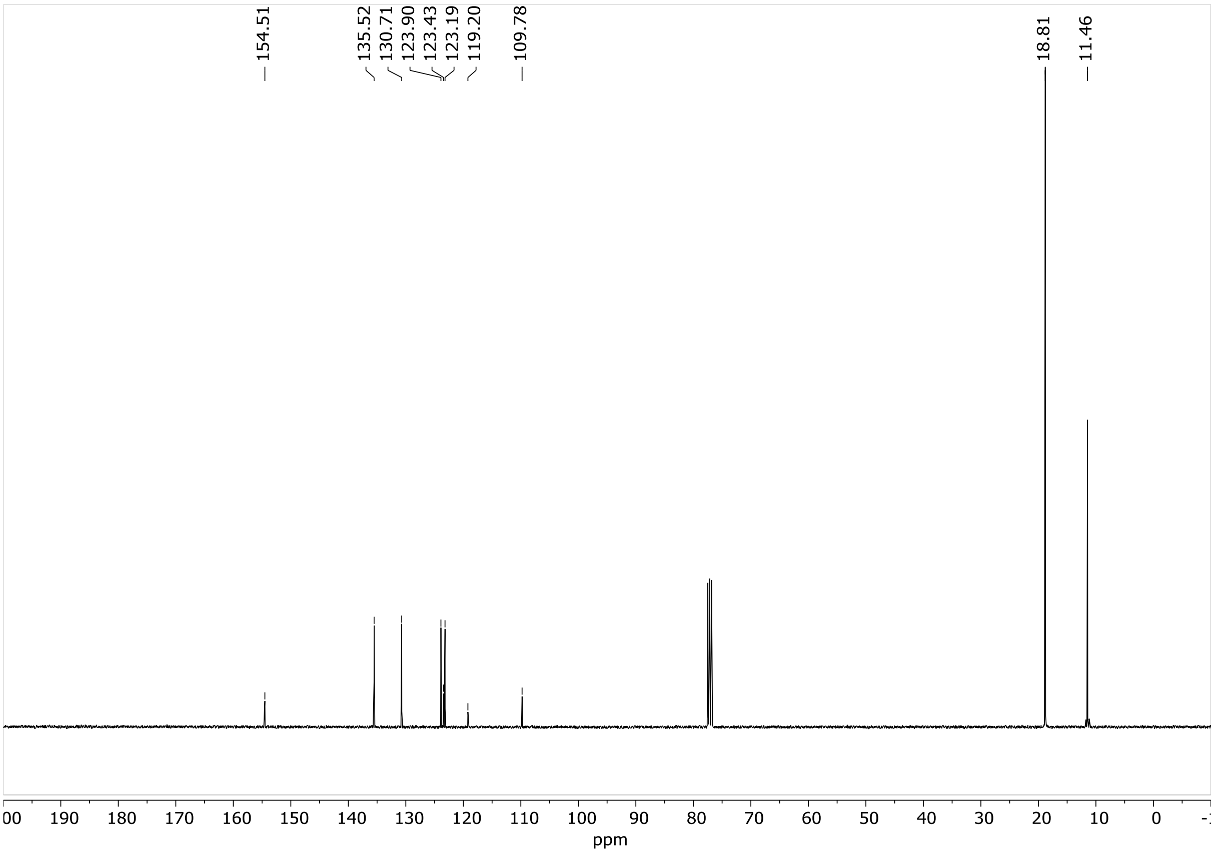

^{1}H NMR and ^{13}C NMR spectra of compound **2l**

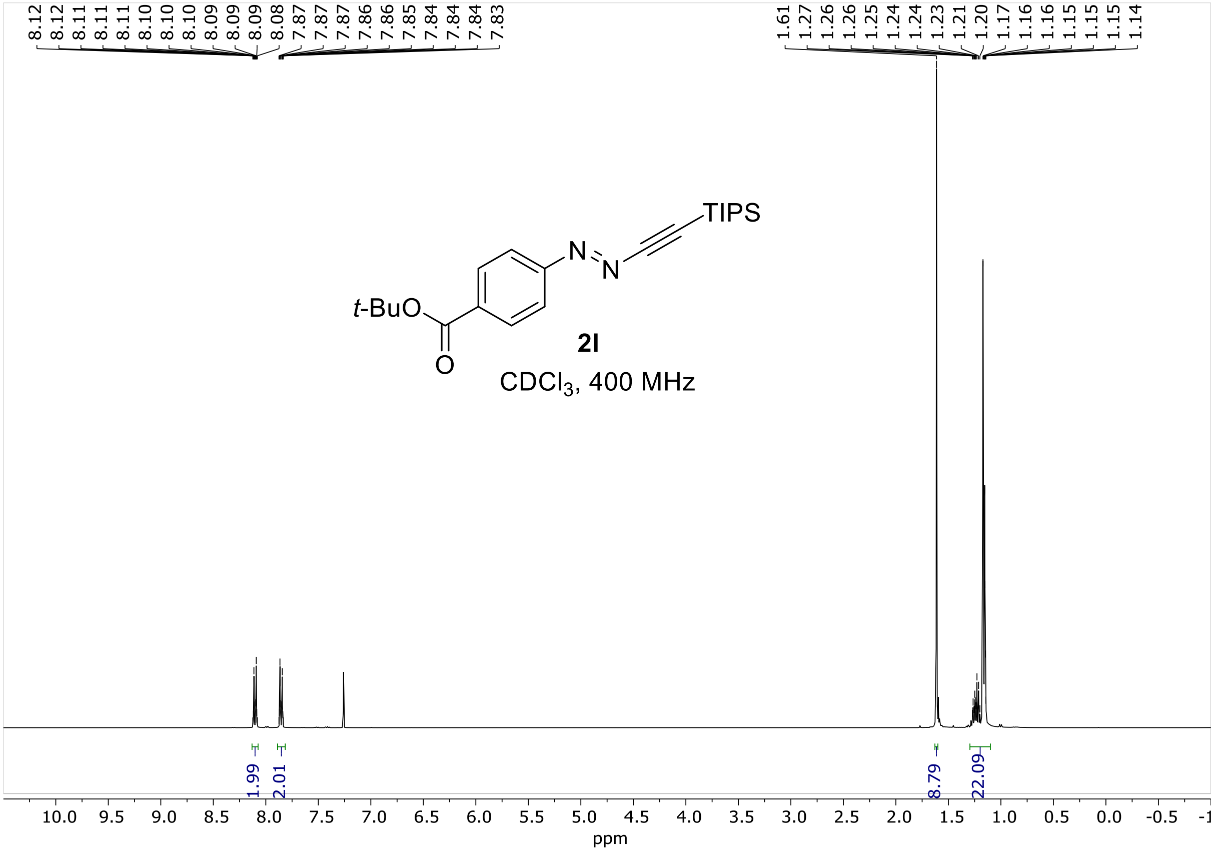

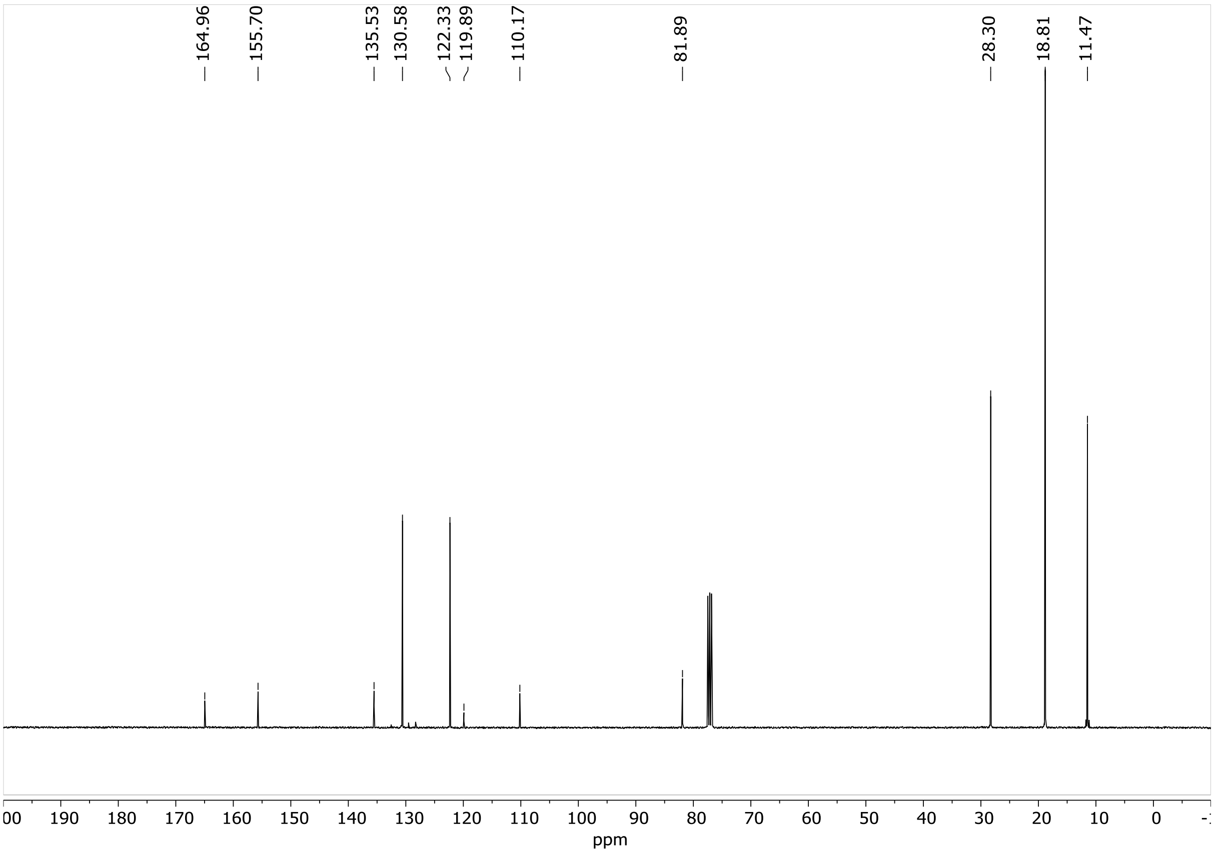

^{1}H NMR and ^{13}C NMR spectra of compound **2m**

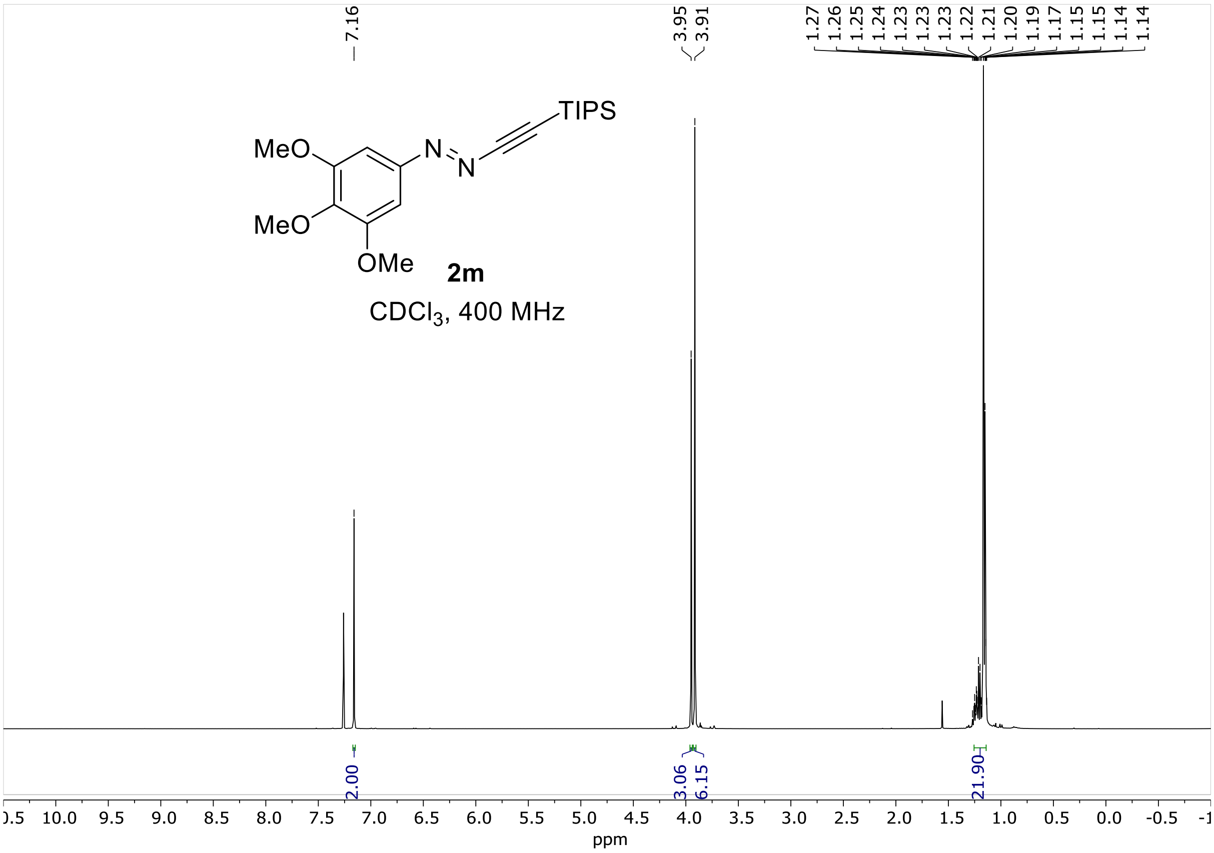

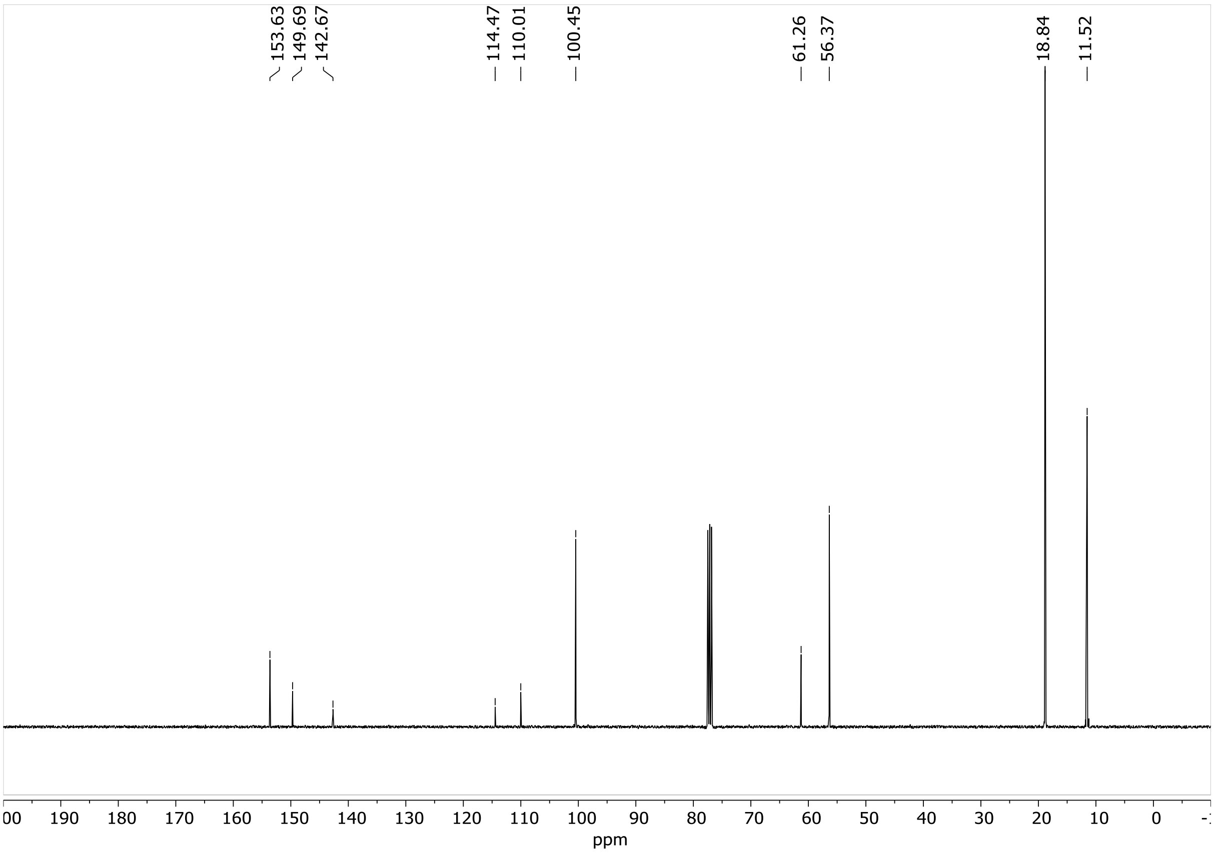

^{1}H NMR and ^{13}C NMR spectra of compound **2n**

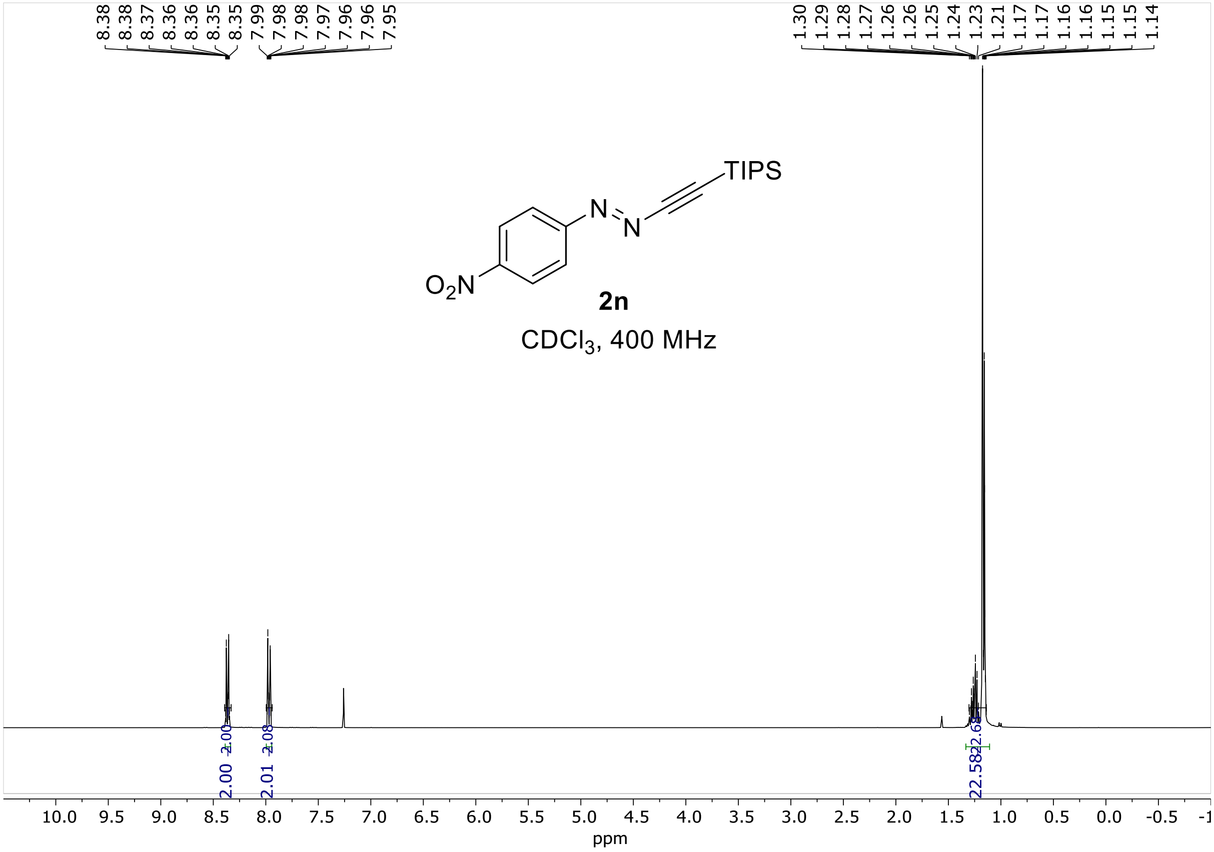

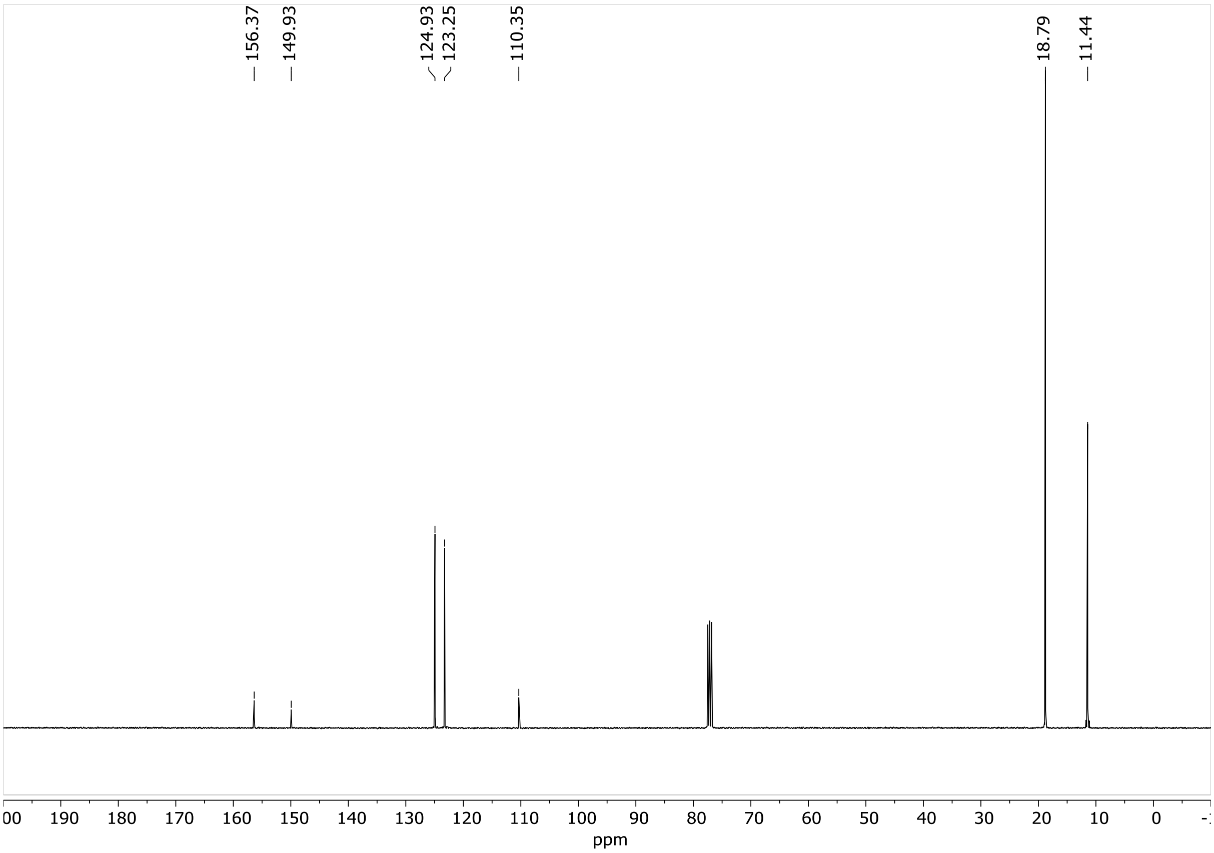

^{1}H NMR and ^{13}C NMR spectra of compound **2o**

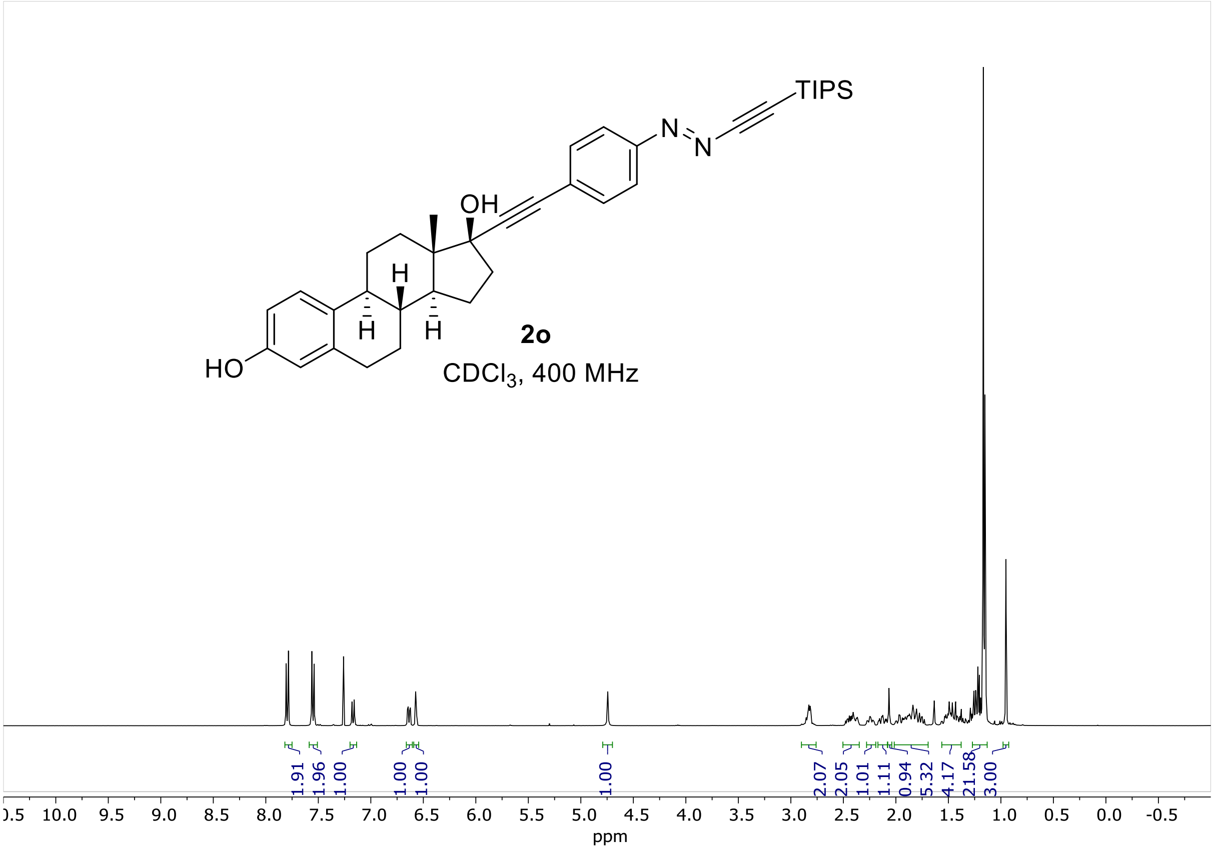

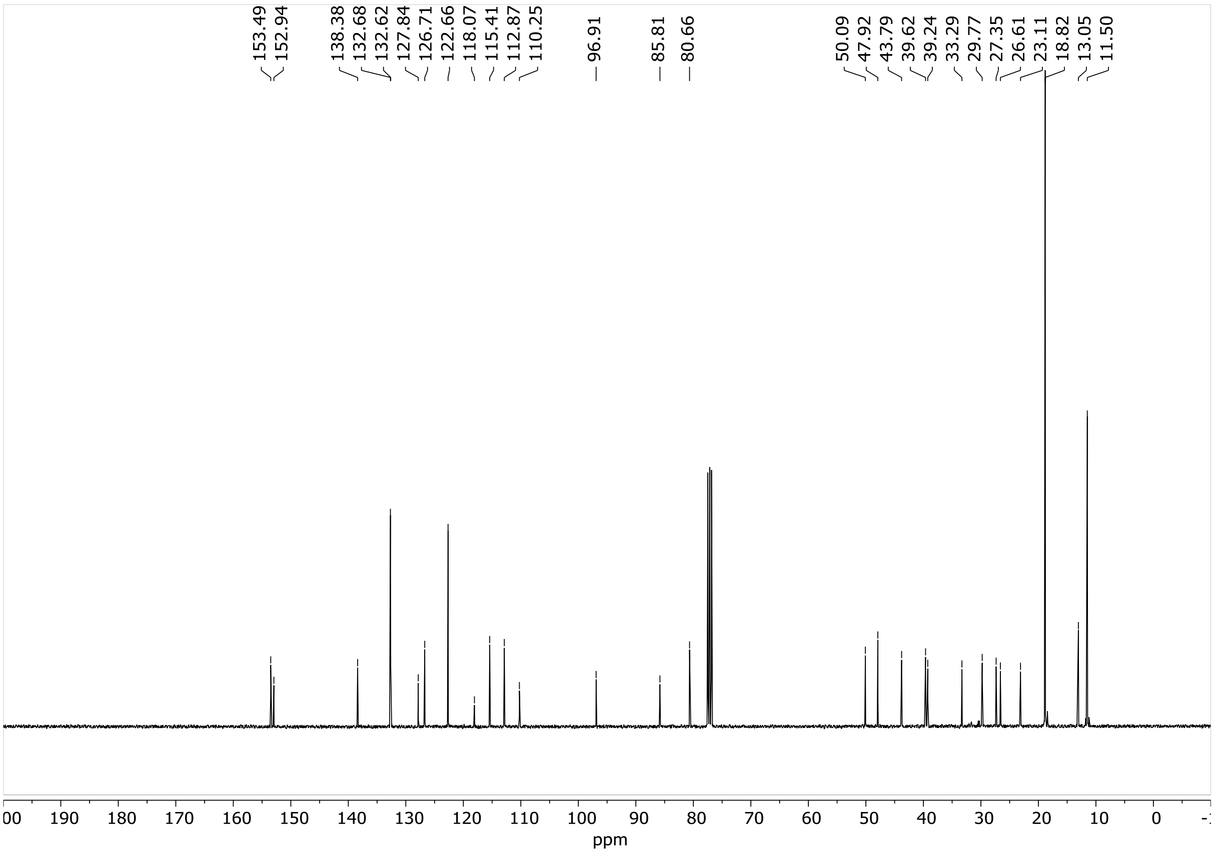

^{1}H NMR and ^{13}C NMR spectra of compound **2p**

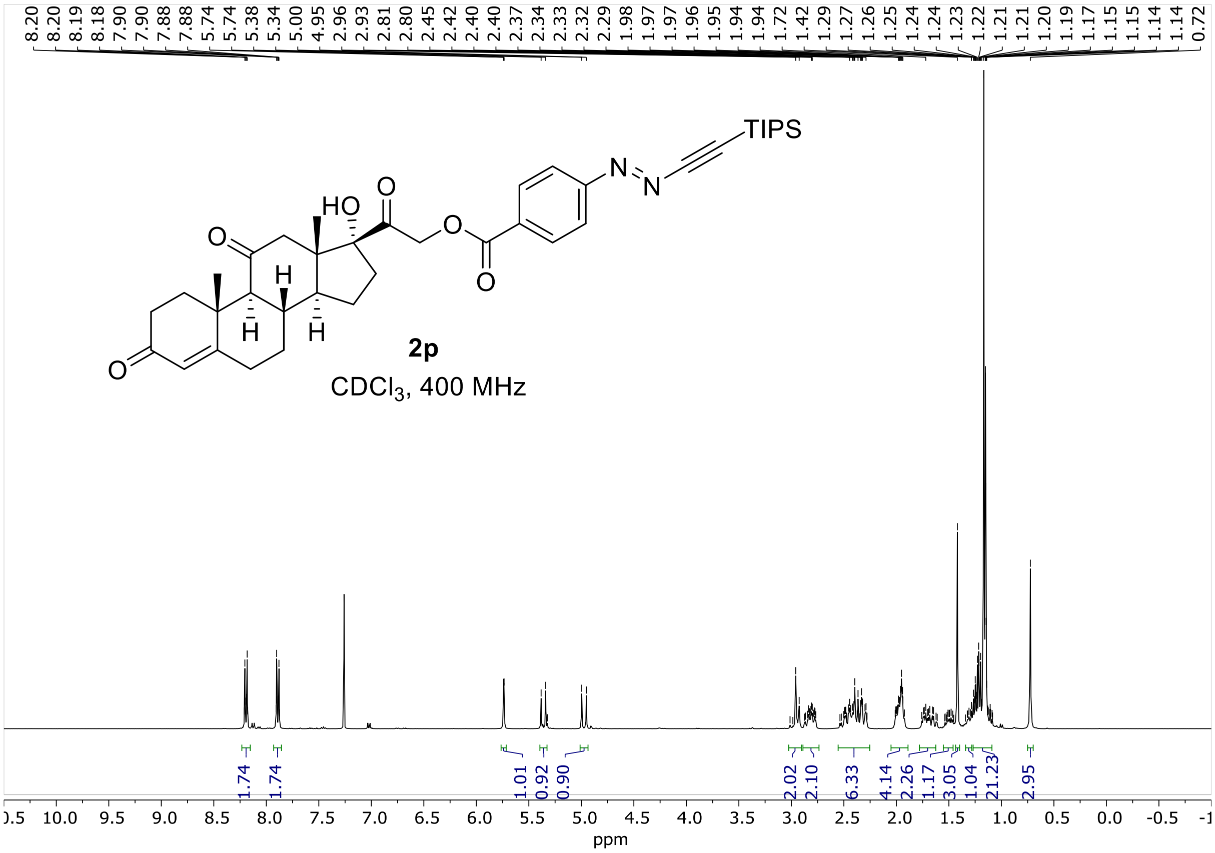

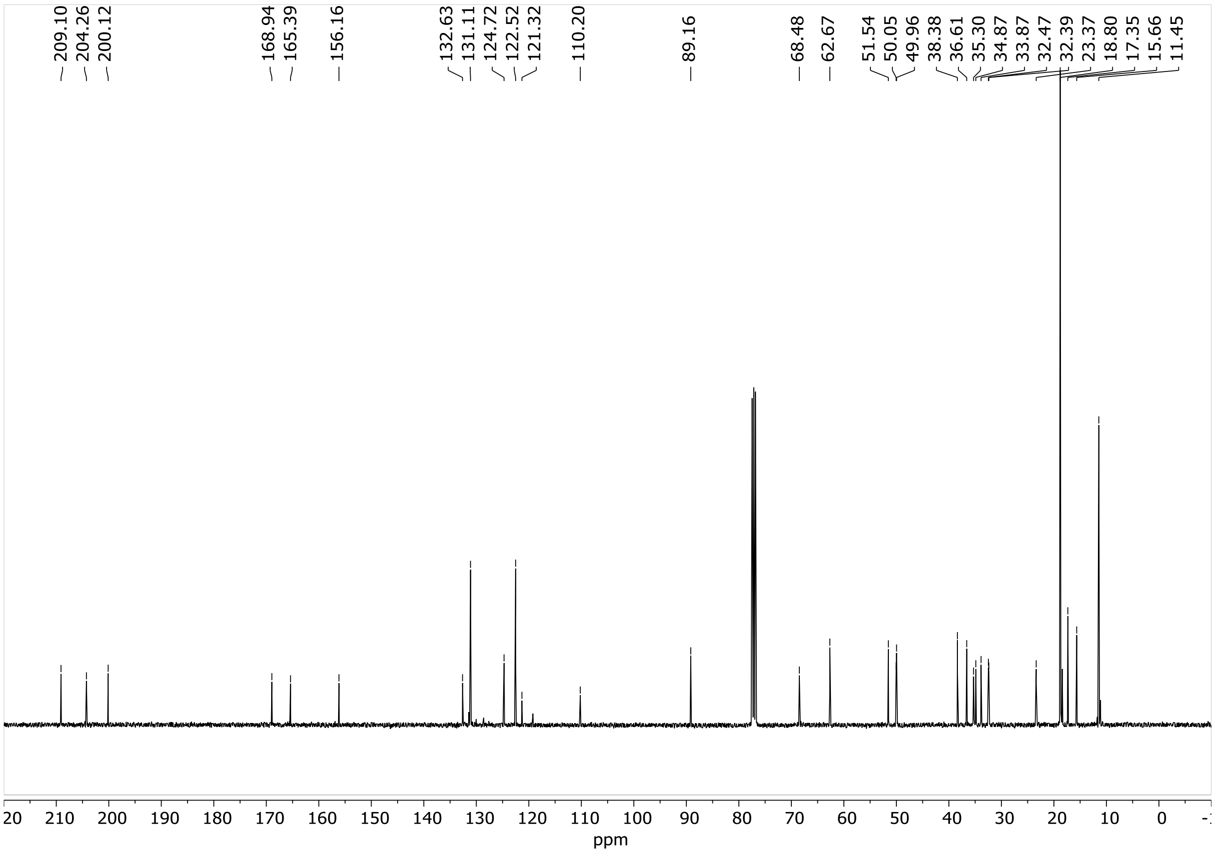

^{1}H NMR and ^{13}C NMR spectrum of compound **4a**

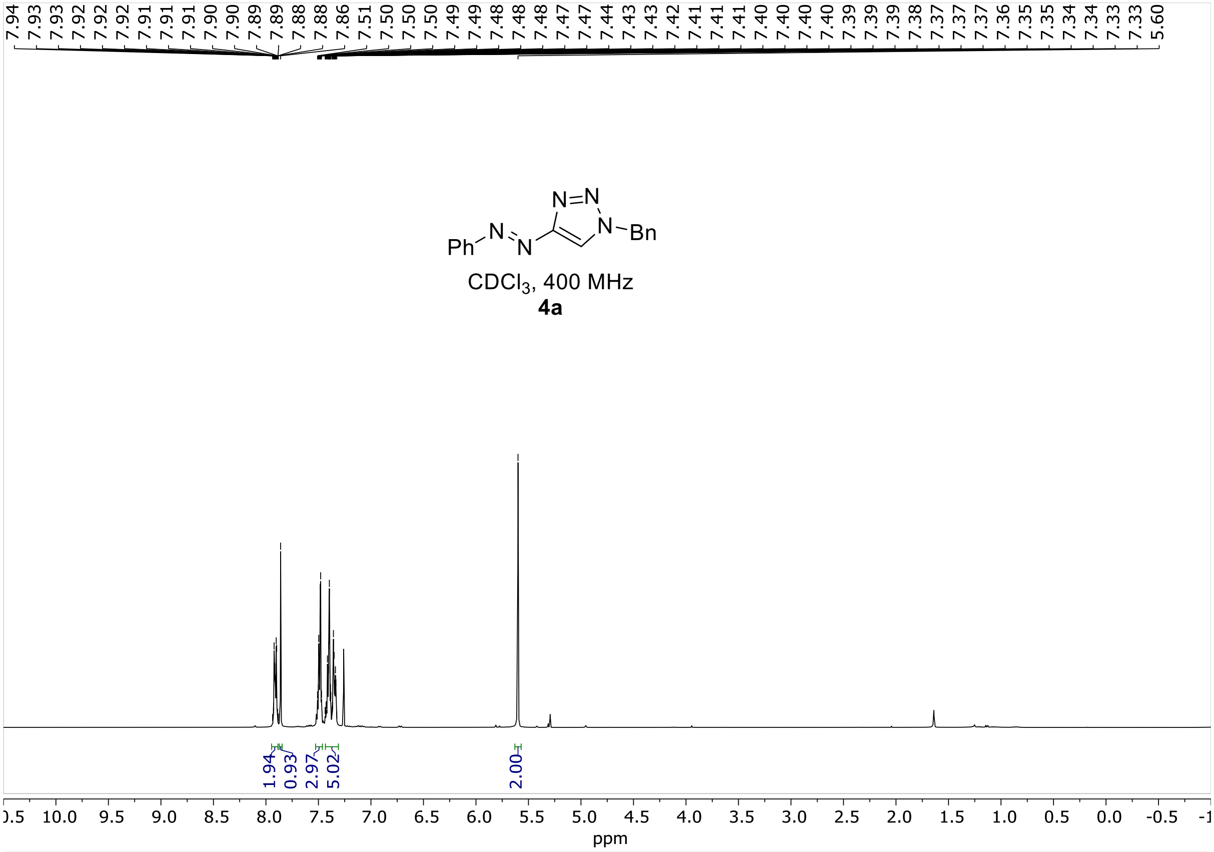

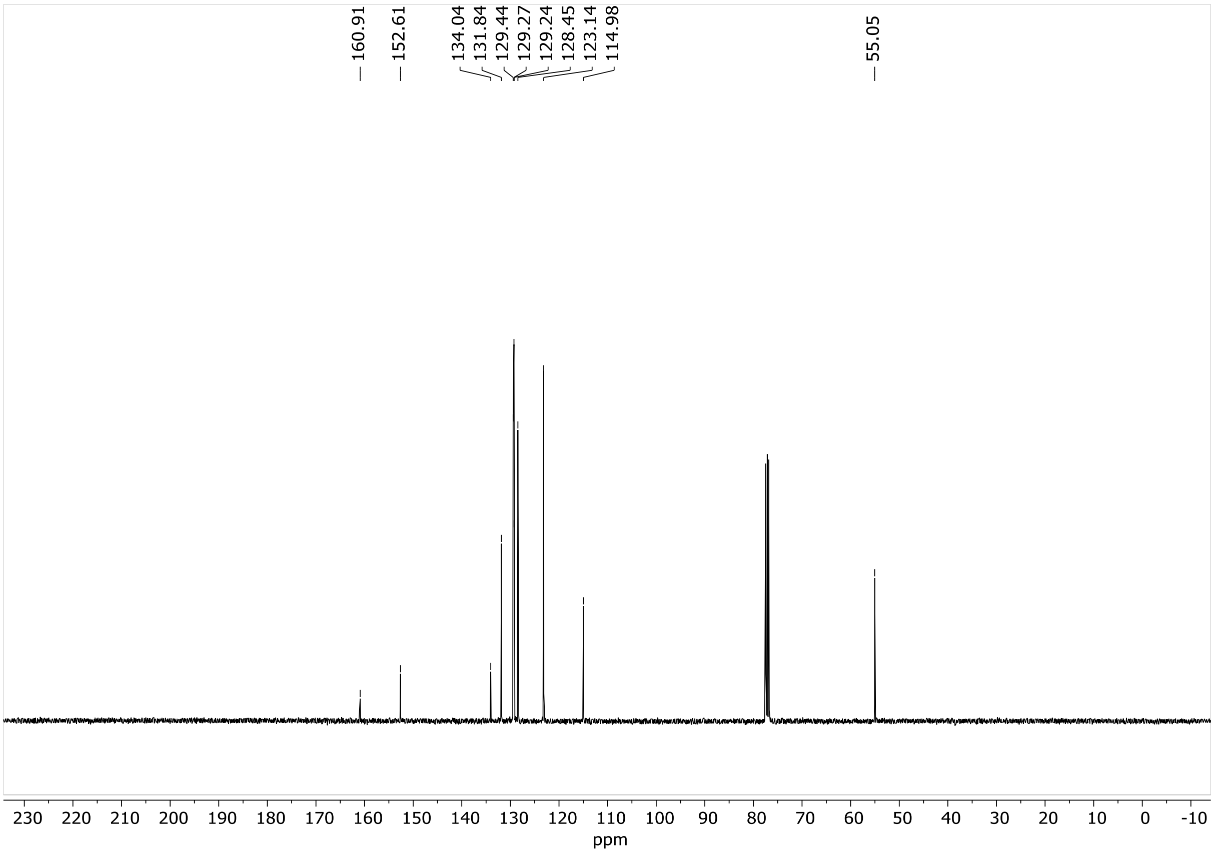

^{1}H NMR and ^{13}C NMR spectrum of compound **4b**

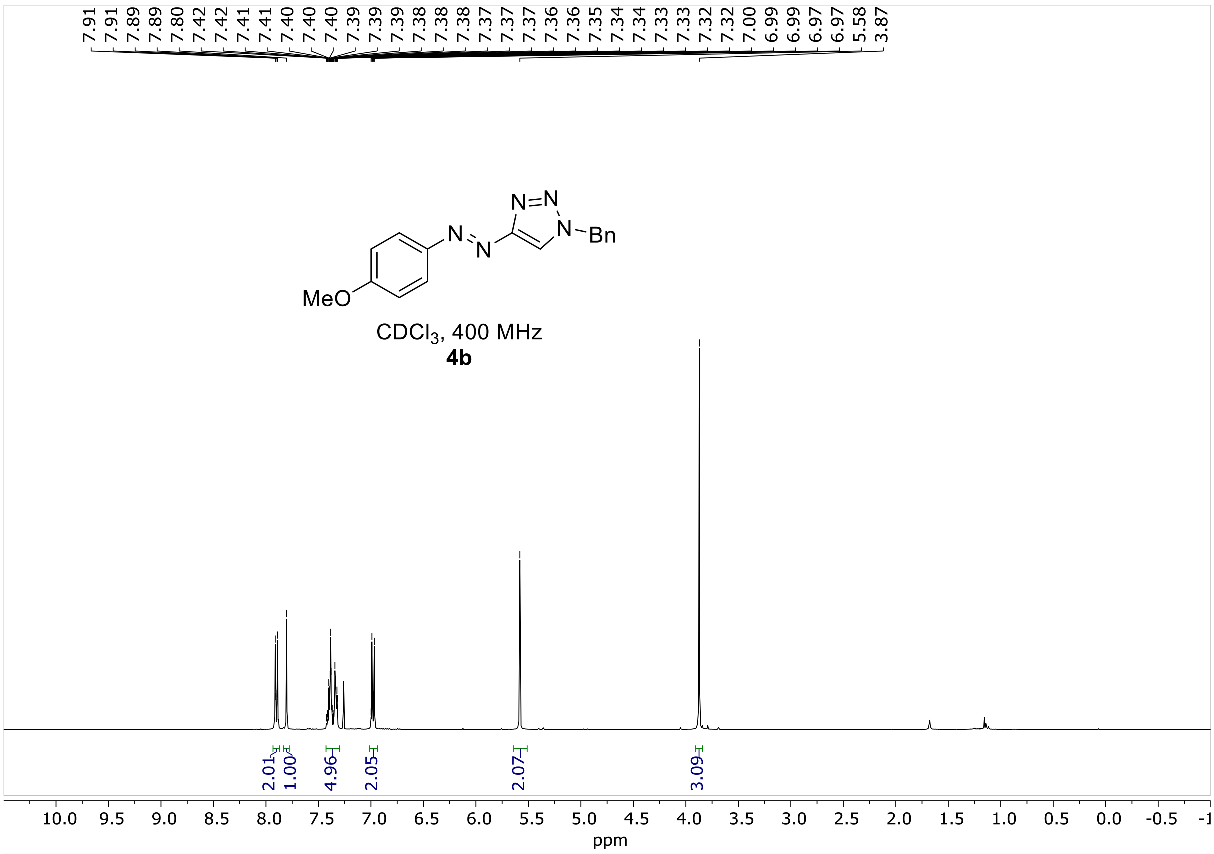
7.91 7.91 7.89 7.89 7.80 7.42 7.42 7.41 7.41 7.40 7.40 7.40 7.39 7.39 7.38 7.38 7.38 7.37 7.37 7.37 7.36 7.35 7.34 7.34 7.33 7.33 7.32 7.32 7.00 6.99 6.99 6.97 6.97 5.58 3.87
N=N
N
N
N
Bn
MeO
CDCl3, 400 MHz
4b
2.01
1.00
4.96
2.05
2.07
3.09
10.0 9.5 9.0 8.5 8.0 7.5 7.0 6.5 6.0 5.5 5.0 4.5 4.0 3.5 3.0 2.5 2.0 1.5 1.0 0.5 0.0 -0.5
ppm

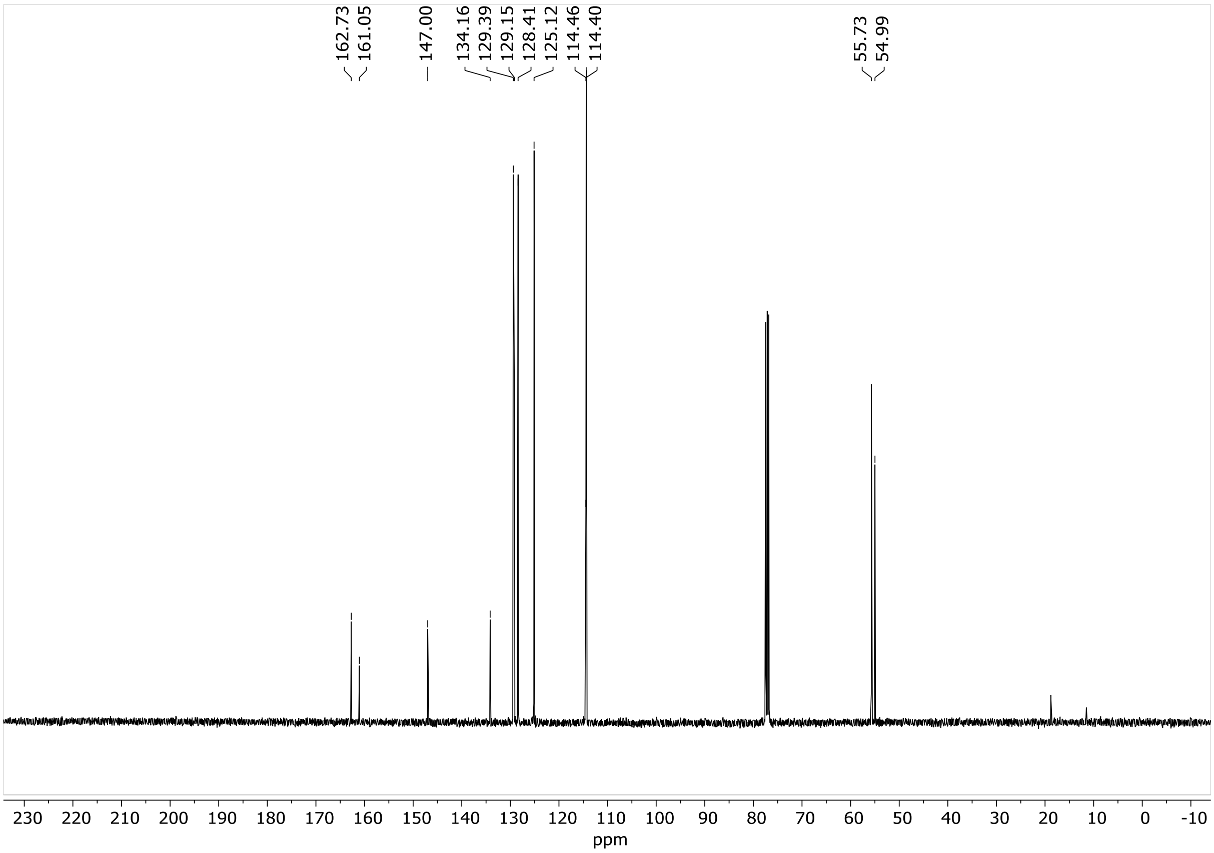
162.73
161.05
147.00
134.16
129.39
129.15
128.41
125.12
114.46
114.40
55.73
54.99
230 220 210 200 190 180 170 160 150 140 130 120 110 100 90 80 70 60 50 40 30 20 10 0 -10
ppm

^{1}H NMR and ^{13}C NMR spectrum of compound **4c**

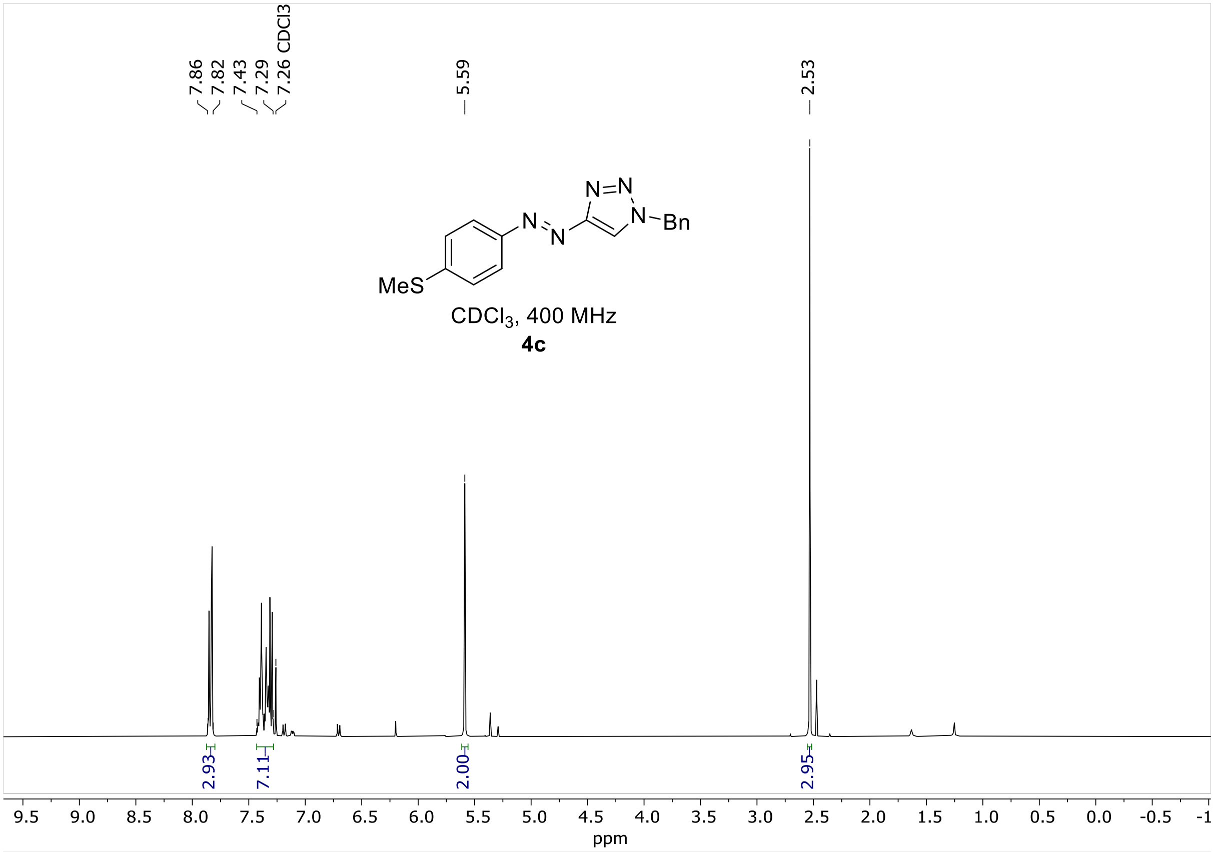

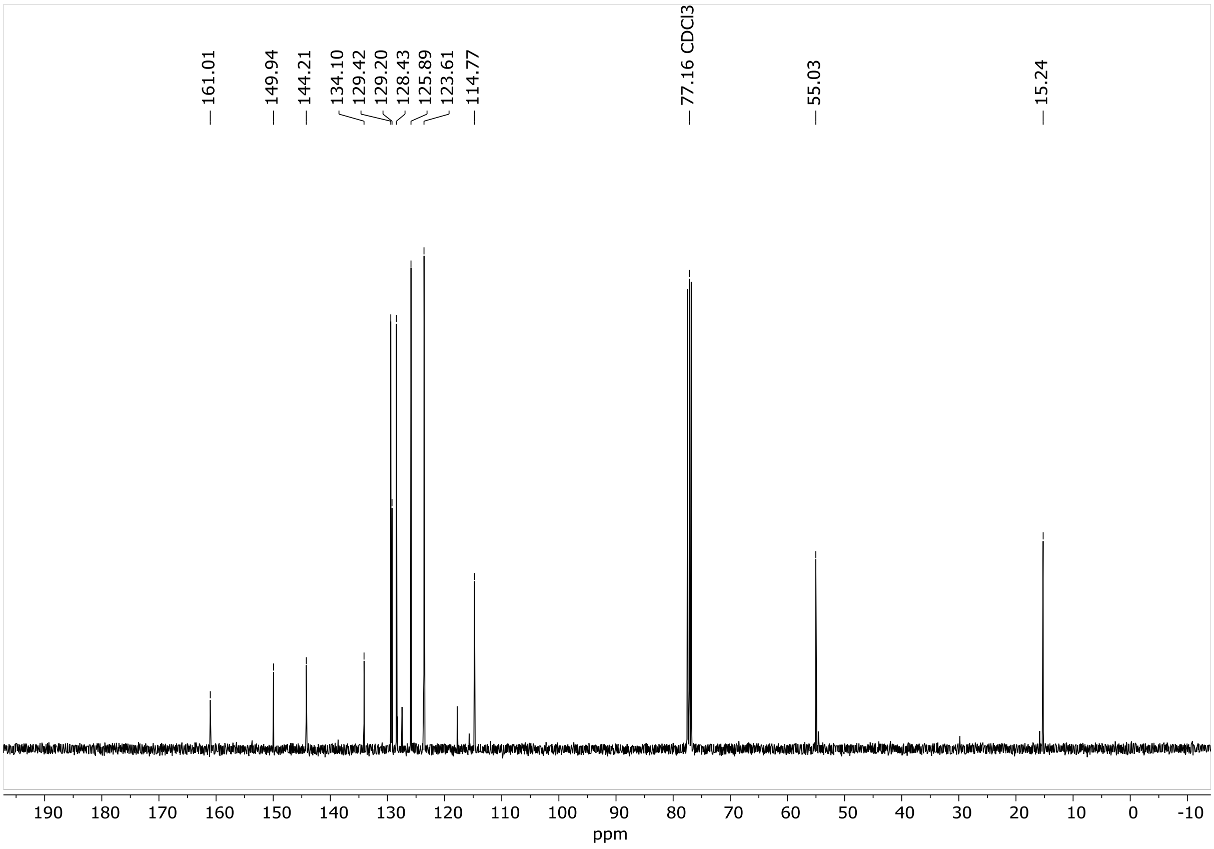

^{1}H NMR and ^{13}C NMR spectrum of compound **4d**

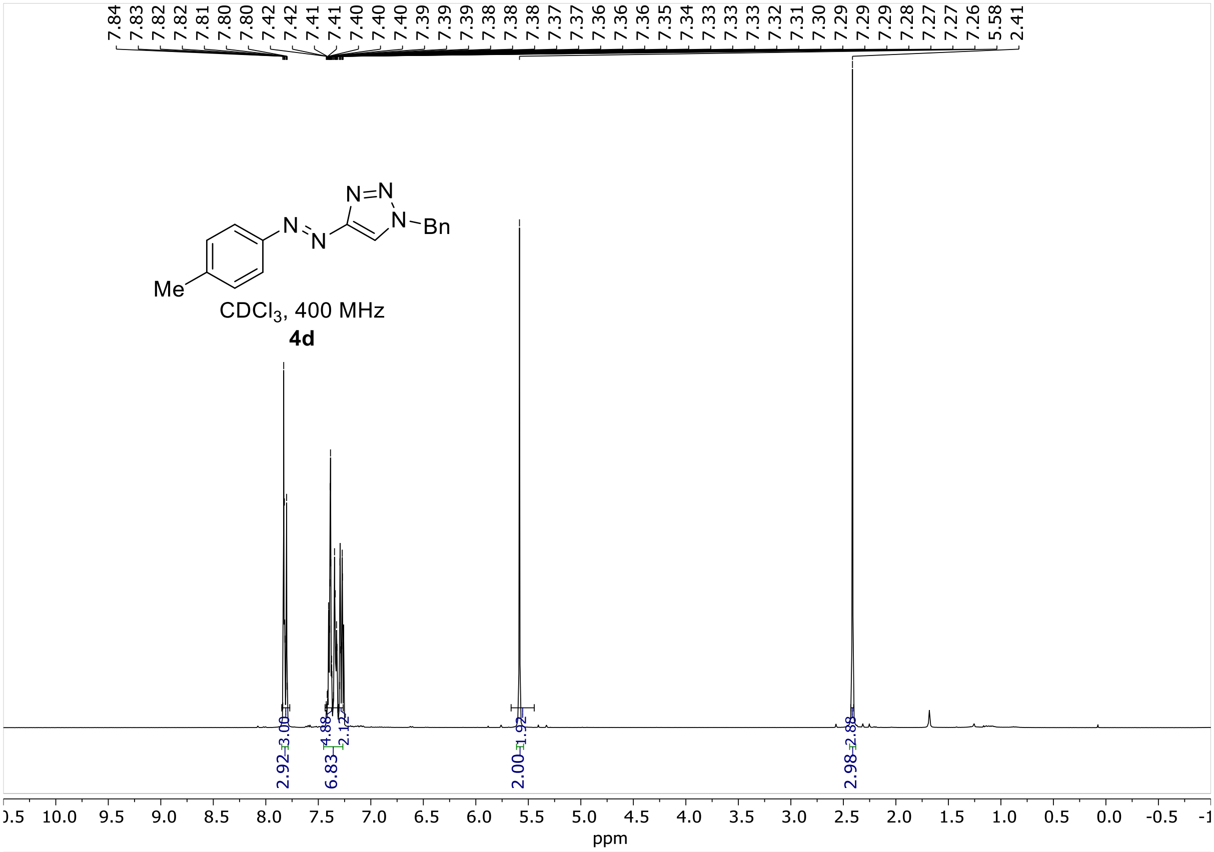

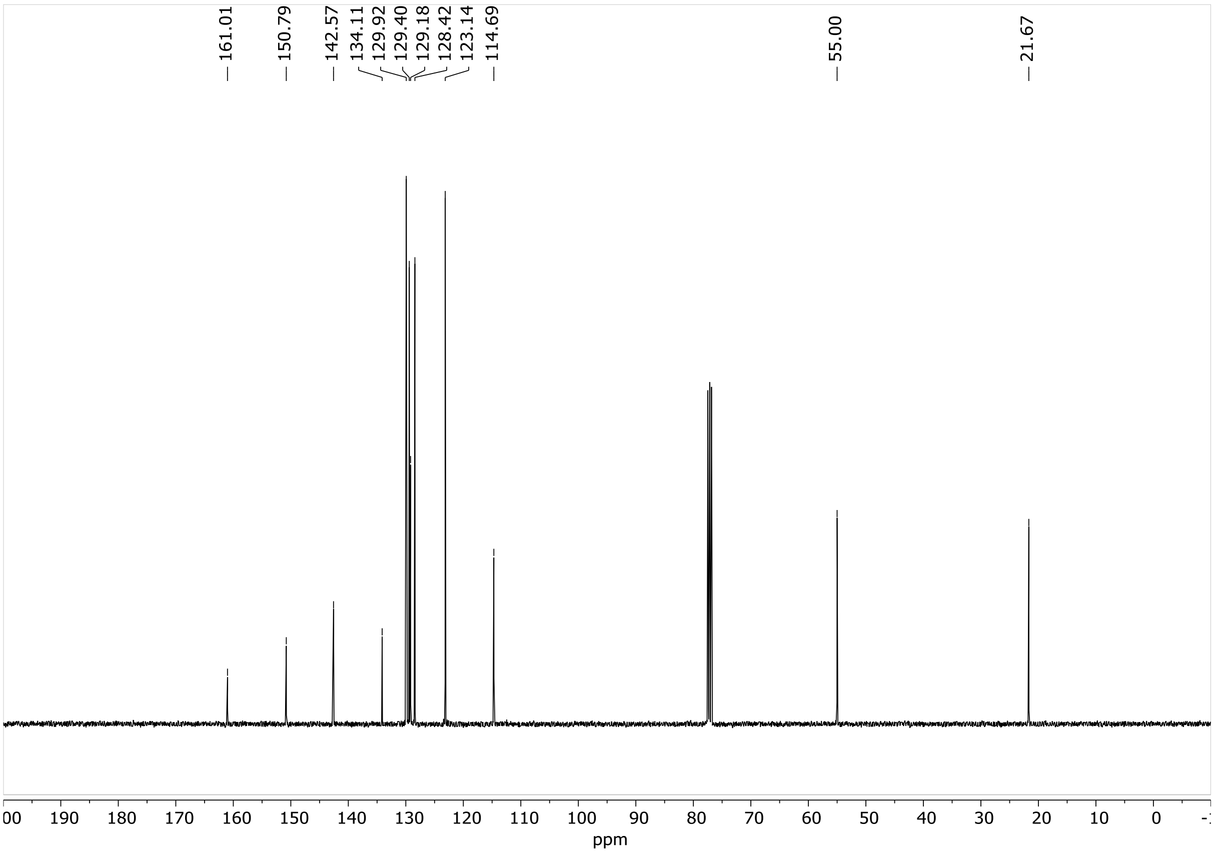

^{1}H NMR and ^{13}C NMR spectrum of compound **4e**

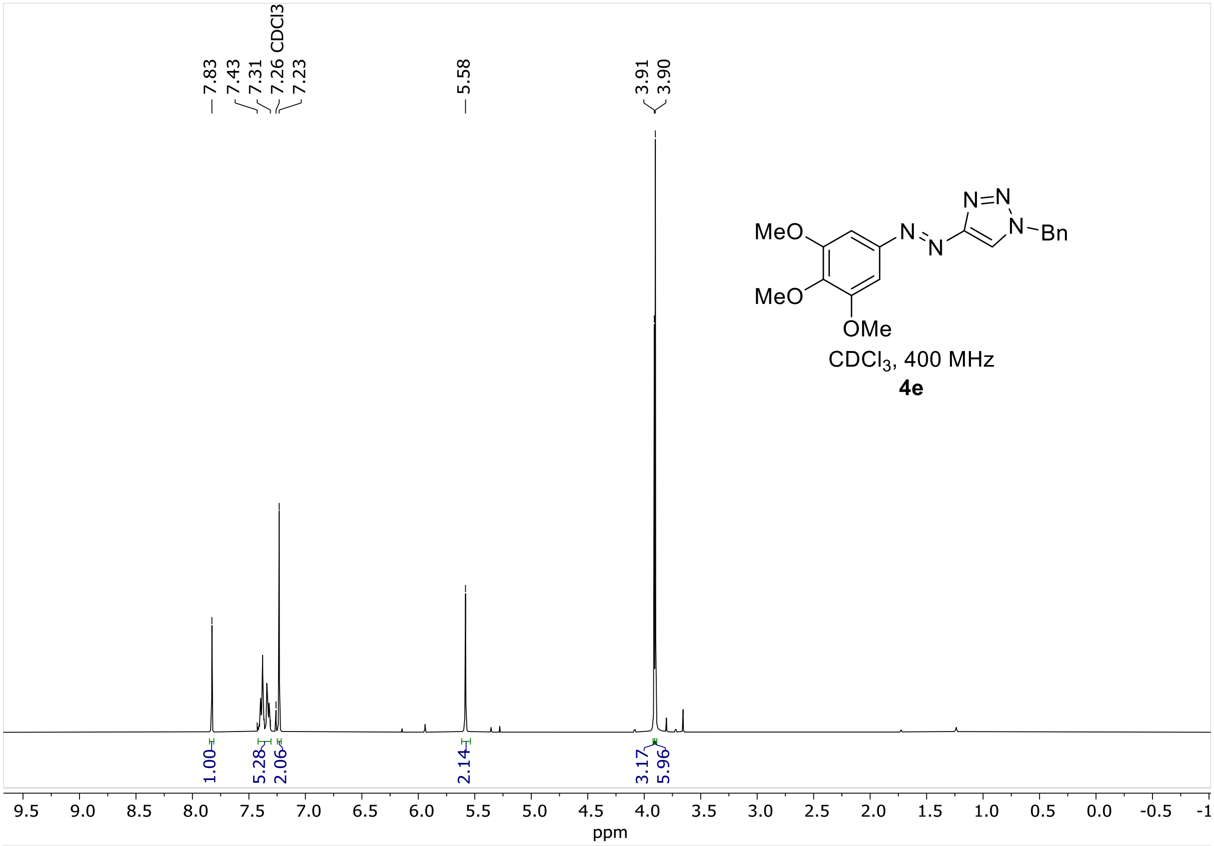

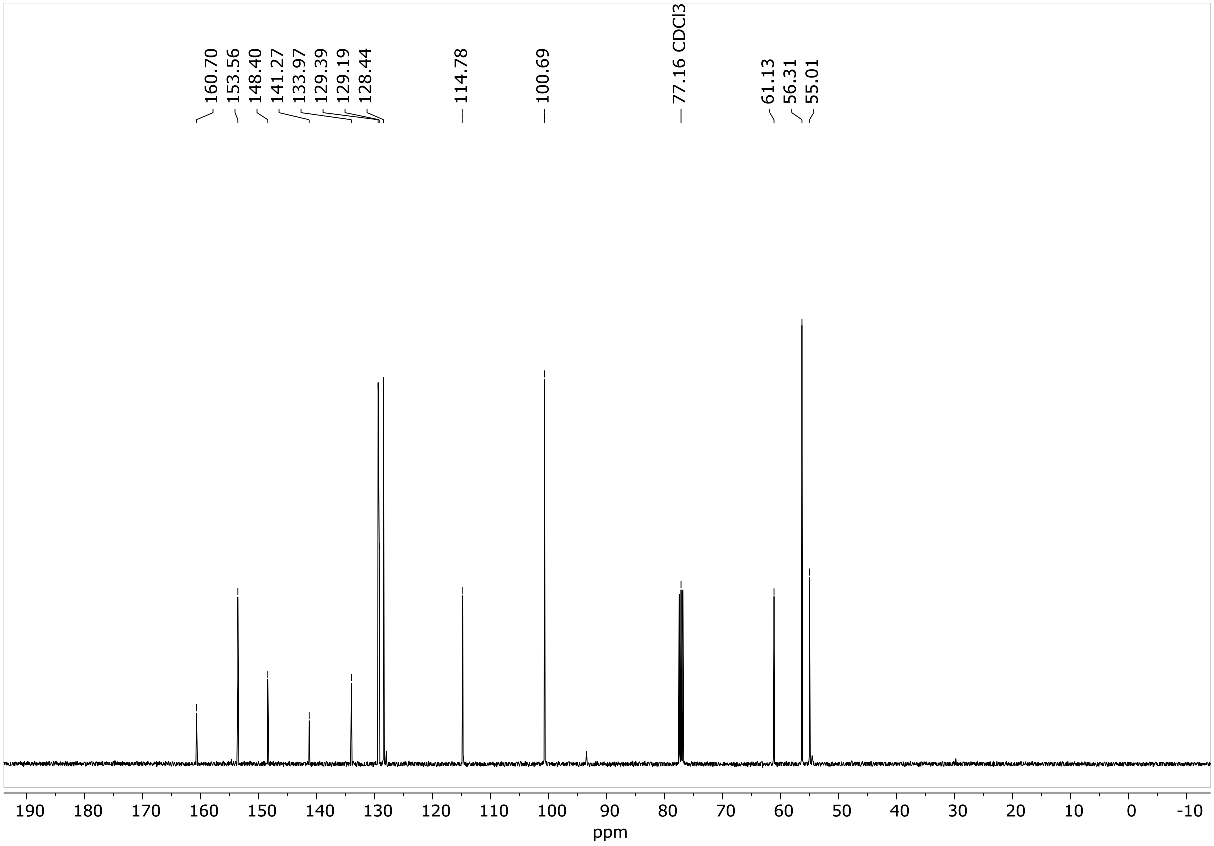

^{1}H NMR and ^{13}C NMR spectrum of compound **4f**

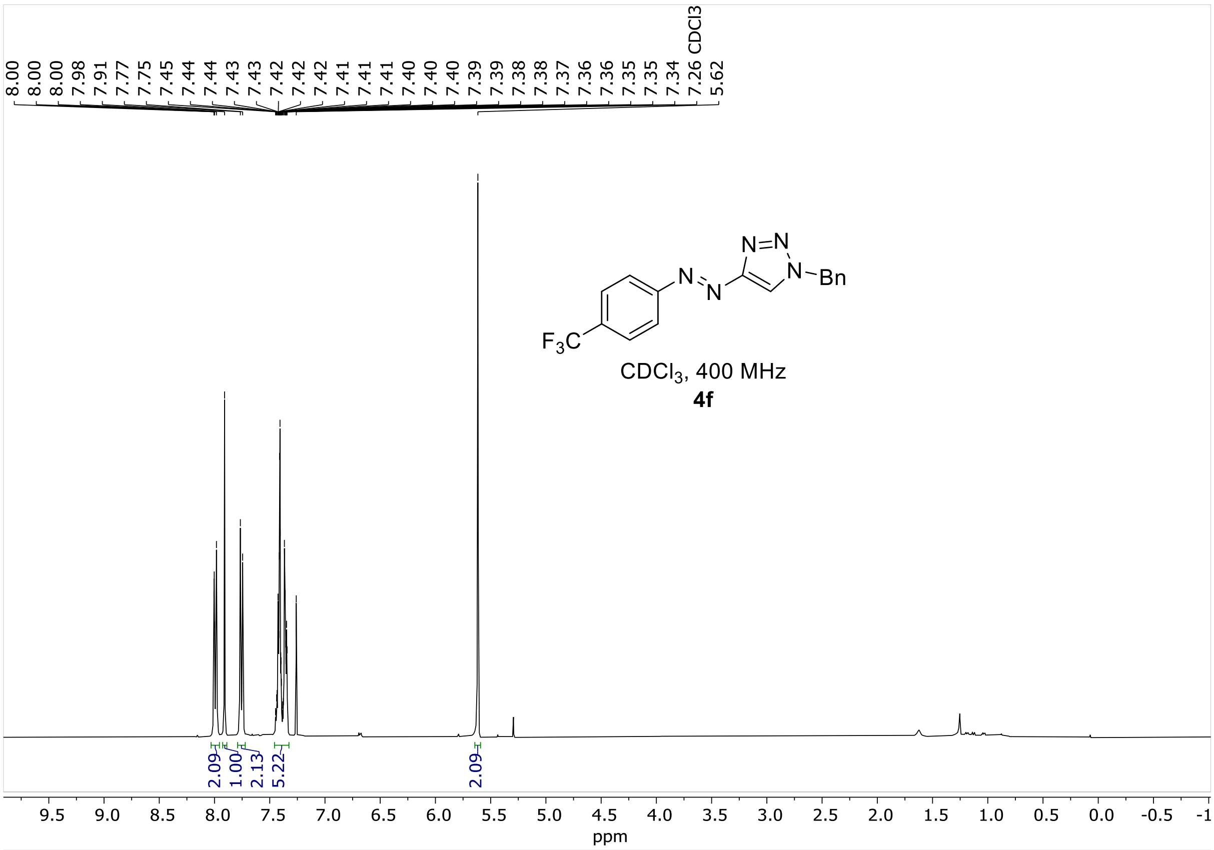

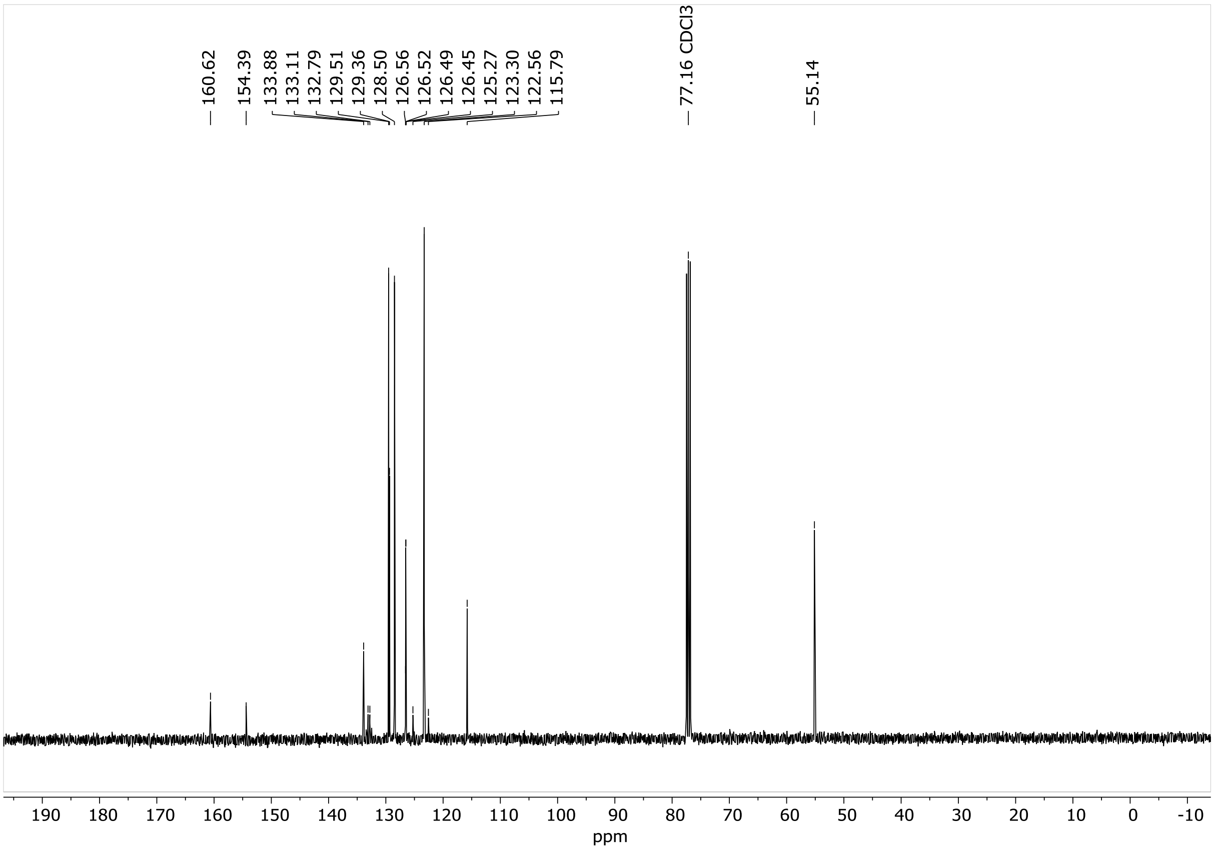

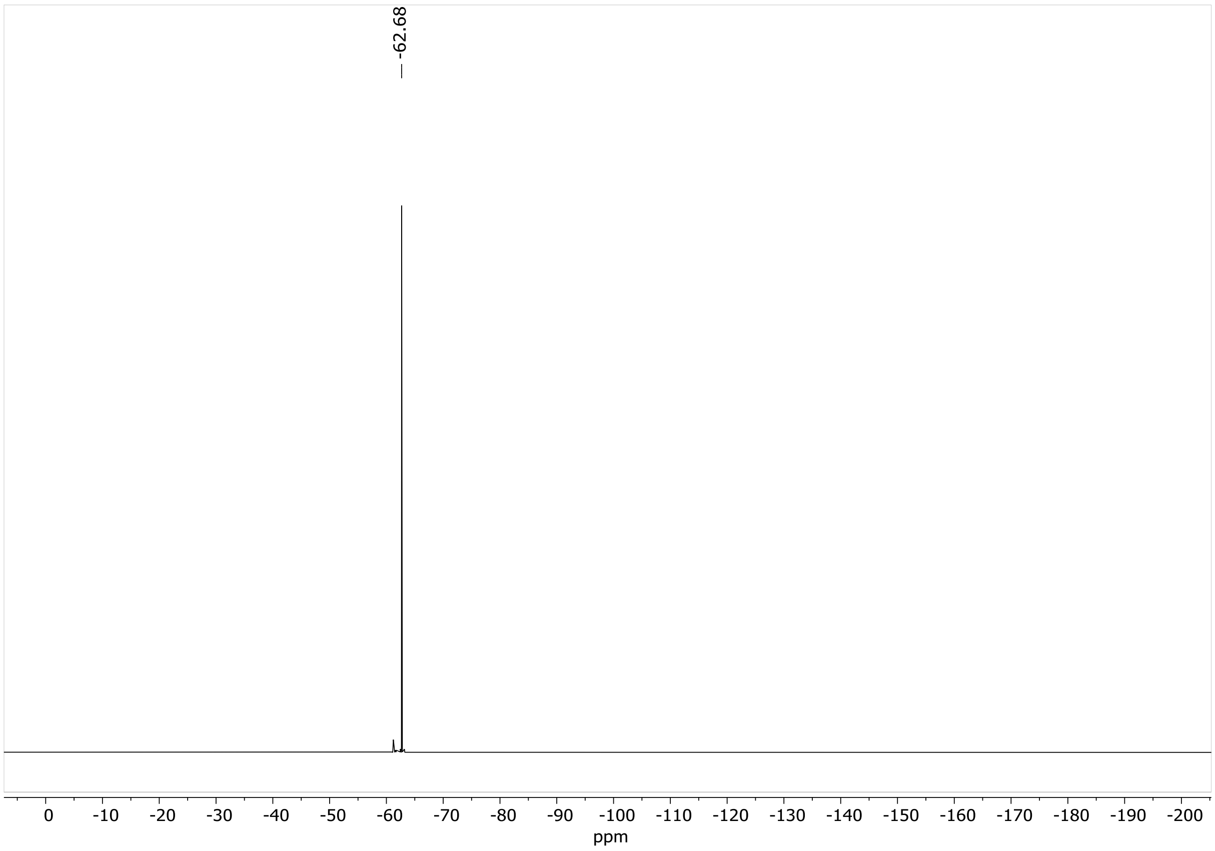
-62.68
0
-10
-20
-30
-40
-50
-60
-70
-80
-90
-100
-110
-120
-130
-140
-150
-160
-170
-180
-190
-200
ppm

^{1}H NMR and ^{13}C NMR spectrum of compound **4g**

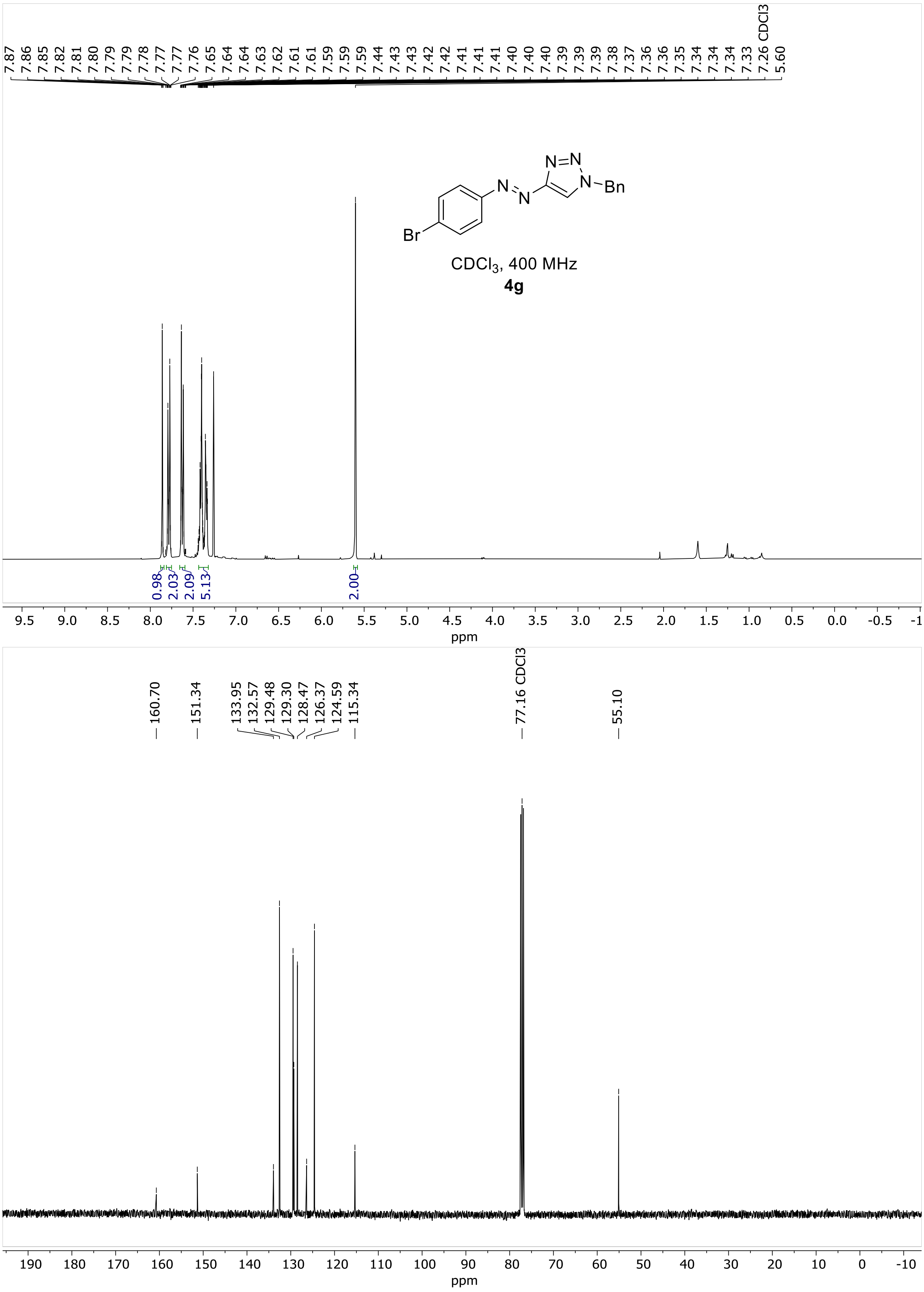

^{1}H NMR and ^{13}C NMR spectrum of compound **4h**

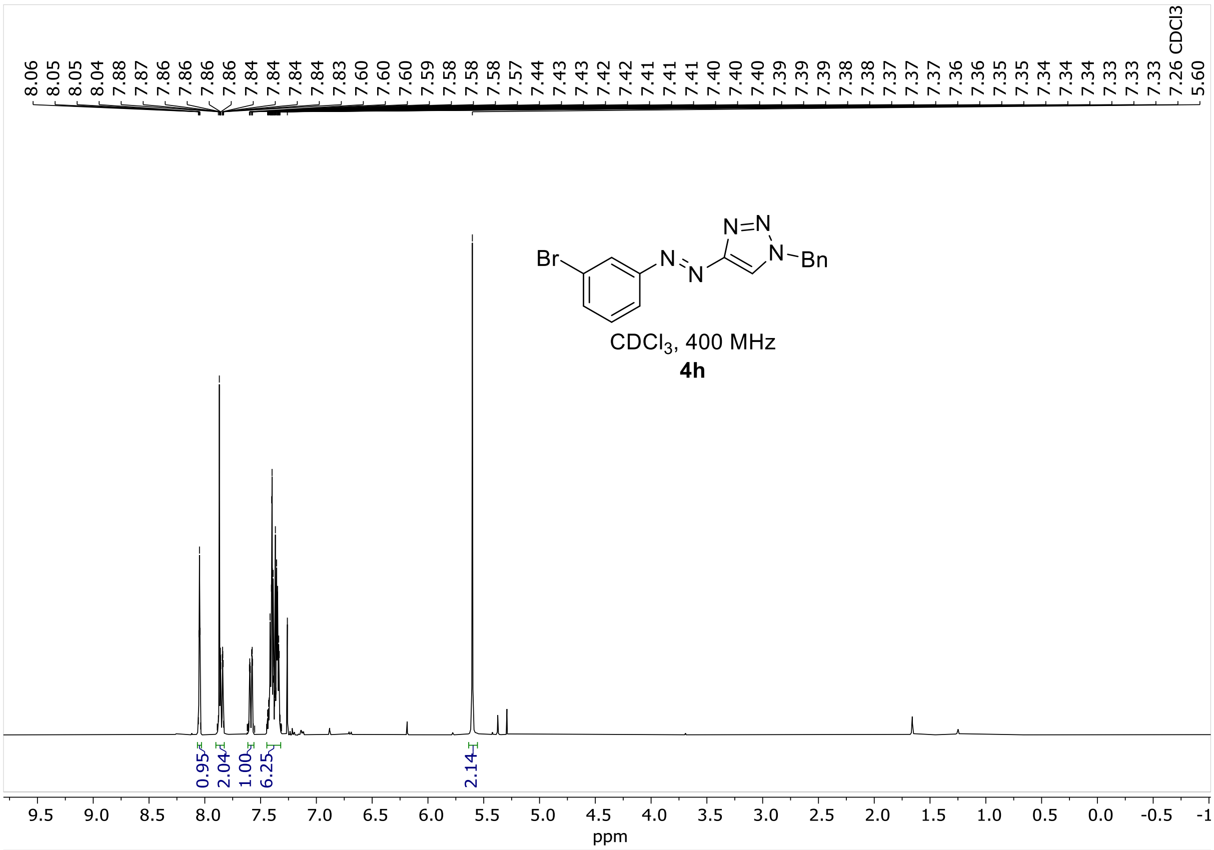

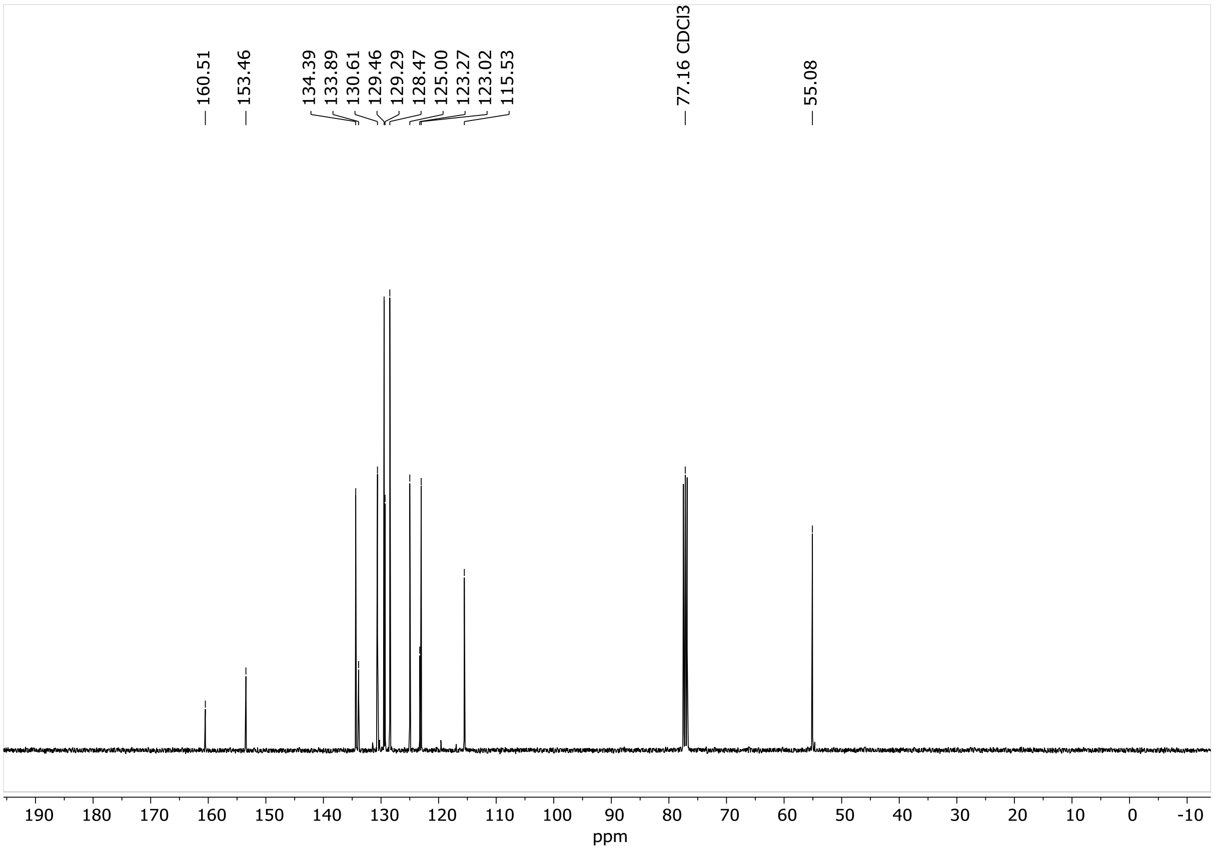

^{1}H NMR and ^{13}C NMR spectrum of compound **4i**

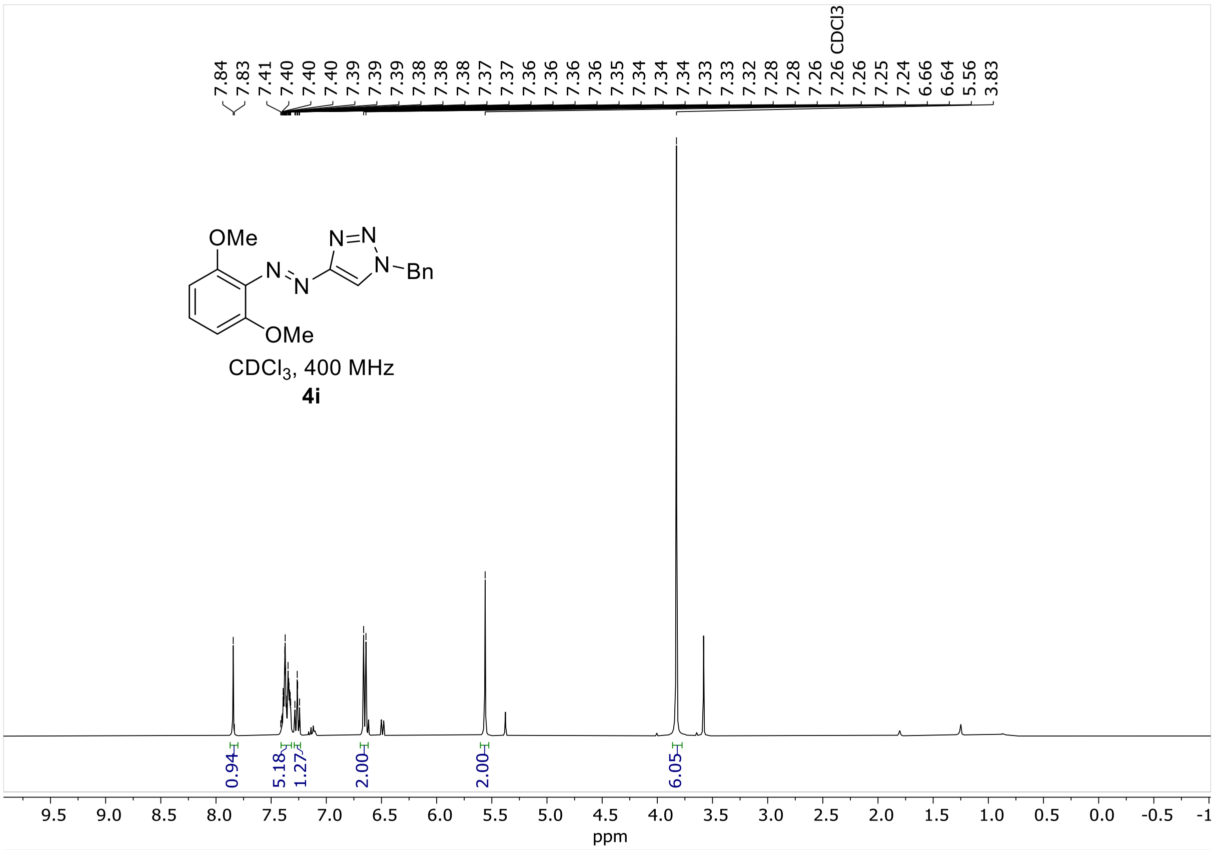

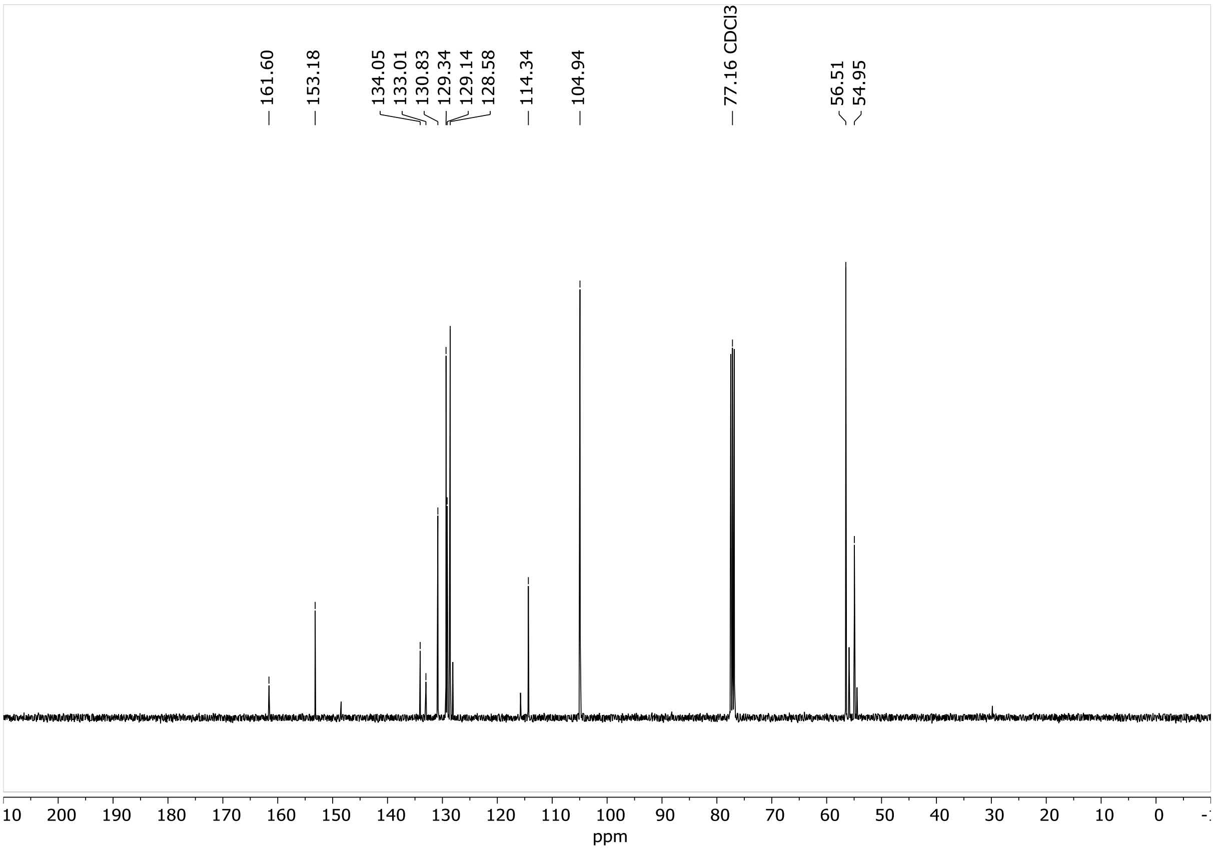

^{1}H NMR and ^{13}C NMR spectrum of compound **4j**

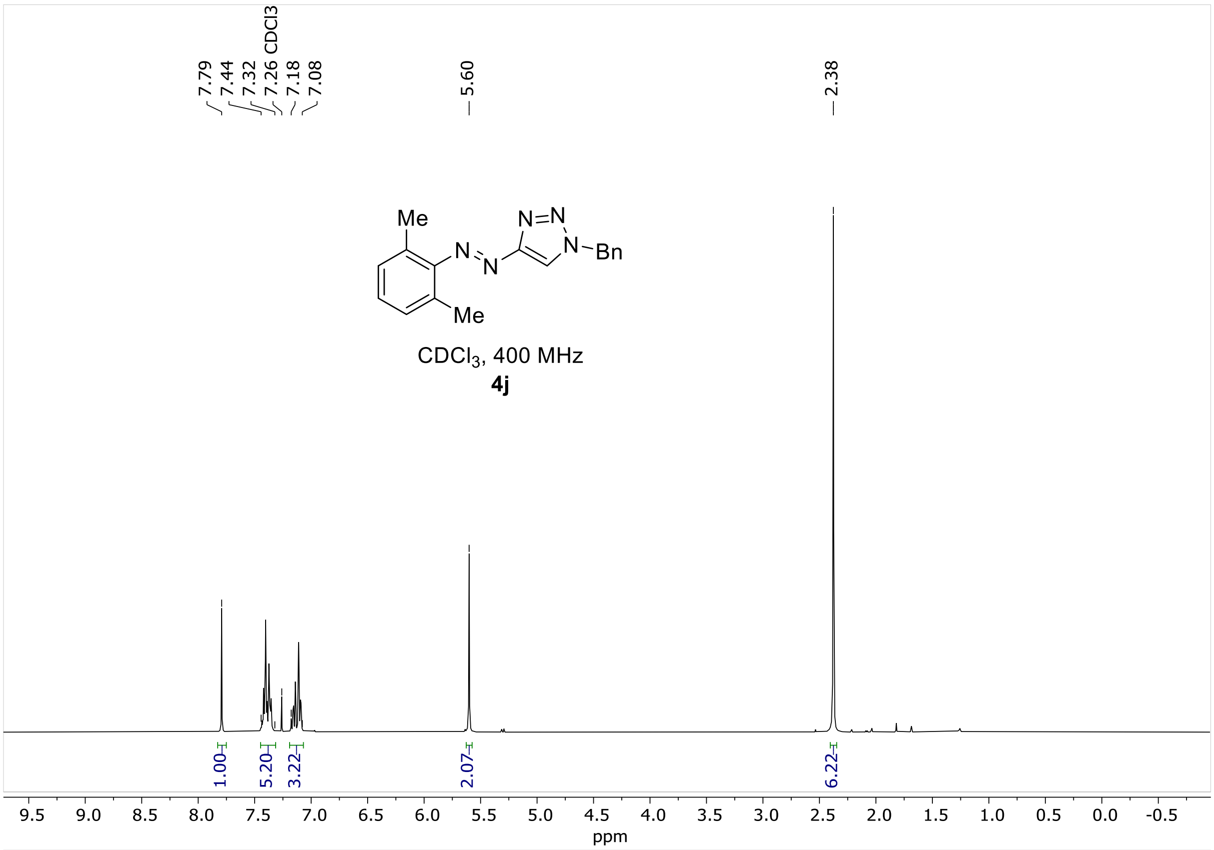

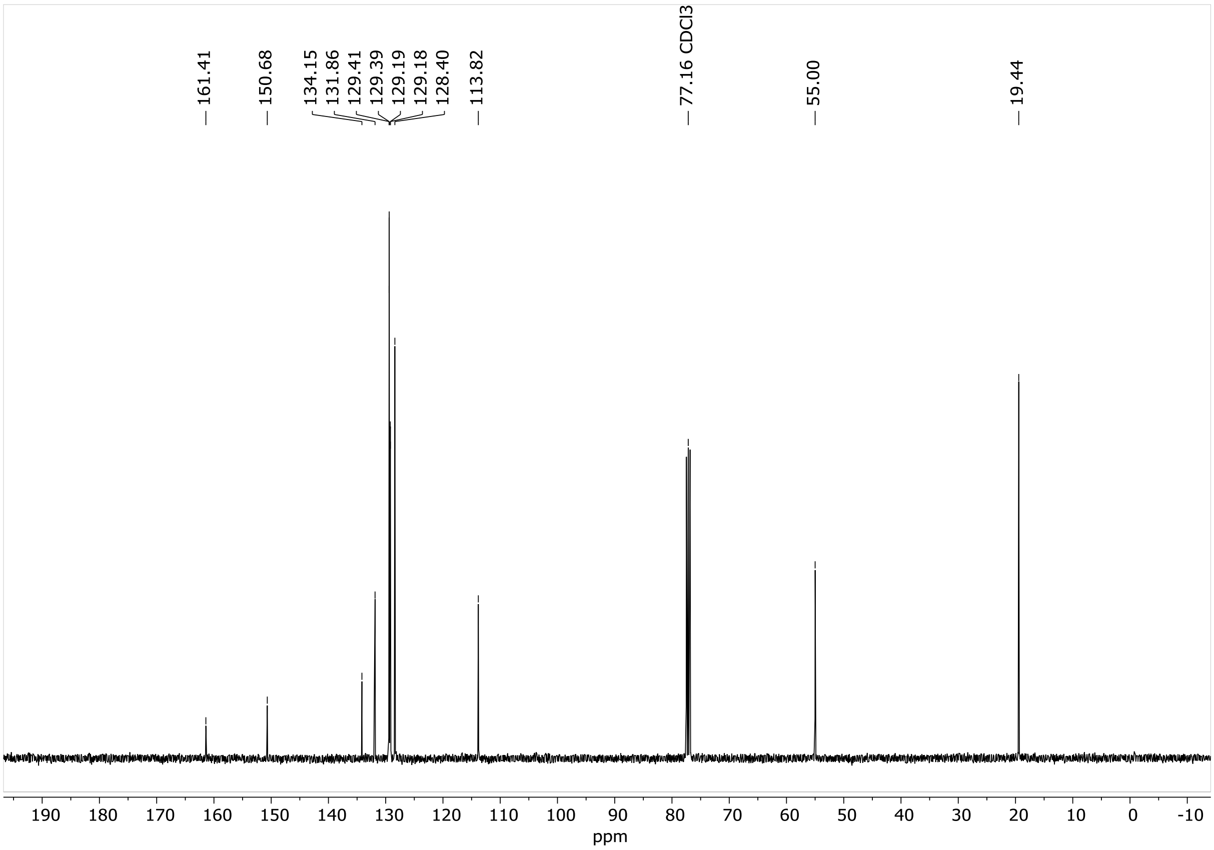

^{1}H NMR ^{13}C NMR and ^{19}F spectrum of compound **4k**

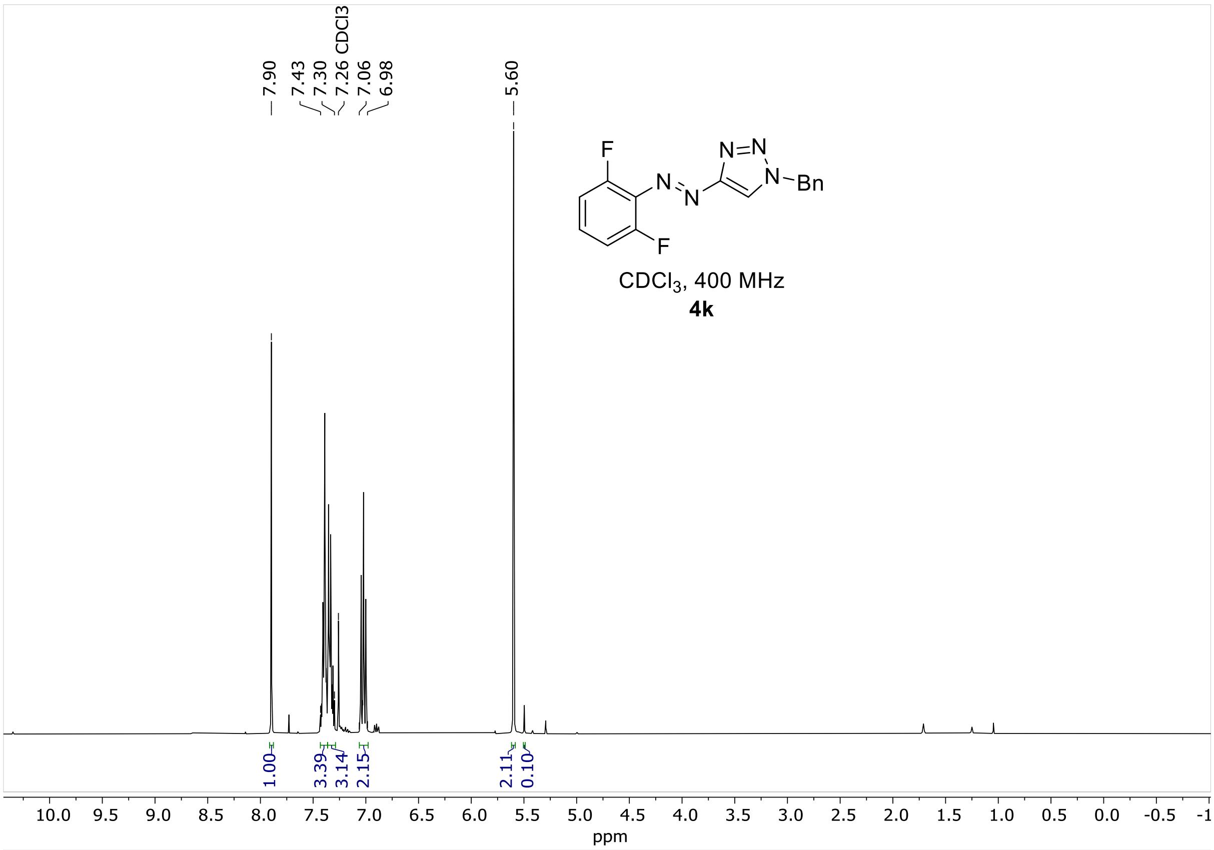

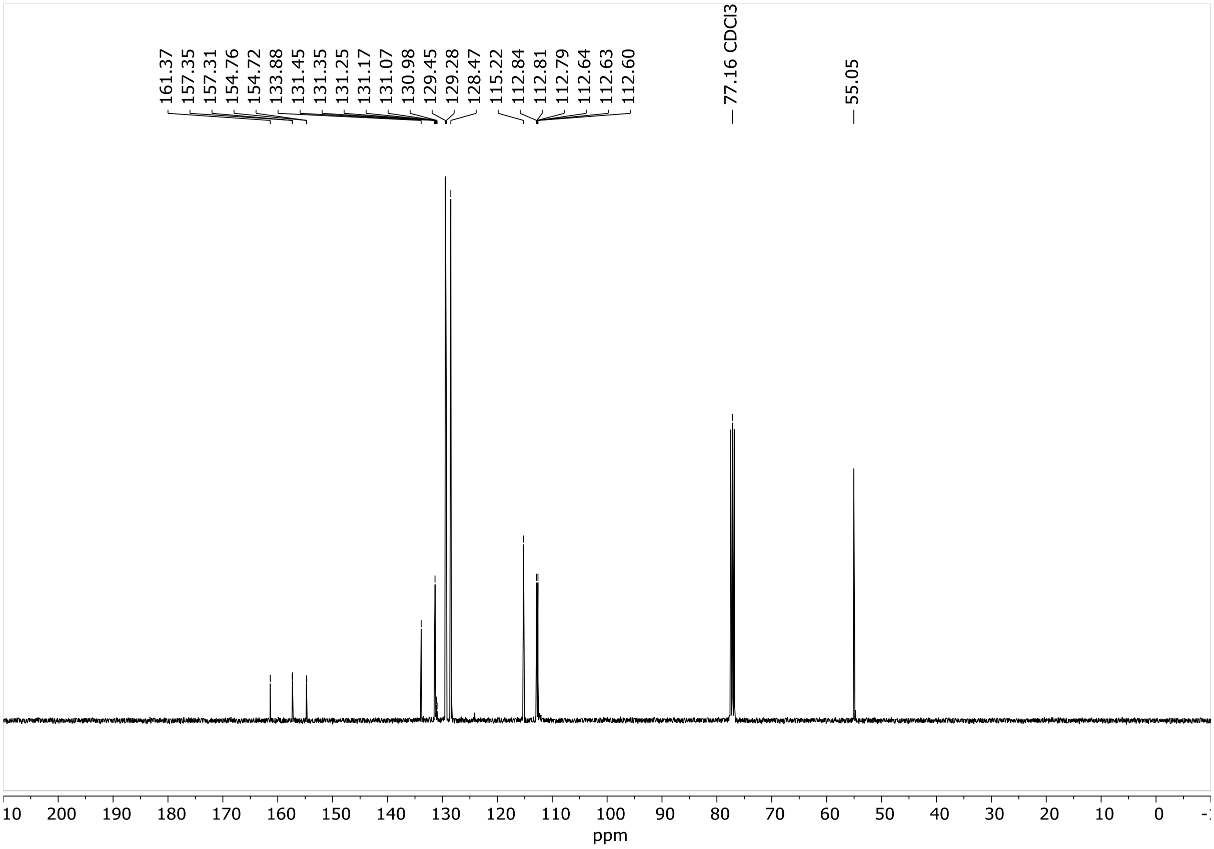

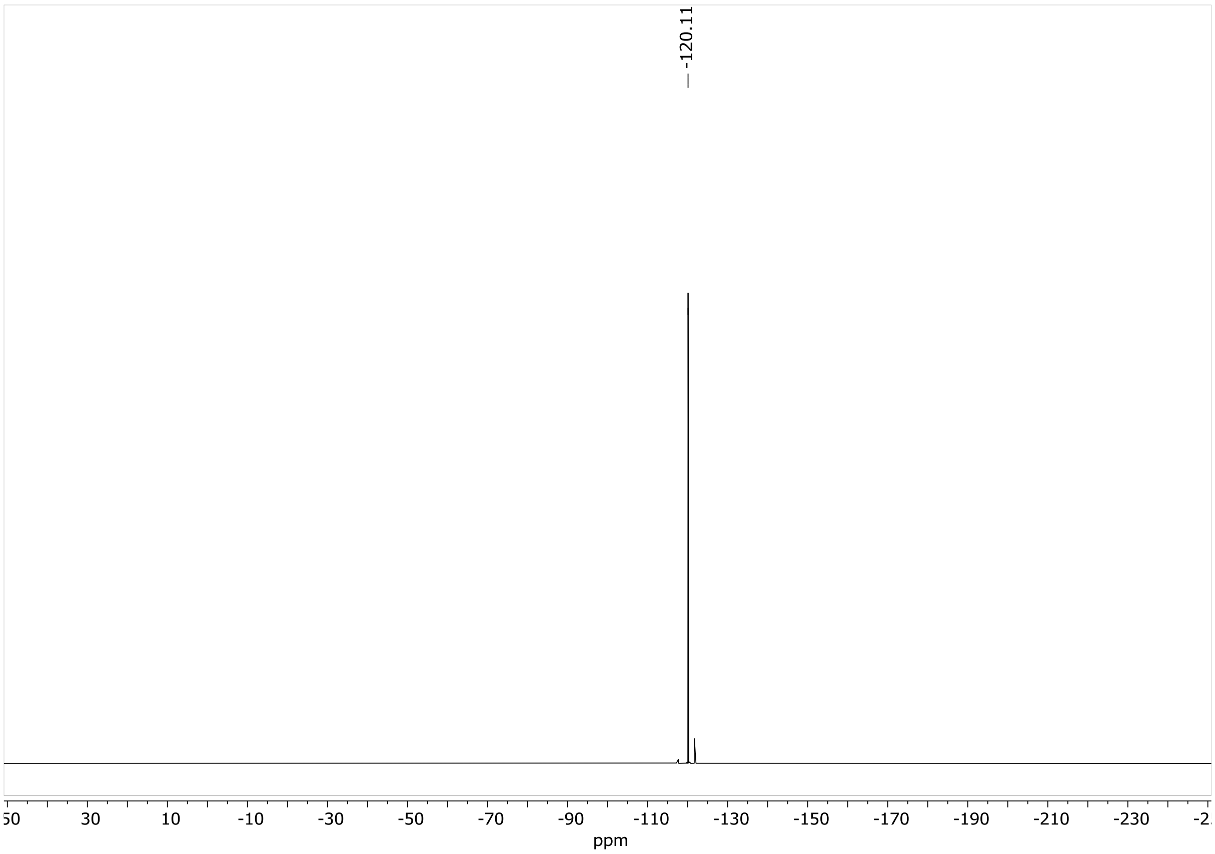
-120.11
50
30
10
-10
-30
-50
-70
-90
-110
-130
-150
-170
-190
-210
-230
ppm

^{1}H NMR and ^{13}C NMR spectrum of compound **4l**

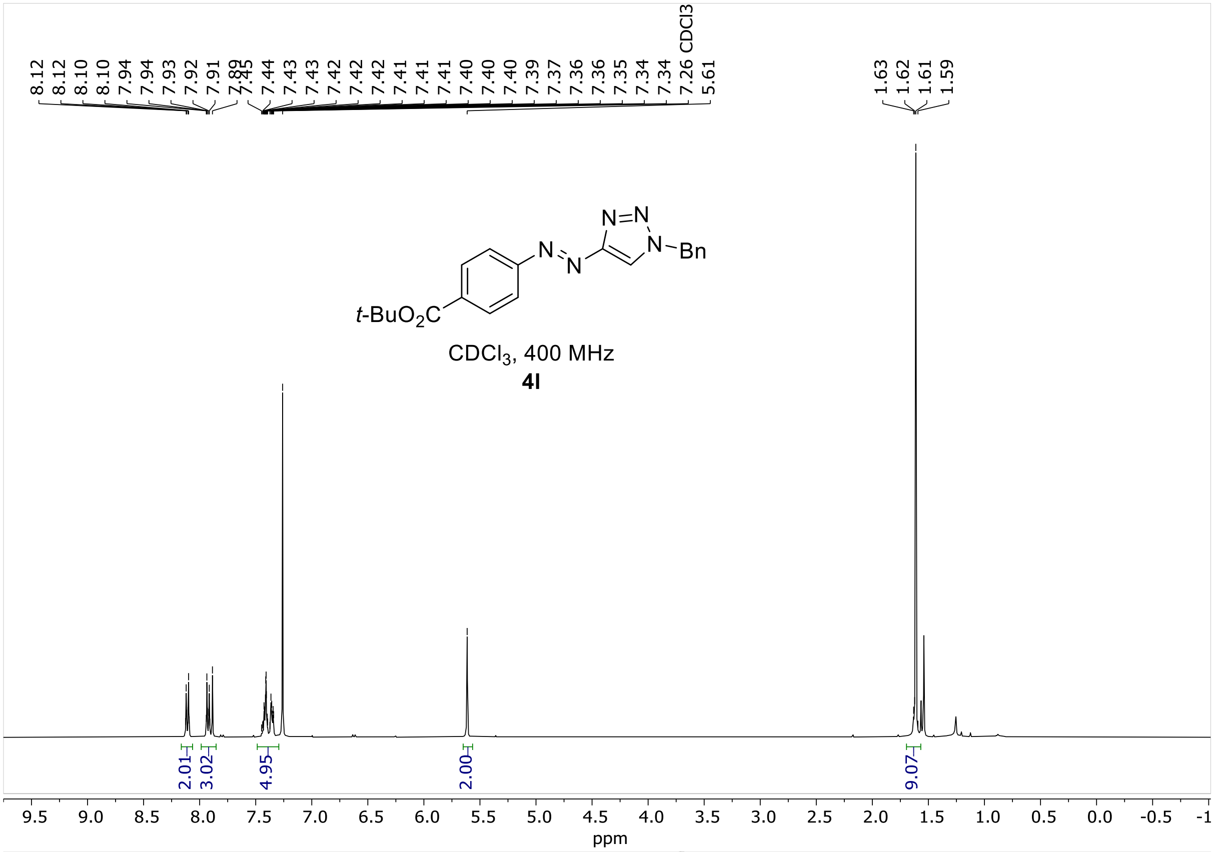

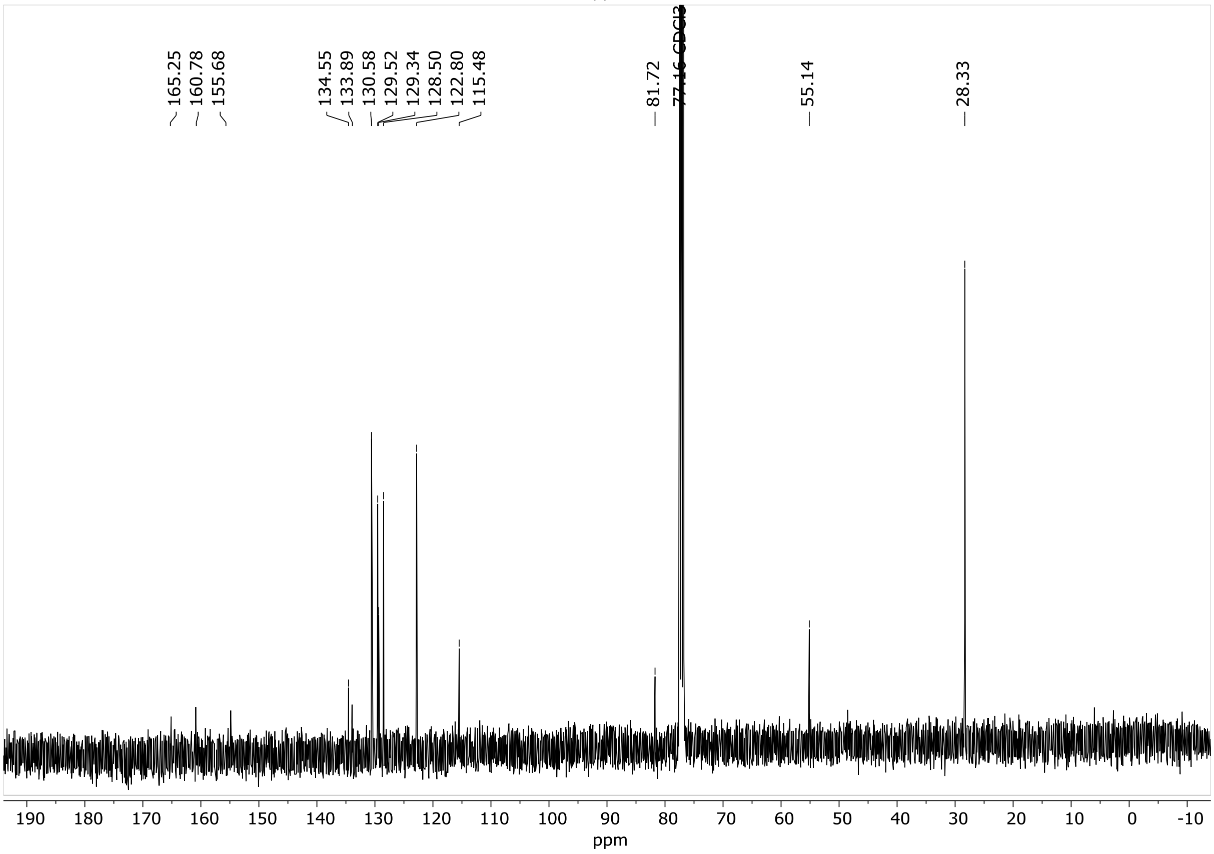

^{1}H NMR and ^{13}C NMR spectrum of compound **4m**

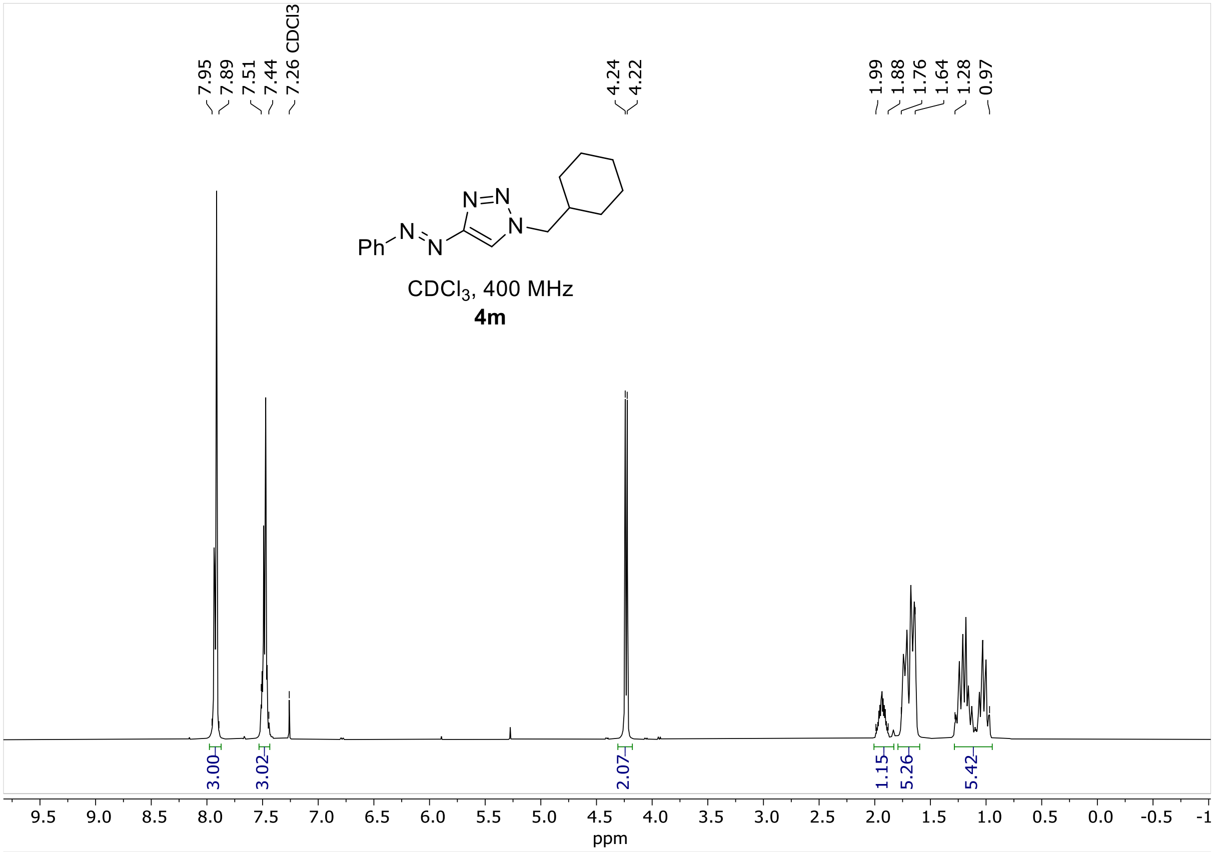

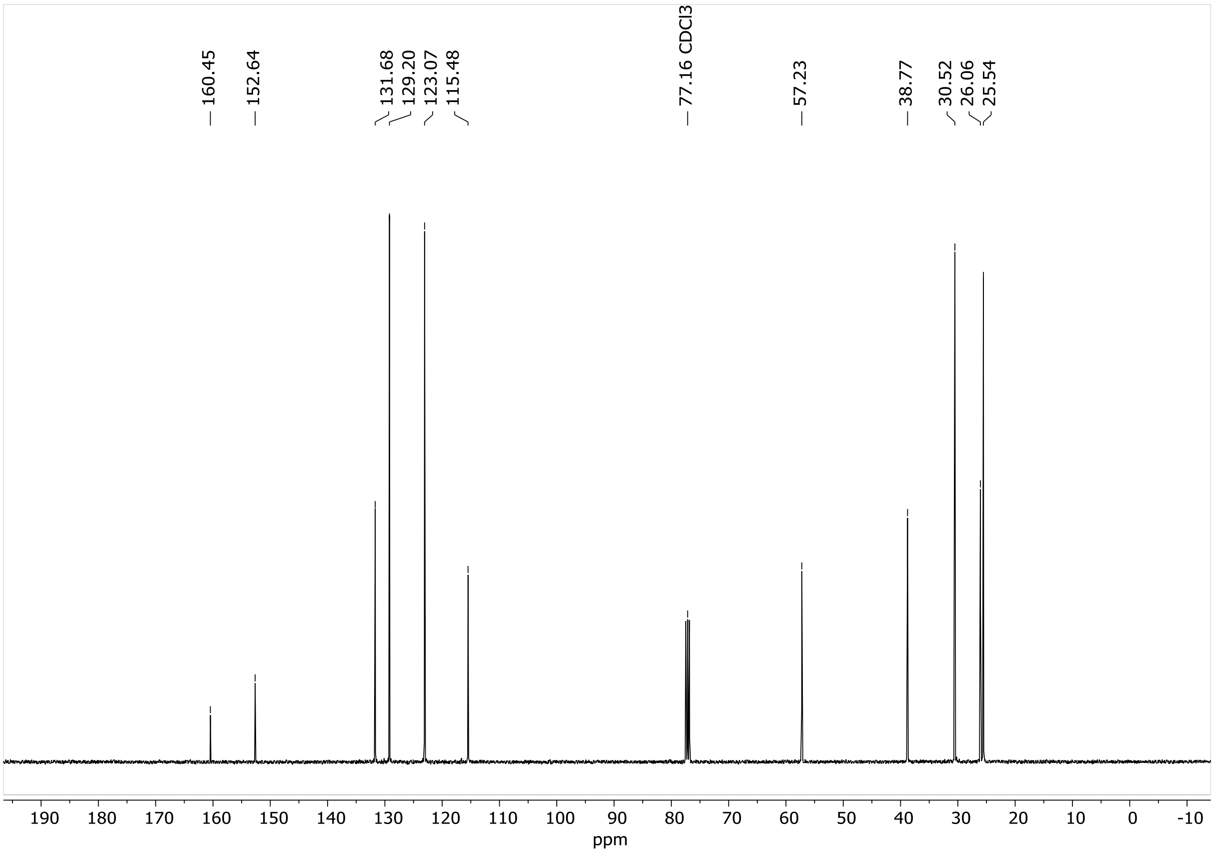

^{1}H NMR and ^{13}C NMR spectrum of compound **4n**

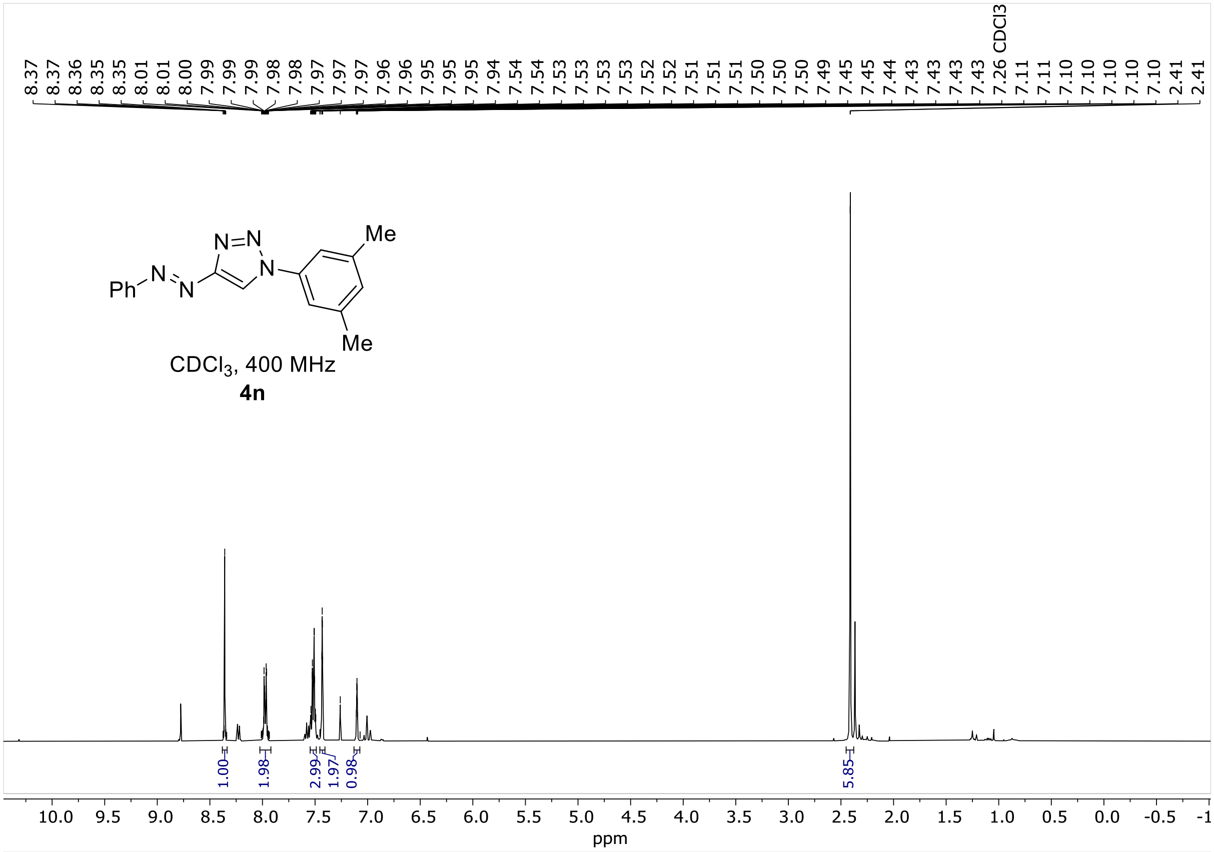

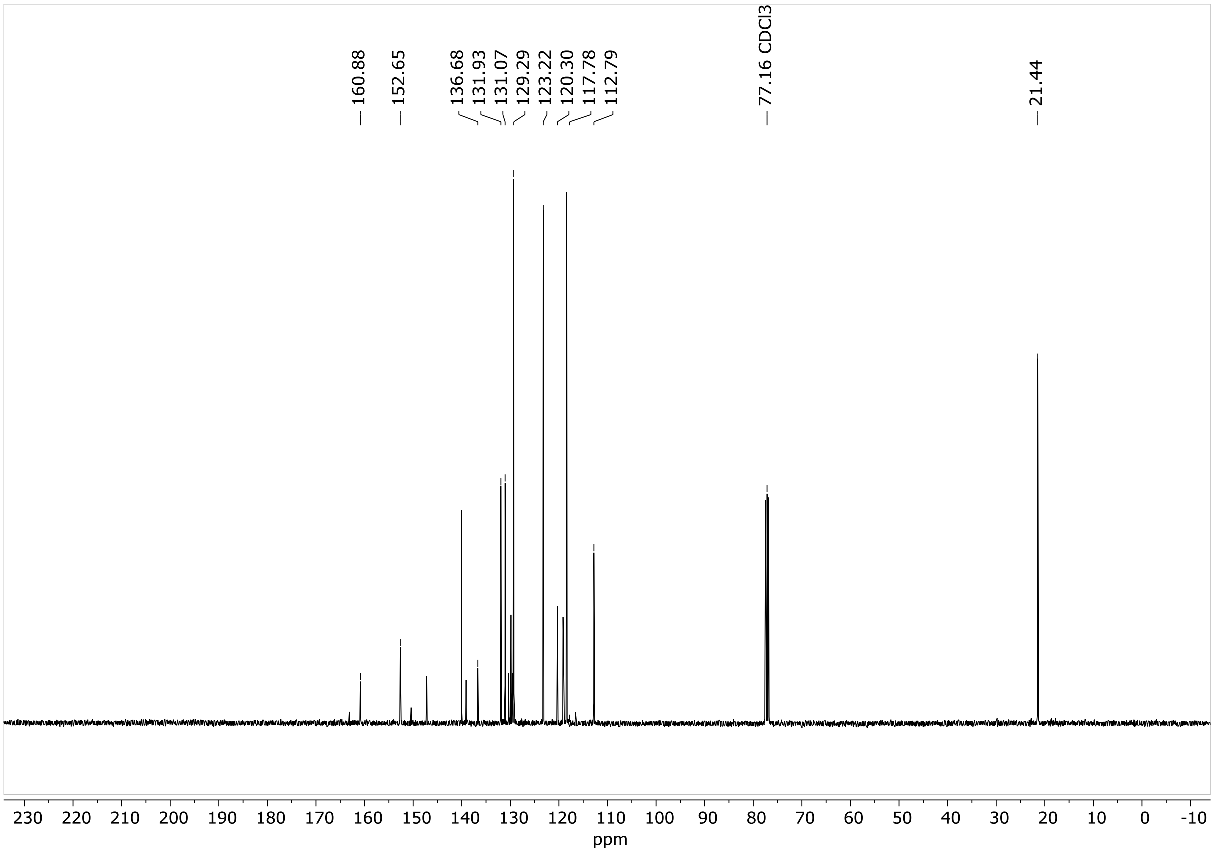

^{1}H NMR ^{13}C NMR and ^{19}F spectrum of compound **4o**

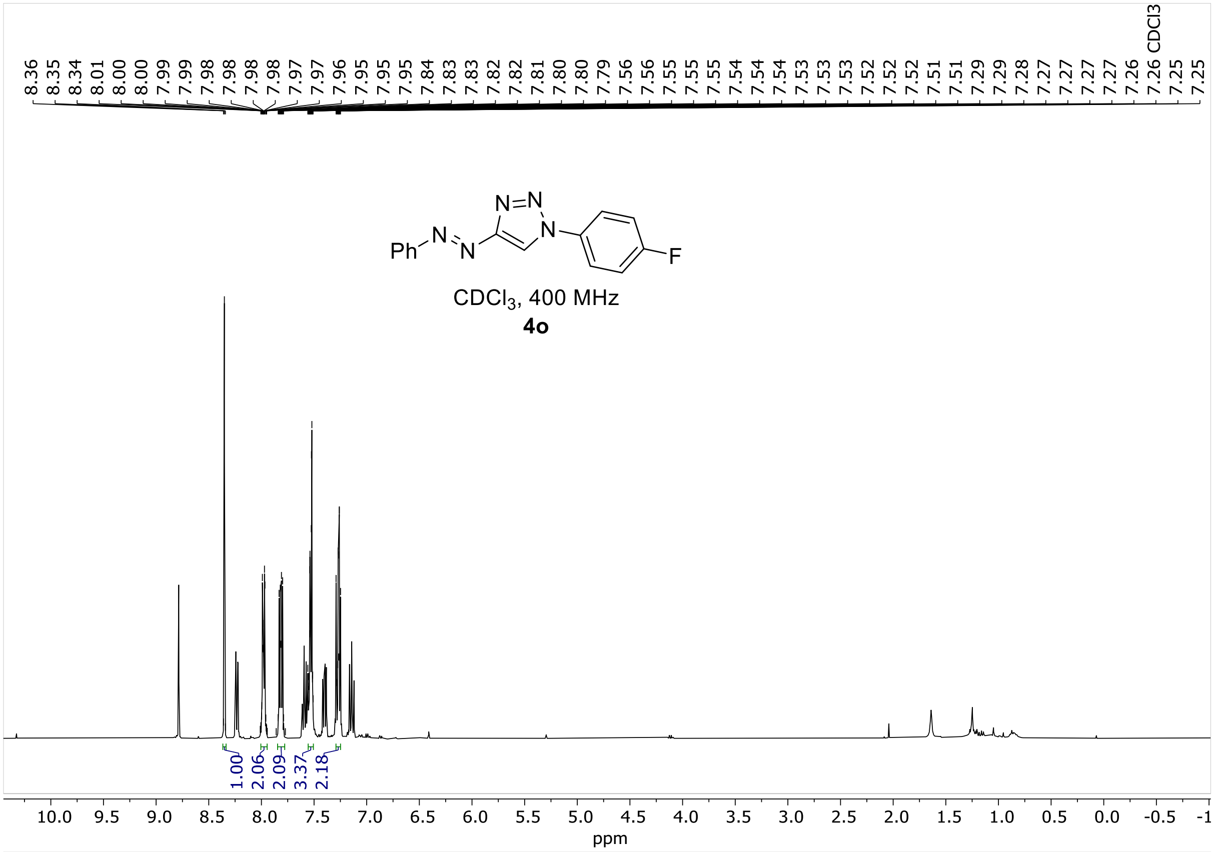

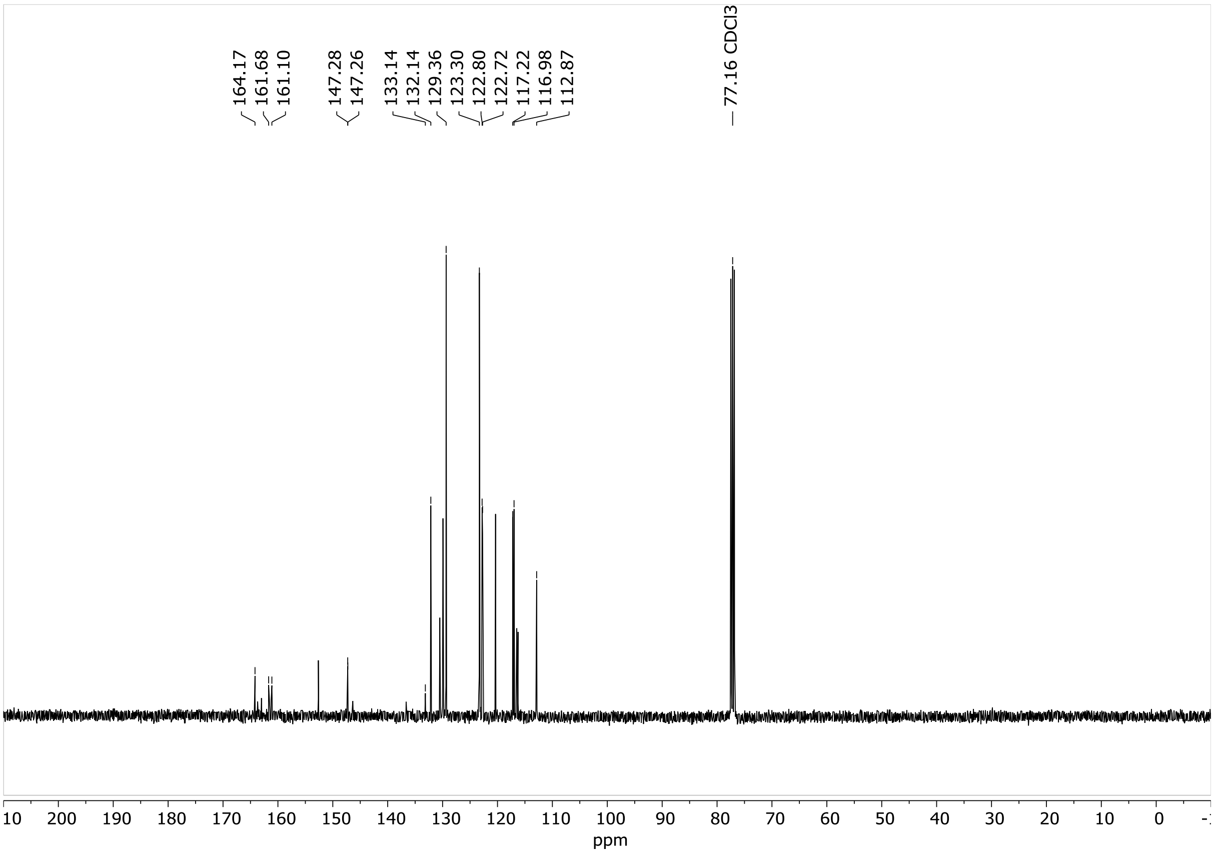

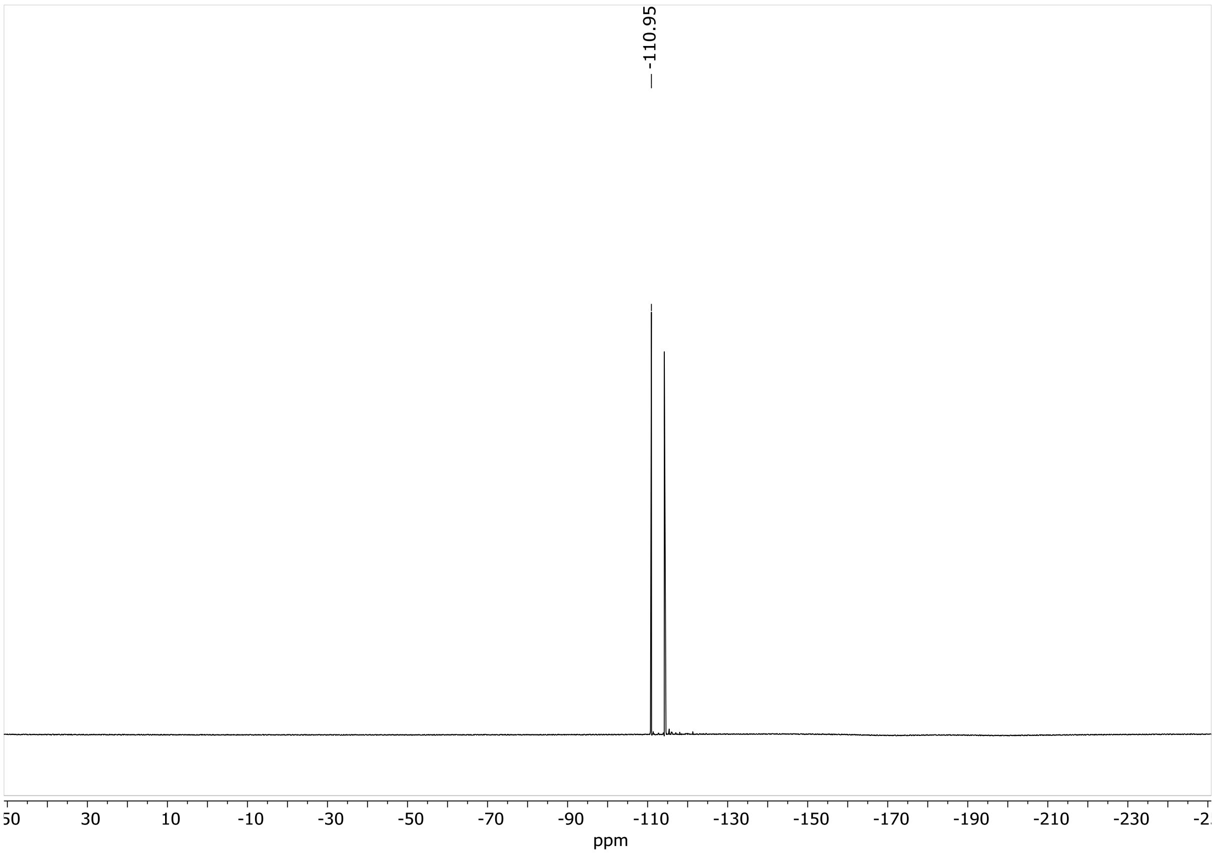
-110.95
50
30
10
-10
-30
-50
-70
-90
-110
-130
-150
-170
-190
-210
-230
ppm

^{1}H NMR and ^{13}C NMR spectrum of compound **4p**

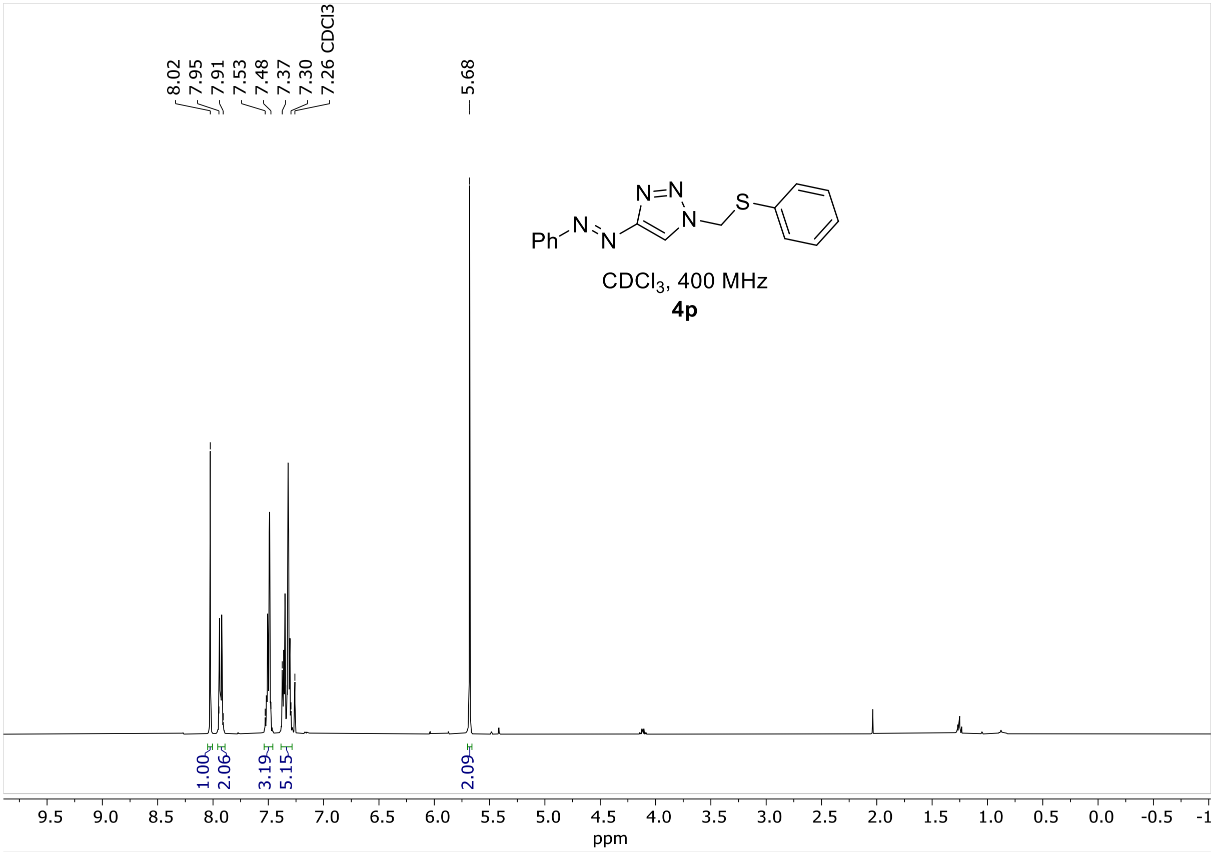

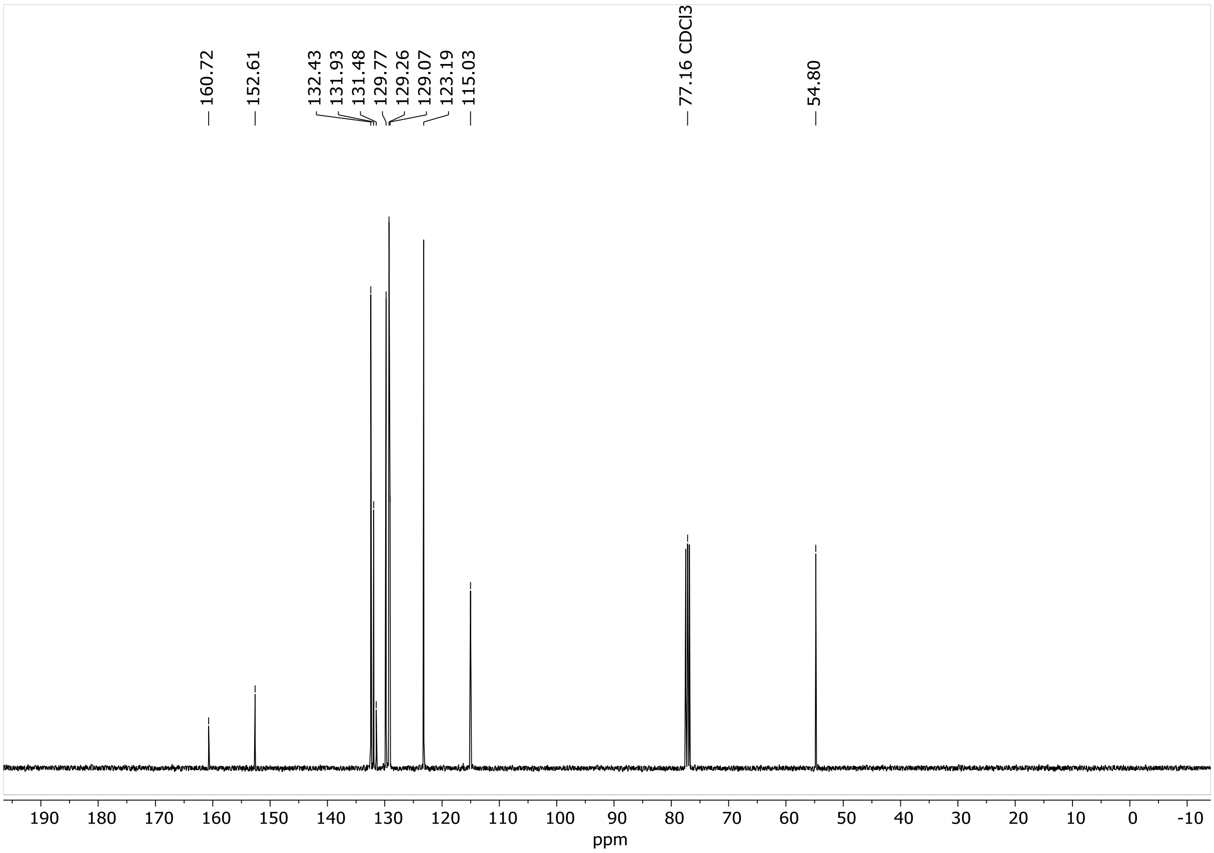

^{1}H NMR and ^{13}C NMR spectrum of compound **4q**

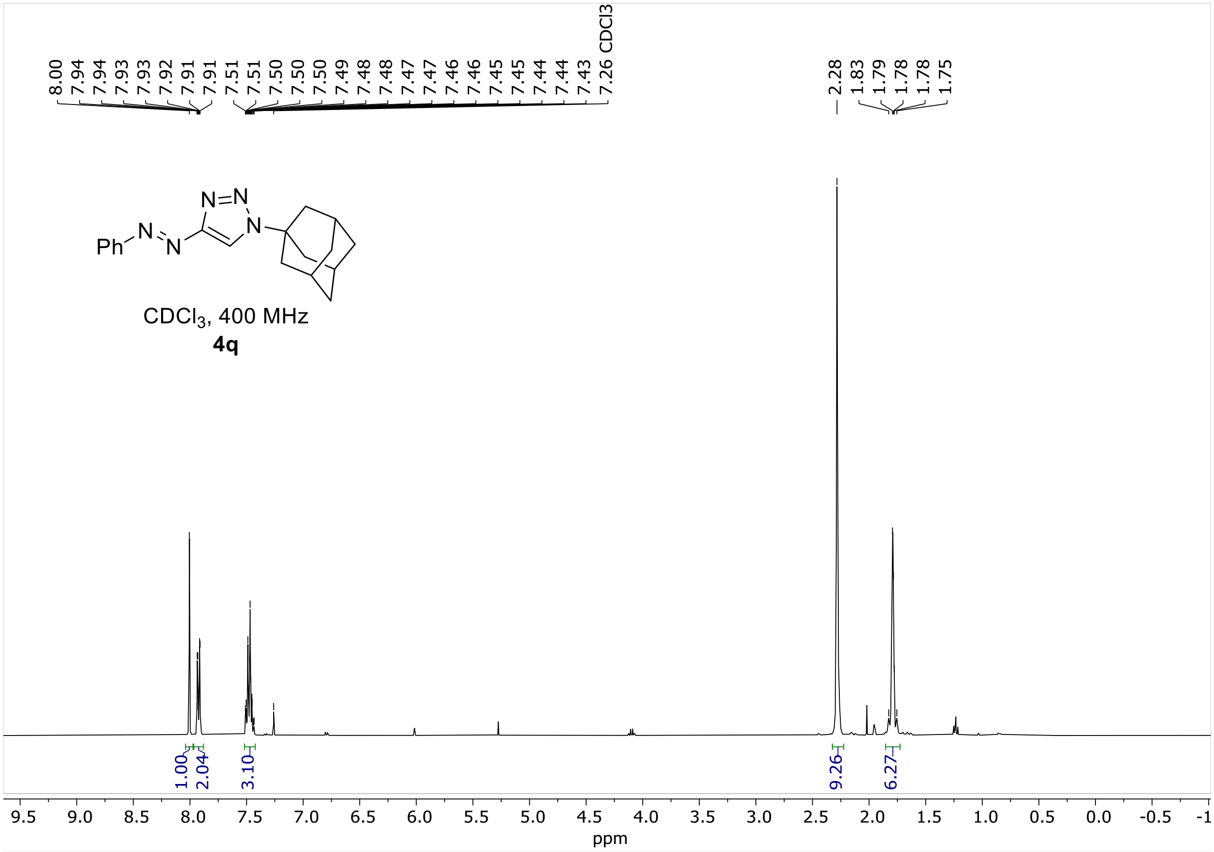

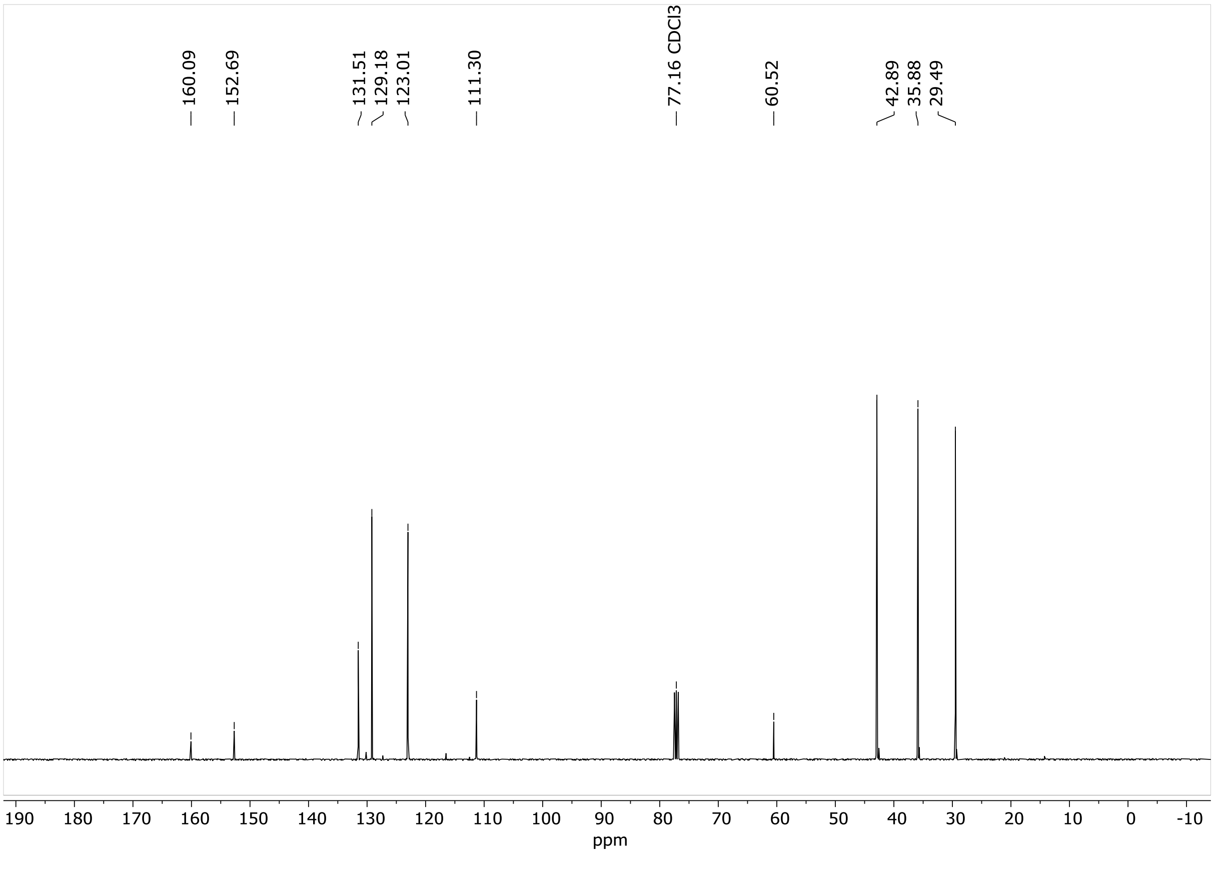

^{1}H NMR and ^{13}C NMR spectrum of compound **5a**

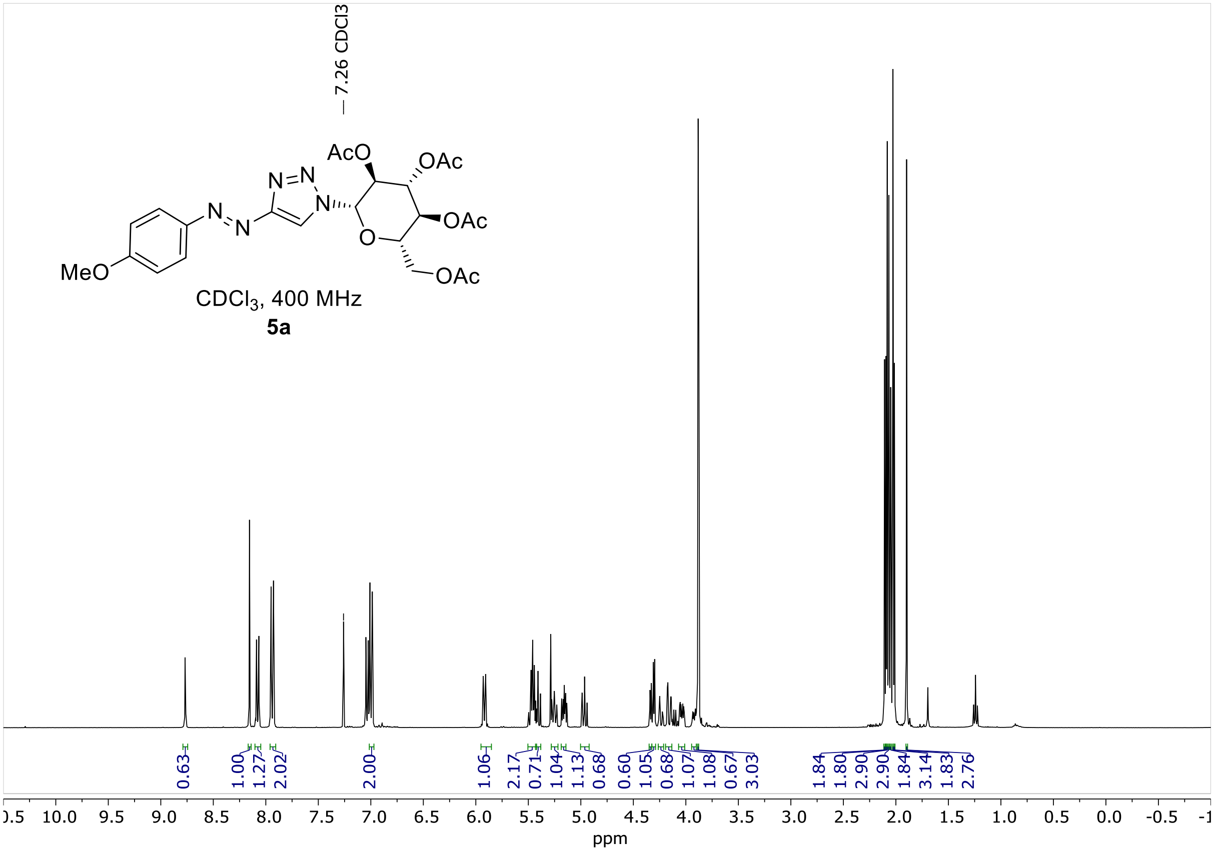

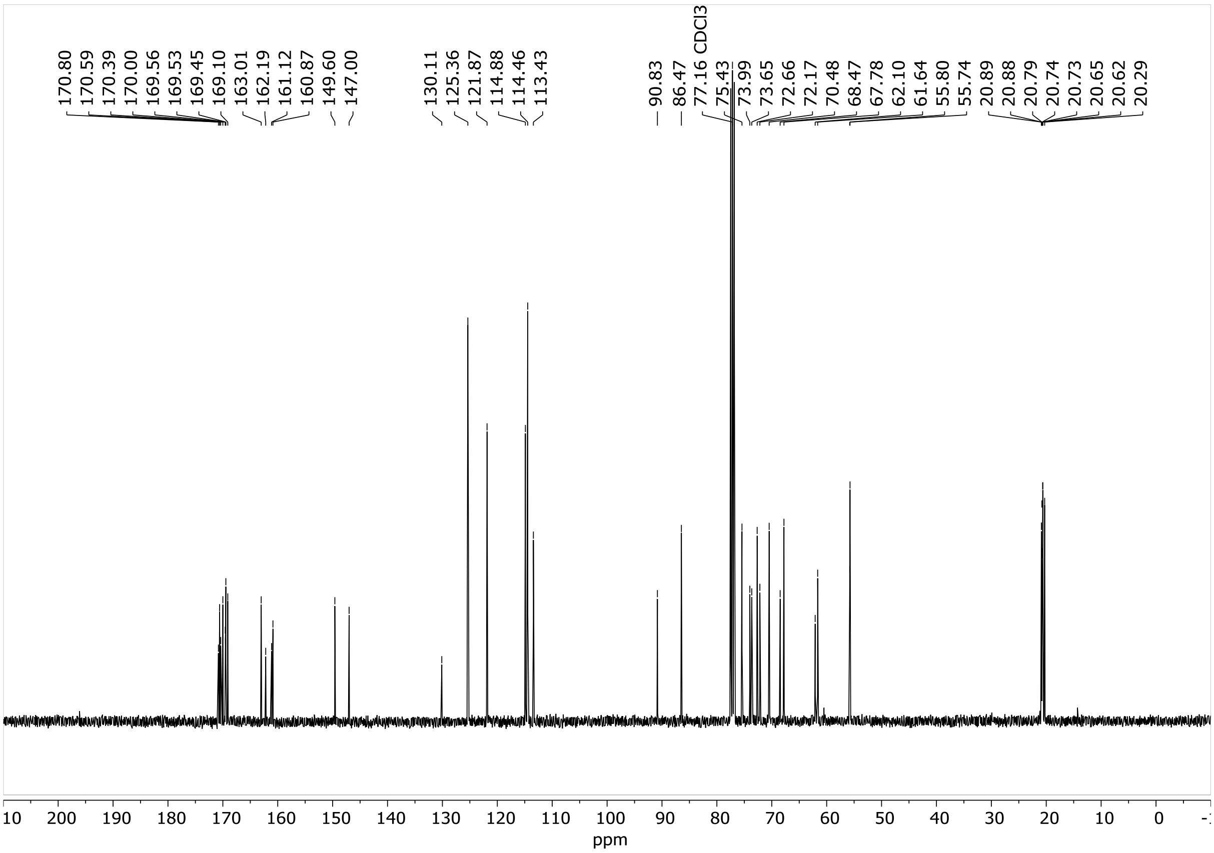

^{1}H NMR and ^{13}C NMR spectrum of compound **5b**

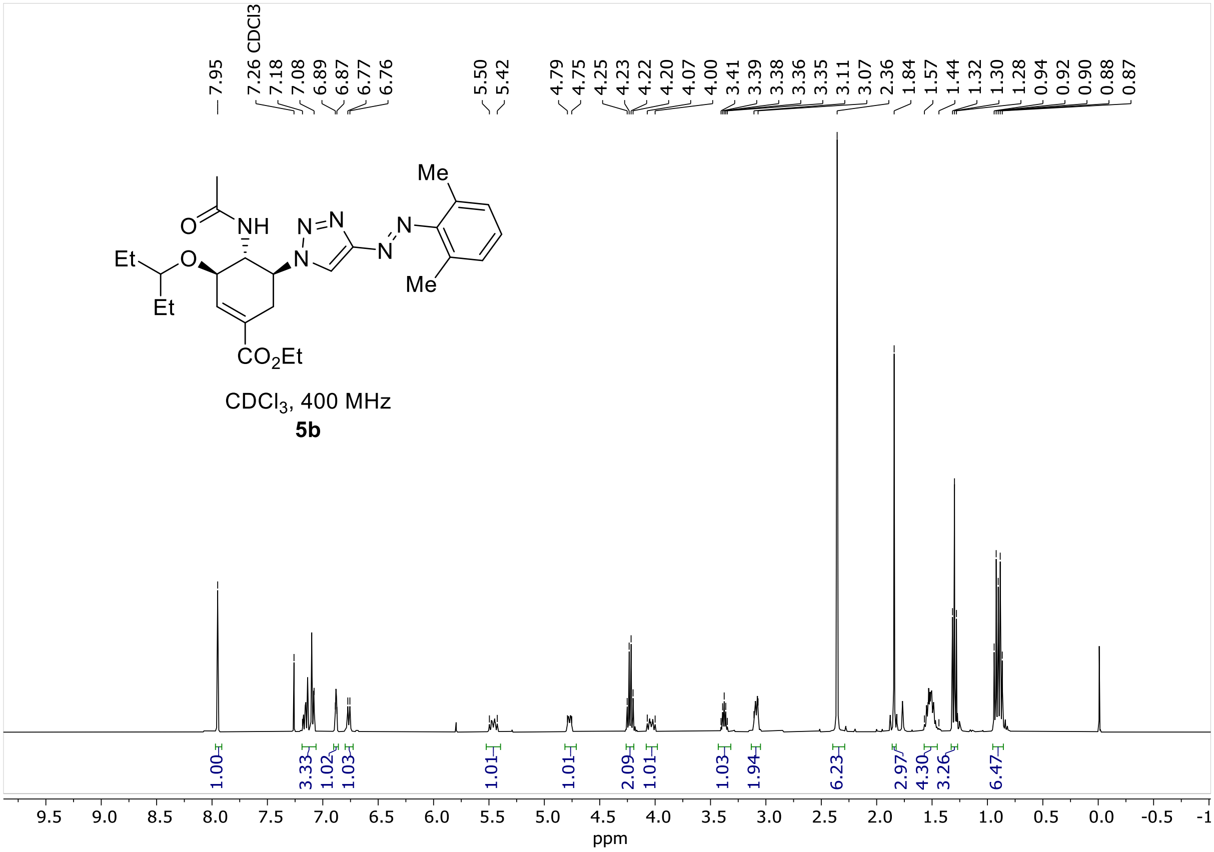

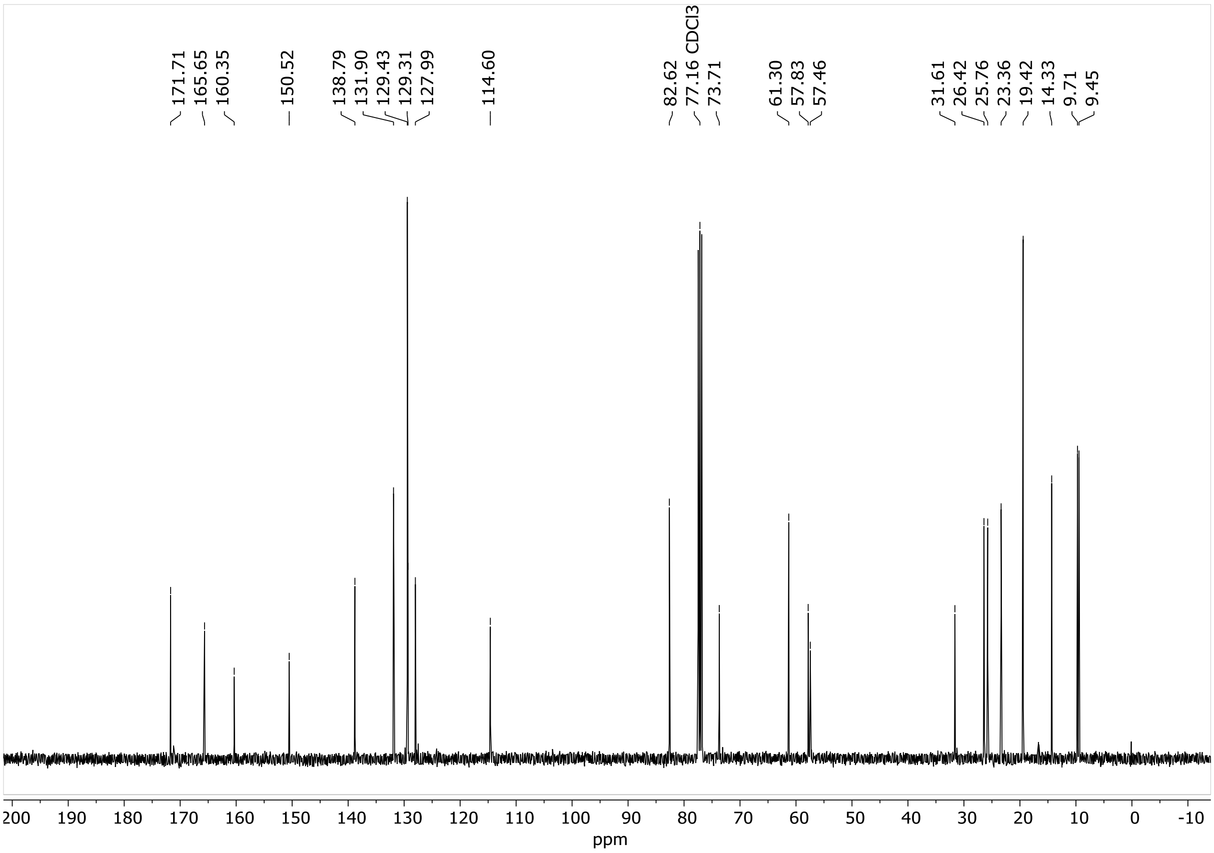

^{1}H NMR and ^{13}C NMR spectrum of compound **5c**

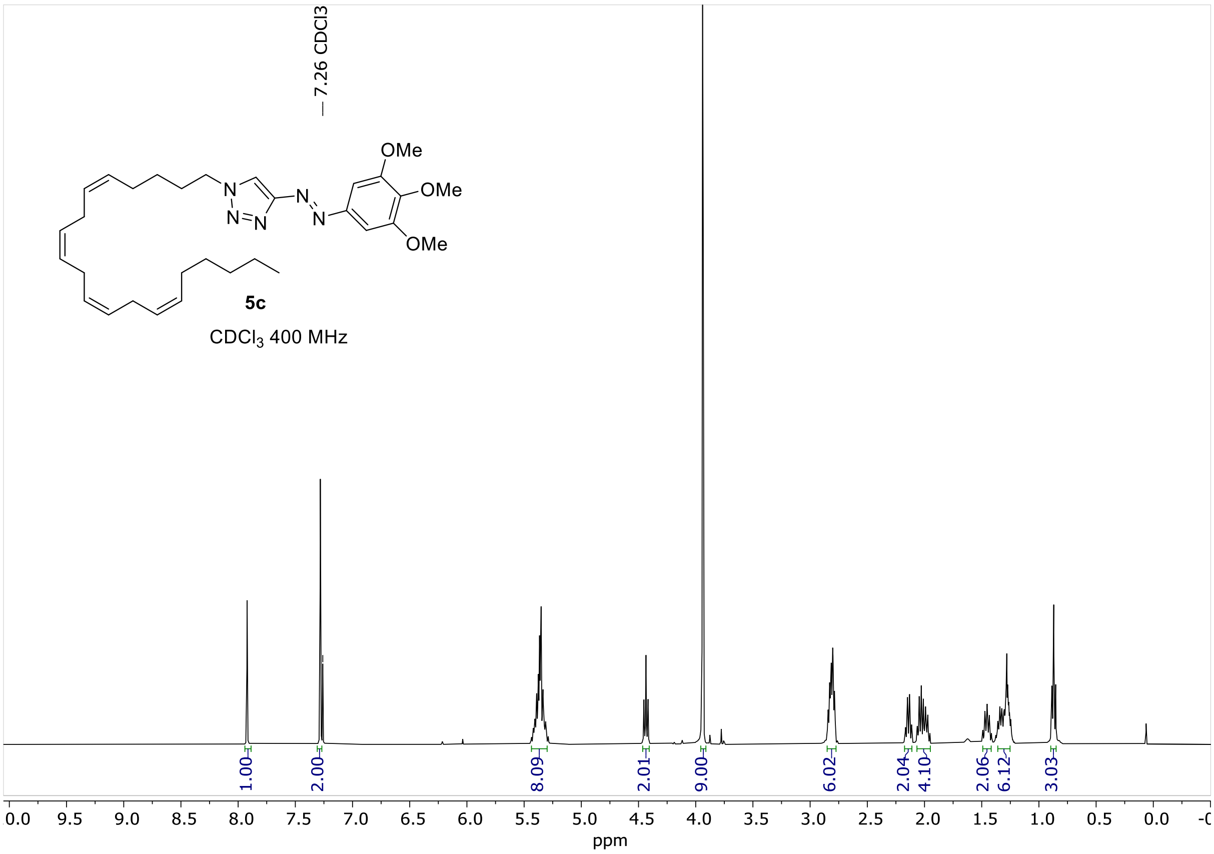

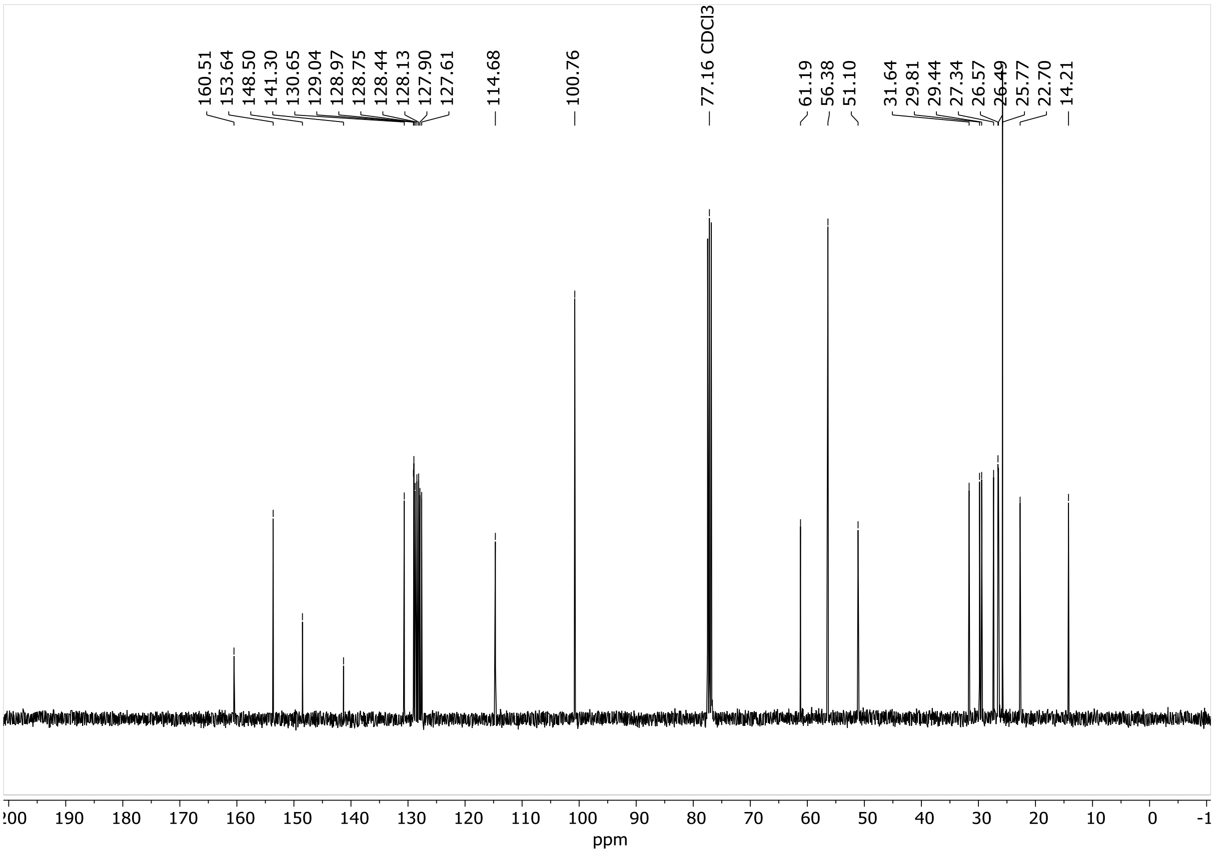

^{1}H NMR and ^{13}C NMR spectrum of compound **5d**

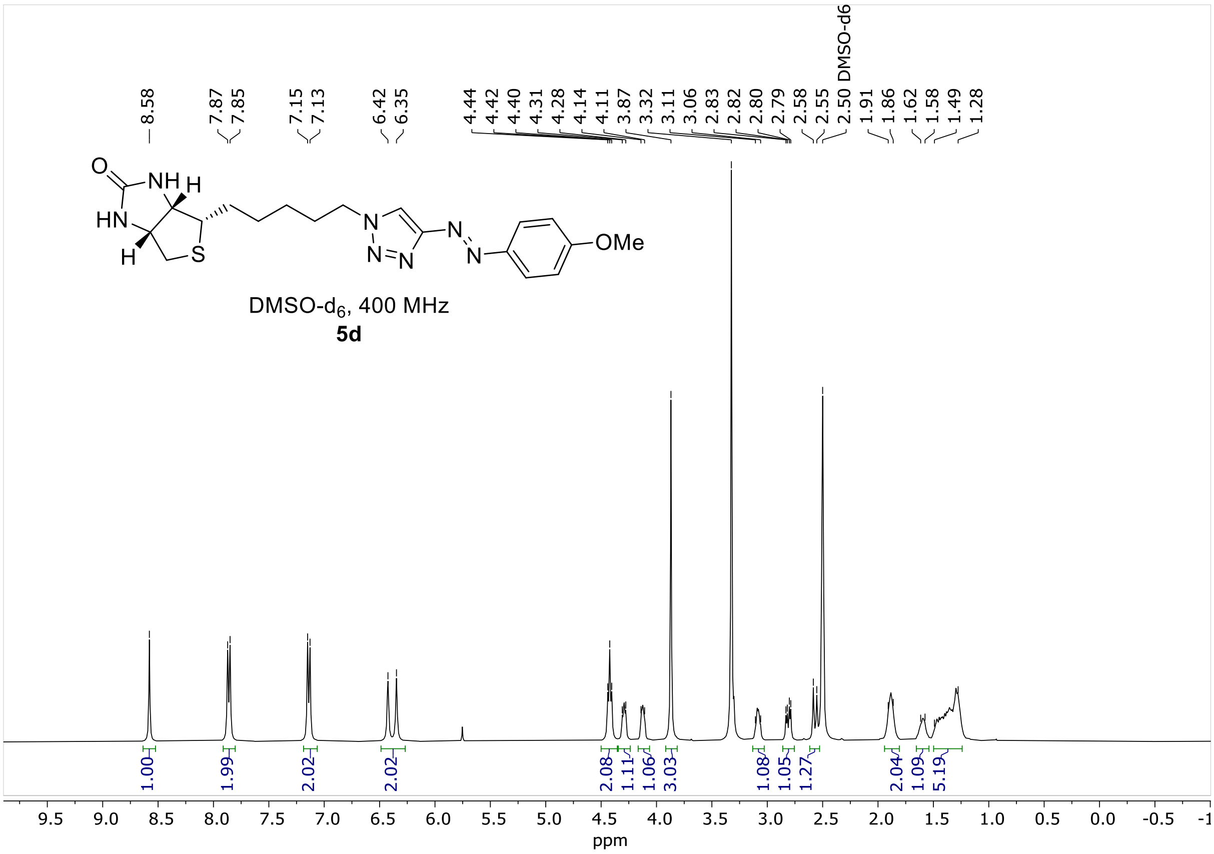

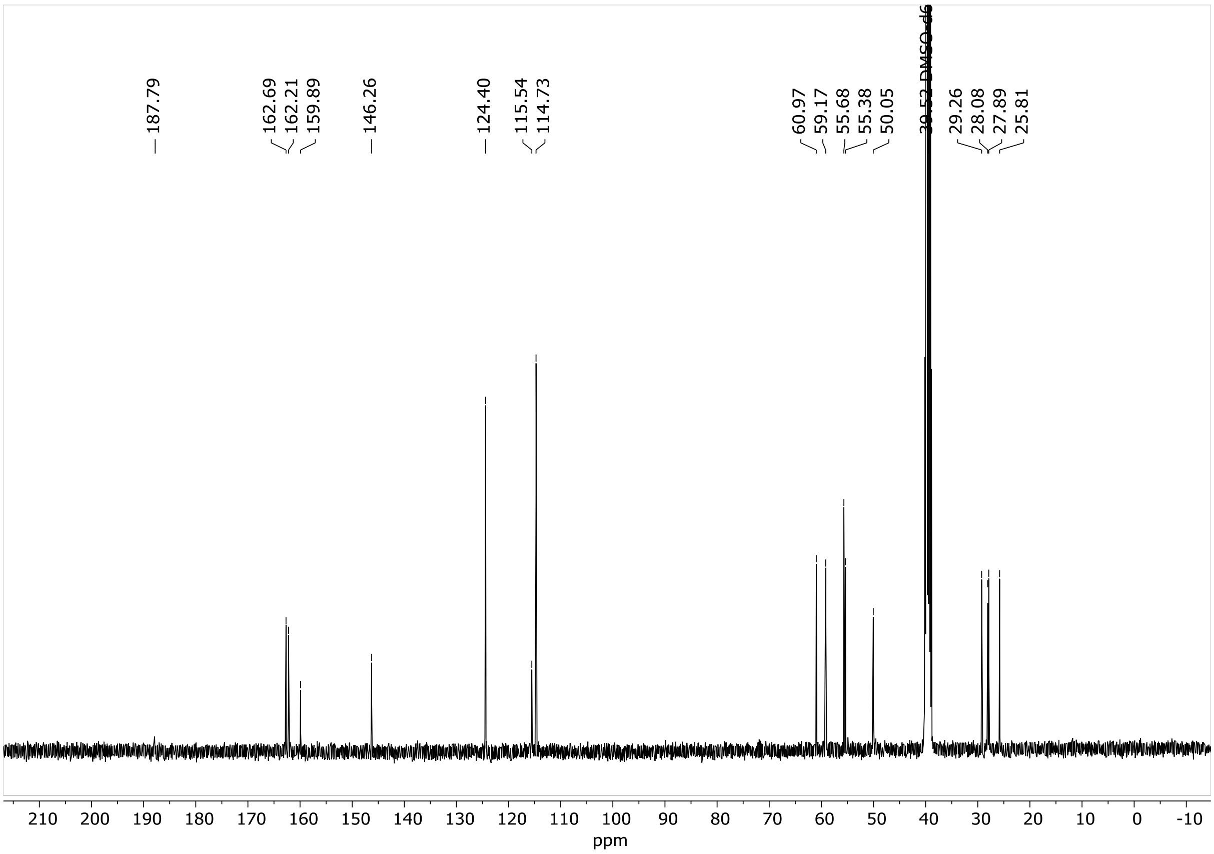

^{1}H NMR and ^{13}C NMR spectrum of compound **5e**

Note: $CDCl_3$ contained TMS as internal reference (Peak at 0.00 ppm).

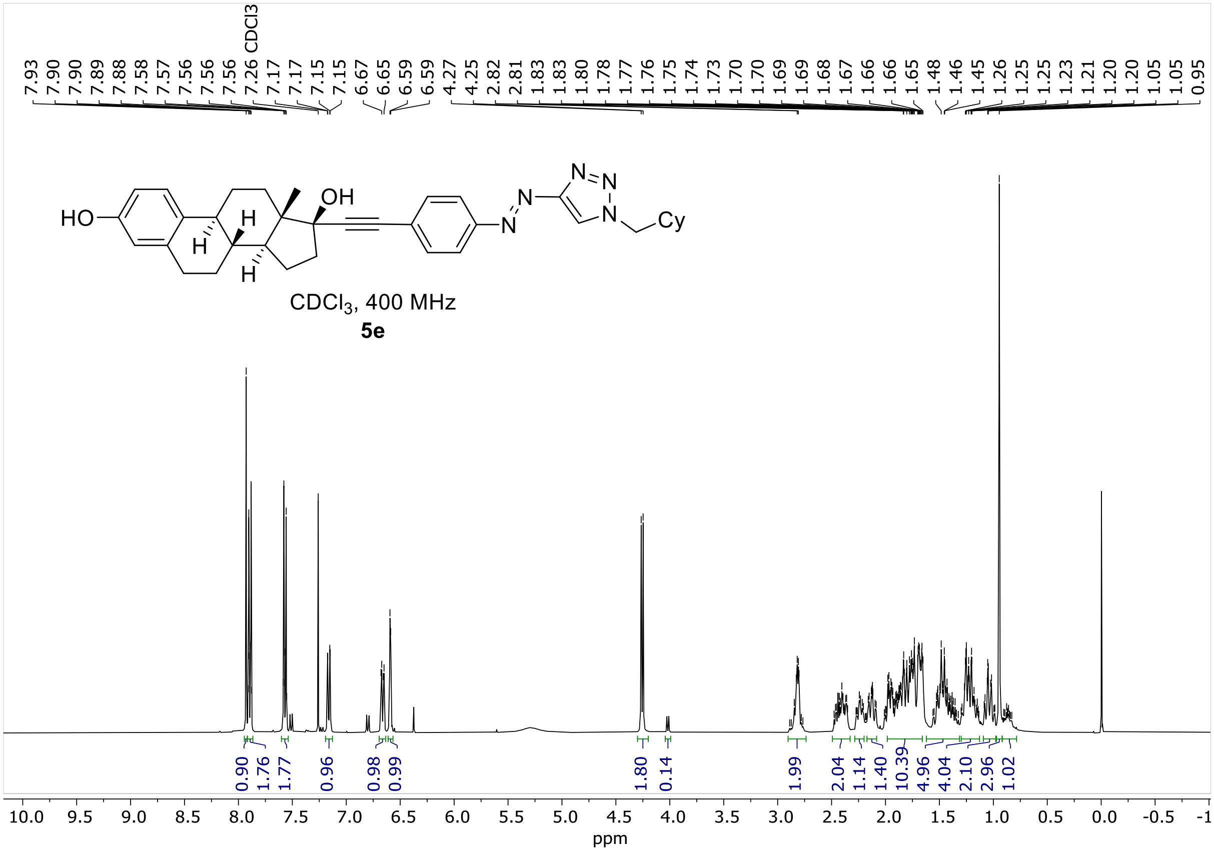

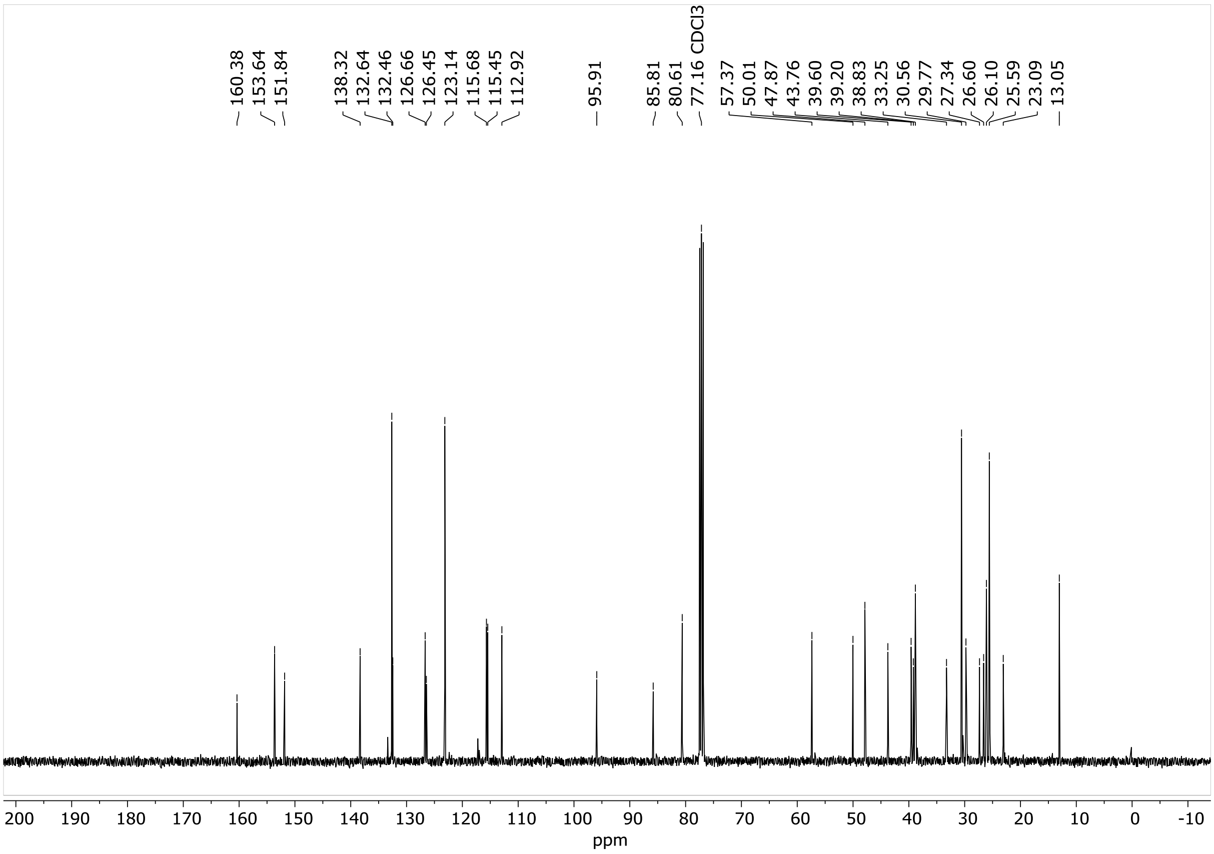

^{1}H NMR and ^{13}C NMR spectrum of compound **5f**

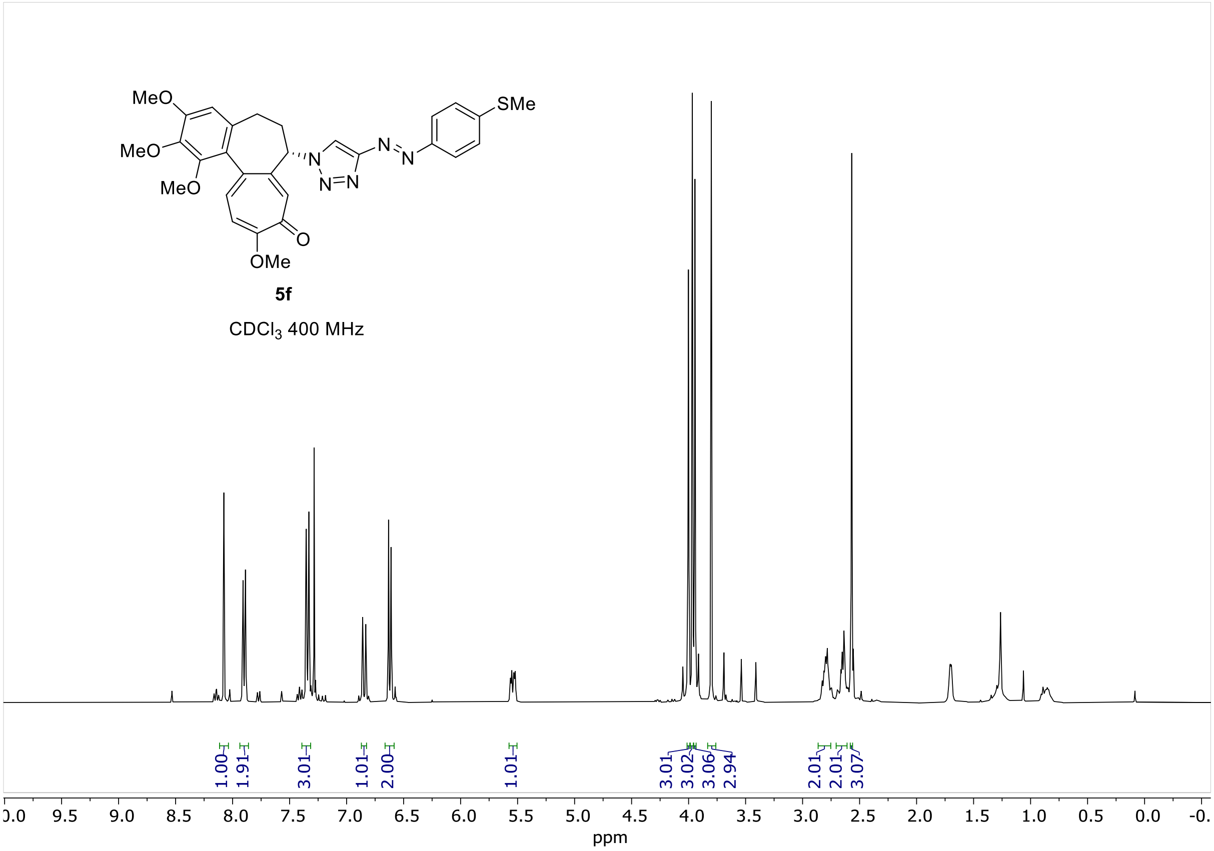

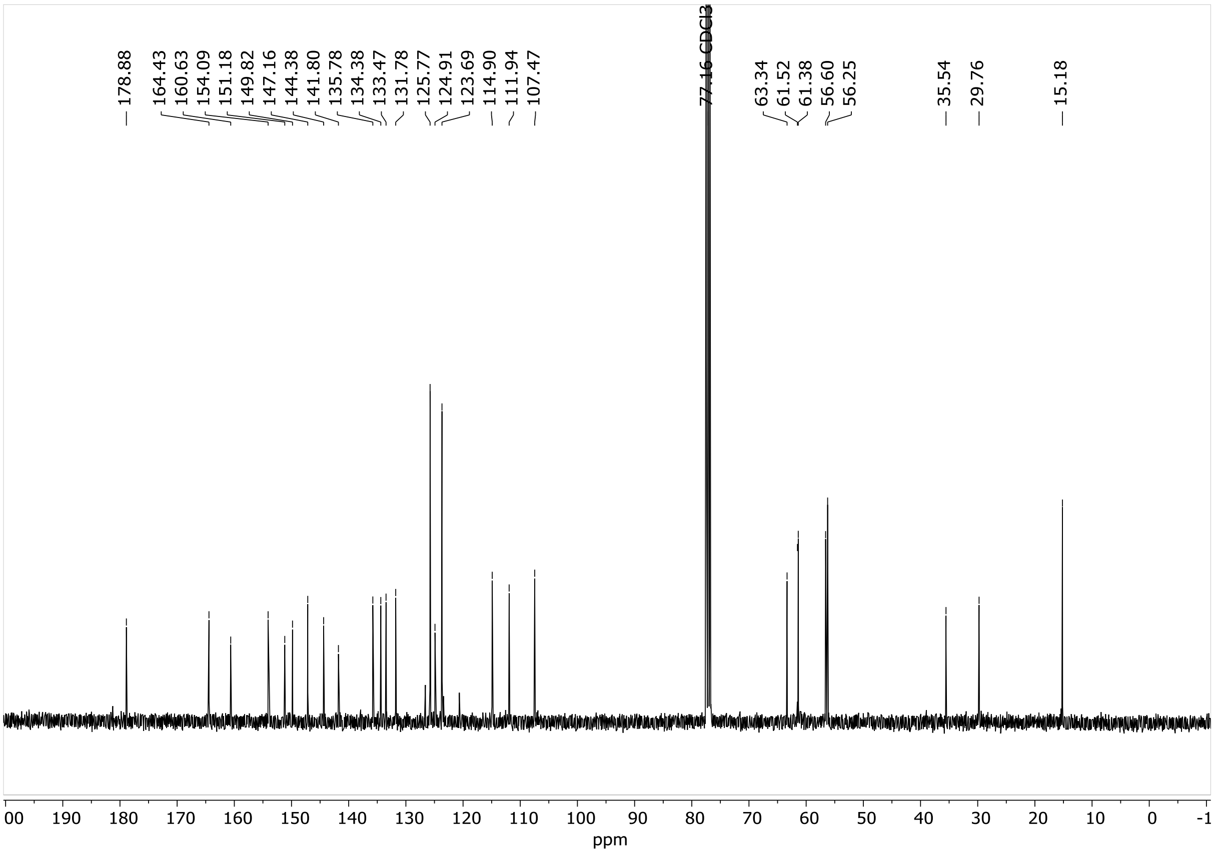

^1H NMR and ^{13}C NMR spectrum of compound **5g**

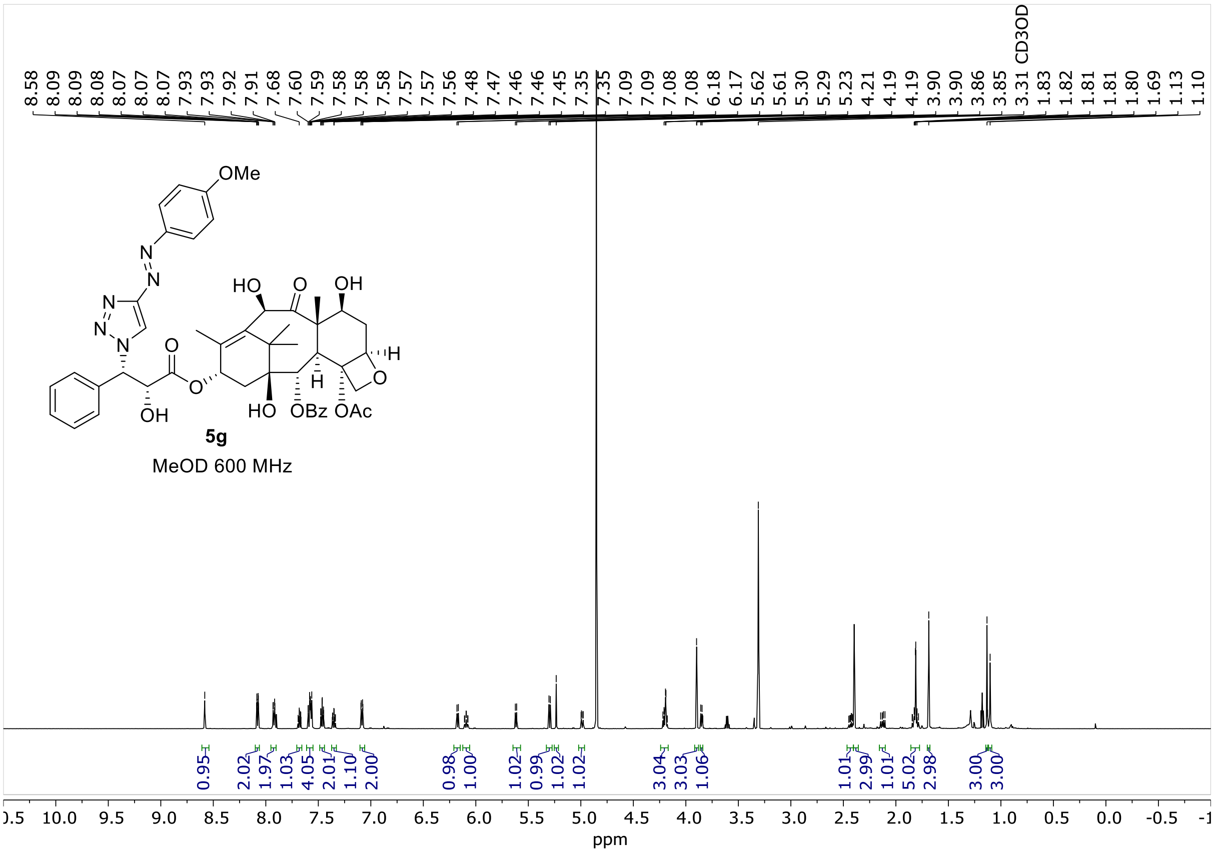

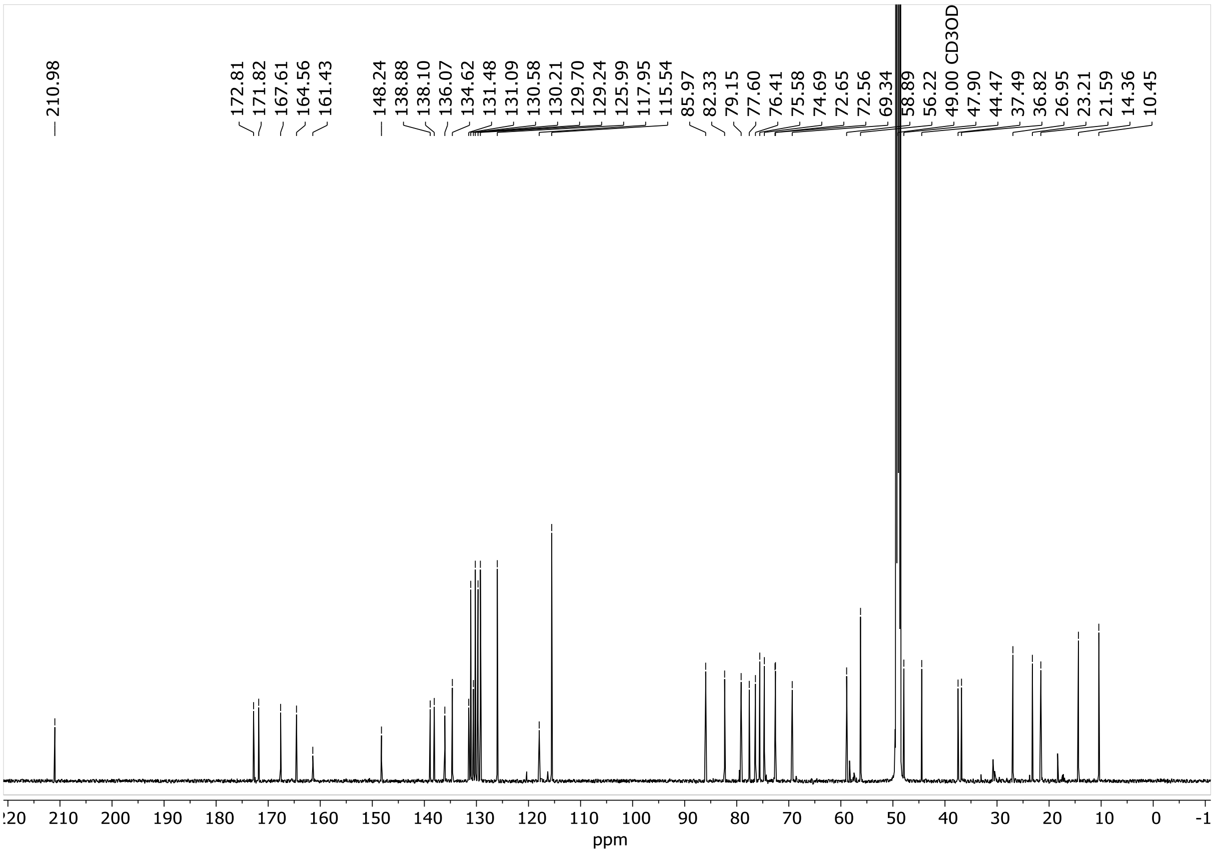

^{1}H NMR and ^{13}C NMR spectrum of compound **7-*epi*-5g**

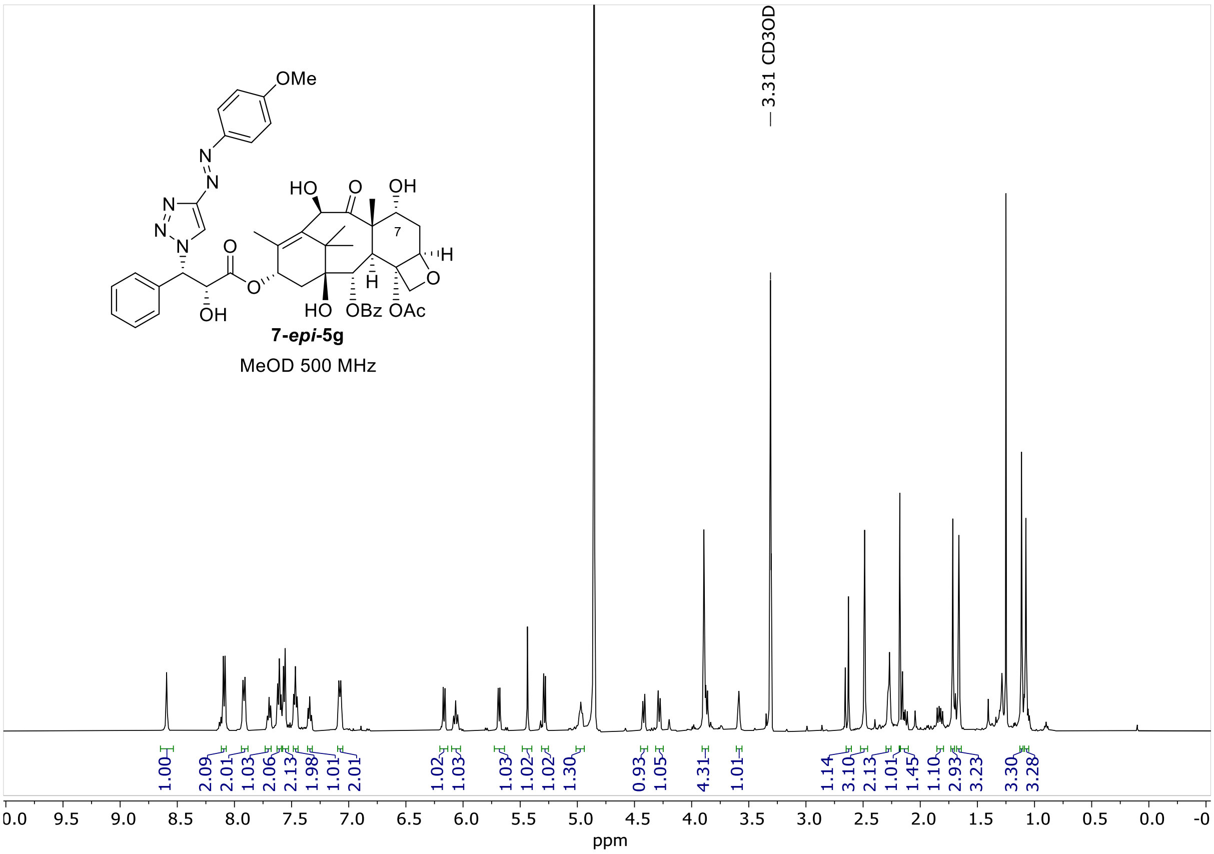

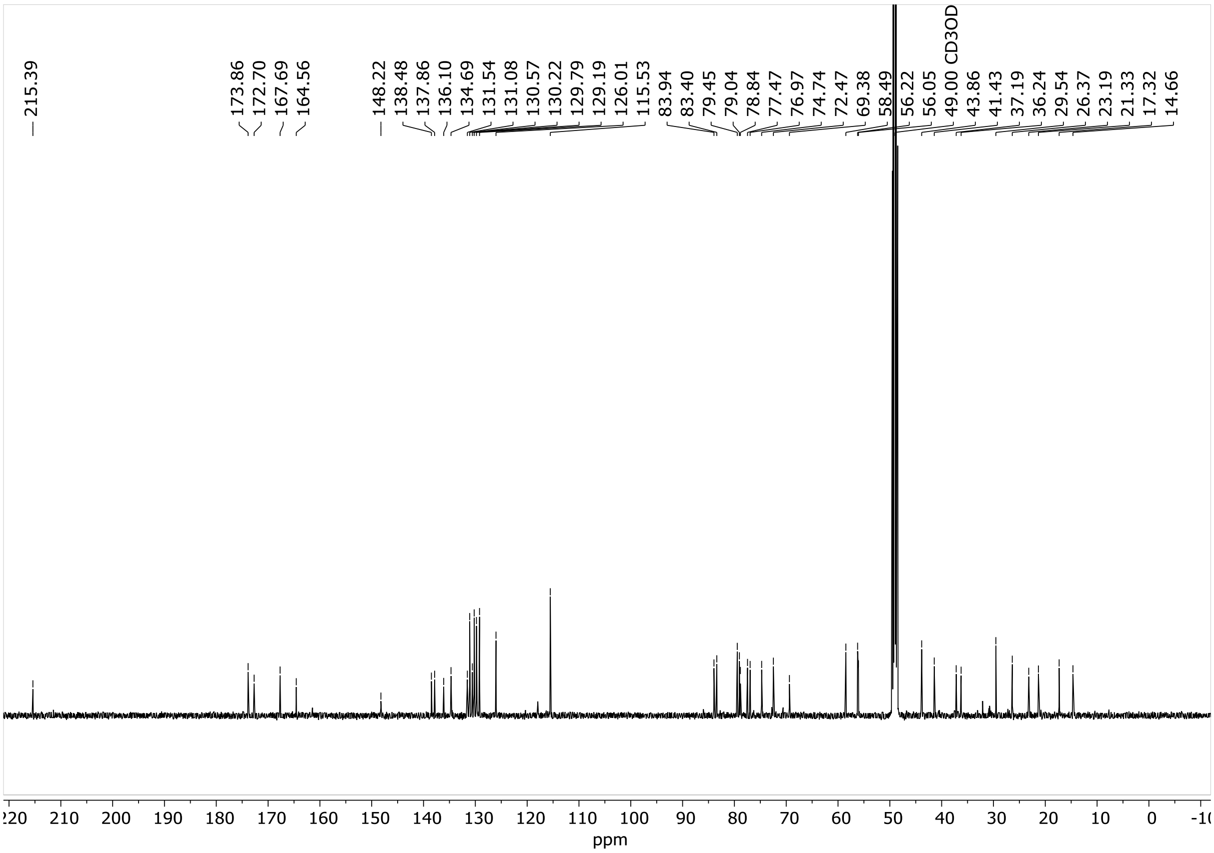

^{1}H NMR and ^{13}C NMR spectrum of compound **6d**

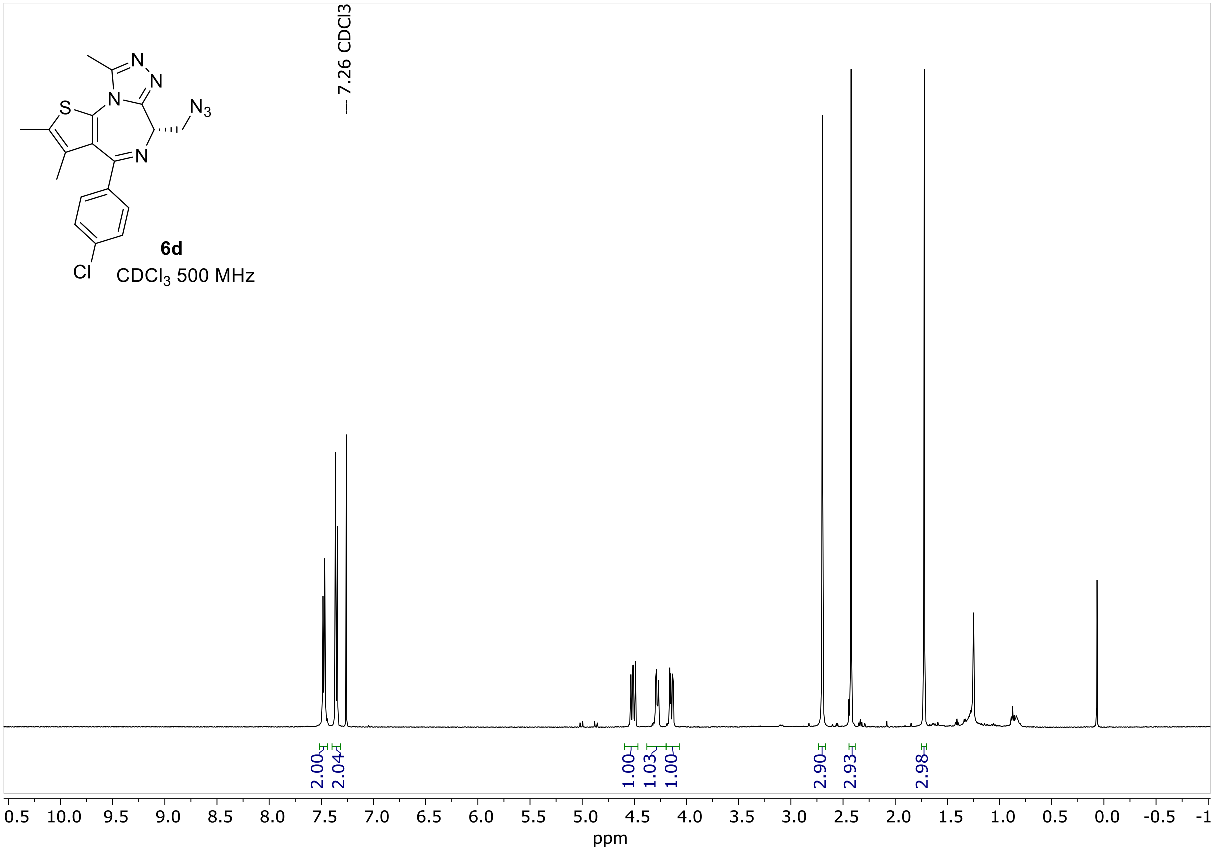

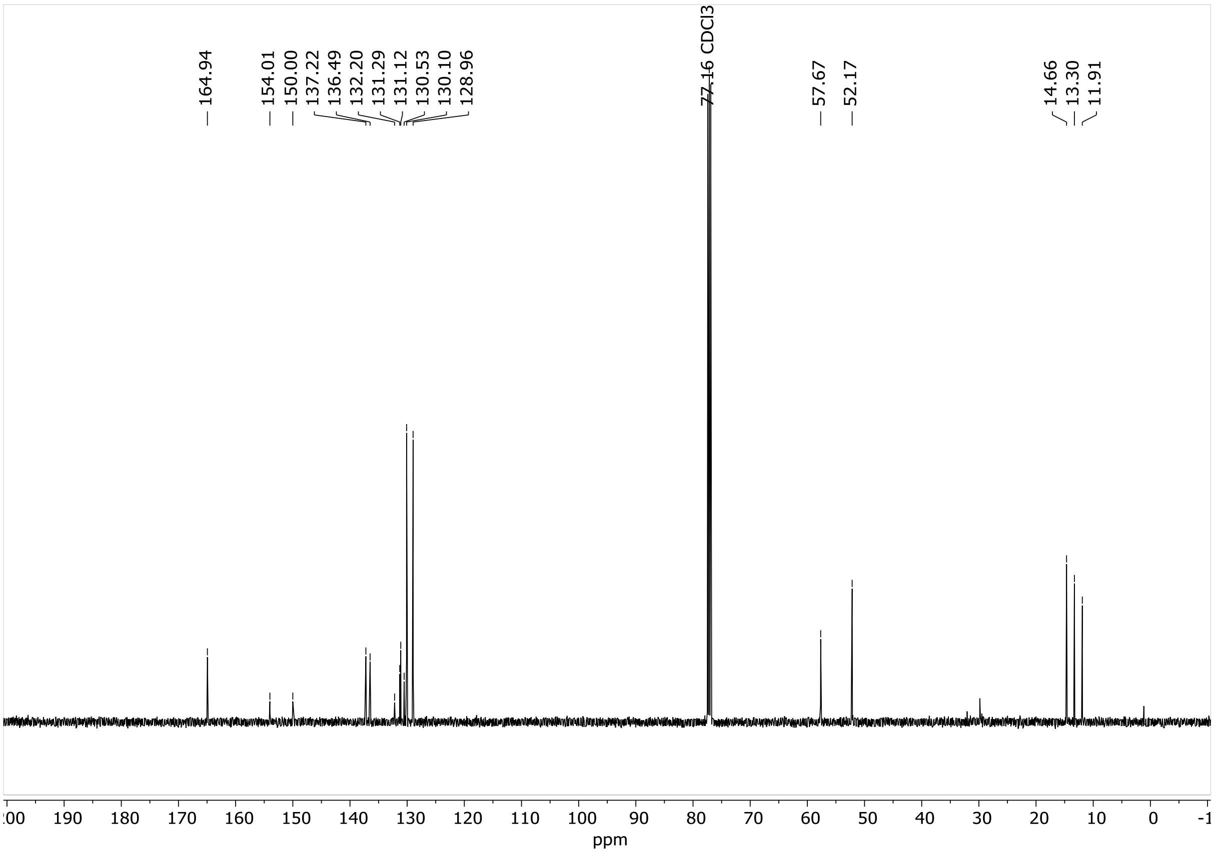

^{1}H NMR, ^{13}C NMR and ^{19}F spectra of compound **SI-2q-1**

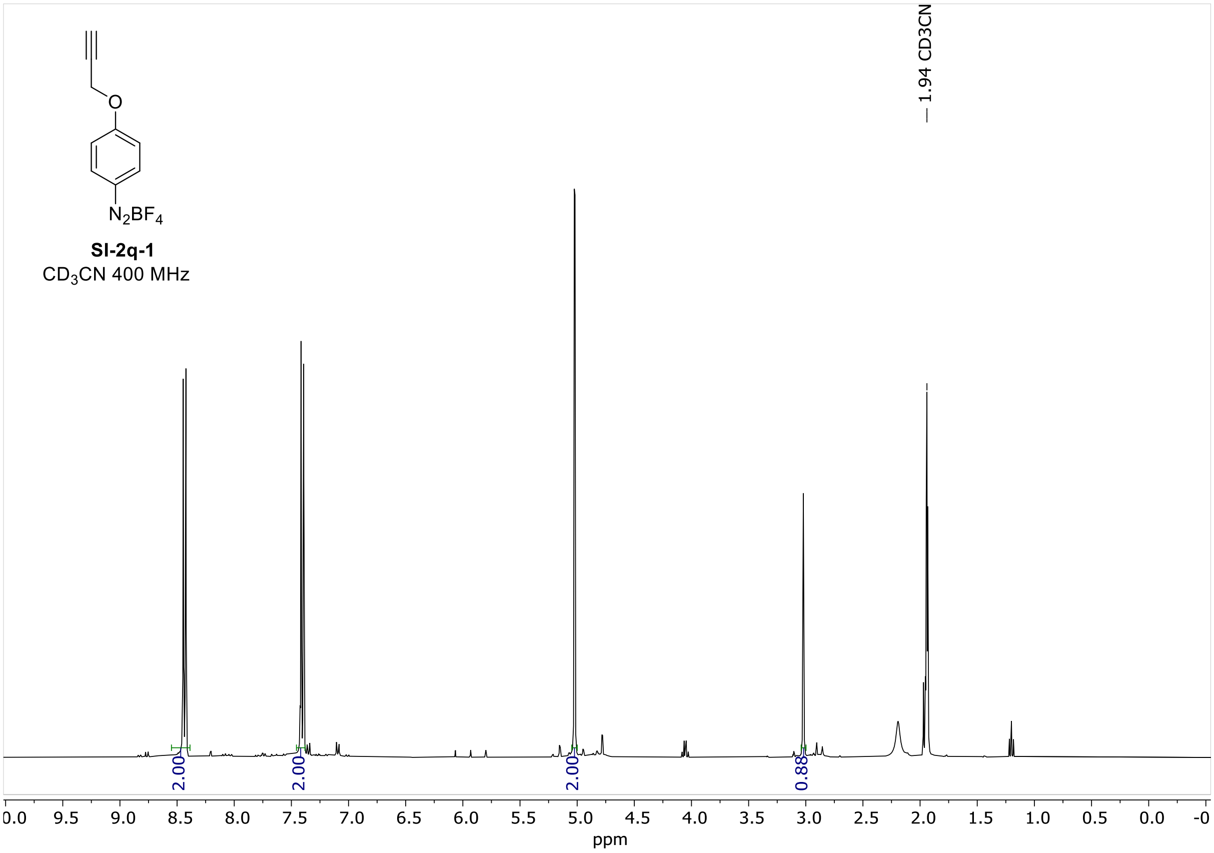

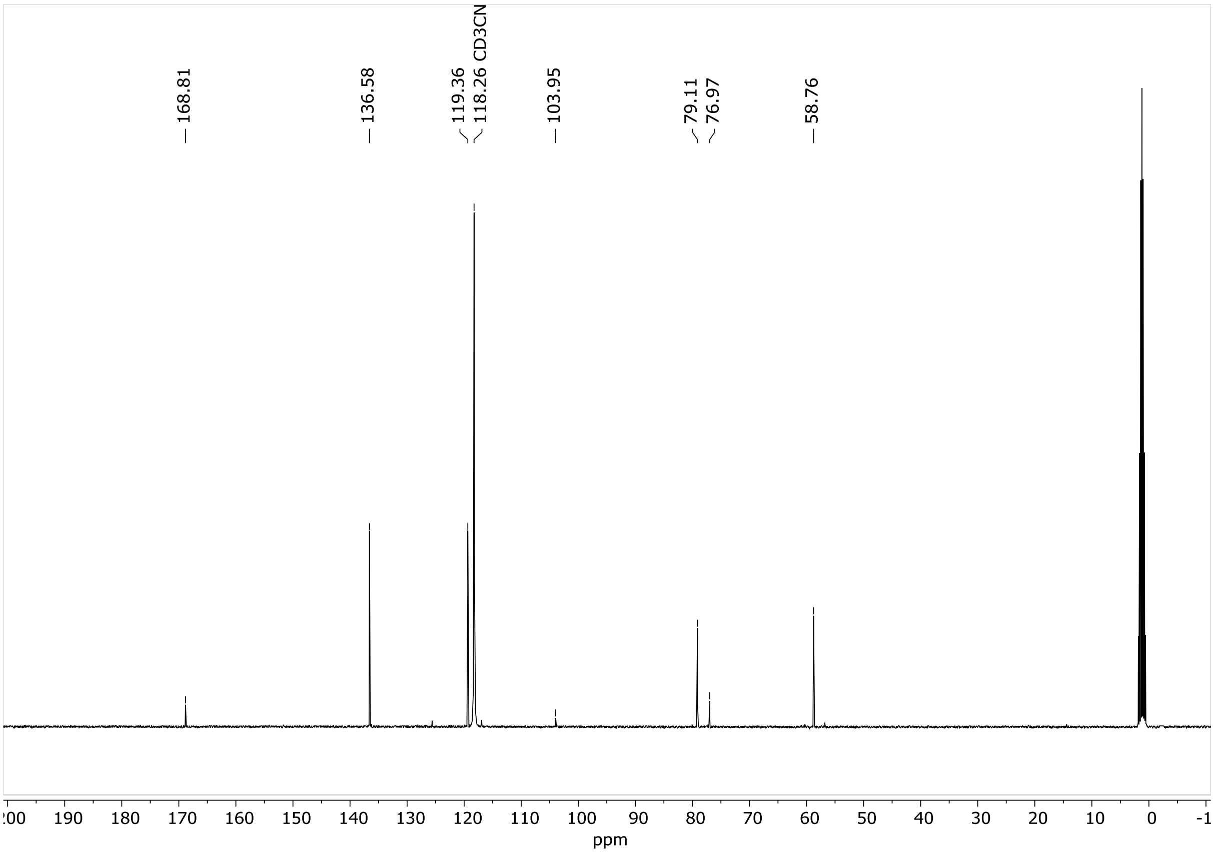

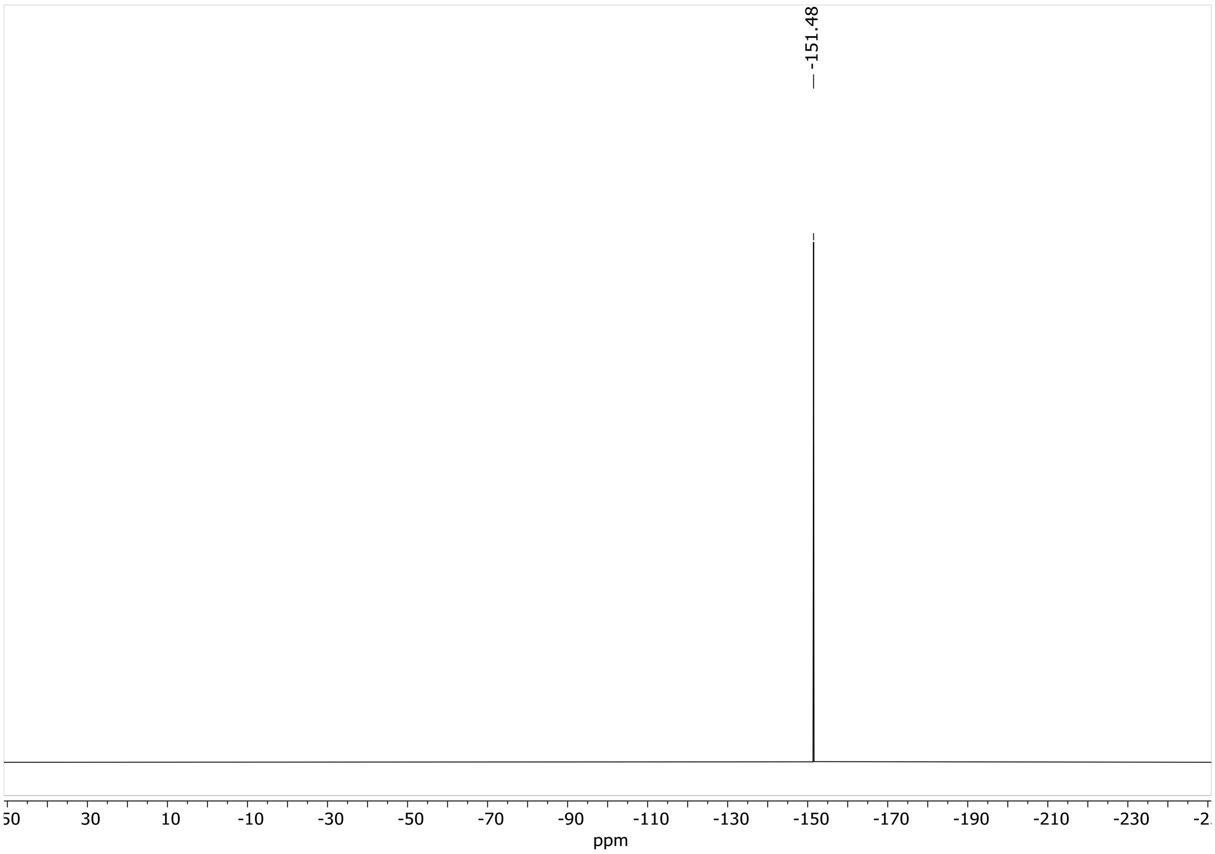
-151.48
50
30
10
-10
-30
-50
-70
-90
-110
-130
-150
-170
-190
-210
-230
ppm

^{1}H NMR and ^{13}C NMR spectrum of compound **2q**

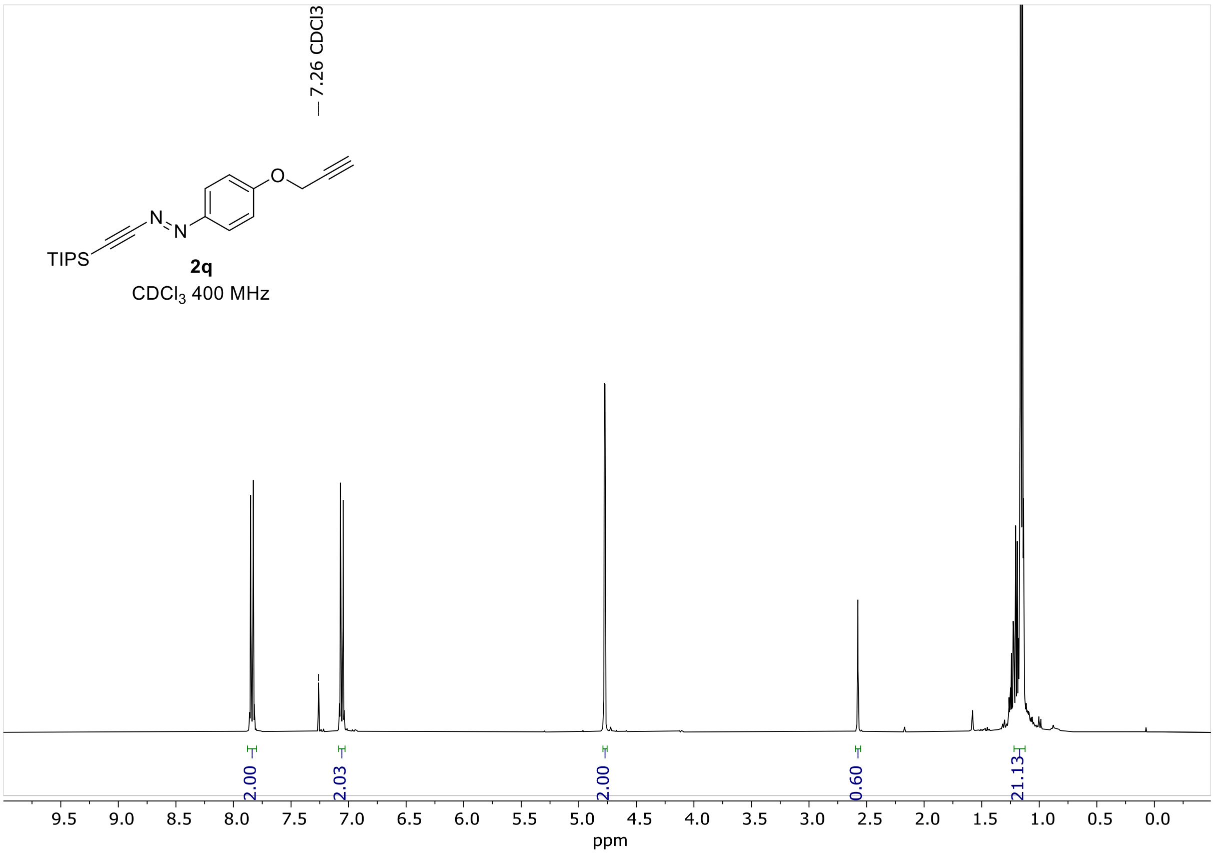

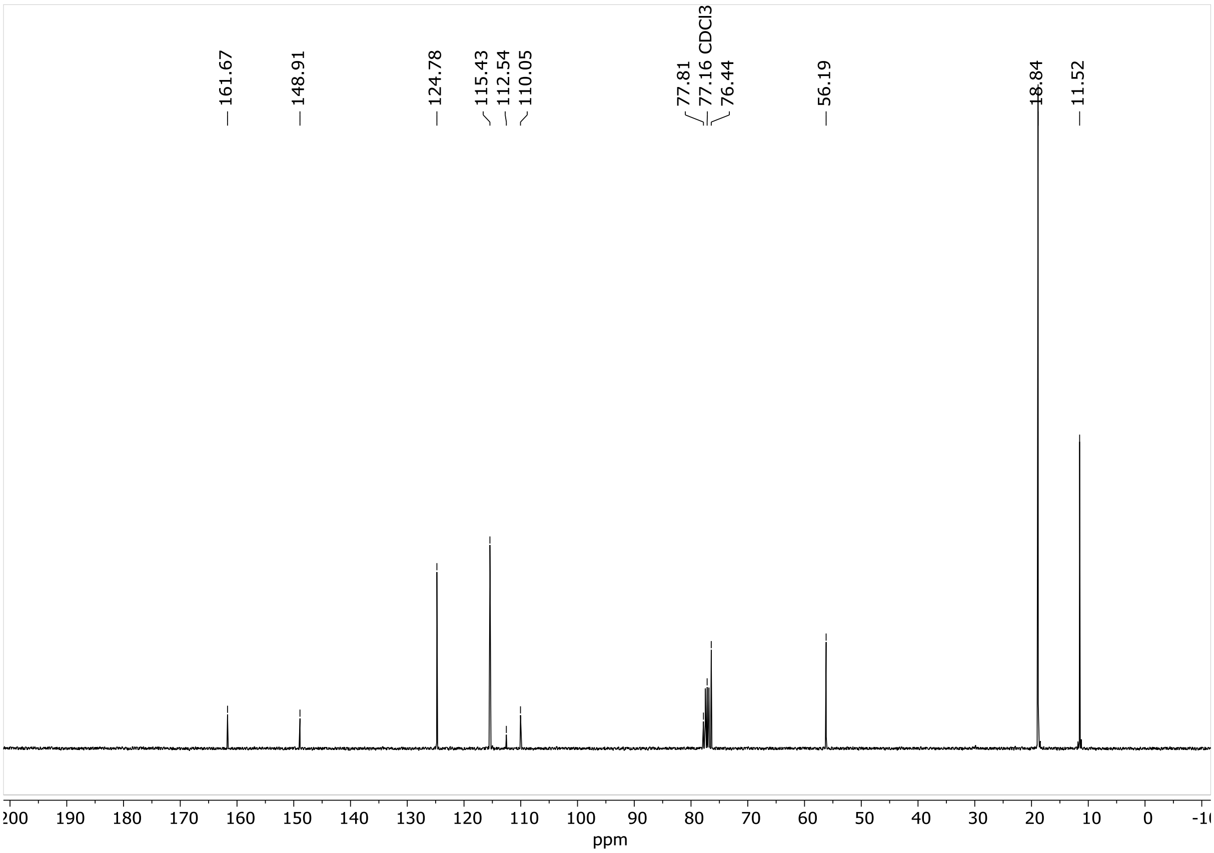

^1H NMR and ^{13}C NMR spectrum of compound **7a**

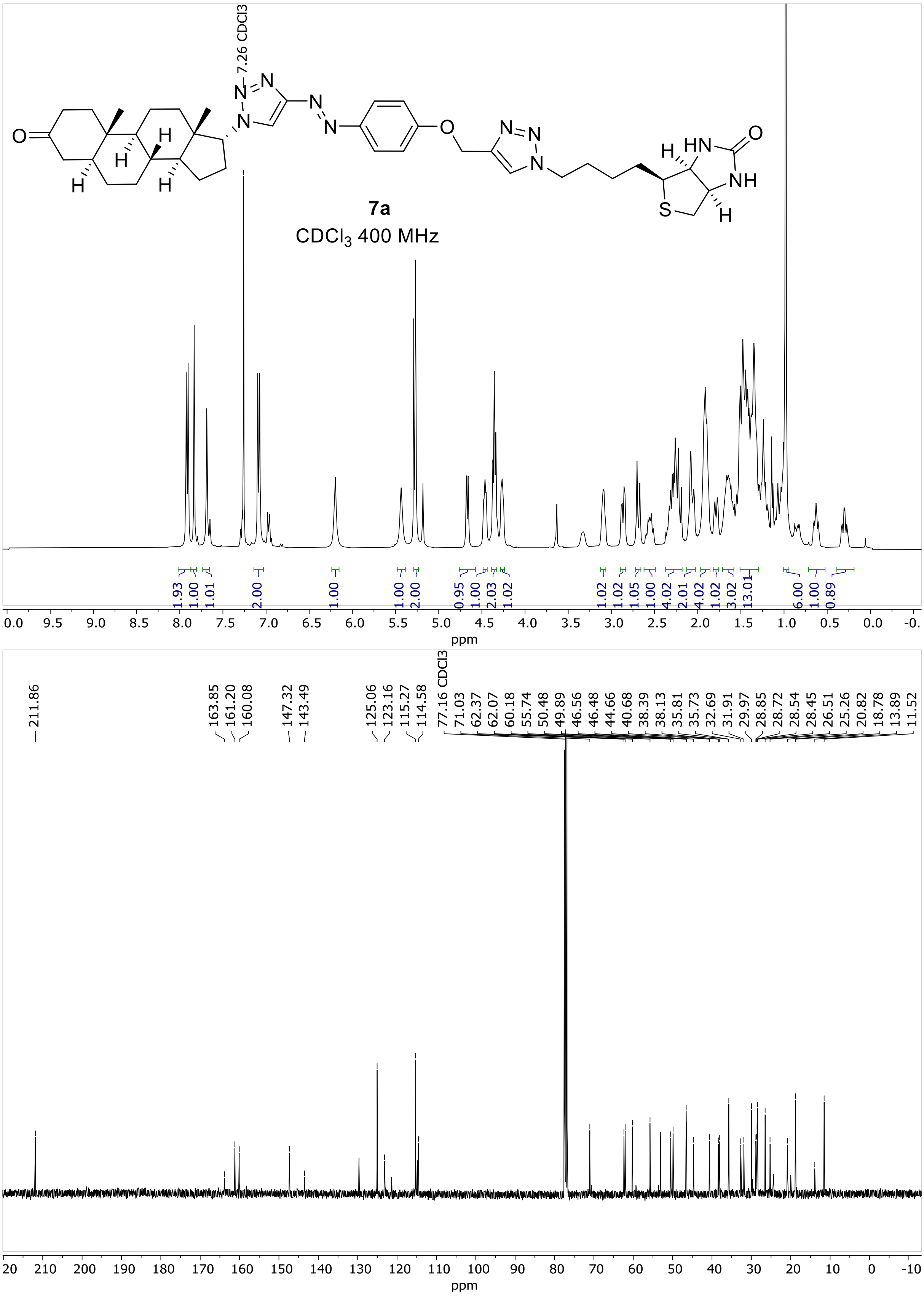

^{1}H NMR and ^{13}C NMR spectrum of compound **SI-7-1**

Note: $CDCl_3$ contained TMS as internal reference (Peak at 0.00 ppm).

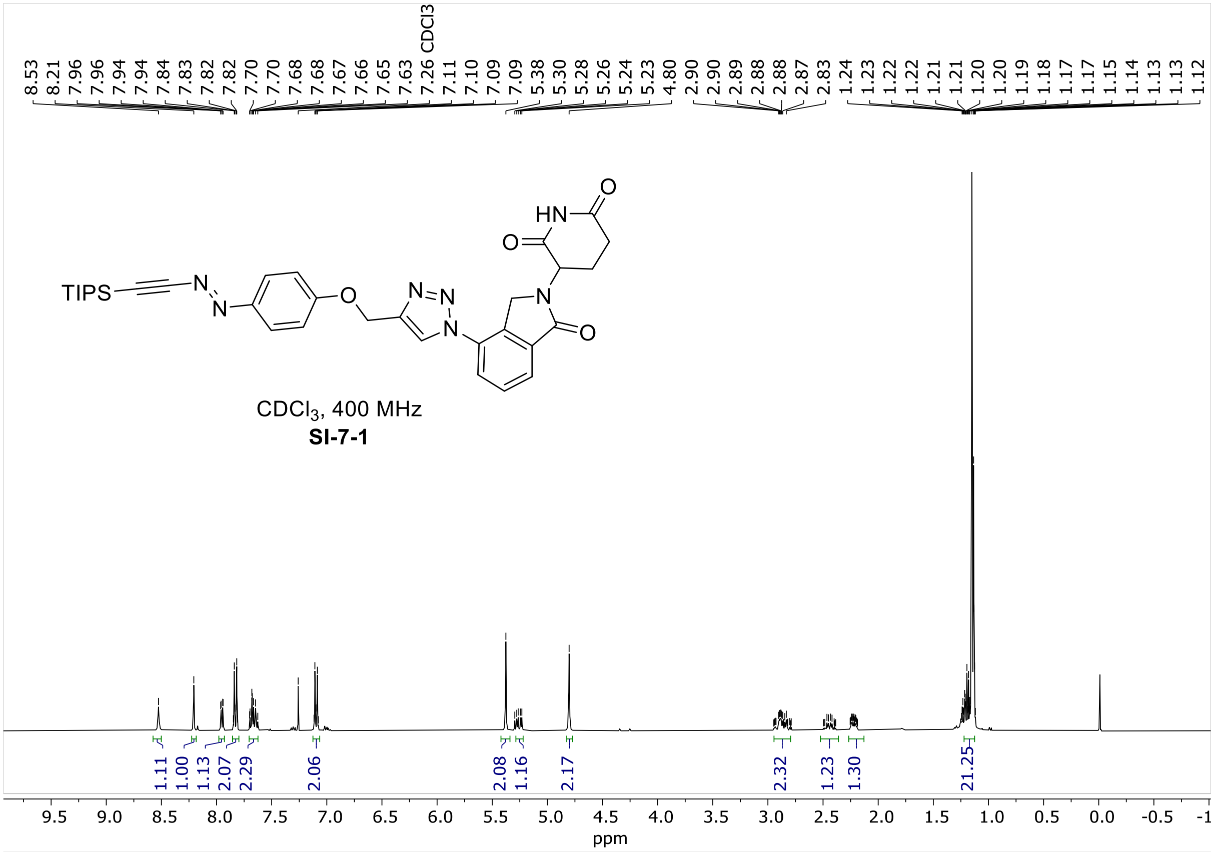

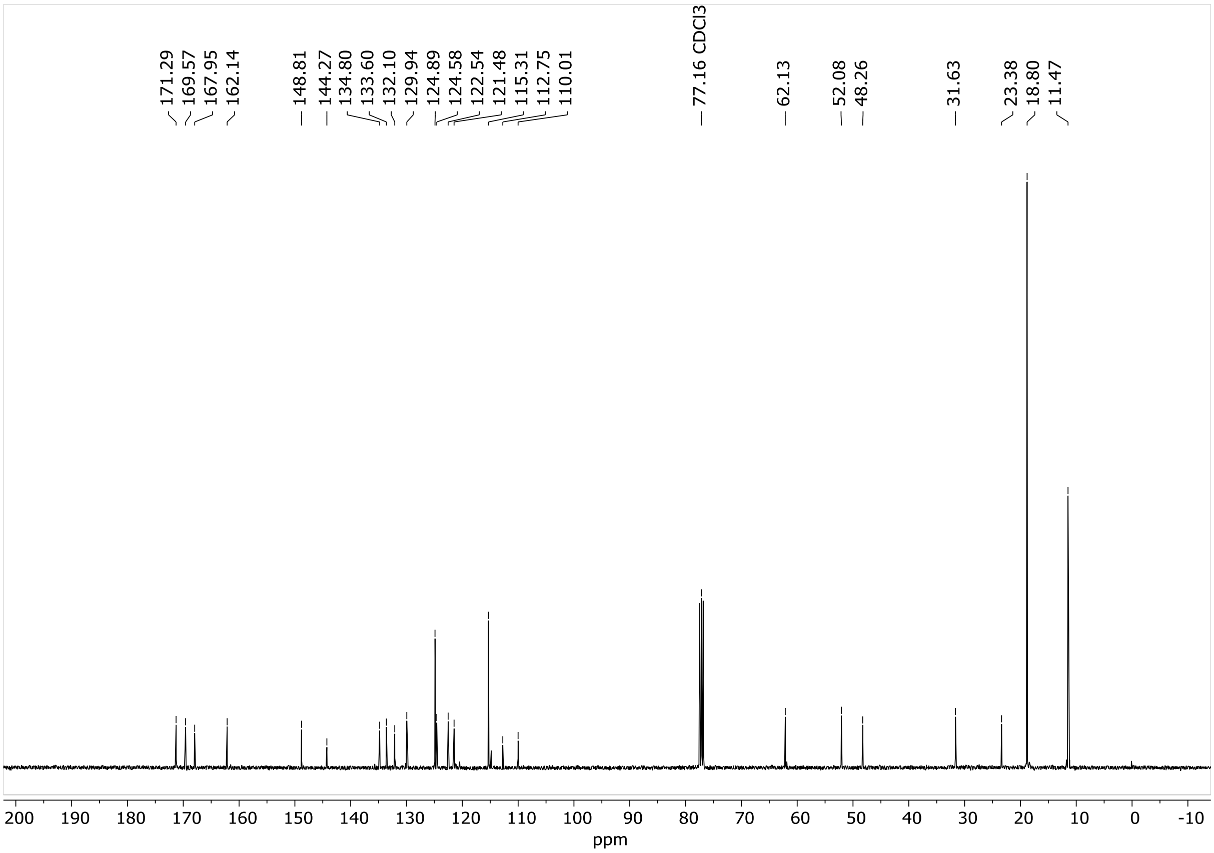

^{1}H NMR and ^{13}C NMR spectrum of compound **7b**

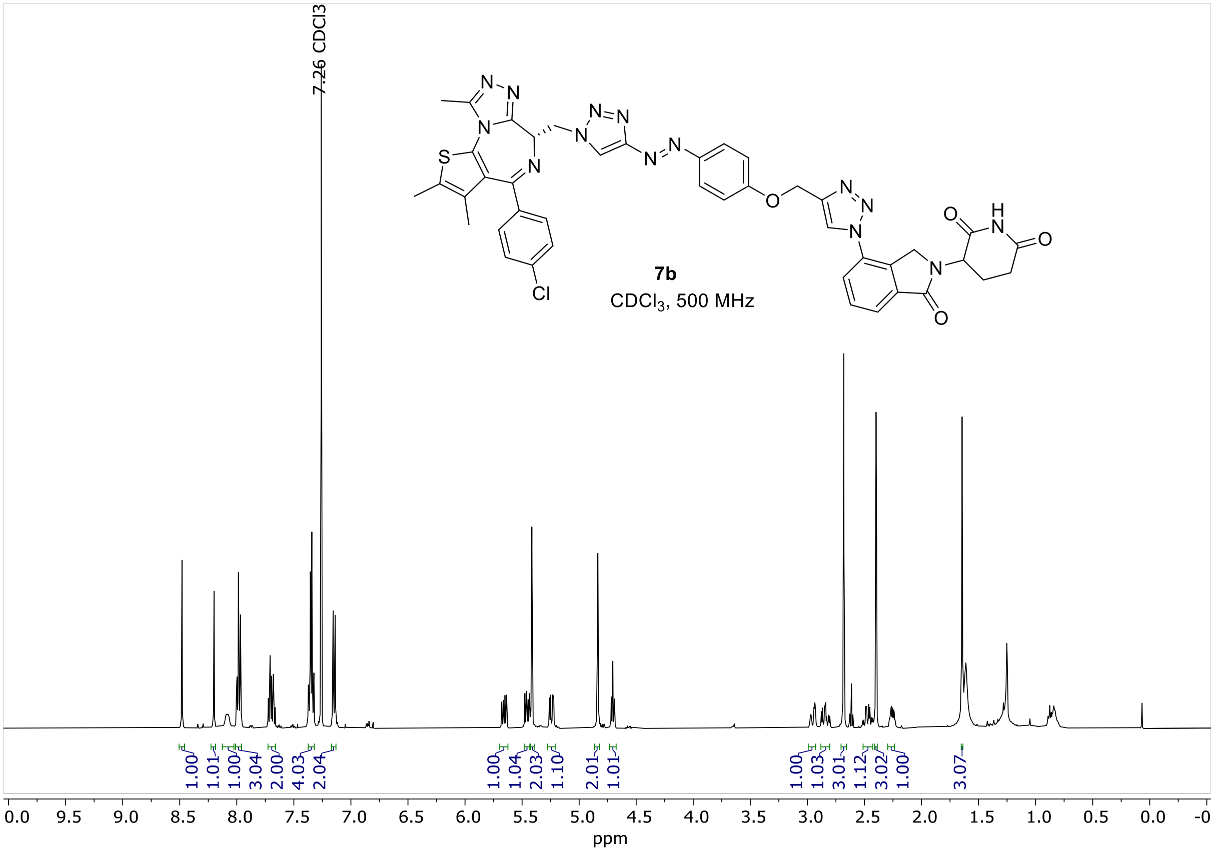

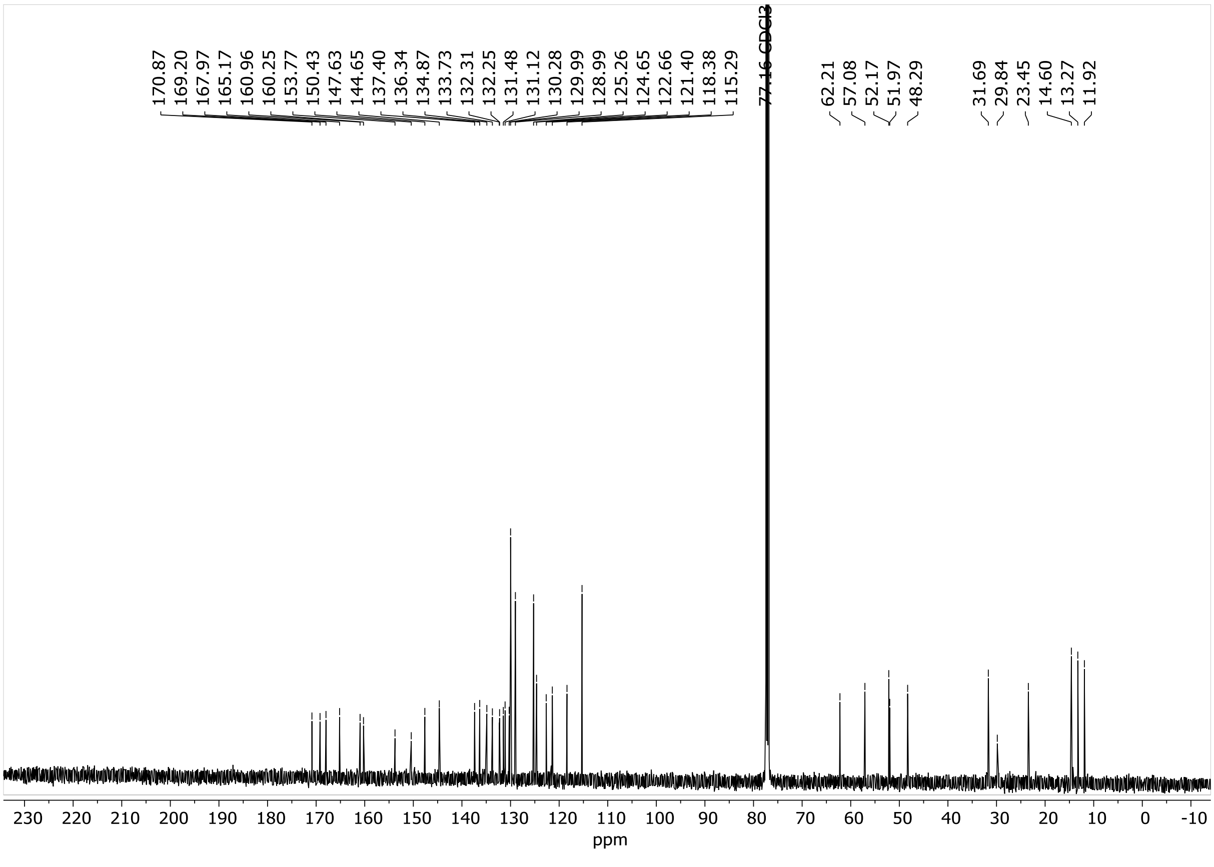

^{1}H NMR and ^{13}C NMR spectrum of compound **7c**

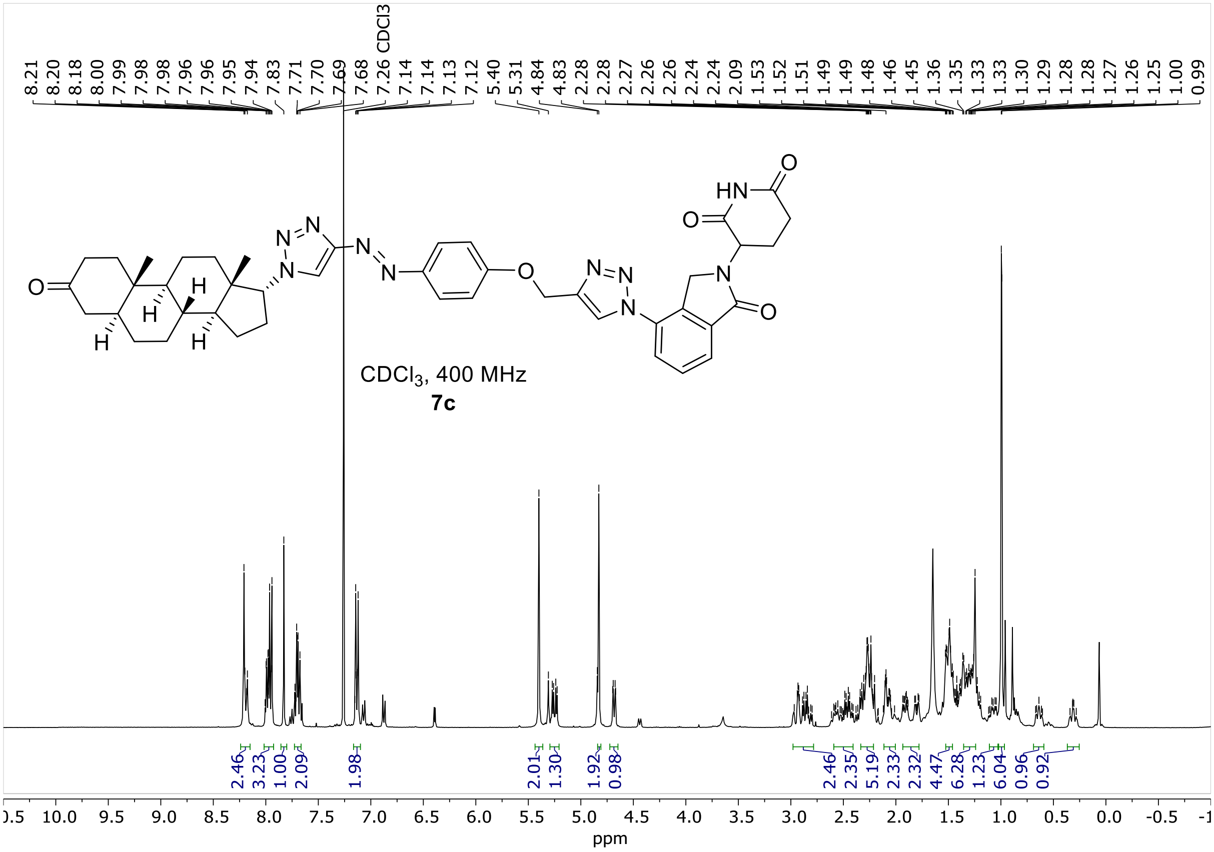

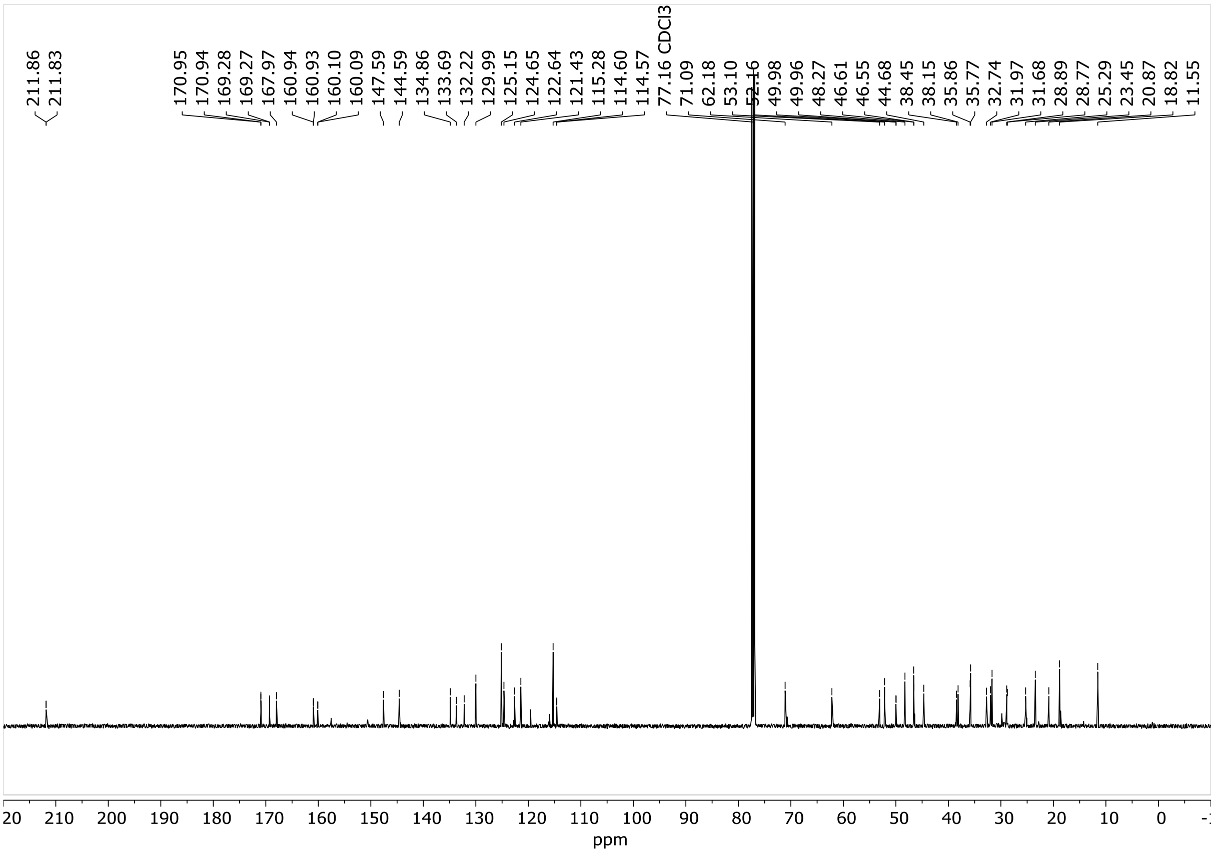

Determination of Isosbestic Points in HPLC Eluent for Photostationary State Measurement Extracted UV-VIS from HPLC-PDA Detector

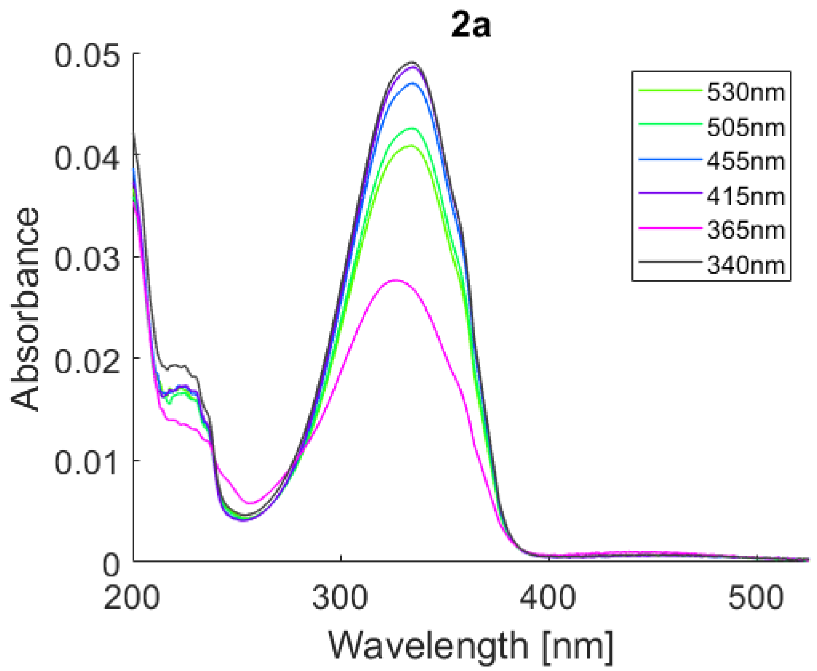

UV-Vis spectra of **2a** in MeCN/H_2O (55:45), recorded after irradiation at the stated wavelengths to reach the photostationary state; isosbestic wavelength estimated to be 280 nm.

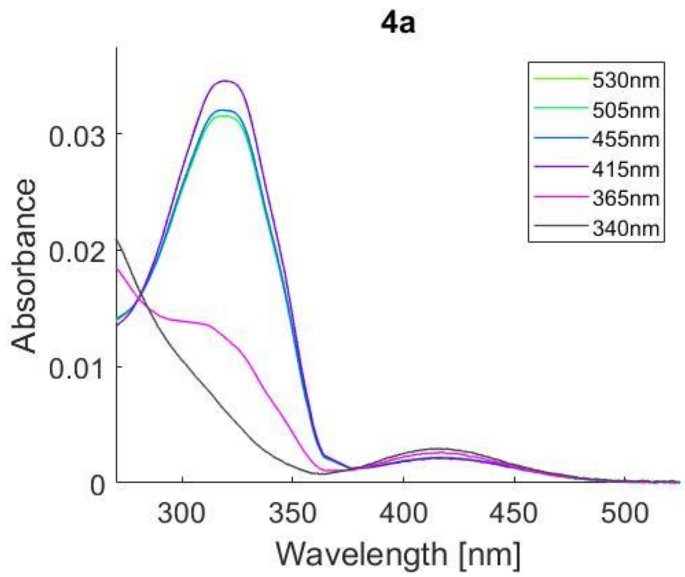

UV-Vis spectra of **4a** in MeCN/H_2O (6:4), recorded after irradiation at the stated wavelengths to reach the photostationary state; isosbestic wavelength estimated to be 281 nm.

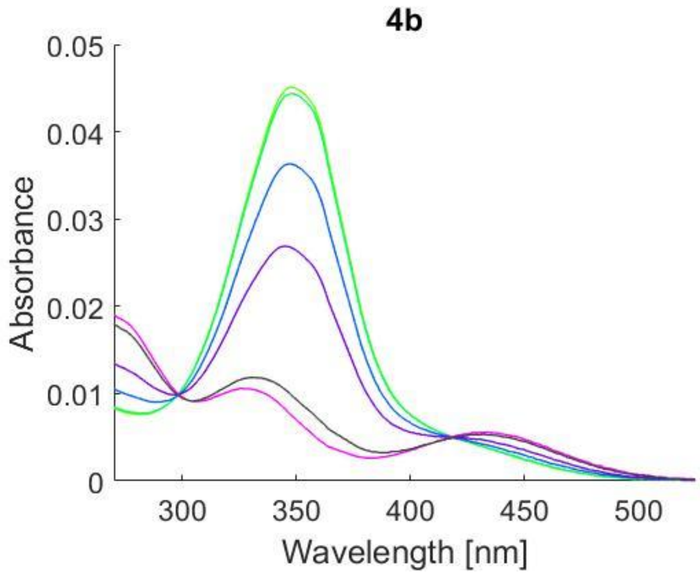

UV-Vis spectra of **4b** in MeCN/H_2O (6:4), recorded after irradiation at the stated wavelengths to reach the photostationary state; isosbestic wavelength estimated to be 298 nm.

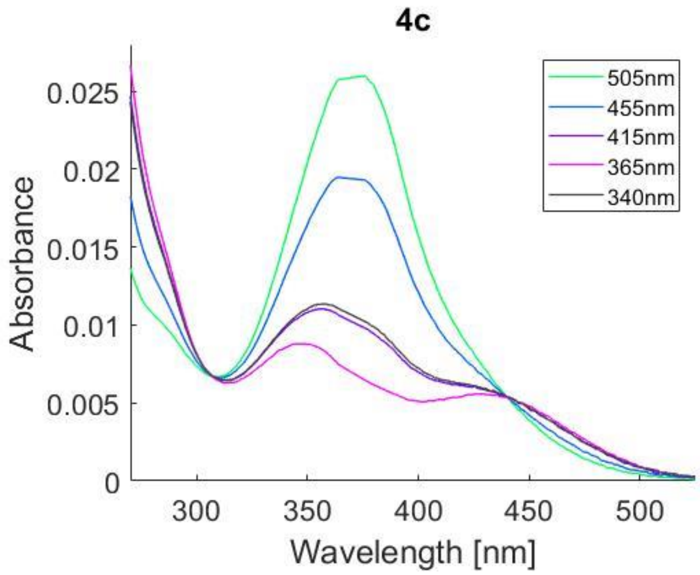

UV-Vis spectra of **4c** in MeCN/H_2O (6:4), recorded after irradiation at the stated wavelengths to reach the photostationary state; isosbestic wavelength estimated to be 308 nm.

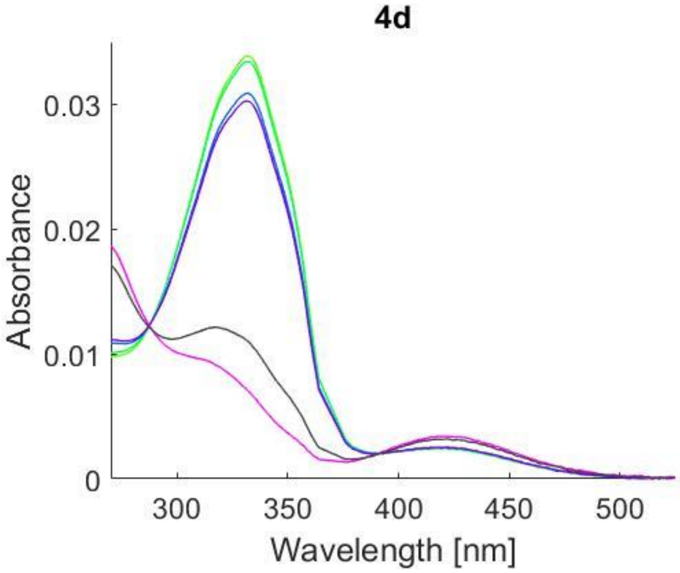

UV-Vis spectra of **4d** in MeCN/H_2O (6:4), recorded after irradiation at the stated wavelengths to reach the photostationary state; isosbestic wavelength estimated to be 287 nm.

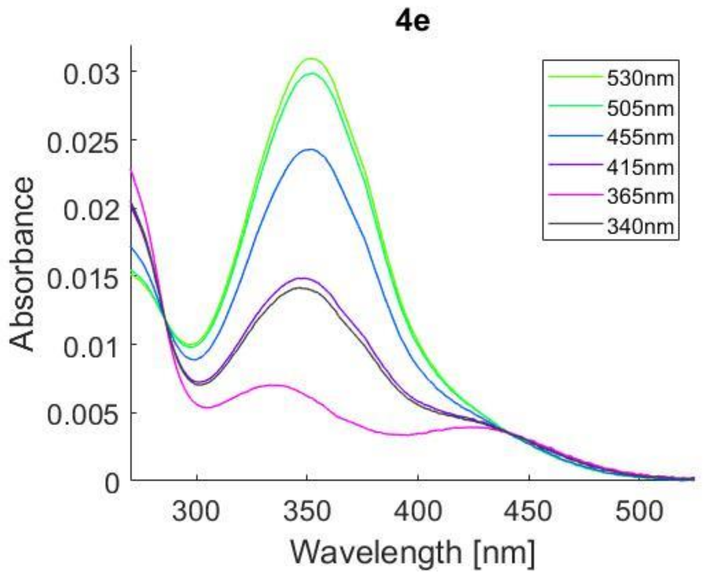

UV-Vis spectra of **4e** in MeCN/H_2O (6:4), recorded after irradiation at the stated wavelengths to reach the photostationary state; isosbestic wavelength estimated to be 278 nm.

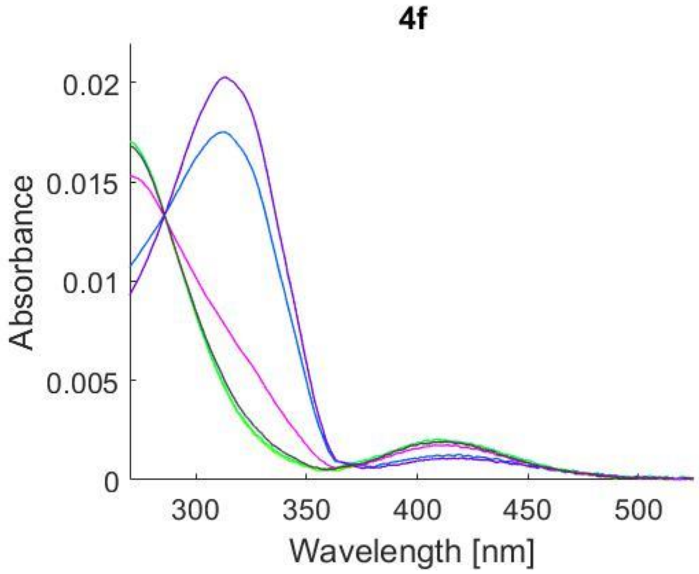

UV-Vis spectra of **4f** in MeCN/H_2O (6:4), recorded after irradiation at the stated wavelengths to reach the photostationary state; isosbestic wavelength estimated to be 286 nm.

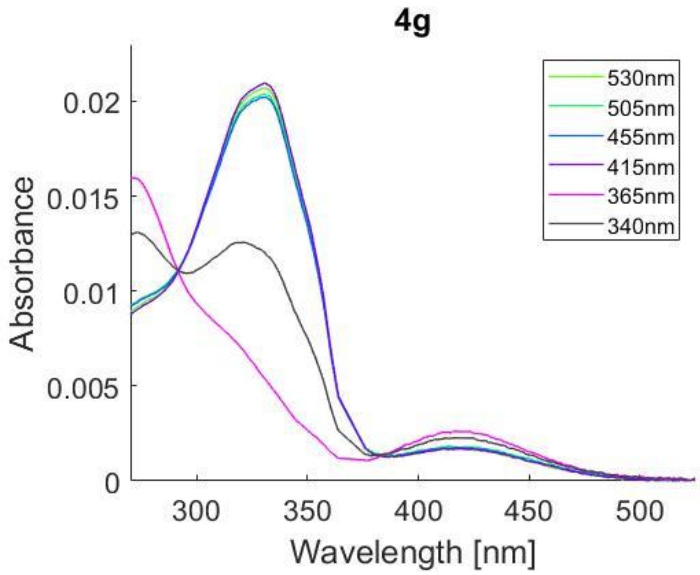

UV-Vis spectra of **4g** in MeCN/H_2O (6:4), recorded after irradiation at the stated wavelengths to reach the photostationary state; isosbestic wavelength estimated to be 292 nm.

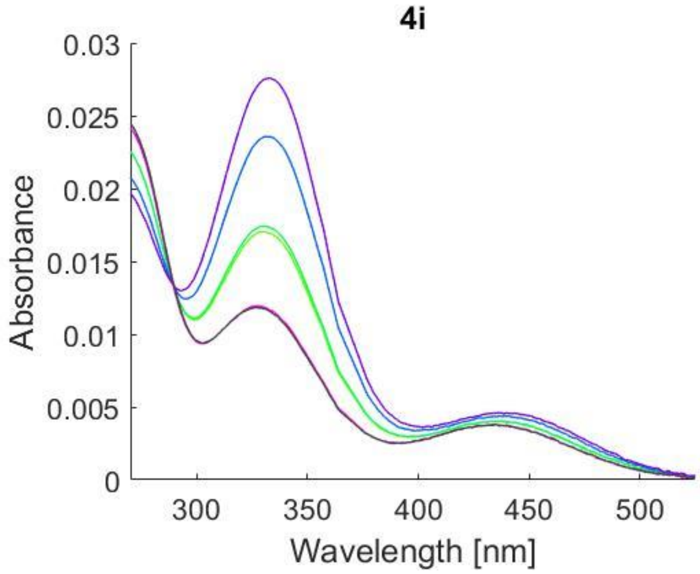

UV-Vis spectra of **4i** in MeCN/H_2O (6:4), recorded after irradiation at the stated wavelengths to reach the photostationary state; isosbestic wavelength estimated to be 289 nm.

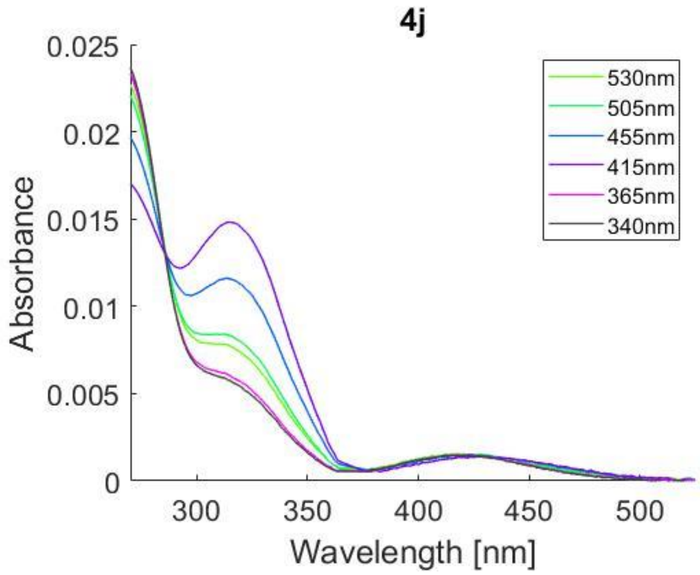

UV-Vis spectra of **4j** in MeCN/H_2O (6:4), recorded after irradiation at the stated wavelengths to reach the photostationary state; isosbestic wavelength estimated to be 286 nm.

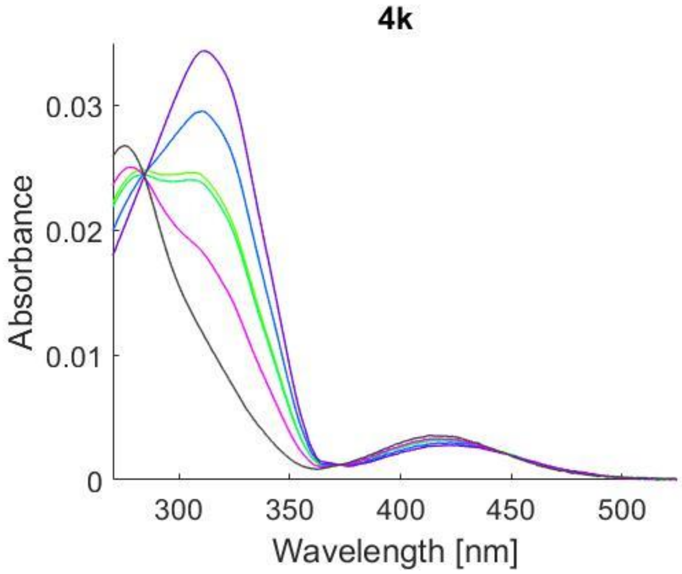

UV-Vis spectra of **4k** in MeCN/H_2O (6:4), recorded after irradiation at the stated wavelengths to reach the photostationary state; isosbestic wavelength estimated to be 284 nm.

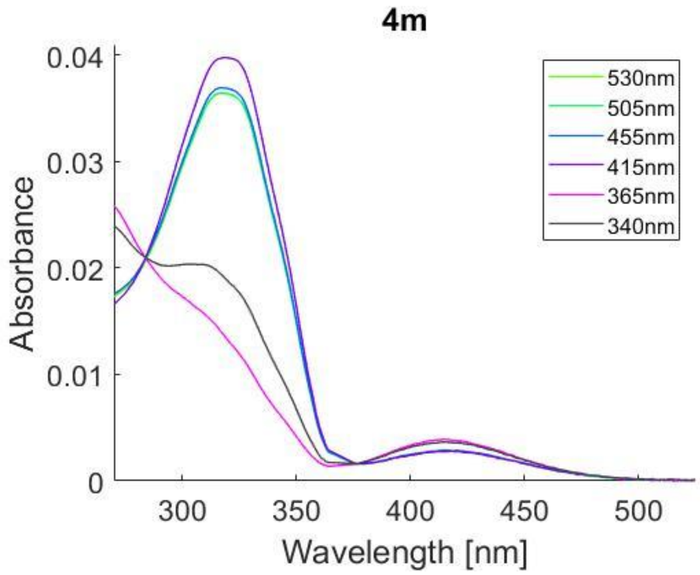

UV-Vis spectra of **4m** in MeCN/H_2O (6:4), recorded after irradiation at the stated wavelengths to reach the photostationary state; isosbestic wavelength estimated to be 284 nm.

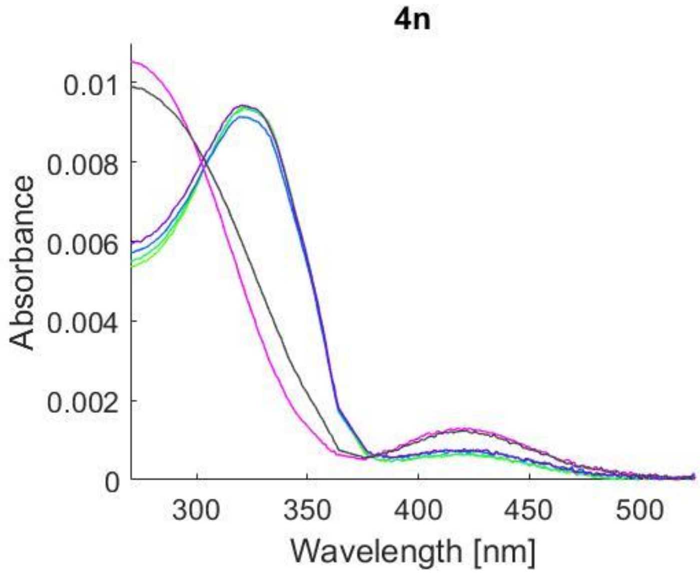

UV-Vis spectra of **4n** in MeCN/H_2O (6:4), recorded after irradiation at the stated wavelengths to reach the photostationary state; isosbestic wavelength estimated to be 303 nm.

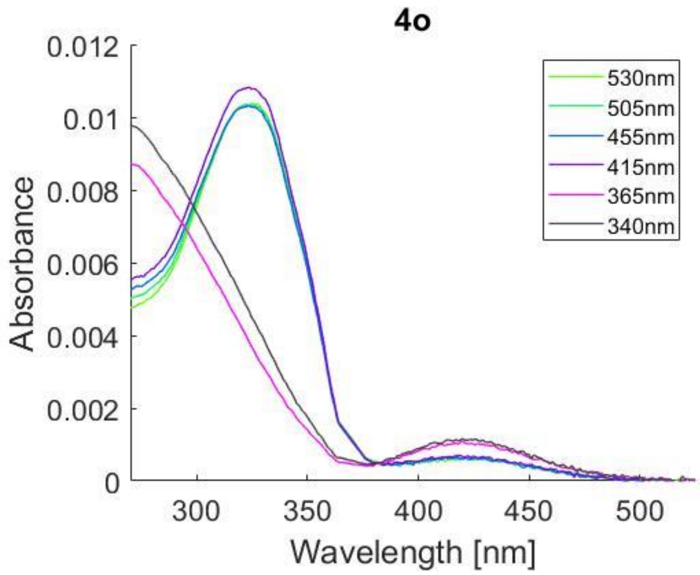

UV-Vis spectra of **4o** in MeCN/H_2O (6:4), recorded after irradiation at the stated wavelengths to reach the photostationary state; isosbestic wavelength estimated to be 296 nm.

Photostationary State Determination from HPLC Chromatograms at Isosbestic Wavelength

2a

340 nm

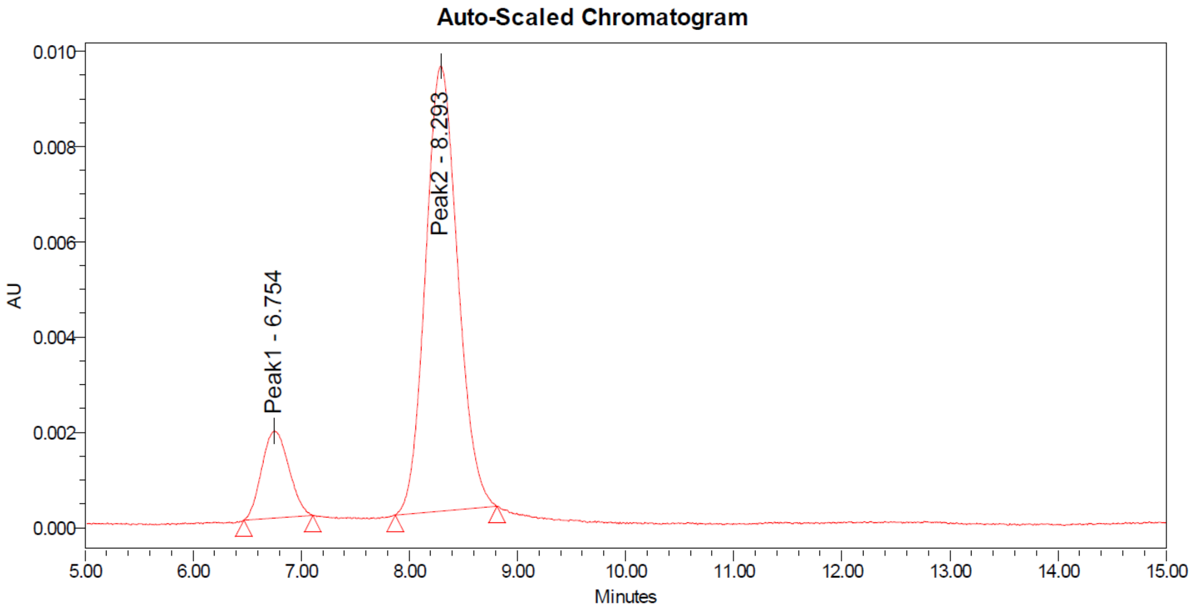

Peak Results

	Name	RT	Height	Area	% Area
1	Peak1	6.754	1836	31811	14.10
2	Peak2	8.293	9341	193859	85.90

365 nm

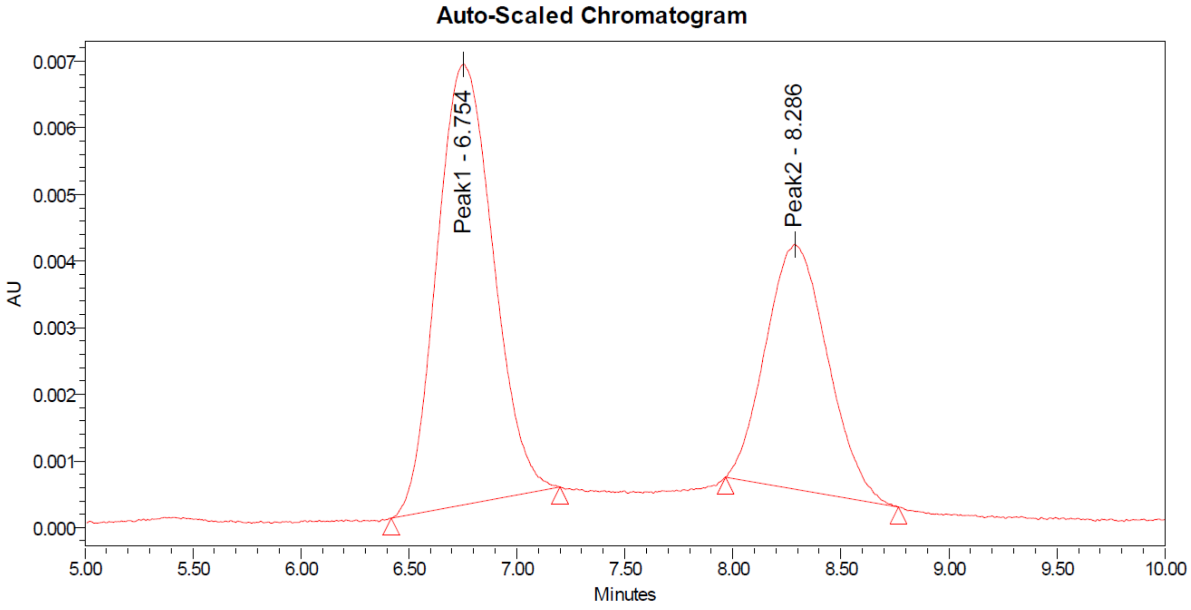

Peak Results

	Name	RT	Height	Area	% Area
1	Peak1	6.754	6611	120141	62.08
2	Peak2	8.286	3678	73384	37.92

415 nm

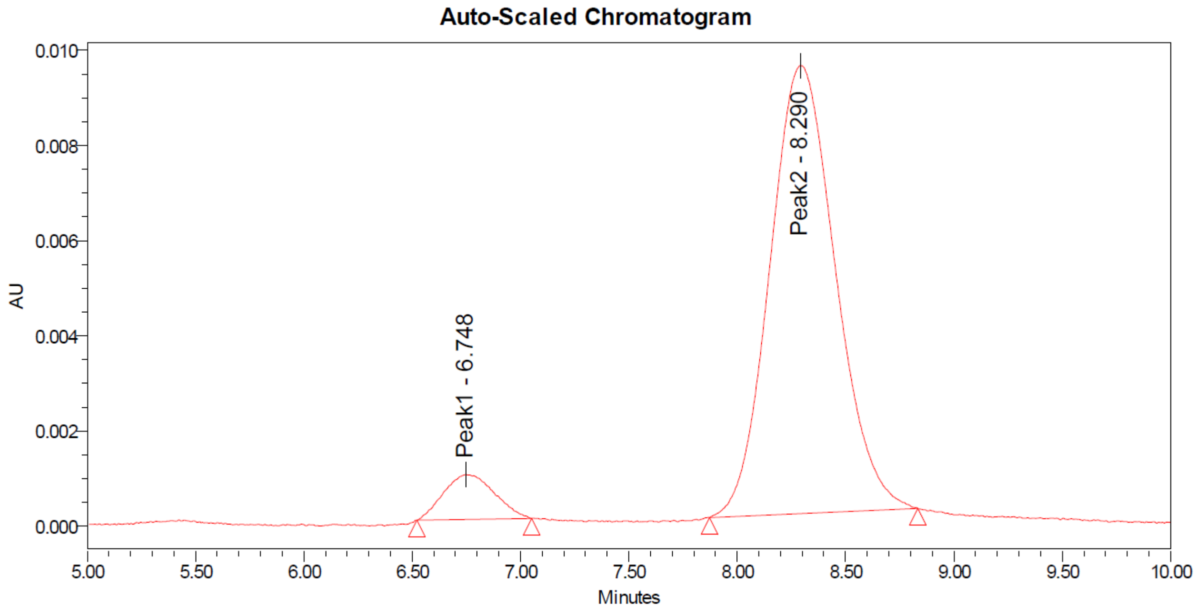

Peak Results

	Name	RT	Height	Area	% Area
1	Peak1	6.748	944	15431	7.23
2	Peak2	8.290	9412	197893	92.77

455 nm

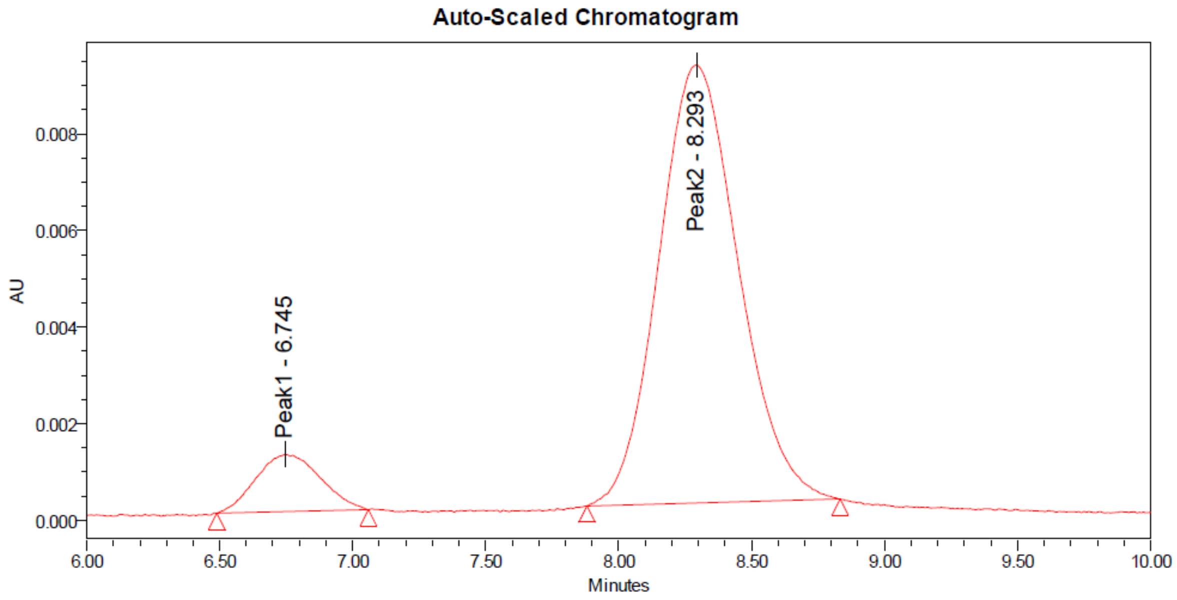

Peak Results

	Name	RT	Height	Area	% Area
1	Peak1	6.745	1184	19910	9.54
2	Peak2	8.293	9065	188886	90.46

505 nm

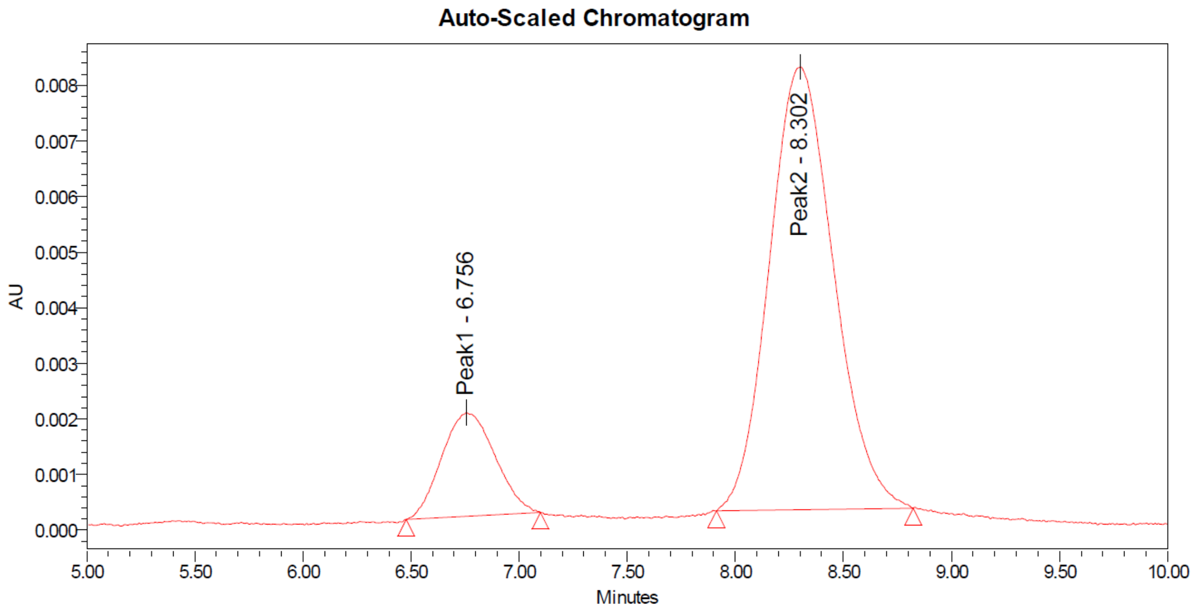

Peak Results

	Name	RT	Height	Area	% Area
1	Peak1	6.756	1866	31851	16.19
2	Peak2	8.302	7964	164928	83.81

530 nm

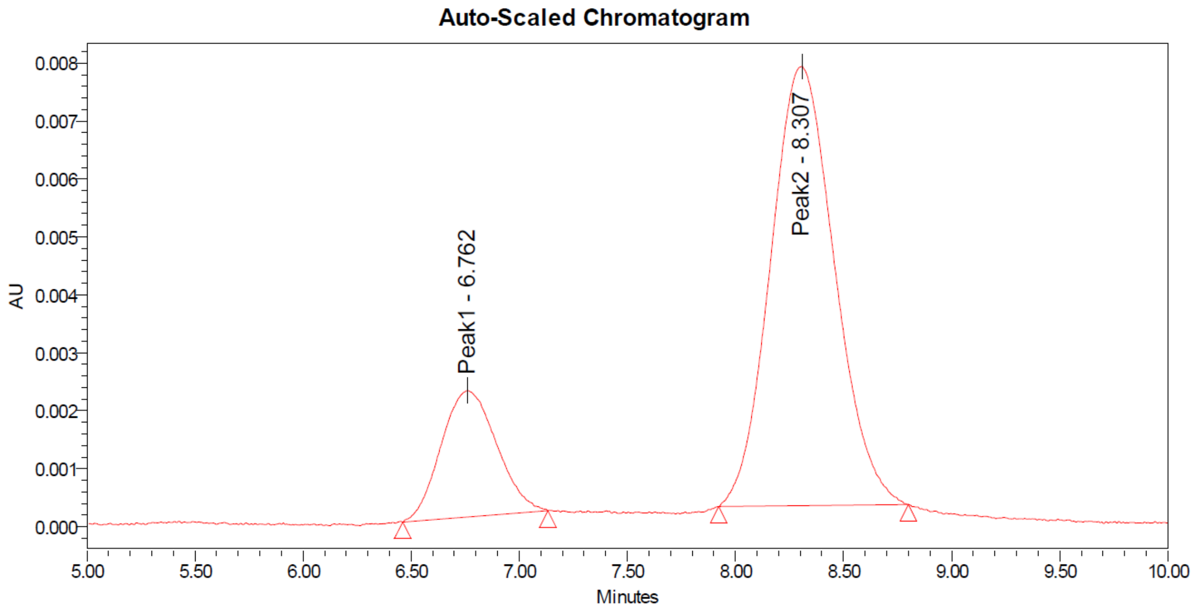

Peak Results

	Name	RT	Height	Area	% Area
1	Peak1	6.762	2180	38859	19.95
2	Peak2	8.307	7581	155919	80.05

4a

340 nm

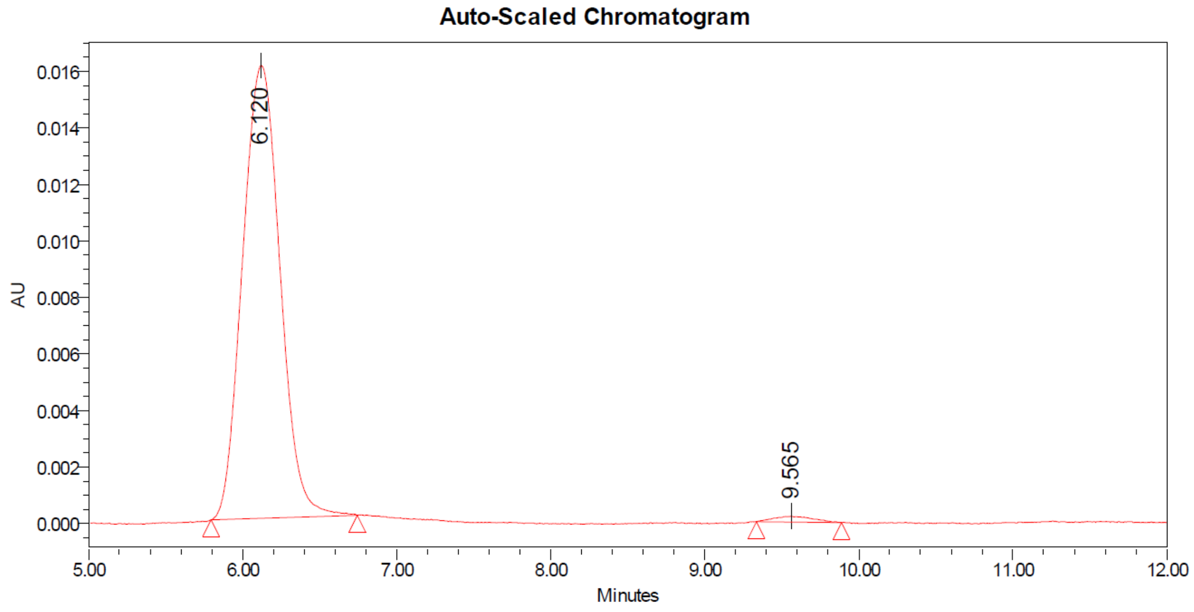

Peak Results

	Name	RT	Height	Area	% Area
1		6.120	16023	271484	98.62
2		9.565	210	3795	1.38

365 nm

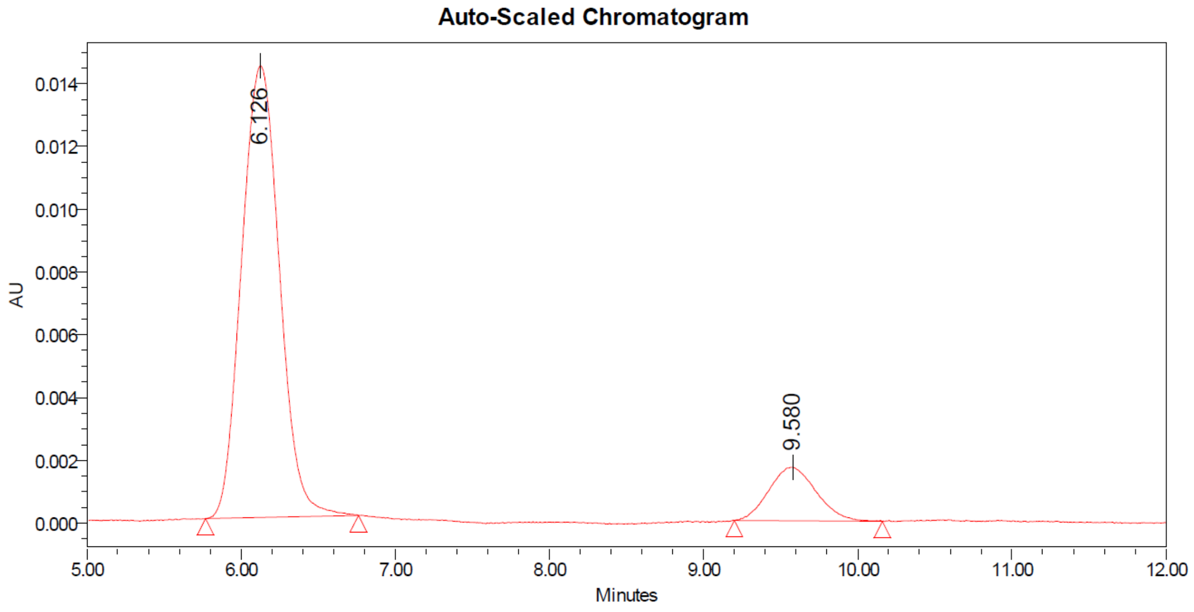

Peak Results

	Name	RT	Height	Area	% Area
1		6.126	14391	245855	87.11
2		9.580	1702	36378	12.89

415 nm

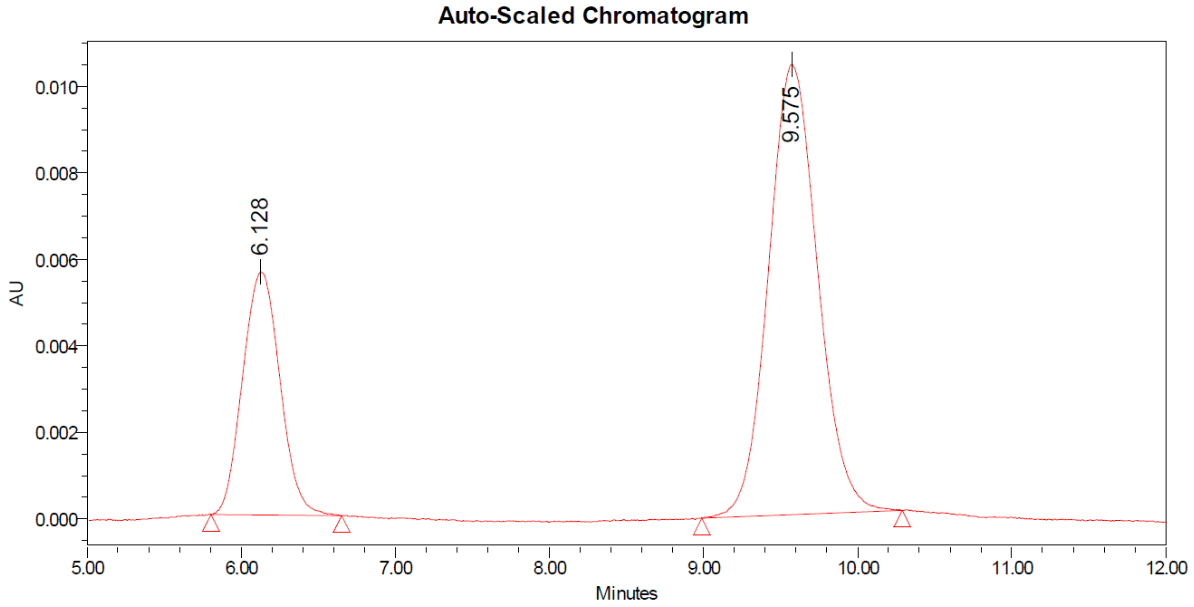

Peak Results

	Name	RT	Height	Area	% Area
1		6.128	5626	94799	29.09
2		9.575	10418	231107	70.91

455 nm

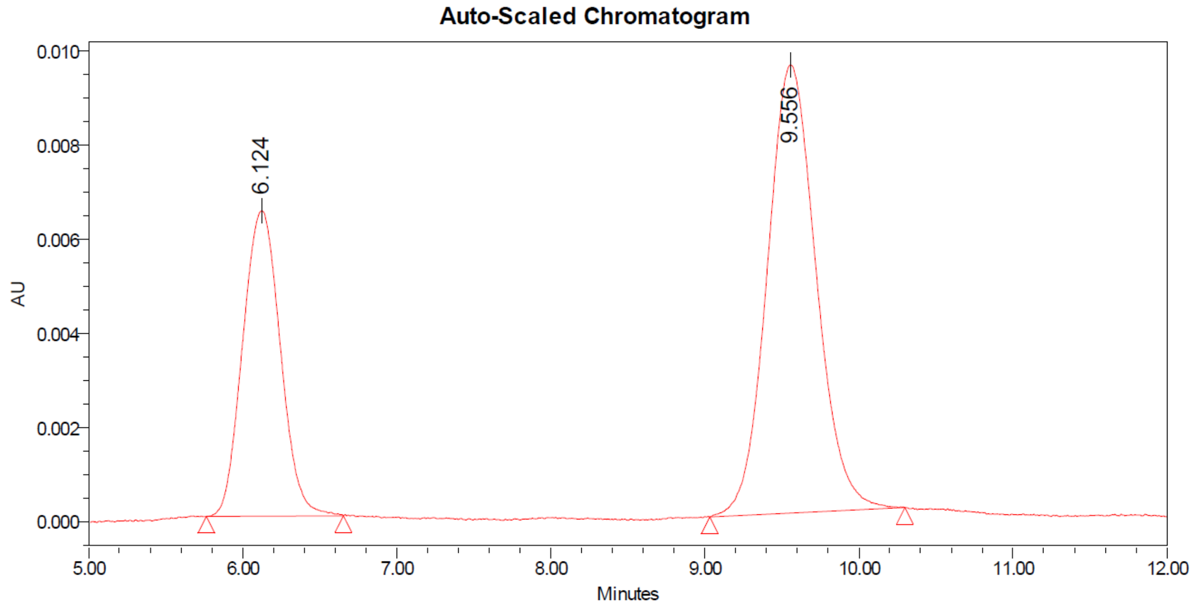

Peak Results

	Name	RT	Height	Area	% Area
1		6.124	6491	110254	34.39
2		9.556	9528	210358	65.61

505 nm

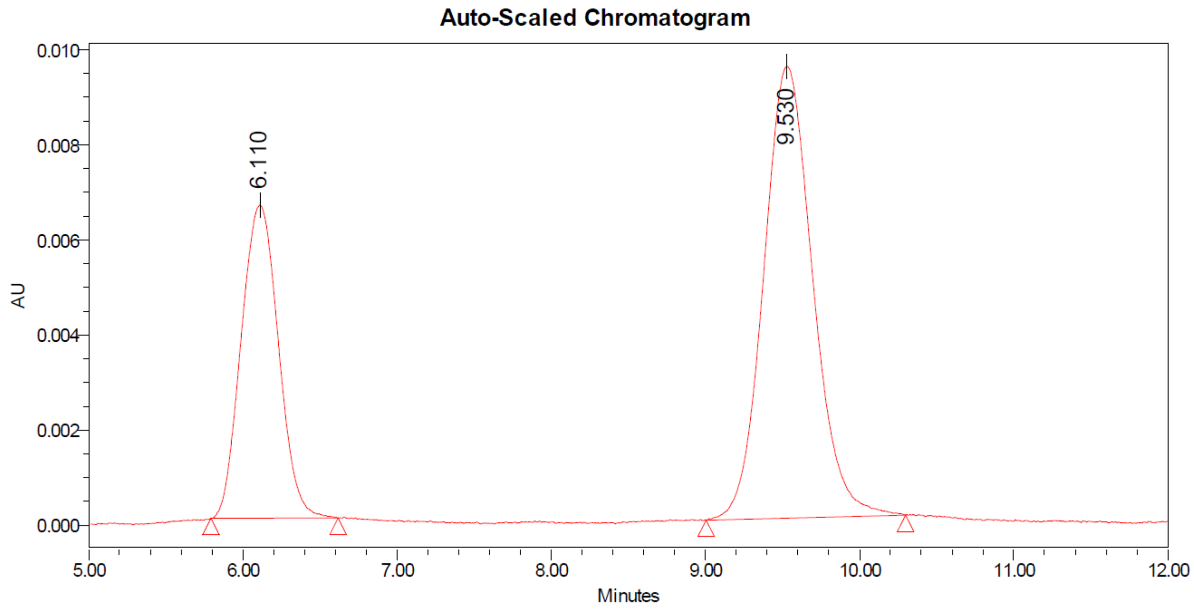

Peak Results

	Name	RT	Height	Area	% Area
1		6.110	6584	110544	34.51
2		9.530	9499	209738	65.49

530 nm

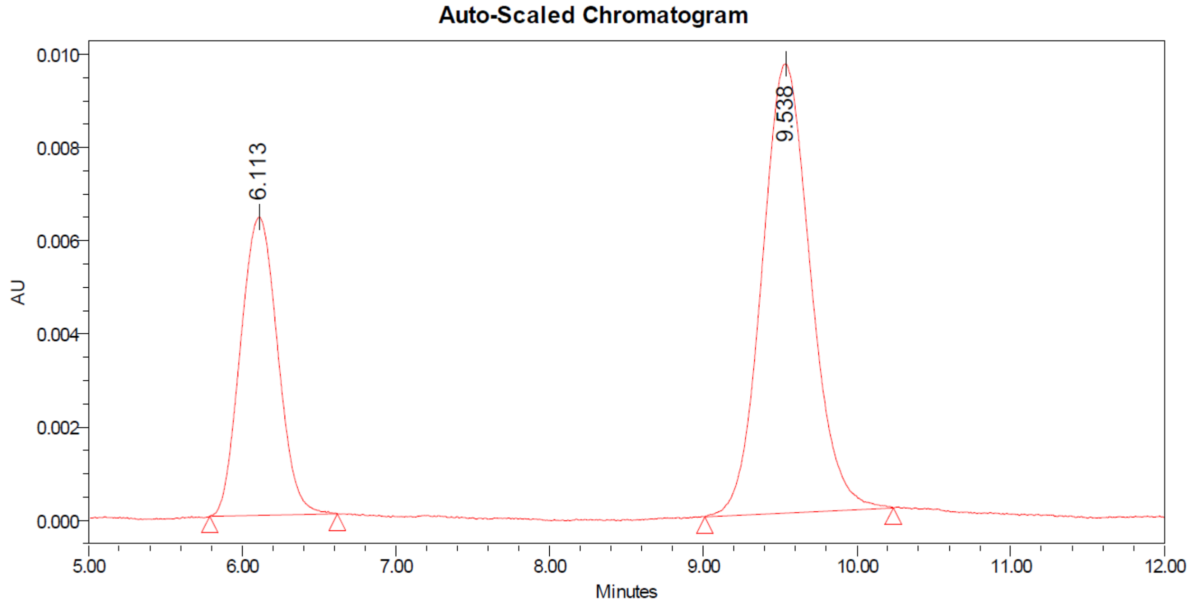

Peak Results

	Name	RT	Height	Area	% Area
1		6.113	6394	107317	33.64
2		9.538	9634	211693	66.36

4b

340 nm

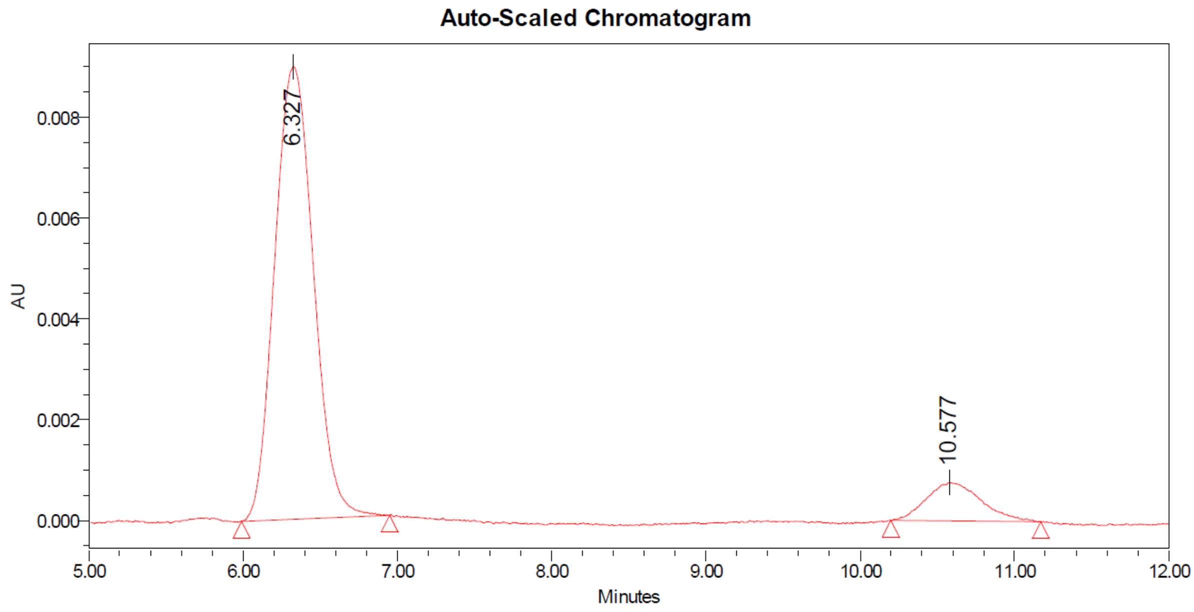

Peak Results

	Name	RT	Height	Area	% Area
1		6.327	8975	156867	89.33
2		10.577	762	18730	10.67

365 nm

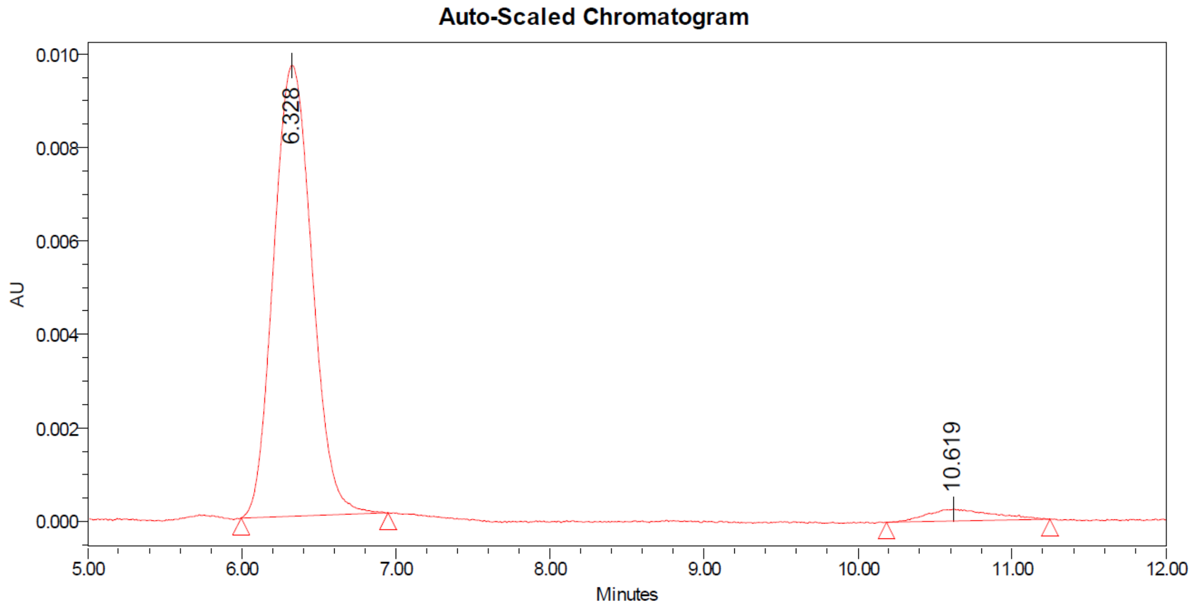

Peak Results

	Name	RT	Height	Area	% Area
1		6.328	9647	167575	95.78
2		10.619	253	7382	4.22

415 nm

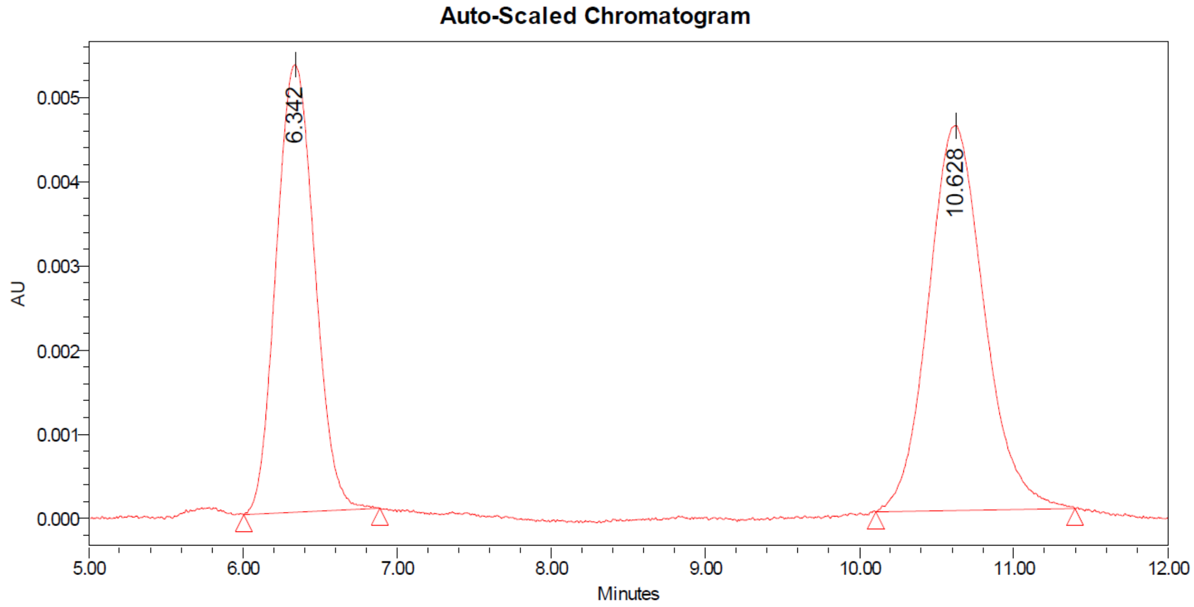

Peak Results

	Name	RT	Height	Area	% Area
1		6.342	5316	91975	44.45
2		10.628	4570	114927	55.55

455 nm

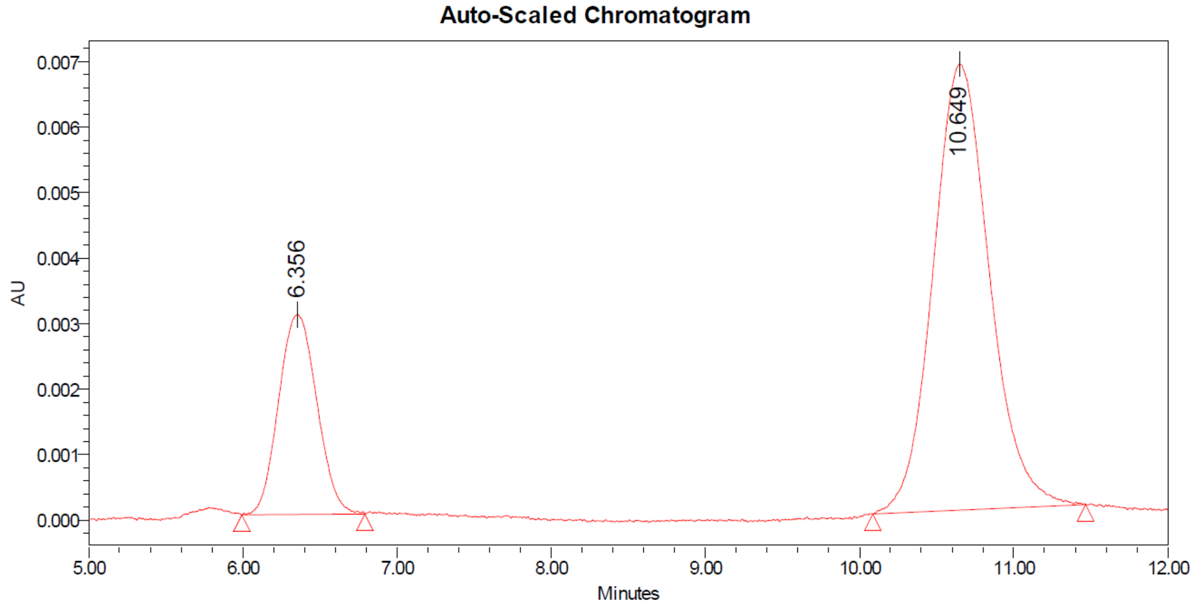

Peak Results

	Name	RT	Height	Area	% Area
1		6.356	3054	52676	23.33
2		10.649	6813	173063	76.67

505 nm

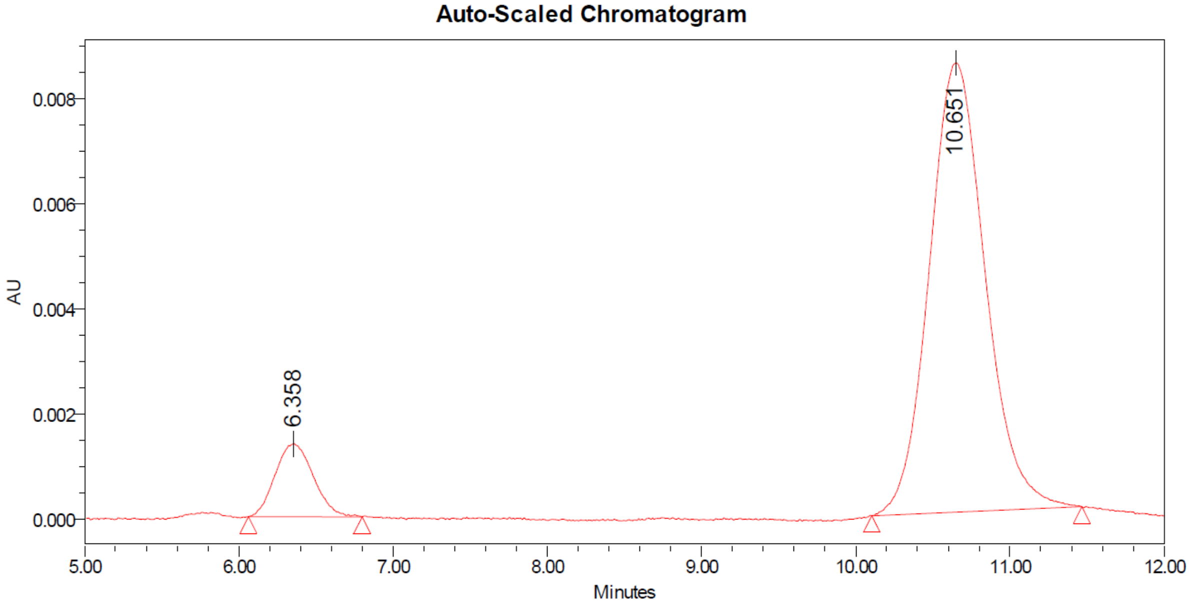

Peak Results

	Name	RT	Height	Area	% Area
1		6.358	1390	23921	9.98
2		10.651	8551	215731	90.02

530 nm

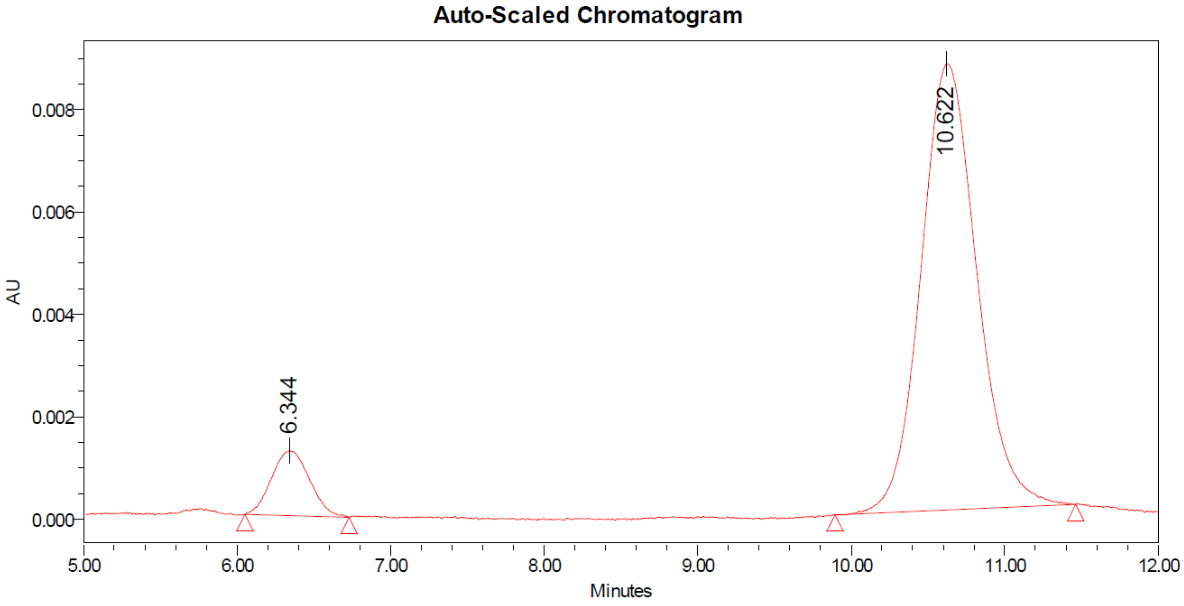

Peak Results

	Name	RT	Height	Area	% Area
1		6.344	1278	21890	9.03
2		10.622	8719	220655	90.97

4c

340 nm

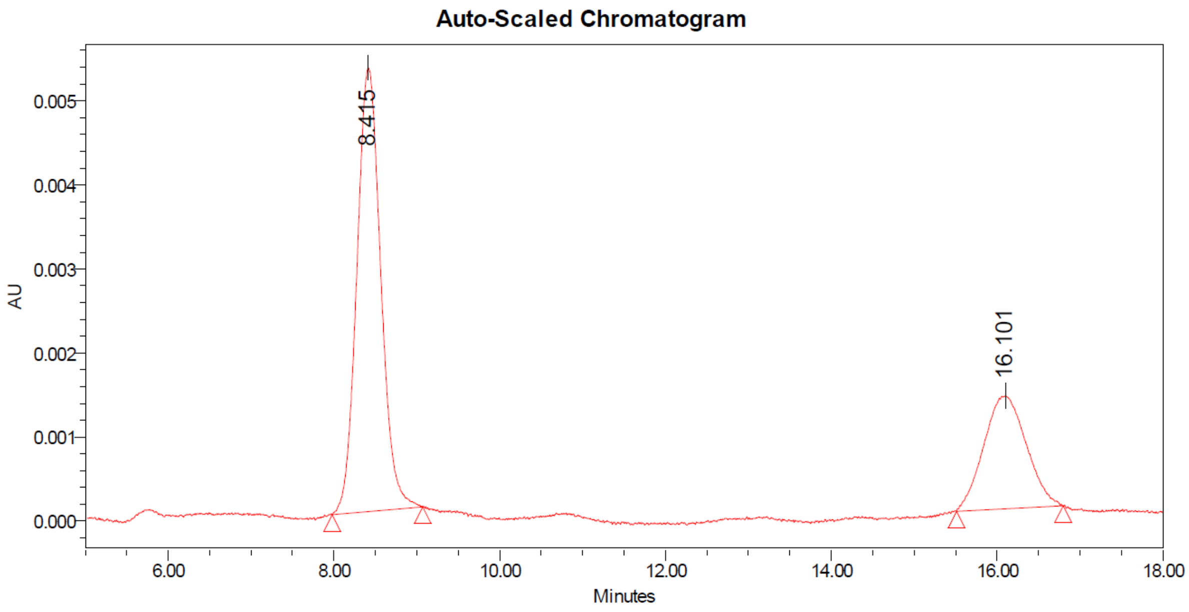

Peak Results

	Name	RT	Height	Area	% Area
1		8.415	5283	105854	68.88
2		16.101	1345	47832	31.12

365 nm

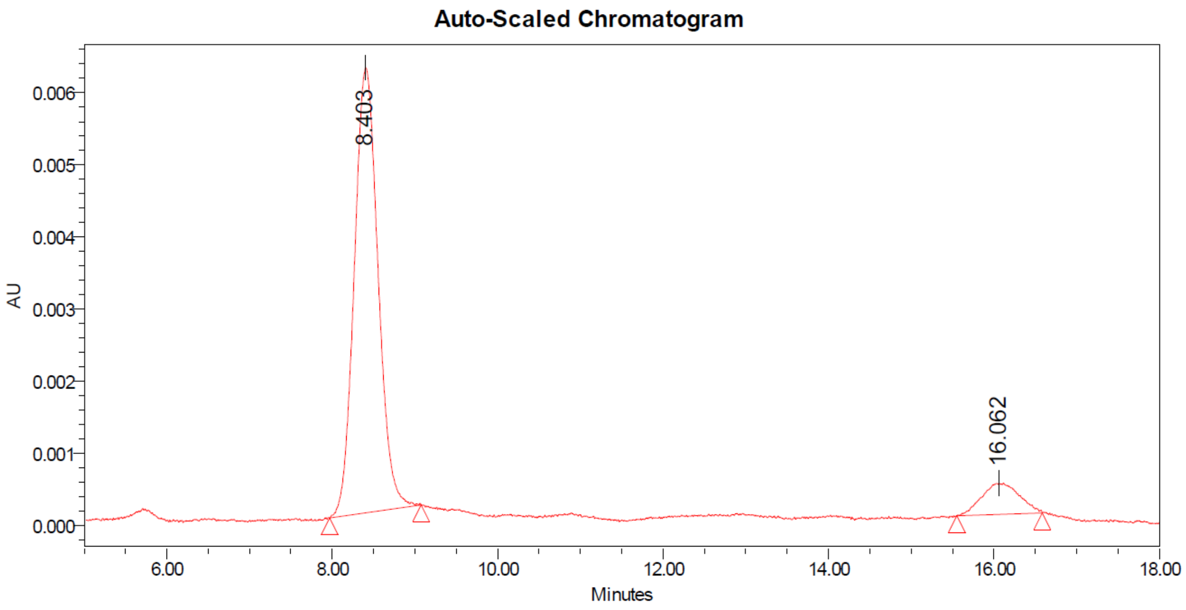

Peak Results

	Name	RT	Height	Area	% Area
1		8.403	6177	123267	89.98
2		16.062	433	13731	10.02

415 nm

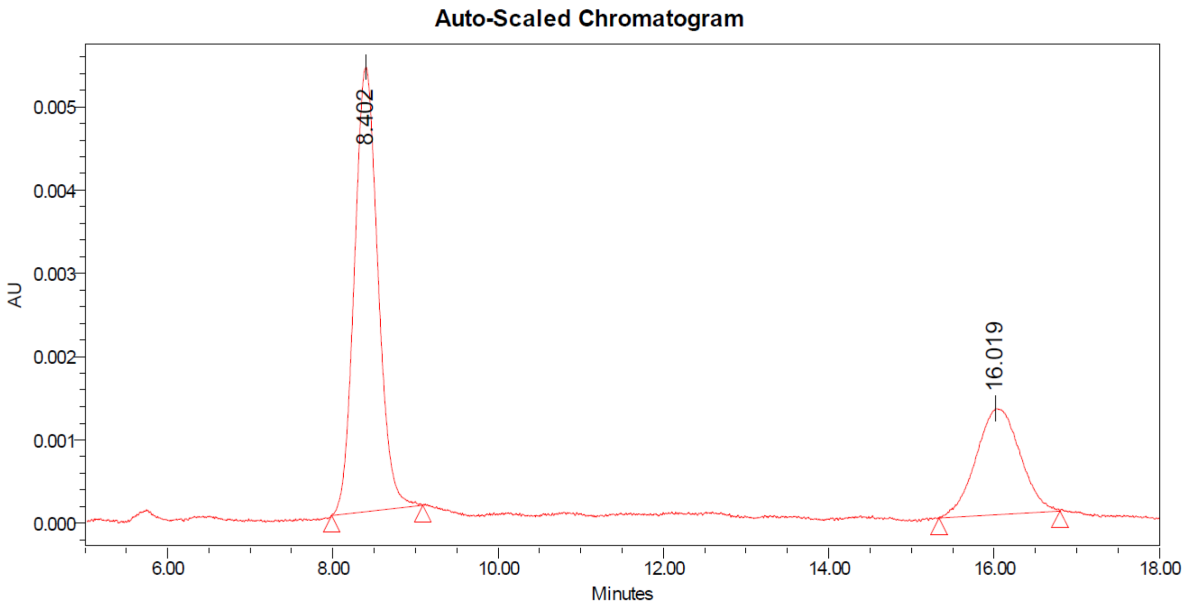

Peak Results

	Name	RT	Height	Area	% Area
1		8.402	5342	105917	69.17
2		16.019	1277	47219	30.83

455 nm

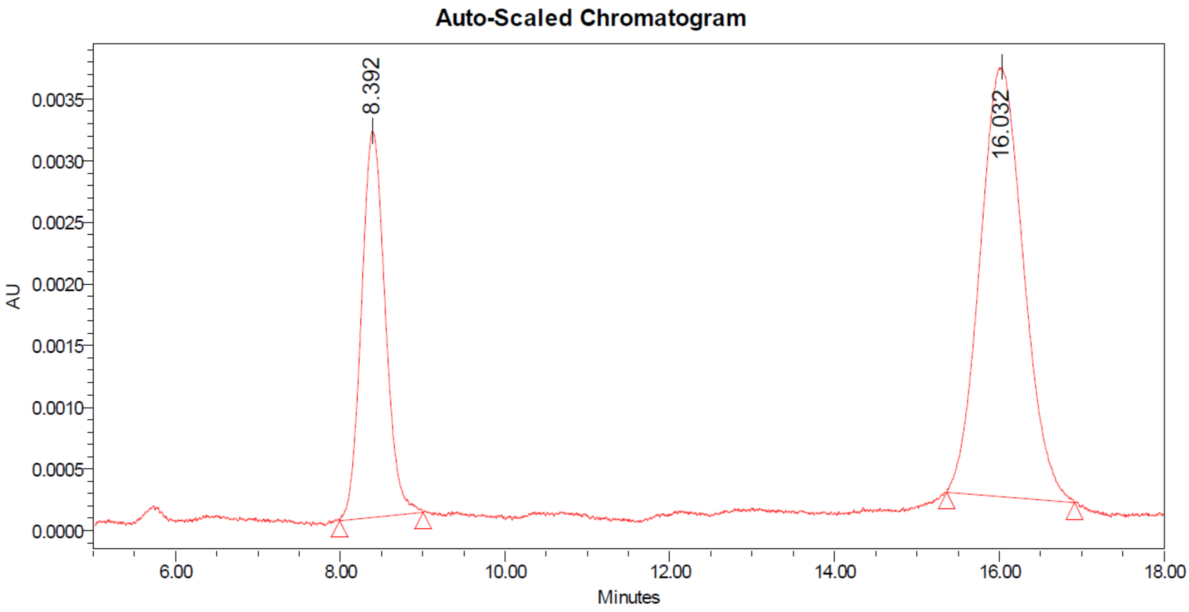

Peak Results

	Name	RT	Height	Area	% Area
1		8.392	3132	62671	32.92
2		16.032	3488	127697	67.08

505 nm

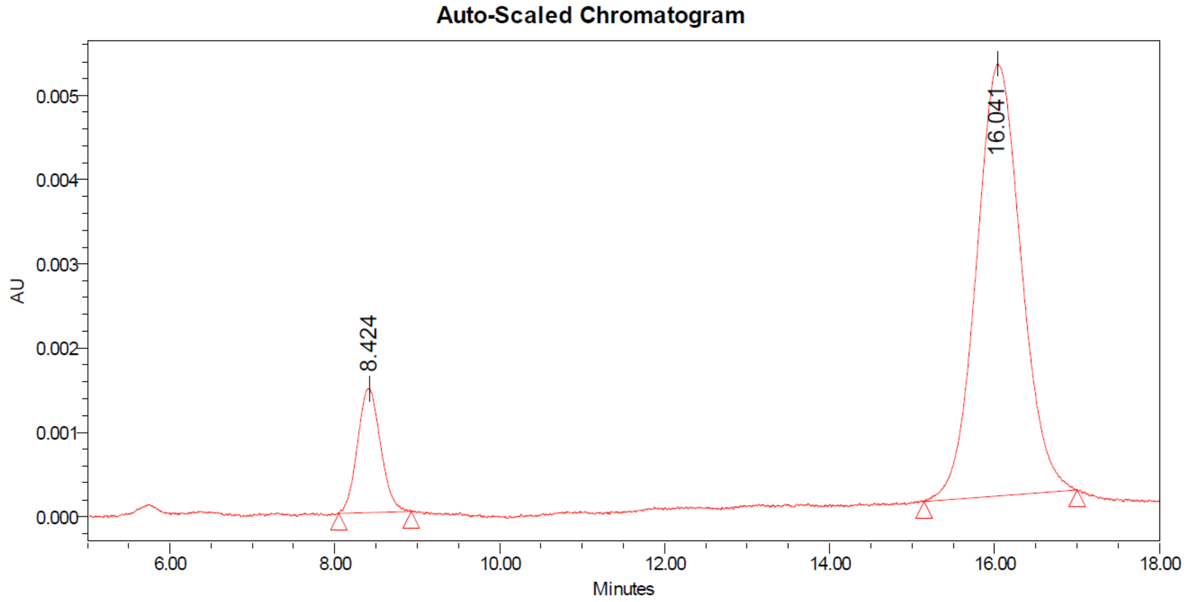

Peak Results

	Name	RT	Height	Area	% Area
1		8.424	1476	28814	12.96
2		16.041	5134	193584	87.04

530 nm

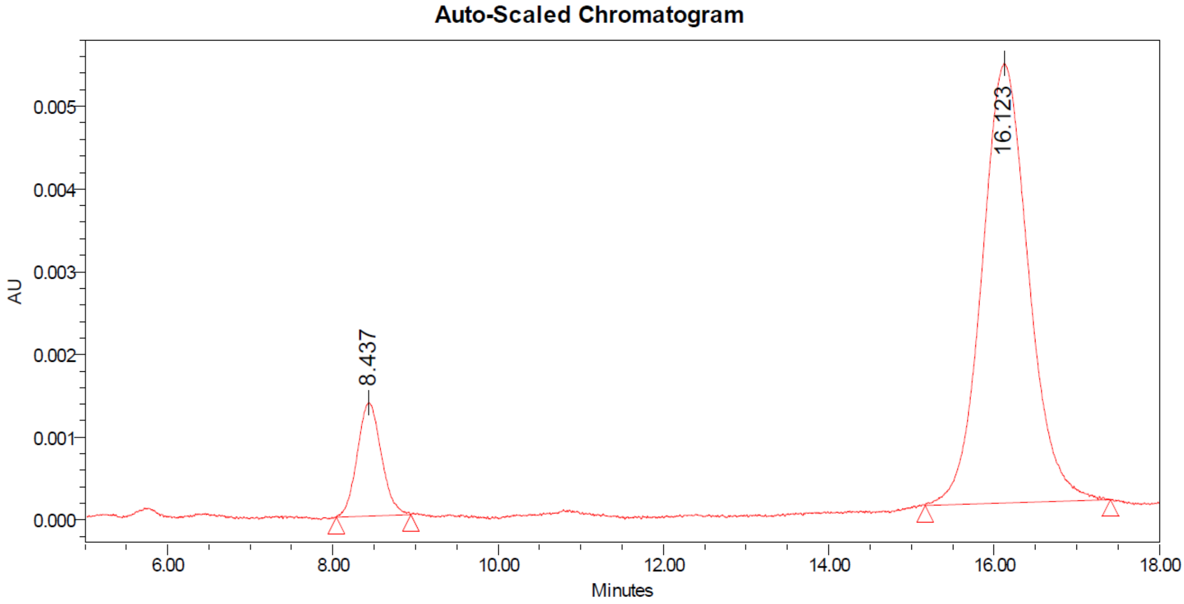

Peak Results

	Name	RT	Height	Area	% Area
1		8.437	1372	27704	11.77
2		16.123	5316	207630	88.23

4d

340 nm

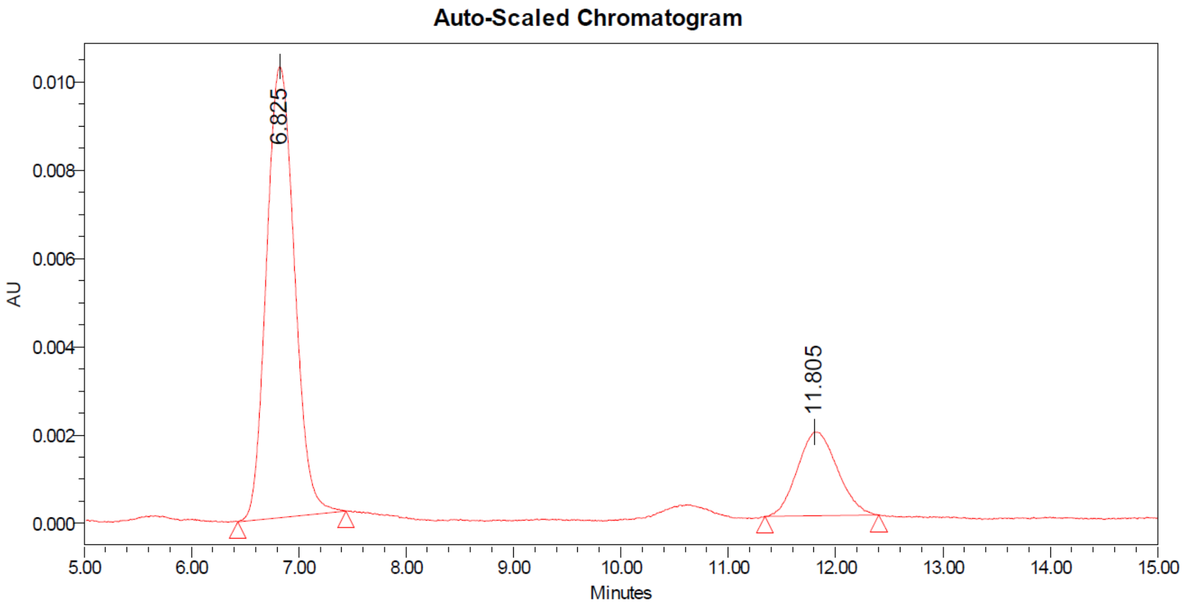

Peak Results

	Name	RT	Height	Area	% Area
1		6.825	10228	186038	79.15
2		11.805	1894	49017	20.85

365 nm

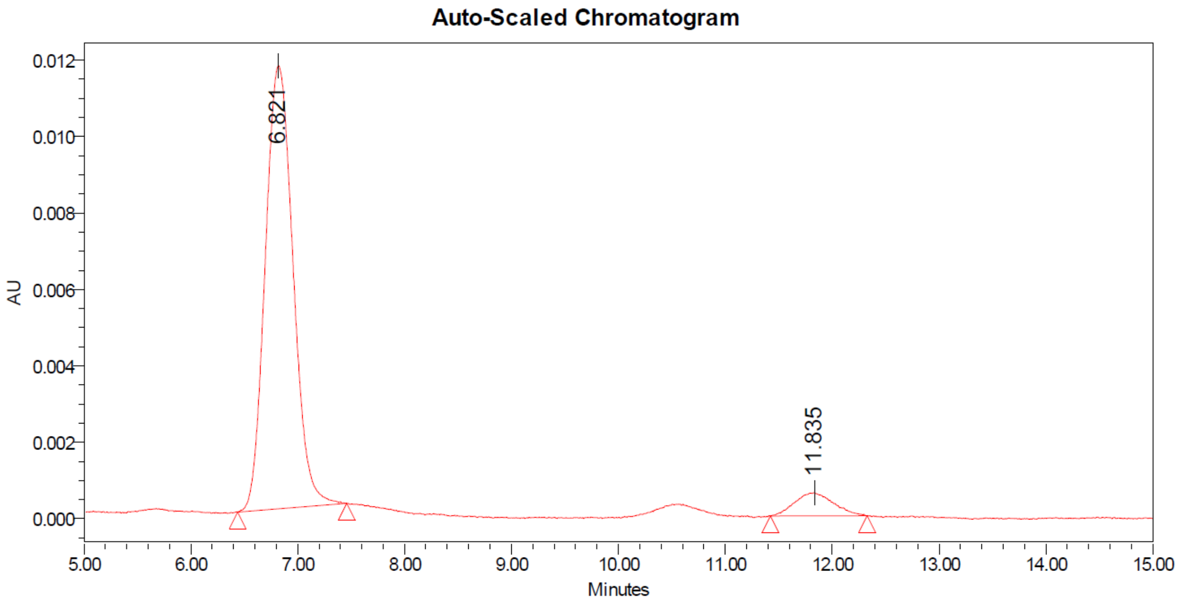

Peak Results

	Name	RT	Height	Area	% Area
1		6.821	11597	209694	93.24
2		11.835	605	15207	6.76

415 nm

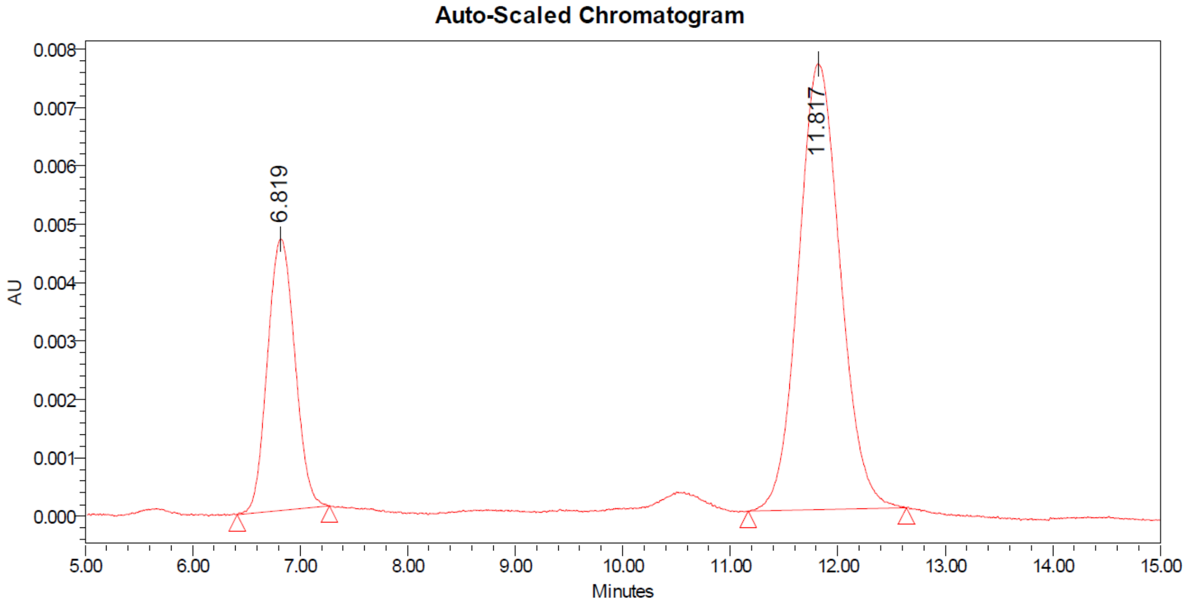

Peak Results

	Name	RT	Height	Area	% Area
1		6.819	4653	82597	28.56
2		11.817	7634	206559	71.44

455 nm

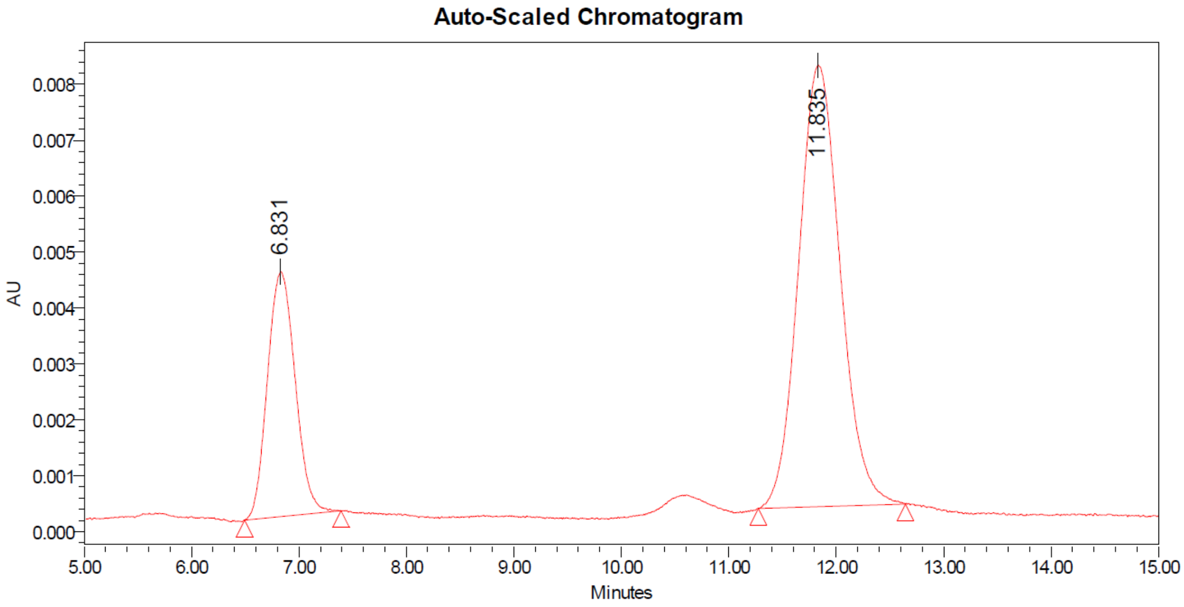

Peak Results

	Name	RT	Height	Area	% Area
1		6.831	4385	78747	27.04
2		11.835	7896	212500	72.96

505 nm

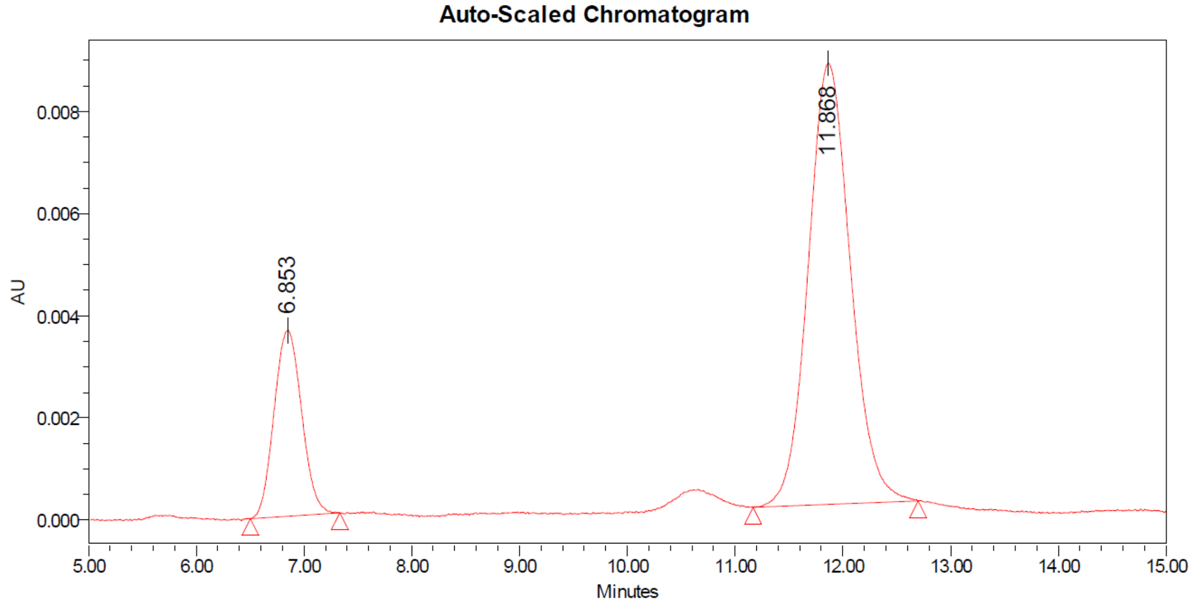

Peak Results

	Name	RT	Height	Area	% Area
1		6.853	3639	64637	21.53
2		11.868	8637	235572	78.47

530 nm

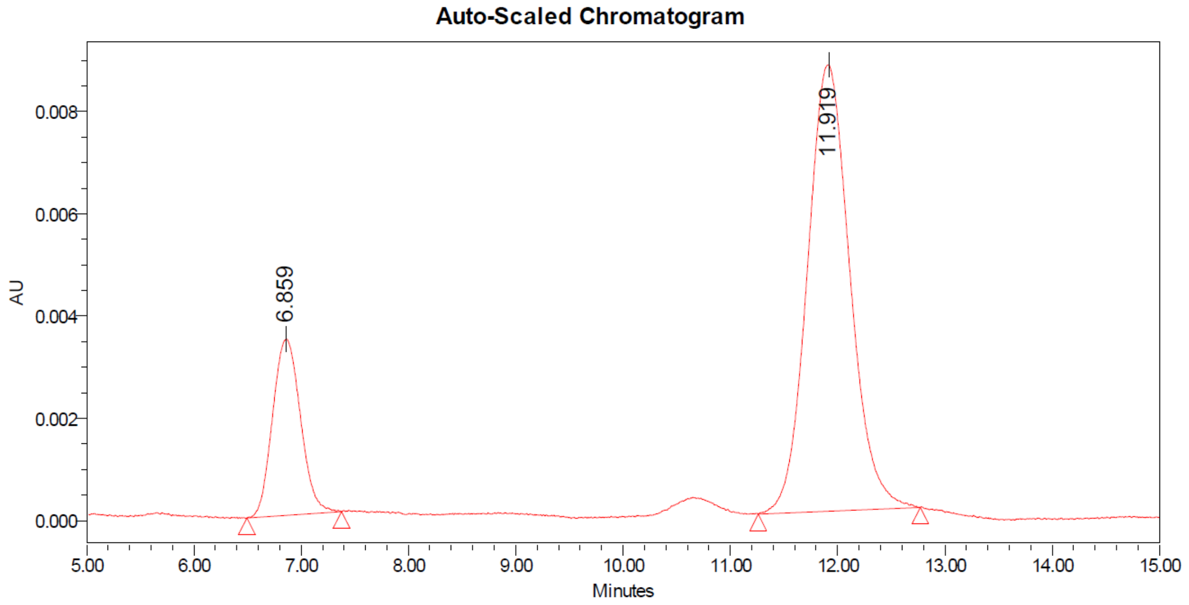

Peak Results

	Name	RT	Height	Area	% Area
1		6.859	3445	62288	20.57
2		11.919	8732	240498	79.43

4e

340 nm

Auto-Scaled Chromatogram

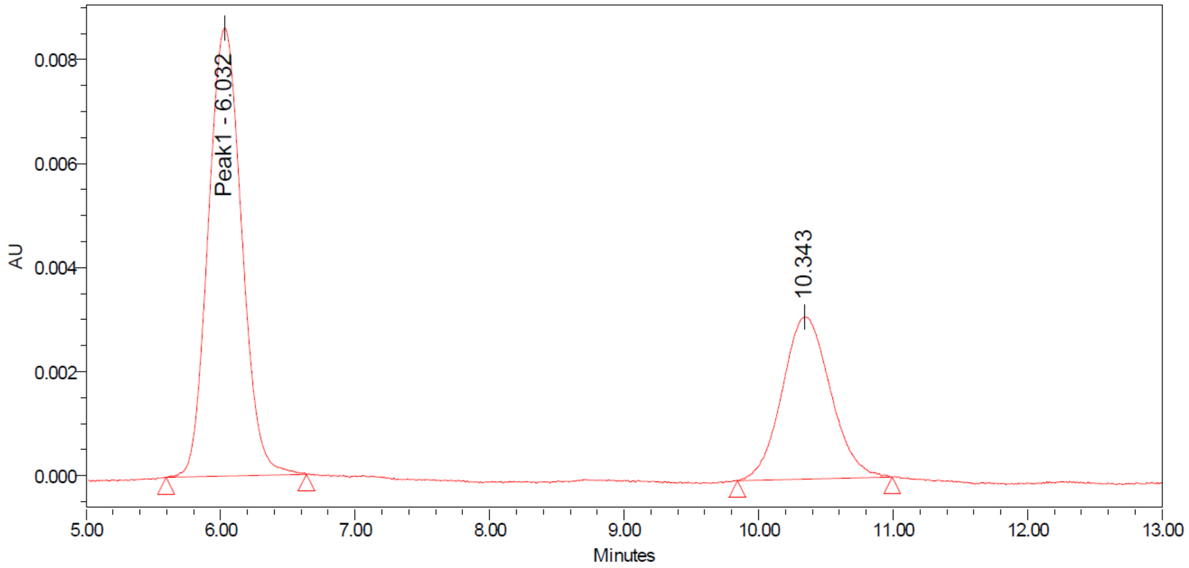

Peak Results

	Name	RT	Height	Area	% Area
1	Peak1	6.032	8619	148637	65.60
2	Peak2	7.091			
3		10.343	3124	77943	34.40

365 nm

Auto-Scaled Chromatogram

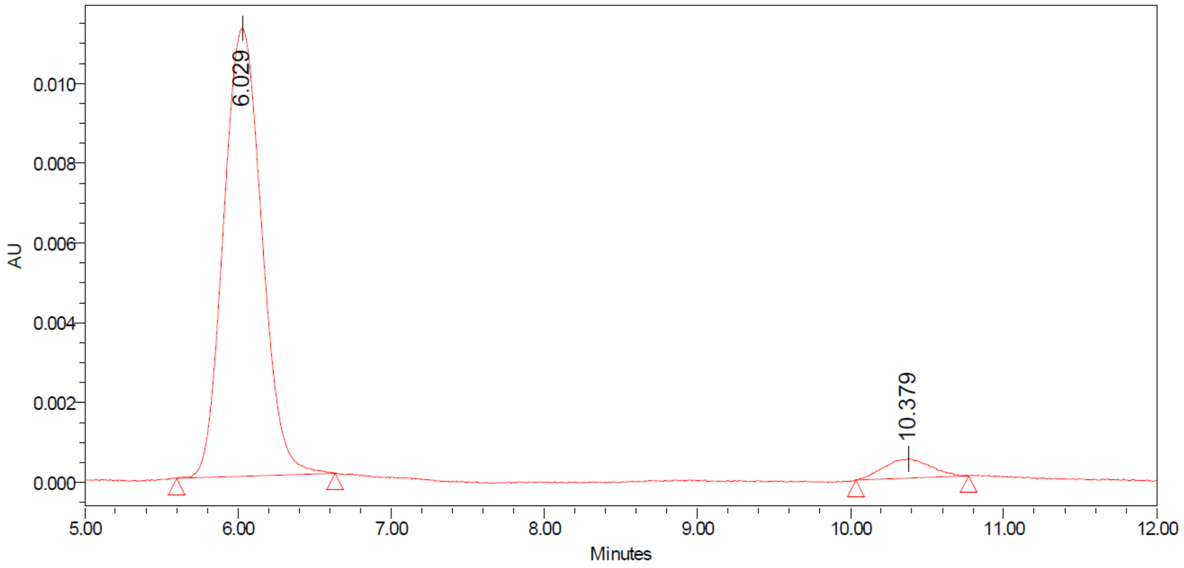

Peak Results

	Name	RT	Height	Area	% Area
1		6.029	11233	194835	95.02
2		10.379	482	10221	4.98

415 nm

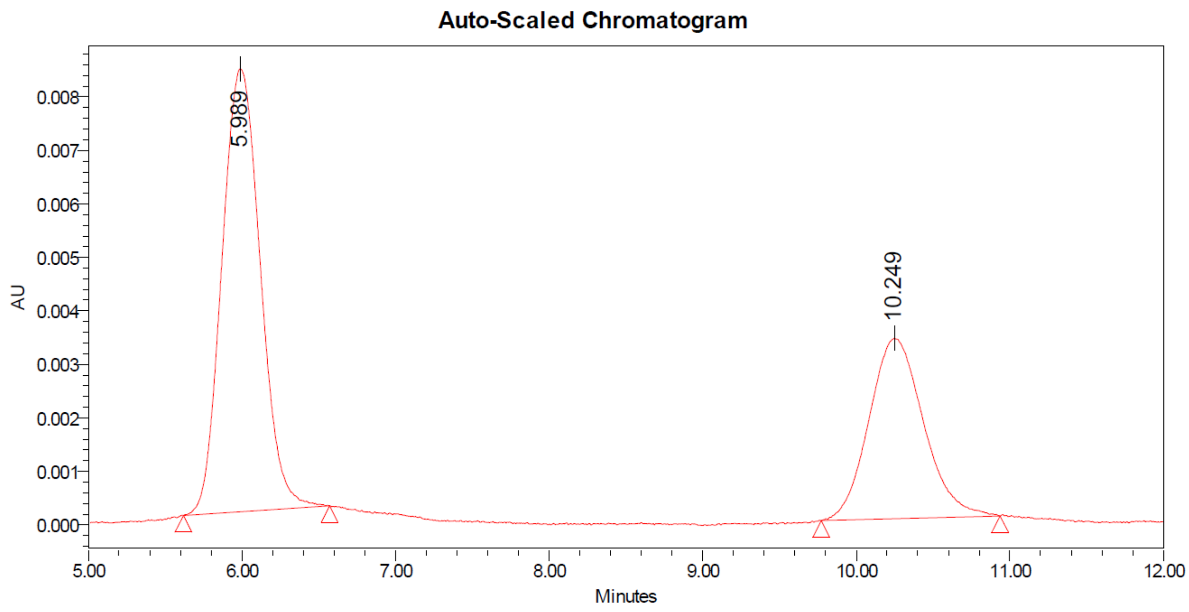

Peak Results

	Name	RT	Height	Area	% Area
1		5.989	8283	144058	63.40
2		10.249	3379	83155	36.60

455 nm

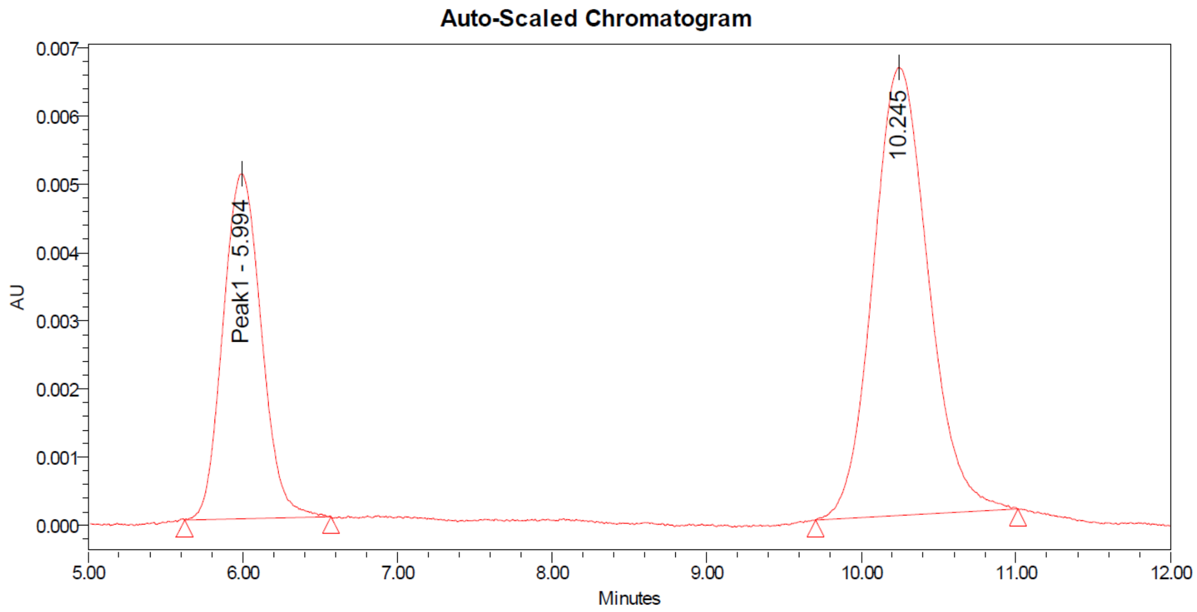

Peak Results

	Name	RT	Height	Area	% Area
1	Peak1	5.994	5058	87234	34.82
2	Peak2	7.091			
3		10.245	6570	163320	65.18

505 nm

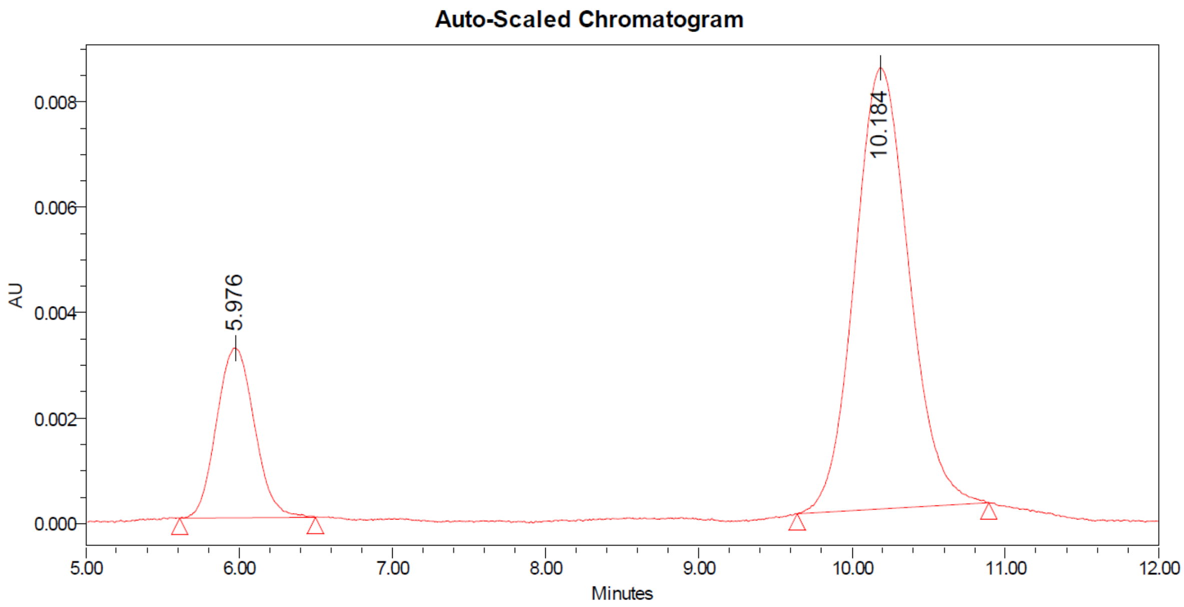

Peak Results

	Name	RT	Height	Area	% Area
1		5.976	3223	55369	21.17
2		10.184	8371	206203	78.83

530 nm

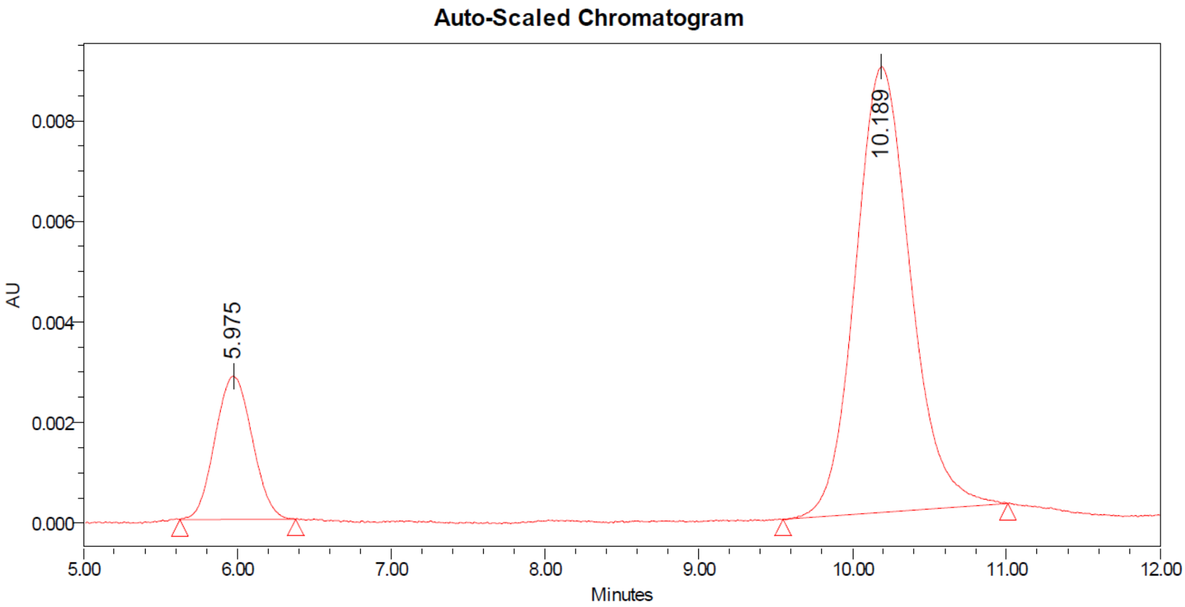

Peak Results

	Name	RT	Height	Area	% Area
1		5.975	2851	47840	17.80
2		10.189	8867	220879	82.20

4f

340 nm

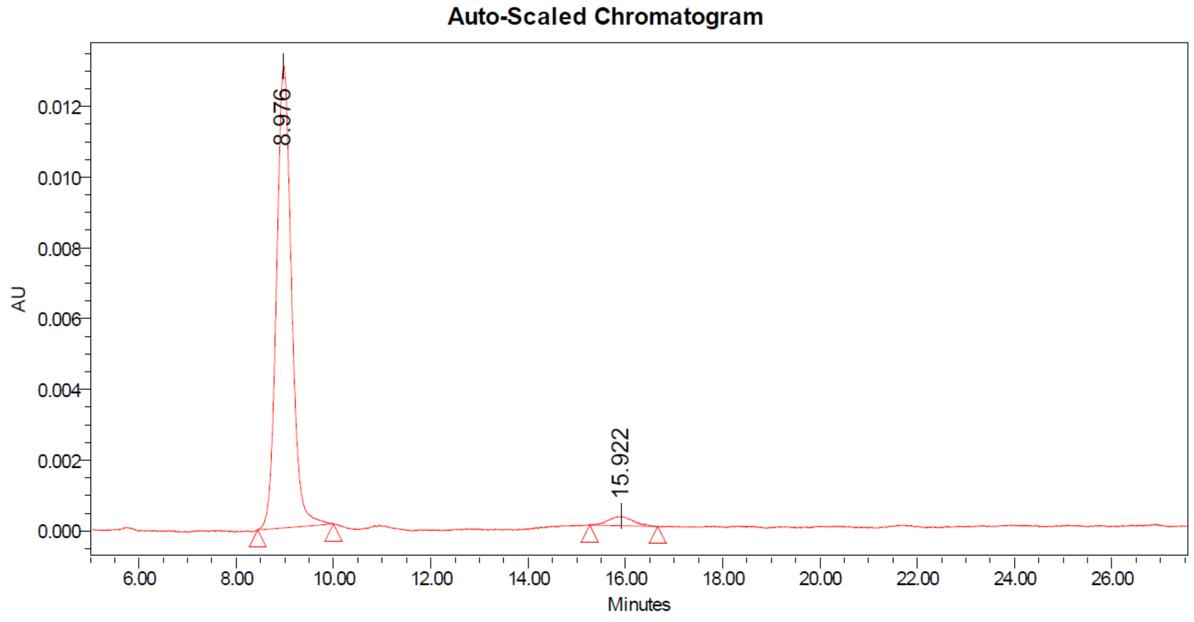

Peak Results

	Name	RT	Height	Area	% Area
1		8.976	13056	280315	96.77
2		15.922	275	9359	3.23

365 nm

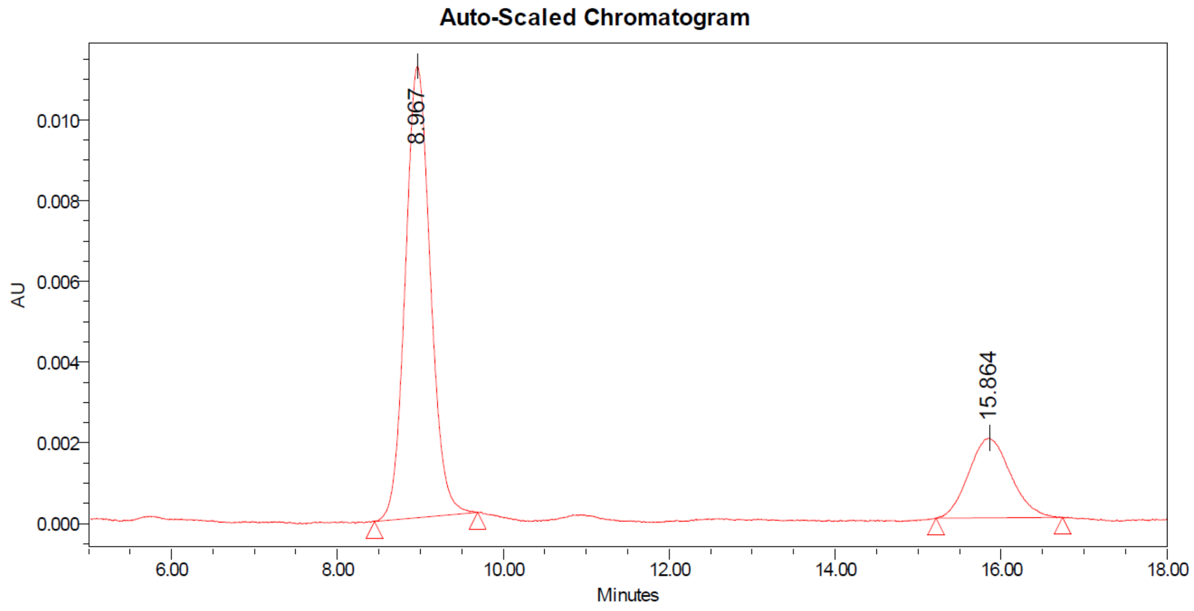

Peak Results

	Name	RT	Height	Area	% Area
1		8.967	1118[illegible]	234539	77.22
2		15.864	1975	69207	22.78

415 nm

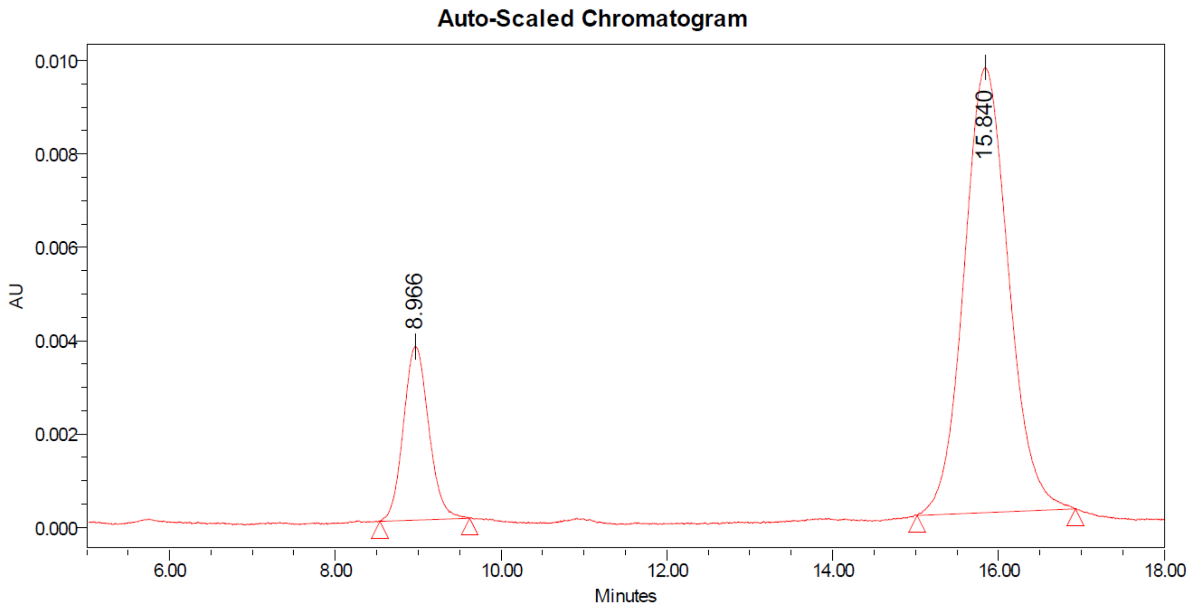

Peak Results

	Name	RT	Height	Area	% Area
1		8.966	3720	79100	18.15
2		15.840	9540	356745	81.85

455 nm

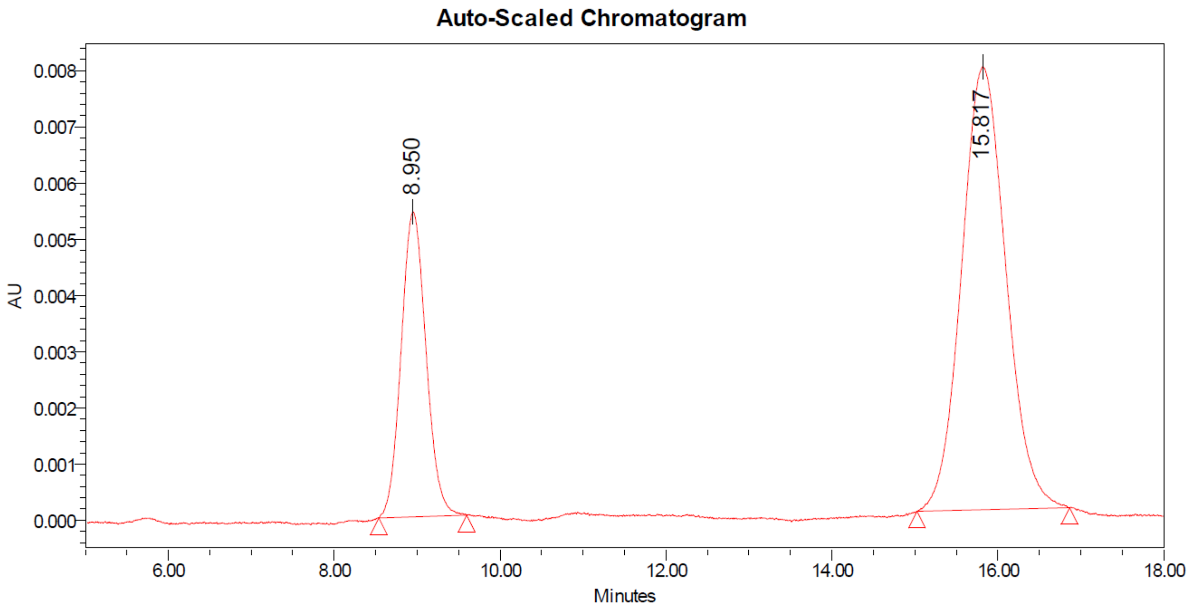

Peak Results

	Name	RT	Height	Area	% Area
1		8.950	5433	113210	27.84
2		15.817	7887	293488	72.16

505 nm

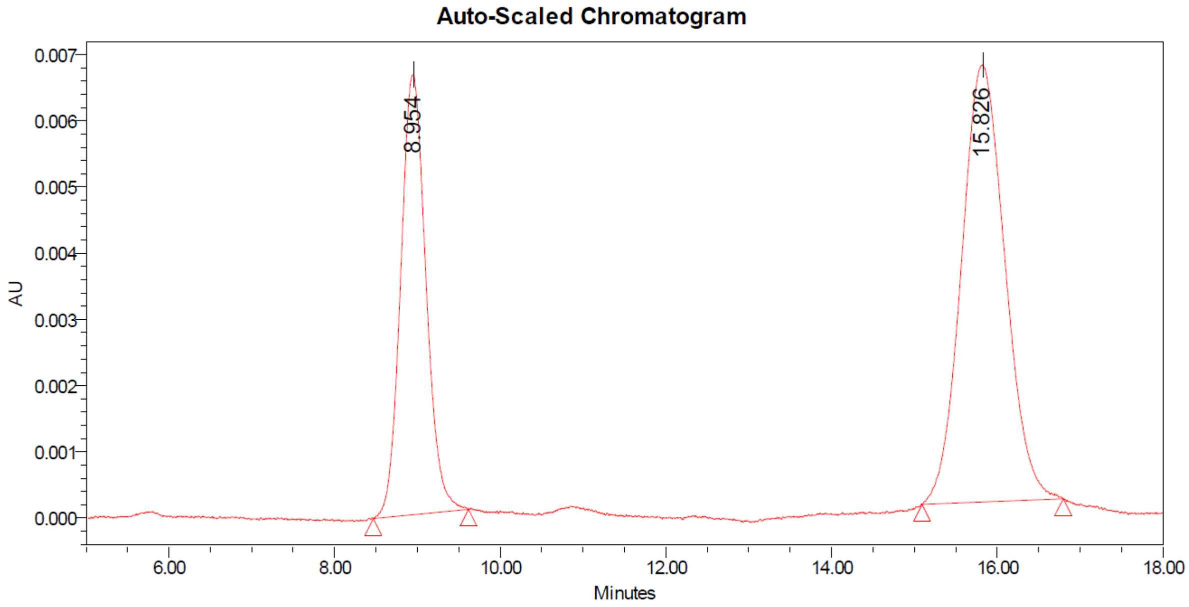

Peak Results

	Name	RT	Height	Area	% Area
1		8.954	6652	140645	36.87
2		15.826	6604	240829	63.13

530 nm

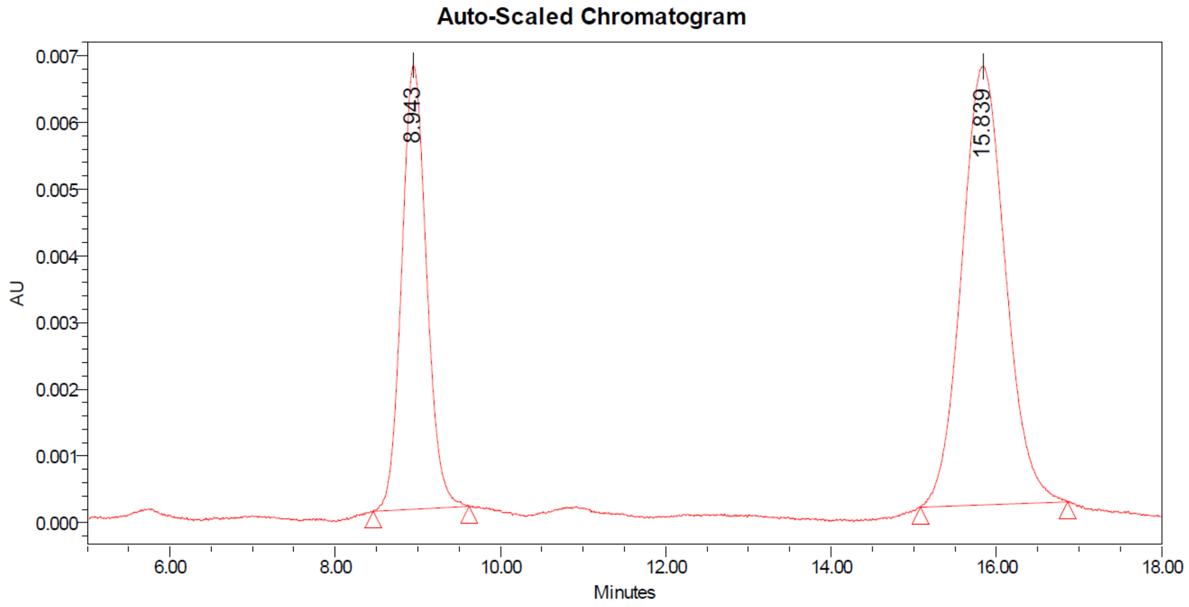

Peak Results

	Name	RT	Height	Area	% Area
1		8.943	6659	138999	36.45
2		15.839	6577	242389	63.55

4g

340 nm

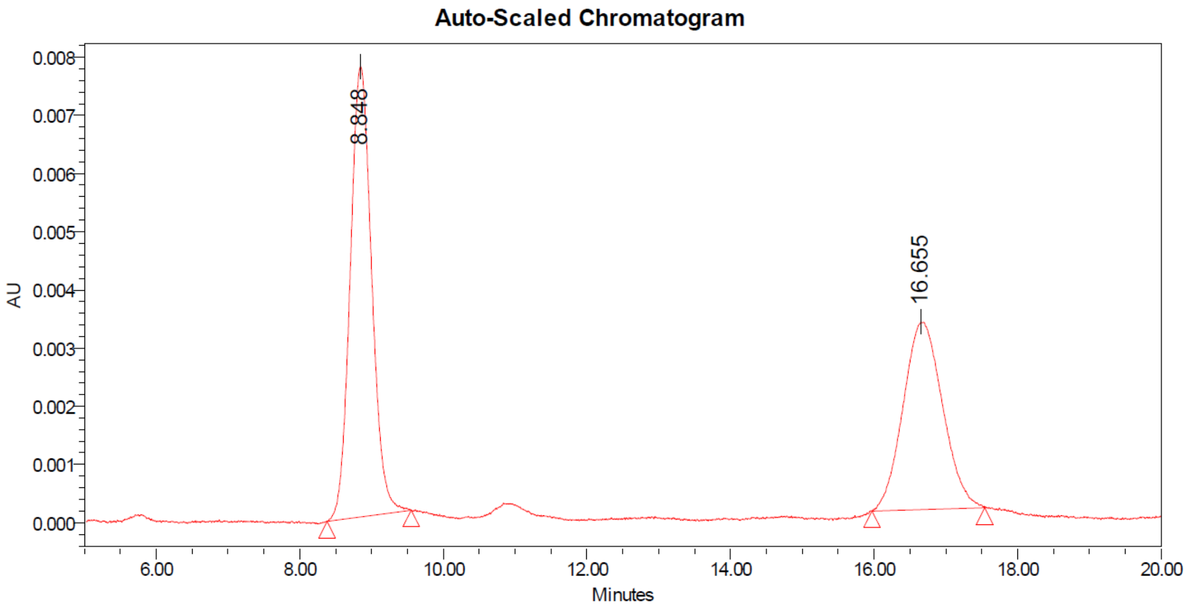

Peak Results

	Name	RT	Height	Area	% Area
1		8.848	7739	160401	56.62
2		16.655	3223	122886	43.38

365 nm

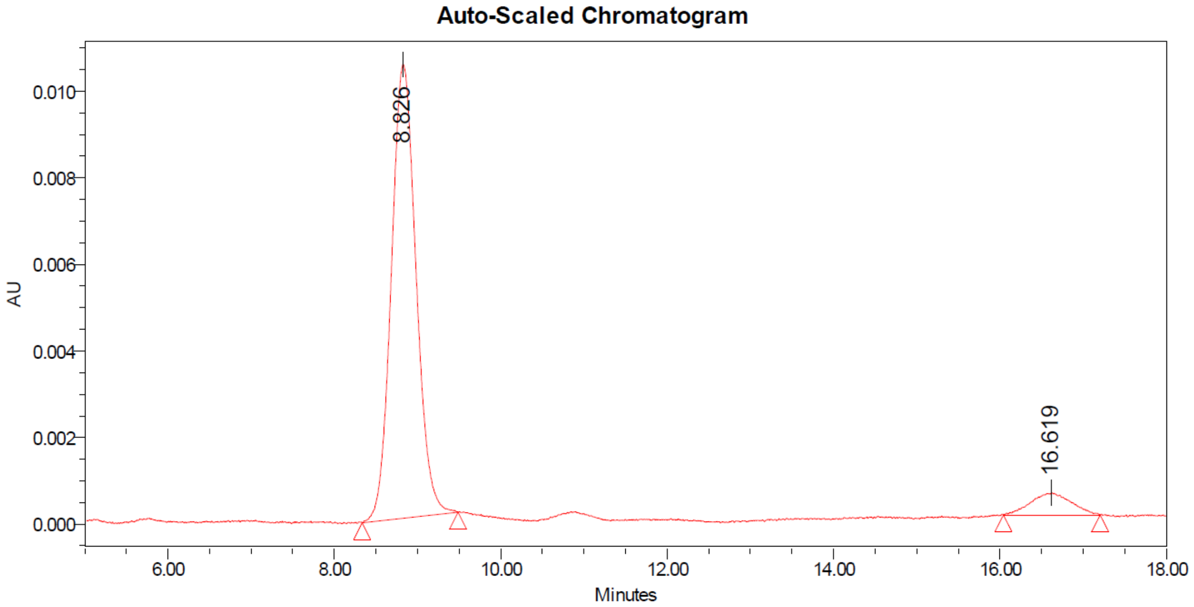

Peak Results

	Name	RT	Height	Area	% Area
1		8.826	10489	215906	92.66
2		16.619	514	17106	7.34

415 nm

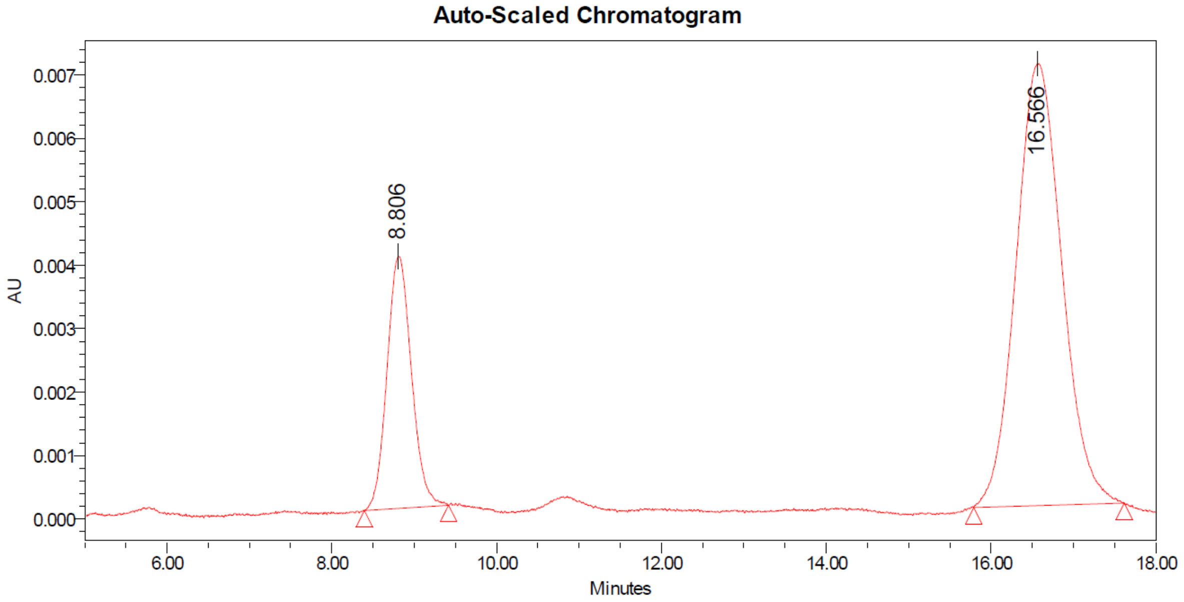

Peak Results

	Name	RT	Height	Area	% Area
1		8.806	3969	82043	23.33
2		16.566	6969	269615	76.67

455 nm

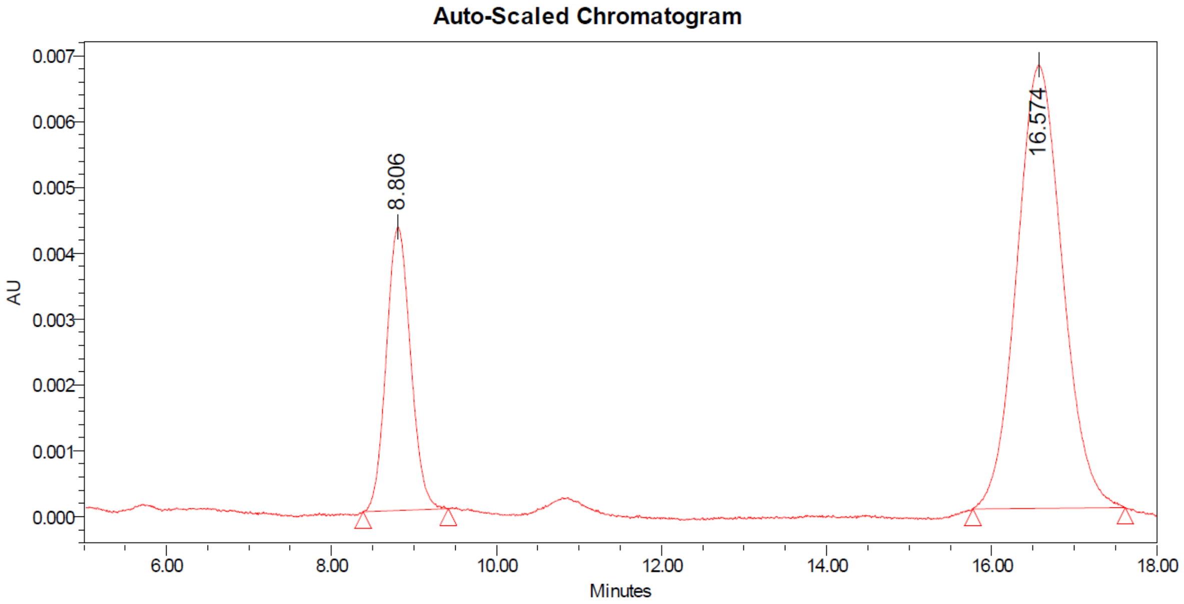

Peak Results

	Name	RT	Height	Area	% Area
1		8.806	4310	88248	25.40
2		16.574	6742	259191	74.60

505 nm

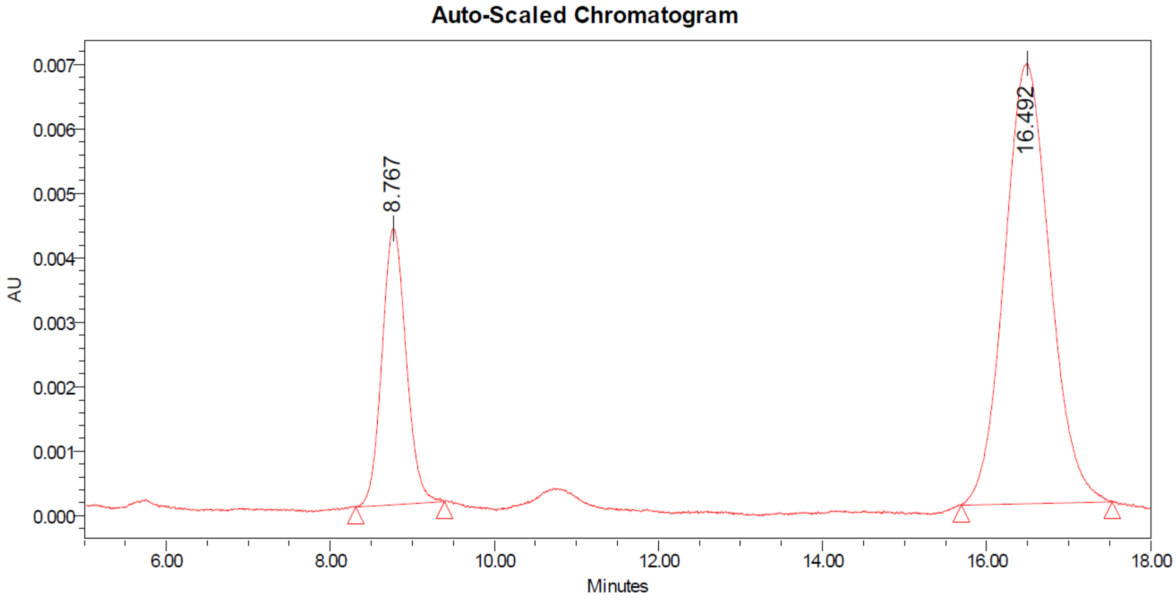

Peak Results

	Name	RT	Height	Area	% Area
1		8.767	4285	87669	24.97
2		16.492	6834	263470	75.03

530 nm

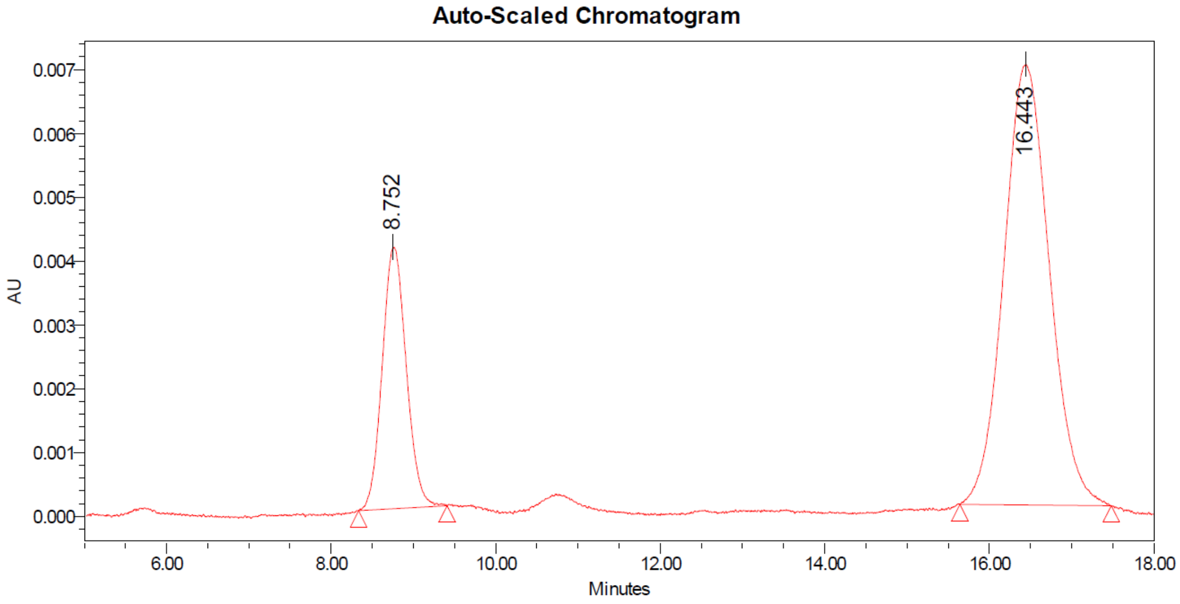

Peak Results

	Name	RT	Height	Area	% Area
1		8.752	4101	83349	23.89
2		16.443	6911	265574	76.11

4i

340 nm

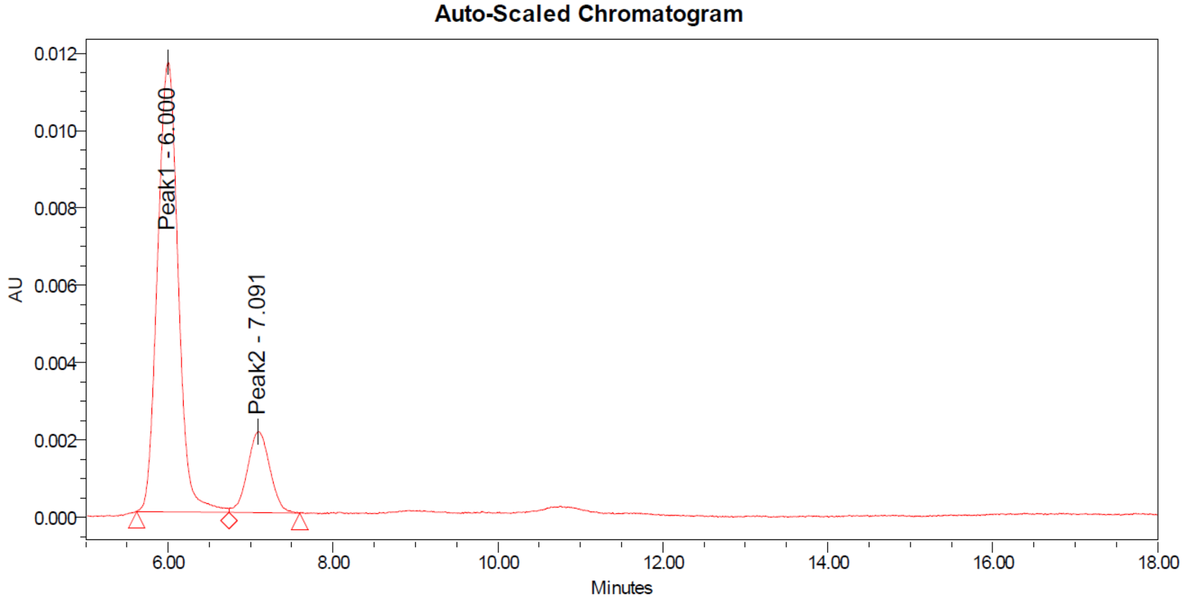

Peak Results

	Name	RT	Height	Area	% Area
1	Peak1	6.000	11622	201521	83.61
2	Peak2	7.091	2100	39505	16.39

365 nm

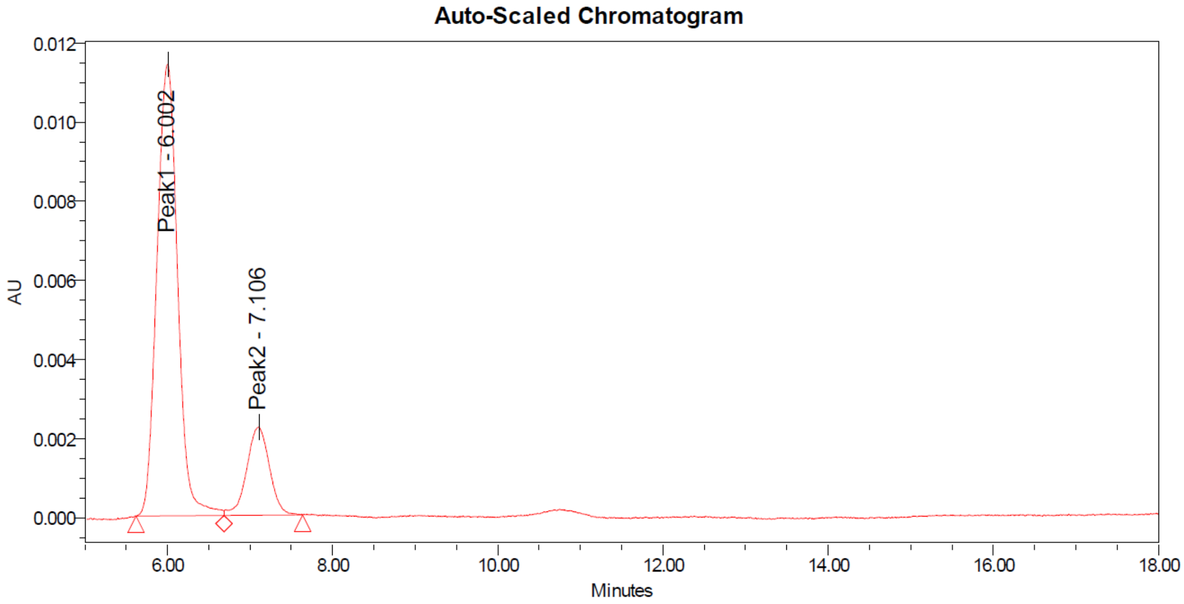

Peak Results

	Name	RT	Height	Area	% Area
1	Peak1	6.002	11412	198730	82.33
2	Peak2	7.106	2227	42647	17.67

415 nm

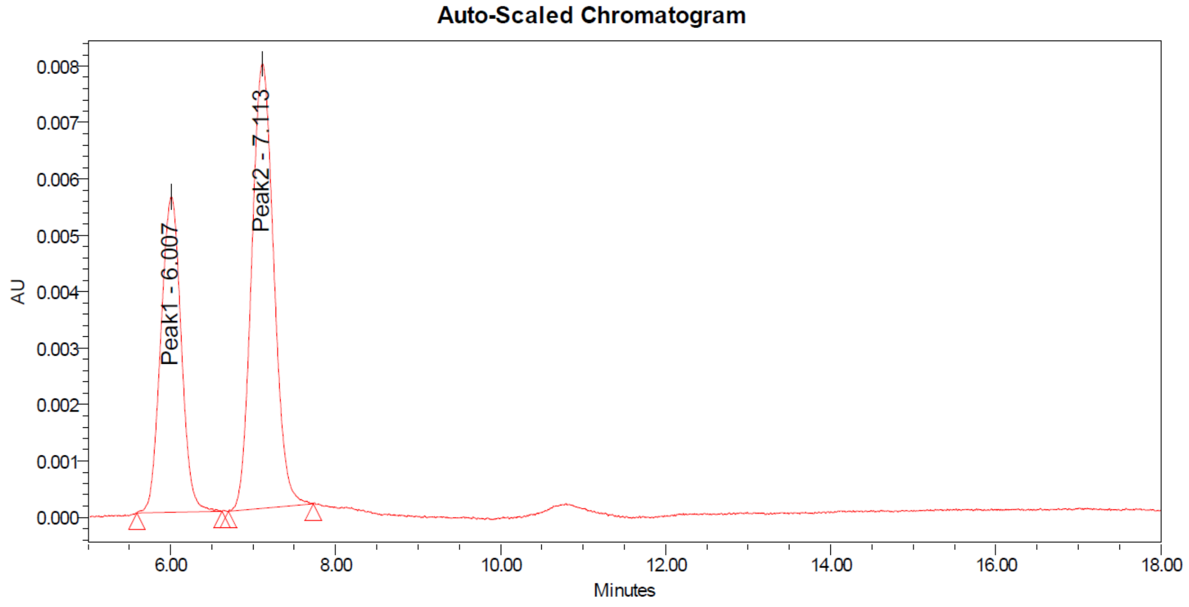

Peak Results

	Name	RT	Height	Area	% Area
1	Peak1	6.007	5592	96061	39.53
2	Peak2	7.113	7883	146939	60.47

455 nm

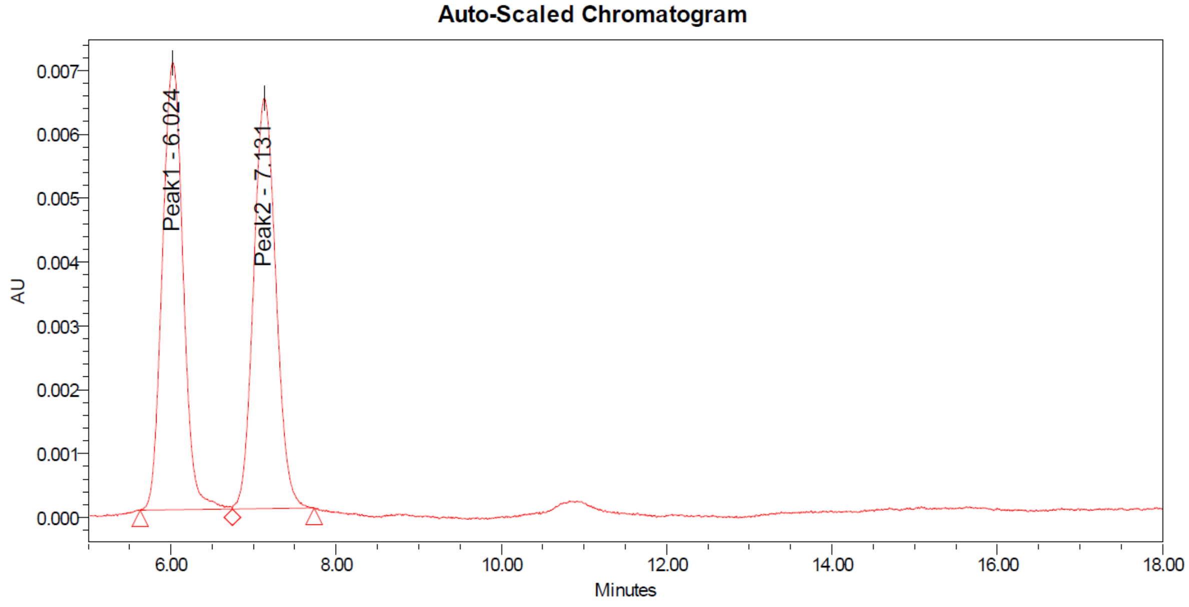

Peak Results

	Name	RT	Height	Area	% Area
1	Peak1	6.024	7001	122047	50.21
2	Peak2	7.131	6428	121034	49.79

505 nm

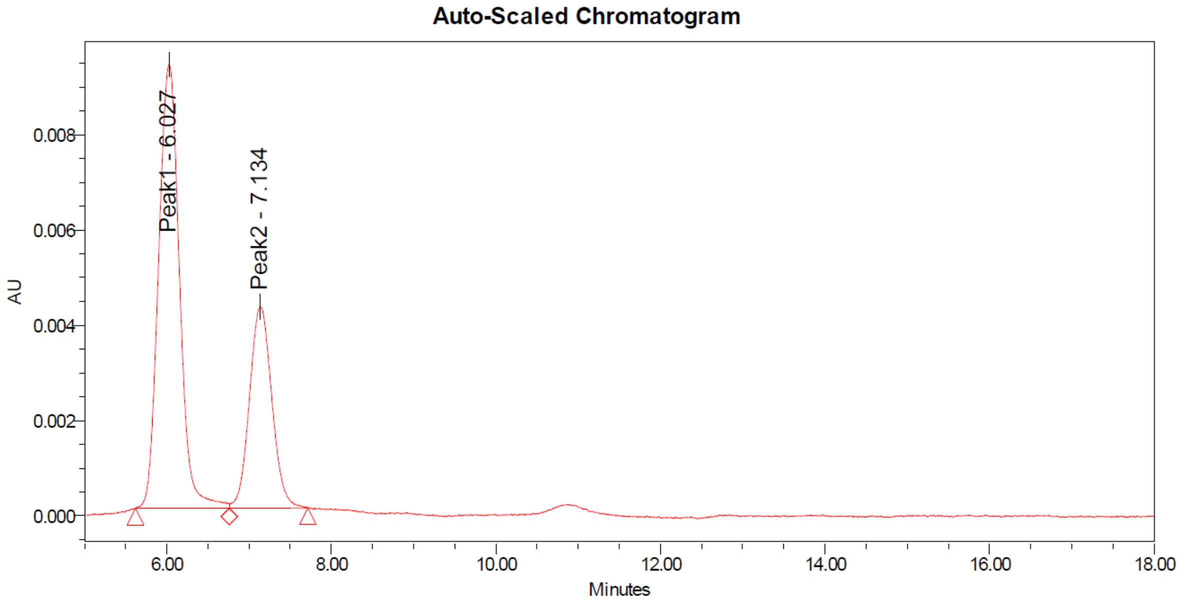

Peak Results

	Name	RT	Height	Area	% Area
1	Peak1	6.027	9321	164085	67.23
2	Peak2	7.134	4226	79974	32.77

530 nm

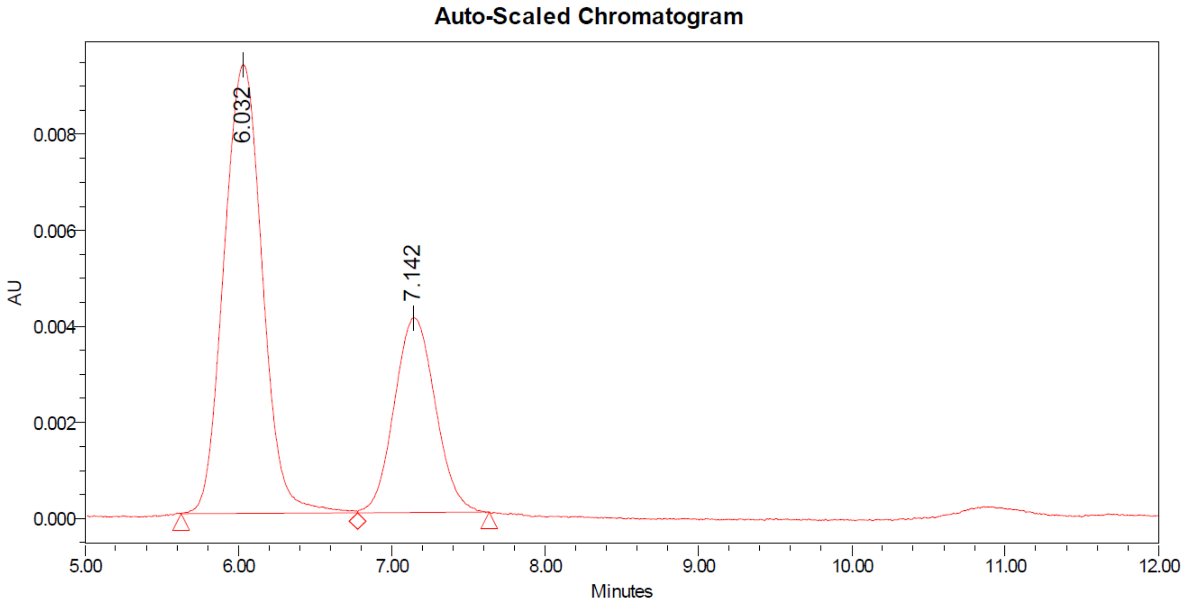

Peak Results

	Name	RT	Height	Area	% Area
1		6.032	9339	162693	68.35
2		7.142	4048	75351	31.65

4j

340 nm

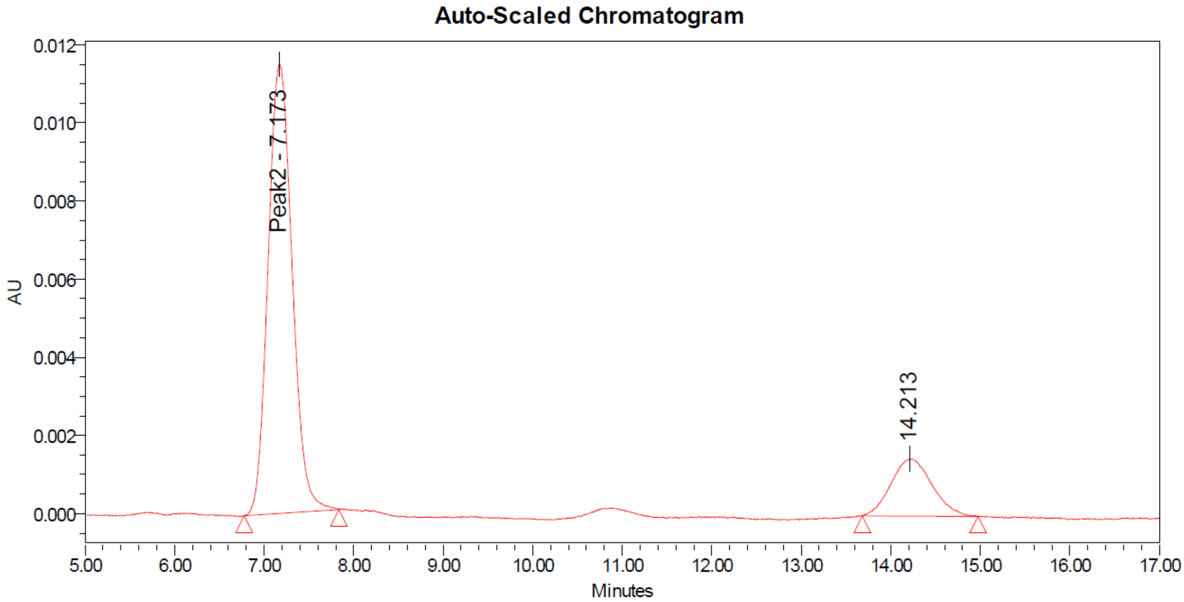

Peak Results

	Name	RT	Height	Area	% Area
1	Peak1	6.000			
2	Peak2	7.173	11502	214937	82.19
3		14.213	1470	46590	17.81

365 nm

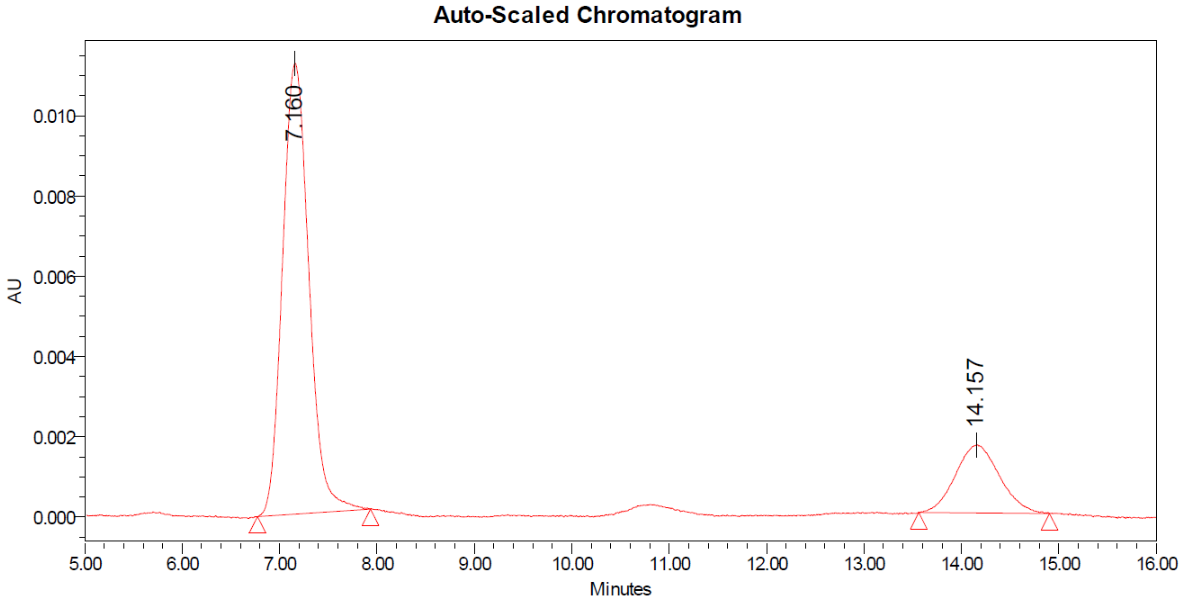

Peak Results

	Name	RT	Height	Area	% Area
1		7.160	11231	211097	79.85
2		14.157	1698	53286	20.15

415 nm

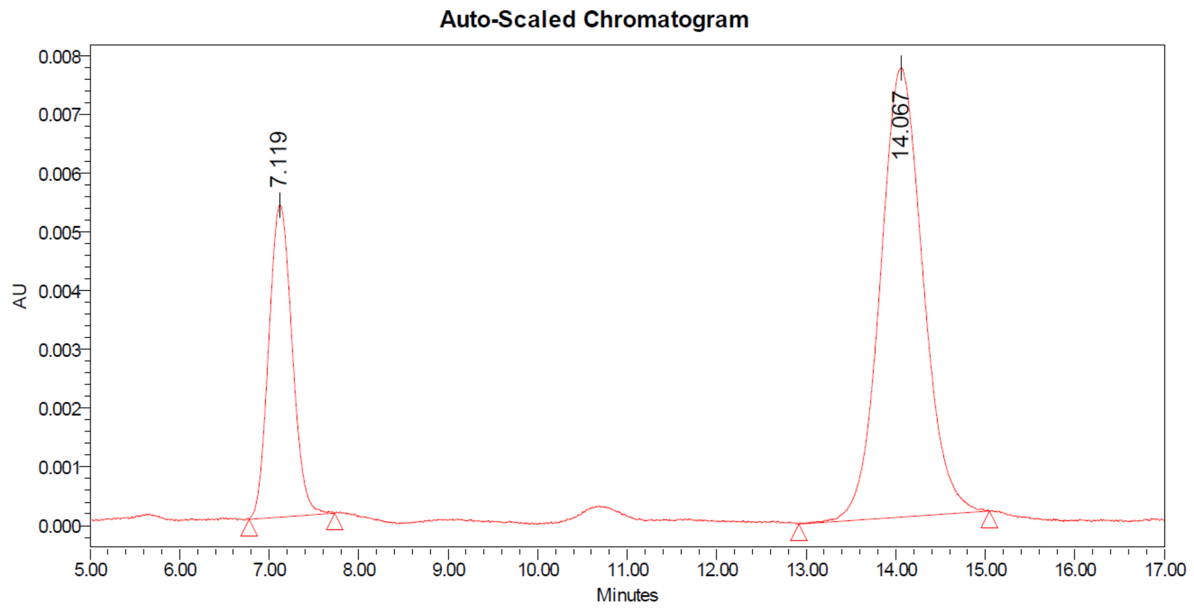

Peak Results

	Name	RT	Height	Area	% Area
1		7.119	5311	96587	27.62
2		14.067	7643	253169	72.38

455 nm

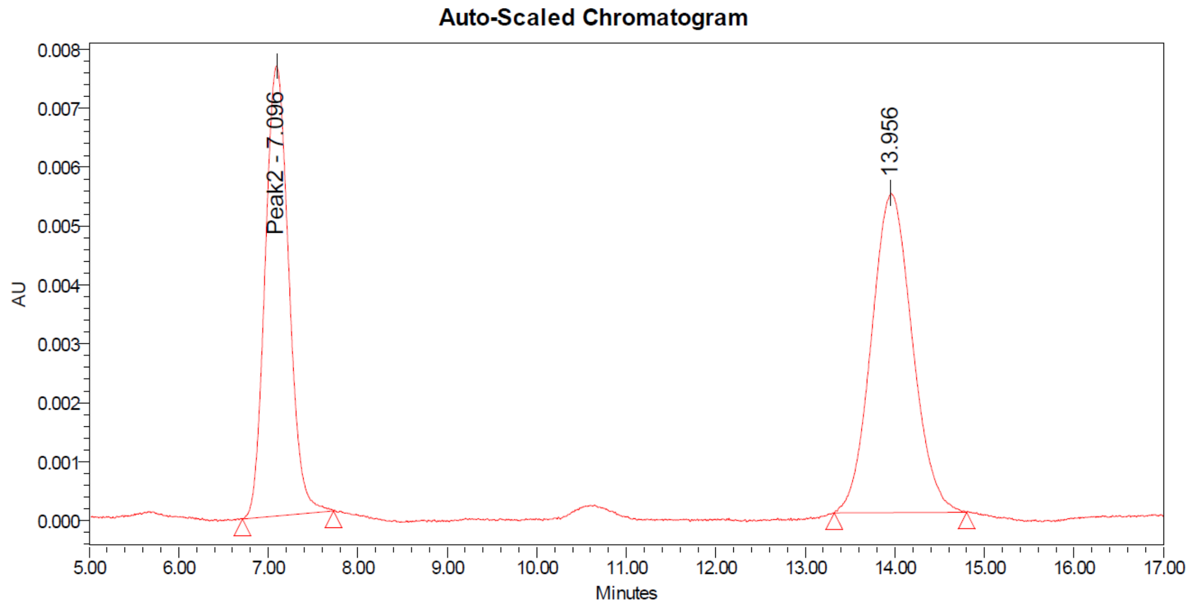

Peak Results

	Name	RT	Height	Area	% Area
1	Peak1	6.000			
2	Peak2	7.096	7640	140305	44.96
3		13.956	5423	171780	55.04

505 nm

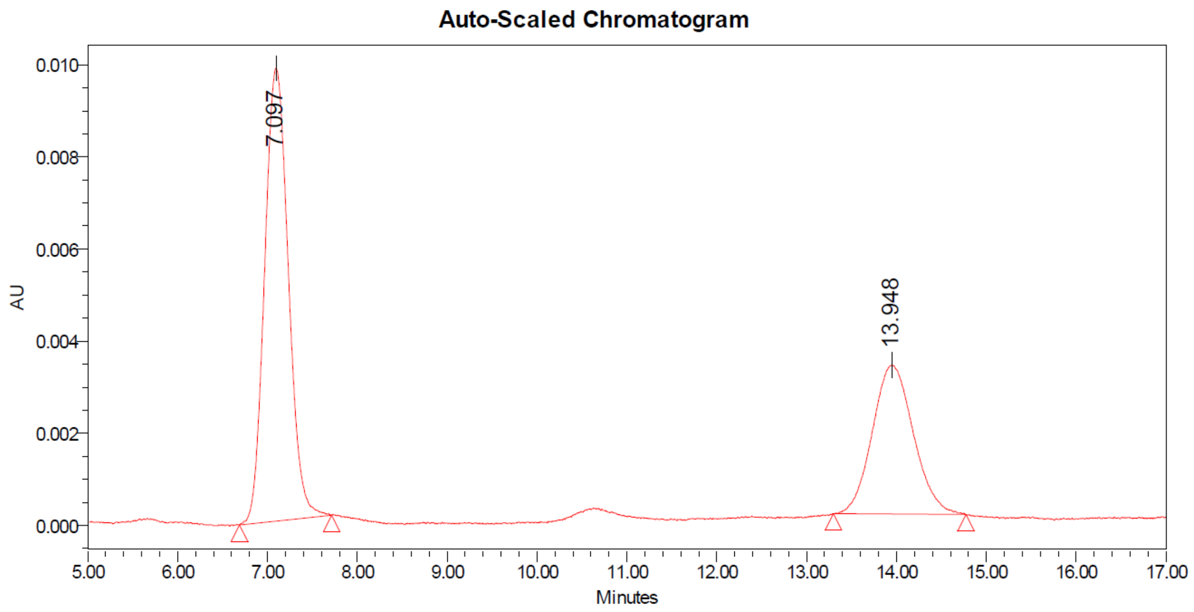

Peak Results

	Name	RT	Height	Area	% Area
1		7.097	9834	180576	63.79
2		13.948	3235	102521	36.21

530 nm

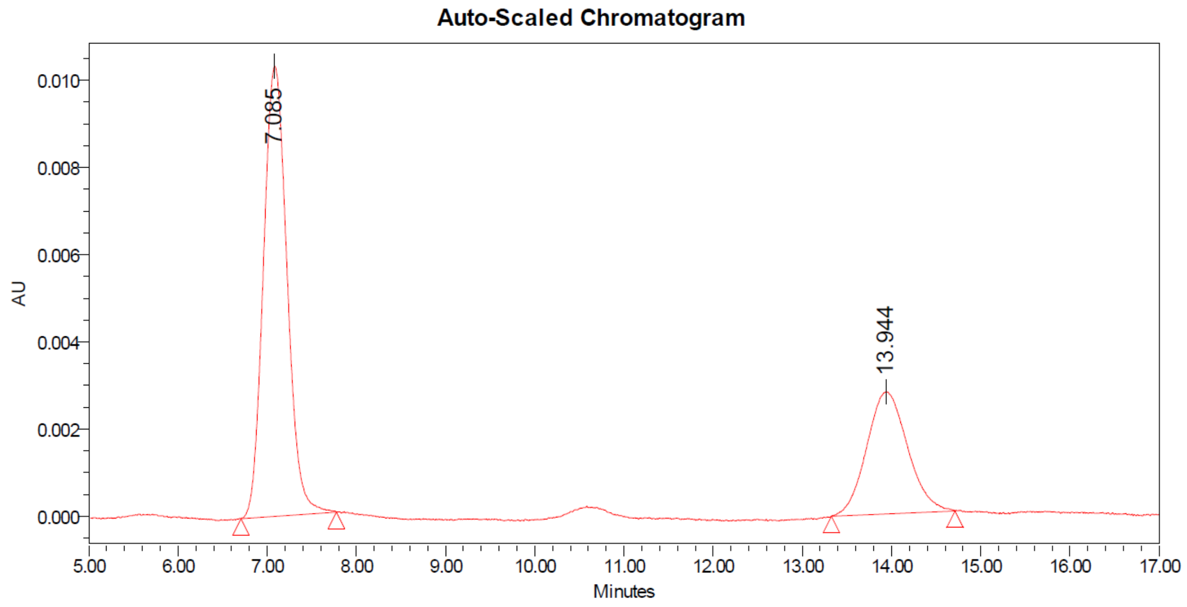

Peak Results

	Name	RT	Height	Area	% Area
1		7.085	10325	190184	68.09
2		13.944	2803	89138	31.91

4k

340 nm

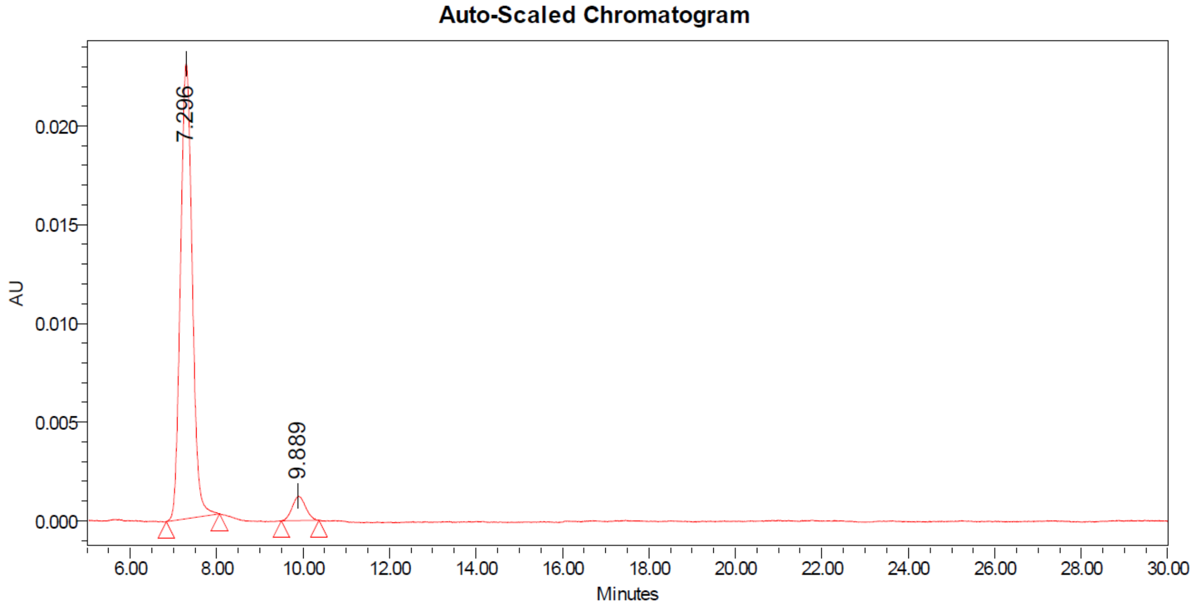

Peak Results

	Name	RT	Height	Area	% Area
1		7.296	23047	433502	94.26
2		9.889	1242	26392	5.74

365 nm

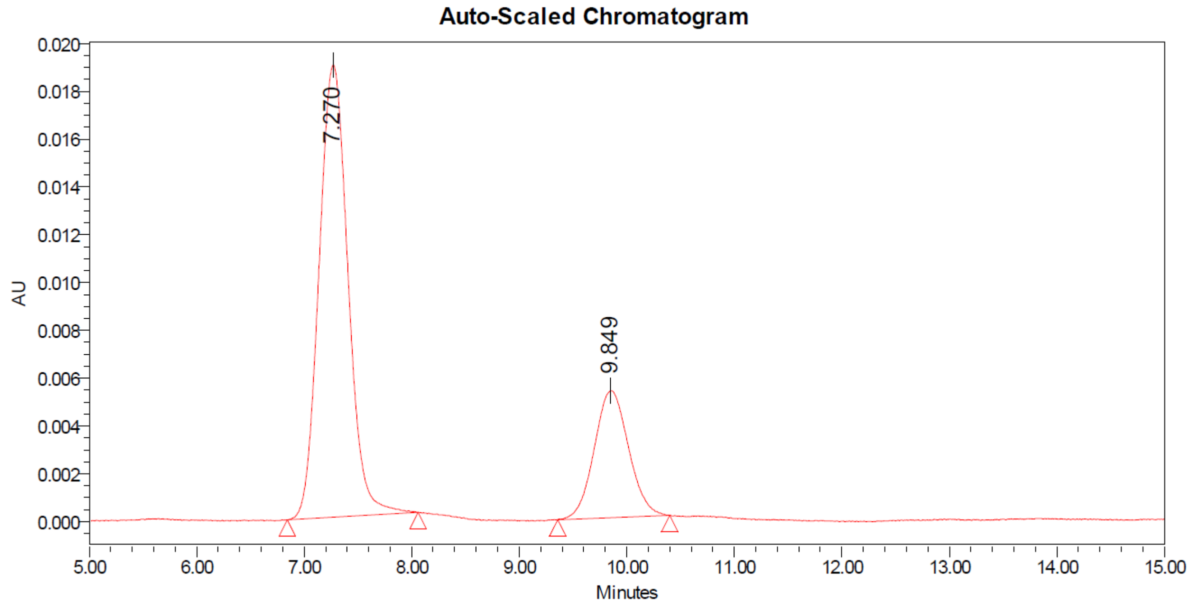

Peak Results

	Name	RT	Height	Area	% Area
1		7.270	18921	354278	75.08
2		9.849	5309	117577	24.92

415 nm

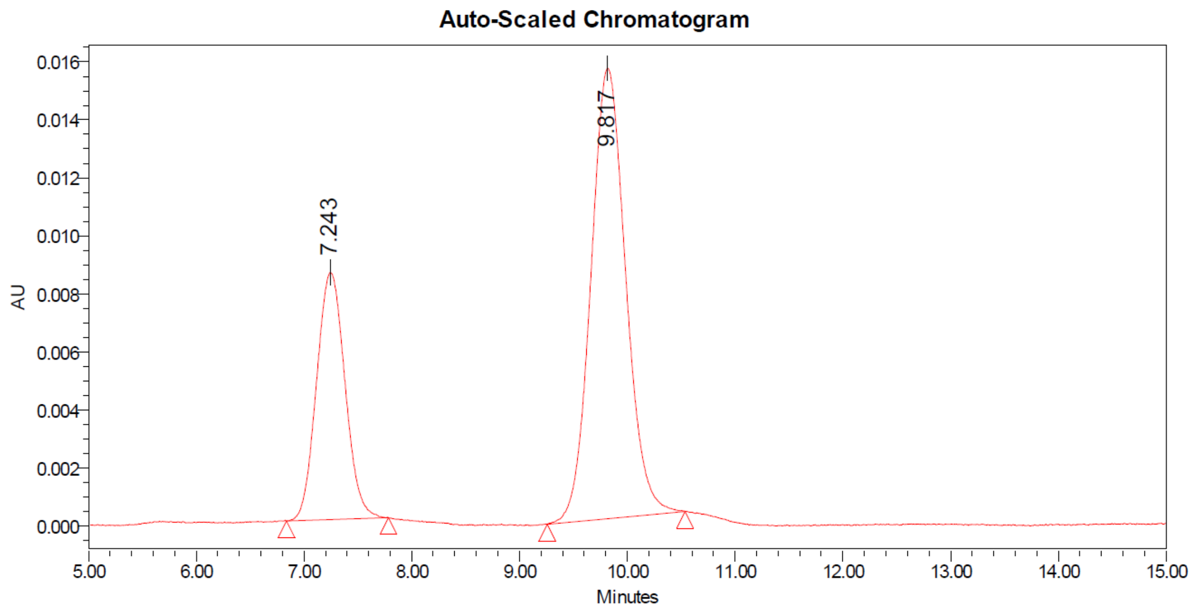

Peak Results

	Name	RT	Height	Area	% Area
1		7.243	8526	156500	31.02
2		9.817	15529	347972	68.98

455 nm

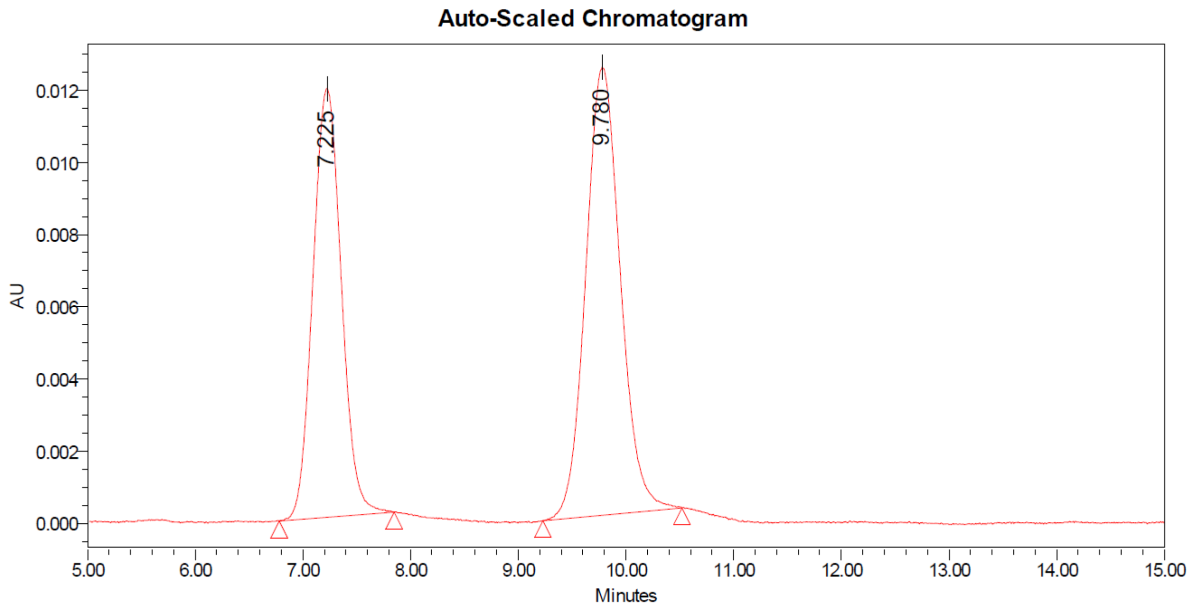

Peak Results

	Name	RT	Height	Area	% Area
1		7.225	11888	218256	44.01
2		9.780	12430	277723	55.99

505 nm

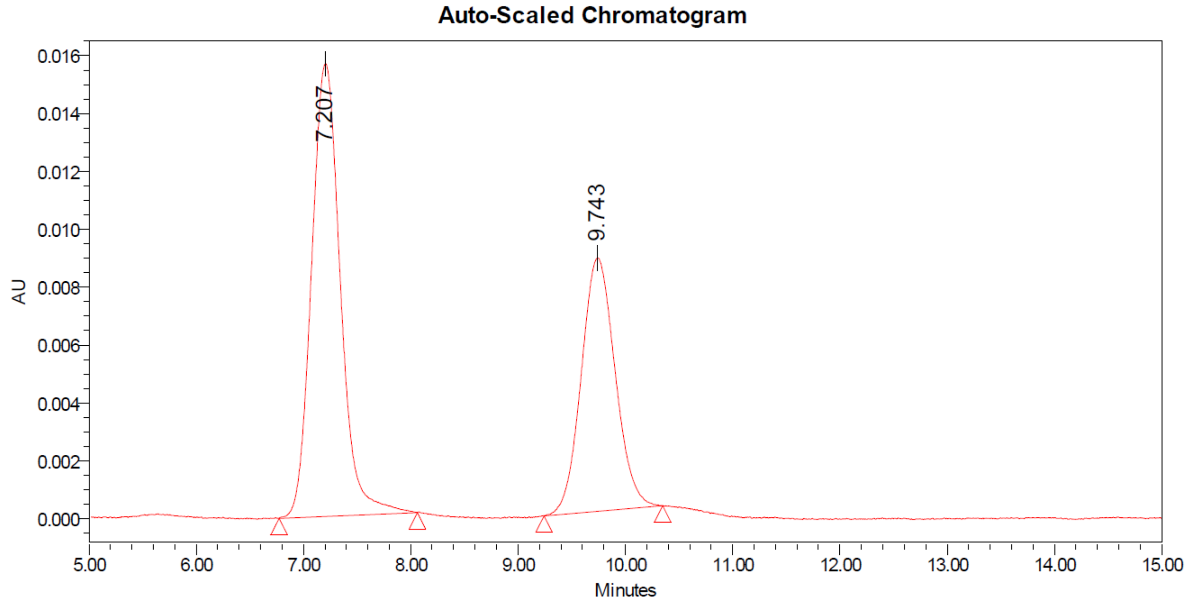

Peak Results

	Name	RT	Height	Area	% Area
1		7.207	15641	292155	60.36
2		9.743	8748	191904	39.64

530 nm

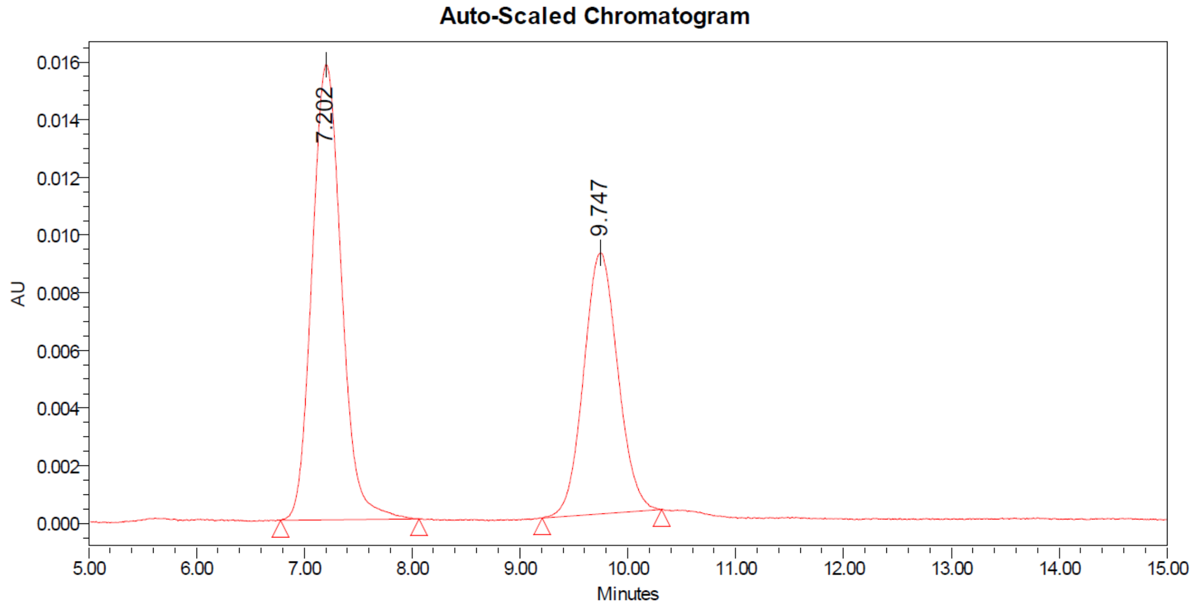

Peak Results

	Name	RT	Height	Area	% Area
1		7.202	15787	300207	60.08
2		9.747	9050	199440	39.92

4m

340 nm

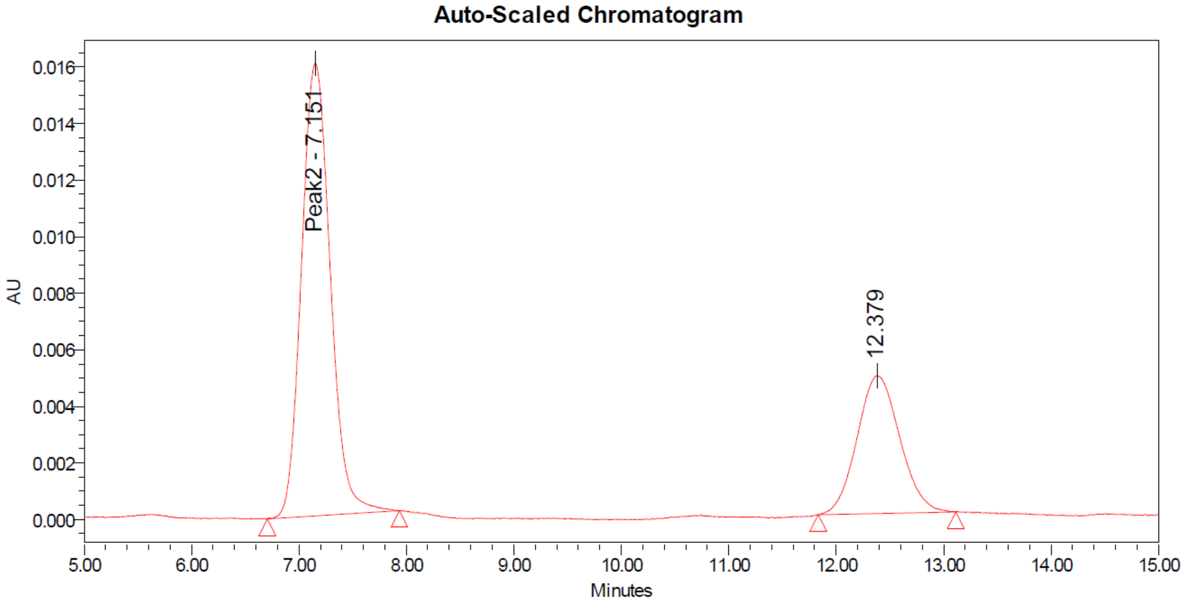

Peak Results

	Name	RT	Height	Area	% Area
1	Peak1	6.000			
2	Peak2	7.151	15999	298034	68.88
3		12.379	4875	134646	31.12

365 nm

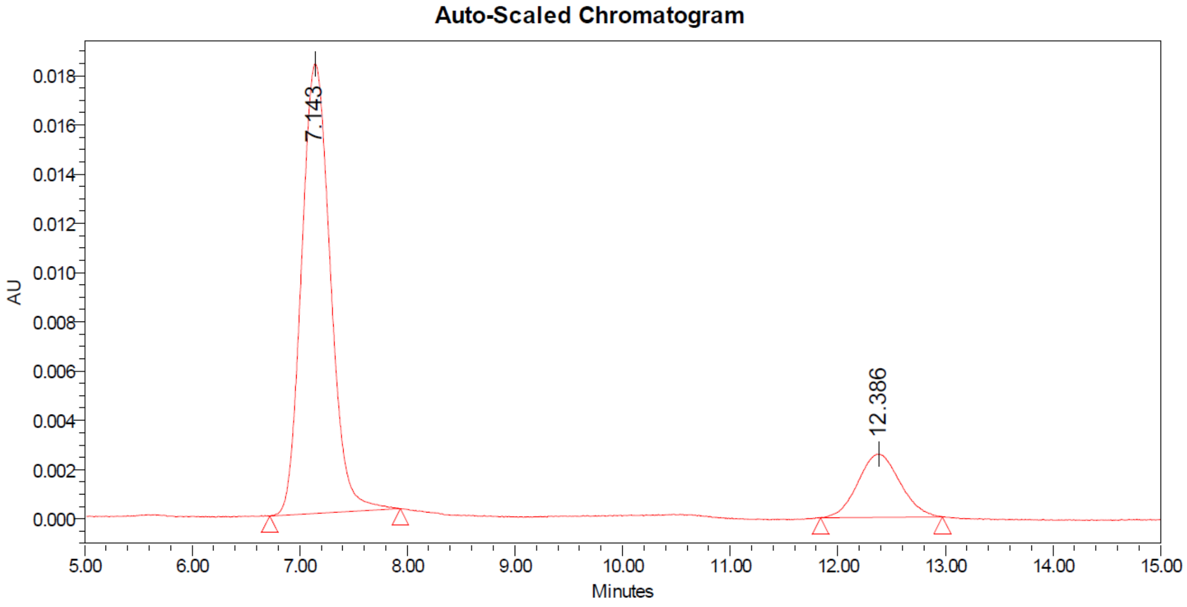

Peak Results

	Name	RT	Height	Area	% Area
1		7.143	18257	339987	82.97
2		12.386	2571	69798	17.03

415 nm

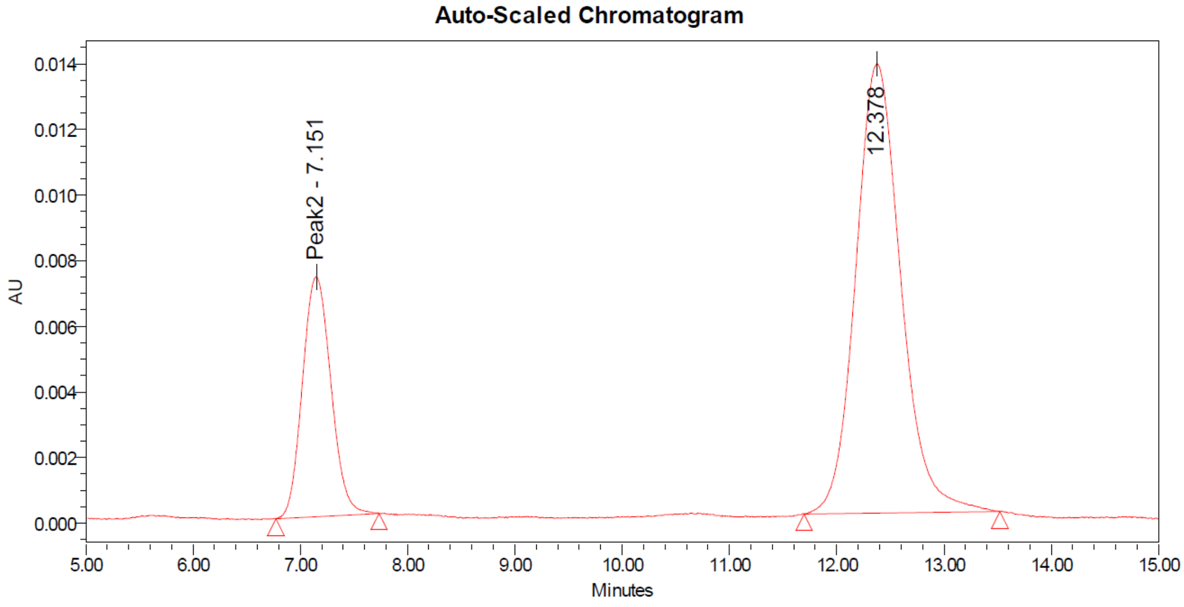

Peak Results

	Name	RT	Height	Area	% Area
1	Peak1	6.000			
2	Peak2	7.151	7309	135253	25.24
3		12.378	13700	400675	74.76

455 nm

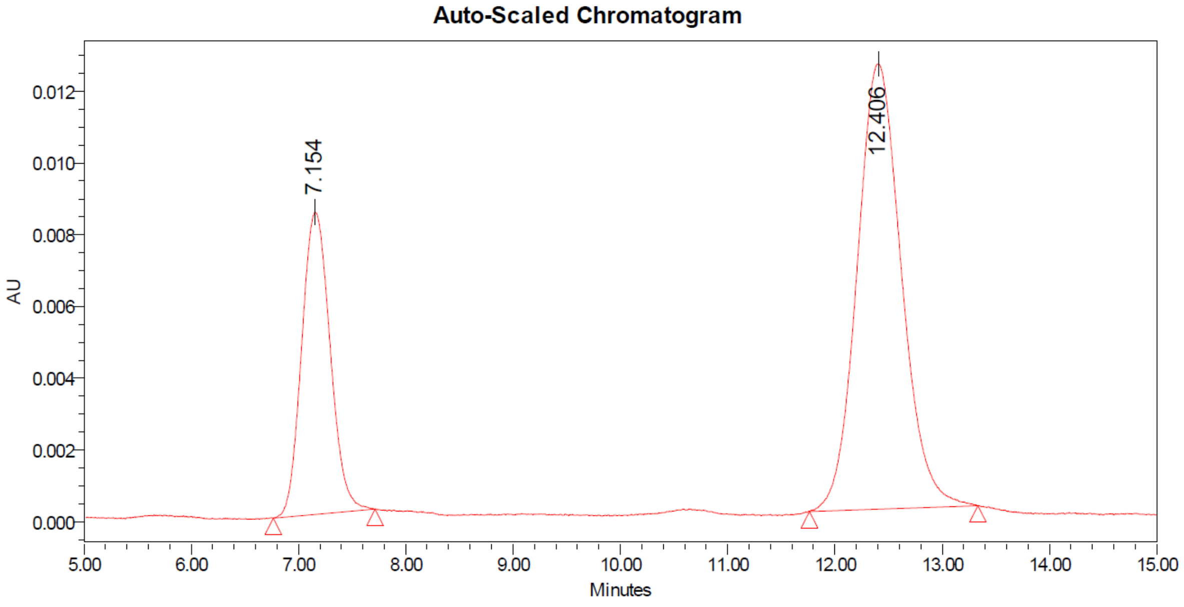

Peak Results

	Name	RT	Height	Area	% Area
1		7.154	8429	155290	30.38
2		12.406	12416	355952	69.62

505 nm

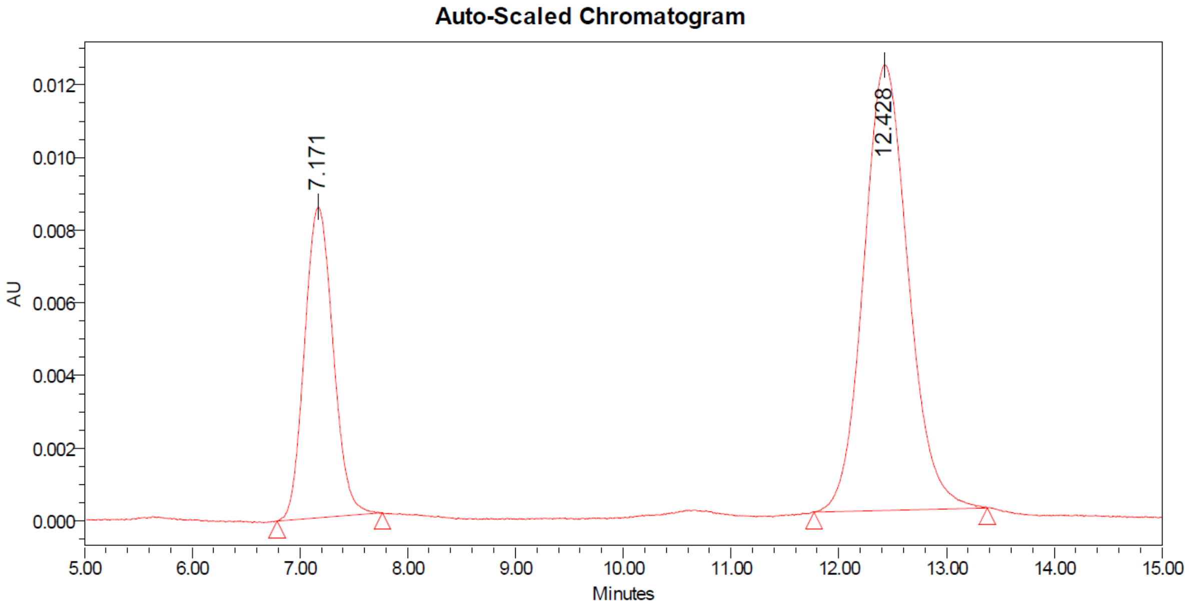

Peak Results

	Name	RT	Height	Area	% Area
1		7.171	8559	157751	30.86
2		12.428	12263	353490	69.14

530 nm

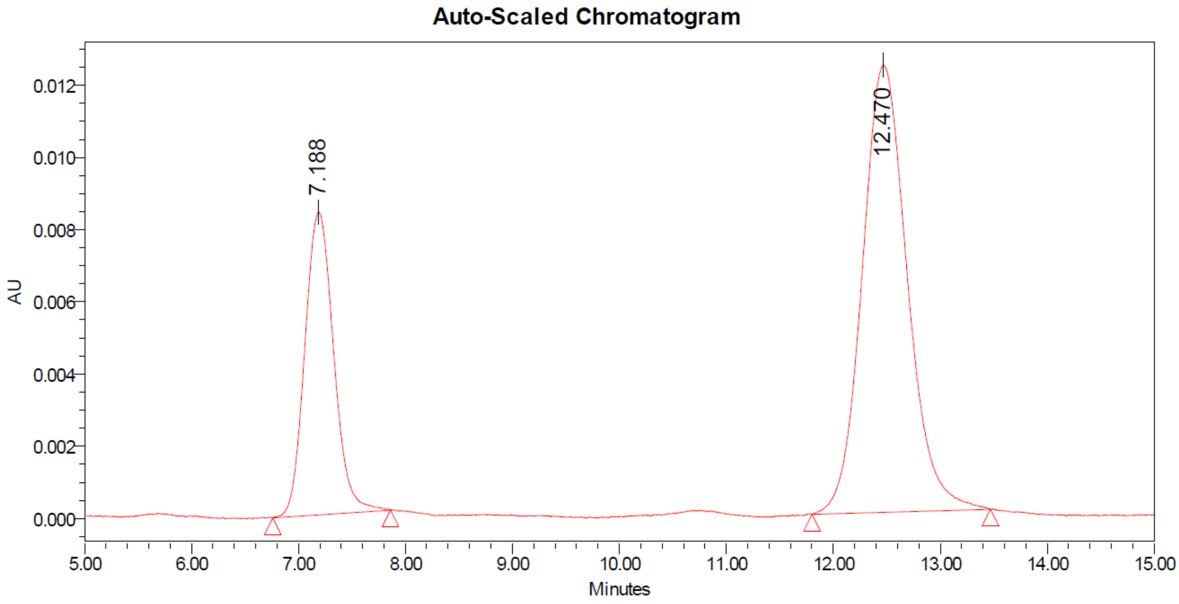

Peak Results

	Name	RT	Height	Area	% Area
1		7.188	8386	157218	30.25
2		12.470	12396	362574	69.75

4n

340 nm

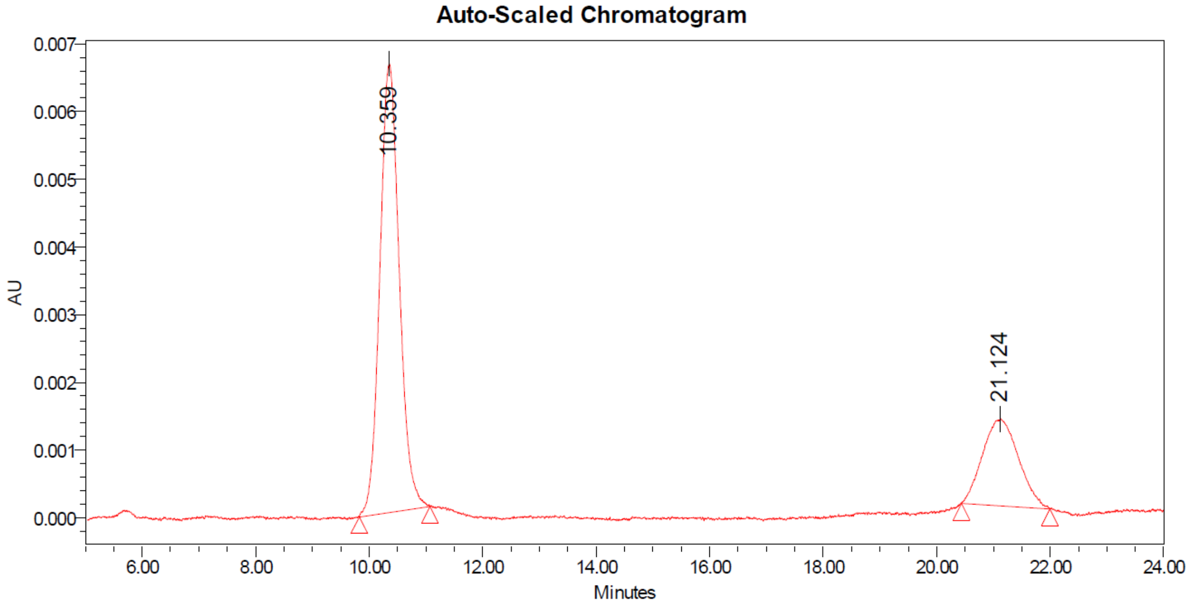

Peak Results

	Name	RT	Height	Area	% Area
1		10.359	6632	157733	73.61
2		21.124	1288	56535	26.39

365 nm

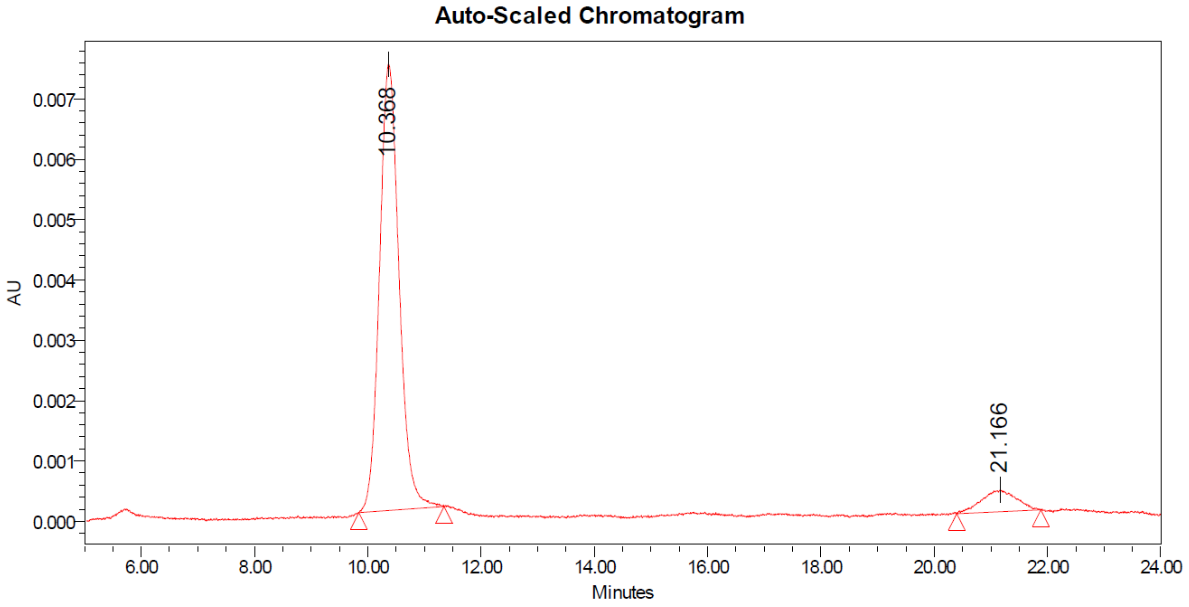

Peak Results

	Name	RT	Height	Area	% Area
1		10.368	7396	177693	92.07
2		21.166	360	15301	7.93

415 nm

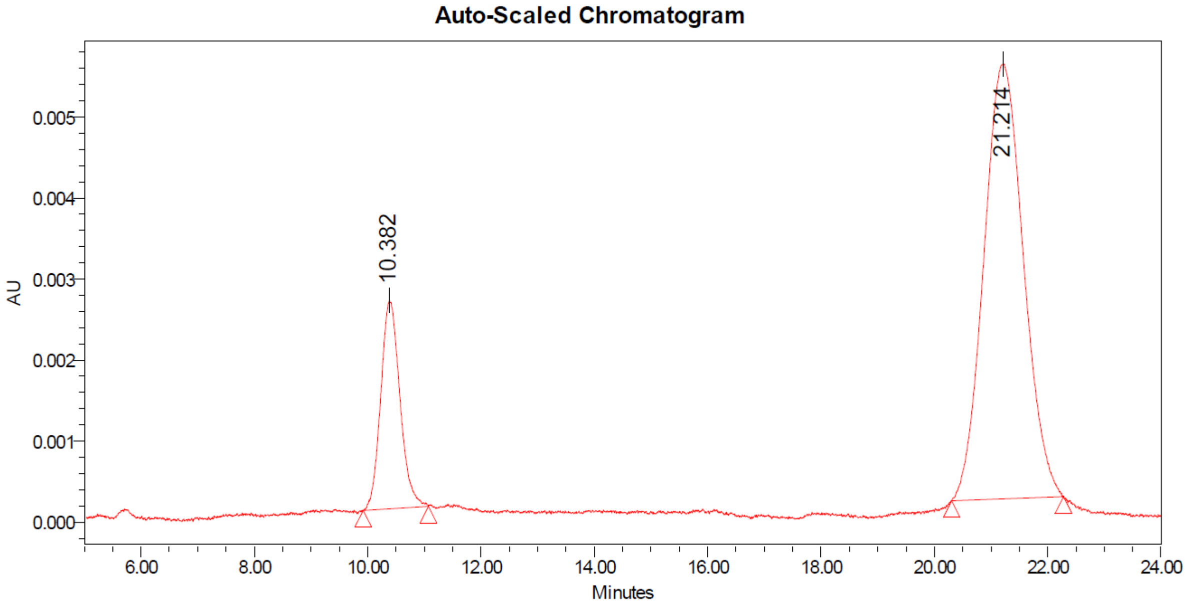

Peak Results

	Name	RT	Height	Area	% Area
1		10.382	2573	60461	18.82
2		21.214	5369	260781	81.18

455 nm

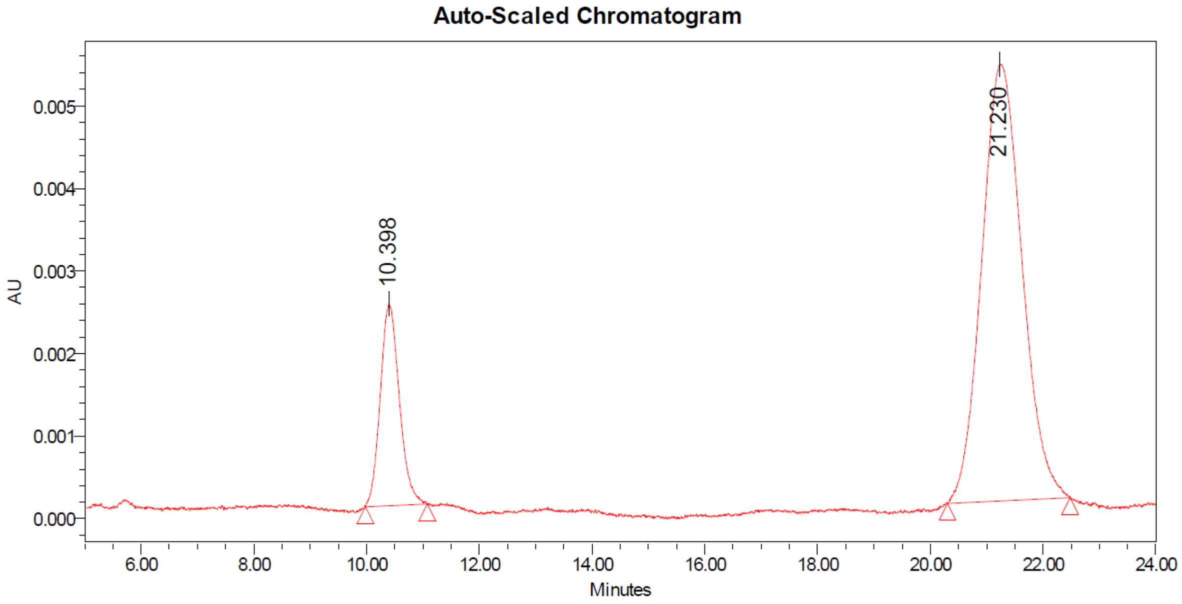

Peak Results

	Name	RT	Height	Area	% Area
1		10.398	2447	57583	17.98
2		21.230	5291	262643	82.02

505 nm

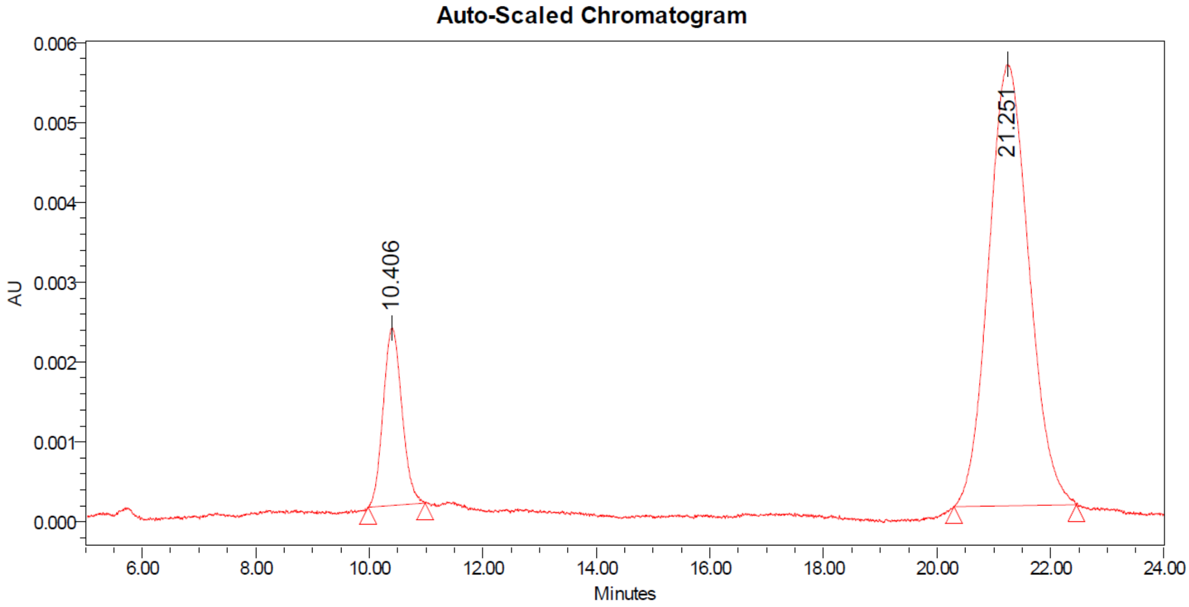

Peak Results

	Name	RT	Height	Area	% Area
1		10.406	2226	51045	15.74
2		21.251	5532	273328	84.26

530 nm

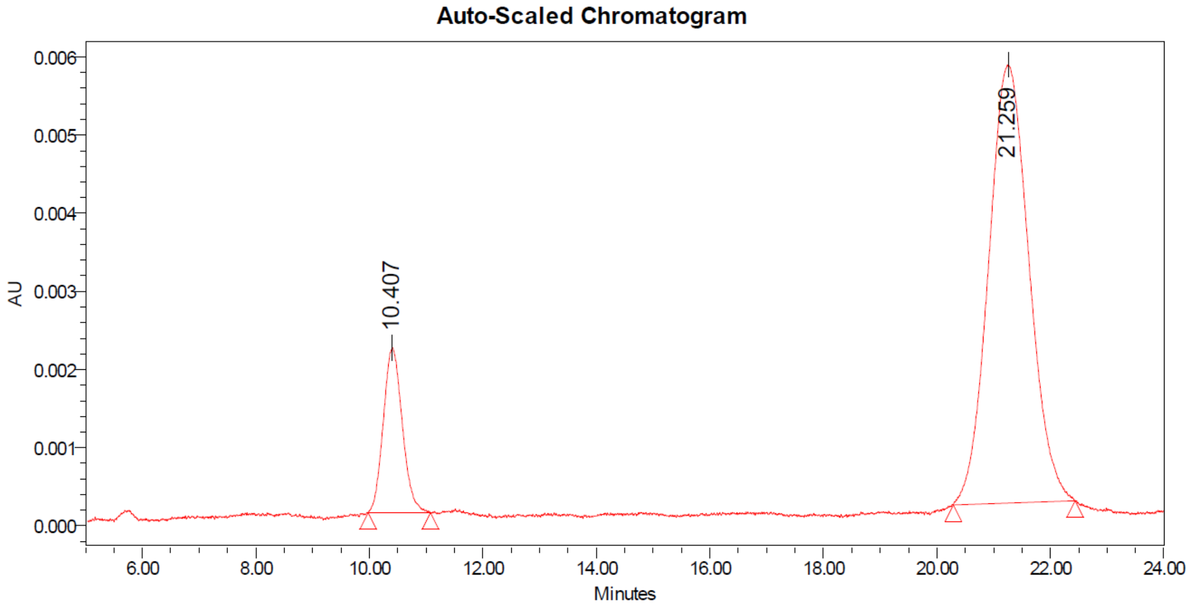

Peak Results

	Name	RT	Height	Area	% Area
1		10.407	211[illegible]	49058	15.05
2		21.259	5608	276832	84.95

4o

340 nm

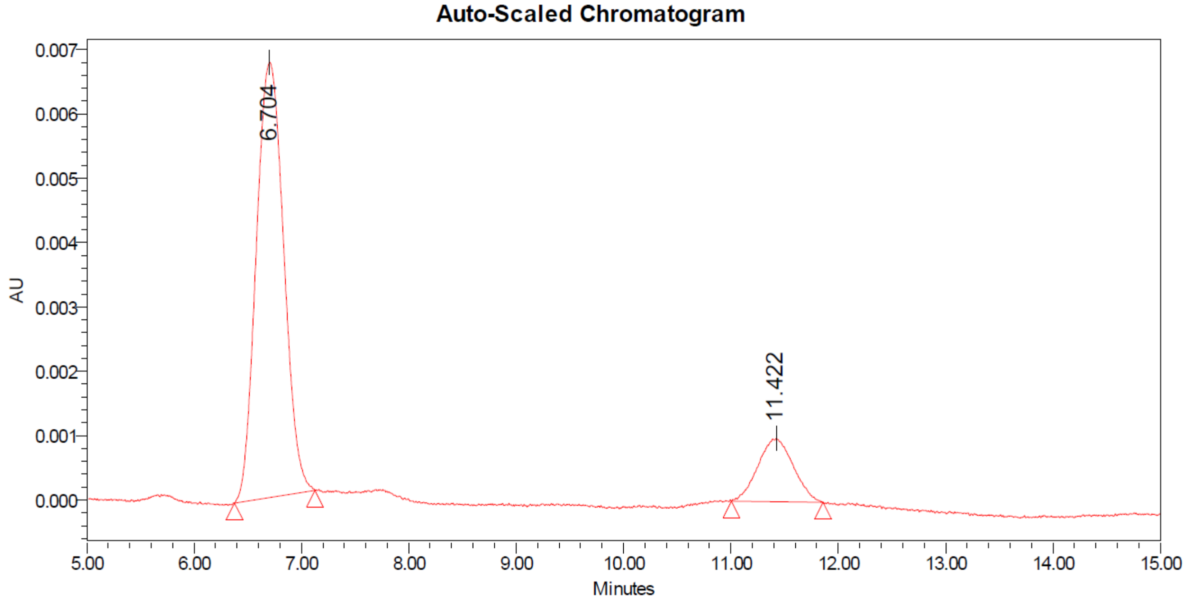

Peak Results

	Name	RT	Height	Area	% Area
1		6.704	6765	120476	84.29
2		11.422	981	22459	15.71

365 nm

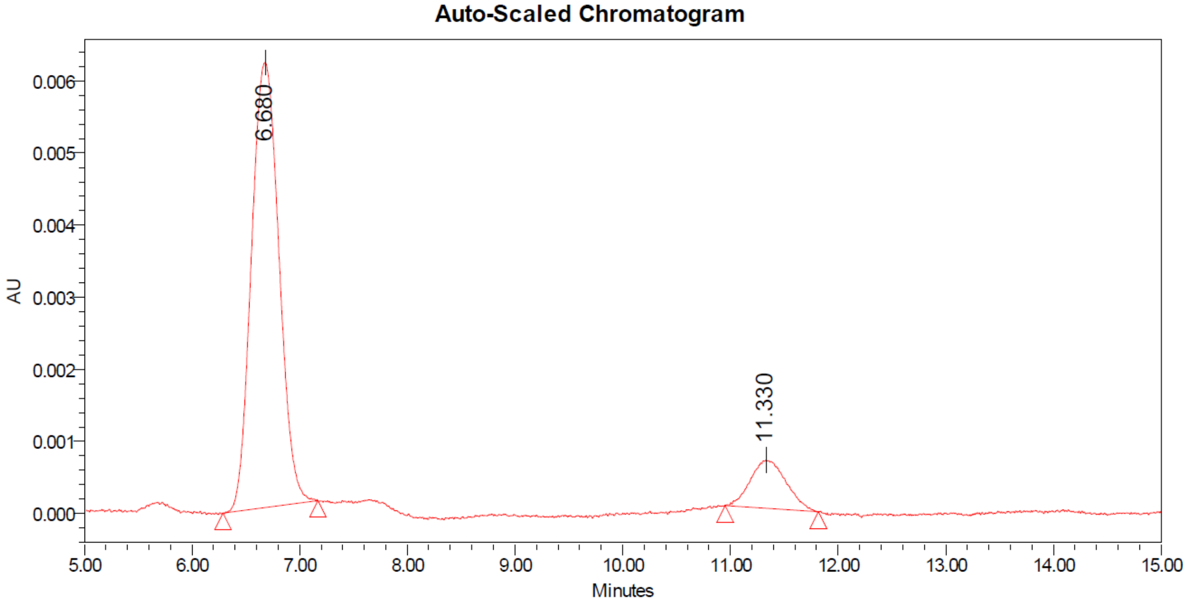

Peak Results

	Name	RT	Height	Area	% Area
1		6.680	6173	109772	87.62
2		11.330	669	15509	12.38

415 nm

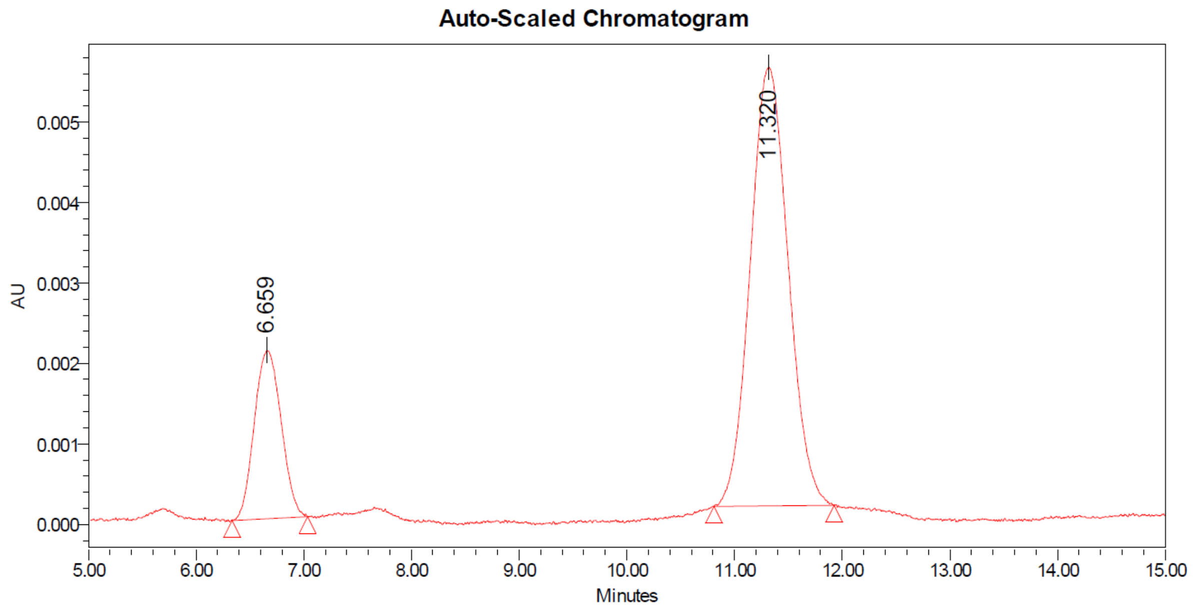

Peak Results

	Name	RT	Height	Area	% Area
1		6.659	2095	36299	21.34
2		11.320	5454	133796	78.66

455 nm

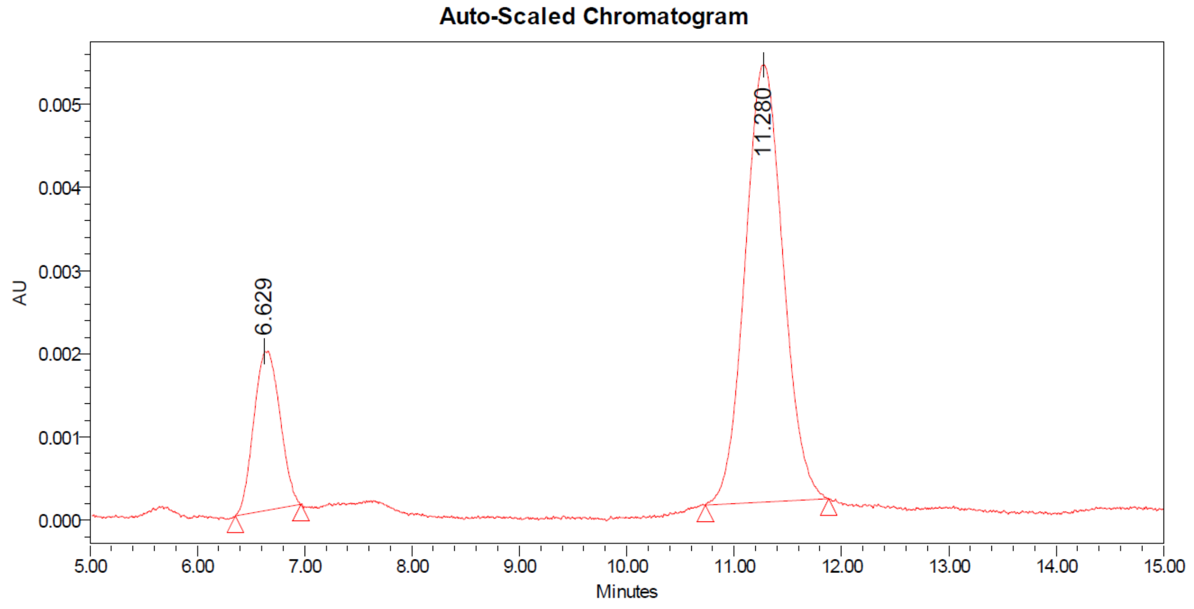

Peak Results

	Name	RT	Height	Area	% Area
1		6.629	1916	32477	20.01
2		11.280	5261	129806	79.99

505 nm

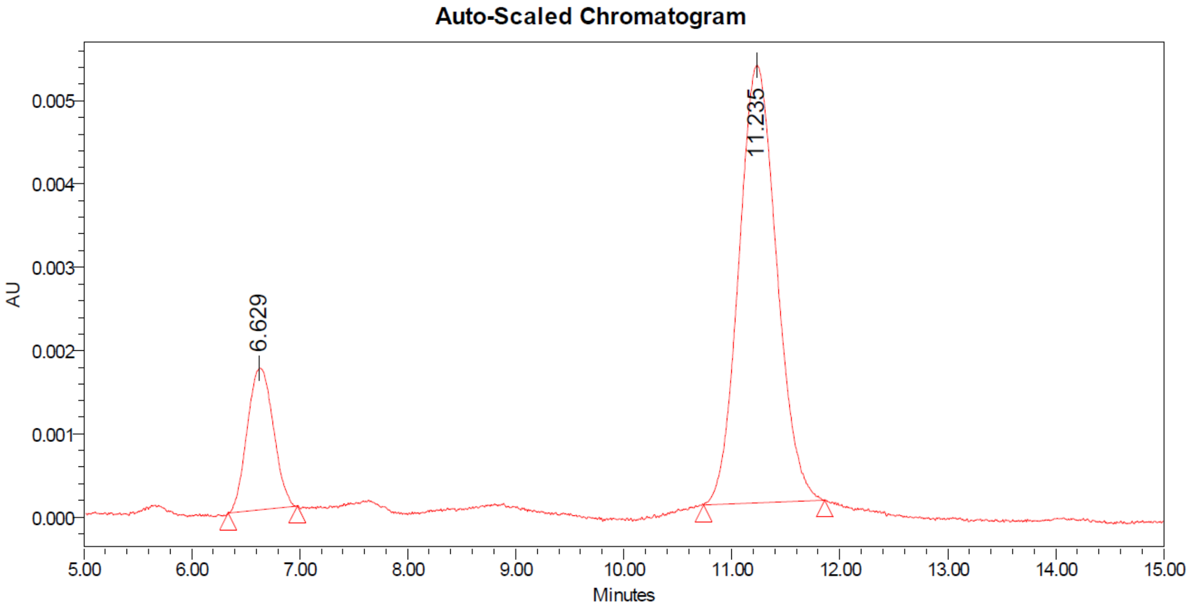

Peak Results

	Name	RT	Height	Area	% Area
1		6.629	1700	28635	18.29
2		11.235	5257	127919	81.71

530 nm

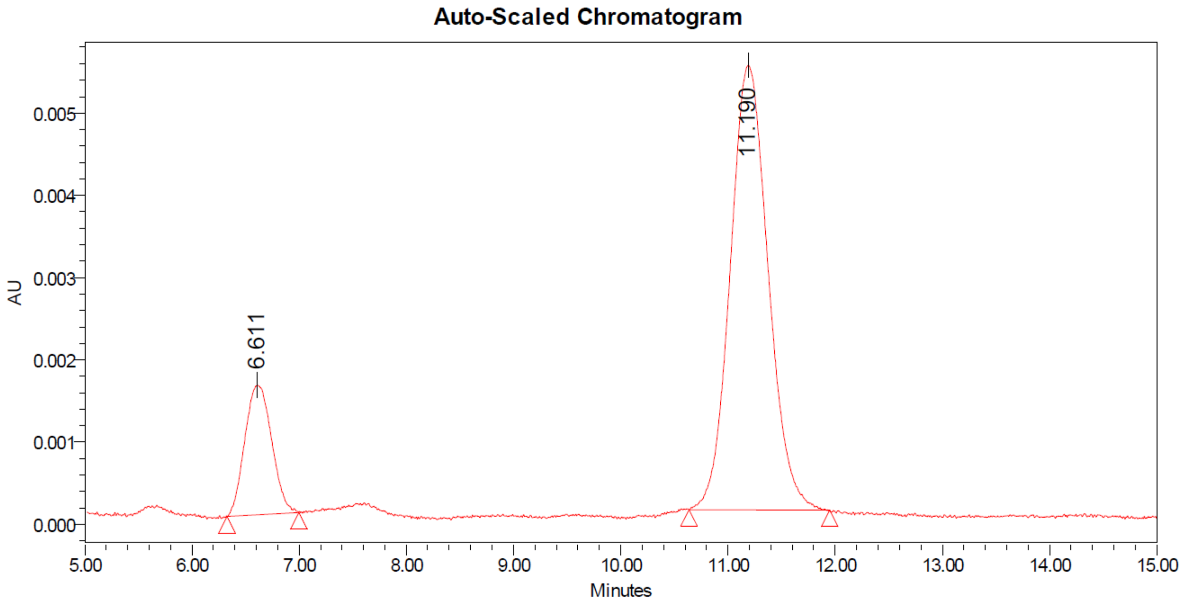

Peak Results

	Name	RT	Height	Area	% Area
1		6.611	1575	27515	17.15
2		11.190	5404	132952	82.85

7b

365 nm

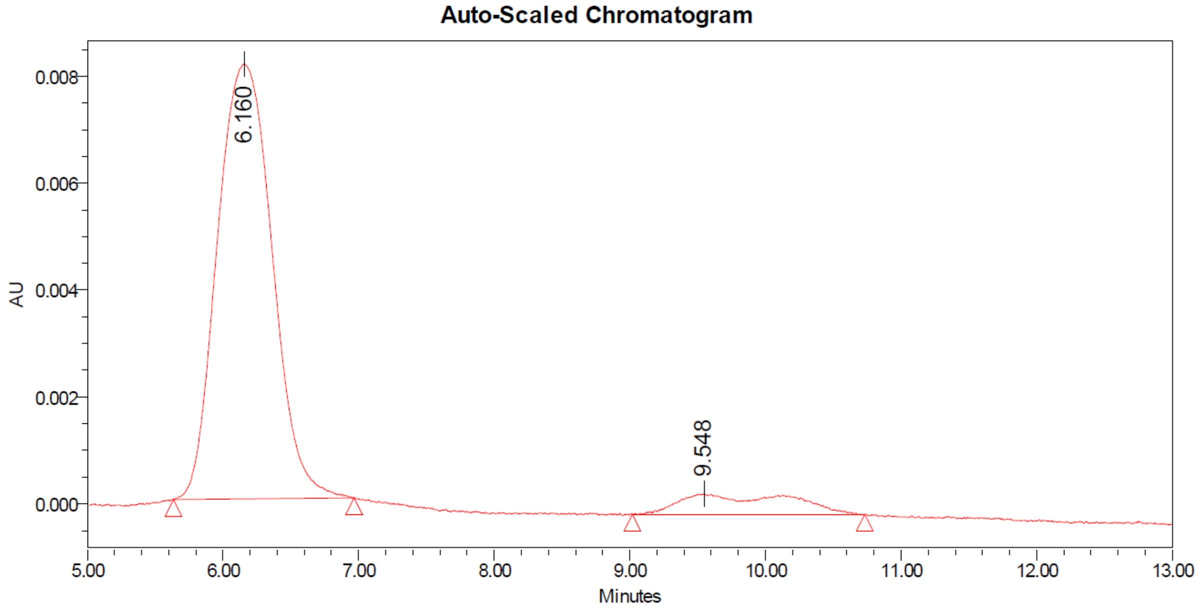

Peak Results

	Name	RT	Height	Area	% Area
1		6.160	8143	228898	91.36
2		9.548	384	21641	8.64

Note: Peak splitting observed due to 1:1 diastereomeric mixture originating from racemic lenalidomide.

1h after irradiation at 365 nm

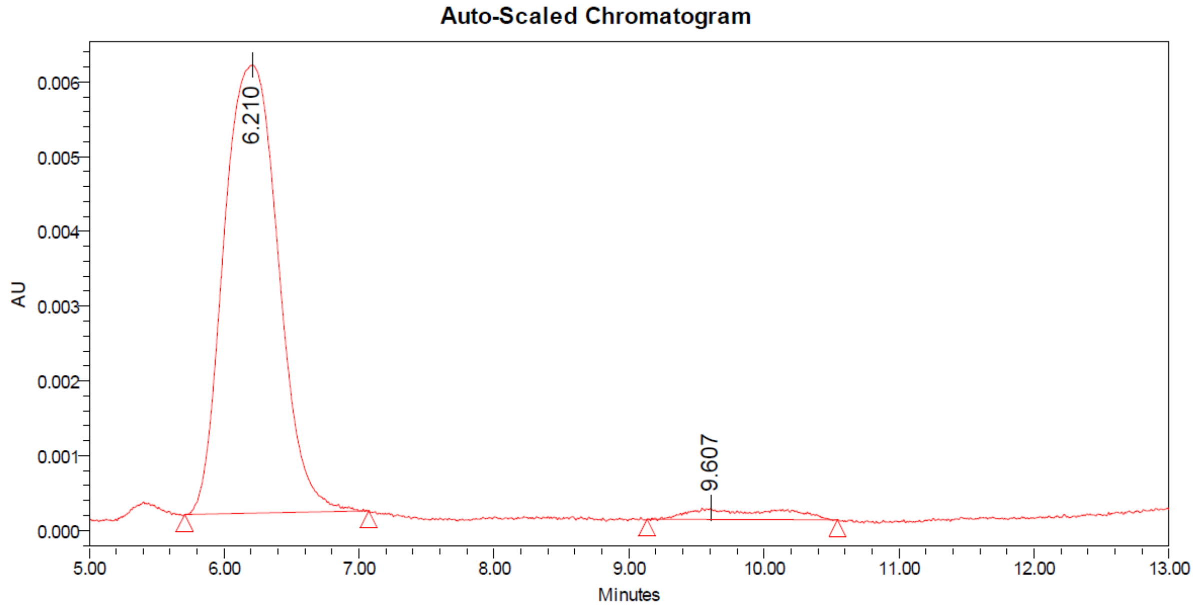

Peak Results

	Name	RT	Height	Area	% Area
1		6.210	5997	168130	96.05
2		9.607	152	6923	3.95

2h after irradiation at 365 nm

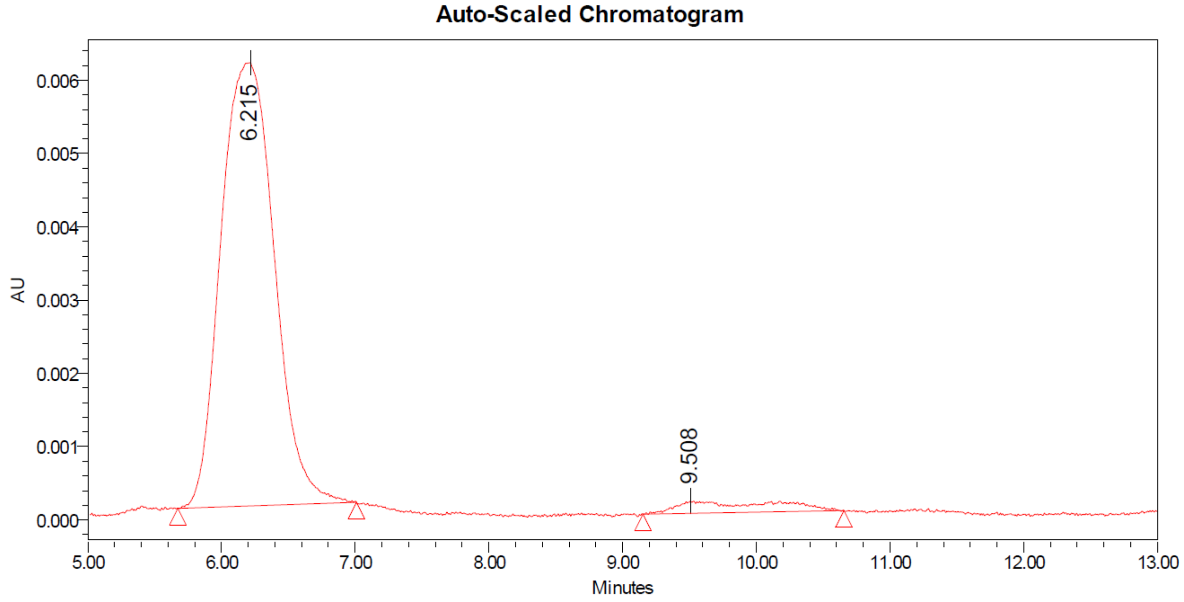

Peak Results

	Name	RT	Height	Area	% Area
1		6.215	6050	168338	95.39
2		9.508	167	8144	4.61

3h after irradiation at 365 nm

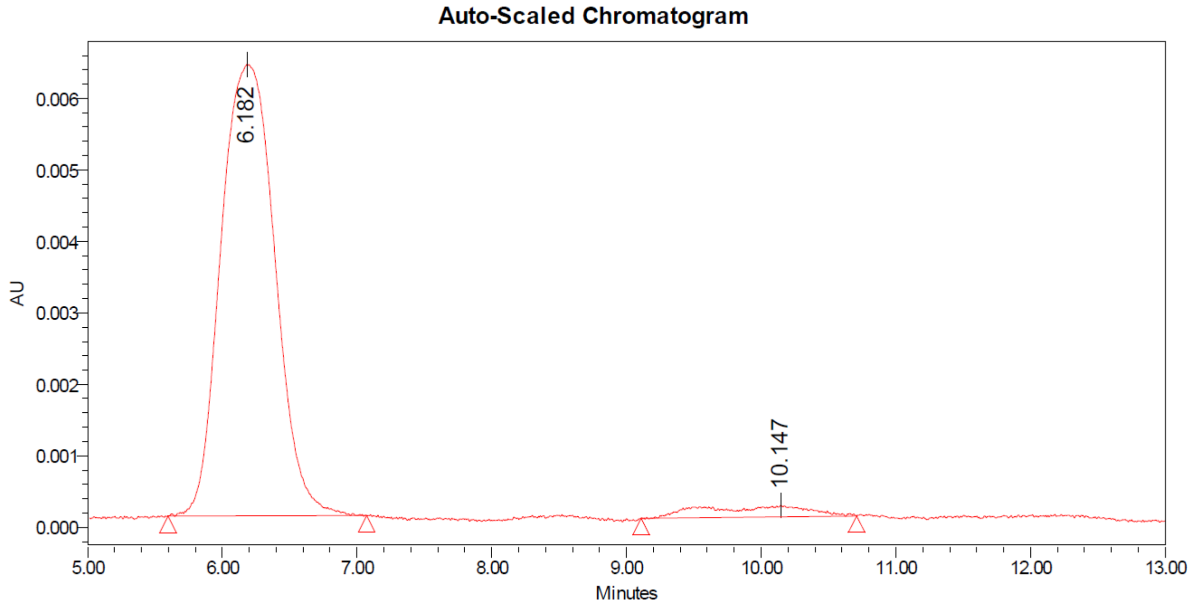

Peak Results

	Name	RT	Height	Area	% Area
1		6.182	6317	176102	95.36
2		10.147	157	8577	4.64

6h after irradiation at 365 nm

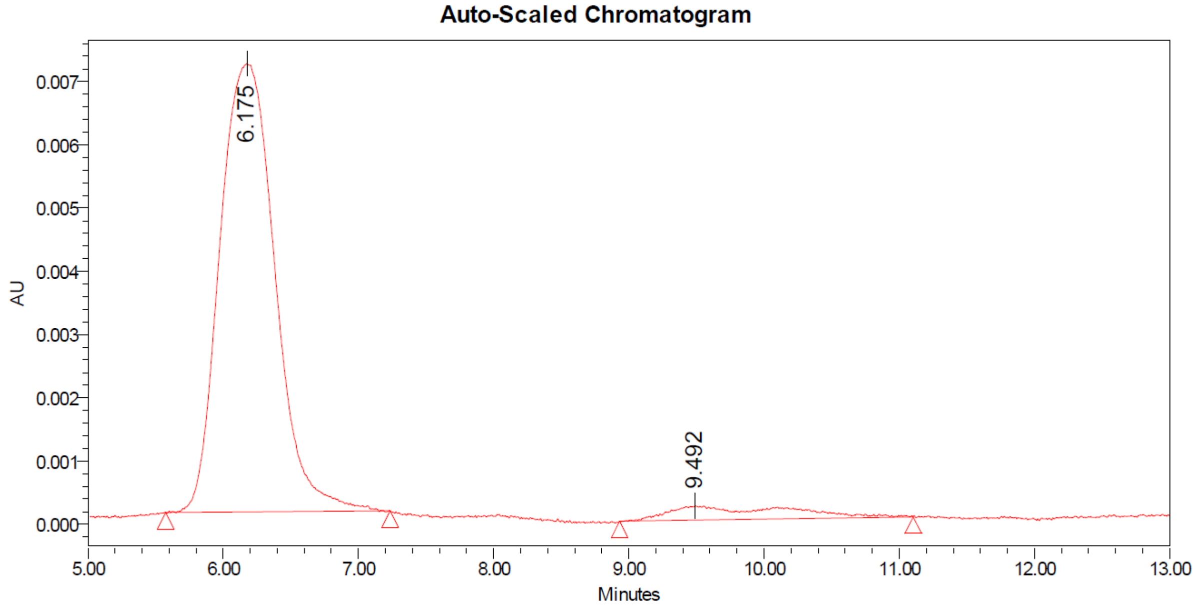

Peak Results

	Name	RT	Height	Area	% Area
1		6.175	7092	200386	93.75
2		9.492	227	13364	6.25

12h after irradiation at 365 nm

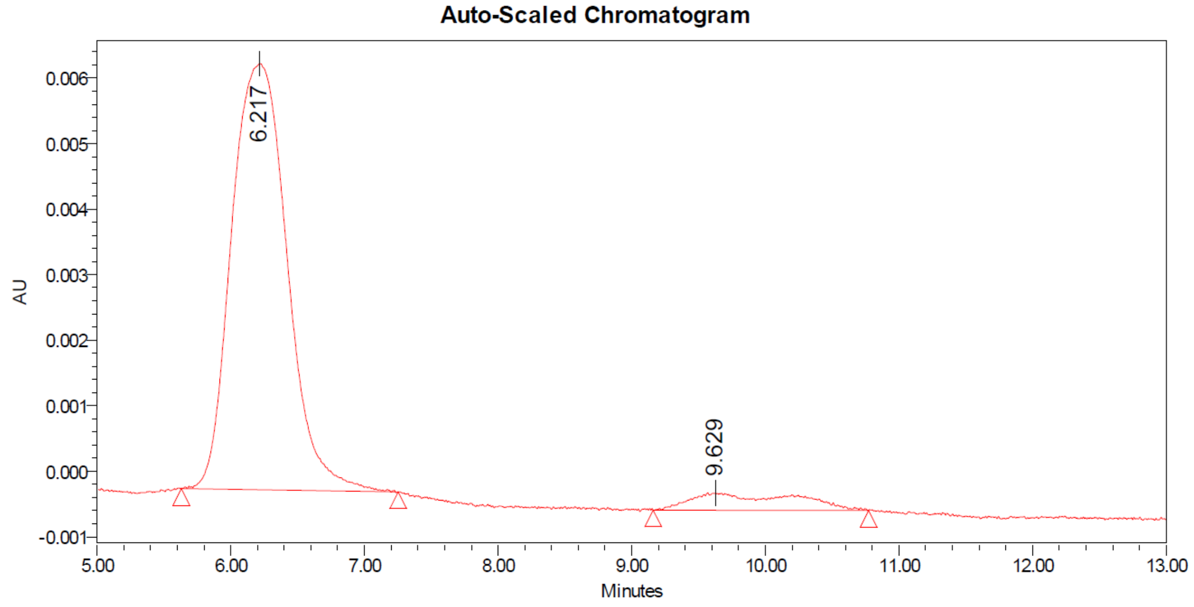

Peak Results

	Name	RT	Height	Area	% Area
1		6.217	6506	188122	92.95
2		9.629	264	14270	7.05

24h after irradiation at 365 nm

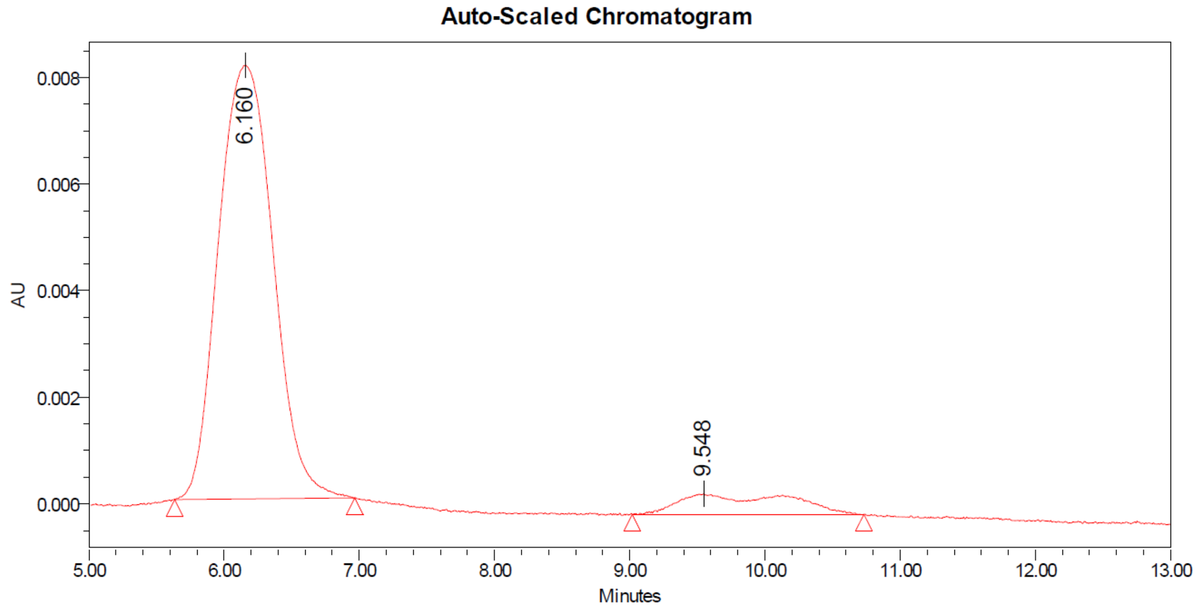

Peak Results

	Name	RT	Height	Area	% Area
1		6.160	8143	228898	91.36
2		9.548	384	21641	8.64

36h after irradiation at 365 nm

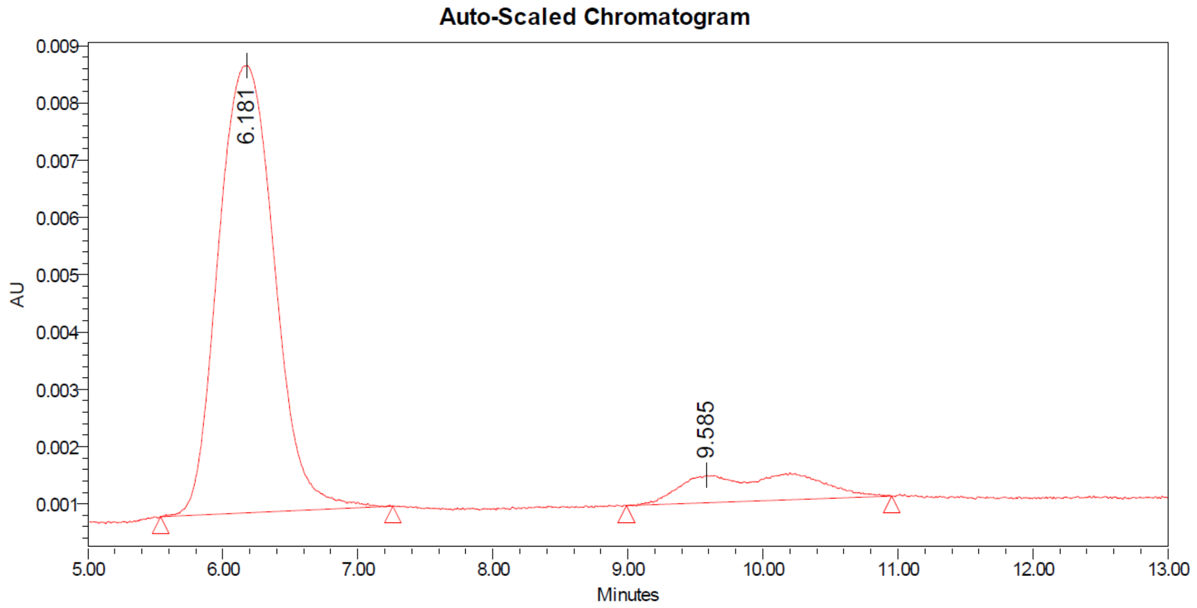

Peak Results

	Name	RT	Height	Area	% Area
1		6.181	7810	225846	88.08
2		9.585	477	30568	11.92

530 nm

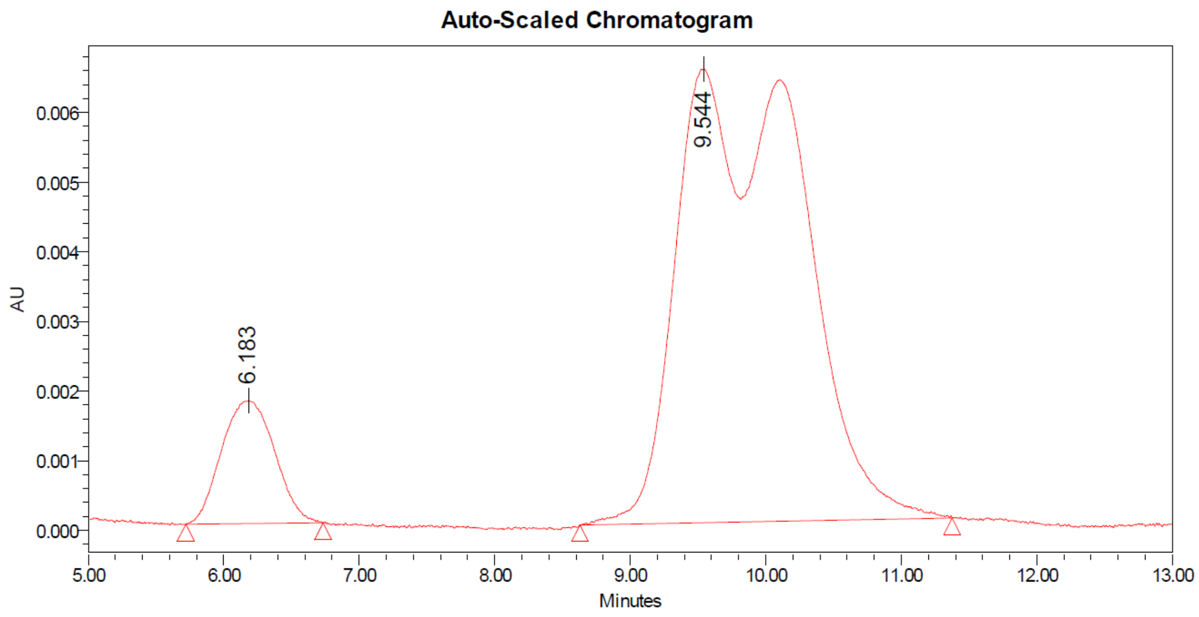

Peak Results

	Name	RT	Height	Area	% Area
1		6.183	1767	48154	10.35
2		9.544	6514	417104	89.65

Note: Peak splitting observed due to 1:1 diastereomeric mixture originating from racemic lenalidomide.
